# Epigenetic interplay between mouse endogenous retroviruses and host genes

**DOI:** 10.1186/gb-2012-13-10-r89

**Published:** 2012-10-03

**Authors:** Rita Rebollo, Katharine Miceli-Royer, Ying Zhang, Sharareh Farivar, Liane Gagnier, Dixie L Mager

**Affiliations:** 1 Terry Fox Laboratory, British Columbia Cancer Agency, 675 West 10th Avenue, Vancouver, BC, V5Z1L3, Canada; 2 Department of Medical Genetics, Faculty of Medicine, University of British Columbia, 675 West 10th Avenue, Vancouver, BC, V5Z1L3, Canada

**Keywords:** DNA methylation, epigenetics, evolution, heterochromatin spreading, mouse endogenous retroviruses, transposable element

## Abstract

**Background:**

Transposable elements are often the targets of repressive epigenetic modifications such as DNA methylation that, in theory, have the potential to spread toward nearby genes and induce epigenetic silencing. To better understand the role of DNA methylation in the relationship between transposable elements and genes, we assessed the methylation state of mouse endogenous retroviruses (ERVs) located near genes.

**Results:**

We found that ERVs of the ETn/MusD family show decreased DNA methylation when near transcription start sites in tissues where the nearby gene is expressed. ERVs belonging to the IAP family, however, are generally heavily methylated, regardless of the genomic environment and the tissue studied. Furthermore, we found full-length ETn and IAP copies that display differential DNA methylation between their two long terminal repeats (LTRs), suggesting that the environment surrounding gene promoters can prevent methylation of the nearby LTR. Spreading from methylated ERV copies to nearby genes was rarely observed, with the regions between the ERVs and genes apparently acting as a boundary, enriched in H3K4me3 and CTCF, which possibly protects the unmethylated gene promoter. Furthermore, the flanking regions of unmethylated ERV copies harbor H3K4me3, consistent with spreading of euchromatin from the host gene toward ERV insertions.

**Conclusions:**

We have shown that spreading of DNA methylation from ERV copies toward active gene promoters is rare. We provide evidence that genes can be protected from ERV-induced heterochromatin spreading by either blocking the invasion of repressive marks or by spreading euchromatin toward the ERV copy.

## Background

Transposable elements (TEs) are DNA sequences able to move from one chromosome location to another, either through an RNA intermediate (retrotransposons) or simply by excising their DNA copies (DNA transposons). Retrotransposons can be further classified into long terminal repeat (LTR)-containing TEs (LTR retrotransposons and endogenous retroviruses (ERV)) or non-LTR retrotransposons (long and short interspersed nuclear elements, LINEs and SINEs). Because of the multiple mechanisms by which TEs can affect host genes [1,2], TEs are tightly regulated by specific host machineries, including epigenetic mechanisms such as DNA methylation. In plants, it has been shown that mutants of the DNA methylation machinery induce bursts of transposition of usually silenced TE copies [3]. In *Dnmt1*-deficient mouse embryos (lacking maintenance of DNA methylation), unmethylated copies of Intracisternal (A) Particles (IAPs, a family of ERVs) are observed along with a significant accumulation of transcripts [4].

Because TEs are abundant and present throughout the genome, their epigenetic silencing might influence host genes through spreading of repressive chromatin marks [5]. DNA methylation has been shown to spread from TE copies to nearby genes in very few cases, with elegant examples in plants regarding *Arabidopsis thaliana *vernalization regulation [6] and melon sex determination [7]. In mammals, it has been suggested that DNA methylation spreads into the mouse *Aprt *and rat *Afp *genes via nearby methylated SINE copies [8-10] and we have recently shown one example of spreading of heterochromatin (histone H3 trimethylation of lysine 9 (H3K9me3) and DNA methylation) from an ERV LTR to a gene promoter in mouse embryonic stem (ES) cells [5]. With the paucity of well-documented examples of spreading of DNA methylation into nearby genes, the impact of TE epigenetic regulation on genome dynamics remains unknown. In *Arabidopsis*, DNA methylation of TE copies is influenced by the genomic environment, as copies near genes are hypomethylated compared with copies far from genes [11]. However, insertionally polymorphic copies between *Arabidopsis *ecotypes do not show any bias in DNA methylation when near genes, suggesting a loss of methylation or a loss of methylated copies over time [11]. These data provide evidence for negative selection against methylated TE insertions near genes, possibly because of the harmful impact on host genes through spreading of DNA methylation. Nevertheless, no information concerning TE family, orientation, and location relative to genes (upstream, inside, downstream) was reported in the *Arabidopsis *study, therefore generalizing a result that might be confined to specific situations. Moreover, in mammals, whereas spreading of DNA methylation remains rarely described, further work is necessary to understand host gene-TE relationships.

The goal of the present study was therefore to understand the epigenetic interactions between ERVs and host genes in a mammalian system. IAPs and Early transposon/*Mus musculus *type D (ETn/MusDs) are two families of mouse ERVs known to be repressed by DNA methylation [4,12] and are responsible for the majority of new insertional mutations in mice [13]. We first asked if the genomic environment, that is, the distance between ERVs and host genes, influences the DNA methylation state of IAP and ETn/MusD copies. Interestingly, we found that most ERV copies are heavily methylated regardless of their genomic environment, with the exception of some ETn/MusD copies which were unmethylated when near transcription start sites (TSSs) of genes. Hence we wondered if any spreading of DNA methylation occurred from the methylated ERV copy into the gene promoter. Such spreading was rarely observed and this observation led us to hypothesize that the DNA sequences located between the methylated ERVs and the nearby genes could act as boundary regions. Consequently, we studied the chromatin environment of these boundary regions. Our data suggest that gene promoters are shielded from such spreading by euchromatic domains enriched in H3K4me3 and CCCTC-binding factor (CTCF), which, in turn, can spread toward nearby ERVs and maintain them in an unmethylated state.

## Results and discussion

### Endogenous retrovirus copies are rare near genes

**Figure 1:**
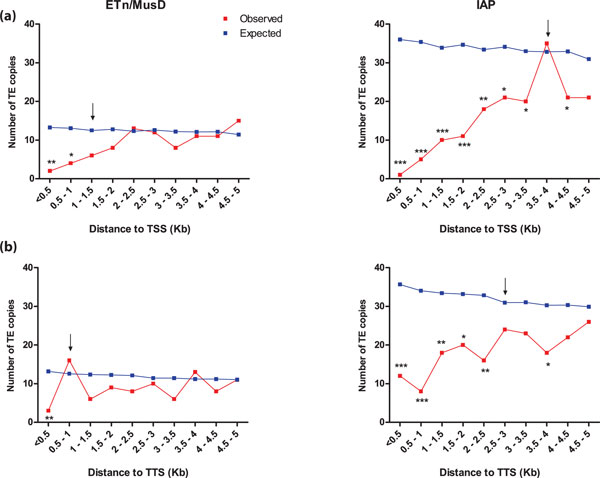
**Distribution of endogenous retrovirus copies in the mouse reference C57Bl/6 genome**. The observed distribution of ERV copies is compared with the expected pattern if these elements were randomly distributed. ERV distribution with regard to gene **(A) **TSSs or **(B) **TTSs. Arrows indicates the maximum distance between an ERV and a gene that is under negative selection based on our statistical analysis. A proportion equality test allowed us to compare both distributions and uncover significant differences. ***P <0.001, **P <0.01 and *P <0.05. ERV copies can be located upstream, inside or downstream of genes. ERV: endogenous retrovirus; TSS: transcription start site; TTS: transcription termination site.

We first analyzed the genomic distributions of IAP and ETn/MusD elements near genes to identify regions where they are underrepresented compared with expectations. Since the initial insertion site preferences for these ERV families are unknown, we assumed a random integration pattern to generate the expected distribution profiles. Specifically, we determined the distribution of annotated ERVs relative to the TSS or transcription termination site (TTS) of RefSeq annotated genes and identified underrepresentation zones that likely reflect the effects of selection against ERVs that insert in these zones. As expected, copies near TSSs are underrepresented for both ERV families, with putative harmful ERV-TSS distances of 1.5 kb and 4 kb for ETn/MusD and IAP copies respectively (Figure 1A). For subsequent analysis, we used the distance threshold of the first bin that was statistically not significant between expected and observed distributions. Curiously, ETn/MusD within 1 kb and IAP within 3 kb of TTSs are also underrepresented compared with the expected distribution (Figure 1B). An overrepresentation of ETn/MusD at 0.5 to 1 kb of TTSs was observed but was not statistically significant. Assuming that IAP and ETn/MusD elements initially insert randomly in the genome, these data suggest a negative selective pressure on ERV copies close to the 5' and 3' termini of genes.

### ETn/MusDs show variable methylation when near transcription start sites

ETn/MusD and IAP copies are often the target of DNA methylation and other repressive chromatin marks [5,14,15]. We asked if copies close to genes (TSSs and TTSs) have the same DNA methylation pattern as copies located far from genes. We used the ERV distribution generated above to separate our dataset into two large classes: those near and those far from genes. Among those close to genes, we checked that both the gene and ERV were correctly annotated and that gene expression data was available (for more information see Materials and methods). Out of 15 ETn/MusD copies extracted from the sequenced genome within 1.5 kb of TSSs, only seven copies passed all our filters for further DNA methylation analysis (Additional file 1). We studied all seven of these ETn/MusD copies. Out of 124 IAPs within 4 kb of TSSs, 82 passed the filtering steps and 24 of these were studied. We prioritized the study of copies closest to gene TSSs (14 IAP copies studied out of 18 copies available after filtering are within 2 kb of TSSs) and that are insertionally polymorphic, based on our previous study [16], so allele-specific analysis could be performed if necessary. We added three insertionally polymorphic copies to our dataset of IAP copies that were absent from the reference C57BL/6 genome but present in other strains because of their close proximity to TSSs (nearby genes *B3galtl *(368 bp), *Gdpd3 *(437 bp), and *Eps15 *(1613 bp)). Additionally, a random set of ETn/MusD and IAP copies far from RefSeq genes were selected for further DNA methylation analysis. Hence, despite analyzing only 30% of the entire dataset available for IAP copies, we believe our sampling represents a genome-wide analysis of copies close to genes for both ERV families. In total, we selected 80 ETn/MusD and IAP copies, of which 34 are close to genes, for further analysis (see Additional file 2 for entire dataset, with detailed information on each copy studied).

DNA methylation of the 34 ERVs close to genes was studied among one of the tissues (liver, spleen, kidney, pancreas or testis) where the gene was expressed (as determined by GNF Expression Atlas microarray dataset [17,18]). To study the DNA methylation of such a high number of copies in a variety of tissues we opted for a method using methylated DNA immunoprecipitation (MeDIP) followed by quantitative PCR (qPCR). The observed methylation status of all copies was confirmed by bisulfite sequencing (comparison between methylation data from bisulfite sequencing with MeDIP-qPCR shows a Spearman r = 0.87, *P *<0.0001), or by a second qPCR primer pair used in two new biological replicates (Spearman r = 0.82, *P *<0.0001) or by COBRA, a method involving bisulfite treatment and restriction enzyme digestion (four copies only). Every copy determined to be unmethylated by MeDIP was also validated by bisulfite sequencing. There were no significant differences in the overall DNA methylation of copies between tissues (Figure S1 in Additional file 3) and mouse strains used (C57BL/6 versus A/J Spearman r = 0.82, *P *<0.0001).

**Figure 2:**
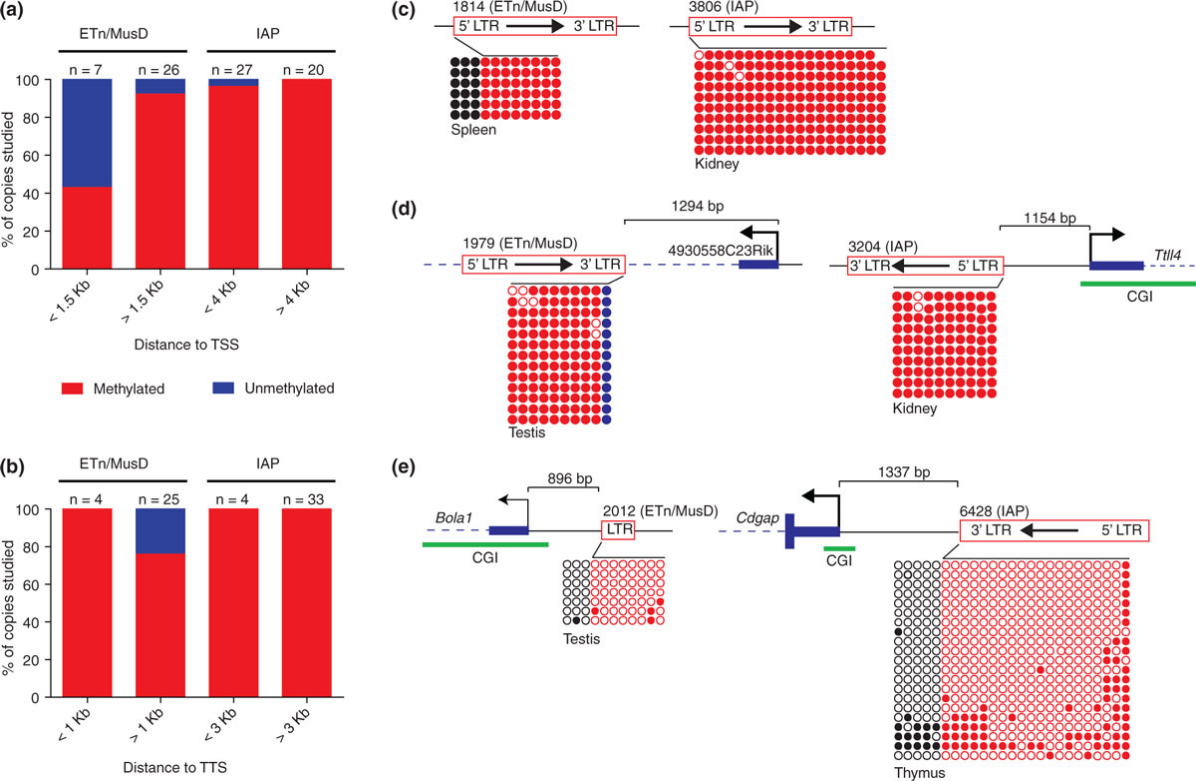
**Global methylation analysis of endogenous retrovirus copies nearby and distant from genes**. **(A) **DNA methylation status of ERV copies relative to their TSS or **(B) **TTS (same dataset of copies for both panels. Note that fewer copies are present in panel B as in some cases the LTR analyzed was not the LTR closest to the TTS. The number of total copies studied in each category (n) is indicated above the bars. Distances were chosen based on Figure 1. Examples of bisulfite sequencing of copies **(C) **far from or **(D,E) **close to genes. The following cartoon legend applies for all figures: blue dashed lines represent introns and an arrow inside the ERV copy indicates the ERV sense of transcription. Empty circles represent unmethylated CpGs and filled ones are methylated CpGs (red for ERVs, blue for genes and black for flanking sequences). Each row of CpGs represents one sequenced bisulfite clone and each block of CpGs represents one sample. The tissue where the copy was studied is depicted below each block. For all DNA methylation data see Additional file 2, Figures S1 (MeDIP) and S2 (bisulfite) in Additional file 3. bp: base pairs; CGI: CpG Island; ERV: endogenous retrovirus; ETn/MusD: Early transposon/*Mus musculus *type D; IAP: Intracisternal (A) Particle; LTR: long terminal repeat; TSS: transcription start site; TTS: transcription termination site.

Nearly all copies analyzed, regardless of distance to a gene, were methylated (see Additional file 2 for entire dataset and Figure S2 in Additional file 3 for bisulfite sequences). However, four of the seven available ETn/MusD elements close to TSSs were unmethylated, while almost all IAP copies were methylated regardless of their genomic environment, with the exception of one copy (Figure 2A, C-E). Variation in the DNA methylation state of IAP copies has been observed previously [19], notably in mice carrying the insertionally polymorphic IAP element responsible for the A^vy ^mutation [20]. Nevertheless, in agreement with our previous findings [5,15], we observed a consistent association between IAP elements and repressive epigenetic marks whereas ETn elements close to genes show variable associations. Since ETn/MusD and IAP are active mouse ERV families, insertionally polymorphic copies exist between different strains [16,21,22]. The only unmethylated IAP copy observed in our analysis (close to the *Cdgap *gene, in thymus (Figure 2E), brain and lung (Figure S2 in Additional file 3)) is present in only one strain of mouse and absent from 17 other mouse strains studied previously [22]. The high strain-specificity suggests this IAP insertion is very recent. No differences in the methylation state of fixed and insertionally polymorphic copies studied were observed.

Interestingly, all ETn/MusD and IAP copies remain methylated when close to TTSs (Figure 2B). Therefore, while negative selection acts on copies close to genes, ERV DNA methylation does not seem to be influenced by the presence of a nearby TTS. Hence, of the two families studied here, DNA methylation of only ETn/MusD copies is generally influenced by nearby TSSs.

### Differential methylation can be observed within ERV copies

**Figure 3:**
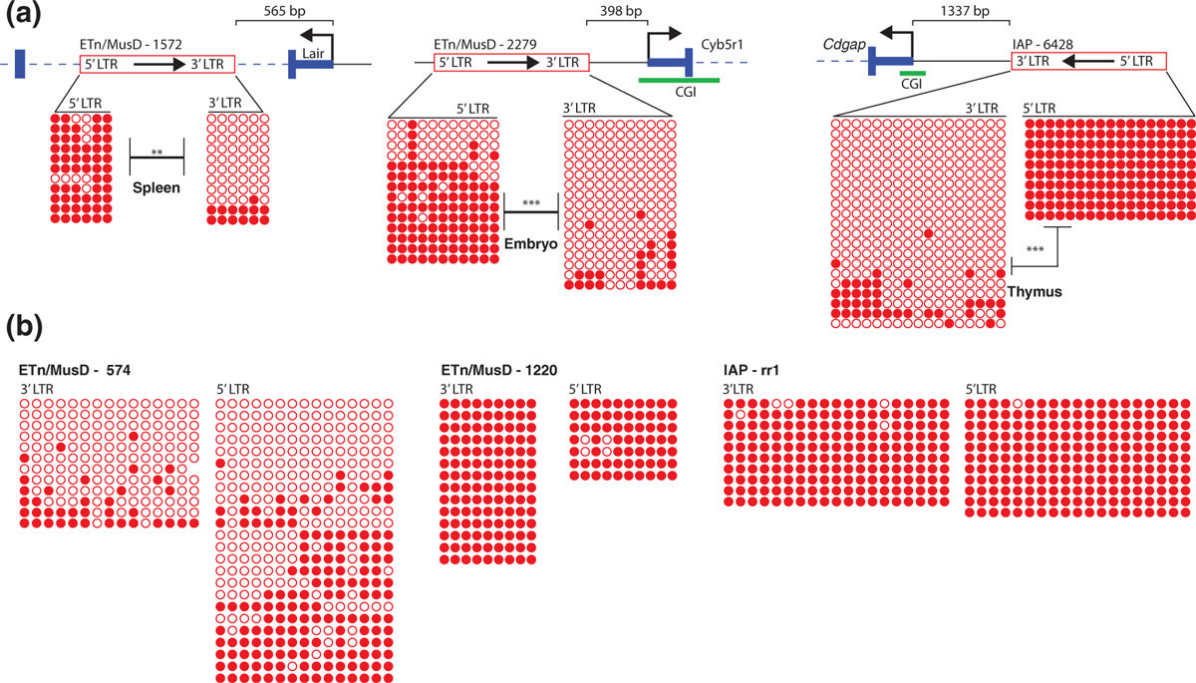
**Differential methylation within endogenous retroviruses is observed when near transcription start sites**. **(A) **DNA methylation comparison between LTRs. Cartoons show the full-length ERV copy relative to the gene studied. Three ERV copies (two ETn/MusDs, and one IAP) located close to gene TSSs show hypomethylation of the LTR near the gene TSS (3' LTR) and hypermethylation of the LTR further from the gene TSS (5' LTR). **(B) **DNA methylation analysis of 3' and 5' LTRs of ERVs far from genes. No differential methylation between LTRs is generally observed. See Figure S3 in Additional file 3 for all the data (note that the 5' LTR bisulfite sequencing was adapted from [15]. Global methylation profile was compared between both LTRs with a Mann-Whitney U-test, only significant results are shown. ***P <0.001, **P <0.01. bp: base pairs; CGI: CpG Island; ERV: endogenous retrovirus; ETn/MusD: Early transposon/*Mus musculus *type D; IAP: Intracisternal (A) Particle; LTR: long terminal repeat; TSS: transcription start site.

Out of the 34 copies studied near genes, representing all ETn/MusD copies available and 30% of IAP copies, only five were unmethylated when close to TSSs and three of these are full-length ERV copies possessing two LTRs. In all three cases, both LTRs are 100% identical and therefore do not present a DNA sequence bias. To test if methylation of these three ERV copies was influenced by the ERV-TSS distance and not dependent on the ERV DNA sequence itself, we compared the DNA methylation state of both LTRs. All three distal LTRs were significantly more methylated when compared with the LTR closer to the gene TSS (Figure 3A). We noted that, for all three cases, the 3' LTR of the ERV was the hypomethylated one. Hence, to determine if 3' LTRs are generally less methylated compared with 5' LTRs, we compared the DNA methylation status of both LTRs of eight full-length ERVs located far from TSSs (LTR-TSS distance greater than 10 kb) but not in gene deserts (Figure 3B and Figure S3 in Additional file 3). With the exception of one copy that showed less DNA methylation within the 3' LTR than the 5' LTR and another copy harboring the opposite pattern, we observed equivalent levels of methylation for both LTRs, indicating that, in general, 3' LTRs are not hypomethylated compared with 5' LTRs. While exhibiting no significant differences between LTRs, it is important to note that ETn/MusD copies were previously described as being variably methylated between individuals and cells [15] and variably associated with repressive chromatin marks [5]. Therefore, it is not surprising that in our study we also observed variable methylation of ETn/MusD throughout the genome. Li and colleagues have recently described differential methylation between a 5' LTR and a 3' LTR of a full-length insertionally polymorphic IAP copy [21]. Nevertheless, both LTRs are still heavily methylated (50% and more than 90% of DNA methylation observed) compared with the unmethylated copies we have observed in our analysis (15% for unmethylated IAP copies and 0% to 20% for ETn/MusD copies). Despite the small number of copies available for study, we clearly show that the ERV-TSS distance in mouse is associated with the unmethylated state of the copies studied.

As mentioned above, all the cases of differential LTR methylation involve hypomethylation of the 3' LTR, with the 5' LTR being heavily methylated. This scenario would be expected to silence transcription of the ERV itself, which initiates in the 5' LTR, and prevent new retrotranspositions of that particular element, regardless of the methylation status of the 3' LTR. To assess whether or not 5' or 3' LTRs are statistically more likely to lie proximal to the TSS of nearby genes, we screened all full-length IAP and ETn/MusD copies in the reference mouse genome. Indeed, no apparent bias exists for the orientation of these ERVs relative to the TSS of an adjacent gene as concluded from our genome-wide analysis (equality of proportion *P *= 0.5, Table 1). Curiously, in our dataset of copies studied close to genes, nearly all instances where a 5' LTR was proximal to the TSS of a gene, a CpG Island (CGI) was part of the gene promoter (Table 1). Indeed, 5' IAP LTRs show significant depletion near non-CGI promoters. In other words, when a 5' LTR is close to a TSS, the TSS is associated with a CGI 80% of the time, which is significantly higher than when the 3' LTR is closest to the TSS (Table 1). This means that 5' LTRs of IAPs are less likely to be found near non-CGI promoters. Unfortunately, the small dataset available of ETn/MusD copies close to genes does not allow us to analyze this ERV family in a similar way.

**Table 1:** 5' LTR distribution and methylation analyses near CpG Island associated genes

DNA methylation analysis	LTR closest to TSS (only full-length copies)	% methylated copies	% methylated copies near CGI promoters
ETn/MusD	5' LTR (n = 2)	100	100
	3' LTR (n =3)	33.3	0
IAP	5' LTR (n = 7)	100	85.7
	3' LTR (n = 12)	91.6	54.5

**Genome-wide analysis**	**LTR closest to TSS**	**% LTR associated with CGI promoters**	***P*-value of equality of proportion test**

IAP (n = 56)	5' LTR (n = 25)	80	0.03143
	3' LTR (n = 31)	48	

CGI: CpG Island; ETn/MusD: Early transposon/*Mus musculus *type D; IAP: Intracisternal (A) Particle; LTR: long terminal repeat; n: number of copies studied; TSS: transcription start site.

### Lack of spreading of DNA methylation into gene promoters

**Figure 4:**
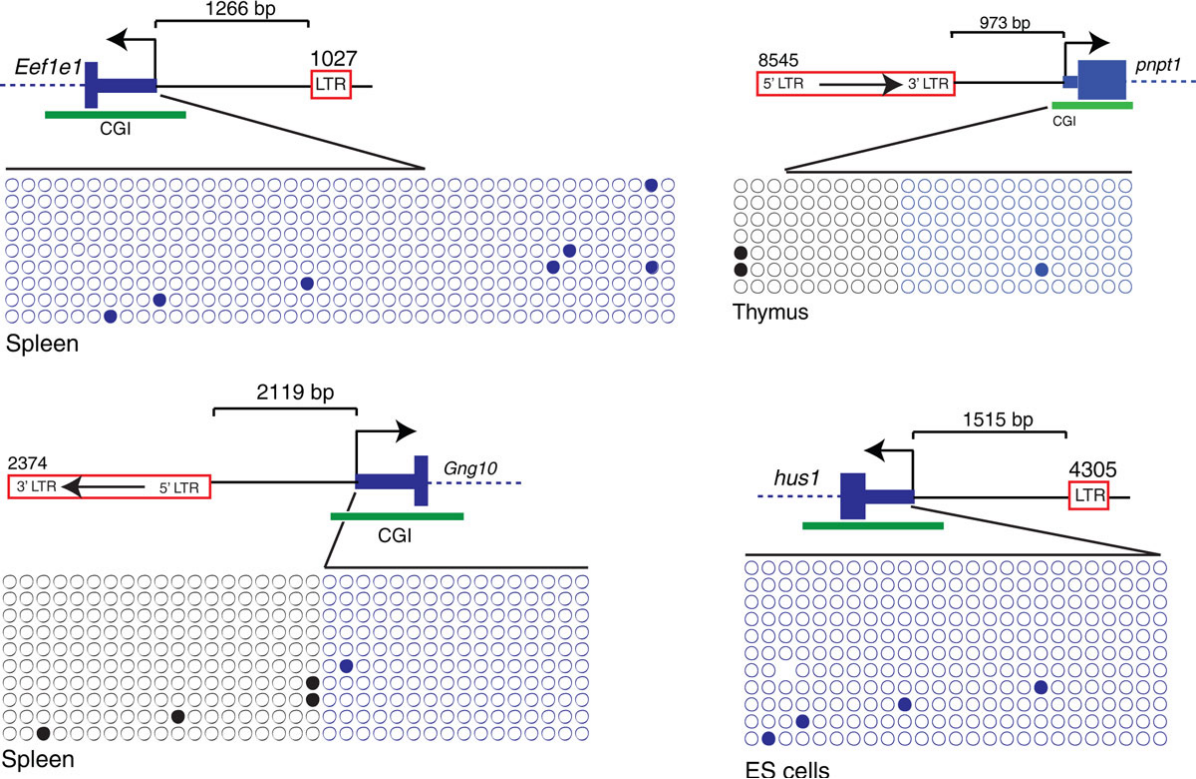
**Lack of DNA methylation spreading from methylated endogenous retrovirus copies into gene transcription start sites**. The cartoons show four examples of ERVs relative to the genes studied and further information can be found in Additional file 2. Only CpGs present in the gene promoter or close flanking region are shown, for the methylated ERV copies please refer to Figure S2 in Additional file 3. bp: base pairs; CGI: CpG Island; ERV: endogenous retrovirus; ES: embryonic stem; LTR: long terminal repeat.

Excluding the five examples of hypomethylated ERVs associated with a TSS of an adjacent gene, a total of 29 ERV copies were found to be methylated close to gene TSSs. We analyzed these ERV copies to better understand if DNA methylation can spread toward the promoters of the nearby genes. We randomly chose nine copies to analyze the DNA methylation of the associated gene promoter (Table 2). Apart from *B3galtl*, the case we previously described in ES cells [5], no spreading of DNA methylation from an ERV copy into CGI promoters was observed (Figure 4 and Table 2). For instance, one ETn/MusD and one IAP copy, located between 700 bp and 1 kb from CGI promoters (*Mthfd2l *in ES cells, embryo and brain and *Pnpt1 *in ES cells and thymus) have 80% to 95% of their CpG sites methylated, while the CGI promoters remain virtually unmethylated (0% and 1% respectively, Figure S2 in Additional file 3). Most non-CGI promoters did not contain enough CpGs to assay for DNA methylation robustly (three ETn/MusD and seven IAP cases) and the only case we have studied (*Gdpd3 *in ES cells and brain) does not show any spreading of DNA methylation (Table 2).

**Table 2:** Lack of spreading of DNA methylation from ERV copies into gene transcription start sites

ERV ID	Family	Type	Gene	Distance to TSS (bp)	% ERV methylation	% Gene promoter methylation	CGI promoter?	Tissue studied
1027	ETn/MusD	Solo	*Eef1e1*	1266	MeDIP (see Table S1)	1.4	Yes	Spleen
2374	IAP	Full length	*Gng10*	2119	92	1	Yes	Spleen
3893	ETn/MusD	Full length	*Mthfd2l*	754	80*	0*	Yes	ES cells, embryo, brain
ti1080339794	IAP	Full length	*Gdpd3*	437	75.7	0	No	ES cells
4305	IAP	Solo	*Hus1*	1515	86	1	Yes	ES cells
8545	IAP	Full length	*Pnpt1*	973	95^a^	1*	Yes	ES cells, thymus
9963	IAP	Full length	*Atxn1l*	1316	97	0	Yes	Brain, liver
ti1072970530	IAP	Solo	*B3galtl*	368	84.1	1.4	Yes	Brain
ti1102177987	IAP	Full length	*Eps15*	1613	97.2	1	Yes	Brain

*Average of tissues studied. bp: base pairs; CGI: CpG Island; ERV: endogenous retrovirus; ES: embryonic stem; ETn/MusD: Early transposon/*Mus musculus *type D; IAP: Intracisternal (A) Particle; TSS: transcription start site.

To assess potentially more subtle effects of ERV impact on the DNA methylation levels of a nearby gene promoter, we exploited F1 hybrids that possess one allele with an insertionally polymorphic ERV copy and an empty allele (Figure S2 in Additional file 3, pages 26, 29 and 37). Despite the presence of a nearby methylated ERV copy, no differences in DNA methylation of the gene promoter were observed between the alleles for all three examples studied. Not surprisingly, most of the genes analyzed contained a CGI promoter, and those are known to be preserved in an unmethylated state throughout development. Nevertheless, we previously observed spreading of DNA methylation into a CGI gene, *B3galtl *[5], indicating that CGIs can occasionally be invaded by DNA methylation spreading from an ERV copy. Curiously, *B3galtl *is associated with a methylated ERV in all tissues studied (ES cells, brain and kidney), but spreading of DNA methylation is only observed in ES cells. In somatic tissues (brain and kidney), spreading seems to be blocked at the CGI promoter (Figure S2 in Additional file 3, page 37). In ES cells, IAPs are associated with H3K9me3 [5] and may promote spreading of both repressive histone marks and DNA methylation, but H3K9me3 is mostly absent in differentiated cells [23]. We observed no spreading of DNA methylation in our study, suggesting DNA methylation by itself is not sufficient to spread into gene promoters. In summary, spreading of DNA methylation from ERV copies close to gene promoters is a rare event and may be tissue specific.

### H3K4me3 and CTCF may protect gene promoters from spreading of DNA methylation

**Figure 5:**
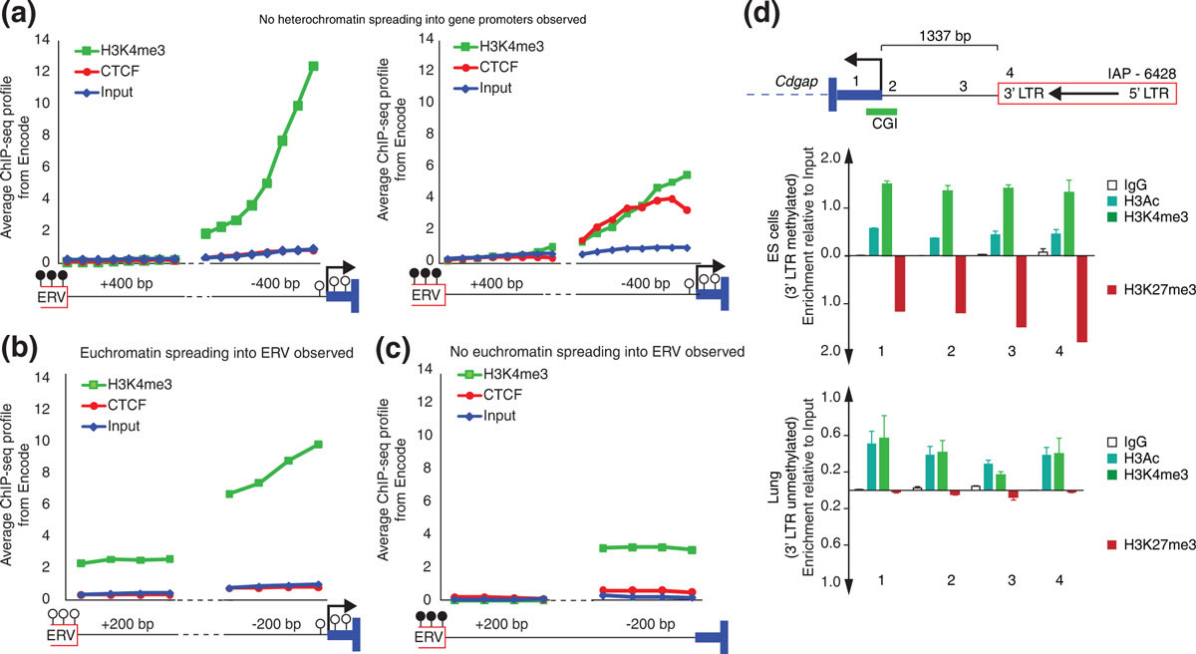
**Chromatin environment of intervening regions of methylated copies**. **(A) **The average H3K4me3 and CTCF profiles of the intervening regions between ERV and gene are shown. Gene regions represented on the left part of panel A show only H3K4me3 enrichment while regions represented on the right harbor both H3K4me3 and CTCF. The genes included in the data set are *Eef1e1*, *Gng10*, *Hus1 *and *Pnpt1 *for the left panel and *Mthfd2l *and *Atxn1l *for the right panel. The four genes not included in this analysis but in Table 2 are either absent from Encode strains or not studied in tissues available at Encode. **(B) **Average profiles of intervening regions adjacent to unmethylated copies (associated genes transcribed). The regions included in this analysis are from the following genes: *Lair*, *Cyb5r1*, *Bola1*, *Cdgap *and *Cml2 *which constitute all the unmethylated copies available. **(C) **Average profile of intervening regions harboring a tissue-specific methylated epiallele. The regions included in this analysis harbor the following genes : *Lair*, *Cdgap *and *Cml2*, which constitutes the only cases where the associated ERV copy is found methylated in one tissue (associated genes silent) and unmethylated in another (associated genes transcribed). The flanking regions chosen for this analysis (400 bp and 200 bp) correspond to a minimum length common for all regions analyzed (with the exception of *Cml2 *which is 68 bp from its ERV copy). **(D) ***Cdgap *chromatin immunoprecipitation-qPCR in ES cells (top) and lung (bottom). We assayed for permissive marks (H3K4me3, H3 acetylation) and repressive marks (H3K27me3) along with a mock control (IgG) in the intervening region between *Cdgap *and the ERV copy. Numbers in the cartoon show the localization of the quantitative PCR primer pairs. H3K27me3 enrichment is shown in opposite direction of H3K4me3. bp: base pairs; CGI: CpG Island; CTCF: CCCTC-binding factor; ERV: endogenous retrovirus; ES: embryonic stem; IgG: immunoglobulin G; LTR: long terminal repeat.

Given that the methylation state of an ERV has no apparent impact on the methylation level of a nearby gene promoter, we decided to explore this phenomenon further. Specifically, we wondered if intervening regions, that is, the sequences between the ERVs and the genes, could act as boundary elements, protecting the gene promoter from spreading of detrimental ERV DNA methylation. H3K4me3 is a known DNA methylation antagonist [24] and it has been suggested that its presence blocks the deposition of methyl groups on cytosines [25]. Furthermore, insulators, such as CTCF, can isolate genes from their regulatory elements as enhancer-blocking elements (reviewed in [26]). Recent reports have also suggested that CTCF is able to block putative heterochromatin spreading and establish a barrier element [27-29]. The barrier-insulator role of CTCF is described as cell-specific and depends on cofactors to block heterochromatin spreading [29]. We exploited available Encode data [30] from Ren's group at the Ludwig Institute for Cancer Research to compute an average profile of H3K4me3 and CTCF enrichment in the intervening regions between six methylated ERV copies and genes that were tested for spreading of DNA methylation in Table 2 (see Figure 5A for average profiles and Figure S2 in Additional file 3 for individual profiles). As expected, active genes bear H3K4me3 in their flanking regions (Figure 5A left panel) however no enrichment is observed in the vicinity of the methylated ERV copies. CTCF along with H3K4me3 is also associated with some of the genes studied (Figure 5A right panel).

The average profile of all genes associated with a methylated ERV copy (not only genes studied in our spreading analysis) show a similar pattern with either H3K4me3 only or with both CTCF and H3K4me3 (Figure S4 in Additional file 3). Curiously, five full-length ERV copies harbor their 5' LTR closest to the gene TSS, and four of them present CTCF binding in their intervening region, while all 3' LTRs, with the exception of one, lack CTCF binding. We hypothesize that if 5' LTRs have a higher selective pressure to be methylated, compared with the 3' LTR, then the presence of a CGI and H3K4me3 may not be sufficient to protect gene promoters from silencing, requiring the binding of CTCF to reinforce the chromatin barrier. Interestingly, the five ERV copies found to be unmethylated near active gene promoters harbor H3K4me3 within their flanking sequences (Figure 5B and Figure S2 in Additional file 3 for individual profiles), suggesting spreading of host gene euchromatin towards ERV copies. Thus, the state of methylation of some ERV copies in the mouse genome appears to be influenced by the spreading of permissive chromatin from nearby gene promoters. The presence of H3K4me3 seems therefore necessary for the integrity of the nearby active gene promoters.

### Impact of gene expression on ERV DNA methylation

Promoters characterized by H3K4me3 and RNA Polymerase II (POL2) are known to be associated with active genes and, as expected, all the genes studied in this analysis harbor an open chromatin enriched in POL2 (Figure S2 in Additional file 3). We hypothesize that the presence of such active marks at the gene promoter generates an open chromatin state at the ERV copy that in turn is unmethylated. In such cases, when the gene is silent, the lack of active marks at the gene promoter would no longer generate spreading of euchromatin and the nearby ERV copy would remain methylated. We decided to analyze the copies described as unmethylated in our study but searched for tissues where the nearby gene is silent and therefore lacks POL2 and also H3K4me3. For three of these cases, the tissue specificity of gene expression correlated with the methylation state of the nearby ERV, in that tissues where the genes are silent exhibit hypermethylation of the ERV sequence (Figure S2 in Additional file 3). Unfortunately, the other two genes are housekeeping genes and so tissues where such genes are silent are not available. Therefore, in all cases available for study, the transcriptional state of the gene appears to impact the methylation state of the nearby ERV.

In tissues where these ERV copies become methylated, we observed a lack of H3K4me3 overlying the ERV flanking sequence even though gene promoters retain an open chromatin structure (Figure 5C). We wondered if repressive chromatin marks would be present in methylated ERV copies whereas H3K4me3 would be associated with unmethylated copies. We analyzed the *Cdgap *promoter as a surrogate for this scenario, because it features a nearby IAP copy methylated in ES cells where the gene is silent, but unmethylated in somatic tissues where the gene is expressed (thymus, brain and lung). We assayed for euchromatic marks (H3 acetylation and H3K4me3) and a repressive mark (H3K27me3, Figure 5D). In ES cells, the *Cdgap *promoter is bivalent, characterized by enrichment for both H3K4me3 and H3K27me3, and this chromatin signature extends to the 3' LTR of the ERV copy. In the relevant F1 hybrid ES cells, the bivalent marks are observed for both empty and full alleles, suggesting no influence of the nearby IAP copy on H3K27me3 enrichment (Figure S5 in Additional file 3). Genes associated with bivalent promoters are often poised to be expressed later in development [23]. In somatic cells, however, the *Cdgap *promoter lacks H3K27me3 and maintains enrichment for the open chromatin mark H3K4me3, which again extends to the nearby IAP copy (Figure 5D), confirming our Encode analysis (Figure 5C). Therefore, together with our Encode analysis, we have shown that permissive chromatin marks in somatic tissues can spread from active gene promoters into ERV copies, most likely blocking the ERV from being methylated; in ES cells or other tissues, the presence of a bivalent domain and a CGI may allow the nearby ERV copy to be methylated and yet block DNA methylation spreading into the gene promoter.

### Impact of nearby ERVs on gene expression

**Figure 6:**
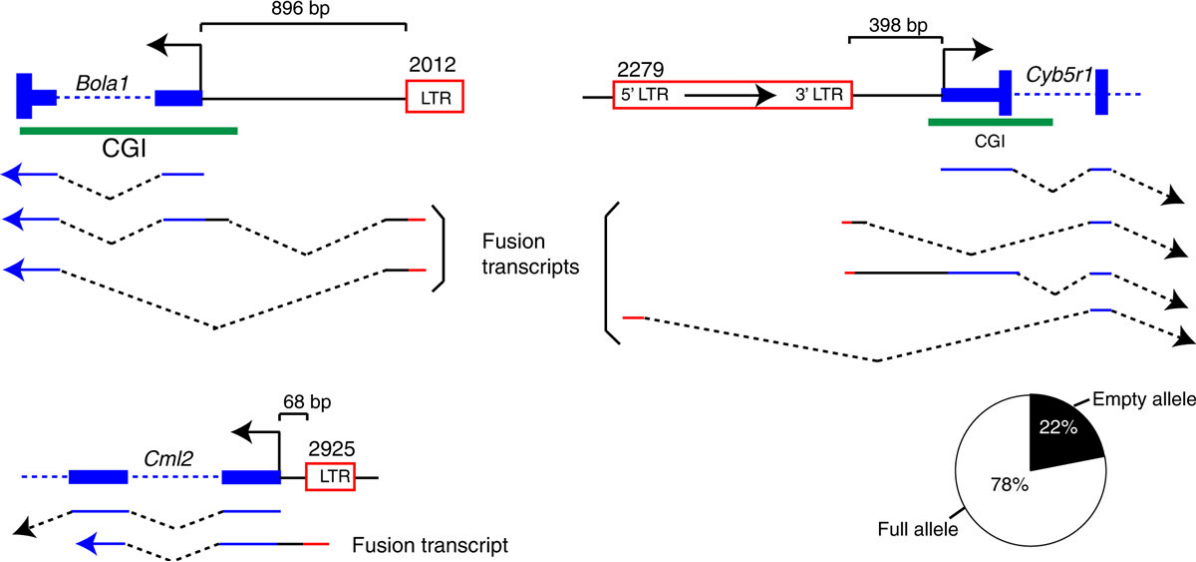
**Unmethylated endogenous retrovirus copies may act as promoters for host genes**. RT-PCR was performed on cDNA samples from tissues where the ERV studied is unmethylated, using primers targeting the ERV and either the first or the second gene exon (see Materials and methods). cDNAs are represented by flat lines (sequenced cDNA) and black dashed lines (inferred introns). Genes are in blue, intergenic regions in black and ERV sequences in red. Allelic expression of *Cyb5r1 *in hybrids is shown as a pie-chart. Hybrids containing one allele with the ERV copy (white) and one allele without it (black), were subjected to allelic expression quantification via single nucleotide polymorphisms (see Materials and methods). bp: base pairs; CGI: CpG Island; ERV: endogenous retrovirus; LTR: long terminal repeat; PCR: polymerase chain reaction; RT: reverse transcriptase.

ERVs are known to occasionally act as promoters for nearby genes [1,31]; we questioned if the five unmethylated ERVs could act as alternative promoters and produce chimeric transcripts. Indeed, such transcripts were found for three of the unmethylated copies analyzed (Figure 6). Given that insertionally polymorphic copies provide a perfect model to study ERV influence on genes, we again exploited F1 hybrid allele-specific expression, where one allele contains the ERV copy and the other does not. The ETn/MusD copy near *Cyb5r1 *is present in B6 but not in A/J and allelic expression analysis in mouse hybrid embryos revealed that the B6 allele, and therefore, putative ERV-gene fusions, accounts for the majority of the gene expression in the embryo (Figure 6). The potential functional impact of the ERV-induced gene transcripts identified here remains unknown.

## Conclusions

IAPs and ETn/MusDs are high copy number ERV families and, while hundreds to thousands of copies are present in the genome, relatively few are present near genes. Because DNA methylation in general targets TE copies, it is important for the host to manage the impact of epigenetic regulation of the copies that remain near genes. We show here, for the first time, that two ERV families, ETn/MusD and IAPs, are differently targeted by DNA methylation when near genes, with nearly all IAP copies remaining methylated throughout the genome but ETn/MusD copies being less methylated when near TSSs. Our dataset, although limited, contains every ETn/MusD copy close to genes and 30% of all IAP copies found near genes (78% of all IAP copies within 2 kb of a TSS). Therefore, our conclusions could reasonably apply to all copies of both types of ERVs in the genome.

We have previously shown that the repressive mark H3K9me3 spreads robustly from IAPs but less so from ETn/MusDs [5]. Further evidence that these two ERV families are distinctly epigenetically regulated comes from a recent study showing that knockdown of both Dnmt1 and SetDB1 (responsible for depositing H3K9me3 on these ERV families) is required in ES cells to achieve robust de-repression of IAP transcription, whereas only SetDB1 knockdown is necessary for activation of ETn/MusD [14]. These data could suggest that IAPs are more detrimental to host genes than ETn/MusDs, and are thus under more stringent control.

A recent study demonstrated that Alu SINE elements are hypomethylated in human when positioned near expressed genes, but are methylated when near silenced genes [32]. However, in marked contrast to ERVs, Alus are generally well-tolerated near genes and in fact show enrichment in gene-rich regions [33,34], suggesting epigenetic interactions between Alus and host genes are quite different than those between ERVs and genes. In rice, the retrotransposon *dasheng *presents tissue-specific DNA methylation correlating with nearby gene expression tissue specificity [35]. Furthermore, *dasheng *unmethylated copies impact host gene expression by producing antisense chimeric transcripts that putatively promote mRNA degradation [35]. Here, we found that mouse ERV elements impact the host gene by donating a promoter and producing fusion transcripts.

All 5' LTRs included in our analysis are methylated. Therefore we hypothesize that, since the regulatory sequences necessary for ERV transcription and possible transposition are present in the 5' LTR, methylation, and consequently silencing, of this LTR is necessary to reduce harmful effects of putative new transpositions. Furthermore, we have shown that, compared with CGI promoters, non-CGI promoters are relatively depleted of instances where the 5' LTR is proximal. This observation suggests that spreading of DNA methylation from 5' LTRs into non-CGI promoters might be the more likely scenario, thereby leading to harmful effects on gene expression and negative selection against such ERV copies. Indeed, the role of CpG methylation on the regulation of non-CGI genes remains unclear. Several reports have shown that expression of non-CGI genes is independent of DNA methylation [36] while a recent report reveals *in vitro *silencing of two CpG-poor genes caused by DNA methylation and nucleosome remodeling [37], confirming our previous observations [38,39]. CGI sequences are known to be resistant to methylation in humans and play an important role in maintaining an open chromatin environment via transcription factor binding and H3K4me3 enrichment ([40] and reviewed in [41]). The presence of H3K4me3 has previously been shown to exclude DNA methylation [24], suggesting CGI promoters may normally be protected from DNA methylation spreading from nearby ERVs. By contrast, CpG-poor genes are thought to harbor less ubiquitous H3K4me3 enrichment than CGI genes ([23] and reviewed in [42]) and hence may be more sensitive to ERV DNA methylation spreading. We show that H3K4me3 euchromatin is able to spread from gene promoters to nearby sequences, likely contributing to the lack of methylation at ERV copies in these regions. In agreement with our observations, Hejnar *et al*. have elegantly constructed a vector harboring a CGI from the mouse *Aprt *gene upstream of avian Rous sarcoma virus-derived sequences and transfected into non-permissive mammalian cells in order to follow methylation status and transcription levels of integrated copies [43]. While the Rous sarcoma virus is known to be methylated when inserted into mammalian cells, the adjacent CGI protects the inserted copies from DNA methylation and allows for virus transcription [43]. Hejnar's group has recently shown that proviruses inserted close to TSSs enriched in H3K4me3 are not immediately silenced compared with intergenic insertions and are resistant to DNA methylation [44], further supporting our hypothesis.

Boundary elements that act to separate euchromatin and heterochromatin domains may also act in blocking the accumulation and spreading of repressive marks, as has been shown for CTCF [26,27] or H2AZ [45]. A high proportion of 5' LTRs close to gene TSSs presented CTCF bound to their intervening regions, suggesting that 5' LTRs that remain after selection may require more than just H3K4me3 enrichment to block heterochromatin spreading. Interestingly, a recent genome-wide study in the human genome showed that gene promoters resistant to aberrant DNA methylation in cancer exhibited an increased frequency of retroelements nearby when compared with promoters prone to methylation. It was hypothesized that methylation-resistant genes may harbor more transcription factor-binding sites or boundary elements that act to prevent methylation, whereas methylation-prone genes do not have these protecting factors and are therefore more susceptible to potential silencing, which results in stronger negative selection against nearby insertions [46]. This hypothesis is in accordance with our data.

**Figure 7:**
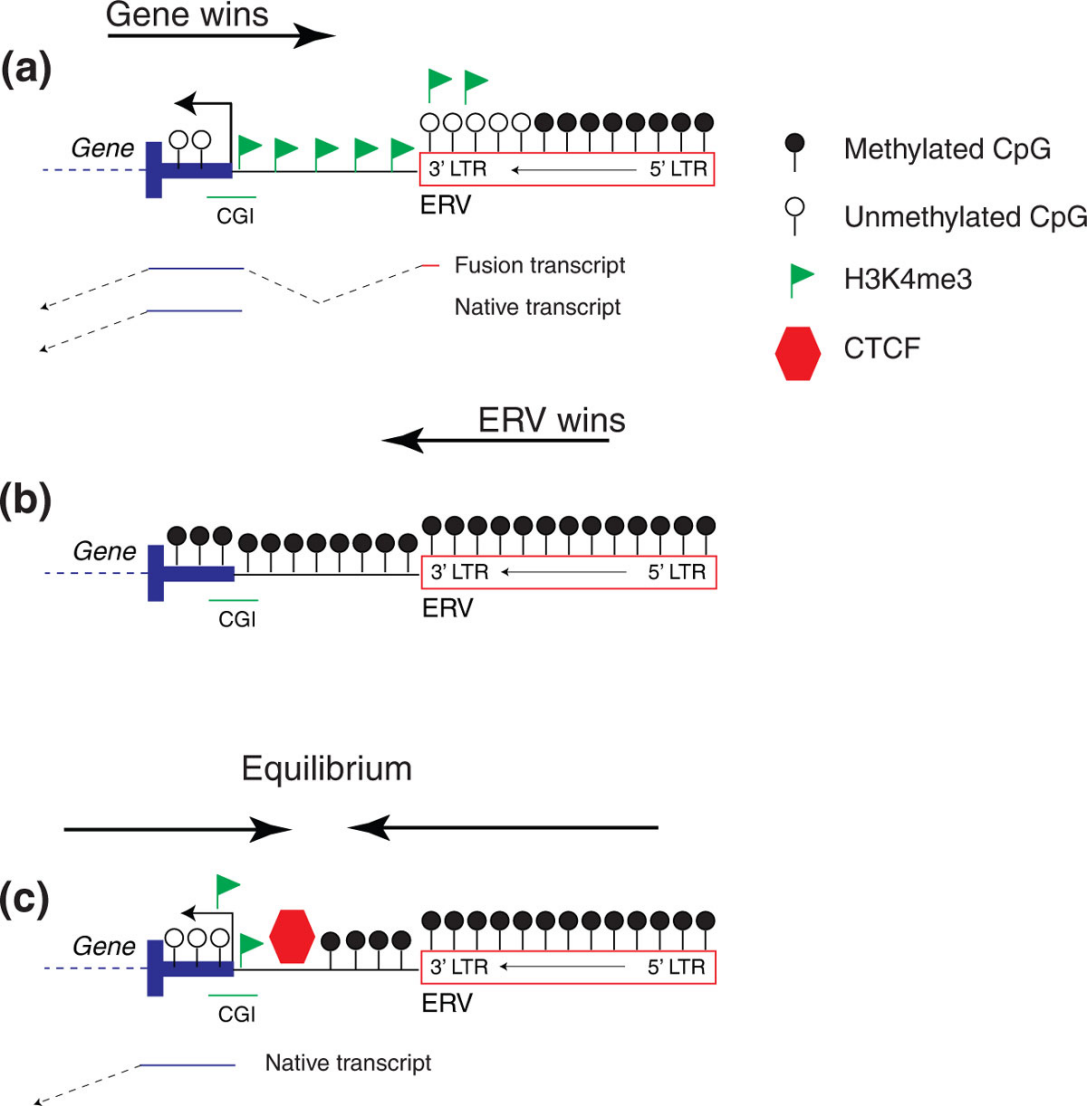
**Gene-endogenous retrovirus confrontation**. **(A) **Cartoon showing spreading of H3K4me3 euchromatin from the gene promoter towards the ERV sequence. The ERV becomes unmethylated and is able to act as an alternative promoter, potentially increasing expression of the gene. **(B) **ERV domination and heterochromatin spreading with consequent silencing of the nearby gene. **(C) **Equilibrium state where both euchromatin and heterochromatin form a boundary that may contain CTCF and allows for the ERV to be methylated while the gene is in an open chromatin conformation and is transcribed. CGI: CpG Island; CTCF: CCCTC-binding factor; ERV: endogenous retrovirus; LTR: long terminal repeat.

The complex relationship that exists between TEs and host genes suggests that selection may act not only on the potential harmful effects of TEs on host genes but also on the epigenetic consequences of the TE presence. The fight between ERV heterochromatin and host CGI promoter euchromatin favors the host gene (Figure 7A), with the gene-induced open chromatin sometimes impacting the nearby ERV and, in turn, increasing expression of the host gene through alternative promoters. Cases where the ERV-induced heterochromatin overcomes the promoter euchromatin (Figure 7B) are likely to be quite rare as most such insertions will be eliminated due to selection unless their effects do not significantly impact host fitness. While all the mechanisms underlying this chromatin battle remain unknown, it is important to note that every TE family may have a different relationship with host genes and most copies that have survived selection seem to have reached an epigenetic equilibrium with their associated host gene (Figure 7C).

## Materials and methods

### Choice of copies

ERV copies were retrieved from our previous analysis of four mouse genomes (A/J, DBA/2J, 129X1/SvJ and C57BL/6) [16]. Additional file 2 includes details of all copies studied, genome coordinates, strains where the copies are present (if they are fixed or insertionally polymorphic), tissues, methylation status and expression data. Figure S1 and S2 in Additional file 3 details all bisulfite and Encode data analysis. Additional file 1 contains all ETn/MusD and IAP copies extracted from our distribution analysis (Figure 1) close to gene TSSs. We have filtered all these copies with the following criteria: one EST should be available along with information on the expression of the gene and the ERV analyzed should be well annotated. We manually examined all 139 copies close to genes, and excluded cases where the gene is mis-annotated in RefSeq, if the gene contains too many TSSs, or if the ERV is inserted in an upstream gene (exonic or intronic). After filtering, we obtained seven ETn/MusD copies and 82 IAP copies close to genes. We studied all ETn/MusD copies but for practical reasons we studied only 30% of the IAP copies. To prioritize copies to study, we selected most IAP copies within 2 kb of a gene TSS (14 copies out of 18). The remaining 10 copies studied (a total of 24 IAP copies close to genes) were chosen randomly or based on their insertionally polymorphic state. We added three insertionally polymorphic IAP copies absent from the sequenced C57BL/6 genome but present in other strains because of their close proximity to the gene TSSs.

### Tissues and cells

C2 (C57BL/6) ES cell pellets were provided by the BC Cancer Research Center for Genetic Modeling and J1 (129S4/SvJae) and TT2 (C57BL/6xCBA) ES cell pellets by Dr I Maksakova. Tissues were dissected from C57BL/6, A/J, 129 and F1 hybrids (C57BL/6×129, C57BL/6×AJ). Hybrid ES cells studied are derived from C57BL/6×129 crosses.

### Endogenous retroviruses distribution and CpG island occurrence

Computational simulations of one million random ERV insertions in the mouse genome (mm9) were repeated three times and an average was calculated as the expected genomic ERV distribution. The actual distributions of ETns/MusDs and IAPs were calculated based on the RepeatMasker annotation downloaded from the University of California Santa Cruz (UCSC) Genome Browser [47]. To calculate the distance between an ERV and the nearest TSS or TTS, we used genomic coordinates of mouse RefSeq genes, which were also downloaded from the UCSC Genome Brower. A proportion equality test allowed us to compare between both distributions and appreciate significant differences. Lengths of CGI promoter regions were adapted from previous analysis [48]: 1.5 kb upstream and downstream of the gene TSS.

### MeDIP and quantitative PCR

All IAP and ETn/MusD copies chosen for this study are described in Additional file 2. ERV copies were all analyzed in C57BL/6 tissues and a panel of ETn/MusD copies was also studied in A/J tissues. ERVs far from genes were studied in tissues assayed for the study of copies close to genes, and ERVs near genes or inside genes were studied in tissues where the gene was expressed (based on the microarray expression data from GNF Expression Atlas [17,18]). No significant bias was observed among tissues for DNA methylation analysis. DNA was extracted from two to four mice, using AllPrep DNA/RNA mini kit from Qiagen (cat n°80204, Venlo, The Netherlands) following manufacturer's instruction. Total RNA was saved for qPCR analysis (see next section). DNA was treated with PureLink RNase A from Invitrogen (Carlsbad, CA, USA) and precipitated with a classic phenol chloroform protocol as described previously [49,50]. 4 µg to 6 µg of DNA was used for MeDIP [49,50]. An *in vitro *methylated DNA from *Drosophila melanogaster *was used as a positive control for the MeDIP. Two different fragments of approximately 150 bp were amplified from *Drosophila *genomic DNA containing several CpG sites. One of the fragments was *in vitro *methylated using a CpG methyltransferase (M.SSSI from New England Biolabs (Ipswich, MA, USA)) and methylation of CpGs was verified through digestion with restriction enzymes sensitive to CpG methylation (HPYCH4IV and HPAII (New England Biolabs), Figure S6 in Additional file 3). Both *Drosophila *fragments were added to all sonicated DNA prior to immunoprecipitation. Antibodies used for the MeDIP assay are anti-5-methylcytosine mouse mAb (162 33 D3) from Calbiochem (cat NA81, Amsterdam, the Netherlands) and IgG (Millipore Cs200580, Billerica, MA, USA). Quantification of DNA methylation was done by real-time PCR using Fast SYBR Green Master Mix from Applied Biosystems (Foster City, CA, USA). All primers presented unique dissociation curves and efficiencies ranged between 1.9 and 2.1 (all primers can be found in Additional file 2). Quantification of DNA methylation for a specific copy was obtained by using the formula: Efficiency of primers ^ (Ct Input - Ct IP) where Cts are cycle thresholds, and IP the immunoprecipitated sample, and normalizing by the *Drosophila *positive control. Values inferior to 0.2 were considered unmethylated and all were confirmed by bisulfite sequencing (Figure S1 in Additional file 3). All copies were confirmed by bisulfite sequencing, or by using different primers for qPCR in different biological replicates or by COBRA (Additional file 2 contains all DNA methylation data values; Figure S1 in Additional file 3 contains MeDIP data; Figure S2 in Additional file 3 contains bisulfite data).

### Bisulfite sequencing

Bisulfite conversion, PCR, cloning and sequencing were carried out as described previously [51]. All the sequences included in the analyses either displayed unique methylation patterns or unique C to T non-conversion errors (remaining Cs not belonging to a CpG dinucleotide) after bisulfite treatment of the genomic DNA. This avoids considering several PCR-amplified sequences resulting from the same template molecule (provided by a single cell). All sequences had a conversion rate greater than 95%. Sequences were analyzed with the Quma free online software (RIKEN, Kobe, Japan) [52]. Primers are available in Additional file 2 and all bisulfite sequences are in Additional file 4.

### COBRA

COBRA was performed as previously described [51]. Results are shown in Additional file 2. Enzymes used were TaqI, RsaI, HinfI, BstBI, AclI, XmnI and MboI.

### Average profiles of H3K4me3 and CTCF from Encode data

Cistrome was used to download and mine all Encode data [30,53]. Briefly, intervening regions for all unmethylated and methylated cases were computed. Through the Genome Browser table from Cistrome we downloaded signal values (wig bedgraph type) for H3K4me3, CTCF, POL2 and Input from all tissues available for all intervening regions. A profile for each intervening region is shown in Figure S2 in Additional file 3. To compute an average profile of H3K4me3, CTCF and Input we calculated the profile for each TE and gene ±400 bp or ±200 bp into the flanking region. The flanking length was chosen as a common minimum length to all intervening regions analyzed, as each case has a different TE to TSS distance (with the exception of *Cml2 *which is 68 bp away from the ERV copy). The average profile was calculated representing the TE at the left side and the TSS at the right side. All intervening regions that did not apply to this configuration were simply flipped. A link for the Encode data can be found at [54] and [55].

### Chromatin immunoprecipitation

Chromatin immunoprecipitation on tissues and ES cells were performed as previously described [5,56]. Briefly, homogenized tissues were cross-linked for 10 minutes and sonicated with a Bioruptor (bath sonicator). Homogenized cell pellets were treated with micrococcal nuclease until chromatin reached mononucleosome size. Chromatin isolated from approximately 30 µg of tissue or 1.5 million cells was used for each immunoprecipitation. An input fraction was separated and antibodies against IgG (Millipore 12370), H3K4me3 (Millipore 17614), H3K27me3 (Abcam 6002, Cambridge, MA, USA) and Histone 3 acetylation (Millipore 06599) were used (3 µg per sample). qPCR was used to estimate histone enrichment by using the formula: Efficiency of Primers ^ (Ct^input ^- Ct^IP^) with primer efficiency being determined by a standard curve with dilutions of input DNA (all primer efficiencies were equivalent and chosen between 1.9 and 2).

### RT-PCR and allelic expression

RT reactions were performed according to the Superscript III First-Strand Synthesis System protocol (Invitrogen). Modifications to the protocol include the following: the cDNA synthesis step was completed for 60 minutes at 50°C, and the reaction was terminated by heating samples at 70°C for 15 minutes. For each sample, two RT reactions were completed, one containing the RT and not the other (control for DNA methylation). cDNAs were diluted and used either for the detection of fusion transcripts or the estimate of allelic expression. For fusion transcripts, primers were designed within the first or second exon of the associated gene and within the nearby ERV copy. Primers are available in Additional file 2. PCR was carried out using Phusion High fidelity DNA polymerase (Finnzymes, Espoo, Finland) with conditions described by the manufacturer. Sequences of the fusion ERV-gene transcripts shown in Figure 6 have been deposited in GenBank with the following accession numbers: [GenBank:JX420285] to [GenBank:JX420290]. Quantification of allelic expression was done as described previously [5]. Primers used for allelic quantification targeted only the exons of the host gene and are available in Additional file 2.

## Abbreviations

bp: base pairs; CGI: CpG Island; CTCF: CCCTC-binding factor; ERV: endogenous retrovirus; ES: embryonic stem; EST: expressed sequence tag; ETn/MusD: Early transposon/*Mus musculus *type D; IAP: Intracisternal (A) Particle; IgG: immunoglobulin G; kb: kilobase pairs; LINE: long interspersed nuclear element; LTR: long terminal repeat; MeDIP: methylated DNA immunoprecipitation; PCR: polymerase chain reaction; qPCR: quantitative polymerase chain reaction; RT: reverse transcriptase; SINE: short interspersed nuclear element; TE: transposable elements; TSS: transcription start site; TTS: transcription termination site.

## Competing interests

The authors declare that they have no competing interests.

## Authors' contributions

RR and KMR carried out most of the molecular genetic studies and drafted the manuscript. YZ carried out the bioinformatics analysis and RR the ENCODE analysis. LG and SF participated in the molecular genetic studies. DLM conceived of the study, and participated in its design and coordination and helped to draft the manuscript. All authors read and approved the final manuscript for publication.

## Supplementary Material

Additional file 1 **ERV dataset before filtering**. ERV copies extracted from the distribution analysis (Figure 1) close to gene TSSs. Click here for file

Additional file 2 **ERV dataset assayed for methylation**. All ERV copies selected for DNA methylation analysis. Click here for file

Additional file 3 **Figures S1 to Figure S6**. An index is present as the first page that will guide readers through the different figures. Click here for file

Additional file 4 **All bisulfite sequencing data**. Compilation of all bisulfite sequences. Click here for file
