# Supplementary material for: Epigenetic interplay between mouse endogenous retroviruses and host genes
Source: Genome Biol. 2012 Oct 3;13(10):R89. doi: 10.1186/gb-2012-13-10-r89 (PMC3491417; doi:10.1186/gb-2012-13-10-r89)
Supplement: Additional file 3 — Figures S1 to Figure S6. An index is present as the first page that will guide readers through the different figures. [file gb-2012-13-10-r89-S3.pdf]

Index

Figure S1	page 2
Figure S2	page 3
Figure S2A (ETn/MusD)	page 3 to page 18
852	page 3
1027 (*Eef1e1*)	page 4
1028	page 5
1572 (*Lair*)	page 6
1593	page 7
1814	page 8
1924	page 9
1979 *(4930558C23Rik)*	page 10
2012 (B*ola1*)	page 11
2279 (*Cyb5r1*)	page 12
2925 (*Cml2*)	page 13
3171	page 14
3173	page 15
3523	page 16
3893 (Mthfd2l)	page 17
3989	page 18
Figure S2B (IAP)	page 19 to page 39
592 (*1190003K10Rik*)	page 19
1267 (*Pol2rm*)	page 20
2374 (*Gng10*)	page 21
2666 (*Ext2*)	page 22
3204 (*Ttll4*)	page 23
3581	page 24
3806	page 25
4305 (*Hus1*)	page 26
4928 (*Parva*)	page 27
5240 (*Ogfod2*)	page 28
6428 (*Cdgap*)	page 29
8253 (*Catsper 3*)	page 30
8532	page 31
8545 (*Pnpt1*)	page 32
8561 (*Rad50*)	page 33
9667 (*3110003A17Rik*	page 34
9963 (*Atxn1l*)	page 35
10767 (*Dnahc6)*	page 36
1072970530 (*B3galtl*)	page 37
ti1080339794 (*Gdpd3*)	page 38
ti1102177987 (*Eps15)*	page 39
Figure S3	page 40
Figure S4	page 41
Figure S5	page 42
Figure S6	page 43

Figure S1

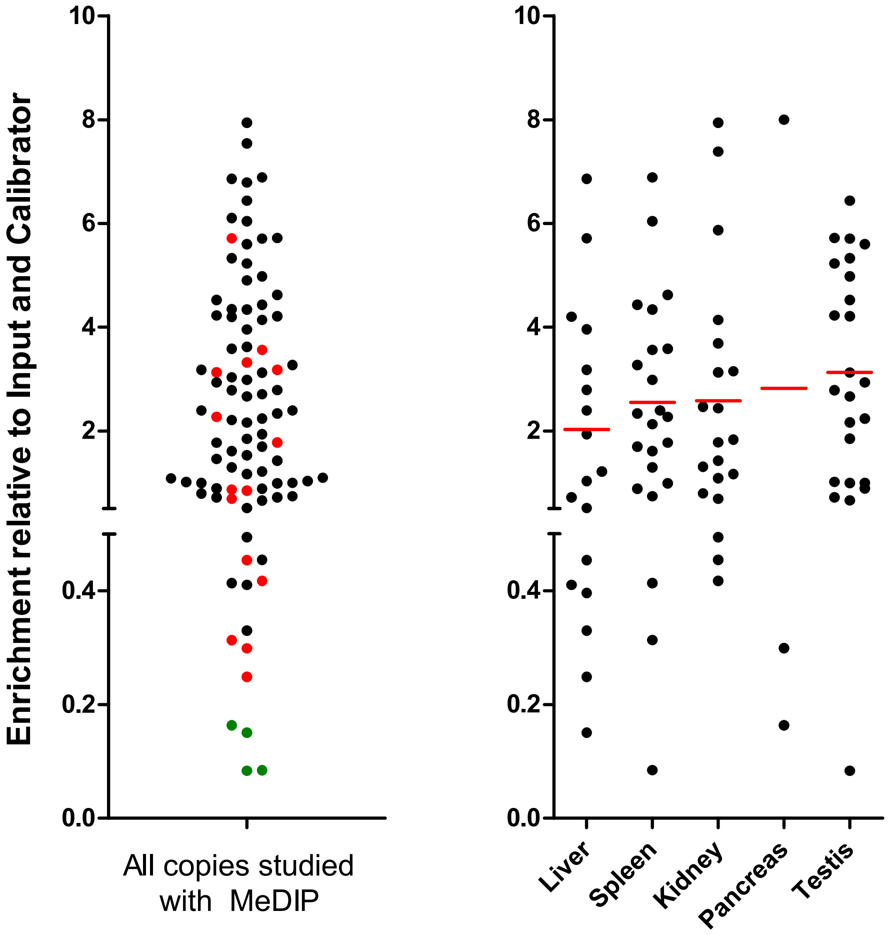

Figure S1. MeDIP-qPCR enrichment for all ERV copies analyzed. Each dot represents a biological replicate (see Materials and Methods). Colored dots (red and green) are ERV copies where the MeDIP-qPCR enrichment was confirmed by bisulfite sequencing (green are unmethylated copies and red methylated ones). Right panel shows MeDIP -qPCR results by tissue studied. No significant difference was observed between tissues. The mean for each tissue is shown as a red horizontal bar.

Figure S2. Bisulfite sequencing data of ERV copies near genes. A. ETn/MusD copies. B. IAP copies. Cartoons show the relative distance and position of the ERV copy studied and the nearby gene when present. ERV IDs are shown along with the state of the copy relative to gene TSSs (close or far). Genes are in blue, ERVs in red and CpG islands in green. Arrows represent the sense of transcription. Empty circles are unmethylated CpGs while filled circles are methylated ones. When necessary global methylation profile was compared between ERV sequences with a Mann-Whitney U-test giving p values : *** < 0.001, ** < 0.01 and * < 0.05. Encode average profiles are shown for copies present in the strain and tissues used by Encode (see Methods).

Figure S2A

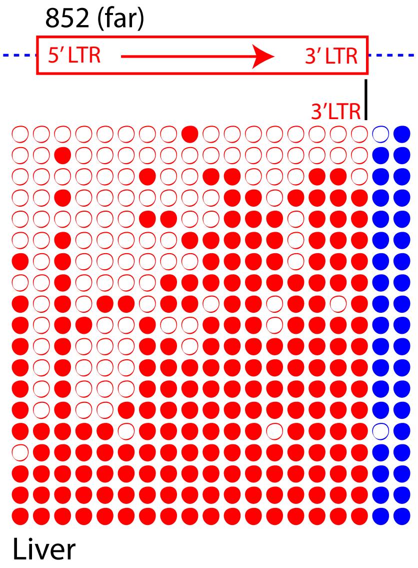

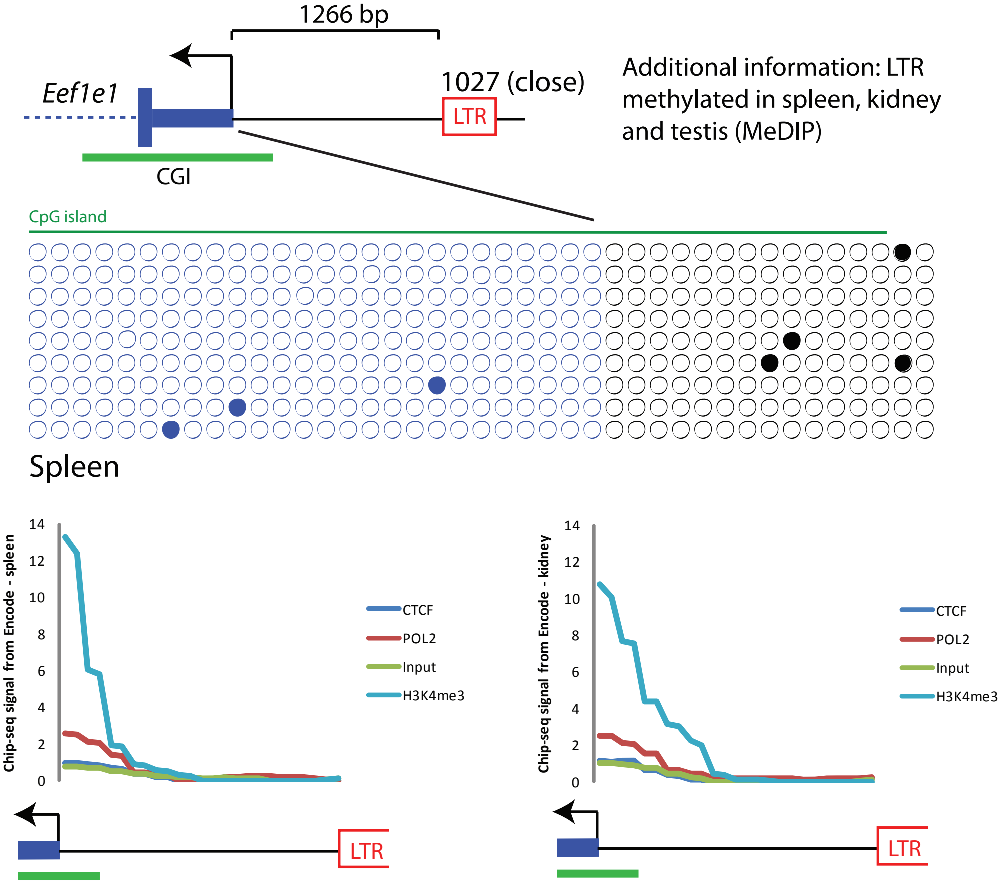
1266 bp
Eef1e1
1027 (close)
LTR
CGI
Additional information: LTR methylated in spleen, kidney and testis (MeDIP)
CpG island
Spleen
Chip-seq signal from Encode - spleen
14
12
10
8
6
4
2
0
CTCF
POL2
Input
H3K4me3
LTR
Chip-seq signal from Encode - kidney
14
12
10
8
6
4
2
0
CTCF
POL2
Input
H3K4me3
LTR

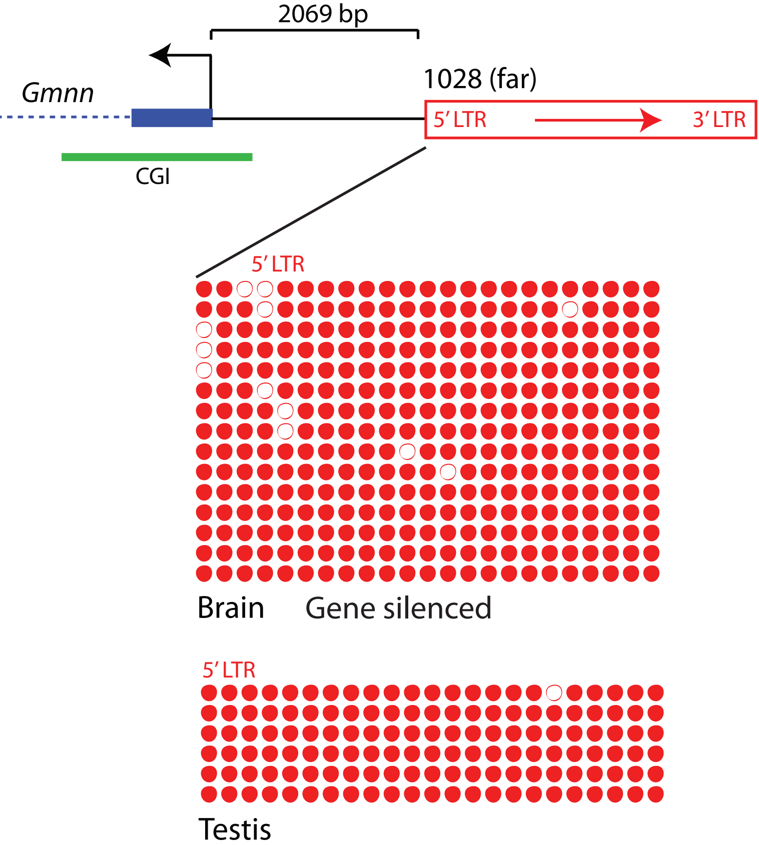
2069 bp
Gmnn
1028 (far)
5′ LTR
3′ LTR
CGI
5′ LTR
Brain
Gene silenced
5′ LTR
Testis

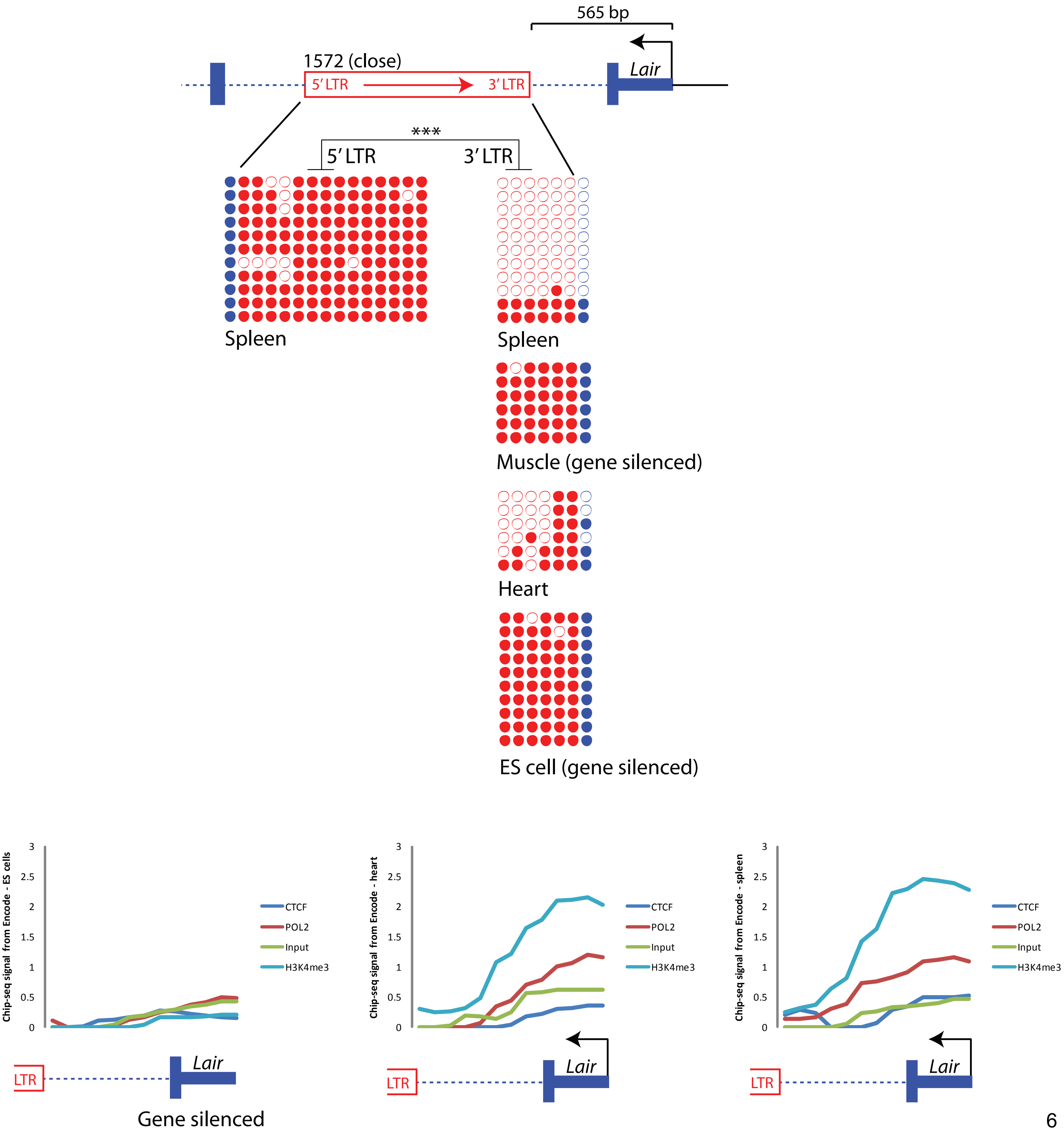
565 bp
1572 (close)
5′ LTR
3′ LTR
Lair
***
5′ LTR
3′ LTR
Spleen
Spleen
Muscle (gene silenced)
Heart
ES cell (gene silenced)
Chip-seq signal from Encode - ES cells
Chip-seq signal from Encode - heart
Chip-seq signal from Encode - spleen
3
2.5
2
1.5
1
0.5
0
CTCF
POL2
Input
H3K4me3
LTR
Lair
Gene silenced

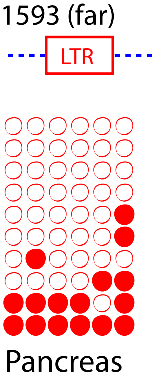
1593 (far)
LTR
Pancreas

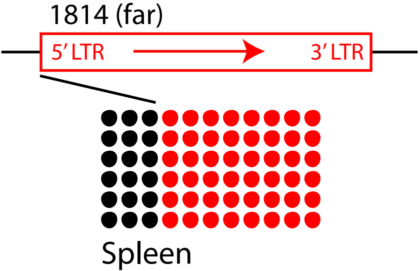
1814 (far)
5' LTR
3' LTR
Spleen

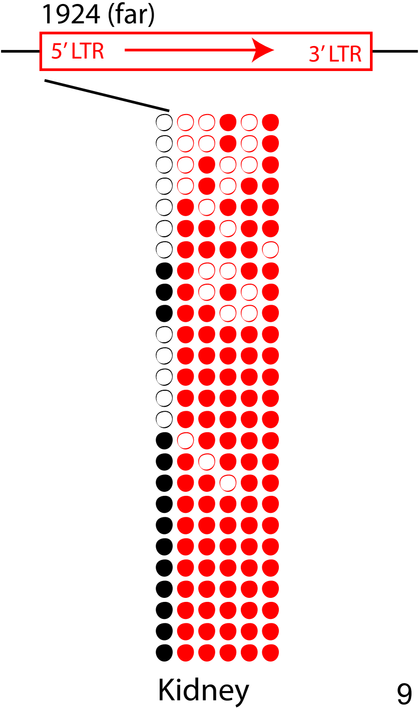
1924 (far)
5′ LTR
3′ LTR
Kidney

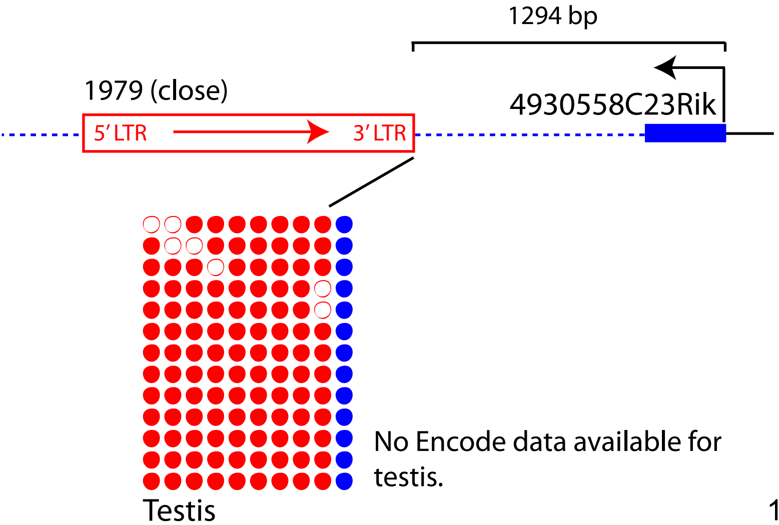

No Encode data available for testis.

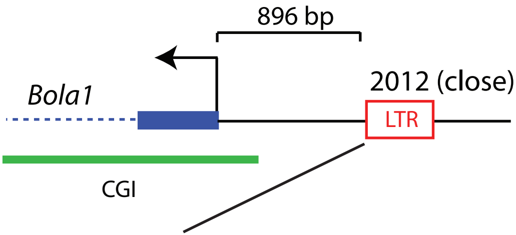

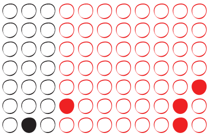

Testis

Additional information : LTR unmethylated in kidney and spleen (MeDIP)

No Encode data available for testis.

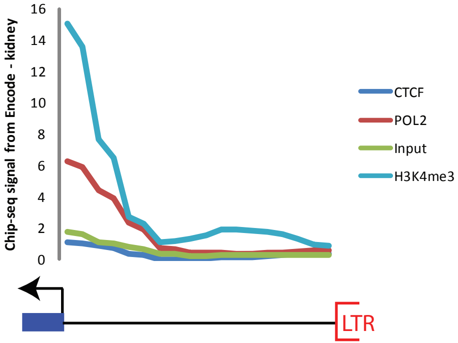

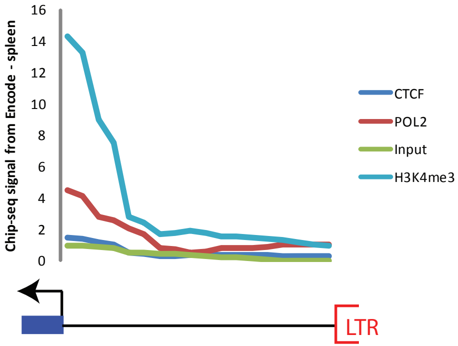

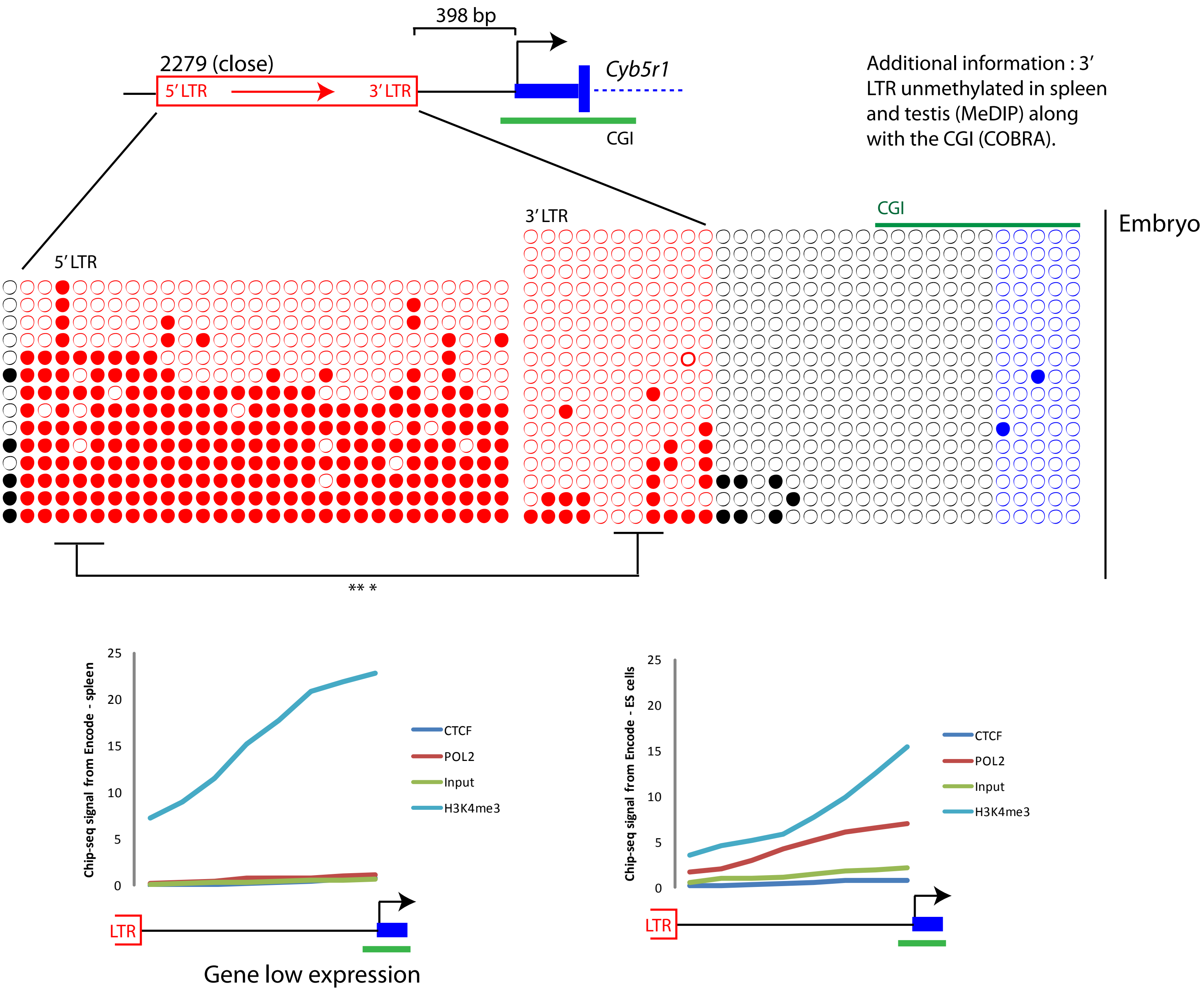
398 bp
2279 (close)
5′ LTR
3′ LTR
Cyb5r1
CGI
Additional information : 3′ LTR unmethylated in spleen and testis (MeDIP) along with the CGI (COBRA).
3′ LTR
CGI
Embryo
5′ LTR
** *
Chip-seq signal from Encode - spleen
25
20
15
10
5
0
CTCF
POL2
Input
H3K4me3
LTR
Gene low expression
Chip-seq signal from Encode - ES cells
25
20
15
10
5
0
CTCF
POL2
Input
H3K4me3
LTR

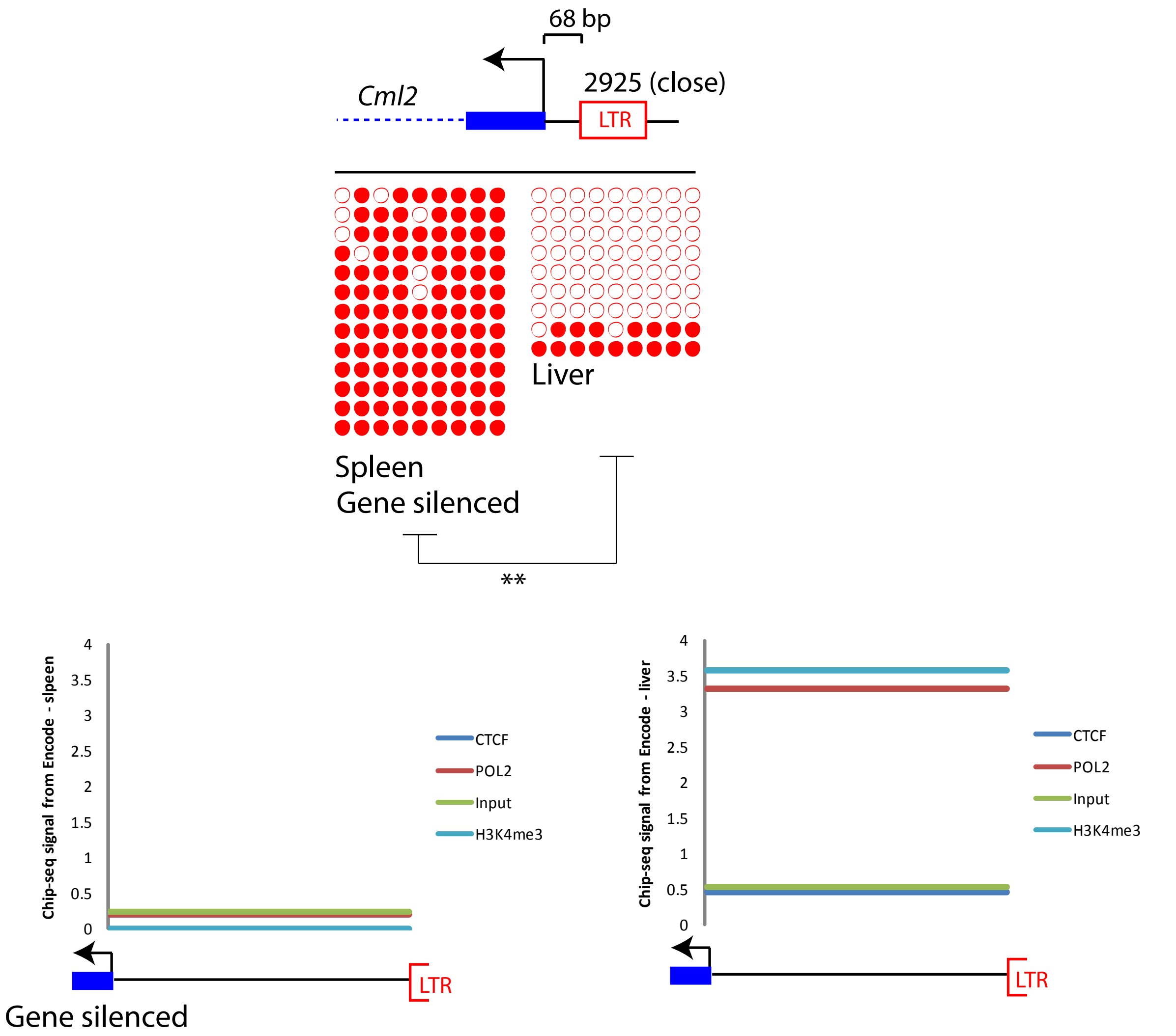
68 bp
Cml2
2925 (close)
LTR
Liver
Spleen
Gene silenced
**
Chip-seq signal from Encode - slpeen
4
3.5
3
2.5
2
1.5
1
0.5
0
CTCF
POL2
Input
H3K4me3
LTR
Gene silenced
Chip-seq signal from Encode - liver
4
3.5
3
2.5
2
1.5
1
0.5
0
CTCF
POL2
Input
H3K4me3
LTR

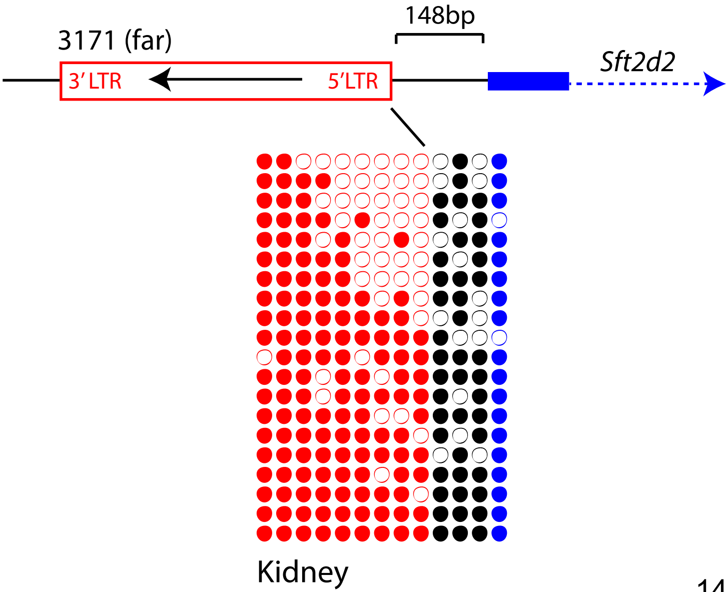
148bp
3171 (far)
3'LTR
5'LTR
Sft2d2
Kidney

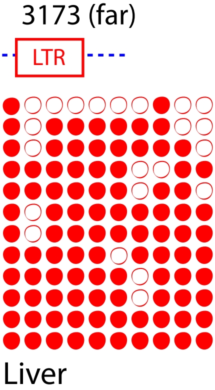
3173 (far)
LTR
Liver

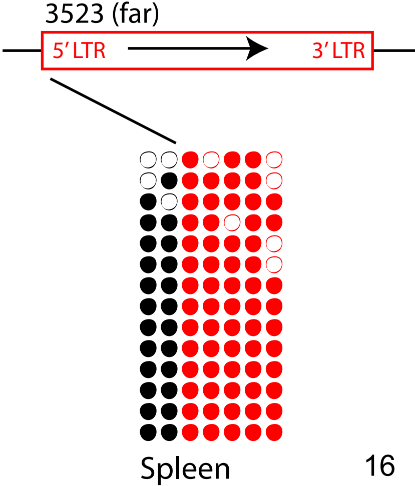
3523 (far)
5' LTR
3' LTR
Spleen

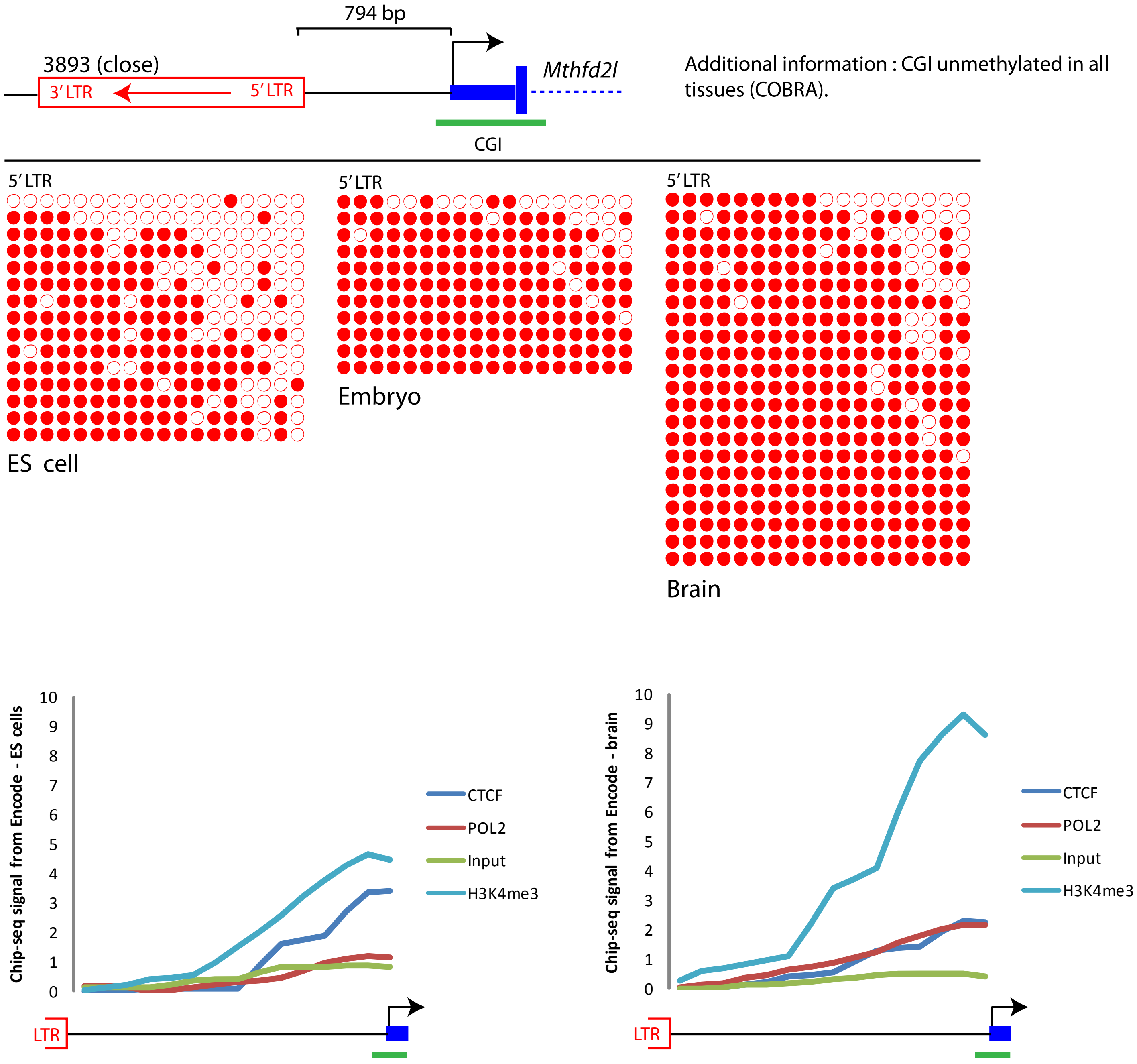
794 bp
3893 (close)
3' LTR
5' LTR
Mthfd2l
CGI
Additional information : CGI unmethylated in all tissues (COBRA).
5' LTR
ES cell
5' LTR
Embryo
5' LTR
Brain
Chip-seq signal from Encode - ES cells
CTCF
POL2
Input
H3K4me3
LTR
Chip-seq signal from Encode - brain
CTCF
POL2
Input
H3K4me3
LTR

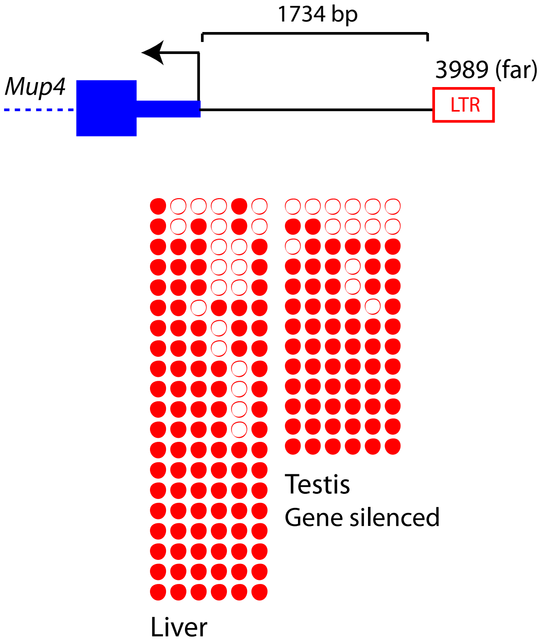
1734 bp
3989 (far)
Mup4
LTR
Testis
Gene silenced
Liver

Figure S2 B

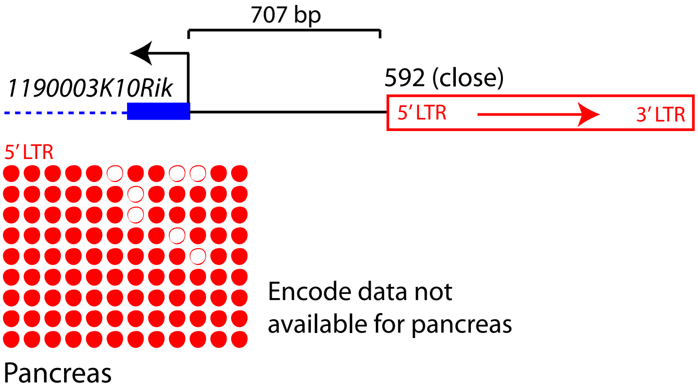
707 bp
1190003K10Rik
592 (close)
5' LTR
3' LTR
5' LTR
Pancreas
Encode data not available for pancreas

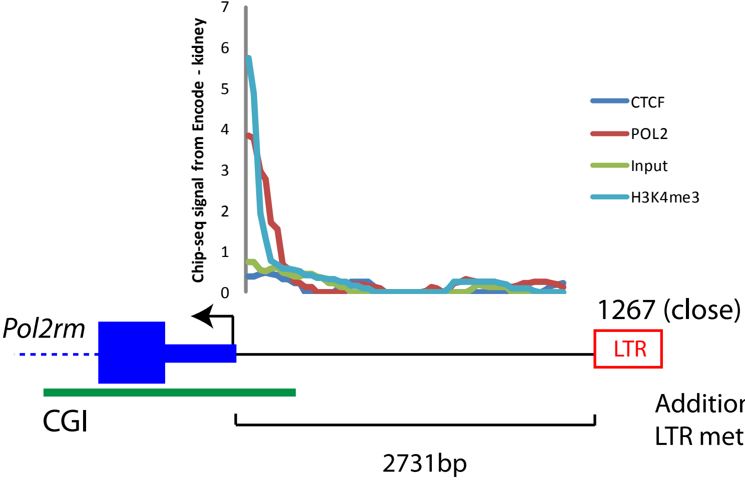

Additional information :
LTR methylated (MeDIP)

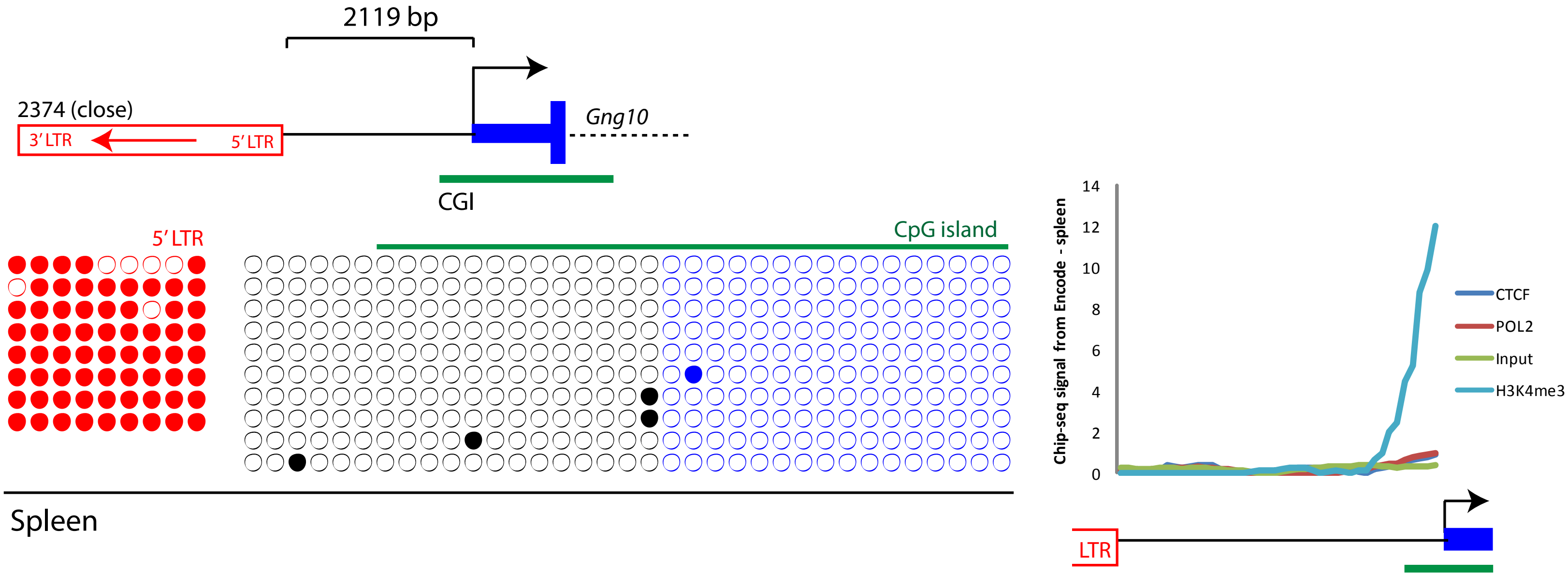
2119 bp
2374 (close)
3′ LTR
5′ LTR
Gng10
CGI
5′ LTR
CpG island
Spleen
Chip-seq signal from Encode - spleen
14
12
10
8
6
4
2
0
CTCF
POL2
Input
H3K4me3
LTR

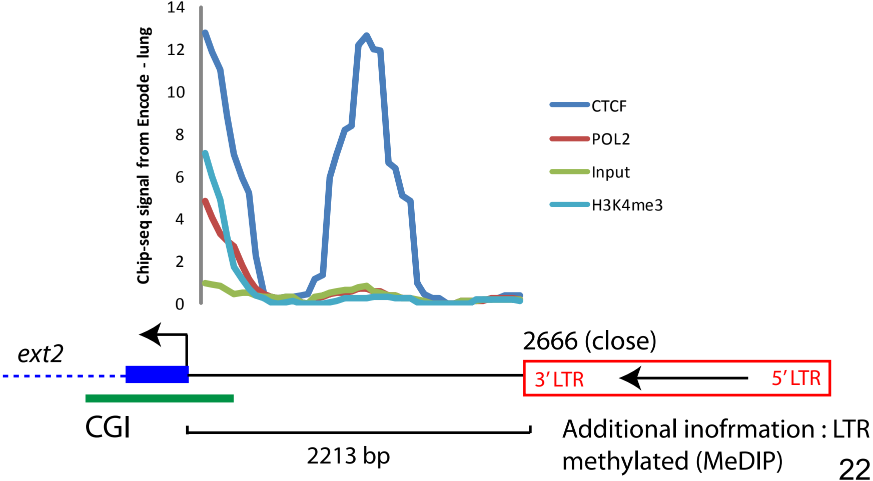

Chip-seq signal from Encode - lung
14
12
10
8
6
4
2
0
CTCF
POL2
Input
H3K4me3
ext2
CGI
2666 (close)
3' LTR
5' LTR
2213 bp
Additional inofrmation : LTR methylated (MeDIP)

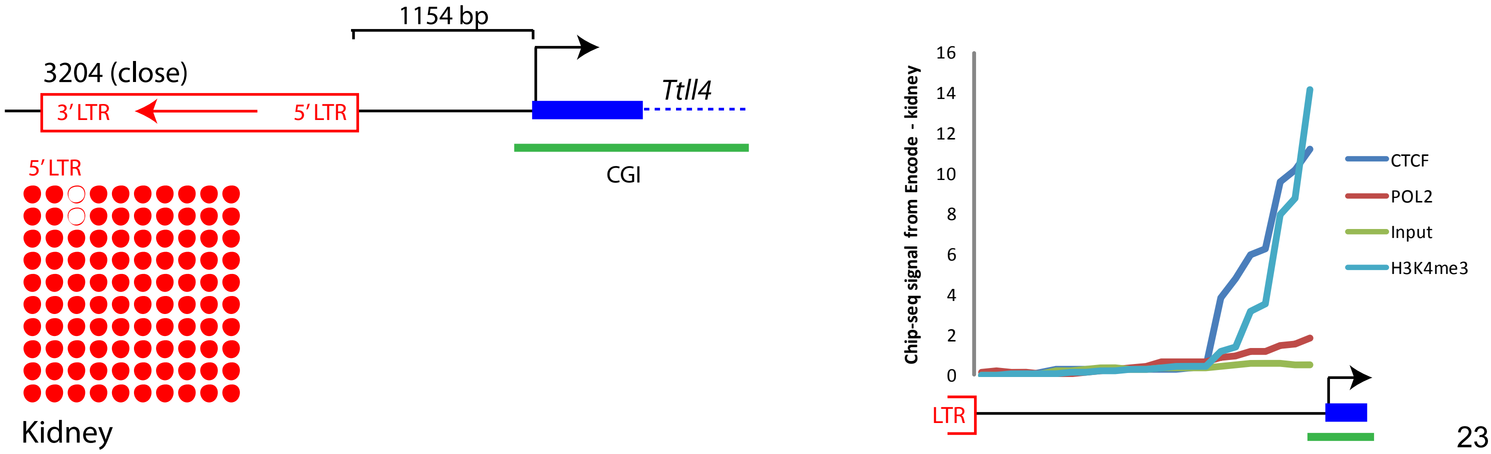

1154 bp
3204 (close)
3' LTR
5' LTR
Ttll4
CGI
5' LTR
Kidney
Chip-seq signal from Encode - kidney
16
14
12
10
8
6
4
2
0
CTCF
POL2
Input
H3K4me3
LTR

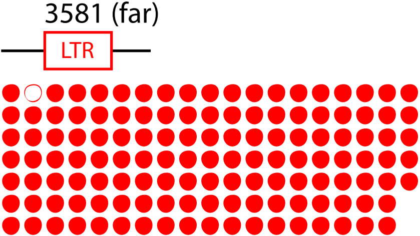

Pancreas

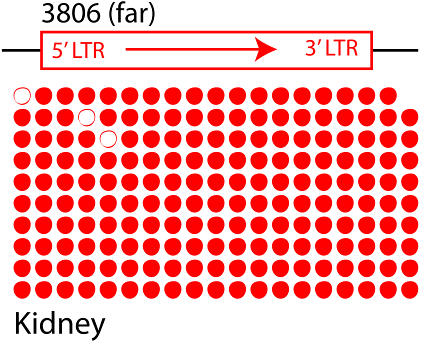
3806 (far)
5′ LTR
3′ LTR
Kidney

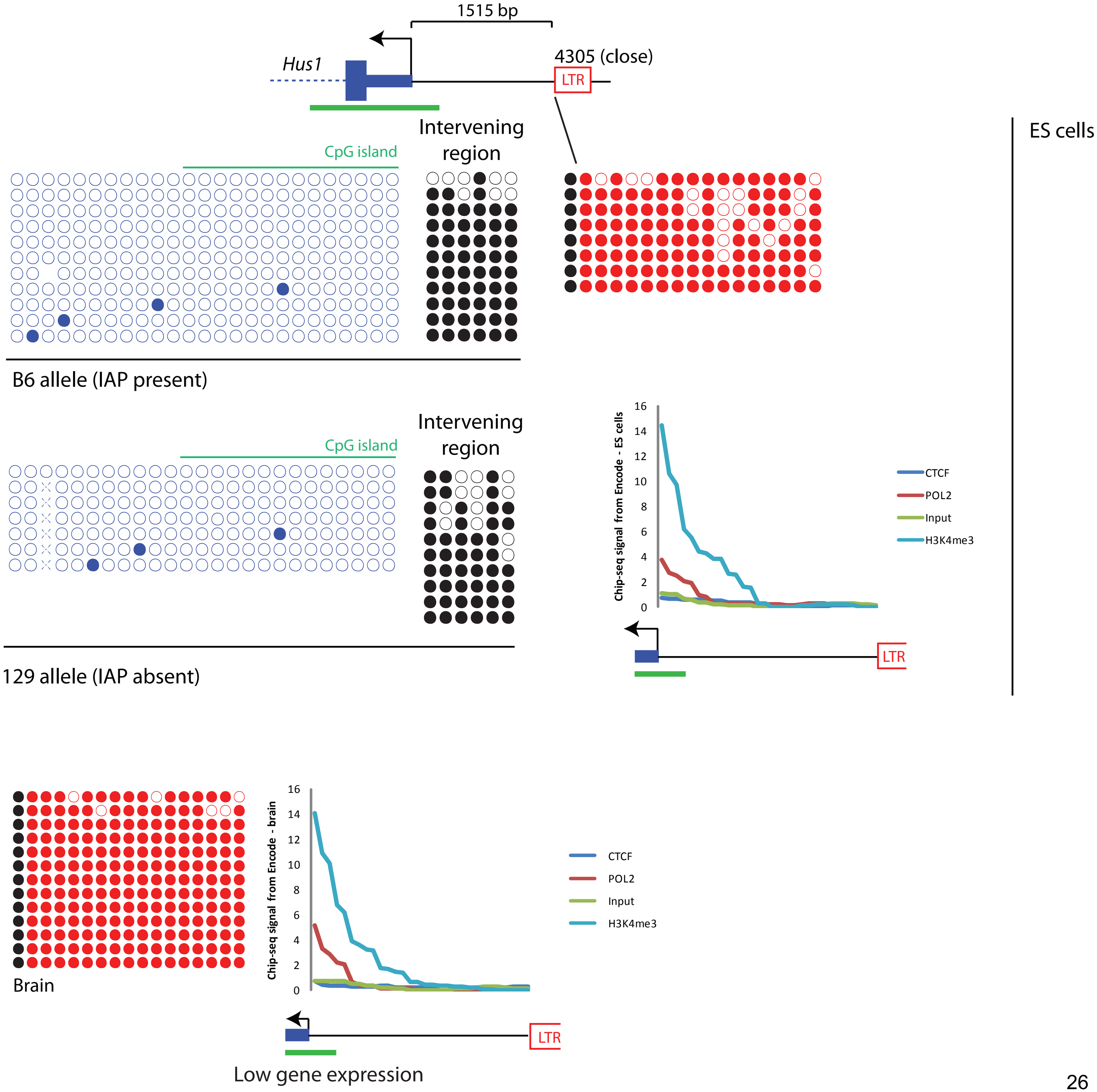
1515 bp
Hus1
4305 (close)
LTR
ES cells
Intervening region
CpG island
B6 allele (IAP present)
Intervening region
CpG island
129 allele (IAP absent)
Chip-seq signal from Encode - ES cells
16
14
12
10
8
6
4
2
0
CTCF
POL2
Input
H3K4me3
LTR
Brain
Chip-seq signal from Encode - brain
16
14
12
10
8
6
4
2
0
CTCF
POL2
Input
H3K4me3
LTR
Low gene expression

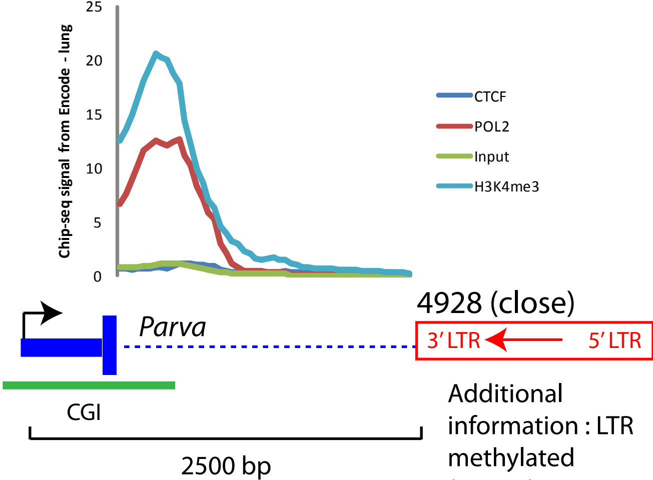

Additional information : LTR methylated (MeDIP)

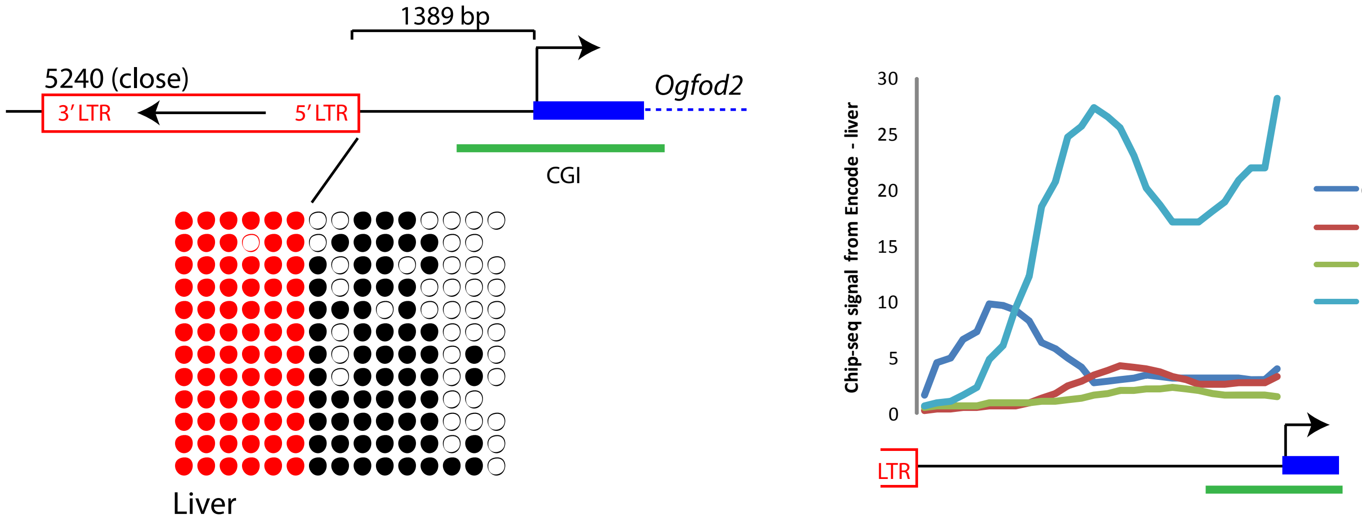
1389 bp
5240 (close)
3′ LTR
5′ LTR
Ogfod2
CGI
Liver
Chip-seq signal from Encode - liver
30
25
20
15
10
5
0
CTCF
POL2
Input
H3K4me3
LTR

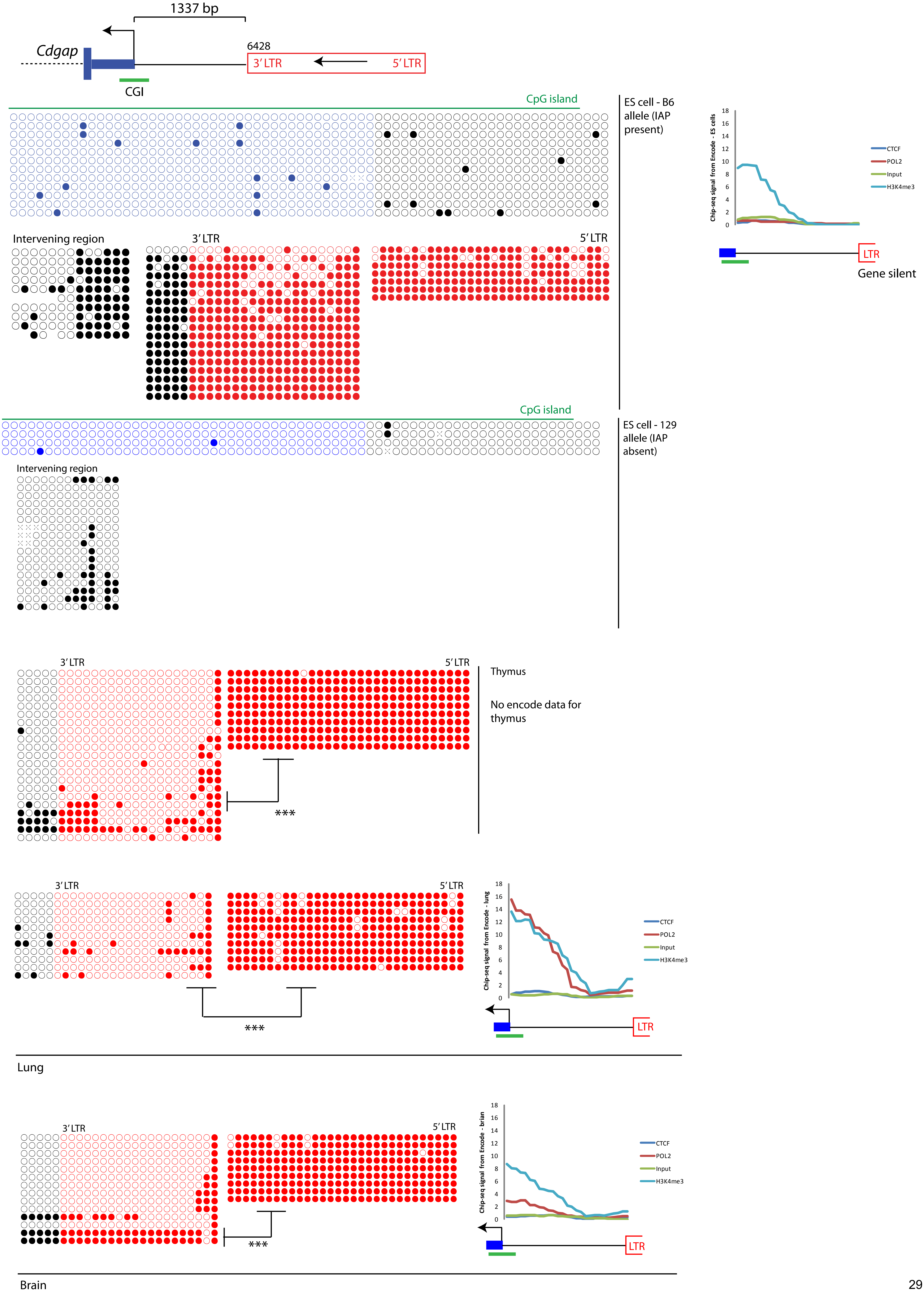
1337 bp
Cdgap
6428
3' LTR
5' LTR
CGI
CpG island
ES cell - B6 allele (IAP present)
Intervening region
3' LTR
5' LTR
Chip-seq signal from Encode - ES cells
CTCF
POL2
Input
H3K4me3
LTR
Gene silent
CpG island
ES cell - 129 allele (IAP absent)
Intervening region
3' LTR
5' LTR
Thymus
No encode data for thymus
***
3' LTR
5' LTR
***
Chip-seq signal from Encode - lung
CTCF
POL2
Input
H3K4me3
LTR
Lung
3' LTR
5' LTR
***
Chip-seq signal from Encode - brian
CTCF
POL2
Input
H3K4me3
LTR
Brain

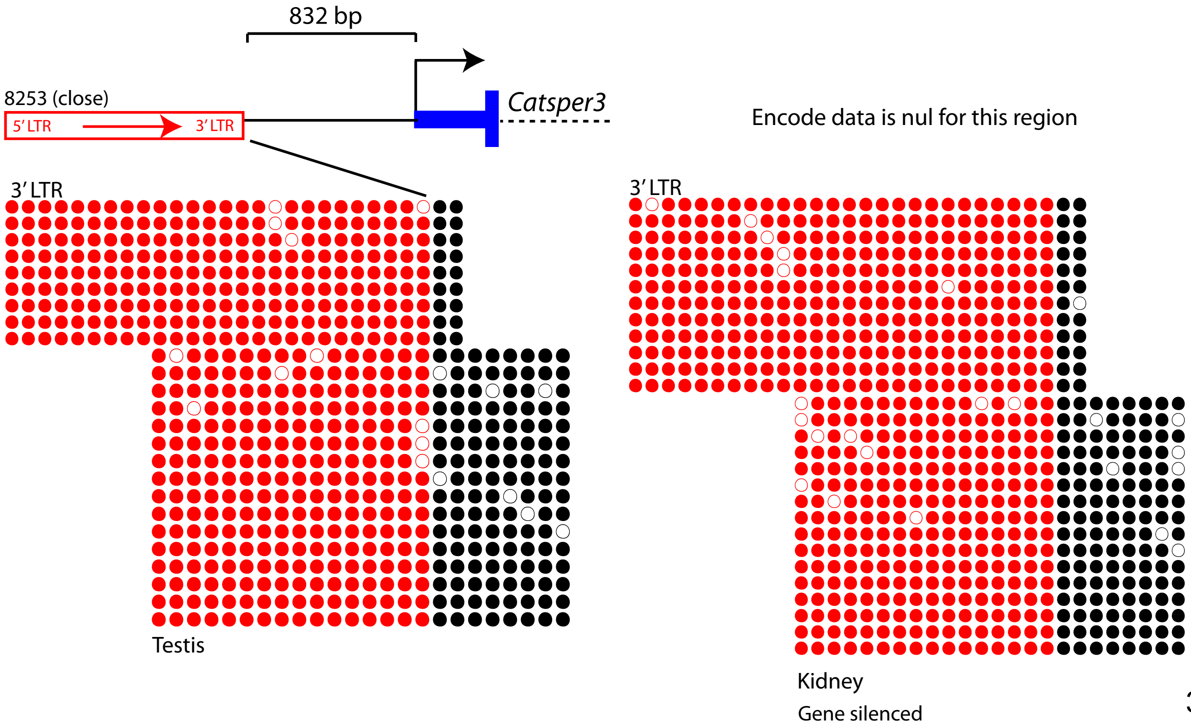
832 bp
8253 (close)
5′ LTR
3′ LTR
Catsper3
Encode data is nul for this region
3′ LTR
3′ LTR
Testis
Kidney
Gene silenced

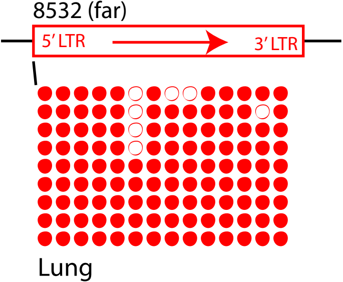

8532 (far)
5′ LTR
3′ LTR
Lung

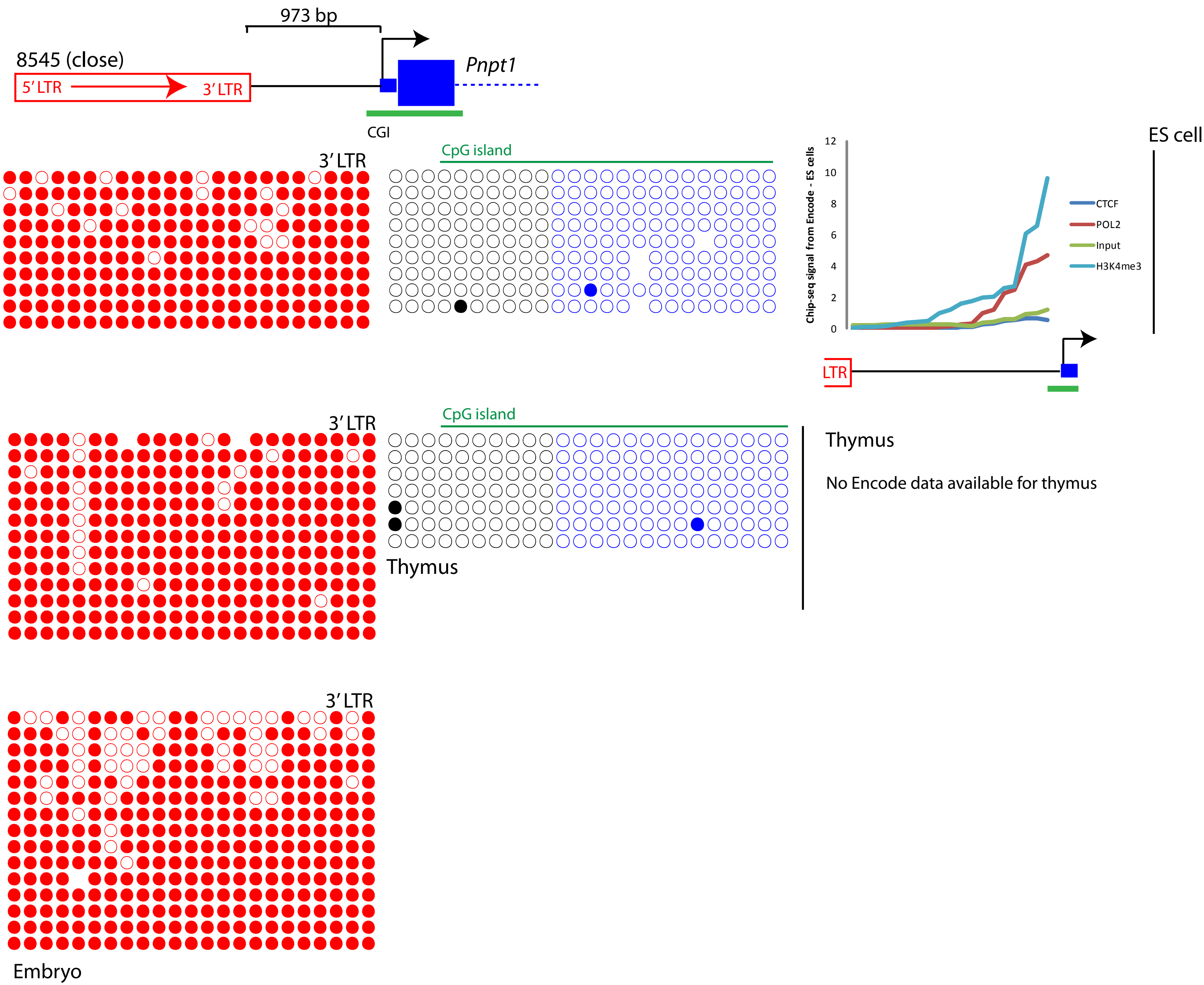

973 bp
8545 (close)
5′ LTR
3′ LTR
Pnpt1
CGI
3′ LTR
CpG island
Chip-seq signal from Encode - ES cells
12
10
8
6
4
2
0
CTCF
POL2
Input
H3K4me3
ES cell
LTR
3′ LTR
CpG island
Thymus
Thymus
No Encode data available for thymus
3′ LTR
Embryo

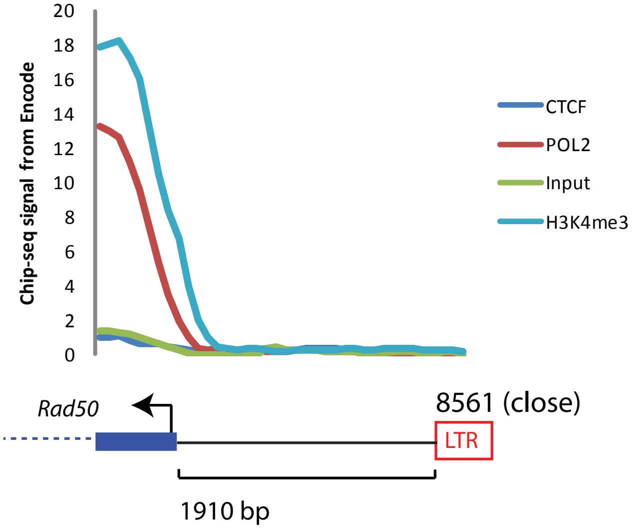

Additional information : LTR methylated (MeDIP)

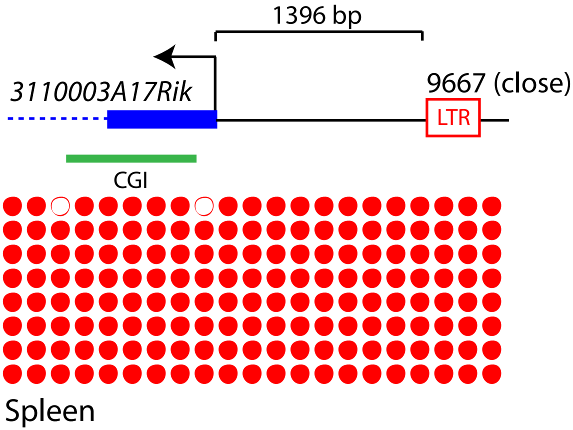
1396 bp
3110003A17Rik
9667 (close)
LTR
CGI
Spleen

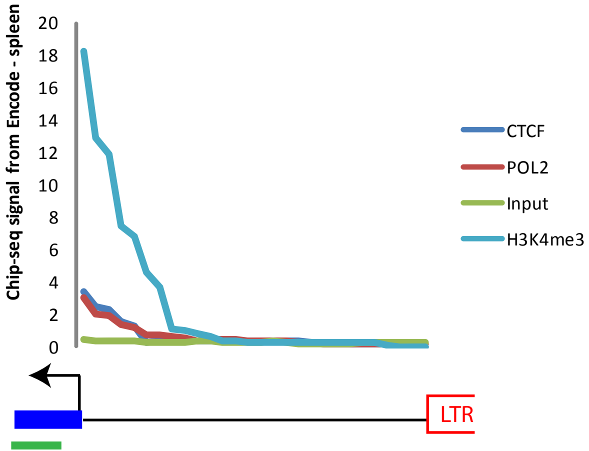
Chip-seq signal from Encode - spleen
20
18
16
14
12
10
8
6
4
2
0
CTCF
POL2
Input
H3K4me3
LTR

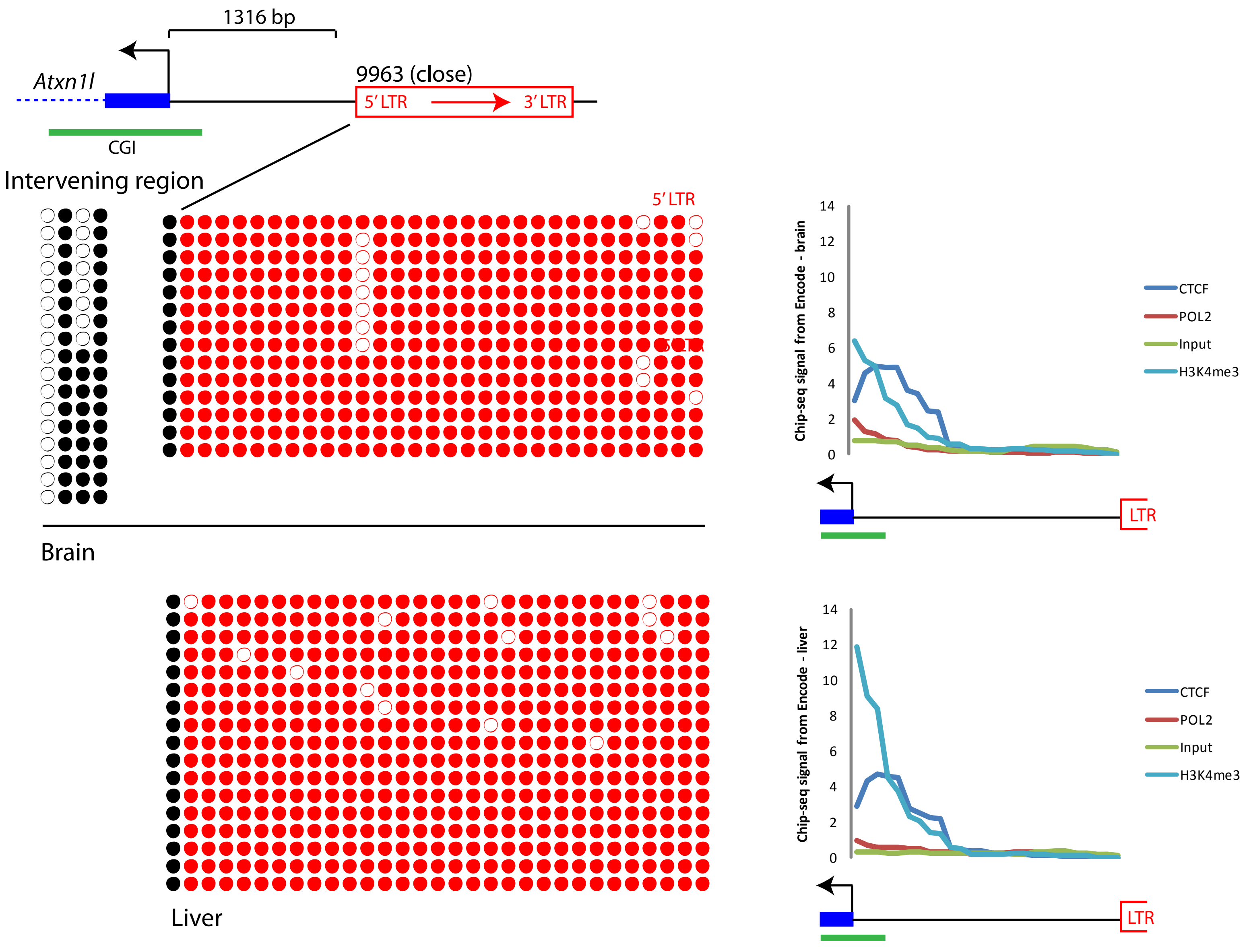

1316 bp
Atxn1l
9963 (close)
5′ LTR
3′ LTR
CGI
Intervening region
5′ LTR
Brain
Liver
Chip-seq signal from Encode - brain
Chip-seq signal from Encode - liver
14
12
10
8
6
4
2
0
CTCF
POL2
Input
H3K4me3
LTR

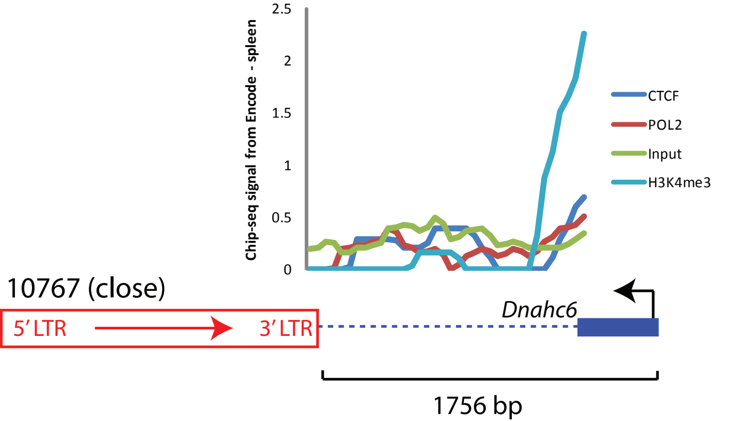

Additional information : LTR methylated (MeDIP)

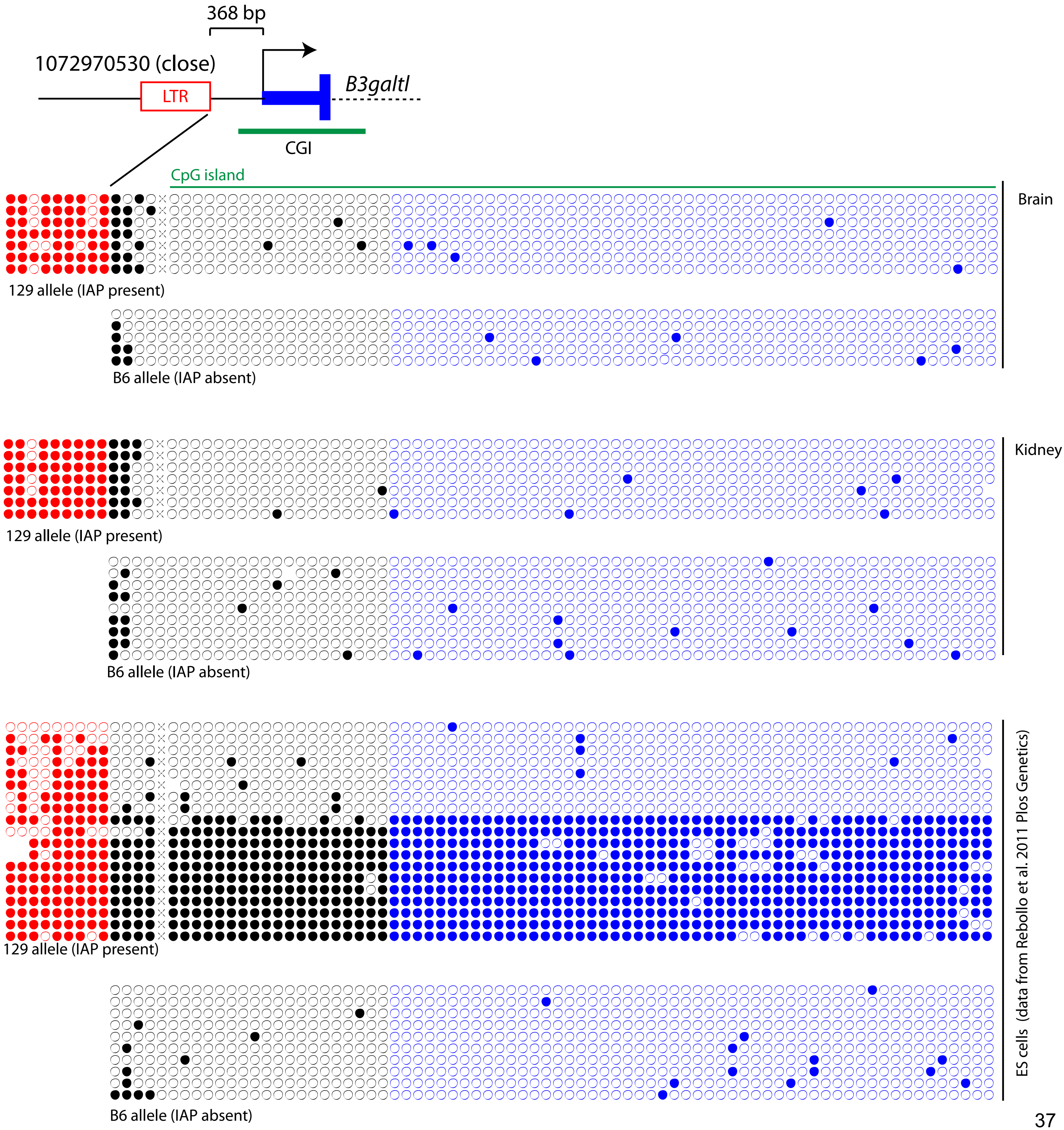

368 bp
1072970530 (close)
LTR
B3galtl
CGI
CpG island
Brain
129 allele (IAP present)
B6 allele (IAP absent)
Kidney
129 allele (IAP present)
B6 allele (IAP absent)
129 allele (IAP present)
B6 allele (IAP absent)
ES cells (data from Rebollo et al. 2011 Plos Genetics)

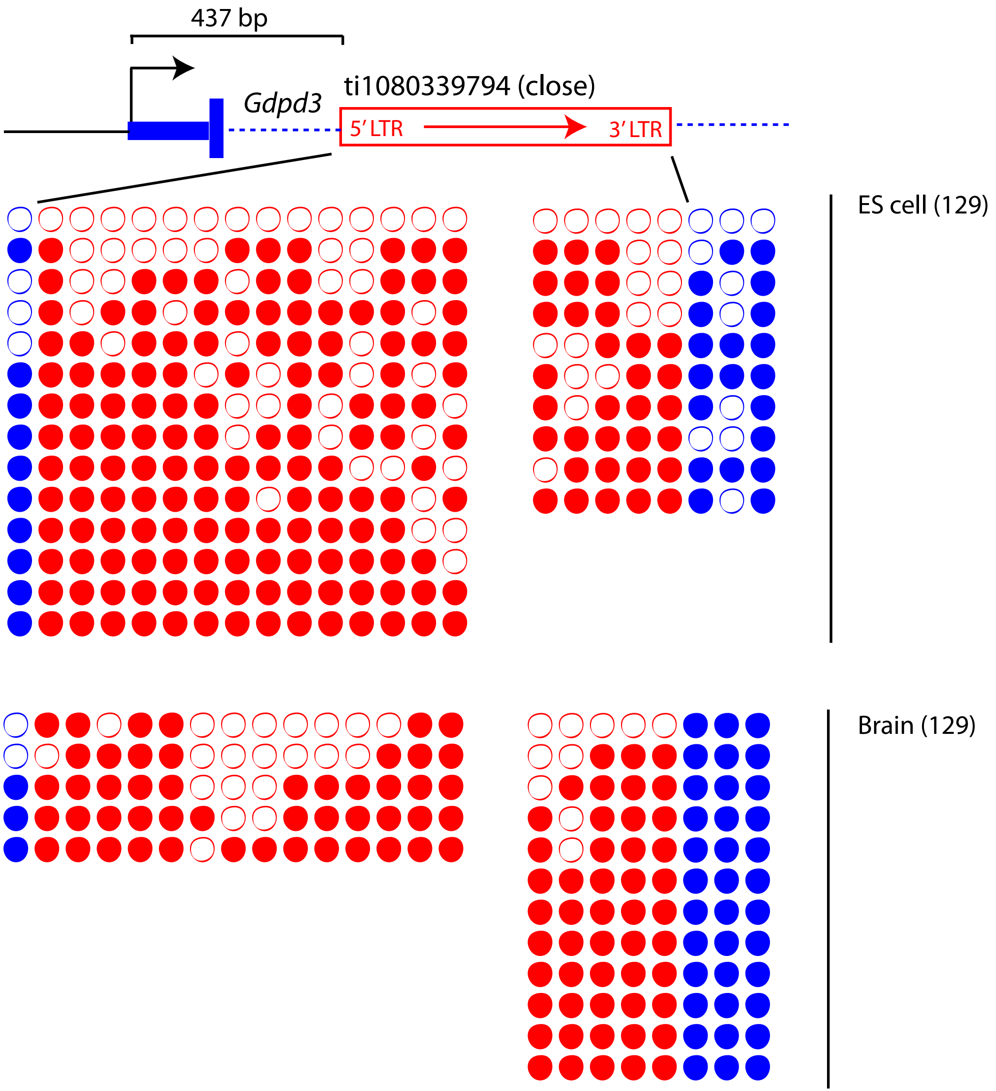
437 bp
Gdpd3
ti1080339794 (close)
5′ LTR
3′ LTR
ES cell (129)
Brain (129)

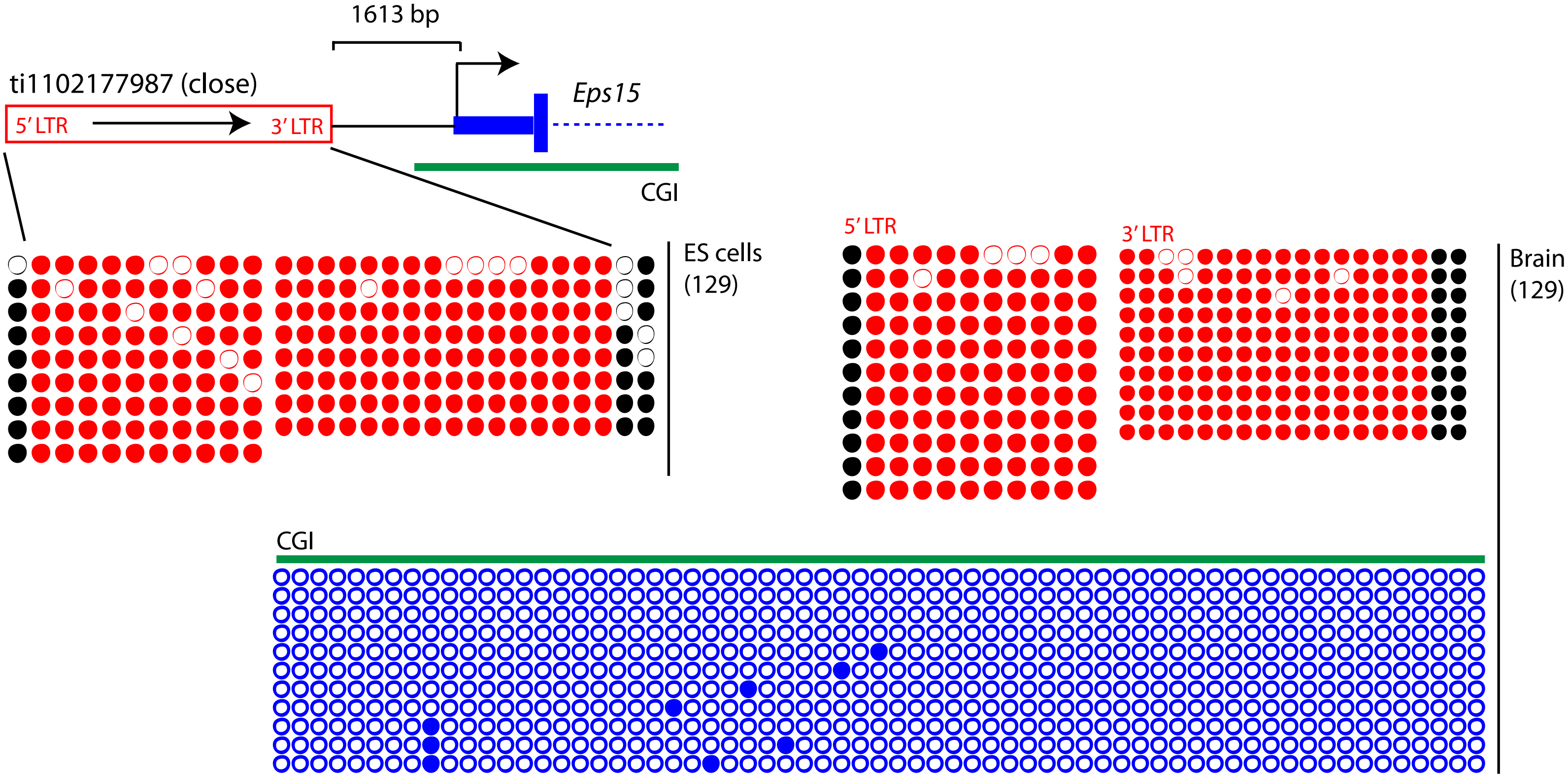
1613 bp
ti1102177987 (close)
5′ LTR
3′ LTR
Eps15
CGI
ES cells
(129)
5′ LTR
3′ LTR
Brain
(129)
CGI

Figure S3 - Copies far from genes

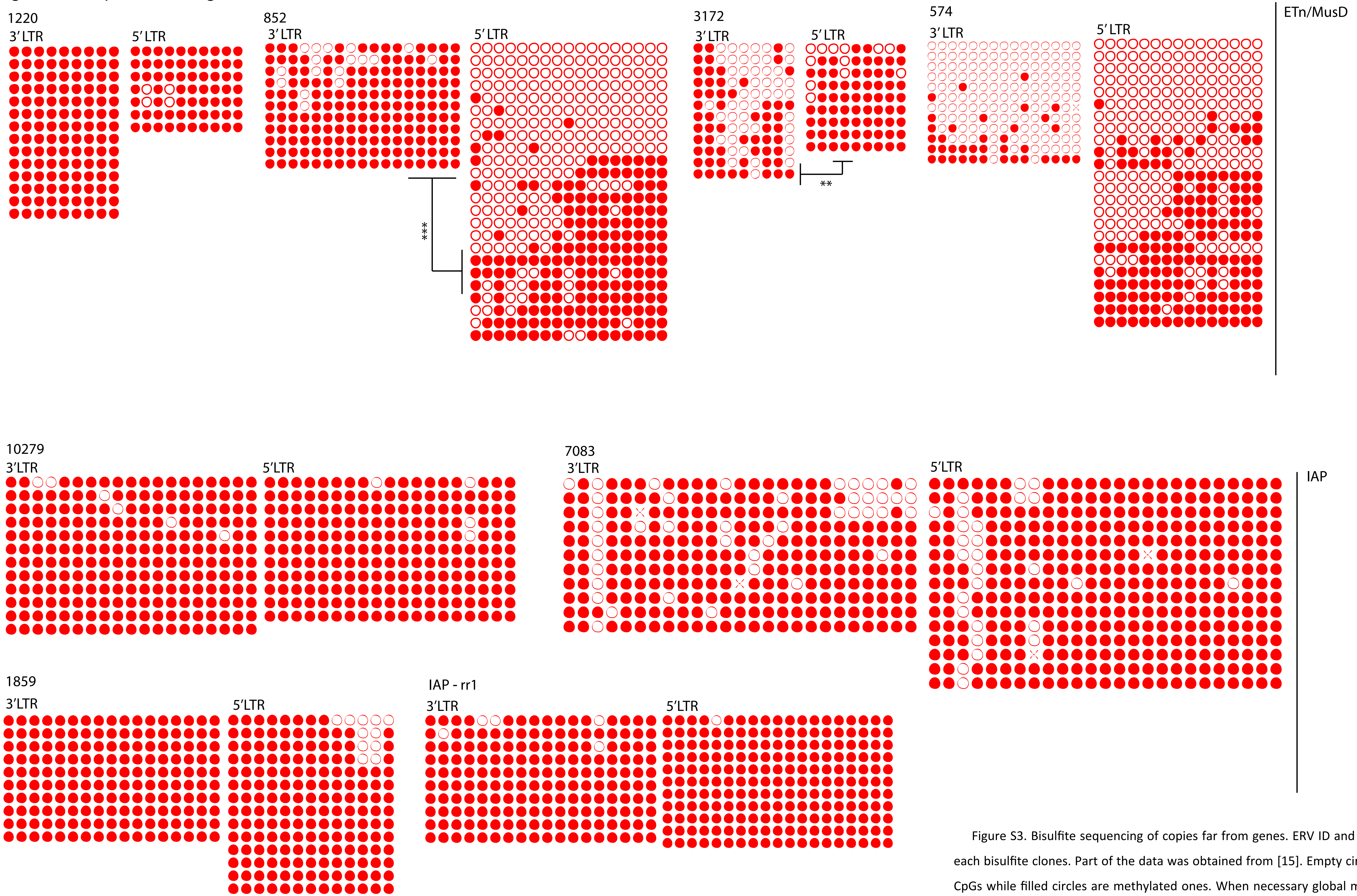

Figure S3. Bisulfite sequencing of copies far from genes. ERV ID and type are shown above each bisulfite clones. Part of the data was obtained from [15]. Empty circles are unmethylated CpGs while filled circles are methylated ones. When necessary global methylation profile was compared between ERV sequences with a Mann-Whitney U-test giving p values : *** < 0.001, ** < 0.01 and * < 0.05.

Figure S4

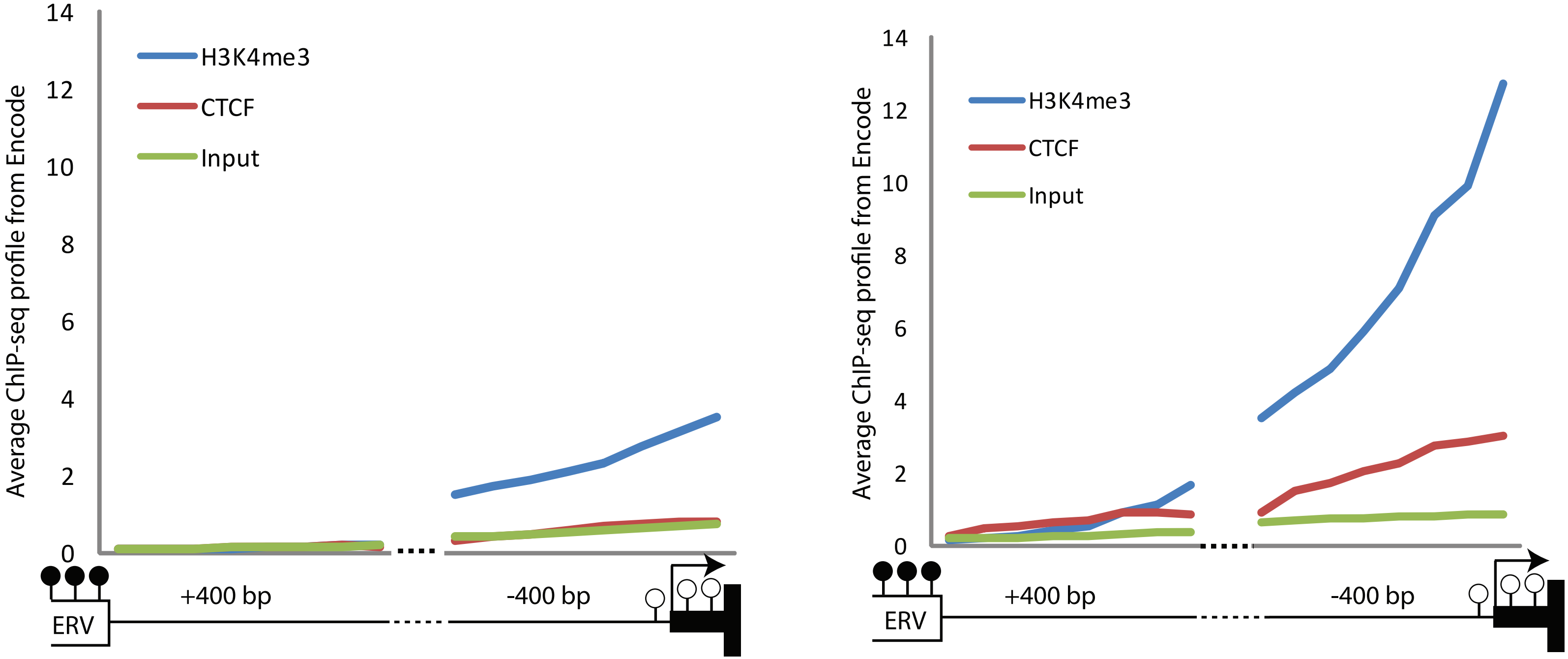

Figure S4. Chromatin environment of intervening regions of all methylated copies. The average H3K4me3 and CTCF profiles of the intervening regions between ERV and gene are shown. Gene regions represented on the left part show only H3K4me3 enrichment while regions represented on the right harbor both H3K4me3 and CTCF. The genes included in the data set are *Eef1e1, Gng10, Hus1, Pnpt1, Pol2, Parva, Rad50, Dnah* for the left panel and *Mthfd2l, Atxn1l, Ee1f1, Ttl4, Ogfod2, 3110003A17Rik* and *Ext2* for the right panel. The flanking regions chosen for this analysis (400bp and 200bp) correspond to a minimum length common for all regions analyzed (with the exception of *Cml2* which is 68bp from its ERV copy).

Figure S5

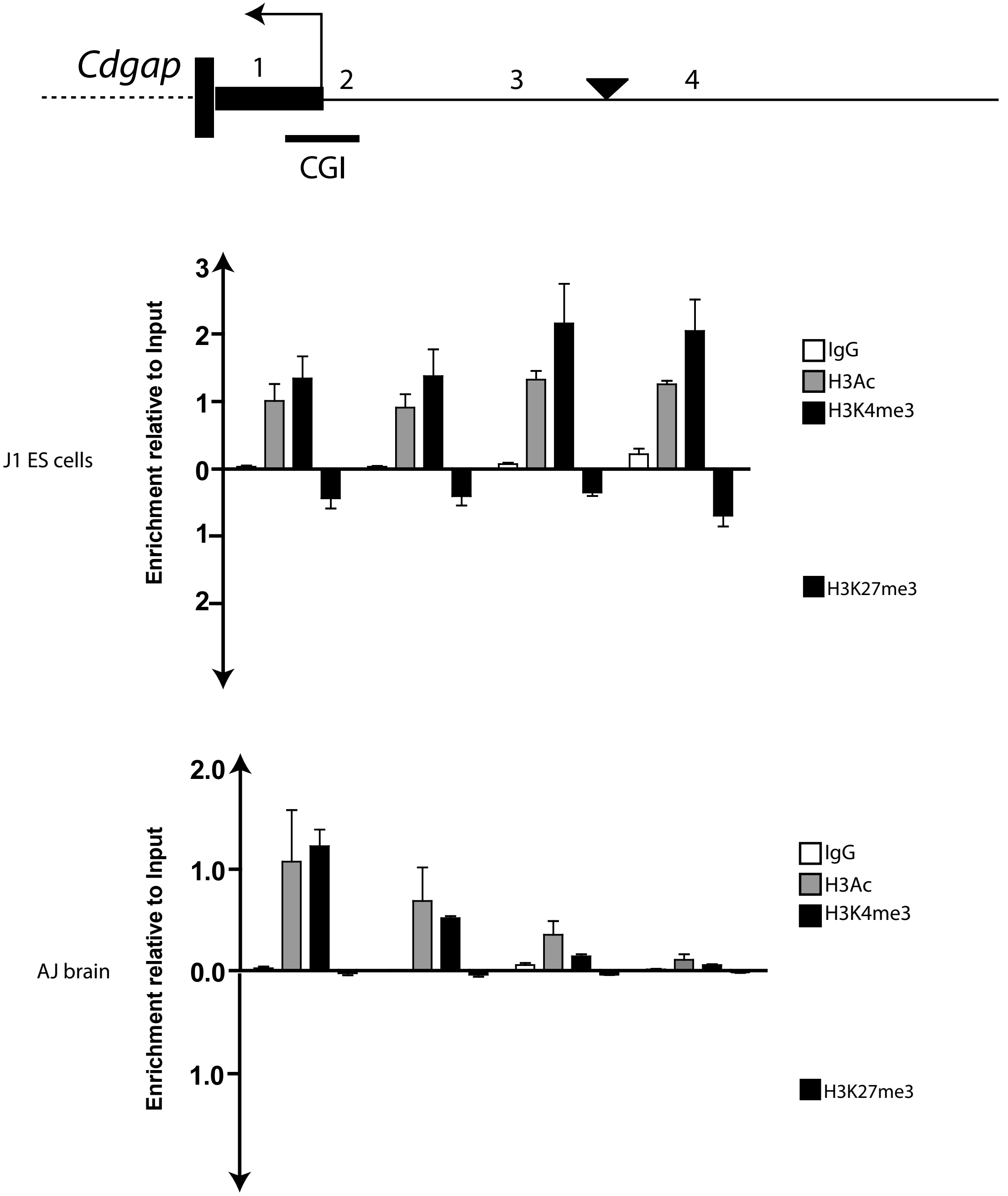

Figure S5. *Cdgap* ChIP-qPCR in strains where the nearby ERV is absent. The cartoon shows the site of insertion of the ERV copy (triangle). Dashed lines represent introns and numbers the localization of the quantitative PCR primer pairs. CGI : CpG Island. H3K27me3 enrichment is shown in opposite direction of H3K4me3.

Figure S6

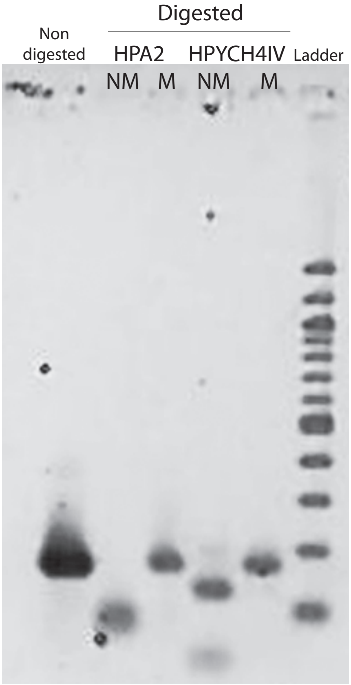

Figure S6. *In vitro* methylated DNA from *D. melanogaster* verification. Methylation of CpGs was verified through digestion with restriction enzymes sensitive to CpG methylation (HPYCH4IV and HPAII).
