# Supplementary material for: Epigenetic interplay between mouse endogenous retroviruses and host genes
Source: Genome Biol. 2012 Oct 3;13(10):R89. doi: 10.1186/gb-2012-13-10-r89 (PMC3491417; doi:10.1186/gb-2012-13-10-r89)
Supplement: Additional file 4 — All bisulfite sequencing data. Compilation of all bisulfite sequences. [file gb-2012-13-10-r89-S4.zip › IAP1859_TE_brain.docx]

Polymorphic IAP Case 74

3’LTR B6AJ Brain-Chr 6

>74LLTRBr_33

TAAGTAAAGTTTGAATGGAGGGTTATTTTTTTGGAGATATTGAGATTTTTGAGATAGTAG

GTAAGGAGTATATTTGTTATTCGATGCGTTTTTACGATCGGTTAGGAAGAACATAATAAT

TAGAATTTTTTACGGTAAAGTTTTATTGTTTACATTTTTATGGGGTTAGAGTGTAAGAAG

TAAGAGCGAGAGTAAGAGAGAGAGAAAAACGAAATTTTTTTTATTTTAAAGAGAATAATT

ATTGTTTAGGGCGTATTATTTTTTGATTGGTTGTAGTTTATGGTCGAGTTGACGTTTACG

GGAAAAATAGAGTATAAGTAGTCGTAAATATTTTTGGTTTATGCGTAGATTATTTGTTTA

TTAATTTAGAATATAGGATGTTAGCGTTATTTTGTGACGGCGAATGTGGGGGCGGTTTTT

TATAGTTTTTTTTTTTTTTTTTAATAAGAGTAAATAGGCTATTTATATTAATGAGAGTGG

AGATAGAGGTTAAATTTTTAGTGTGTAGGTAAAGGAGTTATGTATAGGATTAGTTTTTAG

GTTTATAGGTTTTTATTTAGAATAATTTTGATTTGTTTTCGTGTCGTTTTGTTTGGGGGA

AGGGAATTAGGATATTGAATTTTTATGAAAGATGATATGTTTTT

>74LLTRBr_35

TAAGTAAAGTTTGAATGGAGGGTTATTTTTTTGGAGATATTGAGATTTTTGAGATAGTAG

GTAAGGAGTATATTTGTTATTCGATGCGTTTTTACGATCGGTTAGGAAGAACATAATAAT

TAGAATTTTTTACGGTAAAGTTTTATTGTTTACATTTTTATGGGGTTAGAGTGTAAGAAG

TAAGAGCGAGAGTAAGAGAGAGAGAAAAACGAAATTTTTTTTATTTTAAAGAGAATAATT

ATTGTTTAGGGCGTATTATTTTTTGATTGGTTGTAGTTTATGGTCGAGTTGACGTTTACG

GGAAAAATAGAGTATAAGTAGTCGTAAATATTTTTGGTTTATGCGTAGATTATTTGTTTA

TTAATTTAGAATATAGGATGTTAGCGTTATTTTGTGACGGCGAATGTGGGGGCGGTTTTT

TATAGTTTTTTTTTTTTTTTTAATAAGAGTAAATAGGCTATTTATATTAATGAGAGTGGA

GATAGAGGTTAAATTTTTAGTGTGTAGGTAAAGGAGTTATGTATAGGATTAGTTTTTAGG

TTTATAGGTTTTTATTTAGAATAATTTTGATTTGTTTTCGTGTCGTTTTGTTTGGGGGAA

GGGAATTAGGATATTGAATTTTTATGAAAGATGATATGTTTTT

>74LLTRBr_36

TAAGTAAAGTTTGAATGGAGGGTTATTTTTTTGGAGATATTGAGATTTTTGAGATAGTAG

GTAAGGAGTATATTTGTTATTCGATGCGTTTTTACGATCGGTTAGGAAGAACATAATAAT

TAGAATTTTTTACGGTAAAGTTTTATTGTTTACATTTTTATGGGGTTAGAGTGTAAGAAG

TAAGAGCGAGAGTAAGAGAGAGAGAAAAACGAAATTTTTTTTATTTTAAAGAGAATAATT

ATTGTTTAGGGCGTATTATTTTTTGATTGGTTGTAGTTTATGGTCGAGTTGACGTTTACG

GGAAAAATAGAGTATAAGTAGTCGTAAATATTTTTGGTTTATGCGTAGATTATTTGTTTA

TTAATTTAGAATATAGGATGTTAGCGTTATTTTGTGACGGCGAATGTGGGGGCGGTTTTT

TATAGTTTTTTTTTTTTTTTTTAATAAGAGTAAATAGGCTATTTATATTAATGAGAGTGG

AGATAGAGGTTAAATTTTTAGTGTGTAGGTAAAGGAGTTATGTATAGGATTAGTTTTTAG

GTTTATAGGTTTTTATTTAGAATAATTTTGATTTGTTTTCGTGTCGTTTTGTTTGGGGGA

AGGGAATTAGGATATTGAATTTTTATGAAAGATGATATGTTTT

>74LLTRBr_37

TAAAGTTTGAATGGAGGGTTATTTTTTTGGAGATATTGAGATTTTTGAGATAGTAGGTAA

GGAGTACATTTGTTATTCGATGCGTTCTTACGATCGGTTAGGAAGAACATAATAATTAGA

ATTTTTTACGGTAAAGTTTTATTGTTTACATTTTTATGGGGTTAGAGTGTAAGAAGTAAG

AGCGAGAGTAAGAGAGAGAGAAAAACGAAATTTTTTTTATTTTAAAGAGAATAATTATTG

TTTAGGGCGTATCACTTTTTGATTGGTTGTAGTTTGTGGTCGAGTTGACGTTTACGGGAA

AAATAGAGTATAAGTAGTCGTAAATATTTTTGGTTTATGCGTAGATTATTTGTTTATTAA

TTTAGAATATAGGATGTTAGCGTTATTTTGTGACGGCGAATGTGGGGGCGGTTTTTTATA

GTTTTTTTTTTTTTTTTTAATAAGAGTAAATAGGTTATTTATATTAATGAGAGTGGAGAT

AGAGGTTAAATTTTTAGTGTGTAGGTAAAGGAGTCATGTATAGGATTAGTTTTTAGGTTT

ACAGGTTTTTATTTAGAATAATTTTGATTTGTTTTTGTGTCGTTTTGTTTGGGGGAAGGG

AATTAGGATATTGAATTTTTATGAAAGATGATATGTTTTT

>74LLTRBr_38

TAAGTAAAGTTTGAATGGAGGGTTATTTTTTTGGAGATATTGAGATTTTTGAGATAGTAG

GTAAGGAGTACATTTGTTATTCGATGCGTTCTTACGATCGGTTAGGAAGAACATAATAAT

TAGAATTTTTTACGGTAAAGTTTTATTGTTTACATTTTTATGGGGTTAGAGTGTAAGAAG

TAAGAGCGAGAGTAAGAGAGAGAGAAAAACGAAATTTTTTTTATTTTAAAGAGAATAATT

ATTGTTTAGGGCGTATCACTTTTTGATTGGTTGTAGTTCATGGTCGAGTTGACGTTTACG

GGAAAAATAGAGTATAAGTAGTCGTAAATATTTTTGGTTTATGCGTAGATTATTTGTTTA

TTAATTTAGAATATAGGATGTTAGCGTTATTTTGTGACGGCGAATGTGGGGGCGGTTTTT

TATAGTTTTTTTTTTTTTTTTTAATAAGAGTAAATAGGTTATTTATATTAATGAGAGTGG

AGATAGAGGTTAAATTTTTAGTGTGTAGGTAAAGGAGTCATGTATAGGATTAGTTTTTAG

GTTTACAGGTTTTTATTTAGAATAATTTTGATTTGTTTTTGTGTCGTTTTGTTTGGGGGA

AGGGAATTAGGATATTGAATTTTTATGAAAGATGATATGTTTTT

>74LLTRBr_41

TAAGTAAAGTTTGAATGGAGGGTTATTTTTTTGGAGATATTGAGATTTTTGAGATAGTAG

GTAAGGAGTATATTTGTTATTCGATGCGTTTTTACGATCGGTTAGGAAGAACATAATAAT

TAGAATTTTTTACGGTAAAGTTTTATTGTTTACATTTTTATGGGGTTAGAGTGTAAGAAG

TAAGAGCGAGAGTAAGAGAGAGAGAAAAACGAAATTTTTTTTATTTTAAAGAGAATAATT

ATTGTTTAGGGCGTATTATTTTTTGATTGGTTGTAGTTTATGGTCGAGTTGACGTTTACG

GGAAAAATAGAGTATAAGTAGTCGTAAATATTTTTGGTTTATGCGTAGATTATTTGTTTA

TTAATTTAGAATATAGGATGTTAGCGTTATTTTGTGACGGCGAATGTGGGGGCGGTTTTT

TATAGTTTTTTTTTTTTTTTTTATAAGAGTAAATAGGCTATTTATATTAATGAGAGTGGA

GATAGAGGTTAAATTTTTAGTGTGTAGGTAAAGGAGTTATGTATAGGATTAGTTTTTAGG

TTTATAGGTTTTTATTTAGAATAATTTTGATTTGTTTTCGTGTCGTTTTGTTTGGGGGAA

GGGAATTAGGATATTGAATTTTTATGAAAGATGATATGTTTTT

>74LLTRBr_42

TAAGTAAAGTTTGAATGGAGGGTTATTTTTTTGGAGATATTGAGATTTTTGAGATAGTAG

GTAAGGAGTACATTTGTTATTCGATGCGTTCTTACGATCGGTTAGGAAGAACATAACAAT

TAGAATTTTTTACGGTAAAGTTTTATTGTTTACATTTTTATGGGGTTAGAGTGTAAGAAG

TAAGAGCGAGAGTAAGAGAGAGAGAAAAACGAAATTTTTTTTATTTTAAAGAGAATAATT

ATTGTTTAGGGCGTATCACTTTTTGATTGGTTGTAGTTTATGGTCGAGTTGACGTTTACG

GGAAAAATAGAGTATAAGTAGTCGTAAATATTTTTGGTTTATGCGTAGATTATTTGTTTA

TTAATTTAGAATATAGGATGTTAGCGTTATTTTGTGACGGCGAATGTGGGGGCGGTTTTT

TATAGTTTTTTTTTTTTTTTTTAATAAGAGTAAATAGGTTATTTATATTAATGAGAGTGG

AGATAGAGGTTAAATTTTTAGTGTGTAGGTAAAGGAGTCATGTATAGGATTAGTTTTTAG

GTTTACAGGTTTTTATTTAGAATAATTTTGATTTGTTTTTGTGTCGTTTTGTTTGGGGGA

AGGGAATTAGGATATTGAATTTTTATGAAAGATGATATGTTTTT

>74LLTRBr_43

TAAGTAAAGTTTGAATGGAGGGTTATTTTTTTGGAGATATTGAGATTTTTGAGATAGTAG

GTAAGGAGTATATTTGTTATTCGATGCGTTTTTACGATCGGTTAGGAAGAACATAATAAT

TAGAATTTTTTACGGTAAAGTTTTATTGTTTACATTTTTATGGGGTTAGAGTGTAAGAAG

TAAGAGCGAGAGTAAGAGAGAGAGAAAAACGAAATTTTTTTTATTTTAAAGAGAATAATT

ATTGTTTAGGGCGTATTATTTTTTGATTGGTTGTAGTTTATGGTCGAGTTGACGTTTACG

GGAAAAATAGAGTATAAGTAGTCGTAAATATTTTTGGTTTATGCGTAGATTATTTGTTTA

TTAATTTAGAATATAGGATGTTAGCGTTATTTTGTGACGGCGAATGTGGGGGCGGTTTTT

ATAGTTTTTTTTTTTTTTTTTAATAAGAGTAAATAGGCTATTTATATTAATGAGAGTGGA

GATAGAGGTTAAATTTTTAGTGTGTAGGTAAAGGAGTTATGTATAGGATTAGTTTTTAGG

TTTATAGGTTTTTATTTAGAATAATTTTGATTTGTTTTCGTGTCGTTTTGTTTGGGGGAA

GGGAATTAGGATATTGAATTTTTATGAAAGATGATATGTTTTT

>74LLTRBr_44

TAAGTAAAGTTTGAATGGAGGGTTATTTTTTTGGAGATATTGAGATTTATGAGATAGTAG

GTAAGGAGTACATTTGTTATTCGATGCGTTCTTACGATCGGTTAGGAAGAACATAATAAT

TAGAATTTTTTACGGTAAAGTTTTATTGTTTACATTTTTATGGGGTTAGAGTGTAAGAAG

TAAGAGCGAGAGTAAGAGAGAGAGAAAAACGAAATTTTTTTTATTTTAAAGAGAATAATT

ATTGTTTAGGGCGTATCACTTTTTGATTGGTTGTAGTTTATGGTCGAGTTGACGTTTACG

GGAAAAATAGAGTATAAGTAGTCGTAAATATTTTTGGTTTATGCGTAGATTATTTGCTTA

TTAATTTAGAATATAGGATGTTAGCGTTATTTTGTGACGGCGAATGTGGGGGCGGTTTTT

TATAGTTTTTTTTTTTTTTTTAATAAGAGTAAATAGGTTATTTATATTAATGAGAGTGGA

GATAGAGGTTAAATTTTTAGTGTGTAGGTAAAGGAGTCATGTATAGGATTAGTTTTTAGG

TTTACAGGTTTTTATTTAGAATAATTTTGATTTGTTTTTGTGTCGTTTTGTTTGGGGGAA

GGGAATTAGGATATTGAATTTTTATGAAAGATGATATGTTTTT

>74LLTRBr_45

TAAGTAAAGTTTGAATGGAGGGTTATTTTTTTGGAGATATTGAGATTTTTGAGATAGTAG

GTAAGGAGTATATTTGTTATTCGATGCGTTTTTACGATCGGTTAGGAAGAACATAATAAT

TAGAATTTTTTACGGTAAAGTTTTATTGTTTACATTTTTATGGGGTTAGAGTGTAAGAAG

TAAGAGCGAGAGTAAGAGAGAGAGAAAAACGAAATTTTTTTTATTTTAAAGAGAATAATT

ATTGTTTAGGGCGTATTATTTTTTGATTGGTTGTAGTTTATGGTCGAGTTGACGTTTACG

GGAAAAATAGAGTATAAGTAGTCGTAAATATTTTTGGTTTATGCGTAGATTATTTGTTTA

TTAATTTAGAATATAGGATGTTAGCGTTATTTTGTGACGGCGAATGTGGGGGCGGTTTTT

TATAGTTTTTTTTTTTTTTTTAATAAGAGTAAATAGGCTATTTATATTAATGAGAGTGGA

GATAGAGGTTAAATTTTTAGTGTGTAGGTAAAGGAGTTATGTATAGGATTAGTTTTTAGG

TTTATAGGTTTTTATTTAGAATAATTTTGATTTGTTTTCGTGTCGTTTTGTTTGGGGGAA

GGGAATTAGGATATTGAATTTTTATGAAAGATGATATGTTTTT

>74LLTRBr_46

TAAGTAAAGTTTGAATGGAGGGTTATTTTTTTGGAGATATTGAGATTTTTGAGATAGTAG

GTAAGGAGTATATTTGTTATTCGATGCGTTTTTACGATCGGTTAGGAAGAACATAATAAT

TAGAATTTTTTACGGTAAAGTTTTATTGTTTACATTTTTATGGGGTTAGAGTGTAAGAAG

TAAGAGCGAGAGTAAGAGAGAGAGAAAAACGAAATTTTTTTTATTTTAAAGAGAATAATT

ATTGTTTAGGGCGTATTATTTTTTGATTGGTTGTAGTTTATGGTCGAGTTGACGTTTACG

GGAAAAATAGAGTATAAGTAGTCGTAAATATTTTTGGTTTATGCGTAGATTATTTGTTTA

TTAATTTAGAATATAGGGTGTTAGCGTTATTTTGTGACGGCGAATGTGGGGGCGGTTTTT

TATAGTTTTTTTTTTTTTTTTTAATAAGAGTAAATAGGCTATTTATATTAATGAGAGTGG

AGATAGAGGTTAAATTTTTAGTGTGTAGGTAAAGGAGTTATGTATAGGATTAGTTTTTAG

GTTTATAGGTTTTTATTTAGAATAATTTTGATTTGTTTTCGTGTCGTTTTGTTTGGGGGA

AGGGAATTAGGATATTGAATTTTTATGAAAGATGATATGTTTT

>74LLTRBr_47

TAAGTAAAGTTTGAATGGAGGGTTATTTTTTTGGAGATATTGAGATTTTTGAGATAGTAG

GTAAGGAGTACATTTGTTATTCGATGCGTTCTTACGATCGGTTAGGAAGAACATAATAAT

TAGAATTTTTTACGGTAAAGTTTTATTGTTTACATTTTTATGGGGTTAGAGTGTAAGAAG

TAAGAGCGAGAGTAAGAGAGAGAGAAAAACGAAATTTTTTTTATTTTAAAGAGAATAATT

ATTGTTTAGGGCGTATCACTTTTTGATTGGTTGTAGTTTATGGTCGAGTTGACGTTTACG

GGAAAAATAGAGTATAAGTAGTCGTAAATATTTTTGGTTTATGCGTAGATTATTTGTTTA

TTAATTTAGAATATAGGATGTTAGCGTTATTTTGTGACGGCGAATGTGGGGGCGGTTTTT

TATAGTTTTTTTTTTTTTTTTTAATAAGAGTAAATAGGTTATTTATATTAATGAGAGTGG

AGATAGAGGTTAAATTTTTAGTGTGTAGGTAAAGGAGTCATGTATAGGATTAGTTTTTAG

GTTTACAGGTTTTTATTTAGAATAATTTTGATTTGTTTTTGTGTCGTTTTGTTTGGGGGA

AGGGAATTAGGATATTGAATTTTTATGAAAGATGATATGTTTTT

>74LLTRBr_48

TAAGTAAAGTTTGAATGGAGGGTTATTTTTTTGGAGATATTGAGATTTTTGAGATAGTAG

GTAAGGAGTATATTTGTTATTCGATGCGTTTTTACGATCGGTTAGGAAGAACATAATAAT

TAGAATTTTTTACGGTAAAGTTTTATTGTTTACATTTTTATGGGGTTAGAGTGTAAGAAG

TAAGAGCGAGAGTAAGAGAGAGAGAAAAACGAAATTTTTTTTATTTTAAAGAGAATAATT

ATTGTTTAGGGCGTATTATTTTTTGATTGGTTGTAGTTTATGGTCGAGTTGACGTTTACG

GGAAAAATAGAGTATAAGTAGTCGTAAATATTTTTGGTTTATGCGTAGATTATTTGTTTA

TTAATTTAGAATATAGGATGTTAGCGTTATTTTGTGACGGCGAATGTGGGGGCGGTTTTT

TATAGTTTTTTTTTTTTTTTTTAATAAGAGTAAATAGGCTATTTATATTAATGAGAGTGG

AGATAGAGGTTAAATTTTTAGTGTGTAGGTAAAGGAGTTATGTATAGGATTAGTTTTTAG

GTTTATAGGTTTTTATTTAGAATAATTTTGATTTGTTTTCGTGTCGTTTTGTTTGGGGGA

AGGGAATTAGGATATTGAATTTTTATGAAAGATGATATGTTTTT

>74LLTRBr_49

TAAGTAAAGTTTGAATGGAGGGTTATTTTTTTGGAGATATTGAGATTTTTGAGATAGTAG

GTAAGGAGTATATTTGTTATTCGATGCGTTTTTACGATCGGTTAGGAAGAACATAATAAT

TAGAATTTTTTACGGTAAAGTTTTATTGTTTACATTTTTATGGGGTTAGAGTGTAAGAAG

TAAGAGCGAGAGTAAGAGAGAGAGAAAAACGAAATTTTTTTTATTTTAAAGAGAATAATT

ATTGTTTAGGGCGTATTATTTTTTGATTGGTTGTAGTTTATGGTCGAGTTGACGTTTACG

GGAAAAATAGAGTATAAGTAGTCGTAAATATTTTTGGTTTATGCGTAGATTATTTGTTTA

TTAATTTAGAATATAGGATGTTAGCGTTATTTTGTGACGGCGAATGTGGGGGCGGTTTTT

TATAGTTTTTTTTTTTTTTTTAATAAGAGTAAATAGGCTATTTATATTAATGAGAGTGGA

GATAGAGGTTAAATTTTTAGTGTGTAGGTAAAGGAGTTATGTATAGGATTAGTTTTTAGG

TTTATAGGTTTTTATTTAGAATAATTTTGATTTGTTTTCGTGTCGTTTTGTTTGGGGGGA

GGGAATTAGGATATTGAATTTTTATGAAAGATGATATGTTTTT

>74LLTRBr_50

TAAGTAAAGTTTGAATGGAGGGTTATTTTTTTGGAGATATTGAGATTTTTGAGATAGTAG

GTAAGGAGTATATTTGTTATTCGATGCGTTTTTACGATCGGTTAGGAAGAACATAATAAT

TAGAATTTTTTACGGTAAAGTTTTATTGTTTACATTTTTATGGGGTTAGAGTGTAAGAAG

TAAGAGCGAGAGTAAGAGAGAGAGAAAAACGAAATTTTTTTTATTTTAAAGAGAATAATT

ATTGTTTAGGGCGTATTATTTTTTGATTGGTTGTAGTTTATGGTCGAGTTGACGTTTACG

GGAAAAATAGAGTATAAGTAGTCGTAAATATTTTTGGTTTATGCGTAGATTATTTGTTTA

TTAATTTAGAATATAGGATGTTAGCGTTATTTTGTGACGGCGAATGTGGGGGCGGTTTTT

TATAGTTTTTTTTTTTTTTTTTAATAAGAGTAAATAGGCTATTTATATTAATGAGAGTGG

AGATAGAGGTTAAATTTTTAGTGTGTAGGTAAAGGAGTTATGTATAGGATTAGTTTTTAG

GTTTATAGGTTTTTATTTAGAATAATTTTGATTTGTTTTCGTGTCGTTTTGTTTGGGGGA

AGGGAATTAGGATATTGAATTTTTATGAAAGATGATATGTTTTT

>74LLTRBr_51

TAAGTAAAGTTTGAATGGAGGGTTATTTTTTTGGAGATATTGAGATTTTTGAGATAGTAG

GTAAGGAGTATATTTGTTATTCGATGCGTTTTTACGATCGGTTAGGAAGAACGTAATAAT

TAGAATTTTTTACGGTAAAGTTTTATTGTTTGCATTTTTATGGGGTTAGAGTGTAAGAAG

TAAGAGCGAGAGTAAGAGAGAGAGAAAAACGAAATTTTTTTTATTTTAAAGAGAATAATT

ATTGTTTAGGGCGTATTATTTTTTGATTGGTTGTAGTTTATGGTCGAGTTGACGTTTACG

GGAAAAATAGAGTATAAGTAGTCGTAAATATTTTTGGTTTATGCGTAGATTATTTGTTTA

TTAATTTAGAATATAGGATGTTAGCGTTATTTTGTGACGGCGAATGTGGGGGCGGTTTTT

TATAGTTTTTTTTTTTTTTTTTAATAAGAGTAAATAGGCTATTTATATTAATGAGAGTGG

AGATAGAGGTTAAATTTTTAGTGTGTAGGTAAAGGAGTTATGTATAGGATTAGTTTTTAG

GTTTATAGGTTTTTATTTAGAATAATTTTGATTTGTTTTCGTGTCGTTTTGTTTGGGGGA

AGGGAATTAGGATATTGAATTTTTATGAAAGATGATATGTTTTT

>74LLTRBr_52

TAAGTAAAGTTTGAATGGAGGGTTATTTTTTTGGAGATATTGAGATTTTTGAGATAGTAG

GTAAGGAGTACATTTGTTATTCGATGCGTTCTTACGATCGGTTAGGAAGAACATAATAAT

TAGAATTTTTTACGGTAAAGTTTTATTGTTTACATTTTTATGGGGTTAGAGTGTAAGAAG

TAAGAGCGAGAGTAAGAGAGAGAGAAAAACGAAATTTTTTTTATTTTAAAGAGAATAATT

ATTGTTTAGGGCGTATCACTTTTTGATTGGTTGTAGTTTATGGTCGAGTTGACGTTTACG

GGAAAAATAGAGTATAAGTAGTCGTAAATATTTTTGGTTTATGCGTAGATTATTTGTTTA

TTAATTTAGAATATAGGATGTTAGCGTTATTTTGTGACGGCGAATGTGGGGGCGGTTTTT

TATAGTTTTTTTTTTTTTTTTTTAATAAGAGTAAATAGGTTATTTATATTAATGAGAGTG

GAGATAGAGGTTAAATTTTTAGTGTGTAGGTAAAGGAGTCATGTATAGGATTAGTTTTTA

GGTTTACAGGTTTTTATTTAGAATAATTTTGATTTGTTTTTGTGTCGTTTTGTTTGGGGG

AAGGGAATTAGGATATTGAATTTTTATGAAAGATGATATGTT

>74LLTRBr_53

TAAGTAAAGTTTGAATGGAGGGTTATTTTTTTGGAGATATTGAGATTTTTGAGATAGTAG

GTAAGGAGTACATTTGTTATTCGATGCGTTCTTACGATCGGTTAGGAAGAACATAATAAT

TAGAATTTTTTACGGTAAAGTTTTATTGTTTACATTTTTATGGGGTTAGAGTGTAAGAAG

TAAGAGCGAGAGTAAGAGAGAGAGAAAAACGAAATTTTTTTTATTTTAAAGAGAATAATT

ATTGTTTAGGGCGTATCACTTTTTGATTGGTTGTAGTTTATGGTCGAGTTGACGTTTACG

GGAAAAATAGAGTATAAGTAGTCGTAAATATTTTTGGTTTATGCGTAGATTATTTGCTTA

TTAATTTAGAATATAGGATGTTAGCGTTATTTTGTGACGGCGAATGTGGGGGCGGTTTTT

TATAGTTTTTTTTTTTTTTTTTAATAAGAGTAAATAGGTTATTTATATTAATGAGAGTGG

AGATAGGGGTTAAATTTTTAGTGTGTAGGTAAAGGAGTCATGTATAGGATTAGTTTTTAG

GTTTACAGGTTTTTATTTAGAGTAATTTTGATTTGTTTTTGTGTCGTTTTGTTTGGGGGA

AGGGAATTAGGATATTGAATTTTTATGAAAGATGATATGTTTTT

>74LLTRBr_54

TAAGTAAAGTTTGAATGGAGGGTTATTTTTTTGGAGATATTGAGATTTTTGAGATAGTAG

GTAAGGAGTACATTTGTTATTCGATGCGTTCTTACGATCGGTTAGGAAGAACATAATAAT

TAGAATTTTTTACGGTAAAGTTTTATTGTTTACATTTTTATGGGGTTAGAGTGTAAGAAG

TAAGAGCGAGAGTAAGAGAGAGAGGAAAACGAAATTTTTTTTATTTTAAAGAGAATAATT

ATTGTTTAGGGCGTATCACTTTTTGATTGGTTGTAGTTTATGGTCGAGTTGACGTTTACG

GGAAAAATAGAGTATAAGTAGTCGTAAATATTTTTGGTTTATGCGTAGATTATTTGTTTA

TTAATTTAGAATATAGGATGTTAGCGTTATTTTGTGACGGCGAATGTGGGGGCGGTTTTT

TATAGTTTTTTTTTTTTTTTTTAATAAGAGTAAATAGGTTATTTATATTAATGAGAGTGG

AGATAGAGGTTAAATTTTTAGTGTGTAGGTAAAGGAGTCATGTATAGGATTAGTTTTTAG

GTTTACAGGTTTTTATTTAGAATAATTTTGATTTGTTTTTGTGTCGTTTTGTTTGGGGGA

AGGGAATTAGGATATTGAATTTTTATGAAAGATGATATGTTTTT

>74LLTRBr_55

TAAGTAAAGTTTGAATGGAGGGTTATTTTTTTGGAGATATTGAGATTTTTGAGATAGTAG

GTAAGGAGTACATTTGTCATTCGATGCGTTCTTACGATCGGTTAGGAAGAACATAATAAT

TAGAATTTTTTACGGTAAAGTTTTATTGTTTACATTTTTATGGGGTTAGAGTGTAAGAAG

TAAGAGCGAGAGTAAGAGAGAGAGAAAAACGAAATTTTTTTTATTTTAAAGAGAATAATT

ATTGTTTAGGGCGTATCACTTTTTGATTGGTTGTAGTTTATGGTCGAGTTGACGTTTACG

GGAAAAATAGAGTATAAGTAGTCGTAAATATTTTTGGTTTATGCGTAGATTATTTGTTTA

TTAATTTAGAATATAGGATGTTAGCGTTATTTTGTGACGGCGAATGTGGGGGCGGTTTTT

TATAGTTTTTTTTTTTTTTTTAATAAGAGTAAATAGGTTATTTATATTAATGAGAGTGGA

GATAGAGGTTAAATTTTTAGTGTGTAGGTAAAGGAGTCATGTATAGGATTAGTTTTTAGG

TTTACAGGTTTTTATTTAGAATAATTTTGATTTGTTTTTGTGTCGTTTTGTTTGGGGGAA

GGGAATTAGGATATTGAATTTTTATGAAAGATGATATGTTTTT

>74LLTRBr_56

TAAGTAAAGTTTGAATGGAGGGTTATTTTTTTGGAGATATTGAGATTTTTGAGATAGTAG

GTAAGGAGTATATTTGTTATTCGATGCGTTTTTACGATCGGTTAGGAAGAACATAATAAT

TAGAATTTTTTACGGTAAAGTTTTATTGTTTACATTTTTATGGGGTTAGAGTGTAAGAAG

TAAGAGCGAGAGTAAGAGAGAGAGAAAAACGAAATTTTTTTTATTTTAAAGAGAATAATT

ATTGTTTAGGGCGTATTATTTTTTGATTGGTTGTAGTTTATGGTCGAGTTGACGTTTACG

GGAAAAATAGAGTATAAGTAGTCGTAAATATTTTTGGTTTATGCGTAGATTATTTGTTTA

TTAATTTAGAATATAGGATGTTAGCGTTATTTTGTGACGGCGAATGTGGGGGCGGTTTTT

TATAGTTTTTTTTTTTTTTTTAATAAGAGTAAATAGGCTATTTATATTAATGAGAGTGGA

GATAGAGGTTAAATTTTTAGTGTGTAGGTAAAGGAGTTATGTATAGGATTAGTTTTTAGG

TTTATAGGTTTTTATTTAGAATAATTTTGATTTGTTCTCGTGTCGTTTTGTTTGGGGGAA

GGGAATTAGGATATTGAATTTTTATGAAAGATGATATGTTTTT

>74LLTRBr_57

TAAGTAAAGTTTGAATGGAGGGTTATTTTTTTGGAGATATTGAGATTTTTGAGATAGTAG

GTAAGGAGTATATTTGTTATTCGATGCGTTTTTACGATCGGTTAGGAAGAACATAATAAT

TAGAATTTTTTACGGTAAAGTTTTATTGTTTACATTTTTATGGGGTTAGAGTGTAAGAAG

TAAGAGCGAGAGTAAGAGAGAGAGAAAAACGAAATTTTTTTTATTTTAAAGAGAATAATT

ATTGTTTAGGGCGTATTATTTTTTGATTGGTTGTAGTTTATGGTCGAGTTGACGTTTACG

GGAAAAATAGAGTATAAGTAGTCGTAAATATTTTTGGTTTATGCGTAGATTATTTGTTTA

TTAATTTAGAATATAGGATGTTAGCGTTATTTTGTGACGGCGAATGTGGGGGCGGTTTTT

TATAGTTTTTTTTTTTTTTTTAATAAGAGTAAATAGGCTATTTATATTAATGAGAGTGGA

GATAGAGGTTAAATTTTTAGTGTGTAGGTAAAGGAGTTATGTATAGGATTAGTTTTTAGG

TTTATAGGTTTTTATTTAGAATAATTTTGATTTGTTTTCGTGTCGTTTTGTTTGGGGGAA

GGGAATTAGGATATTGAATTTTTATGAAAGATGATATGTTTTT

>74LLTRBr_58

TAAGTAAAGTTTGAATGGAGGGTTATTTTTTTGGAGATATTGAGATTTTTGAGATAGGAG

GTAAGGAGTATATTTGTTATTCGATGCGTTTTTACGATCGGTTAGGAAGAACATAATAAT

TAGAATTTTTTACGGTAAAGTTTTATTGTTTACATTTTTATGGGGTTAGAGTGTAAGAAG

TAAGAGCGAGAGTAAGAGAGAGAGAAAAACGAAATTTTTTTTATTTTAAAGAGAATAATT

ATTGTTTAGGGCGTATTATTTTTTGATTGGTTGTAGTTTATGGTCGAGTTGACGTTTACG

GGAAAAATAGAGTATAAGTAGTCGTAAATATTTTTGGTTTATGCGTAGATTATTTGTTTA

TTAATTTAGAATATAGGATGTTAGCGTTATTTTGTGACGGCGAATGTGGGGGCGGTTTTT

TATAGTTTTTTTTTTTTTTTTTTAATAAGAGTAAATAGGCTATTTATATTAATGAGAGTG

GAGATAGAGGTTAAATTTTTAGTGTGTAGGTAAAGGAGTTATGTATAGGATTAGTTTTTA

GGTTTATAGGTTTTTATTTAGAATAATTTTGATTTGTTTTCGTGTCGTTTTGTTTGGGGG

AAGGGAATTAGGATATTGAATTTTTATGAAAGATGATATGTTTTT

>74LLTRBr_59

TAAGTAAAGTTTGAATGGAGGGTTATTTTTTTGGAGATATTGAGATTTTTGAGATAGTAG

GTAAGGAGTACATTTGTTATTCGATGCGTTCTTACGATCGGTTAGGAAGAACATAATAAT

TAGAATTTTTTACGGTAAAGTTTTATTGTTTACATTTTTATGGGGTTAGAGTGTAAGAAG

TAAGAGCGAGAGTAAGAGAGAGAGAAAAACGAAATTTTTTTTATTTTAAAGAGAATAATT

ATTGTTTAGGGCGTATCACTTTTTGATTGGTTGTAGTTTATGGTCGAGTTGACGTTTACG

GGAAAAATAGAGTATAAGTAGTCGTAAATATTTTTGGTTTATGCGTAGATTATTTGTTTA

TTAATTTAGAATATAGGATGTTAGCGTTATTTTGTGACGGCGAATGTGGGGGCGGTTTTT

TATAGTTTTTTTTTTTTTTTTAATAAGAGTAAATAGGTTATTTATATTAATGAGAGTGGA

GATAGAGGTTAAATTTTTAGTGTGTAGGTAAAGGAGTCATGTATAGGATTAGTTTTTAGG

TTTACAGGTTTTTATTTAGAATAATTTTGATTTGTTTTTGTGTCGTTTTGTTTGGGGGAA

GGGAATTAGGATATTGAATTTTTATGAAAGATGATATGTTTTT

>74LLTRBr_60

TAAGTAAAGTTTGAATGGAGGGTTATTTTTTTGGAGATATTGAGATTTTTGAGATAGTAG

GTAAGGAGTATATTTGTTATTCGATGCGTTTTTACGATCGGTTAGGAAGAACATAATAAT

TAGAATTTTTTACGGTAAAGTTTTATTGTTTACATTTTTATGGGGTTAGAGTGTAAGAAG

TAAGAGCGAGAGTAAGAGAGAGAGAAAAACGAAATTTTTTTTATTTTAAAGAGAATAATT

ATTGTTTAGGGCGTATTATTTTTTGATTGGTTGTAGTTTATGGTCGAGTTGACGTTTACG

GGAAAAATAGAGTATAAGTAGTCGTAAATATTTTTGGTTTATGCGTAGATTATTTGTTTA

TTAATTTAGAATATAGGATGTTAGCGTTATTTTGTGACGGCGAATGTGGGGGCGGTTTTT

TATAGTTTTTTTTTTTTTTTTAATAAGAGTAAATAGGCTATTTATATTAATGAGAGTGGA

GATAGAGGTTAAATTTTTAGTGTGTAGGTAAAGGAGTTATGTATAGGATTAGTTTTTAGG

TTTATAGGTTTTTATTTAGAATAATTTTGATTTGTTTTCGTGTCGTTTTGTTTGGGGGAA

AGGAATTAGGATATTGAATTTTTATGAAAGATGATATGTTT

>74LLTRBr_61

TAAGTAAAGTTTGAATGGAGGGTTATTTTTTTGGAGATATTGAGATTTTTGAGATAGTAG

GTAAGGAGTATATTTGCTATTCGATGCGTTTTTACGATCGGTTAGGAAGAACATAATAAT

TAGAATTTTTTACGGTAAAGTTTTATTGTTTACATTTTTATGGGGTTAGAGTGTAAGAAG

TAAGAGCGAGAGTAAGAGAGAGAGAAAAACGAAATTTTTTTTATTTTAAAGAGAATAATT

ATTGTTTAGGGCGTATTATTTTTTGATTGGTTGTAGTTTATGGTCGAGTTGACGTTTACG

GGAAAAATAGAGTATAAGTAGTCGTAAATATTTTTGGTTTATGCGTAGATTATTTGTTTA

TTAATTTAGAATATAGGATGTTAGCGTTATTTTGTGACGGCGAATGTGGGGGCGGGTTTT

TATAGTTTTTTTTTTTTTTTTTAATAAGAGTAAATAGGCTATTTATATTAATGAGAGTGG

AGATAGAGGTTAAATTTTTAGTGTGTAGGTAAAGGAGTTATGTATAGGATTAGTTTTTAG

GTTTATAGGTTTTTATTTAGAATAATTTTGATTTGTTTTCGTGTCGTTTTGTTTGGGGGA

AGGGAATTAGGATATTGAATTTTTATGAAAGATGAT

>74LLTRBr_63

TAAGTAAAGTTTGAATGGAGGGTTATTTTTTGGAGATATTGAGATTTTTGAGATAGTAGG

TAAGGAGTACATTTGTTATTCGATGCGTTCTTACGATCGGTTAGGAAGAACATAATAATT

AGAATTTTTTACGGTAAAGTTTTATTGTTTACATTTTTATGGGGTTAGAGTGTAAGAAGT

AAGAGCGAGAGTAAGAGAGAGAGAAAAACGAAATTTTTTTTATTTTAAAGAGAATAATTA

TTGTTTAGGGCGTATCACTTTTTGATTGGTTGTAGTTTATGGTCGAGTTGACGTTTACGG

GAAAAATAGAGTATAAGTAGTCGTAAATATTTTTGGTTTATGCGTAGATTATTTGTTTAT

TAATTTAGAATATAGGATGTTAGCGTTATTTTGTGACGGCGAATGTGGGGGCGGTTTTTT

ATAGTTTTTTTTTTTTTTTTAATAAGAGTAAATAGGTTATTTATATTAATGAGAGTGGAG

ATAGAGGTTAAATTTTTAGTGTGTAGGTAAAGGAGTCATGTATAGGATTAGTTTTTAGGT

TTACAGGTTTTTATTTAGAATAATTTTGATTTGTTTTTGTGTCGTTTTGTTTGGGGGAAG

GGAATTAGGATATTGAATTTTTATGAAAGATGATATGTTTTT

>74LLTRBr_64

TAAGTAAAGTTTGAATGGAGGGTTATTTTTTTGGAGATATTGAGATTTTTGAGATAGTAG

GTAAGGAGTACATTTGTTATTCGATGCGTTCTTACGATCGGTTAGGAAGAACATAATAAT

TAGAATTTTTTACGGTAAAGTTTTATTGTTTACATTTTTATGGGGTTAGAGTGTAAGAAG

TAAGAGCGAGAGTAAGAGAGAGAGAAAAACGAAATTTTTTTTATTTTAAAGAGAATAATT

ATTGTTTAGGGCGTATCACTTTTTGATTGGTTGTAGTTTATGGTCGAGTTGACGTTTACG

GGAAAAATAGAGTATAAGTAGTCGTAAATATTTTTGGTTTATGCGTAGATTATTTGCTTA

TTAATTTAGAATATAGGATGTTAGCGTTATTTTGTGACGGCGAATGTGGGGGCGGTTTTT

TATAGTTTTTTTTTTTTTTTTAATAAGAGTAAATAGGTTATTTATATTAATGAGAGTGGA

GATAGAGGTTAAATTTTTAGTGTGTAGGTAAAGGAGTCATGTATAGGATTAGTTTTTAGG

TTTACAGGTTTTTATTTAGAATAATTTTGATTTGTTTTTGTGTCGTTCTGTTTGGGGGAA

GGGAATTAGGATATTGAATTTTTATGAAAGATGATATGTTTTT

>74LLTRBr_65

TAAGTAAAGTTTGAATGGAGGGTTATTTTTTTGGAGATATTGAGATTTTTGAGATAGTAG

GTAAGGAGTACATTTGTTATTCGATGCGTTCTTACGATCGGTTAGGAAGAACATAATAAT

TAGAATTTTTTACGGTAAAGTTTTATTGTTTACATTTTTATGGGGTTAGAGTGTAAGAAG

TAAGAGCGAGAGTAAGAGAGAGAGAAAAACGAAATTTTTTTTATTTTAAAGAGAATAATT

ATTGTTTAGGGCGTATCACTTTTTGATTGGTTGTAGTTTATGGTCGAGTTGACGTTTACG

GGAAAAATAGAGTATAAGTAGTCGTAAATATTTTTGGTTTATGCGTAGATTATTTGTTTA

TTAATTTAGAATATAGGATGTTAGCGTTATTTTGTGACGGCGAATGTGGGGGCGGTTTTT

TATAGTTTTTTTTTTTTTTTTTTAATAAGAGTAAATAGGTTATTTATATTAATGAGAGTG

GAGATAGAGGTTAAATTTTTAGTGTGTAGGTAAAGGAGTCATGTATAGGATTAGTTTTTA

GGTTTACAGGTTTTTATTTAGAATAATTTTGATTTGTTTTTGTGTCGTTTTGTTTGGGGG

AAGGGAATTAGGATATTGAATTTTTATGAAAGATGATATGTTTTT

>74LLTRBr_66

TAAGTAAAGTTTGAATGGAGGGTTATTTTTTTGGAGATATTGAGATTTTTGAGATAGTAG

GTAAGGAGTATATTTGTTATTCGATGCGTTTTTACGATCGGTTAGGAAGAACATAATAAT

TAGAATTTTTTACGGTAAAGTTTTATTGTTTGCATTTTTATGGGGTTAGAGTGTAAGAAG

TAAGAGCGAGAGTAAGAGAGAGAGAAAAACGAAATTTTTTTTATTTTAAAGAGAATAATT

ATTGTTTAGGGCGTATTATTTTTTGATTGGTTGTAGTTTATGGTCGAGTTGACGTTTACG

GGAAAAATAGAGTATAAGTAGTCGTAAATATTTTTGGTTTATGCGTAGATTATTTGTTTA

TTAATTTAGAATATAGGATGTTAGCGTTATTTTGTGACGGCGAATGTGGGGGCGGTTTTT

TATAGTTTTTTTTTTTTTTTTTAATAAGAGTAAATAGGCTATTTATATTAATGAGAGTGG

AGATAGAGGTTAAATTTTTAGTGTGTAGGTAAAGGAGTTATGTATAGGATTAGTTTTTAG

GTTTATAGGTTTTTATTTAGAATAATTTTGATTTGTTTTCGTGTCGTTTTGTTTGGGGGA

AGGGAATTAGGATATTGAATTTTTATG

>74LLTRBr_67

GAATGGAGGGTTATTTTTTTGGAGATATTGAGATTTTTGAGATAGTAGGTAAGGAGTATA

TTTGTTATTCGATGCGTTTTTACGATCGGTTAGGAAGAACATAATAATTAGAATTTTTTA

CGGTAAAGTTTTATTGTTTACATTTTTATGGGGTTAGAGTGTAAGAAGTAAGAGCGAGAG

TAAGAGAGAGAGAAAAACGAAATTTTTTTTATTTTAAAGAGAATAATTATTGTTTAGGGC

GTATTATTTTTTGATTGGTTGTAGTTTATGGTCGAGTTGACGTTTACGGGAAAAATAGAG

TATAAGTAGTCGTAAATATTTTTGGTTTATGCGTAGATTATTTGTTTATTAATTTAGAAT

ATAGGATGTTAGCGTTATTTTGTGACGGCGAATGTGGGGGCGGTTTTTTATAGTTTTTTT

TTTTTTTTTTAATAAGAGTAAATAGGCTATTTATATTAATGAGAGTGGAGATAGAGGTTA

AATTTTTAGCGTGTAGGTAAAGGAGTTATGTATAGGATTAGTTTTTAGGTTTATAGGTTT

TTATTTAGAATAATTTTGATTTGTTTTCGTGTCGTTTTGTTTGGGGGAAGGGAATTAGGA

TATTGAATTTTTATGAAAGATGATATGTTTTT

>74LLTRBr_68

TAAAGTTTGATGGAGGGTTATTTTTTTGGAGATATTGAGATTTTTGAGATAGTAGGTAAG

GAGTACATTTGTTATTCGATGCGTTCTTACGATCGGTTAGGAAGAACATAATAATTAGAA

TTTTTTACGGTAAAGTTTTATTGTTTACATTTTTATGGGGTTAGAGTGTAAGAAGTAAGA

GCGAGAGTAAGAGAGAGAGAAAAACGAGATTTTTTTTATTTTAAAGAGAATAATTATTGT

TTAGGGCGTATCACTTTTTGATTGGTTGTAGTTTATGGTCGAGTTGACGTTTACGGGAAA

AATAGAGTATAAGTAGTCGTAAATATTTTTGGTTTATGCGTAGATTATTTGTTTATTAAT

TTAGAATATAGGATGTTAGCGTTATTTTGTGACGGCGAATGTGGGGGCGGTTTTTTATAG

TTTTTTTTTTTTTTTTTAATAAGAGTAAATAGGTTATTTATATTAATGAGAGTGGAGATA

GAGGTTAAATTTTTAGTGTGTAGGTAAAGGAGTCATGTATAGGATTAGTTTTTAGGTTTA

CAGGTTTTTATTTAGAATAATTTTGATTTGTTCTTGTGTCGCTTTGTTTGGGGGAAGGGA

ATTAGGATATTGAATTTTTATGAAAGATGATATGTTTTT

>74LLTRBr_69

TAAGTAAAGTTTGAATGGAGGGTTATTTTTTTGGAGATATTGAGATTTTTGAGATAGTAG

GTAAGGAGTATATTTGTTATTCGATGCGTTTTTACGATCGGTTAGGAAGAACATAATAAT

TAGAATTTTTTACGGTAAAGTTTTATTGTTTGCATTTTTATGGGGTTAGAGTGTAAGAAG

TAAGAGCGAGAGTAAGAGAGAGAGAAAAACGAAATTTTTTTTATTTTAAAGAGAATAATT

ATTGTTTAGGGCGTATTATTTTTTGATTGGTTGTAGTTTATGGTCGAGTCGACGTTTACG

GGAAAAATAGAGTATAAGTAGTCGTAAATATTTTTGGTTTATGCGTAGATTATTTGTTTA

TTAATTTAGAATATAGGATGTTAGCATTATTTTGTGACGGCGAATGTGGGGGCGGTTTTT

TATAGTTTTTTTTTTTTTTTAATAAGAGTAAATAGGCTATTTATATTAATGAGAGTGGAG

ATAGAGGTTAAATTTTTAGTGTGTAGGTAAAGGAGTTATGTATAGGATTAGTTTTTAGGT

TTATAGGTTTTTATTTAGAATAATTTTGATTTGTTTTCGTGTCGTTTTGTTTGGGGGAAG

GGAATTAGGATATTGAATTTTTATGAAAGATGATATGTTTTT
