# Supplementary material for: Epigenetic interplay between mouse endogenous retroviruses and host genes
Source: Genome Biol. 2012 Oct 3;13(10):R89. doi: 10.1186/gb-2012-13-10-r89 (PMC3491417; doi:10.1186/gb-2012-13-10-r89)
Supplement: Additional file 4 — All bisulfite sequencing data. Compilation of all bisulfite sequences. [file gb-2012-13-10-r89-S4.zip › IAP4305_gene_ES.rtf]

6/16/10
JM426 & JM425-Hus1 CpG Island B6129 ES Cells Miniprep Sequences
B6 Clones
>HusCpGES_37
GTTTAGAATTTTTGGGTTTTTTTGTTTATTTGTTTTAGTTTATATTATATGTTTATGTTT
TTATTGGTTAGTTTGTTGTATAGGATGAAGTTTAGTTTTTTTGGGTTGATGTGGAGGGTG
TAGGTTTTGGTAAGTTTGGTTATTATGTTATTGATTTTTATAGTGAGGGGGGTAAATAGT
AATTAAGTTTATTGAGGAGTTTATTGGTTTTTTTGTAGAATTTTTTTTTAGTTTATAGAT
TATTTTTGTATTTTAATGTTGTTGATATTTTGTTAGTTTTTTAATAATTTTGTGAGTCTA
TAGTATTAGAATTTAAAATGGAAATAGAATAAAGAAATAGTTTTAGAAGATAATTGATAA
TTGGGGGTTAGGTGGTTTTATATTTTATAGAAGTGTAGGAGTTATTGGGTATTGTAGTTT
ATAATTGTTATAAGGTTAGGTAGATTTTGGGTGGAAGGAGGGGTGTGTGTGGGAGGGTTG
TAAAAGGAAGGTTTTTGGTAGTTGTGGTTTTTTTTAGGGTTGTTTTTATTTTGTTTGTTT
TTTTATAAGGGTTGTATTTTTTGTTTGTTTTTTGTAGTTTTGTTAGTTTATGTGTGAAAT
GATTTAGATAAGTTAGGTTTATGATTTTGGTGTGAAATTTTATGGTGGATATTAGGTTGA
CGGTGGTGTTGGTTTAAGGTGGATAAAAGTTTTTTTTTGAGTGTTTTGGTGGGGAAATTT
GGGTGTGGGTATAGAGGTTTTGTGGGGGTGGTGTGGTTGGAGAGGTTTGTTTATTGTTTG
TTTAGGATAAAGGTTTTTT
>HusCpGES_47
GTTTAGAATTTTTGGGTTTTTTTGTTTATTTGTTTTAGTTTATATTATATGTTCATGTTT
TTATTGGTTAGTTTGTTGTATAGGATGAAGTTTAGTTTTTTTGGGTTGATGTGGAGGGTG
TAGGTTTTGGTAAGTTTGGTTATTATGTTATTGATTTTTATAGTGAGGGGGGTAAATAGT
AATTAAGTTTATTGAGGAGTTTATTGGTTTTTTTGTAGAATTTTTTTTTAGTTTATAGAT
TATTTTTGTATTTTAATGTTGTTGATATTTTGTTAGTTTTTTAATAATTTTGTGAGTTTA
TAGTATTAGAATTTAAAATGGAAATAGAATAAAGAAATAGTTTCAGAAGATAATTGATAA
TTGGGGGTTAGGTGGTTTTATATTTTATAGAAATGTAGGAGTTATTGGGTATTGTAGTTT
ATAATTGTTATAAGGTTAGGTAGATTTTGGGTGGAAGGGGGGGTGTGTGTGGGAGGGTTG
TAAAAGGAAGGTTTTTGGTAGTTGTGGTTTTTTTTAGGGTTGTTTTTATTTTGTTTGTTT
TTTTATAAGGGTTGTATTTTTTGTTTGTTTTTTGTAGTTTTGTTAGTTTATGTGTGAAAT
GATTTAGATAAGTTAGGTTTATGATTTTGGTGTGAAATTTTATGGTGGATATTAGGTTGA
TGGTGGTGTTGGTTTAAGGTGGATAAAAGTTTTTTTTGAGTGTTTTGGTGGGGAAATTTG
GGTGTGGGTATAGAGGTTTTGTGGGGATGGTGTGGTTGGAGAGGTTTGTTTATTGTTTGT
TTAGGATAAAGGTTTTTT
>HusCpGES_90
GTTTAGAATTTTTGGGTTTTTTTGTTTATTTGTTTTAGTTTTACATTATATGTTTATGTT
TTTATTGGTTAGTTTGTTGTATAGGATGAAGTTTAGTTTTTTTGGGTTGATGTGGAGGGT
GTAGGTTTTGGTAAGTTTGGTTATTATGTTATTGATTTTTATAGTGAGGGGGGTAAACAG
TAATTAAGTTTATTGAGGAGTTTATTGGTTTTTTTGTAGAATTTTTTTTTAGTTTATAGA
TTATTTTTGTATTTTAATGTTGTTGATATTTTGTTAGTTTTTTAATAATTTTGTGAGTTT
ATAGTACTAGAATTTAAAATGGAAATAGAATAAAGAAATAGTTTTAGAAGATAATTGATA
ATTGGGGGTTAGGTGGTTTTATATTTTATAGAAATGTAGGAGTTATTGGGTATTGTAGTT
TATAATTGTTATAAGGTTAGGTAGATTTTGGGTGGAAGGAGGGGTGTGTGTGGGAGGGTT
GTAAAAGGAAGGTTTTTGGTAGTTGTGGTTTTTTTTAGGGTTGTTTTTATTTTGTTTGTT
TTTTTATAAGGGTTGTATTTTTTGTTTGTTTTTTGTAGTTTTGTTAGTTCATGTGTGAAA
TGATTTAGATAAGTTAGGTTTATGATTTTGGTGTGAAATTTTATGGTGGATATTAGGTTG
TTGGTGGTGTTGGTTTAAGGTGGATAAAAGTTTTTTTTTGAGTGTTTTGGTGGGGAAATT
TGGGTGTGGGTATAGAGGTTTTGTGGGGATGGTGTGGTTGGAGAGGTTTGTTTATTGTTT
GTTTAGGATAAAGGTTTTTT
>HusCpGES_87
GTTTAGAATTTTTGGGTTTTTTTGTTTATTTGTTTTAGTTTATATTATATGTTTATGTTT
TTATTGGTTAGTTTGTTGTATAGGATGAAGTTTAGTTTTTTTGGGTTGATGTGGAGGGTG
TAGGTTTTGGTAAGTTTGGTTATTATGTTATTGATTTTTATAGTGAGGGGGGTAAATAGT
AATTAAGTTTATTGAGGAGTTTATTGGTTTTTTTGTAGAATTTTTTTTAGTTTATAGATT
ATTTTTGTATTTTAATGTTGTTGATATTTTGTTAGTTTTTTAATAATTTTGTGAGTTCAT
AGTATTAGAATTTAAAATGGAAATAGAATAAAGAAATAGTTTTAGAAGATAATTGATAAT
TGGGGGTTAGGTGGTTTTATATTTTATAGAAATGTAGGAGTTATTGGGTATTGTAGTTTA
TAATTGTTATAAGGTTAGGTAGATTTTGGGTGGAAGGAGGGGTGTGTGTGGGAGGGTTGT
AAAAGGAAGGTTTTTGGTAGTTGTGGTTTTTTTTAGGGTTGTTTTTATTTCGTTTGTTTT
TTTATAAGGGTTGTATTTTTTGTTTGTTTTTTGTAGTTTTGTTAGTTTATGTGTGAAATG
ATTTAGATAAGTTAGGTTTATGATTTTGGTGTGAAATTTTATGGTGGATATTAGGTTGAT
GGTGGTGTTGGTTTAAGGTGGATAAAAGTTTTTTTTTGAGTGTTTTGGTGGGGAAATTTG
GGTGTGGGTATAGAGGTTTTGTGGGGATGGTGTGGTTGGAGAGGTTTGTTTATTGTTTGT
TT
>HusCpGES_90
GTTTAGAATTTTTGGGTTTTTTTGTTTATTTGTTTTAGTTTATATTATATGTTTATGTTT
TTATTGGTTAGTTTGTTGTATAGGATGAAGTTTAGTTTTTTAGGGTTGATGTGGAGGGTG
TAGGTTTTGGTAAGTTTGGTTATTATGTTATTGATTTTTATAGTGAGGGGGGTAAATAGT
AATTAAGTTTATTGAGGAGTTTATTGGTTTTTTTGTAGAATTTTTTTTTAGTTTATAGAT
TATTTTTGTATTTTAATGTTGTTGATATTTTGTTAGTTTTCTAATAATTTTGTGAGTTTA
TAGTATTAGAATTTAAAATGGAAATAGAATAAAGAAATAGTTTTAGAAGATAATTGATAA
TTGGGGGTTAGGTGGTTTTATATTTTATAGAAATGTAGGAGTTATTGGGTATTGTAGTTT
ATAATTGTTATAAGGTTAGGTAGATTTTGGGTGGAAGGAGGGGTGTGTGTGGGAGGGTTG
TAAAAGGAAGGTTTTTGGTAGTTGTGGTTCTTTTTAGGGTTGTTTTTATTTTGTTTGTTT
TTTTATAAGGGTTGTATTTTTTGTTTGTTTTTTGTAGTTTTGTTAGTTTATGTGTGAAAT
GATTTAGATAAGTTAGGTTTATGATTTTGGTGTGAAATTTTATGGTGGATATCAGGTTGA
TGGTGGTGTTGGTTTAAGGTGGATAAAAGTTTTTTTTGAGTGTTTTGGTGGGGAAATTTG
GGTGTGGGTATAGAGGTTTTGTGGGGATGGTGTGATTGGAGAGGTTTGTTTATTGTTTGT
TTAGGATAAAGGTTTTTT
>HusCpGES_91
GTTTAGAATTTTTGGGTTTTTTTGTTTATTTGTTTTAGTTTATATTATATGTTTATGTTT
TTATTGGTTAGTTTGTTGTATAGGATGAAGTTTAGTTTTTTTGGGTTGATGTGGAGGGTG
TAGGTTTTGGTAAGTTTGGTTATTATGTTATTGATTTTTATAGTGAGGGGGGTAAATAGT
AATTAAGTTTATTGAGGAGTTTATTGGTTTTTTTGTAGAATTTTTTTTTAGTTTATAGAT
TATTTTTGTATTTTAATGTTGTTGATATTTTGTTAGTTTTTTAATAATTTTGTGAGTTTA
TAGTATCAGAATTTAAAATGGAAATAGAATAAAGAAATAGTTTTAGAAGATAATTGATAA
TTGGGGGTTAGGTGGTTTCATATTTTATAGAAACGTAGGAGTTATTGGGTATTGTAGTTT
ATAATTGTTATAAGGTTAGGTAGATTTTGGGTGGAAGGAGGGGTGTGTGTGGGAGGGTTG
TAAAAGGAAGGTTTTTGGTAGTTGTGGTTTTTTTTAGGGTTGTTTTTATTTTGTTTGTTT
TTTTATAAGGGTTGTATTTTTTGTTTGTTTTTTGTGGTTTTGTTAGTTTATGTGTGAAAT
GATTTAGATAAGTTAGGTTTATGATTTTGGTGTGAAATTTTATGGTGGATATTAGGTTGA
TGGTGGTGTTGGTTTAAGGTGGATAAAAGTTTTTTTTTGAGTGTTTTGGTGGGGAAATTT
GGGTGTGGGTATAGAGGTTTTGTGGGGATGGTGTGGTTGGAGAGGTTTGTTTATTGTTTG
TTTAGGATAAAGGTTTTTT
>HusCpGES_94
GTTTAGAATTTTTGGGTTTTTTTGTTTATTTGTTTTAGTTTATATTATATGTTTATGTTT
TTATTGGTTAGTTTGTTGTATAGGATGAAGTTTAGTTTTTTTGGGTTGATGTGGAGGGTG
TAGGTTTTGGTAAGTTTGGTTATTATGTTATTGATTTTTATAGTGAGGGGGGTAAATAGT
AATTAAGTTTATTGAGGAGTTTATTGGTTTTTTTGTAGAATTTTTTTTTAGTTTACAGAT
TATTTTTGTATTTTAATGTTGTTGATATTTTGTTAGTTTTTTAATAATTTTGTGAGTTTA
TAGTATTAGAATTTAAAATGGAAATAGAATAAAGAAATAGTTTTAGAAGATAATTGATAA
TTGGGGGTTAGGTGGTTTCATATTTTATAGAAATGTAGGAGTTATTGGGTATTGTAGTTT
ATAATTGTTATAAGGTTAGGTAGATTTTGGGTGGAAGGAGGGGTGTGTGTGGGAGGGTTG
TAAAAGGAAGGTTTTTGGTAGTTGTGGTTTTTTTTAGGGTTGTTTTTATTTTGTTTGTTT
TTTTATAAGGGTTGTATTTTTTGTTTGTTTTTTGTAGTTTTGTTAGTTTATGTGTGAAAT
GATTTAGATAAGTTAGGTTTATGATTTTGGTGTGAAATTTTATGGTGGATATTAGGTTGA
TGGTGGTGTTGGTTTAAGGTGGATAAAAGTTTTTTTTTGGGTGTTTTGGTGGGGAAATTT
GGGTGTGGGCATAGAGGTTTTGTGGGGATGGTGTGGTTGGAGAGGTTTGTTTATTGTTTG
TTTAGGATAAAGGTTTTTT
>HusCpGES_96
GTTTAGAATTTTTGGGTTTTTTTGTTTATTTGTATTAGTTTATATTATATGTTTATGTTT
TTATTGGTTAGTTTGTTGTATAGGATGAAGTTTAGTTTTTTTGGGTTGATGCGGAGGGTG
TAGGTTTTGGTAAGTTTGGTTATTATGTTATTGATTTTTATAGTGAGGGGGGTAAATAGT
AATTAAGTTTATTGAGGAGTTTATTGGTTTTTTTGTAGAATTTTTTTTTAGTTTATAGAT
TATTTTTGTATTTTAATGTTGTTGATATTTTGTTAGTTTTTTAATAATTTTGTGAGTTTA
TAGTATTAGAATTTAAAATGGAAATAGAATAAAGAAATAGTTTTAGAAGATAATTGATAA
TTGGGGGTTAGGTGGTTTTATATTTTATAGAAATGTAGGAGTTATTGGGTATTGTAGTTT
ATAATTGTTATAAGGTTAGGTAGATTTTGGGTGGAAGGAGGGGTGTGTGTGGGAGGGTTG
TAAAAGGAAGGTTTTTGGTAGTTGTGGTTTTTTTTAGGGTTGTTTTTATTTTGTTTGTTT
TTTTATAAGGGTTGTATTTTTTGTTTGTTTTTTGTAGTTTTGTTAGTTTATGTGTGAAAT
GATTTAGATAAGTTAGGTTTATGATTTTGGTGTGAAATTTTATGGTGGATATTAGGTTGA
TGGTGGTGTTGGTTTAAGGTGGATAAAAGTTTTTTTTTGAGTGTCTTGGTGGGGAAATTT
GGGTGTGGGTATAGAGGTTTTGTGGGGATGGTGTGGTTGGAGAGGTTTGTTTATTGTTTG
TTTAGGA
>HCpGMP3ES_SP6
GTTTAGAATTTTTGGGTTTTTTTGTTTATTTGTTTTAGTTTATATTATATGTTTATGTTT
TTATTGGTTAGTTTGTTGTATAGGATGAAGTTTAGTTTTTTTGGGTTGATGTGGAGGGTG
TAGGTTTTGGTAAGTTTGGTTATTATGTTATTGATTTTTATAGTGAGGGGGGTAAATAGT
AATTAAGTTTATTGAGGGGTTTATTGGTTTTTTTGTAGAATTTTTTTTTAGTTTATAGAT
TGTTTTTGTATTTTAATGTTGTTGATATTTTGTTAGTTTTTTAATAATTTTGTGAGTTTA
TAGTATTAGAATTTAAAATGGAAATAGAATAAAGAAATAGTTTTAGAAGATAATTGATAA
TTGGGGGTTAGGTGGTTTTATATTTTATAGAAATGTAGGAGTTATTGGGTATTGTAGTTT
ATAATTGTTATAAGGTTAGGTAGATTTTGGGTGGAAGGAGGGGTGTGTGTGGGAGGGTTG
TAAAAGGAAGGTTTTTGGTAGTTGTGGTTTTTTTTAGGGTTGTTTTTATTTTGTTTGTTT
TTTTATAAGGGTTGTATTTTTTGTTTGTTTTTTGTAGTTTTGTTAGTTTATGTGTGAAAT
GATTTAGATAAGTTAGGTTTATGATTTTGGTGTGGAATTTTATGGTGGATATTAGGTTGA
TGGTGGTGTTGGTTTAAGGTGGATAAAAGTTTTTCTTTGAGTGTTTTGGTGGGGAAATTT
GGGTGTGGGTATAGAGGTTTTGTGGGGATGGTGTGGTTGGAGAGGTTTGTTTATTGTTTG
TTTAGGATAAAGGTTTTTT
>HCpGMP6ES_SP6
GTTTAGAATTTTTGGGTTTTTTTGTTTATTTGTTTTAGTTTATATTATACGTTTATGTTT
TTATTGGTTAGTTTGTCGTATAGGATGAAGTTTAGTTTTTTTGGGTTGATGTGGAGGGTG
TAGGTTTTGGTAAGTTTGGTTATTATGTTATTGATTTTTATAGTGAGGGGGGTAAATAGT
AATTAAGTTTATTGAGGAGTTTATTGGTTTTTTTGTAGAATTTTTTTTTAGTTCCTTAGA
TTATTTTTGTATTTTAATGTTGCTGATATTTTGTTAGTTTTTTAATAATTTTGTGAGTTT
ATAGTATTAGAATTTAAAATGGAAATAGAATAAAGAAATAGTTTTAGAAGATAATTGATA
ATTGGGGGTTAGGTGGTTTTCCCCTTTTATAGAAATGTAGGAGTTATTGGGTATTGTAGT
TTATAATTGTTATAAGGTTAGGTAGATTTTGGGTGGAAGGAGGGGTGTGTGTGGGAGGGT
TGTAAAAGGAAGGTTTTTGGTAGTTGTGGTTTTTTTTAGGGTTGTTTTTATTTTGTTTGT
TTTTTTATAAGGGTTGTATTTNTTGNTTGTTTTTTGTAGTTTTGTTAGTTTATGTGTGAA
ATGATTTAGATAAGTTAGGTTTATGATTTTGGTGTGAAATTTTATGGTGGATATTAGGTT
GATGGTGGTGTTGGTTTAAGGTGGATAAAAGTTTTTTTTTGAGTGTTTTGGTGGGGAAAT
TTGGGTGTGGGTATAGAGGTTTTGTGGGGATGGTGTGGTTGGAGAGGTTTGTTTATTGTT
TGTTTAGGAT
>HCpGMP7ES_SP6
GTTTAGAATTTTTGGGTTTTTTTGTTTATTTGTTTTAGTTTATATTATATGTTTTACGTT
TTTATTGGTTAGTTTGTTGTATAGGACGAAGTTTAGTTTTTTTGGGTTGATGTGGAGGGT
GTAGGTTTCGGTAAGTTCGGTTATTANGTTATCGATTTTTATAGCGAGGGGGGTAAATAG
TAATTAAGTTTATCGAGGAGNTTATCGGTTTTTTTGTAGAATTTTTTTTTAGTTTATAGA
TTATTTTTGTATTTTAATGTCGTTGATATTTTGTTAGTTTTTTAATAATTTTGCGAGTTT
ATAGTATTAGAATTTAAAATGGAAATAGAATAAAGAAACAGTTTTAGAAGATAATTGATA
ATTGGGGGTTAGGCGGTTTTATATTTTATAGAAACGTAGGAGTTATTGGGTATCGTAGTT
TATAATCGTTATAAGGTTAGGTAGATTTTGGGTGGAAGGAGGGGTGTGCGTGGGAGGGTC
GTAAAAGGAGGGTTTTTGGTAGTTGTGGTTTTTTTTAGGGTTGTTTTTATTTCGTTTGTT
TTTTTATAAGGGTCGTATTTTCTGTTTGTTTTTCGTAGTTTTGTTAGTTTATGTGCGAAA
CGATTTAGATAAGTTAGGTTTATGATTTTGGTGCGAAATTTTATGGCGGATATTAGGTTG
ATGGTGGTGTCGGTTTAAGGTGGATAAAAGTTTTTTTTTGAGTGTTTCGGTGGGGAAATT
TGGGCGCGGGTATAGAGGTTTCGTGGGGATGGTGTGGTTGGAGAGGTTTGTTTACTTGTT
TGTTTAGGAT


>HCpGMP9ES_SP6
GTTTAGAATTTTTGGGTTTTTTTGTTTATTTGTTTTAGTTTATATTATATGTTTATGTTT
TTATTGGTTAGTTTGTTGTATAGGATGAAGTTTAGTTTTTTTGGGTTGATGTGGAGGGTG
CAGGTTTTGGTAAGTTTGGTTATTATGTTATTGATTTTTATAGTGAGGGGGGTAAATAGT
AATTAAGTTTATTGAGGAGTTTATTGGTTTTTTTGTAGAATTTTTTTTTAGTTTATAGAT
TATTTTTGTATTTTAATGTTGTTGATATTTTGTTAGTTTTTTAATAATTTTGTGAGTTTA
TAGTATTAGAATTTAAAATGGAAATAGAATAAAGAAATAGTTTTAGAAGATAATTGATAA
TTGGGGGTTAGGTGGTTTTATATTTTATAGAAATGTAGGAGTTATTGGGTATTGTAGTTT
ATAATTGTTATAAGGTTAGGTAGATTTTGGGTGGAAGGAGGGGTGTGTGTGGGAGGGTTG
TAAAAGGAAGGTTTTTGGTAGTTGTGGTTTTTTTTAGGGTTGTTTTTATTTTGTTTGTTT
TTTTATAAGGGTTGTATTTTTTGTTTGTTTTTTGTAGTTTTGTTAGTTTATGTGTGAAAT
GATTTAGATAAGTTAGGTTTATGATTTTGGTGTGAAATTTTATGGTGGATATTAGGTTGA
TGGTGGTGTTGGTTTAAGGTGGATAAAAGTTTTTTTTTGAGTGTTTTGGTGGGGAAATTT
GGGTGTGGGTATAGAGGTTTTGTGGGGATGGTGTGGTCGGAGAGGTTTGTTTATTGTTTG
TTTAGGATAAAGGTTTTTTT


129 Clones
>HusCpGES_42
GTTTAGAATTTTTGGGTTTTTTTGTTTATTTGTTTTAGTTTATATTATATGTTTATGTTT
TTATTGGTTAGTTTGTTGTAAAGGATGAAGTTTAGTTTTTTTGGGTTGATGTGGAGGGTG
TAGGTTTTGGTAAGTTTGGTTATTATGTTATTGATTTTTATAGTGAGGGGGGTAAATAGT
AATTAAGTTTATTGAGGAGTTTATTGGTTTTTTTGTAGAATTTTTTTTTAGTTTACAGAT
TATTTTTGTATTTTAATGTTGTTGATATTTTGTTAGTTTTTTAATAATTTTGTGAGTTTA
TAGTATTAGAATTTAAAATGGAAATAGAATAAAGAAATAGTTTTAGAAGATAATTGATAA
TTGGGGGTTAGGTGGTTTCATATTTTATAGAAATGTAGGAGTTATTGGGTATTGTAGTTT
ATAATTGTTATAAGGTTAGGTAGATTTTGGGTGGAAGGAGGGGTGTGTGTGGGAGGGTTG
TAAAAGGAAGGTTTTTGGTAGTTGTGGTTTTTTTTAGGGTTGTTTTTATTTTGTTTGTTT
TTTTATAAGGGTTGTATTTTTTGTTTGTTTTTTGTAGTTTTGTTAGTTTATGTGTGAAAT
GATTTAGATAAGTTAGGTTTATGATTTTGGTGTGAAATTTTATGGTGGATATTAGGTTGA
TGGTGGTGTTGGTTTAAGGTGGATAAAAGTTTTTTTTTGAGTGTTTTGGTGGGGAAATTT
GGGTGTGGGCATAGAGGTTTTGTGGGGATGGTGTGGTTGGAGAGGTTTGTTTATTGTTTG
TTTAGGATAAAGGTTTTTT

>HusCpGES_46
GTTTAGAATTTTTGGGTTTTTTTGTTTATTTGTTTTAGTTTATATTATATGTTTATGTTT
TTATTGGTTAGTTTGTTGTAAAGGATGAAGTTTAGTTTTTTAGGGTTGATGTGGAGGGTG
TAGGTTTTGGTAAGTTTGGTTATTATGTTATTGATTTTTATAGTGAGGGGGGTAAATAGT
AATTAAGTTTATTGAGGAGTTTATTGGTTTTTTTGTAGAATTTTTTTTTAGTTTATAGAT
TATTTTTGTATTTTAATGTTGTTGATATTTTGCTAGTTTTTTAATAATTTTGTGAGTTTA
TAGTATTAGAATTTAAAATGGAAATAGGATAAAGAAATAGTTTTAGAAGATAATTGATAA
TTGGGGGTTAGGTGGTTTTATATTTTATAGAAATGTAGGAGTTATTGGGTATTGTAGTTT
ATAATTGTTATAAGGTTAGGTAGATTCTGGGTGGAAGGAGGGGTGTGTGTGGGAGGGTTG
TAAAAGGAAGGTTTTTGGTAGTTGTGGTTTTTTTTAGGGTTGTTTTTATTTCGTTTGTTT
TTTATAAGGGTTGCATTTTTTGTTTGTTTTTTGTAGTTTTGTTAGTTTGTGTGTGAAATG
ATTTAGATAAGTTAGGTTTATGATTTTGGTGTGAAATTTTATGGTGGATATTAGGTTGAT
GGTGGTGTTGGTTTAAGGTGGATAAAAGTTTTTTTTGAGTGTTTTGGTGGGGAAATTTGG
GTGTGGGTATAGAGGTTTTGTGGGGATGGTGTGGTTGGAGAGGTTTGTTTATTGTTTGTT
TAGGATAAAGGTTTTTT
>HusCpGES_85
GTTTAGAATTTTTGGGTTTTTTTGTTTATTTGTTTTAGTTTATATTATATGTTTATGTTT
TTATTGGTTAGTTTGTTGTAAAGGATGAAGTTTAGTTTTTTAGGGTTGATGTGGAGGGTG
TAGGTTTTGGTAAGTTTGGTTATTATGTTATTGATTTTTATAGTGAGGGGGGTAAATAGT
AATTAAGTTTATTGAGGAGTTTATTGGTTTTTTTGTAGAATTTTTTTTTAGTTTATAGAT
TATTTTTGTATTTTAATGTTGTTGATATTTTGTTAGTTTTTTAATAATTTTGTGAGTTTA
TAGTATTAGAATTTAAAATGGAAATAGAATAAAGAAATAGTTTTAGAAGATAATTGATAA
TTGGGGGTTAGGTGGTTTTATATTTTATAGAAATGTAGGAGTTATTGGGTATTGTAGTTT
ATAATTGTTATAAGGTTAGGTAGATTTTGGGTGGAAGGAGGGGTGTGTGTGGGAGGGTTG
TAAAAGGAAGGTTTTTGGTAGTTGTGGTTTTTTTTAGGGTTGTTTTTATTTTGTTTGTTT
TTTTATAAGGGTTGTATTTTTTGTTTGTTTTTTGTAGTTTTGTTAGTTTATGTGTGAAAT
GATTTAGATAAGTTAGGTTTATGATTTTGGTGTGAAATTTTATGGTGGATATTAGGTTGA
TGGTGGTGTTGGTTTAAGGTGGATAAAAGTTTTTTTTTGAGTGTTTTGGTGGGGAAATTT
GGGTGTGGGTATAGAGGTTTTGTGGGGATGGTGTGGCTGGAGAGGTTTGTTTATTGTTTG
TTTAGGATAAAGGTTTTTT
>HusCpGES_86
GTTTAGAATTTTTGGGTTTTTTTGTTTATTTGTTTTAGTTTATATTATATGTTTATGTTT
TTATTGGTTAGTTTGTTGTAAAGGATGAAGTTTAGTTTTTTAGGGTTGATGTGGAGGGTG
TAGGTTTTGGTAAGTTTGGTTATTATGTTATTGATTTTTATAGTGAGGGGGGTAAATAGT
AATTAAGTTTATTGAGGAGTTTATTGGTTTTTTTGTAGAATTTTTTTTTAGTTTATAGAT
TATTTTTGTATTTTAATGTTGTTGATATTTTGTTAGTTTTTTAATAATTTTGCGAGTTTA
TAGTATTAGAATTTAAAATGGAAATAGAATAAAGAAATAGTTTTAGAAGATAATTGATAA
TTGGGGGTTAGGTGGTTTTATATTTTATAGAAATGTAGGAGTTATTGGGTATTGTAGTTT
ATAATTGTTATAAGGTTAGGTAGATTTTGGGTGGAAGGAGGGGTGTGTGTGGGAGGGTTG
TAAAAGGAAGGTTTTTGGTAGTTGTGGTTTTTTTTAGGGTTGTTTTTATTTTGTTTGTTT
TTTTATAAGGGTTGTATTTTTTGTTTGGTTTTTGTAGTTTTGTTAGTTTATGTGTGAAAT
GATTTAGATAAGTTAGGTTTATGATTTTGGTGTGAAATTTTATGGTGGATATTAGGTTGA
TGGTGGTGTTGGTTTAAGGTGGATAAAAGTTTTTTTTTGAGTGTTTTGGTGGGGAAATTT
GGGTGTGGGTATAGAGGTTTTGTGGGGATGGTGTGGTTGGAGAGGTTTGTTTATTGTTTG
TTTAGGATAAAGGTTTTTT
>HusCpGES_89
GTTTAGAATTTTTGGGTTTTTTGTTTATTTGTTTTAGTTTATATTATATGTTTATGTTTT
TATTGGTTAGTTTGTTGTAAAGGATGAAGTTTAGTTTTTTAGGGTTGATGTGGAGGGTGT
AGGTTTTGGTAAGTTTGGTTATTATGTTATTGATTTTTATAGTGAGGGGGGTAAATAGTA
ATTAAGTTTATTGAGGAGTTTATTGGTTTTTTTGTAGAATTTTTTTTTAGTTTATAGATT
ATTTTTGTATTTTAATGTTGTTGATATTTTGTTAGTTTCTTAATAATTTTGTGAGTTTAT
AGTATTAGAATTTAAAATGGAAATAGAATAAAGAAATAGTTTTAGAAGATAATTGATAAT
TGGGGGTTAGGCGGTTTTATATTTTATAGAAATGTAGGAGTTATTGGGTATTGTAGTTTA
TAATTGTTATAAGGTTAGGTAGATTTTGGGTGGAAGGAGGGGTGTGTGTGGGAGGGTTGT
AAAAGGAAGGTTTTTGGTAGTTGTGGTTTTTTTTAGGGTTGTTTTTATTTTGTTTGTTTT
TTTATAAGGGTTGTATTTTTTGTTTGTTTTTTGTAGTTTTGTTAGTTTATGTGTGAAATG
ATTTAGATAAGTTAGGCTTATGATTTTGGTGTGAAATTTTATGGTGGATATTAGGTTGAT
GGTGGTGTTGGTTTAAGGTGGATAAAAGTTTTTTTGAGTGTTTTGGTGGGGAAATTTGGG
TGTGGGTATAGAGGTTTTGTGGGGATGGTGTGGTTGGAGAGGTTTGTTTATTGTTTGTTT
AGGATAAAGGTTTTTT
>HusCpGES_92
GTTTAGAATTTTTGGGTTTTTTTGTTTATTTGTTTTAGTTTATATTATATGTTTATGTTT
TTATTGGTTAGTTTGTTGTAAAGGATGAAGTTTAGTTTTTTAGGGTTGATGTGGAGGGTG
TAGGTTTTGGTAAGTTTGGTTATTATGTTATTGATTTTTATAGTGAGGGGGGTAAATAGT
AATTAAGTTTATTGAGGAGTTTATTGGTTTTTTTGTAGAATTTTTTTTTAGTTTATAGAT
TATTTTTGTATTTTAATGTTGTTGATATTTTGTTAGTTTCTTAATAATTTTGTGAGTTTA
TGGTATTAGAATTTAAAATGGAAATAGAATAAAGAAATAGTTTTAGAAGATAATTGATAA
TTGGGGGTTAGGTGGTTTTATATTTTATAGAAATGTAGGAGTTATTGGGTATTGTAGTTT
ATAATTGTTATAAGGTTAGGTAGATTTTGGGTGGAAGGAGGGGTGTGTGTGGGAGGGTTG
TAAAAGGAAGGTTTTTGGTAGTTGTGGTTTTTTTTAGGGTTGCTTTTATTTTGTTTGTTT
TTTTATAAGGGTTGTATTTTTTGTTTGTTTTTTGTAGTTTTGTTAGTTTATGTGTGAAAT
GATTTAGATAAGTTAGGTTTATGATTTTGGTGTGAAATTTTATGGTGGATATTAGGTTGA
TGGTGGTGTTGGTTTAAGGTGGATAAAAGTTTTTTTTTGAGTGTTTTGGTGGGGAAATTT
GGGTGTGGGTACAGAGGTTTTGTGGGGATGGTGTGGTTGGAGAGGTTTGTTTATTGTTTG
TTTAGGATAAAGGTTTTTT

>HusCpGES_95
GTTTAGAATTTTTGGGTTTTTTTGTTTATTTGTTTTAGTTTATATTATATGTTTATGTTT
TTATTGGTTAGTTTGTTGTAAAGGATGAAGTTTAGTTTTTTAGGGTTGATGTGGAGGGTG
TAGGTTTTGGTAAGTTTGGTTATTATGTTATTGATTTTTATAGTGAGGGGGGTAAATAGT
AATTAAGTTTATTGAGGAGTTTATTGGTTTTTTTGTAGAATTTTTTTTTAGTTTACAGAT
TATTTTTGTATTTTAATGTTGTTGGTATTTTGTTAGTTTTTTAATAATTTTGTGAGTTTA
TAGTATTAGAATTTAAAATGGAAATAGAATAAAGAAATAGTTTTAGAAGATAATTGATAA
TTGGGGGTTAGGTGGTTTTATATTTTATAGAAATGTAGGAGTTATTGGGTATTGTAGTTT
ATAATTGTTATAAGGTTAGGTAGATTTTGGGTGGAAGGAGGGGTGTGTGTGGGAGGGTTG
TAAAAGGAAGGTTTTTGGTAGTTGTGGTTTTTTTTAGGGTTGTTTTTATTTTGTTTGTTT
TTTTATAAGGGTTGTATTTTTTGTTTGTTTTTTGTAGTTCTGTTAGTTTATGTGTGAAAT
GATTTAGATAAGTTAGGTTTATGATTTTGGTGTGAAATTTTATGGTGGATATTAGGTTGA
TGGTGGTGTTGGTTTAAGGTGGATAAAAGTTTTTTTTTGAGTGTTTTGGTGGGGAAATTT
GGGTGTGGGTATAGAGGTTTTGTGGGGATGGTGTGGTTGGAGAGGTTTGTTTATTGTTTG
TTTAGATAAAGGTTTTTT
