# Supplementary material for: Epigenetic interplay between mouse endogenous retroviruses and host genes
Source: Genome Biol. 2012 Oct 3;13(10):R89. doi: 10.1186/gb-2012-13-10-r89 (PMC3491417; doi:10.1186/gb-2012-13-10-r89)
Supplement: Additional file 4 — All bisulfite sequencing data. Compilation of all bisulfite sequences. [file gb-2012-13-10-r89-S4.zip › IAP4305_interveningregion_ES.rtf]

usHukkkkkHus1 Intervening Region
B6129 ES Cells
B6 Clones
>HLTRMP9ES_SP6
GGTATTGGGATTAATATTTTTTTTAGTTTTTTTATTTTTTAAGTAAGTATTTTTTCGATG
TATTATATATTCAAACAATAAAATATTTTTAAAATTTTTTGTTAAATATGTAAAAGTATA
TTTTTGTAATTTATAAGTCGGTTTCGTAGGTGAAAGTATTTATTATAAAGGTTTGAGTTT
GGGTTTTGGAATATTAGGTATATTTTTGAAAGTTGTTTTTTGATTTTTGTATGTGTATTA
TGGAATGAGTATGTTTATTTTTATATAAAAATAGTAAATAAAGAAGTTTTAAAAATTTGT
TTTTAATTTTGATATATAGAATGAATATATTAATAAAATAGTTGATAGTGTAGATTATTG
GTAAAGTGAAAAATAGAAAAATATATATAATATAAAATTATATTTTAGTTTATATTTTAA
CGGATAAGTGATTGGTAAGTTAAATATTATTAATTTTGAAATATTAAATGTGATATGAAG
TAAAGTAATTGTTATATGTATTGTGTGGTTAGAGGTTGTGTTGTTTGTGTGTGTATGTAT
GGATATAAAGTTATTTACGTTTCGGAATTTATGGGAAGGTTAAAGGGGAGTTTTTGGTTT
TAGTATTTAATTTTTATTAAGTTTGAGGTAGGTTTTTTTTTTGTTGTTAGTTGATGAATA
GTTTAGGTTAGTTGGAATATAGTTTTTGGGG
>HLTRMP10ES_SP6
GGTATTGGGATTAATATTTTTTTTAGTTTTTTTATTTTTTAAGTAAGTATTTTTTCGATG
TATTATATATTTAAATAATAAAATATTTTTAAAATTTTTTGTTAAATATGTAAAAGTATA
TTTTTGTAATTTATAAGTCGGTTTCGTAGGTGAAAGTATTTATTATAAAGGTTTGAGTTT
GGGTTTTGGAATATTAGGTATATTTTTGAAAGTTGTTTTTTGATTTTTGTATGTGTATTA
TGGAATGAGTATGTTTATTTTTATATAAAAATAGTAAATAAAGAAGTTTTAAAAATTTGT
TTTTAATTTTGATATATAGAATGAATATATTAATAAAATAGTTGGTAGTGTAGATTATTG
GTAAAGTGAAAAATAGAAAAATATATATAATATAAAATTATATTTTAGTTTATATTTTAA
CGGATAAGTGGTTGGTAAGTTAAATATTATTAATTTTGAAATATTAAATGTGATATGAAG
TAAAGTAATTGTTATATGTATTGTGTGGTTAGAGGTTGTGTTGTTTGTGTGTGTATGTAT
GGATATAAAGCTATTTACGTTTCGGAATTTATGGGAAGGTTAAAGGGGAGTTTTTGGTTT
TAGTATTTAATTTTTATTAAGTTTGAGGTAGGTTTTTTTTTTGTTGTTAGTTGATGAATA
GTTTAGGTTAGTTGGAATATAGTTTTTGGGG
>HLTRMP12ES_SP6
GGTATTGGGATTAATATTTTTTTTAGTTTTTTTATTTTTTAAGTAAGTATTTTTTCGATG
TATTATATATTTAAATAATAAAATATTTTTAAAATTTTTTGTTAAATATGTAAAAGTATA
TTTTTGTAATTTATAAGTCGGTTTCGTAGGTGAAAGTATTTATTATAAAGGTTTGAGTTT
GGGCTTTGGAATATTAGGTATATTTTTGAAAGTTGTTTTTTGATTTTTGTATGTGTATTA
TGGAATGAGTATGTTTATTTTTATATAAAAATAGTAAATAAAGAAGTTTTAAAAATTTGT
TTTTAATTTTGATATATAGAATGAATATATTAATAAAATAGTTGATAGTGTAGATTATTG
GTAAAGTGAAAAATAGAAAAATATATATAATACAAAATTATATTTTAGTTTATATTTTAA
CGGATAAGTGGTTGGTAAGTTAAATATTATTAATTTTGAAATATTAAATGTGATATGAAG
TAAAGTAATTGTTATATGTATTGTGTGGTTAGAGGTTGTGTTGTTTGTGTGTGTATGTAT
GGATATAAAGTTATTTACGTTTCGGAATTTATGGGAAGGTTAAAGGGGAGTTTTTGGTTT
TAGTATTTAATTTTTATTAAGTTTGAGGTAGGTTTTTTTTTGTTGTTAGTTGATGAATAG
TTTAGGTTAGTTGGAATATAGTTTTTGGGG
>HLTRMP4ES_SP6
GGTATTGGGATTAATATTTTTTTTAGTTTTTTTATTTTTTAAGTAAGTATTTTTTCGATG
TATTATATATTTAAATAATAAAATATTTTTAAAATTTTTTGTTAAATATGTAAAAGTATA
TTTTTGTAATTTATAAGTCGGTTTCGTAGGTGAAAGTATTTATTATAAAGGTTTGAGTTT
GGGTTTTGGAATATTAGGTATATTTTTGAAAGTTGTTTTTTGATTTTTGTATGTGTATTA
TGGAATGAGTATGTTTATTTTTATATAAAAATAGTAAATAAAGAAGTTTTAAAAATTTGT
TTTTAATTTTGATATATAGAATGAATATATTAATAAAATAGTTGATAGTGTAGATTATTG
GTAAAGTGAAAAATAGAAAAATATATATAATATAAAATTATATTTTAGTTTATATTTTAA
CGGATAAGTGGTTGGTAAGTTAAATATTATTAATTTTGAAATATTAAATGTGATATGAAG
TAAAGTAATTGTTATATGTATTGTGTGGTTAGAGGTTGTGTTGTTTGTGTGTGTATGTAT
GGATATAAAGTTATTTACGTTTCGGAATTTATGGGAAGGTTAAAGGGGAGTTTTTGGTTT
TAGTAATTAATTTTCATTAAGTTTGAGGTAGGTTTTTTTTTGTTGTTAGTTGATGAATAG
TTTAGGTTAGTTGGAATATAGTTTTTGGGG
>HLTRMP7ES_SP6
GGTATTGGGATTAATATTTTTTTTAGTTTTTTTATTTTTTAAGTAAGTATTTTTTCGATG
TATTATATATTTAAATAATAAAATATTTTTAAAATCTTTTGTTAAATATGTAAAAGTATA
TTTTTGTAATTCATAAGTCGGTTTCGTAGGTGAAAGTATTTATTATAAAGGTTTGAGTTT
GGGTTTTGAAATATTAGGTATATTTTTGAAAGTTGTTTTTTGATTTTTGTATGTGTATTA
TGGAATGAGTATGTTTATTTTTATATAAAAATAGTAAATAAAGAAGTTTTAAAAACTTGT
TTTTAATTTTGATATATAGAATGAATATATTAATAAAATAGTTGATAGTGTAGATTATTG
GTAAAGTGAAAAATAGAAAAATATATATAATATAAAATTATATTTTAGTTTATATTTTAA
CGGATAAGTGGTTGGTAAGTTAAATATTATTAATTTTGAAATATTAAATGTGATATGAAG
TAAAGTAATTGTTATATGTATTGTGTGGTTAGAGGTTGTGTTGTTTGTGTGTGTATGTAT
GGATATAAAGTTATTTACGTTTCGGAATTTATGGGAAGGTTAAAGGGGAGTTTTTGGTTT
TAGTATTTAATTTTTATTAAGTTTGAGGTAGGTTTTTTTTTTGTTGTTAGTTGATGAATA
GTTTAGGTTAGTTGGAATATAGTTTTTGGGG
>HLTRMP8ES_SP6
GGTATTGGGATTAATATTTTTTTTAATTTTTTTGTTTTTTAAGTAGGTATTTTTTCGATG
TATTATATATTTAAATAATAAAATATTTTTAAAATTTTTTGTTAAATATGTAAAAGTATA
TTTTTGTAATTCATAAGTCGGTTTCGTAGGTGAAAGTATTTATTATAAAGGTTTGAGTTT
GGGTTTTGGAATATTAGGTATATTTTTGAAAGTTGTTTTTTGATTTTTGTATGTGTATTA
TGGAATGAGTATGTTTATTTTTATATAAAAATAGTAAATAAAGAAGTTTTAAAAATTTGT
TTTTAATTTTGATATATAGAATGAATATATTAATAAAATAGTTGATAGTGTAGATTATTG
GTAAAGTGAAAAATAGAAAAATATATATAATATAAAATTATATTTTAGTTTATATTTTAA
CGGATAAGTGGTTGGTAAGTTAAATATTATTAATTTTGAAATATTAAATGTGATATGAAG
TAAAGTAATTGTTATATGTATTGTGTGGTTAGAGGTTGTGTTGTTTGTGTGTGTATGTAT
GGATATAAAGTTATTCACGTTTCGGAATTTATGGGAAGGTTAAAGGGGAGTTTTTGGTTT
TAGTATTTAATTTTTATTAAGTTTGAGGTAGGTTTTTTTTTTGTTGTTAGTTGATGAATA
GTTTAGGTTAGTTGGAATATAGTTTTTGGGG
>HLTRMP9ES_SP6
GGTATTGGGATTAATATTTTTTTTAGTTTTTTTATTTTTTAAGTAAGTATTTTTTCGATG
TATTATATATTCAAACAATAAAATATTTTTAAAATTTTTTGTTAAATATGTAAAAGTATA
TTTTTGTAATTTATAAGTCGGTTTCGTAGGTGAAAGTATTTATTATAAAGGTTTGAGTTT
GGGTTTTGGAATATTAGGTATATTTTTGAAAGTTGTTTTTTGATTTTTGTATGTGTATTA
TGGAATGAGTATGTTTATTTTTATATAAAAATAGTAAATAAAGAAGTTTTAAAAATTTGT
TTTTAATTTTGATATATAGAATGAATATATTAATAAAATAGTTGATAGTGTAGATTATTG
GTAAAGTGAAAAATAGAAAAATATATATAATATAAAATTATATTTTAGTTTATATTTTAA
CGGATAAGTGATTGGTAAGTTAAATATTATTAATTTTGAAATATTAAATGTGATATGAAG
TAAAGTAATTGTTATATGTATTGTGTGGTTAGAGGTTGTGTTGTTTGTGTGTGTATGTAT
GGATATAAAGTTATTTACGTTTCGGAATTTATGGGAAGGTTAAAGGGGAGTTTTTGGTTT
TAGTATTTAATTTTTATTAAGTTTGAGGTAGGTTTTTTTTTTGTTGTTAGTTGATGAATA
GTTTAGGTTAGTTGGAATATAGTTTTTGGGG
>HLTRMP3BES_SP6
GGTATTGGGATTAATATTTTTTTTAGTTTTTTTATTTTTTAAGTAAGTATTTTTTTGATG
TATTATATATTTAAATAATAAAATATTTTTAAAATTTTTTGTTAAATATGTAAAAGTATA
TTTTTGTAATTTATAAGTTGGTTTTGTAGGTGAAAGTATTTATTATAAAGGTTTGAGTTT
GGGTTTTGGAATATTAGGTATATTTTTGAAAGTTGTTTTTTGATTTTTGTATGTGTATTA
TGGAATGAGTATGTTTATTTTTATATAAAAATAGTAAATAAAGAAGTTTTAAAAATTTGT
TTTTAATTTTGATATATAGAATGAATATATTAATAAAATAGTTGATAGTGTAGATTATTG
GTAAAGTGAAAAATAGAAAAATATATATAATATAAAATTATATTTTAGTTTATATTTTAA
CGGATAAGTGGTTGGTAAGTTAAATATTATTAATTTTGAAATATTAAATGTGATATGAAG
TAAAGTAATTGTTATATGTATTGTGTGGTTAGAGGTTGTGTTGTTTGTGTGTGTATGTAT
GGATATAAAGTTATTTATGTTTTGGAATTTATGGGAAGGTTAAAGGGGAGTTTTTGGTTT
TAGTATTTAATTTTTATTAAGTTTGAGGTAGGTTTTTTTTTTGTTGTTAGTTGGTGAATA
GTTTAGGTTAGTTGGAATATAGTTTTTGGGG
>HTransES3-M13R
GGTATTGGGATTAATATTTTTTTTAGTTTTTCTATTTTTTAAGTAAGTATTTTTTCGATG
TATTATATATTTAAATAATAAAATATTTTTAAAATTTTTTGTTAAATATGTAAAAGTATA
TTTTTGTAATTTATAAGTCGGTTTTGTAGGTGAAAGTATTTATTACAAAGGTTTGAGTTT
GGGTTTTGGAATATTAGGTATATTTTTGAAAGTTGTTTTTTGATTTTTGTATGTGTATCA
TGGAATGAGTATGTTTATTTTTATATAAAAATAGTAAATAAAGAAGTTTTAAAAATTTGT
TTTTAATTTTGATATATAGAATGAATATATTAATAAAATAGTTGATAGTGTAGATTATTG
GTAAAGTGAAAAATAGAAAAATATATATAATATAAAATTATATTTTAGTTTATATTTTAA
CGGATAAGTGGTTGGTAAGTTAAATATTATTAATTTTGAAATATTAAATGTGATATGAAG
TAAAGTAATTGTTATATGTATTGTGTGGTTAGAGGTTGTGTTGTTTGTGTGTGTATGTAT
GGATATAAAGTTATTTATGTTTTGGAATTTATGGGAAGGTTAAAGGGGAGTTTTTGGTTT
TAGTATTTAATTTTTATTAAGTTTGAGGTAGGTTTTTTTTTGTTGTTAGTTGATGAATAG
TTTAGGTTAGTTGGAATATAGTTTTTGGGG
>HTransES8-M13R
GGTATTGGGATTAATATTTTTTTTAGTTTTTTTATTTTTTAAGTAAGTATTTTTTCGATG
TATTATATATTTAAATAATAAAATATTTTTAAAATTTTTTGTTAAATATGTAAAAGTATA
TTTTTGTAATTTATAAGTCGGTTTCGTAGGTGAAAGTATTTATTATAAAGGTTTGAGTTT
GGGTTTTGGAATATTAGGTATATTTTTGAAAGTTGTTTTTTGATTTTTGTATGTGTATTA
TGGAATGAGTATGTTTATTTTTATATAAAAATAGTAAATAAAGAAGTTTTAAAAATTTGT
TTTTAATTTTGATATATAGAATGAATATATTAATAAAATAGTTGATAGTGTAGATTATTG
GTAAAGTGAAAAATAGAAAAATATATATAATATAAAATTATATTTTAGTTTATATTTTAA
CGGATAAGTGGTTGGTAAGATAAATATTATTAATTTTGAAATATTAAATGTGATATGAAG
TAAAGTAATTGTTATATGTATTGTGTGGTTAGAGGTTGTGTTGTTTGTGTGTGTATGTAT
GGATATAAAGTTATTTACGTTTCGGAATTTATGGGAAGGTTAAAGGGGAGTTTTTGGTTT
TAGTATTTAATTCTTATTAAGTTTGAGGTAGGTTTTTTTTTTGTTGTTAGTTGATGAATA
GTTTAGGTTAGTTGGAATATAGTTTTTGGGG
>HTransES16-M13R
GGTATTGGGATTAATATTTTTTTTAGTTTTTTTATTTTTTAAGTAAGTATTTTTTCGATG
TATTATATATTTAAACAATAAAATATTTTTAAAATTTTTTGTTAAATATGTAAAAGTATA
TTTTTGTAATTTATAAGTCGGTTTCGTAGGTGAAAGTATTTATTACAAAGGTTTGAGTTT
GGGTTTTGGAATATTAGGTATATTTTTGAAAGTTGTTTTTTGATTTTTGTATGTGTATTA
TGGAATGAGTATGTTTATTTTTATATAAAAATAGTAAATAAAGAAGTTTTAAAAATTTGT
TTTTAATTTTGATATATAGAATGAATATATTAATAAAATAGTTGATAGTGTAGATTATTG
GTAAAGTGAAAAATAGAAAAATATATATAATATAAAATTATATTTTAGTTTACATTTTAA
CGGATAAGTGGTTGGTAAGTTAAATATTATTAATTTTGAAATATTAAATGTGATATGAAG
TAAAGTAATTGTTATATGTATTGTGTGGTTAGAGGTTGTGTTGTTTGTGTGTGTATGTAT
GGATATAAAGTTATTTACGTTTCGGAATTTATGGGAAGGTTAAAGGGGAGTTTTTGGTTT
TAGTATTTAATTTTTATTAAGTTTGAGGTAGGTTTTTTTTTGTTGTTAGTTGATGAATAG
TTTAGGTTAGTTGGAATATAGTTTTTGGGG


129 Clones
>HLTRMP6ES_SP6
GGTATTGGGATTAATATTTTTTTTAGTTTTTTTATTTTTTAAGTAAGTATTTTTTCGATG
TATTATATATTTAAATAATAAAATATTTTTAAAATTTTTTGTTAAATATGTAAAAGTATA
TTTTTGTAATTTATAAGTCGGTTTCGTAGGTGAAAGTATTTATTATAAAGGTTTGAGTTT
GGGTTTTGGAATATTAGGTATATTTTTGAAAGTTGTTTTTTGATTTTTGTATGTGTATTA
TGGAATGAGTATGTTTATTTTTATATAAAAATAGTAAATAAAGAAGTTTTAAAAATTTGT
TTTTAATTTTGATATATAGAATGAATATATTAATAAAATAGTTGATAGTGTAGATTATTG
GTAAAGTGAAAAATAGAAAAATATATATAATATAAAATTATATTTTAGTTTATATTTTAA
CGGATAAGTGGTTGGTAAGTTAAATATTATTAATTTTGAAATATTAAATGTGATATGAAG
TAAGGTAATTGTTATATGTATTGTGTGGTTAGAGGTTGTGTTGTTTGTGTGTGTATGTAT
GGATATAAAGTTATTTACGTTTCGGAATTTACGGGAAGGTTAAAGGGGAGTTTTTGGTTT
TAGTATTTAATTTTCATTAAGTTTGAGGTAGGTTTTTTTTTGTTGTTAGTTGATAAGTAG
TTTAGGTTAGTTGGAATATAGTTTTTGGGG
>HLTRMP11ES_SP6
GGTATTGGGATTAATATTTTTTTTAGTTTTTTTATTTTTTAAGTAAGTATTTTTTCGATG
TATTATATATTTAAACAATAAAATATTTTTAAAATTTTTTGTTAAATATGTAAAAGTATA
TTTTTGTAATTTATAAGTTGGTTCCGTAGGTGAAAGTATTTATTATAAAGGTTTGAGTTT
GGGTTTTGGAATATTAGGTATATTTTTGAAAGTTGTTTTTTGATTTTTGTATGTGTATTA
TGGAATGAGTATGTTTATTTTTATATAAAAATAGTAAATAAAGAAGTTTTAAAAATTTGT
TTTTAATTTTGATATATAGAATGAATATATTAATAAAATAGTTGATAGTGTAGATTATTG
GTAAAATGAAAAATAGAAAAATATATATAATATAAAATTATATTTTAGTTTATATTTTAA
TGGATAAGTGGTTGGTAAGTTAAATATTATTAATTTTGAGATATTAAATGTGATATGAAG
TAAGGTAATTGTTATATGTATTGTGTGGTTAGAGGTTGTGTTGTTTGTGTGTGTATGTAT
GGATATAAAGTTATTTACGTTTCGGAATTTACGGGAAGGTTAAAGGGGAGTTTTTGGTTT
TAGTATTTAATTTTTATTAAGTTTGAGGTAGGTTTTTTTTTTGTTGTTAGTTGATAAGTA
GTTTAGGTTAGTTGGAATATAGTTTTTGGGG
>HLTRMP2BES_SP6
GGTATTGGGATTAATATTTTTTTTAGTTTTTTTATTTTTTAAGTAAGTATTTTTTCGATG
TATTATATATTTAAATAATAAAATATTTTTAAAATTTTTTGTTAAATATGTAAAAGTATA
TTTTTGTAATTTATAAGTCGGTTTCGTAGGTGAAAGTATTTATTATAAAGGTTTGAGTTT
GGGTTTTGGAATATTAGGTATATTTTTGAAAGTTGTTTTTTGATTTTTGTATGTGCATTA
TGGAATGAGTATGTTTATTTTTATATAAAAATAGTAAATAAAGAAGTTTTAAAAATTTGT
TTTTAATTTTGATATATAGAATGAATATATTAATAAAATAGTTGATAGTGTAGATTATTG
GTAAAGTGAAAAATAGAAAAATATATATAATATAAAATTATATTTTAGTTTACATTTTAA
CGGATAAGTGGTTGGTAAGTTAAATATTATTAATTTTGAAATATTAAATGTGATATGAAG
TAAGGTAATTGTTATATGTATTGTGTGGTTAGAGGTTGTGTTGTTTGTGTGTGTATGTAT
GGATACAAAGTTATTTACGTTTCGGAATTTACGGGAAGGTTAAAGGGGAGTTTTTGGTTT
TAGTATTTAATTTTTATTAAGTTTGAGGTAGGTTTTTTTTTTGTTGTTAGTTGATAAGTA
GTTTAGGTTAGTTGGAATATAGTTTTTGGGG
>HTransES6-M13R
GGTATTGGGATTAATATTTTTTTTAGTTTTTTTATTTTTTAAGTAAGTATTTTTTCGATG
TATTATATATTTAAATAATAAAATATTTTTAAAATTTTTTGTTAAATATGTAAAAGTATA
TTTTTGTAATTTATAAGTCGGTTTCGTAGGTGAAAGTATTTATTATAAAGGTTTGAGTTT
GGGTTTTGGAATATTAGGTATATTTTTGAAAGTTGTTTTTTGATTTTTGTATGTGTATTA
TGGAATGAGTATGTTTATTTTTATATAAAAATAGTAAATAAAGAAGTTTTAAAAATTTGT
TTTTAATTTTGATATATAGAATGAATATATTAATAAAACAGTTGATAGTGTAGATTATTG
GTAAAGTGAAAAATAGAAAAATATATATAATATAAAATTATATTTTAGTTTATATTTTAA
CGGATAAGTGGTTGGTAAGTTAAATATTATTAATTTTGAAATATTAAATGTGATATGAAG
TAAGGTAATTGTTATATGTATTGTGTGGTTAGAGGTTGTGTTGTTTGTGTGTGTATGTAT
GGATATAAAGTTATTTACGTTTTGGAATTTATGGGAAGGTTAAAGGGGAGTTTTTGGTTT
TAGTATTTAATTTTCATTAAGTTTGAGGTAGGTTTTTTTTTGTTGTTAGTTGATAAGTAG
TTTAGGTTAGTTGGAATATAGTTTTTGGGG
>HTransES15-M13R
GGTATTGGGATTAATATTTTTTTTAGTTTTTTTATTTTTTAAGTAAGTATTTTTTCGATG
TATTATATATTTAAATAATAAAATATTTTTAAAATTTTTTGTTAAATATGTAAAAGTATA
TTTTTGTAATTTATAAGTCGGTTTTGTAGGTGAAAGTATTTATCATAAAGGTTTGAGTTT
GGGTTTTGGAATATTAGGTATATTTTTGAAAGTTGTTTTTTGATTTTTGTATGTGTATTA
TGGAATGAGTATGTTTATTTTTATATAAAAATAGTAAATAAAGAAGTTTTAAAAATTTGT
TTTTAATTTTGATATATAGAATGAATATATTAATAAAATAGTTGATAGTGTAGATTATTG
GTAAAGTGAAAAATAGAAAAATATATATAATATAAAATTATATTTTAGTTTATATTTTAA
TGGATAAGTGGTTGGTAAGTTAAATATTATTAATTTTGAAATATTAAATGTGATATGAAG
TAAGGTAATTGTTATATGTATTGTGTGGTTAGAGGTTGTGTTGTTTGTGTGTGTATGTAT
GGATATAAAGTTATTTACGTTTTGGAATTTATGGGAAGGTTAAAGGGGAGTTTTTGGTTT
TAGTATTTAATTTTTACTAAGTTTGAGGTAGGTTTTCTTTTTGTTGTTAGTTGATAAGTA
GTTTAGGTTAGTTGGAATATAGTTTTTGGGG
>HTransES7_M13R
GGTATTGGGATTAATATTTTTTTTAGTTTTTTCATTTTTTAAGTAAGTATTTTTTCGATG
TATTATATATTTAAATAATAAAATATTTTTAAAATTTTTTGTTAAATATGTAAAAGTATA
TTTTTGTAATTTATAAGTCGGTTTCGTAGGTGAAAGTATTTATTATAAAGGTTTGAGTTT
GGGTTTTGGAATATTAGGTATATTTTTGAAAGTTGTTTTTTGATTTTTGTATGTGTATTA
TGGAATGAGTATGTTTATTTTTATATAAAAATAGTAAATAAAGAAGTTTTAAAAATTTGT
TTTTAATTTTGATATATAGAATGAATATATTAATAAAATAGTTGATAGTGTAGATTATTG
GTAAAGTGAAAAATAGAAAAATATATATAATATAAAATTATATTTTAGTTTATATTTTAA
CGGATAAGTGGTTGGTAAGTTAAATATTATTAATTTTGAAATATTAAATGTGATATGAAG
TAAGGTAATTGTTATATGTATTGTGTGGTTAGAGGTTGTGTTGTTTGTGTGTGTATGTAT
GGATATAAAGTTATTTATGTCTTGGAATTTACGGGAAGGTTAAAGGGGAGTTTTTGGTTT
TAGTATTTAATTTTTATTAAGTTTGAGGTAGGTTTTTTTTGTTGTTAGTTGATAAGTAGT
TTAGGTTAGTTGGAATATAGTTTTTGGGG
>HLTRMP8BR_SP6
GGTATTGGGATTAATATTTTTTTTAGTTTTTTTATTTTTTAAGTAAGTATTTTTTTGATG
TATTATATATTTAAATAATAAAATGTTTTTAAAATTTTTTGTTAAATATGTAAAAGTATA
TTTTTGTAATTTATAAGTCGGTTTCGTAGGTGAAAGTATTTATTATAAAGGTTTGAGTTT
GGGTTTTGGAATATTAGGTATATTTTTGAAAGTTGTTTTTTGATTTTTGTATGTGTATTA
TGGAATGAGTATGTTTATTTTTATATAAAAATAGTAAATAAAGAAGTTTTAAAAATTTGT
TTTTAATTTTGATATATAGAATGAATATATTAATAAAATAGTTGATAGTGTAGATTATTG
GTAAAGTGAAAAATAGAAAAATATATATAATATAAAATTATATTTTAGTTTATATTTTAA
CGGATAAGTGGTTGGTAAGTTAAATATTATTAATTTTGAAATATTAAATGTGGTATGAAG
TAAAGTAATTGTTATATGTATTGTGTGGTTAGAGGTTGTGTTGTTTGTGTGTGTATGTAT
GGATATAAAGTTATTTACGTTTTGGAATTTATGGGAAGGTTAAAGGGGAGTTTTTGGTTT
TAGTATTTAATTTTTATTAAGTTTGAGGTAGGTTTTTTTTTTGTTGTTAGTTGATGAATA
GTTTAGGTTAGTTGGAATATAGTTTTTGGGG
>HLTRMP9BR_SP6
GGTATTGGGATTAATATTTTTTTTAGTTTTTTTATTTTTTAAGTAAGTATTTTTTTGATG
TATTATATATTTAAATAATAAAATATTTTTAAAATTTTTTGTTAAATATGTAAAAGTATA
TTTTTGTAATTCATAAGTCGGTTTCGTAGGTGAAAGTATTTATTATAAAGGTTTGAGTTT
GGGTTTTGGAATATTAGGTATATTTTTGAAAGTTGTTTTTTGATTTTTGTATGTGTATTA
TGGAATGAGTATGTTTATTTTTATATAAAAATAGTAAATAAAGAAGTTTTAAAAATTTGT
TTTTAATTTTGATATATAGAATGAATATATTAATAAAATAGTTGATAGTGTAGATTATCG
GTAAAGTGAAAAATAGAAAAATATATATAATATAAAATTATATTTTAGTTTATATTTTAA
CGGATAAGTGGTTGGTAAGTTAAATATTATTAATTTTGAAATATTAAATGTGATATGAAG
TAAAGTAATTGTTATATGTATTGTGTGGTTAGAGGCTGTGTTGTTTGTGTGTGTATGTAT
GGATATAAAGTTATTTACGTTTCGGAATTTATGGGAAGGTTAAAGGGGAGTTTTTGGTTT
TAGTATTTAATTTTTATTAAGTTTGAGGTAGGTTTTTTTTGTTGTTAGTTGATGAATAGT
TTAGGTTAGTTGGAATATAGTTTTTGGGG
>HLTRMP3BBR_SP6
GGTATTGGGATTAATATTTTTTTTAGTTTTTTTATTTTTTAAGTAAGTATTTTTTTGATG
TATTATATATTTAAATAATAAAATATTTTTAAAATTTTTTGTTAAATATGTAAAAGTATA
TTTTTGTAATTTATAAGTCGGTTTTGTAGGTGAAAGTATTTATTATAAAGGTTTGAGTTT
GGGTTTTGGAATATTAGGTATATTTTTGAAAGTTGTTTTTTGATTTTTGTATGTGTATTA
TGGAATGAGTATGTTTATTTTTATATAAAAATAGTAAATAAAGAAGTTTTAAAAATTTGT
TTTTAATTTTGATATATAGAATGAATATATTAATAAAATAGTTGATAGTGTAGATTATTG
GTAAAGTGAAAAATAGAAAAATATATATAATATAAAAGTATATTTTAGTTTATATTTTAA
CGGATAAGTGGTTGGTAAGTTAAATATTATTAATTTTGAAATATTAAATGTGATATGAAG
TAAAGTAATTGTTATATGTATTGTGTGGTTAGAGGTTGTGTTGTTTGTGTGTGTATGTAT
GGATATAAAGTTATTTATGTTTCGGAATTTATGGGAAGGTTAAAGGGGAGTTTTTGGTTT
TAGTATTTAATTTTTATTAAGTTTGAGGTAGGTTTTTTTTGTTGTTAGTTGATGAATAGT
TTAGGTTAGTTGGAATATAGTTTTTGGGG
