# Supplementary material for: Epigenetic interplay between mouse endogenous retroviruses and host genes
Source: Genome Biol. 2012 Oct 3;13(10):R89. doi: 10.1186/gb-2012-13-10-r89 (PMC3491417; doi:10.1186/gb-2012-13-10-r89)
Supplement: Additional file 4 — All bisulfite sequencing data. Compilation of all bisulfite sequences. [file gb-2012-13-10-r89-S4.zip › IAP4305_TE_brain.rtf]

Hus1 “Real” LTR
B6129 Brain
Miniprep Sequences
B6 Clones
>HLTRBrMP3-M13R
GGGGAGTTTTTGGTTTTAGTATTTAATTTTCATTAAGTTTGAGGTAGGTTTTTTTTTGTT
GTTAGTTGATGAATAGTTTAGGTTAGTTGGAATATAGTTTTTGGGGAATTTATTTATTTT
TGTTTTTTATTTTACGGTAGGAATAATAGAATTATAAATATTTGTTAAGTTTATTTGTGT
GTGTGTTTTAAATGAGGATTTTAATTTGGTTTTTTTTTATACGCGTTTTCGCGATCGGTT
AGGAAGAATATAATAAATCGGAATTTTTTGCGGTAAAGTTTTATTGTTTATATTTTTAGG
AGATAGAGAGTAAGAGAGTAAGAGTTTTATTGTTTATATTTTTAGGAGTTAGAGCGTAAG
AGCGTAAGAGTTTTATTGTTTATATTTTTAGGAGTAAGAAGTAAGAGAGTAAGAGAGTAA
GAGTGTAAGAGTAAGAGAGAGTAAGAAAGTAAGAATAAGAATAAGAAAGTAAGAGAAAGA
ATGGTAAAATTTCGTTTTTTTTAAGGAGAATTATTTTTCGTTTAGGACGTATTATTTTTT
GATTGGTTGTAGTTTATCGGTTTAGTTGTTATTACGAGAAAGGTAGAATATATGGCGGGA
AAACTGTTTTTGTACGTGTGTAGATTATGTTTATTATTTAGAATATAGTTGT
>HLTRBrMP4-M13R
GGGGAGTTTTTGGTTTTAGTATTTAATTTTTATTAAGTTTGAGGTAGGTTTTTTTTGTTG
TTAGTTGATGAATAGTTTAGGTTAGTTGGAATATAGTTTTTGGGGAATTTATTTATTTTT
GTTTTTTATTTTACGGTAGGAATAATAGAATTATAAATATTTGTTAAGTTTATTTGTGTG
TGTGTTTTAAATGAGGATTTTAATTTGGTTTTTTTTTATACGCGTTTTCGCGATCGGTTA
GGAAGAATATAATAAATCGGAATTTTTTGCGGTAAAGTTTTATTGTTTATATTTTTAGGA
GATAGAGAGTAAGAGAGTAAGAGTTTTATTGTTTATATTTTTAGGAGTTAGAGCGTAAGA
GCGTAAGAGTTTTATTGTTTATATTTTTAGGAGTAAGAAGTAAGAGAGTAAGAGAGTAAG
AGTGTAAGAGTAAGAGAGAGTAAGAAAGTAAGAATAAGAATAAGAAAGTAAGAGAAAGAA
TGGTAAAATTTCGTTTTTTTAAGGAGAATTATTTTTCGTTTAGGACGTATTATTTTTTGA
TTGGTTGTAGTTTATCGGTTTAGTTGTTATTACGAGAAAGGTAGAATATATGGCGGGAAA
ATTGTTTTTGTACGTGTGTAGATTATGTTTATTATTTAGAATATAGTTGT
>HLTRBrMP5-M13R
GGGGAGTTTTTGGTTTTAGTATTTAATTTTTATTAAGTTTGAGGTAGGTTTTTTTTTGTT
GTTAGTTGATGAATAGTTTAGGTTAGTTGGAATATAGTTTTTGGGGAATTTATTTATTTT
TGTTTTTTATTTTACGGTAGGAATAATAGAATTATAAATATTTGTTAAGTTTATTTGTGT
GTGTGTTTTAAATGAGGATTTTAATTTGGTTTTTTTTTATACGCGTTTTCGCGATCGGTT
AGGAAGAATATAATAAATTGGAATTTTTTGCGGTAAAGTTTTATTGTTTATATTTTTAGG
AGACAGAGAGTAAGAGAGTAAGAGTTTTATTGTTTATATTTTTAGGAGTTAGAGCGTAAG
AGCGTAAGAGTTTTATTGTTTATATTTTTAGGAGTAAGAAGTAAGAGAGTAAGAGAGTAA
GAGTGTAAGAGTAAGAGAGAGTAAGAAAGTAAGAATAAGAATAAGAAAGTAAGAGAAAGA
ATGGTAAAATTTCGTTTTTTTTAAGGAGAATTATTTTTCGTTTAGGACGTATTATTTTTT
GACTGGTTGTAGTTTATCGGTTTAGTTGTTATTATGAGAAAGGTAGAATATATGGTGGGA
AAATTGTTTTTGTACGTGTGTAGATTATGTTTATTATTTAGAATATAGTTGT
>HLTRBrMP6-M13R
GGGGAGTTTTTGGTTTTAGTATTTAATTTTTATTAAGTTTGAGGTAGGTTTTTTTTTTGT
TGTTAGTTGATGAATAGTTTAGGTTAGTTGGAATATAGTTTTTGGGGAATTTATTTATTT
TTGTTTTTTATTTTACGGTAGGAATAATAGAATTATAAATATTTGTTAAGTTTATTTGTG
TGTGTGTTTTAAATGAGGATTTTAATTTGGTTTTTTTTTATACGCGTTTTCGCGATCGGT
TAGGAAGAATATAATAAATCGGAATTTTTTGCGGTAAAGTTTTATTGTTTATATTTTTAG
GAGATAGAGAGTAAGAGAGTAAGAGTTTTATTGTTTACATTTTTAGGAGTTAGAGCGTAA
GAGCGTAAGAGTTTTATTGTTTATATTTTTAGGAGTAAGAAGTAAGAGAGTAAGAGAGTA
AGAGTGTAAGAGTAAGAGAGAGTAAGAAAGTAAGAATAAGAATAAGAAAGTAAGAGAAAG
AATGGTAAAATTTCGTTTTTTTTAAGGAGAATTATTTTTCGTTTAGGACGTATTATTTTT
TGATTGGTTGTAGTTTATCGGTTTAGTTGTTATTACGAGAAAGGTAGAATATATGGCGGG
AAAATTGTTTTTGTACGTGTGTAGATTATGTTTATTATTTAGAATATAGTTGT
>HLTRBrMP7-M13R
GGGGAGTTTTTGGTTTTAGTATTTAATTTTTATTAAGTTTGAGGTAGGTTTTTTTTTTGT
TGTTAGTTGATGAATAGTTTAGGTTAGTTGGAATATAGTTTTTGGGGAATTTATTTATTT
TTGTTTTTTATTTTACGGTAGGAATAATAGAATTATAAATATTTGTTAAGTTTATTTGTG
TGTGTGTTTTAAATGAGGATTTTAATTTGGTTTTTTTTTTACACGCGTTTTCGCGATCGG
TTAGGAAGAATATAATAAATCGGAATTTTTTGCGGTAAAGTTTTATTGTTTATATTTTCA
GGAGATAGAGAGTAAGAGAGTAAGAGTTTTATTGTTTATATTTTTAGGAGTTAGAGCGTA
AGAGCGTAAGAGTTTTATTGTTTATATTTTTAGGAGTAAGAAGTAAGAGAGTAAGAGAGT
AAGAGTGTAAGAGTAAGAGAGAGTAAGAAAGTAAGAATAAGAATAAGAAAGTAAGAGAAA
GAATGGTAAAATTTCGTTTTTTTTAAGGAGAATTATTTTTCGTTTAGGACGTATTATTTT
TTGATTGGTTGTAGTTTATCGGTTTAGTTGTTATTACGAGAAAGGTAGAATATATGGCGG
GAAAATTGTTTTTGTACGTGTGTAGATTATGTTTATTATTTAGAATATAGTTGT
>HLTRBrMP8-M13R
GGGGAGTTTTTGGTTTTAGTATTTAATTTTTATTAAGTTTGAGGTAGGTTTTTTTTTGTT
GTTAGTTGATGAATAGTTTAGGTTAGTTGGAATATAGTTTTTGGGGAATTTATTTATTTT
TGTTTTTTATTTTACGGTAGGAATAATAGAATTATAAATATTTGTTAAGTTTATTTGTGT
GTGTGTTTTAAATGAGGATTTTAATTTGGTTTTTTTTTATACGCGTTTTCGTGATCGGTT
AGGAAGAATATAATAAATCGGAATTTTTTGCGGTAAAGTTTTATTGTTTATATTTTTAGG
AGATAGAGAGTAAGAGAGTAAGAGTTTTATTGTTTATATTTTTAGGAGTCAGAGCGTAAG
AGCGTAAGAGTTTTATTGTTTATATTTTTAGGAGTAAGAAGTAAGAGAGTAAGAGAGTAA
GAGTGTAAGAGTAAGAGAGAGTAAGAAAGTAAGAATAAGAATAAGAAAGTAAGAGAAAGA
ATGGTAAAATTTTGTTTTTTTTAAGGAGAATTATTTTTCGTTTAGGACGTATTATTTTTT
GATTGGTTGTAGTTTATCGGTTTAGTTGTTATTACGAGAAAGGTAGAATATATGGCGGGA
AAATTGTTTTTGTATGTGTGTAGATTATGTTTATTATTTAGAATATAGTTGT
>HLTRBrMP9-M13R
GGGGAGTTTTTGGTTTTAGTATTTAATTTTTATTAAGTTTGAGGTAGGTTTTTTTTTGTT
GTTAGTTGATGAATAGTTTAGGTTAGTTGGAATATAGTTTTTGGGGAATTTATTTATTTT
TGTTTTCTATTTTACGGTAGGAATAATAGAATTATAAATATTTGTTAAGTTTATTTGTGT
GTGTGTTTTAAATGAGGATTTTAATTTGGTTTTTTTTTATACGCGTTTTCGCGATCGGTT
AGGAAGAATATAATAAATCGGAATTTTTTGCGGTAAAGTTTTATTGTTTATATTTTTAGG
AGATAGAGAGTAAGAGAGTAAGAGTTTTATTGTTTATATTTTTAGGAGTTAGAGCGTAAG
AGCGTAAGAGTTTTATTGTTTATATTTTTAGGAGTAAGAAGTAAGAGAGTAAGAGAGTAA
GAGTGTAAGAGTAAGAGAGAGTAAGAAAGTAAGAATAAGAATAAGAAAGTAAGAGAAAGA
ATGGTAAAATTTCGTTTTTTTTAAGGAGAATTATTTTTCGTTTAGGACGTATTATTTTTT
GATTGGTTGTAGTTTATCGGTTTAGTTGTTATTACGAGAAAGGTAGAATATATGGCGGGA
AAATTGTTTTTGTACGTGTGTAGATTATGTTTATTATTTAGAATATAGTTGT
>HLTRBrMP10-M13R
GGGGAGTTTTTGGTTTTAGTATTTAATTTTTATTAAGTTTGAGGTAGGTTTTTTTTGTTG
TTAGTTGATGAATAGTTTAGGTTAGTTGGAATATAGTTTTTGGGGAATTTATTTATTTTT
GTTTTTTATTTTACGGTAGGAATAATAGAATTATAAATATTTGTTAAGTTTATTTGTGTG
TGTGTTTTAAATGAGGATTTCAATTTGGTTTTTTTTTATACGCGTTTTCGCGACCGGTTA
GGAAGAATATAATAAATCGGAATTTTTTGCGGTAAAGTTTTATTGTTTATATTTTTAGGA
GATAGAGAGTAAGAGAGTAAGAGTTTTATTGTTTATATTTTTAGGAGTTAGAGCGTAAGA
GCGTAAGAGTTTTATTGTTTATATTTTTAGGAGTAAGAAGTAAGAGAGTAAGAGAGTAAG
AGTGTAAGAGTAAGAGAGAGTAAGAAAGTAAGAATAAGAATAAGAAAGTAAGAGAAAGAA
TGGTAAAATTTCGTTTTTTTTAAGGAGAATTATTTTTCGTTTAGGACGTATTATTTTTTG
ATTGGTTGTAGTTTATCGGTTTAGTTGTTATTACGAGAAAGGTAGAATATATGGCGGGAA
AATTGTTTTTGTACGTGTGTAGATTATGTTTATTATTTAGAATATAGTTGT
>HLTRBrMP11-M13R
GGGGAGTTTTTGGTTTTAGTATTTAATTTTTATTAAGTTTGAGGTAGGTTTTTTTTTGTT
GTTAGTTGATGAATAGTTTAGGTTAGTTGGAGTATAGTTTTTGGGGAATTTATTTATTTT
TGTTTTTTATTTTACGGTAGGAATAATAGAATTATAAATATTTGTTAAGTTTATTTGTGT
GTGTGTTTTAAATGAGGATTTTAATTTGGTTTTTTTTTATACGCGTTTTCGCGATCGGTT
AGGAAGAATATAATAAATCGGAATTTTTTGCGGTAAAGTTTTATTGTTTATATTTTTAGG
AGATAGAGAGTAAGAAAGTAAGAGTTTTATTGTTTATATTTTTAGGAGTTAGAGCGTAAG
AGCGTAAGAGTTTTATTGTTTATATTTTTAGGAGTAAGAAGTAAGAGAGTAAGAGAGTAA
GAGTGCAAGAGTAAGAGAGAGTAAGAAAGTAAGAATAAGAATAAGAAAGTAAGAGAAAGA
ATGGTAAAATTTCGTTTTTTTTAAGGAGAATTATTTTTCGTTTAGGACGTATTATTTTTT
GATTGGTTGTAGTTTATCGGTTTAGTTGTTATTACGAGAAAGGTAGAATATATGGCGGGA
AAATTGTTTTTGTACGTGTGTAGATTATGTTTATTATTTAGAATATAGTTGT
>HLTRBrMP13-M13R
GGGGAGTTTTTGGTTTTAGTATTTAATTTTTATTAAGTTTGAGGTAGGTTTTTTTTTGTT
GTTAGTTGATGAATAGTTTAGGTTAGCTGGAACATAGTTTTTGGGGAATTTATTTATTTT
TGTTTTTTATTTTACGGTAGGAATAATAGAATTATAAATATTTGTTAAGTTTATTTGTGT
GTGTGTTTTAAATGAGGATTTTAATTTGGTTTTTTTTTATACGCGTTTTCGCGATCGGTT
AGGAAGAATATAATAAATCGGAATTTTTTGCGGTAAAGTTTTATTGTTTATATTTTTAGG
AGATAGAGAGTAAGAGAGTAAGAGTTTTATTGTTTACATTTTTAGGAGTTAGAGCGTAAG
AGCGTAAGAGTTTTATTGTTTATATTTTTAGGAGTAAGAAGTAAGAGAGTAAGAGAGCAA
GAGTGTAAGAGTAAGAGAGAGTAAGAAAGTAAGAATAAGAATAAGAAAGTAAGAGAAAGA
ATGGTAAAATTTCGTTTTTTTTAAGGAGAATTATTCTTCGTTTAGGACGTATTATTTTTT
GATTGGTTGTAGTTTATCGGTTTAGTTGTTATTACGAGAAAGGTAGAATATATGGCGGGA
AAATTGTTTTTGTACGTGTGTAGATTATGTTTATTATTTAGAATATAGTTGT
>HLTRBrMP14-M13R
GGGGAGTTTTTGGTTTTAGTATTTAATTTTTATTAAGTTTGAGGTAGGTTTTTTTTTGTT
GTTAGTTGATGAATAGTTTAGGTTAGTTGGAGTATAGTTTTTGGGGAATTTATTTATTTT
TGTTTTTTATTTTACGGTAGGAATAATAGAATTATAAATATTTGTTAAGTTTATTTGTGT
GTGTGTTTTAAATGAGGATTTTAATTTGGTTTTTTTTTATACGCGTTTTCGCGATCGGTT
AGGAAGAATATAATAAATCGGAATTTTTTGCGGTAAAGTTTTATTGTTTATATTTTTAGG
AGATAGAGAGTAAGAAAGTAAGAGTTTTATTGTTTATATTTTTAGGAGTTAGAGCGTAAG
AGCGTAAGAGTTTTATTGTTTATATTTTTAGGAGTAAGAAGTAAGAGAGTAAGAGAGTAA
GAGTGCAAGAGTAAGAGAGAGTAAGAAAGTAAGAATAAGAATAAGAAAGTAAGAGAAAGA
ATGGTAAAATTTCGTTTTTTTTAAGGAGAATTATTTTTCGTTTAGGACGTATTATTTTTT
GATTGGTTGTAGTTTATCGGTTTAGTTGTTATTACGAGAAAGGTAGAATATATGGCGGGA
AAATTGTTTTTGTACGTGTGTAGATTATGTTTATTATTTAGAATATAGTTGT
>HLTRBrMP15-M13R
GGGGAGTTTTTGGTTTTAGTATTTAATTTTTATTAAGTTTGAGGTAGGTTTTTTTTTGTT
GTTAGTTGATGAATAGTTTAGGTTAGTTGGAATATAGTTTTTGGGGAATTTATTTATTTT
TGTTTTTTATTTTACGGTAGGAATAATAGAATTATAAATATTTGTTAAGTTTATTTGTGT
GTGTGTTTTAAATGAGGATTTTAATTTGGTTTTTTTTTACACGCGTTTTCGCGATCGGTT
AGGAAGAATATAATAAATCGGAATTTTTTGCGGTAAAGTTTTATTGTTTATATTTTTAGG
AGATAGAGAGTAAGAGAGTAAGAGTTTTATTGTTTATATTTTTAGGAGTTAGAGCGTAAG
AGCGTAAGAGTTTTATTGTTTATATTTTTAGGAGTAAGAAGTAAGAGAGTAAGAGAGTAA
GAGTGTAAGAGTAAGAGAGAGTAAGAAAGTAAGAATAAGAATAAGAAAGTAAGAGAAAGA
ATGGTAAAATTTCGTTTTTTTTAAGGAGAATTATTTTTCGTTTAGGACGTATTATTTTTT
GATTGGTTGTAGTTTATCGGTTTAGTTGTTATTACGAGAAAGGTAGAATATATGGCGGGA
AAATTGTTTTTGTACGTGTGTAGATTATGTTTATTATTTAGAATATAGTTGT
>HLTRBrMP16-M13R
GGGGAGTTTTTGGTTTTAGTATTTAATTTTTATTAAGTTTGAGGTAGGTTCTCTTTTTGT
TGTTAGTTGATGAATAGTTTAGGTTAGTTGGAATATAGTTTTTGGGGAATTTATTTATTT
TTGTTTTTTATTTTACGGTAGGAATAATAGAATTATAAATATTTGTTAAGTTTATTTGTG
TGTGTGTTTTAAATGAGGATTTTAATTTGGTTTTTTTTTATACGCGTTTTCGCGATCGGT
TAGGAAGAATATAATAAATCGGAATTTTTTGCGGTAAAGTTTTATTGTTTATATTTTTAG
GAGATAGAGAGTAAGAGAGTAAGAGTTTTATTGTTTATATTTTTAGGAGTTAGAGCGTAA
GAGCGTAAGAGTTTTATTGTTTATATTTTTAGGAGTAAGAAGTAAGAGAGTAAGAGAGTA
AGAGTGTAAGAGTAAGAGAGAGTAAGAAAGTAAGAATAAGAATAAGAAAGTAAGAGAAAG
AATGGTAAAATTTCGTTTTTTTTAAGGAGAATTATTTTTCGTTTAGGACGTATTATTTTT
TGATTGGTTGTAGTTTATCGGTTTAGTTGTTATTACGAGAAAGGTAGAATATATGGCGGG
AAAATTGTTTTTGTACGTGTGTAGATTATGTTTATTATTTAGAATATAGTTGT
