# Supplementary material for: Epigenetic interplay between mouse endogenous retroviruses and host genes
Source: Genome Biol. 2012 Oct 3;13(10):R89. doi: 10.1186/gb-2012-13-10-r89 (PMC3491417; doi:10.1186/gb-2012-13-10-r89)
Supplement: Additional file 4 — All bisulfite sequencing data. Compilation of all bisulfite sequences. [file gb-2012-13-10-r89-S4.zip › IAP4305_TE_ES.rtf]

Hus1 LTR Miniprep Sequences
B6129 ES
B6 Clones
>HLTRES-2R
GGGGAGTTTTTGGTTTTAGTATTTAATTTTTATTAAGTTTGAGGTAGGTTTTTTTTTTGT
TGTTAGTTGATGAATAGTTTAGGTTAGTTGGAATATAGTTTTTGGGGAATTTATTTATTT
TTGTTTTTTATTTTACGGTAGGAATAATAGAATTATAAATATTTGTTAAGTTTATTTGTG
TGTGTGTTTTGAATGAGGATTTCAATTTGGTTTTTTTTTATACGCGTTTTCGCGATCGGT
TAGGAAGAATATAATAAATCGGAATTTTTTGCGGTAAAGTTTTATTGTTTATATTTTTAG
GAGATAGAGAGTAAGAGAGTAAGAGTTTTATTGTTTATATTTTTAGGAGTTAGAGCGTAA
GAGCGTAAGAGTTTTATTGTTTATATTTTTAGGAGTAAGAAGTAAGAGAGTAAGAGAGTA
AGAGTGTAAGAGTAAGAGAGAGTAAGAAAGTAAGAATAAGAATAAGAAAGTAAGAGAAAG
AATGGTAAAATTTCGTTTTTTTTAAGGAGAATTATTTTTCGTTTAGGACGTATTATTTTT
TGATTGGTTGTAGTTTATCGGTTTAGTTGTTATTACGAGAAAGGTAGAATATATGGCGGG
AAAATTGTTTTTGTATGTGTGTAGATTATGTTTATTATTTAGAATATAGTTGT
>HLTRES-8R
GGGGAGTTTTTGGTTTTAGTATTTAATTTTTATTAAGTTTGAGGTAGGTTTTTTTTTTGT
TGTTAGTTGATGAATAGTTTAGGTTAGTTGGAATATAGTTTTTGGGGAATTTATTTATTT
TTGTTTTTTATTTTACGGTAGGAATAATAGAATTATAAATATTTGTTAAGTTTATTTGTG
TGTGTGTTTTAAATGAGGATTTTAATTTGGTTTTTTTTTATACGCGTTTTCGCGATCGGT
TAGGAAGAATATAATAAATCGGAATTTTTTGCGGTAAAGTTTTATTGTTTATATTTTTAG
GAGATAGAGAGTAAGAGAGTAAGAGTTTTATTGTTTATATTTTTAGGAGTTAGAGCGTAA
GAGCGTAAGAGTTTTATTGTTTATATTTTTAGGAGTAAGAAGTAAGAGAGTAAGAGAGTA
AGAGTGTAAGAGTAAGAGAGAGTAAGAAAGTAAGAACAAGAATAAGAAAGTAAGAGAAAG
AATGGTAAAATTTTGTTTTTTTTAAGGAGAATTATTTTTCGTTTAGGACGTATTATTCTT
TGATTGGTTGTAGTTTATTGGTTTAGTTGTTATTACGAGAAAGGTAGAATATATGGCGGG
AAAATTGTTTTTGTACGTGTGTAGATTATGTTTATTATTTAGAATATAGTTGT
>HLTRES-9R
GGGGAGTTTTTGGTTTTAGTATTTAATTTTTATTAAGTTTGAGGTAGGTTTTTTTTTGTT
GTTAGTTGATGAATAGTTTAGGTTAGTTGGAATATAGTTTTTGGGGAATTTATTTATTTT
TGTTTTTTATTTTACGGTAGGAATAATAGAATTATAAATATTTGCTAAGTTTATTTGTGT
GTGTGTTTTAAATGAGGATTTTAATTTGGTTTTTTTTTATACGCGTTTTCGCGATCGGTT
AGGAAGAATATAATAAATCGGAATTTTTTGCGGTAAAGTTTTATTGTTTATATTTTTAGG
AGATAGAGAGTAAGAGAGTAAGAGTTTTATTGTTTATATTTTTAGGAGTTAGAGTGTAAG
AGCGTAAGAGTTTTATTGTTTACATTTTTAGGAGTAAGAAGTAAGAGAGCAAGAGAGTAA
GAGTGTAAGAGTAAGAGAGAGTAAGAAAGTAAGAATAAGAATAAGAAAGTAAGAGAAAGA
ATGGTAAAATTTTGTTCTTTTTAAGGAGAATTATTTTTTGTTTAGGACGTATTATTTTTT
GATTGGTTGTAGTTTATCGGTTTAGTTGTTATTACGAGAAAGGTAGAATATATGGTGGGA
AAATTGTTTTTGTACGTGTGTAGATTATGTTTATTATTTAGAATATAGT
>HLTRES-10R
GGGGAGTTTTTGGTTTTAGTATTTAATTTTTATTAAGTTTGAGGTAGGTTTTTTTTTGTT
GTTAGTTGATGAATAGTTTAGGTTAGTTGGAATATAGTTTTTGGGGAATTTATTTATTTT
TGTTTTTTATTTTACGGTAGGAATAATAGAATTATAAATATTTGCTAAGTTTATTTGTGT
GTGTGTTTTAAATGAGGATTTTAATTTGGTTTTTTTTTATACGCGTTTTCGCGATCGGTT
AGGAAGAATATAATAAATCGGAATTTTTTGCGGTAAAGTTTTATTGTTTATATTTTTAGG
AGATAGAGAGTAAGAGAGTAAGAGTTTTATTGTTTATATTTTTAGGAGTTAGAGTGTAAG
AGCGTAAGAGTTTTATTGTTTACATTTTTAGGAGTAAGAAGTAAGAGAGCAAGAGAGTAA
GAGTGTAAGAGTAAGAGAGAGTAAGAAAGTAAGAATAAGAATAAGAAAGTAAGAGAAAGA
ATGGTAAAATTTTGTTCTTTTTAAGGAGAATTATTTTTTGTTTAGGACGTATTATTTTTT
GATTGGTTGTAGTTTATCGGTTTAGTTGTTATTACGAGAAAGGTAGAATATATGGTGGGA
AAATTGTTTTTGTACGTGTGTAGATTATGTTTATTATTTAGAATATAGTTGT
>HLTRES-12R
GGGGAGTTTTTGGTTTTAGTATTTAATTTTTATTAAGTTTGAGGTAGGTTTTTTTTTTGT
TGTTAGTTGATGAATAGTTTAGGTTAGTTGGAATATAGTTTTTGGGGAATTTATTTATTT
TTGTTTTTTATTTTACGGTAGGAATAATAGAATTATAAATATTTGTTAAGTTTATTTGTG
TGTGTGTTTTAAATGAGGATTTTAATTTGGTTTTTTTTTATACGTGTTTTCGTGATTGGT
TAGGAAGAATATAATAAATCGGAATTTTTTGCGGTAAAGTTTTATTGTTTATATTTTCAG
GAGATAGAGAGTAAGAGAGTAAGAGTTTTATTGTTTATATTTTTAGGAGTTAGAGCGTAA
GAGCGTAAGAGTTTTATTGTTTATATTTTTAGGAGTAAGAAGTAAGAGAGTAAGAGAGTA
AGAGTGTAAGAGTAAGAGAGAGTAAGAAGGTAAGAATAAGAATAAGAAAGTAAGAGAAAG
AATGGTAAAATTTCGTTTTTTTTAAGGAGAATTATTTTTCGTTTAGGACGTATTATTTTT
TGATTGGTTGTAGTTTATCGGTTTAGTTGTTATTACGAGAAAGGTAGAATATATGGCGGG
AAAATTGTTTTTGTATGTGTGTAGATTATGTTTATTATTTAGAATATAGTTGT
>HLTRES-14R
GGGGAGTTTTTGGTTTTAGTATTTAATTTTTATTAAGTTTGAGGTAGGTTTTTTTTTTGT
TGTTAGTTGATGAATAGTTTAGGTTAGTTGGAATATAGTTTTTGGGGAATTTATTTATTT
TTGTTTTTTATTTTACGGTAGGAATAATAGAATTATAAATATTTGTTAAGTTCATTTGTG
TGTGTGTTTTAAATGAGGATTTTAATTTGGTTTTTTTTTATACGCGTTTTCGCGATCGGT
TAGGAAGAGTATAATAAATCGGAATTTTTTGCGGTAAAGTTTTATTGTTTATATTTTTAG
GAGATAGAGAGTAAGAGAGTAAGAGTTTTATTGTTTATATTTTTAGGAGTTAGAGCGTAA
GAGCGTAAGAGTTTTATTGTTTATATTTTTAGGAGTAAGAAGTAAGAGAGTAAGAGAGTA
AGAGTGTAAGAGTAAGAGAGAGTAAGAAAGTAAGAATAAGAATAAGAAAGTAAGAGAAAG
AATGGTAAAATTTTGTTTTTTTTAAGGAGAATTATTTTTCGTTTAGGATGTATTATTTTT
TGATTGGTTGTAGTTTATCGGTTTAGTTGTTATTATGAGAAAGGTAGAATATATGGCGGG
AAAATTGTTTTTGTACGTGTGTAGATTATGTTTATTATTTAGAATATAGT
>HLTRES-15R
GGGGAGTTTTTGGTTTTAGTATTTAATTTTTATTAAGTTTGAGGTAGGTTTTTTTTTTGT
TGTTAGTTGATGAATAGTTTAGGTTAGTTGGAATATAGTTTTTGGGGAATTTATTTATTT
TTGTTTTTTATTTCACGGTAGGAATAATAGAATTACAAATATTTGTTAAGTTTATTTGTG
TGTGTGTTTTAAATGAGGATTTTAATTTGGTTTTTTTTTATACGCGTTTTCGCGATCGGT
TAGGAAGAACATAATAAATCGGAATTTTTTGCGGTAAAGTTTTATTGTTTATATTTTTAG
GAGATAGAGAGTAAGAGAGTAAGAGTTTTATTGTTTATATTTTTAGGAGTTAGAGCGTAA
GAGCGTAAGAGTTTTATTGTTTATATTTTTAGGAGTAAGAAGTAAGAGAGTAAGAGAGTA
AGAGTGTAAGAGTAAGAGAGAGTAAGAAAGTAAGAATAAGAATAAGAAAGTAAGAGAAAG
AATGGTAAAATTTTGTTTTTTTTAAGGAGAATTATTTTTCGTTTAGGACGTATTATTTTT
TGATTGGTTGTAGTTTATCGGTTTAGTTGTTATTACGAGAAAGGTAGAATATATGGCGGG
AAAATTGTTTTTGTACGTGTGTAGATTATGTTTATTATTTAGAATATAGTTGT
>HLTRES-16R
GGGGAGTTTTTGGTTTTAGTATTTAATTTTTATTAAGTTTGAGGTAGGTTTTTTTTTGTT
GTTAGTTGATGAATAGTTTAGGTTAGTTGGAATATAGTTTTTGGGGAATTTATTTATTTT
TGTTTTTTATTTTACGGTAGGAATAATAGAATTATAAATATTTGTTAAGTTTATTTGTGT
GTGTGTTTTAAATGAGGATTTTAATTTGGTTTTTTTTACACGCGTTTTCGCGATCGGTTA
GGAAGAATATAATAAATCGGAATTTTTTGCGGTAAAGTTTTATTGTTTATATTTTTAGGA
GATAGAGAGTAAGAGAGTAAGAGTTTTATTGTTTACATTTTTAGGAGTTAGAGCGTAAGA
GCGTAAGAGTTTTATTGTTTATATTTTCAGGAGTAAGAAGTAAGAGAGTAAGAGAGTAAG
AGTGTAAGAGTAAGAGAGAGTAAGAAAGTAAGAATAAGAATAAGAAAGTAAGAGAAAGAA
TGGTAAAATTTCGTTTTTTTTAAGGAGAATTATTTTTCGTTTAGGACGTATTATTTTTTG
ATTGGTTGTAGTTTATCGGTTTAGTTGTTATTACGAGAAAGGTAGAATATATGGCGGGAA
AATTGTTTTTGTACGTGTGTAGATTATGTTTATTATTTAGAATATAGTTGT
