# Supplementary material for: Epigenetic interplay between mouse endogenous retroviruses and host genes
Source: Genome Biol. 2012 Oct 3;13(10):R89. doi: 10.1186/gb-2012-13-10-r89 (PMC3491417; doi:10.1186/gb-2012-13-10-r89)
Supplement: Additional file 4 — All bisulfite sequencing data. Compilation of all bisulfite sequences. [file gb-2012-13-10-r89-S4.zip › IAP6428_emptysite_ES.rtf]

12 21 10 J1 Trans MP Sequences
Plate A
>J1T_87
TGTTGAGGTTAGGATATTTTCTGGGTGAGAGAGAGGTGTAGTTTTGTAATTATTGTTTAG
GAAGTTTATATTTTTAGTTATAAATTTAAGTTTAGGTATATATATATATATATATACATA
TATATATATATATATATATGCATATACATATATATTTTGATTTTATTAATTGTAAGAGAT
TTGAGTAAAGAAATTAAGATTTAAGGGGCGTTGATATTTTGTTAGTTTTTTAGGTTGTTT
TAAAATTTGAGGTATATGTTTTTATTTTGGTATGTTTTTTTTGTTTTAGTTGGAATTTTT
TGATTTGTGTGTATTTAGTATATAGTGAATATTTAAAGTTTTGTGTTTTGATAATTTAAA
GATTAGATTAAATTTCTTGTATTTAGTTAAATTTATATATAGGTTGAAGGGTAGGTATAT
TT
>J1T_88
AAGATTGGGATTTTAGAAATTTGATCGGTAAAGTTTTTATGTTGGTTATTTTTATTTTTT
TTTGTTTGGGTTTGTGGTTTTTGAAGTTTAGACGTTTTTTTTTTTATGTTGAGGTTAGGA
TATTTTTGGGTGAGAGAGAGGTGTAGTTTTGTAATTATTGTTTAGGAAGTTATATTTTTA
GTTATAAATTTAAGTTTAGGTATATATATATACATATATACATATATATACATATACATA
TATACGTATATATATATATATTTCGATTTTATTAATTGTAAGAGATTTGAGTAAAGAAAT
TAAGATTTAAGGGGTGTTGATATTTTGTTAGTTTTTTAGGTTGTTTTAAAATTTGAGGTA
TATGTTTTTATTTCGGTATGTTTTTTTTGTTTTAGTTGGAATTTTTTGATTCGTGTGTAT
TTAGTATATAGTGAATATTTAAAGTTTTGTGTTTTGATAATTTAAAGATTAGATTAAATT
TTTTGTATTTATTTAAATTTATATATAGGTTTGAAGGGTAGGTATATTT
>J1T_89
AAGATTGGGATTTTAGAAATTTGATTGGTAAAGTTTTTATGTTGGATTATTTTTATTTTT
TTTTGTTTGGGTTTGTGGTTTTTGAAGTCTAGATGTTTTTTTTTTATGTTGAGGTTAGGA
TATCTTCGGGTGAGAGAGAGGTGTAGTTTTGTAATTATTGTTAGGAAGTTATATTTTTAG
TTATAAATTTAAGTTTAGGTATATATATATATATACATATATATATATATATATACATAT
ATATGTATATATATATATATTTCGATTTTATTAATTGTAGGAGATTTGAGTAAAGAAATT
AAGATTTAAGGGGCGTTGATATTTTGTTAGTTTTCTAGGTTGTTTTAAAATTTGAGGTAT
ATGTTTTTATTTCGGTATGTTTTTTTTGTTTTAGTTGGAATTTTTTGATTTGTGTGTATT
TAGTATATAGTGAATATTT
>J1T_90
AGAAATTTGATTGGTAAAGTTTTTATGTTCGGTTATTTTTTATTTTTTTTTGTTTGGGTT
TGTGGTTTTTGAAGTTTAGACGTTTTTTTTTTTATGTCACGTTAGGATATTTTTGGGTGA
GAGAGAGGTGTAGTTTTGTAATTATTGTTTAGGAAGTTATATTTTTAGTTATAAATTTAA
GTTTAGGTATATATATATACATATATATATACATATATATATATATATATATGTATATAT
ATATATATTTTGATTTTATTAATTGTAAGAGATTTGAGTAAAGAAATTAAGATTTAAGGG
GCGTTGATATTTTGTTAGTTTTTTAGGTTGTTTTAAAATTTGAGGTATATGTTTTTATTT
CGGTATGTTTTTTTTGTTTTAGTTGGAATTTTTTGATTCGTGTGTATTTAGTATATAGTG
AATATTTAAAGTTTTGTGTTTTGATAATTTAAAGATTAGATTAAATTTTTTGTATTTATT
TAAATTTATATATAGGTTTGAAGGGTAGGTATTATTT
>J1T_91
AAGATTGGGATTTTAGAAATTTGATTGGTAAAGTTTTTATGTTGGTTATTTTTATTTTTT
TTGTTTGGGTTTGTGGTTTTTGAAGTTTAGATGTTTTTTTTTTTTATGTTGAGGTTAGGA
TATTTTTGGGTGAGAGAGGGGTGTAGTTTTGTAATTATTGTTTAGGAAGTTATATTTTTA
GTTATAAATTTAAGTTTAGGTATATGTATACATATATATATATATATATATATATATGTA
CATATGTATATATATATATGTTTTGATTTTATTAATTGTAAGAGATTTGAGTAAAGAAAT
TAAGATTTAAGGGGTGTTGATATTTTGTTAGTTTTTTAGGTTGTTTTAAAACTTGAGGTA
TATGTTTTTATTTTGGTATGTTTTTTTTGTTTTAGTTGGAATTTTTTGATTTGTGTGTAT
TTAGTATATAGTGAATATTTAAAGCTTTGTGTTTTGATAATTTAAAGATT

>J1T_92
AAGATTGGGATTTTCGAAATTTGATTGGTAAAGTTTTTTATGTTGGTTATTTTTATTTTT
TTTTGTTTGGGTTTGTGGTTTTTGAAGTTTAGATGTTTTTTTTTTTATGTTGAGGTTAGC
ATATCTTCGGGTGAGAGAGAGGTGTAGTTTTGTAATTATTGTTTAGGAAGTTATATTTTT
AGTTATAAATTTAAGTTTAGGTATATATATATATATACATATATATATATATATATACAT
ATATATGTATATATATATATATTTCGATTTTATTAATTGTAAGAGATTTGAGTAAAGAAA
TTAAGATTTAAGGGGCGTTGATATTTTGTTAGTTTTCTAGGTTGTTTTAAAATTTGAGGT
ATATGTTTTTATTTCGGTATGTTTTTTTTGTTTTAGTTGGAATTTTTTGATTTGTGTGTA
TTTAGTATATAGTGAATATTTAAAGTTTTGTGTTTTGATAATTTAAAGATTAGATTAAAT
TTTTTGCATTTATTTAAATTTATATATAGGTTTGAAGGGTAGGTATATTT
>J1T_93
AAGATTGGGATTTTTAGAAACTTGATTGGTAAAGTTTTTATGTTGGTTATTTTTATTTTT
TTTTGTTTGGGTTTGTGGTTTTTGAAGTTTAGATGTTTTTTTTTTTTATGTTGAGGTTAG
GATATTTTTGGGTGAGAGAGAGGTGTAGTTTTGTAATTATTGTTTAGGAAGTTATATTTT
TAGTTATAAATTTAAGTTTAGGTATATATATATATATATATATATATACATATATATATA
TACGTATACATATATATATTTCGATTTTATTAATTGTAAGAGATTTGAGTAAAGGAATTA
AGATTTAAGGGGCGTTGATATTTTGTTAGTTTTTTAGGTTGTTTTAAAATTTGAGGTATA
TGTTTTTATTTCGGTATGTTTTTTTTGTTTTAGTTGGAATTTTTTGATTCGTGTGTATTT
AGTATATAGTGAATATTTAAAGTTTTGTGTTTTGATAATTTAAAGATTAGATTAAATTTT
TTGTATTTATTTAAATTTATATATAGGTTTGAAGGGTAGGTTATATTT
>J1T_94
AAGATTGGGATTTTAGAAATTTGATTGGTAAAGTTTTTATGTTGGTTATTTTTATTTTTT
TTTGTTTGGGTTTGTGGTTTTTGAAGTTTAGATGTTTTTTTTTTTATGTTAGGTTAGGAT
ATTTTTGGGTGAGAGAGAGGTGTAGTTTTGTAATCATTGTTTAGGAAGTTATATTTTTAG
TTATAAATTTAAGTTTAGGTATATATATATATATATATATACATATACATATATACATAT
ATACGTATATATATATATATTTTGATTTTATTAATTGTAAGAGATTTGAGTAAAGAAATT
AAGATTTAAGGGGCGTTGATATTTTGTTAGTTTTTTAGGTTGTTTTAAAATTTGAGGTAT
ATGTTTTTATTTTGGTATGTTTTTTTTGTTTTAGTTGGAATTTTTTGATTTGTGTGTATT
TAGTATATAGTGAATATTTAAAGTTTTGTGTTTTGATAATTTAAAGATT
>J1T_96
AAGATTGGGATTTTAGAAATTTGATTGGTAAAGTTTTTATGTTGGTTATTTTTATTTTTT
TTTGTTTGGGTTTGTGGTTTCTGAAGTTTAGATGTTTTTTTTTTTATGTTGAGGTTAGGA
TATTTTTGGGTGAGAGAGAGGTGTAGTTTTGTAATTATTGTTTAGGAAGTTATATTTTTA
GTTATAAATTTAAGTTTAGGTATATATATATATATATACATATATATATATATATACATA
CGTATATATATATATATTTCGATTTTATTAATTGTAAGAGATTTGAGTAAAGAAATTAAG
ATTTAAGGGGCGTTGATATTTTGTTAGTTTTTTAGGTTGTTTTAAAATTTGAGGTATATG
TTTTTATTTCGGTATGTTTTTTTTGTTTTAGTTGGAATTTTTTGATTCGTGTGTATTTAG
TATATAGTGAATATTTAAAGTTTTGTGTTTTGATAATTTAAAGATTAGATTAAATTTTTT
GTATTTATTTAAATTTATATATAGGTTTGAAGGGTAGGTATATTT
Plate B
>J1T_78
GAAGTTTAGATGTTTTTTTTTTTATGCTGAGGTTAGGATATTTTTGGGTGAGAGAGAGGT
GTAGTTTTGTAATCATTGTTTTAGGAAGTTATATTTTTAGTTATAAATTTAAGTTTAGGT
ATATATACACATACACATATATACATATATATACACATATACATGTATATATATATATAT
TTTGATTTTATTAATTGTAAGAGATTTGAGTAAAGAAATTAAGATTTAAGGGGCGTTGAT
ATTTTGTTAGTTTTTTAGGTTGTTTTAAAATTTGAGGTATATGTTTTTATTTTGGTATGT
TTTTTTTGTTTTAGTTGGAATTTTTTGATTTGTGTGTATTTAGTATATAGTGAATATTTA
AAGTTTTGTGTTTTGATAATTTAAAGATTAGATTAAATTTTTTGTATTTATTTAAATTTA
TATATAGGTTGAAGGGTAGGTATATTT

>J1T_79
GGGTGAGAGAGAGGTGTAGTTTCGTAATTCTTGTTTAGGAAGTTATATTTTTAGTTATAA
ATTTAAGTGTAGGTATATATATATATACATACATATACATATATATATACACATATATGT
ATATATATATATATTTCGATTTTATTAATTGTAAGAGATTTGAGTAAAGAAATTAAGATT
TAAGGGGCGTTGATATTTTGTTAGTTTTTTAGGTTGTTTTAAAATTTGAGGTATATGTTT
TTATTTTGGTATGTTTTTTTTGTTTTAGTTGGAATTTTTTGATTCGTGTGTATTTAGTAT
ATAGTGAATATTTAAAGTTTTGTGTTTTGATAATTTAAAGATTAGATTAAATTTTTTGTA
TTTATTTAAATTTATATATAGGTTTGAAGGGTAGGTATATTT
>J1T_81
GGGTTTTGTGGTTTTTGAAGTTTAGATGTTTTTTTTTTTATGTTGAGGTTAGGATATTTT
TGGGTGAGAGAGAGGTGTAGTTTTGTAATTATTGTTTAGGAAGTTATATTTTCAGTTATA
AATTTAAGTTTAGGTATATACATATATACACATATATATATATATATATATATATACGTA
TATATATATATATTTCGATTTTATTAATTGTAAGAGATTTGAGTAAAGAAATTAAGATTT
AAGGGGTGTTGATATTTTGTTAGTTTTTTAGGTTGTTTTAAAATTTGAGGTATATGTTTT
TATTTTGGTATGTTTTTTTTGTTTTAGTTGGAATTTTTTGATTTGTGTGTATTTAGTATA
TAGTGAATACTTAAAGTTTTGTGTTTTGATAATTTAAAGATTAGATTAAATTTTTTGTAT
TTATTTAAATTTATATATAGGTTTGAAGGGTAGG
>J1T_82
AAGATTGGGATTTTAGAAATTTGATTGGTAAAGTTTTTATGTTGGTTATTTTTATTTTTC
TTTTGTTTGGGTTTGTGGTTTTTGAAGTTTAGATGTTTTTTTTTTATGTTGAGGTTAGGA
TATTTTTGGGTGAGAGAGAGGTGTAGTTTTGTAATTATTGTTTAGGAAGTTATATTTTTA
GTTATAAATTTAAGTTTAGGTATATGTATACATATATATATATATATATATATATGTACA
TATGTATATATATATATGTTTTGATTTTATTAATTGTAAGAGATTTGAGTAAAGAAATTA
AGATTTAAGGGGTGTTGATATTTTGTTAGTTTTTTAGGTTGTTTTAAAATTTGAGGTATA
TGTTTTTATTTTGGTATGTTTTTTTTGTTTTAGTTGGAATTTTTTGATTTGTGTGTATTT
AGTATATAGTGAATATTTAAAGTTTTGTGTTTTGATAATTTAAAGATTAGATTAAATTTT
TTGTATTTATTTAAATCTATATATAGGTTTGAAGGGTAGGTATATTT
>J1T_83
AAGATTGGGATTTTAGAAATTTGATTGGTAAAGTTTTTATGTTGGTTATTTTTATTTTTT
TTTTGTTTGGGTTTGTGGTTTTTGAAGTTTAGATGTTTTTTTTTTATGTTGAGGTTAGGA
TATTTTTGGGTGAGAGAGAGGTGTAGTTTTGTAATTATTGTTTAGGAAGTTATATTTTTA
GTTATAAATTTAAGTTTAGGTATATGTATACATATATATATATATATATATATATGTACA
TATGTATATATATATATGTTTTGATTTTATTAATTGTAAGAGATTTGAGTAAAGAAATTA
AGATTTAAGGGGTGTTGATATTTTGTTAGTTTTTTAGGTTGTTTTAAAATTTGAGGTATA
TGTTTTTATTTTGGTATGTTTTTTTTGTTTTAGTTGGAATTTTTTGATTTGTGTGTATTT
AGTATATAGTGAATATTTAAAGTTTTGTGTTTTGATAATTTAAAGATT
>J1T_84
TAGGATATTCTTGGGTGAGAGAGAGGTGTAGTTTTGTAATTACTGTTTAGGAAGTTATAT
TTTTAGTTATAAATTTAAGTTTAGGTATATATATATATATATATACATACATACATATAT
ACATATATATGTATATATATATATATTTTGATTTTATTAATTGTAAGAGATTTGAGTAAA
GAAATTAAGATTTAAGGGGTGTTGATATTTTGTTAGTTTTTTAGGTTGTTTTAAAATTTG
AGGTATATGTTTTTATTTTGGTATGTTTTTTTCGTTTTAGTTGGAATTTTTTGATTTGTG
TGTATTTAGTATATAGTGAATATTTAAAGTTTTGTGTTTTGATAATTTAAAGATTAGATT
AAATTTTTTGTATTTATTTAAATTTATATATAGGTTTGAAGGGTAGG
>J1T_85
AAGATTGGGATTTTAGAAATTTGATTGGTAAAGTTTTTATGTTGGTTATTTTTATTTTTT
TTTNGTTCGGGTTNGTGGTTTTTGAAGTTTAGATGTTTTTTTTTTTATGTTGAGGTTAGG
ATATTTTTGGGTGAGAGAGAGGTGTAGTTTTGTAATCATTGTTTAGGAAGTTATATTTTT
AGTTATAAATTTAAGTTTAGGTATATATACACATACACATATATACATATATATACACAT
ATACATGTATATATATATATATTTTGATTTTATTAATTGTAAGAGATTTGAGTAAGGAAA
TTAAGATTTAAGGGGCGTTGATATTTTGTTAGTTTTTTAGGTTGTTTTAAAATTTGAGGT
ATATGTTTTTATTTTGGTATGTTTTTTTTGTTTTAGTTGGAATTTTTTGATTTGTGTGTA
TTTAGTATATAGTGAATATTTAAAGTTTTGTGTTTTGATAATTTAAAGATTAGATTAAAT
TTTTTGTATTTATTTAAATTTATATATAGGTTTGAAGGGTAGGT
>J1T_86
GGGTTGGTGGTTTTTGAAGTTTAGATGTTTTTTTTTTTATGTTGAGGTTAGGATATTTTT
GGGTGAGAGAGAGGTGTAGTTTTGTAATCATTGTTTAGGAAGTTATATTTTTAGTTATAA
ATTTAAGTTTAGGTATATATACACATACACATATATACATATATATACACATATACATGT
ATATATATATATATTTTGATTTTATTAATTGTAAGAGATTTGAGTAAAGAAATTAAGATT
TAAGGGGCGTTGATATTTTGTTAGTTTTTTAGGTTGTTTTAAAATTTGAGGTATATGTTT
TTATTTTGGTATGTTTTTTTTGTTTTAGTTGGAATTTTTTGATTTGTGTGTATTTAGTAT
ATAGTGAATATTTAAAGTTTTGTGTTTTGATAATTTAAAGATTAGATTAAATTTTTTGTA
TTTATTTAAATTTATATATAGGTTTGAAGGGTAGGT
>J1T_87
GATTTTAGAAATTTGGATTGGTAAAGTTTTTATGTTGGTTATTTTTATTTTTTTTTTGTT
TGGGTTTGTGGTTTTTGAAGTTTAGATGTTTTTTTTTTTTATGTTGAGGTTAGGATATTT
TTGGGTGAGAGAGAGGTGTAGTTTTGTAATTATTGTTTAGGAAGTTATATTTTTAGTTAT
AAATTTAAGTTTAGGTATATATATATATATATACATACATACATATATACATATATATGT
ATATATATATATTTTGATTTTATTAATTGTAAGAGATTTGAGTAAAGAAATTAAGATTTA
AGGGGTGTTGATATTTTGTTAGTTTTTTAGGTTGTTTTAAAATTTGAGGTATATGTTTTT
ATTTTGGTATGTTTTTTTTGTTTTAGTTGGAATTTTTTGATTTGTGTGTATTTAGTATAT
AGTGAATATTTAAAGTTTTGTGTTTTGATAATTTAAAGATTAGATTAAATTTTTTG
>J1T_94
AAGATTGGGATTTTAGAAATTTGATTGGTAAAGTTTTTATGTTGGTTATTTTTTATTTTT
TTTTGTTTGGGTTTGTGGTTTTTGAAGTTTAGATGTTTTTTTTTTTTATGTTGAGGTTAG
GATATTTTTTGGGTGAGAGAGAGGTGTAGTTTTGTAATTATTGTCTAGGAAGTTATATTT
TTAGTTATAAATTTAAGTTTAGGTATATATATATATATATACATATACATACATATATAT
ATGTATATATATATATATTTTGATTTTATTAATTGTAAGAGATTTGAGTAAAGAAATTAA
GATTTAAGGGGTGTTGATATTTTGTTAGTTTTTTAGGTTGTTTTAAAATTTGAGGTATAT
GTTTTTATTTTGGTATGTTTTTTTTGTTTTAGTTGGAATTTTTTGATTTGTGTGTATTTA
GTATATAGTGAATATTTAAAGTTTTGTGTTTTGATAATTTAAAGATTAGATTAAATTTTT
TGTATTTATTTAAATTTATATATAGGTTTGAAGGGTAGG
>J1T_95
TTAGGATATTTTTGGGTGAGAGAGAGGTGTAGTTTTGTAATTACTGTTTAGGAAGTTATA
TTCTTTAGTTATAAATTTAAGTTTAGGTATATATATATATATATATACATATACATATAT
ATATATATACGTATATATATATATATTTTGATTTTATTAATTGTAAGAGATTTGAGTAAA
GAAATTAAGATTTAAGGGGCGTTGATATCTTGTTAGTTTTTTAGGTTGTTTTAAAATTTG
AGGTATATGTTTTTATTTTGGTATGTTTTTTTTGTTTTAGTTGGAATTTTTTGATTTGTG
TGTATTTAGTATATAGTGAATATTTAAAGTTTTGTGTTTTGATAATTTAAAGATTAGATT
AAATTTTTTGTATTTATTTAAATTTATATATAGGTTTGAAGGGTAGGTATATTT

VA011
>J1T_96
AAGATTGGGATTTTAGAAATTTGATTTGGTAAAGTTTTCTATGTTGGTTATTTTTATTTT
TTTTTGTTTGGGTTTGTGGTTTTTGAAGTTTAGATGTTCTTTTTTTTATGTTGAGGTTAG
GATATTTTTGGGCGAGAGAGAGGTGTAGTTTTGTAATTATTGTTTAGGAAGTATTATTTT
TAGTTATAAATTTAAGTTTAGGTATATATATATACGTATATACATATATATATATATGTA
TATATACGTATATATATATATATTTCGATTTTATTAATTGTAAGAGATTTGAGTAAAGAA
ATCAAGATTTAAGGGGCGTTGATATTTTGTTAGTTTTTTAGGTTGTTTTAAAATTTGAGG
TATATGTTTTTATTTCGGTATGTTTTTTTTGTTTTAGTTGGAATTTTTTGATTTGTGTGT
ATTTAGTATATAGTGAATATTTAAAGTTTTGTGTTTTGATAATTTAAAGATTAGATTAAA
TTTTTTGTATTTATTTAAATTTATATATAGGTTTGAAGGGTAGG
>J1T_39
AAGATTGGGATTTTTAGAAATTTGATTGGGTAAAGTTATTTTATGTTGGGCGATTTGTTA
ATTTTTGCGTTGCCGTGGGCGTCGTGGTTATTGAAGTTTAAGATGCTTTTTTCTTTTATG
CTGAGGTTAGGATATTTTTGGGTGAGAGAGAGGTGTAGTTTATGTAATTATTGTTTAGGA
AGTTATATTTTTAGTTATAAATTTAAGTTTAGGTATATATACATATATACATATATATAT
ATATATACATATATATATGTATATATATATATATTTTGATTTTATTAATTGTAAGAGATT
TGAGTAAAGAAATTAAGATTTAAGGGGCGTTGATATTTTGTTAGTTTTTTAGGTTGTTTT
AAAATTTGAGGTATATGTTTTTATTTTGGTATGTTTTTTTTGTTTTAGTTGGAATTTTTT
GATTTGTGTGTATTTAGTATATGGTGAATATTTAAAGTTTTGTGTTTTGATAATTTAAAG
ATTAGATTAAATTTTTTGTATTTATTTAAATTTATATATAGGTTTGAAGGGTAGGTATAT
TT
