# Supplementary material for: Epigenetic interplay between mouse endogenous retroviruses and host genes
Source: Genome Biol. 2012 Oct 3;13(10):R89. doi: 10.1186/gb-2012-13-10-r89 (PMC3491417; doi:10.1186/gb-2012-13-10-r89)
Supplement: Additional file 4 — All bisulfite sequencing data. Compilation of all bisulfite sequences. [file gb-2012-13-10-r89-S4.zip › IAP6428_fullsite_ES.rtf]

12 21 10
CdGAP Trans
B6 ES Transition Region 
Miniprep Sequences
>B6T_78
AAGATTGGGATTTTAGAAATTTGATTGGTAAAGTTTTTATGTTGGTTATTTTTATTTTTT
TTTGTTTGGGTTTGTGGTTTTTGAAGTTTAGATGTTTTTTTTTTATGTTGAGGTTAGGAT
ATTTTTGGGCGAGAGAGAGGTGTAGTTTTGTAATTATTGTTTAGGAAGAAGTTATATTTT
TAGTTATAAATTTAAGTTTAGGTATATATATATATATACATACACATATATATATATACA
TATACGTACATATATATTTCGATTTTATTAATTGTAAAAGATTTGAGTAAAGAAATTAAG
ATTTAAGGGGCGTTGATATTTTGTTAGTTTTTCAGGTTGTTTTAAAATTTGAGGCGTATG
TTTTTATTTCGGTATGTTTTTTTTGTTTTAGTTGGAATTTTTTGATTCGTGTGTATTCAG
CATATAGTGAACATTTAAAGTTTTGTGTTTTGATAATTTAAAGATTAGATTAAATTTTTT
GTATTTATTTAAATTTATATATAGGTTTGAAGGGTAGGTATATTT
>B6T_79
AAGATTGGGATTTTAGAAATTTGATTGGTAAAGGTTTTATGTTGGTTATTTTAATTTTTT
TTCGTTTGGGTCTGTGGTTTTTGAAGCTTAGATGTTTTTTTTTTTATGTGAGGTTAGGAT
ATTTTTGGGCGAGAGAGAGGTGTAGTTTTGTAATTATTGTTTAGGAAGAAGTTATATTTT
TAGTTATAAATATAAGTTTAGGTATATATATATATATACATATATATATATATATATATA
TATGTATATATATATTTCGATTTTATTAATTGTAAGAGATTTGAGTAAAGAAATTAAGAT
TTAAGGGGCGTTGATATTTTGTTAGTTTTTTAGGTTGTTTTAAAATTTGAGGCGTATGTT
TTTATTTCGGTATGTTTTTTTTGTTTTAGTTGGAATTTTTTGATTCGTGTGTATTTAGTA
TATAGTGAATATTTAAAGTTTTGTGTTTTGATAATTTAAAGATTAGATTAAATTTTTTGT
ATTTATTTAAATTTATATATAGGTTTGAAGGGTAGGTATATTT
>B6T_80
TAGGATATTTTTGGGTGAGAGAGAGGCGCAGTTTTGTAATTATTGCTTAGGAAGAAGTTA
TATTTTTAGTTATAAATTTAAGTTTAGGTATATATATATATATATACATATACATACATA
TATATACATATACGCATATATATATTTCGATTTTATTAATTGCAAGAGATTTGAGTAAAG
AAATTAAGATTTAAGGGGCGTTGATATTTTGTTAGTTTTTTAGGTTGTTTTAAAATTTGA
GGCGTATGTTTTTATCTCGGTATGTTTTTTTTGTTTTAGTTGGAATTTTTTGATTCGTGT
GTATTTAGTATATAGTGAATATTTAAAGTTTTGTGTTTTGATAATTTAAAGATTAGATTA
AATTTTTTGTATTTATTTAAATTTATATATAGGTTTGAAGGGTAGGTATATTT
>B6T_81
GAAGTTTAGATGTTTTTGTTGTTAAGGTGAGGTTAGGATAATTTTGGGTGAGAGAGAGGT
GTAGTTTTGTAATTATTGTTTAGGAAGAAGTTATATTTTTAGTTACAAACTTAAGTTTAG
GTACACACACATATACATACATATATATATATATACACACACGTACACACACATTTCGAT
CTTACTAACTGTAAGAGATTTGAGTAAAGAAACCAAGATTTAAGGGGCGTTGACATTTTG
TTAGTTTTTTAGGTTGTTTTAAAATTTGAGGCGTATGTTTTTATTTTGGTATGTTTTTTT
TGTTTTAGTTGGAATTTCTTGATTCGTGTGTATTTAGTATATAGTGAACATTTAAAGTTT
TGTGTTTTGATAACTTAAAGATTAGATTAAATTTTTTGTATCCATTTAAATTTATATATA
GGTTTGAAGGGTAGGTAT
>B6T_82
GAGGTTAGGATATTTTTGGGTGAGAGAGACGCGCACTCCTGTAATTATTGTTTAGGAAGA
AGTTATATTTTTAGTTATAAATATAAGTTTAGGTATATACATATATATATATATATATAT
ATATATATATATACGTATATATATATTTCGATTTTATTAATTGTAAGAGATTTGAGTAAA
GAAATTAAGATTTAAGGGGCGTTGATATTTTGTTAGTTTTTTAGGTTGTTTTAAAATTTG
AGGCGTATGTTTTTATTTCGGTATGTTTTTTTTGTTTTAGTTGGAATTTTTTGATTCGTG
TGTATTTAGTATATAGTGAACATTTAAAGTTTTGTGTCTTGATAATTTAAAGATTAGATT
AAATTTTTTGTATTTATTTAAATTTATATATAGGTTTGAAGGGTAGGTATATTT
>B6T_83
AAGATTGGGATTTTAGAAATTTTGATTGGTAAAGTTTTTATGTTGGTTTATTTTTATTTT
TTTTTGTTTGGGTTTGTGGTTTGTGAAGTTTAGATGTTTTTTTTTTTATGTTGAGGTTAG
GATATTTTTGGGTGAGAGAGAGGTGTAGTTTTGTAATTATTGTCTAGGAAGAAGTTATAT
TTTTAGTTACAAACTTAAGTTTAGGTACACACACATATACATACATATATATATATATAC
ACACACGCACACACACATTTCGATCTTACTAACTGTAAGAGATTTGAGTAAAGAAACCAA
GATTTAAGGGGCGTTGACATTTTGTTAGTTTTTTAGGTTGTTTTAAAATTTGAGGCGTAT
GTTTTTATTTTGGTATGTTTTTTTTGTTTTAGTTGGAATTTCTTGATTCGTGTGTATTTA
GTATATAGTGAACATTTAAAGTTTTGTGTTTTGATAACTTAAAGATTAGATTAAATTTTT
TGTATCCATTTAAATTTATATATAGGTTTGAAGGGTAGGTATATTT
>B6T_84
AAGATTGGGATTTTAGAAATTTGATTGGTAAAGTGTTTATGTTGGTTATTTATTATTTTT
TTTTGTTTGGGTTCGTGGTTTTTGAAGTTTAGATGTTTTTTTTTTTATGTTGAGGTTAGG
ATATTTTTGGGTGAGAGAGAGGTGCAGTTTTGTAATTATTGTTTAGGAAGAAGTTATATT
TTTAGTTATAAATTTAAGTTTAGGTATATATATATACATATACATATACATATATATACA
TATACGCATATATATATTTCGATTTTATTAATTGTAAGAGATTTGAGTAAAGAAATTAAG
ATTTAAGGGGCGTTGATATTTTGTTAGTTTTTTAGGTTGTTTTAAAATTTGAGGCGTATG
TTTTTATTTCGGTATGTTTTTTTTGTTTTAGTTGGAATTTTTTGATTTGTGTGTATTTAG
TACATAGTGAATATTTAAAGTTTTGTGTTTTGATAATTTAAAGATTAGATTAAATTTTTT
GTATTTATTTAAATTTATATATAGGTTTGAAGGGTAGGTATATTT
>B6T_85
TATTTTTGGTGAGAGAGAGGTGTAGTTTTGTAATTATTGTCTAGGAAGAAGTTATATTTT
TAGTTACAAACTTAAGTTTAGGTACACACACATATACATACATATATATATATATACACA
CACGTACACACACATTTCGATCTTACTAACTGTAAGAGATTTGAGTAAAGAAACCAAGAT
TTAAGGGGCGTTGACATTTTGTTAGTTTTTTAGGTTGTTTTAAAATTTGAGGCGTATGTT
TTTATTTTGGTATGTTTTTTTTGTTTTAGTTGGAATTTCTTGATTCGTGTGTATTTAGTA
TATAGTGAACATTTAAAGTTTTGTGTTTTGATAACTTAAAGATTAGATTAAATTTTTTGT
ATCCATTTAAATTTATATATAGGTTTGAAGGGTAGGTATATTT
>B6T_86
AAGATTGGGATTTTAGAAATTTGATTGGTAAAGTTTTTATGTTGGTTATTTTTATTTTTT
TTGTTTGGGTTTGTGGTTTTTGAAGTTTAGATGTTTTTTTTTTTATGTTGAGGTTAGGAT
ATTTTTGGGTGAGAGAGAGGTGTAGTTTTGTAATTATTGTTTAGGAAGAAGTTATATTTT
TAGTTATAAATTTAAGTTTAGGTATATATACATACATACATATATATATATATATATATA
CGTATATATGTATTTCGATTTTATTAATTGTAAGAGATTTGAGTAAAGAAATTAAGATTT
AAGGGGCGTTGATATTTTGTTAGTTTTTTAGGTTGTTTTAAAATTTGAGGCGTATGTTTT
TATTTCGGTATGTTTTTTTTGTTTTAGTTGGAATTTTTTGATTCGTGTGTATTTAGTATA
TAGTGAATATTTAAAGTTTTGTGTTTTGATAATTTAAAGATTAGATTAAATTTTTTGTAT
TTATTTAAATTTATATATAGGTTTGAAGGGTAGGTATATTT

Plate B

>B6T_67
AAGATTGGGATTTTAGAAATTTGATTGGTAAAGGTTTTTTATGTTGGTTATTTTTTATTT
TTTTTCGTTTGGGTTTGCGGTTTTTGAAGGTTTAGATGTTTTTTTTTTTATGTTGAGGTT
AGGATATTTTTTGGGTGAGAGAGAGGTGTAGTTTTGTAATTATTGTTTAGGAAGAAGTTA
TATTTTTAGTTATAAATTTAAGTTTAGGTATATATATATACATATATATACACATATATA
TATACGTATATATATATTTTGATTTTATTAATTGTAAGAGATTTGAGTAAAGAAATTAAG
ATTTAAGGGGCGTTGATATTTTGTTAGTTTTTTAGGTTGTTTTAAAATTTGAGGCGTATG
TTTTCATTTCGGTATGTTTTTTTTGTTTTAGTTGGAATTTTTTGATTCGTGTGTATTTAG
TATATAGTGAATATTTAAAGTTTTGTGTTTTGATAATTTAAAGATTAGATTAAATTTTTT
GTATTTATTTAAATTTATATATAGGTCGAAGGGTAGGTATATTT
>B6T_68
AAGATTGGGATTTTAGAAATTTGAATTGGTAAAGTTTTTATGTTGGTTATTTTTATTTTT
TTTTGTTTGGGTTTGTGGTTTTTGAAGTTTAGACGTTTTTTTTTTTATGTTGAGGTTAGG
ATATTTTTGGGTGAGAGAGAGGTGTAGTTTTGTAATTATTGTTTAGGAAGAAGTTATATT
TTTAGTTACAAACTTAAGTTTAGGTACACACACATATACATACATATATATATATATACA
CACACGTACACACACATTTCGATCTTACTAACTGTAAGAGATTTGAGTAAAGAAACCAAG
ATTTAAGGGGCGTTGACATTTTGTTAGTTTTTTAGGTTGTTTTAAAATTTGAGGCGTATG
TTTTTATTTTGGTATGTTTTTTTTGTTTTAGTTGGAATTTCTTGATTCGTGTGTATTTAG
TATATAGTGAACATTTAAAGTTTTGTGTTTTGATAACTTAAAGATTAGATTAAATTTTTT
GTATCCATTTAAATTT
>B6T_70
GAAGTTTAGATGTTTTTTTTTTATGTTGAGGTTAGGATATTTTTGGGTGAGAGAGAGGTG
TAGTTTTTTGTAATTATTGTTTAGGAAGAAGTTTTATTTTTAGTTATAAATTTAAGCTTA
GGTATATATATATATACATACATATACATACATATATACATACGTATACATATATTTCGA
TTTTATTAATTGTAAGAGATTTGAGTAAAGAAATTAAGATTTAAGGGGCGTTGATATTTT
GTTAGTTTTTTAGGTTGTTTTAAAATTTGAGGCGTATGTTTTTATTTCGGTATGTTTTTT
TTGTTTTAGTTGGAATTTTTTGATTCGTGTGTATTTAGTATATAGTGAATATTTAAAGTT
TTGTGTTTTGATAATTTAAAGATTACGATTAAATTTTTTGCATTTATTTAAATTTATATA
TAGGTTTGAAGGGTAGGTATATTT
>B6T_71
TAGATGTTTTTTTTTTCCTATGTTGAGGTAGGATATTTTTGGGTCAGAGAGAGGTGTAGT
TTTGTAATTATTGTCTAGGAAGAAGTTATATTTTTAGTTATAAATTTAAGTTTAGGTATA
TATATATATATATATATATATACATATATATATACGTATATATATATTTCGATTTTATTA
ATTGTAAGAGATTTGAGTAAAGAAATTAAGATTTAAGGGGCGTTGATATTTTGTTAGTTT
TTTAGGTTGTTTTAAAATTTGAGGCGTATGTTTTTATTTTGGTATGTTTTTTTTGTTTTA
GTTGGAATTTTTTGATTTGTGTGTATTTAGTATACAGTGAATATTTAAAGTTTTGTGTTT
TGATAATTTAAAGATTAGATTAAATTTTTTGCATTTATTTAAATTTATATATAGGTTTGA
AGGGTAGGTATATTT
>B6T_72
AAGATTGGGATTTTAGAAATTTGATTGGTAAAGTTTTTATGTTGGTTATTTTTATCTTTT
TTTGTTTGGGTTTGTGGTTTTTGAAGTTTAGATGTTTTTTTTTTATGTTGAGGTTAGGAT
ATTTTTGGGTGAGAGAGAGGTGTAGTTTTGTAATTATTGTTTAGGAAGAAGTTATATTTT
TAGTTATAAATTTAAGTTTAGGTATATATATATATATATATACATATATATATATATATA
TACGTATATATATATTTCGATTTTATTAATTGTAAGAGATTTGAGTAAAGAAATTAAGAT
TTAAGGGGCGTTGATATTTTGTTAGTTTTTTAGGTTGTTTTAAAATTTGAGGCGTATGTT
TTTATTTCGGTATGTTTTTTTTGTTTTAGTTGGAATTTTTTGATTCGTGTGTATTTAGTA
TATAGTGAATATTTAAAGTTTTGTGTTTTGATAATTTAAAGATTAGATTAAATTTTTTGT
ATTTATTTAAATTTATATATAGGTTTGAAGGGTAGGTATATTT
>B6T_73
AAGATTGGGATTTTAGAAATTTGATTGGTAAAGTTTTTATGTTGGTTATTTTTATCTTTT
TTTGTTTGGGTTTGTGGTTTTTGAAGTTTAGATGTTTTTTTTTTATGTTGAGGTTAGGAT
ATTTTTGGGTGAGAGAGAGGTGTAGTTTTGTAATTATTGTTTAGGAAGAAGTTATATTTT
TAGTTATAAATTTAAGTTTAGGTATATATATATATATATATATATACATATATACATATA
CGCATATATATATTTCGATTTTATTAATTGTAAGAGATTTGAGTAAAGAAATTAAGATTT
AAGGGGCGCTGATATTTTGTTAGTTTTTTAGGTTGTTTTAAAATTTGAGGCGTATGTTTT
TATTTCGGTATGTTTTTTTTGTTTTAGTTGGAATTTTTTGATTCGTGTGTATTTAGTATA
CAGTGAATATTTAAAGTTTTGTGTTTTGATAATTTAAAGATTAGATTAAATTTTTTGCAT
TTATTTAAATTTATATATAGGTTTGAAGGGTAGGTATATTT
>B6T_90
AAGATTGGGATTTTAGAAATTTGATTGGTAAAGTTTTTATGGTGGTTATTTTTATTTTTT
TTGGTTTGGGTTTGTGGTTTTTGAAGTTTAGATGTTTTTTTTTTATGTTGAGGTTAGGAT
ATTTTTGGGTGAGAGAGAGGTGTAGTTTTGTAATTATTGTTTAGGAAGAAGTTATATTTT
TAGTTACAAATTTAAGTTTAGGTACATATATATACATACACACACACATATACACACACA
CACGTATATATATATTTCGATTTTATTAATTGTAAGAGATTTGAGTAAAGAAATTAAGAT
TTAAGGGGCGTTGATATTTTGTTAGTTTTTTAGGTTGTTTTAAAATTTGAGGCGTATGTT
TTTATTTCGGTATGTTTTTTTTGTTTTAGTTGGAATTTTTTGATTCGTGTGTATTTAGTA
CATAGTGAACTATTTAAAGTTTTGTGTTTTGATAATTCAAAGATTAGATTAAATTTTTTG
TATTCATTTAAATTTATATATAGGTTTGAAGGGTAGGTATATTT
>B6T_92
AAGATTGGGATTTTAGAAATTTGATTGGTAAAGTTTTTATGTTGGTTATTTTTATTTTTT
TTTGTTTGGGTTTGTGGTTTTTGAAGTTTAGATGTTTTTTTTTTTATGTTGAGGTTAGGA
TATTTTTGGGTGAGAGAGAGGTGTAGTTTTGTAATTATTGTTTAGGAAGAAGTTATATTT
TTAGTTATAAATTTAAGTTTAGGTACATATACATATATATATATATATATATACATATAT
ATACGTATATATATATTTTGATTTTATTAATTGCAAGAGATTTGAGTAAAGAAATTAAGA
TTTAAGGGGTGTTGATATTTTGTTAGTTTTTTAGGTTGTTTTAAAATTTGAGGCGTATGT
TTTTATTTCGGTATGTTTTTTTTGTTTTAGTTGGAATTTTTTGATTCGTGTGTATTTAGT
ATATAGTGAATATTTAAAGTTTTGTGTTTTGATAATTTAAAGATTAGATTAAATTTTTTG
TATTTATTTAAATTTATATATAGGTTTGAAGGGTAGGTATATTT
>B6T_93
AAGGATTGGGATTTTAGAATTTTGATTGGTAAAGTTTTTACGTTGGTTATTTTTATTTTT
TTTTGTTTGGGTTTGTGGTTTTTTGAAGTTTAGATGTTTTTTTTTTATGTTGAGGTTAGG
ATATTTTTGGGTGAGAGAGAGGTGTAGTTCTGTAATTATTGTTTAGGAAGAAGTTATATT
TTTAGTTATAAATTTAAGTTCAGGTATATATATATATATACATATATATACATATATATA
TACACGTATATATATATTCCGATTTTATTAATTGTAAGAGATTTGAGTAAAGAAATTAAG
ATTTAAGGGGCGTTGATATTTTGTTAGTTTTTTAGGTTGTTTTAAAATTTGAGGCGTATG
TTTTTATTTTGGTATGTTTTTTTTGTTTTAGTTGGAATTTTTTGATTCGTGTGTATTTAG
TATATAGTGAATATTTAAAGTTTTGTGTTTTGATAATTTAAAGATTAGATTAAATTTTTT
GTATTTATTTAAATTTATATAT
>B6T_91
AAGATTGGGATTTTAGAAATTTGATTGGTAAAGTTTTTATGTTGGTTATATTTTATTTTT
TTTTGTTTGGGTTTGTGGTTTTTGAAGTTTAGATGTTTTTTTATTTTATGTTGAGGCTAG
GATATTTTTGGGTGAGAGAGAGGTGTAGTTTTGTAATTATTGTTTAGGAAGAAGTTATAT
TTTTAGTTATAAATTTAAGTTTAGGTATATATATATATATATATATATATATATATATAT
ATATACGTATATATATATTTCGATTTTACTAATTGTAAGAGATTTGAGTAAAGAAATTAA
GATTTAAGGGGCGTTGATATTTTGTTAGTTTTTTAGGTTGTTTTAAAATTTGAGGCGTAT
GTTTTCATTTCGGTATGTTTTTTTTGTTTTAGTTGGAATTTTTTGATTCGTGTGTATTTA
GTATATAGTGAATATTTAAAGTTTTGTGTTTTGATAATTTAAAGATTAGATTAA

VA011
>B6T_17
CGGTTTTTTGAAGGTTTAGATGTTTTTTTTTTTGAAGTTGAGGTTAGGATATTTTTGGGT
GAGAGAGAGGTGTAGTTTTTGTAATTATTGTTTAGGAAGAAGTTTATATTTTTAGTTATA
AATTTAGTTTTAGGTCGATATATACATACATATATATATATATATATATAGCGTATATAT
ATATTTCGATTTTATTAATTGTAAGAGATTTGAGTAAAGAAATTAAGATTTAAGGGGCGT
TGATATTTTGTTAGTTTTTTAGGTTGTTTTAAAATTTGAGGCGTATGTTTTTATTTCGGT
ATGTTTTTTTTGTTTTAGTTGGAATTTTTTGATTCGTGTGTATTTAGTATATAGTGAATA
TTTAAAGTTTTGTGTCTTGATAATTTAAAGATTAGATTAAATTTTTTGTATTTATTTAAA
TTTATATATAGGTTTGAAGGGTAGGTATATTT
>B6T_19
AAGATTGGGATTTTAGGAAATTTGATTGGTAAAGTTTTTACGTTGGTTATTTTTATTTTT
TTTTGTTTGGGTTTGTGGTTTTTGAAGTTTAGATGTTTTTTTTTTTACGTTGAGGTTAGG
ATATTTTCGGGTGAGAGAGAGGTGTAGTTTTGTAATTATTGTTTAGGAAGAAGTTATATT
TTTAGTTATAAATTTAAGCTTAGGTATATATATATATATATACATATATATATATATACA
CATACGTATATATATATTTCGATTTTATTAATTGTAAGAGATTTGAGTAAAGAAATTAAG
ATTTAAGGGGCGTTGATATTTTGTTAGTTTTTTAGGTTGTTTTAAAATTTGAGGCGTATG
TTTTTATTTTGGTATGTTTTTTTTGTTTTAGTTGGAATTTTTTGATTCGTGTGTATTTAG
TATATAGTGAACATTTAAAGTTTTGTGTTTTGATAATTTAAAGATTAGATTAAATTTTTT
GTATTTATTTAAATTTATATATAGGTTTGAAGGGTAGGTATATTT
