# Supplementary material for: Epigenetic interplay between mouse endogenous retroviruses and host genes
Source: Genome Biol. 2012 Oct 3;13(10):R89. doi: 10.1186/gb-2012-13-10-r89 (PMC3491417; doi:10.1186/gb-2012-13-10-r89)
Supplement: Additional file 4 — All bisulfite sequencing data. Compilation of all bisulfite sequences. [file gb-2012-13-10-r89-S4.zip › IAP6428_gene_ES.rtf]

6/14/10
CdGAP CpG Island Promtoer B6129 ES Cells Miniprep Sequences
B6 Clones
>GAPCpGES_64
GGGGTTGGTTATTAGGGTAGAGTGGTGTTGTGGGTTTGTATGGGTGTGTAGATTGGGTTG
TGTATTGTTTTGGGTGGGTGTTATTTTTTTATTTGGGTTCTGGTGGTGTTTTTTTGTGTT
TTTTGGTTGAGTTTTGTGAGGAGTTGGGGTTGGGGTGTGGTGGTTAGGGGATTGTTTTGG
GGTTGAGTGTGTTTTGTTTTAGGTTTGTGTTTTGTTTTTAGAGTGTTGGAGGATTGTGAA
TAGTTAGTATTATGTTTTTTTGTTTGTTTTTGTGTTGTTTTTTTTTTTTGTTTTATAAAT
TTTAGGATTGTTTAGTTTAGGAAGTGTTGGAAGGAAGTTAGTTTTGGAGGGGGGTGGAGG
TTGGAAATTTTTGGTAGTTGTGTGTGTGTGTGTGTGTGTGTGTGTGTGTTTGTGAGTGTA
GTTGAAGGAAGTAAGTGGGTGGGTTGAGTTGTGTGGGTTGGGGGAGAGATTGTGTTTTTT
TGAAGTGGTTTTGTTAGTTTAGAGTATTGTTTAGGGTTGTTTTGGAGGAAATTGGTTTTG
TGAGGAGTTGTGGTTGTTTGTTTGTTTTTTGTTGTTAGTTAGGTTTTTTGTATAGTGTAG
GAGGTGGTGTTGGATTTTGTAGGGAGGGTTTGGGATGGGTGTTTATGGAATTGTTTTTTT
TGT
>GAPCpGES_69
GTTTGGTATTTTTGTTTTTTATGAGTTAGTTTTATAGAGGTTGTTTTTTTGTTTGTGTTT
TTTGTTTTGTATTGTTGATGAGGGTTTTGGGGTTGGGTTATTAGGGTAGAGCGGTGTTGT
GGGTTTGTATGGGTGTGTAGATTGGGCTGTGTATTGTTTTGGGTGGGTGTTATTTTTTAT
TTGGGTTTTGGTGGTGTTTTTTTGTGTTTTTTGGTTGAGTTTTGTGAGGAGTTGGGGTTG
GGGTGTGGTGGTTAGGGGATTGTTTTGGGGTTGAGTGTGTTTTGTTTTAGGTTTGTGTTT
TGTTTTTAGAGTGTTGGAGGATCGTGAATAGTTAGTATTATGTTTTTTTGTTTGTTTTTG
TGTTGTTTTTTTTTTTTTGTTTTATAAATTTTAGGATTGTTTAGTTTAGGAAGTGTTGGA
AGGAAGTTAGTTTTGGAGGGGGGTGGAGGTTGGAAATTTTTGGTAGTTGTGTGTGTGTGT
GTGTGTGTGTGTGTGTGTTTGTGAGTGTAGTTGAAGGAAGTAAGTGGGTGGGTTGAGTTG
TGTGGGTTGGGGGAGAGATTGTGTTTTTTTGAAGTGGTTTTGTTAGTTTAGAGTATTGTT
TAGGGTTGTTTTGGAGGAAATTGGTTTTGTGAGGAGTTGTGGTTGTTTGTTTGTTTTTTG
TTGTTAGTTAGGTTTTTTGTATAGTGTAGGAGGTGGTGTTGGATTTTGTAGGGAGGGTTT
GGGATGGGTGTTTATGGAATTGTTTTTTTTGT
>GAPCpGES_70
TTTAGTTTTTGTTTGGTATTTTTGTTTTTTATGAGTTAGTTTTATAGAGGTTGTTTTTTT
GTTTGTGTTTTTTGTTTTGTATTGTTGATGAGGGTTTTGGGTTTGGGTTATTAGGGTAGA
GTGGTGTTGTGGGTTTGTATGGGTGCGTAGATTGGGTTGTGTATTGTTTTGGGTGGGTGT
TATTTTTTTATTTGGGTTTTGGTGGTGTTTTTTCGTGTTTTTTGGTTGAGTTTTGTGAGG
AGTTGGGGTCGGGGTGTGGTGGTTAGGGGATTGTTTTGGGGTTGAGTGTGTTTTGTTTTA
GGTTTGTGTTTTGTTTTTAGAGTGTTGGAGGATTGTGAATAGTTAGTATTATGTTTTTTT
GTTTGTTTTTGTGTTGTTTTTTTTTTTTGTTTTATAAATTTTAGGATTGTTTAGTTTAGG
AAGTGTTGGAAGGAAGTTAGTTTTGGAGGGGGGTGGAGGTTGGAAATTTTTGGTAGTTGT
GTGTGTGTGTGTGTGTGTGTGTGTGTTTGTGAGTGTAGTTGAAGGAAGTAAGTGGGTGGG
TTGAGTTGTGTGGGTTGGGGGAGAGATTGTGTTTTTTTGAAGTGGTTTTGTTAGTTTAGA
GTATTGTTTAGGGTTGTTTTGGAGGAAATTGGTTTTGTGAGGAGTTGTGGTTGTTTGTTT
GTTTTTTGTTGTTAGTTAGGTTTTTTGTATAGTGTAGGAGGTGGTGTTGGATTTTGTAGG
GAGGGTTTGGGATGGGTGTTTATGGAATTGTTTTTTTTGT
>GAPCpGES_71
GTTTGGTATTTTTGTTTTTTATGAGTTAGTTTTATAGAGGTTGTTTTTTTGTTTGTGTTT
TTTGTTTTGTATTGTTGATGAGGGTTTTGGGGTTGGGTTATTAGGGTAGAGTGGTGTTGT
GGGTTTGCATGGGTGTGTAGATTGGGTTGTGTATTGTTTTGGGTGGGTGTTATTTTTTTA
TTTGGGTTTTGGTGGTGTTTTTTTGTGTTTTTTGGTTGAGTTTTGTGAGGAGTTGGGGTT
GGGGTGTGGTGGTTAGGGGATTGTTTTGGGGTTGAGTGTGTTTTGTTTTAGGTTTGTGTT
TTGTTTTTAGAGTGTTGGAGGATTGTGAATAGTTAGTATTATGTTTTTTTGTTTGTTTTT
GTGTTGTGTTTTTTTTTTTGTTTTATAAATTTTAGGATTGTTTAGTTTAGGAAGTGTTGG
AAGGAAGTTAGTTTTGGAGGGGGGTGGAGGTTGGAAATTTTTGGTAGTTGTGTGTGTGTG
TGTGTGTGTGTGTGTTTGTGAGTGTAGTTGAAGGAAGTAAGTGGGTGGGTTGAGTTGTGT
GGGTTGGGGGAGAGATTGTGTTTTTTTGAAGTGGTTTTGTTAGTTTAGAGTATTGTTTAG
GGTTGTTTTGGAGGAAATTGGTTTTGTGAGGAGTTGTGGTTGTTTGTTTGTTTTTTGTCG
TTAGTTAGGTTCTTTGTATAGTGTAGGAGGTGGTGTTGGATTTTGTAGGGAGGGTTTGGG
ATGGGTGTTTATGGAATTGTTTTTTTTGT
>GAPCpGES_72
GTTTGGTATTTTTGTTTTTTATGAGTTAGTTTTACAGAGGTTGCTTTTTTGTTTGTGTTT
TTTGTTTTGTATTGTTGATGAGGGTTTTGGGGTTGGTTATTAGGGTAGAGTGGTGTTGTG
GGTTTGTATGGGTGTGTAGATTGGGTTGTGTATTGTTTTGGGTGGGTGTTATTTTTTTAT
TTGGGTTTTGGTGGTGTTTTTTTGTGTTTTTTGGTTGAGTTTTGTGAGGAGTTGGGGTTG
GGGTGTGGTGGTTAGGGGATTGTTTTGGGGTTGAGCGTGTTTTGTTTTAGGTTTGTGTTT
TGTTTTTAGAGTGTTGGAGGACTGTGAATAGTTAGTATCATGTTTTTTTGTTTGTTTTTG
TGTTGTTTTTTTTTTTGTTTTATAAATTTTAGGATTGTTTAGTTTAGGAAGTGTTGGAAG
GAAGTTAGTTTTGGAGGGGGGTGGAGGTTGGAAATTTTTGGTAGTTGTGTGTGTGTGTGT
GTGTGTGTGTGTGTGTTTGTGAGTGTAGTTGAAGGAAGTAAGTGGGTGGGTTGAGTTGTG
TGGGTTGGGGGAGAGATTGTGTTTTTTTGAAGTGGTTTTGTTAGTTTAGAGTATTGTCTA
GGGTTGTTTTGGAGGAAATTGGTTTTGTGAGGAGTTGTGGTTGTTTGTTTGTTTTTTGTT
GTTAGTTAGGTTTTTTGTATAGTGTAGGAGGTGGTGTTGGATTTTGTAGGGAGGGTTTGG
GATGGGTGTTTATGGAATTGTTTTTTTTGT
>GAPCpGES_21
ATTGTTGAGTGTGTTTTGTTTTAGGTTTGTGTTTTGTTTTTAGAGTGTTGGAGGATTGTG
AATAGTTAGTATTATGTTTTTTTGTTTGTTTTTGTGCTGTTTTTTTTTTTTGCTTTATAA
ATTTTAGGACTGTTTAGTTTAGGAAGTGTTGGAAGGAAGTTAGTTTTGGAGGGGGGTGGA
GGTTGGAAATTTTTGGTAGTTGTGTGTGTGTGTGTGTGTGTGTGTGTGTGTTTGTGAGTG
TAGTTGAAGGAAGTAAGTGGGTGGGTTGAGTTGTGCGGGTTGGGGGAGAGATTGTGTTTT
TTTGAAGTGGTTTTGTTAGTTTAGAGTATTGTTTAGGGTTGTTTTGGAGGAAATTGGTTT
TGTGAGGAGTTGCGGTTGTTTGTTTGTTTTTTGTTGTTAGTTAGGTTTTTTGTGTAGTGT
AGGAGGTGGTGTTGGATTTTGTAGGGAGGGTTCGGGATGGGTGTTTATGGAATTGTTTTT
TTTGT
>GAPCpGES_20
ATTGGTTGTTTTTTTGTTTGTGTTTTTTGTTTTGTATTGTTGATGAGGGTTTTGGGGTTG
GGTTATTAGGGTAGAGTGGTGTTGTGGGTTTGTATGGGTGTGTAGATTGGGTTGTGTATT
GTTTTGGGTGGGTGTTATTTTTTTATTTGGGTTTTGGTGGTGTTTTTTTGTGTTTTTTGG
TTGAGTTTTGTGAGGAGTTGGGGTTGGGGTGTGGTGGTTAGGGGATTGTTTTGGGGTTGA
GTGTGTTTTGTTTTAGGTTTGTGTTTTGTTTTTAGAGTGTTGGAGGATTGTGGATAGTTA
GTACTATGTTTTTTTGTTTGTTTTTGTGTTGTTTTTTTTTTTTTGTTTTATAAATTTTAG
GATTGTTTAGTTTAGGAAGTGTTGGAAGGAAGTTAGTTTTGGAGGGGGGTGGAGGTTGGA
AATTTTTGGTAGTTGTGTGTATGTGTGTGTGTGTGTGTGTGTGTGTGTGTGTGTGTGTGT
GTGTGTGTGTTTGTGAGTGTAGTTGAAGGAAGTAAGTGGGTGGGTTGAGTTGTGTGGGTT
GGGGGAGAGATTGTGTTTTTTTGAAGTGGTTTTGTTAGTTTAGAGTATCGTTTAGGGTTG
TTTTGGAGGAAATTGGTTTTATGAGGAGTTGTGGTTGTTTGTTTGTTTTTTGTTGTTAGT
TAGGTTTTTTGCATAGTGTAGGAGGTGGCGTTGGATTTTGTAGGGAGGGTTTGGGATGGG
TGTTTATGGAATTGTTTTTTTTGT
>GAPCpGES_19
GAGGTTGTTTTTTTGTTTGTGTTTTTTGTTTTGTATCGTTGATGAGGGTTTTGGGGTTGG
TTATTAGGGTAGAGTGGTGTTGTGGGTTTGTATGGGTGTGTAGATTGGGTTGTGTATTGT
TTTGGGTGGGTGTTATTTTTTTATTTGGGTTTTGGTGGTGTTTTTTTGTGTTTTTTGGTT
GAGTTTTGTGAGGAGTTGGGGTTGGGGTGCGGTGGTTAGGGGATTGTTTTGGGGTTGAGT
GTGTTTTGTTTTAGGTTTGTGTTTTGTTTTTAGAGTGTTGGAGGATTGTGAATAGTTAGT
ATTATGTTTTTTTGTTTGTTTTTGTGTTGTTTTTTTTTTTTGTTTTATAAATTTTAGGAT
TGTTTAGTTTAGGAAGTGTTGGAAGGAAGTTAGTTTTGGAGGGGGGTGGAGGTTGGAAAT
TTTTGGTAGTTGTGTGTGTGTGTGTGTGTGTGTGTGTGTGTTTGTGAGTGTAGTTGAAGG
AAGTAAGTGGGTGGGTTGAGTTGTGTGGGTTGGGGGAGAGATTGTGTTTTTTTGAAGTGG
TTTTGTTAGTTTAGAGTATTGTTTAGGGTTGTTTTGGAGGAAATTGGTTTTGTGAGGAGT
TGTGGTTGTTTGTTTGTTTTTTGTTGTTAGTTAGGTTTTTTGTATAGTGTAGGAGGTGGT
GTTGGATTTTGTAGGGAGGGTTTGGGATGGGTGTTTATGGAATTGTTTTTTTTGT
>GAPCpGES_18
ATTGGTATTTTTGTTTTTTATGAGTTAGTTTTATAGAGGTTGTTTTTTTGTTTGTGTTTT
TTGTTTTGTATTGTTGATGAGGGTTTTGGGGTTGGGTTATTAGGGTAGAGTGGTGTTGTG
GGTTTGTATGGGTGTGTAGATTGGGTTGTGTATTGTTTTGGGTGGGTGTTATTTTTTTAT
TTGGGTTTTGGTGGTGTTTTTTTGTGTTTTTTGGTTGAGTTTTGTGAGGAGTTGGGGTTG
GGGTGTGGTGGTTAGGGGATTGTTTTGGGGTTGAGTGTGTTTTGTTTTAGGTTTGTGTTT
TGTTTTTAGAGTGTTGGAGGATTGTGAATAGTTAGTATTATGTTTTTTTGTTTGTTTTTG
TGTTGTTTTTTTTTTTTTTGTTTTATAAATTTTAGGATTGTTTAGTTTAGGAAGTGTTGG
AAGGAAGTTAGTTTTGGAGGGGGGTGGAGGTTGGAAATTTTTGGTAGTTGTGTGTGTGTG
TGTGTGTGTGTGTGTGTGTTTGTGAGTGTAGTTGAAGGAAGTAAGTGGGTGGGTTGAGTT
GTGTGGGTTGGGGGAGAGATTGTGTTTTTTTGAAGTGGTTTTGTTAGTTTAGAGTATCGT
TTAGGGTTGTTTTGGAGGAAATTGGTTTTGTGAGGAGTTGTGGTTGTTTGTTTGTTTTTT
GTTGTTAGTTAGGTTTTTTGTATAGTGTAGGAGGTGGTGTTGGATTTTGTAGGGAGGGTT
TGGGATGGGTGTTTATGGAATTGTTTTTTTTGT
>GAPCpGES_16
GATTTGTTTTGTATTGTTGATGAGGGTTTCGGGGTTGGGTTATTAGGGTAGAGTGGTGTT
GTGGGTTTGTATGGGTGTGTAGATTGGGTTGTGTATTGTTTTGGGTGGGTGTTATTTTTT
TATTTGGGTTTTGGTGGTGTTTTTTTGTGTTTTTTGGTTGAGTTTTGTGAGGAGTTGGGG
TTGGGGTGCGGTGGTTAGGGGATTGTTTTGGGGTTGAGTGTGTTTTGTTTTAGGTTTGTG
TTTTGTTTTTAGAGTGTTGGAGGATTGTGAATAGTTAGTATTATGTTTTTTTGTTTGTTT
TTGTGTTGTTTTTTTTTTGTTTTATAAATTTTAGGATTGTTTAGTTTAGGAAGTGTTGGA
AGGAAGTTAGTTTTGGAGGGGGGTGGAGGTTGGAAATTTTTGGTAGTTGTGTGTGTGTGT
GTGTGTGTGTGTGTTTGTGAGTGTAGTTGAAGGAAGTAAGTGGGTGGGTTGAGTTGTGTG
GGTTGGGGGAGAGATTGTGTTTTTTTGAAGTGGTTTTGTTAGTTTAGAGTATTGTTTAGG
GTTGTTTTGGAGGAAATTGGTTTTGTGAGGAGTTGTGGTTGTTTGTTTGTTTTTTGTTGT
TAGTTAGGTTTTTTGTATAGTGTAGGAGGTGGTGTTGGATTTTGTAGGGAGGGTTTGGGA
TGGGTGTTTATGGAATTGTTTTTTTTGT
>GAPCpGES_12
TATATTATTATTTTGTTTTGAATTTTTTAGATATTTCGTTAGGTTATATTTAAATGTATT
GGTGGTTTTTTTTTTGTTTTAGTTTTTGTTTGGTATTTTTGTTTTTTATGAGTTAGTTTT
ATAGAGGTTGTTTTTTTGTTTGTGTTTCTTGTTTTGTATTGTTGATGAGGGTTTTGGGGT
TGGTTATTAGGGTAGAGTGGTGTCGTGGGTTTGTATGGGTGTGTAGATTGGGTTGTGTAT
TGTTTTGGGTGGGTGTTATTTTTTTATTTGGGTTTTGGTGGTGTTTTTTTGTGTTTTTTG
GTTGAGTTTTGTGAGGAGTTGGGGTCGGGGTGTGGTGGTTAGGGGATTGTTTTGGGGTTG
AGTGTGTTTTGTTTTAGGTTTGTGTTTTGTTTTTAGAGTGTTGGAGGATTGTGAATAGTT
AGTATTATGTTTTTTTGTTTGTTTTTGTGTTGTTTTTTTTTTGTTTTATAAATTTTAGGA
TTGTTTAGTTTAGGAAGTGTTGGAAGGAAGTTAGTTTTGGAGGGGGGTGGAGGTTGGAAA
TTTTTGGTAGTTGTGTGTGTGTGTGTGTGTGTGTGTGTGTTTGTGAGTGTAGTTGAAGGA
AGTAAGTGGGTGGGTTGAGTTGTGTGGGTTGGGGGAGAGATTGTGTTTTTTTGAAGTGGT
TTTGTTAGTTTAGAGTATTGTTTAGGGTTGTTTTGGAGGAAATTGGTTTTGTGAGGAGTT
GTGGTTGTTTGTTTGTTTTTTGTTGTTAGTTAGGTTTTTTGTATAGTGTAGGAAGTGGTG
TTGGATTTTGTAGGGAGGGTTTGGGATGGGTGTTTATGGAATTGTTTTTTTTGT

129 Clones
>GAPCpGES_15
GTTTGGTATTTTTGTTTTTCATGAGTTAGTTCTATAGAGGTTGTTTTTTTGTTTGTGTTT
TTTGTTTTGTATTGTTGATGAGGGTTTCGGGGTTGGTTATTAGGGTAGAGTGGTGTTGTG
GGTTTGTATGGGTGTGTAGATTGGGTTGTGTATTGTTTTGGGTGGGTGTTATTTTTTTAT
TTGGGTTTTGGTGGTGTTTTTTTGTGTTTTTTGGTTGAGTTTTGTGAGGAGTTGGGGTTG
GGGTGTGGTGGTTAAGGGATTGTTTTGGGGTTGAGTGTGTTTTGTTTTAGGTTTGTGTTT
TGTTTTTAGAGTGTTGGAGGATTGTGAATGGTTAGTATTTTGTTTTCTTGTTTGTTTTTG
TGTTGTTTTTTTTTTTTGTTTTATAAATTTTAGGATTGTTTAGTTTAGGAAGTGTTGGAA
GGAAGTTAGTTTGGGAGGGGGGTGGAGGTTGGAAATTTTTGGTAGTTGTGTGTATGTGTG
TGTGTGTGTGTGTGTGTGTGTGTGTGTGTGTGTGTGTGTGTGTGTGTTTGTGAGTGTAGT
TGAAGGAAGTAAGTGGGTGGGTTGAGTTGTGTGGGTTGGGGGAGAGATTGTGTTTTTTTG
AAGTGGTTTTGTTAGTTTAGAGTATTGTTTAGGGTTGTTTTGGAGGAAATTGGTTTTGTG
AGGAGTTGTGGTTGTTTGTTTGTTTTTTGTTGTTAGTTGGGTTTTTTGTATAGTGTAGGA
GGTGGTGTTGGATTTTGTAGGGAGGGTTTGGGATGGGTGTTTATGGAATTGTTTTTTTTG
T
>CdGAP139MP5_SP6
GTTTGGTATTTTTGTTTTTTATGAGTTAGTTTTATAGAGGTTGTTTTTTTTGTTTGTGTT
TTTTGTTTTGTATTGTTGATGAGGGTTTTGGGGTTGGGTTATTAGGGTAGAGTGGTTGTG
TGGGTTTGTATGGGTGTGTAGATTGGGTTGTGTATTGTTTTGGGTGGGTGTTATTTTTTT
ATTTGGGTTTTGGTGGTGTTTTTTTGTGTTTTTTGGTTGAGTTTTGTGAGGAGTTGGGGT
TGGGGTGTGGTGGTTAGGGGATTGTTTTGGGGTTGAGTGTGTTTTGTTTTAGGTTTGTGT
TTTGTTTTTAGAGTGTTGGAGGATTGTGAATAGTTAGTATTATGTTTTTTTGTTTGTTTT
TGTGTTGTTTTTTTTTTTTTGTTTTATAAATTTTAGGATTGTTTAGTTTAGGAAGTGTTG
GAAGGAAGTTAGTTTCGGAGGGGGGTGGAGGTTGGAAATTTTTGGTAGTTGTGTGTGTGT
GTGTGTTGTGTGTGTGTGTTTGTGAGTGTAGTTGAAGGAAGTAAGTGGGTGGGTTGAGTT
GTGTGGGTTGGGGGAGAGATTGNGTTTTTTTGAAGTGGTTTTGTTAGTTTAGAGTATTGT
TTAGGGTTGTTTTGGAGGAAATTGGTTTTGTGAGGAGTTGTGGTTGTTTGTTTGTTTTTT
GTTGTTAGTTGGGTTTTTTGTATAGTGTAGGAGGTGGTGTTGGATTTTGTAGGGAGGGTT
TGGGATGGGTGTTTATGGAATTGTTTTTTTTGT
>CdGAP139MP6_SP6
GTTTGGTATTTTTGTTTTTTTATGAGTTAGTTTTTATAGAGGTTTGTTTTTTTGTTTGTG
TTTTTTTGTTTTGTATTGTTGAAGAGGGTTTTGGGGTTGGGTTATTAGGGTAGAGTGGTG
TTGTGGGTTTGTATGGGTGTGTAGATTGGGTTGTGTATTGTTTTGGGTGGGTGTTATTTT
TTTATTTGGGTTTTGGTGGTGTTTTTTTGTGTTTTTTGGTTGAGTTTTGTGAGGAGTTGG
GGTTGGGGTGTGGTGGTTAGGGGATTGTTTTGGGGTTGAGTGTGTTTTGTTTTAGGTTTG
TGTTTTGTTTTTAGAGTGTTGGAGGATTGTGAATAGTTAGTATTATGTTTTTTTGTTTGT
TTTTGTGTTGTTTTTTTTTTTTTGTTTTATAAATTTTAGGATTGTTTAGTTTAGGAAGTG
TTGGAAGGAAGTTAGTTTCGGAGGGGGGTGGAGGTTGGAAATTTTTGGTAGTTGTGTGTG
TGTGTGTGTGTGTGTGTGTGTGTTTGTGAGTGTAGTTGAAGGAAGTAAGTGGGTGGGTTG
AGTTGTGTGGGTTGGGGGAGAGATTGTGTTTTTTTGAAGTGGTTTTGTTAGTTTAGAGTA
TTGTTTAGGGTTGTTTTGGAGGAAATTGGTTTTGTGAGGAGTTGTGGTTGTTTGTTTGTT
TTTTGTTGTTAGTTGGGTTTTTTGTATAGTGTAGGAGGTGGTGTTGGATTTTGTAGGGAG
GGTTTGGGATGGGTGTTTATGGAATTGTTTTTTTTGT
>CdGAP155MP3_SP6
GTTTGGTATTTTTGTTTTTTATGATTTAGTTCTATAGAGGTTGTTTTTTTGTTTGTGTTT
TTTGTTTTGTATTGTTGATGAGGGTTTTGGGGTTGGGTTATTAGGGTAGAGTGGTGTTGT
GGGTTTGTATGGGTGTGTAGATTGGGTTGTGTATTGTTTTGGGTGGGTGTTATTTTTTTT
ATTTGGGTTTTGGTGGTGTTTTTTTGTGTTTTTTGGTTGAGCTTTTTGCGAGGAGTTGGG
GTTGGGGTGTGGTGGTTAGGGGATTGTTTTGGGGTTGAGTGTGTATTGTTTTAGGTTTGT
GTTTTGTTTTTAGAGTGTTGGAGGATTGTGAATAGTTAGTATTATGTTTCTTTGTTTGTT
TTTGTGTTGTTTTTTTTTTTTGTTTTATAAATTTTAGGATTGTTTAGTTTAGGAAGTGTT
GGAAGGAAGTTAGTTTTGGAGGGGGGTGGAGGTTGGAAATTTTTGGTAGTTGTGTGTGTG
TGTGTGTGTGTGTGTGTGTTTGTGAGTGTAGTTGAAGGAAGTAAGTGGGTGGGTTGAGTT
GTGTGGGTTGGGGGAGAGATTGTGTTTTTTGAAGTGGTTTTGTTAGTTTAGAGTATTGTT
TAGGGTTGTTTTGGAGGAAATTGGTTTTGTGAGGAGTTGTGGTTGTTTGTTTGTTTTTTG
TTGTTAGTTGGGTTTTTTGTATAGTGTAGGAGGTGGTGTTGGATTTTGTAGGGAGGGTTT
GGGATGGGTGTTTATGGAATTGTTTTTTTTGT
