# Supplementary material for: Epigenetic interplay between mouse endogenous retroviruses and host genes
Source: Genome Biol. 2012 Oct 3;13(10):R89. doi: 10.1186/gb-2012-13-10-r89 (PMC3491417; doi:10.1186/gb-2012-13-10-r89)
Supplement: Additional file 4 — All bisulfite sequencing data. Compilation of all bisulfite sequences. [file gb-2012-13-10-r89-S4.zip › IAP6428_TE_ES.rtf]

CdGAP 3LTR
B6129 ES Cells
B6 clones
>CLLTRES1-M13R
GGGAAGGAAGTTTTGTTTATATTATAAGTGCGTTTTCGTAAAATAAGGAGTGTTATACGT
TAAGATACGGCGGTTGGTTTTTAAGATAAGTTTTTTAGTTTGGAGGTATTTTTGTTTGTT
TGTTATTCGTCGCGTTTTTACGATCGGTTAGGAAGAATATAATAATTAGAATTTTTTACG
GTAAAGTTTTATTGTTTATATTTTTTTGGGGTTAGAGTGTAAGAAGTAAGAGAGAGAGAA
AAACGAAATTTTTTTTATTTAAAAGAGAATAATAATTGTTTAGGACGTATTATTTTTTGA
TTGGTTGTAGTCTATGGTCGAGTTGACGTTTACGGGAAAAATAGAGTATAAGTAGTCGTA
AATATTTTTGGTTTATGCGTAGATTATTTGTTTATTAATTTAGAATATAGGATGTTAGCG
TTATTTTGTGACGGCGAATGTGGGGGCGGTTTTTTATAGTTTTTTTTTTTTTTTTTAATA
AGAGTAAATAGGTTATTTATATTAATGAGAGTGGAGATAGAGGTTAAATTTTTAGTGTGT
AGGTAAAGGAGTTATGTATAGGATTAGTTTTTAGGTTTATAGGTTTTTATTTAGAGTAAT
TTTGATTTGTCTTCGTGTCGTTTTGTTTGGGGGAAGGGAATTAGGATATTGAATTTTTAT
GAAAGATGATATGTTTTTTTAGAATAGGTTTATATATGTCGTAGAGTTTTTTTATTGTAG
TGTTTAGTTTTGTAATT
>CLLTRES2-M13R
GGGAAGGAAGTTTTGTTTATATTATAAGTGCGTTTTCGTAAAATAAGGAGTGCTATACGT
TAAGATACGGCGGTTGGTTTTTAAGGTAAGTTTTTTAGTTTGGAGGTATTTTTGTTTGTT
TGTTATTCGTCGCGTTTTTACGATCGGTTAGGAAGAATATAATAATTAGAATTTTTTACG
GTAAAGTTTTATTGTTTATATTTTTTTGGGGTTAGAGTGTAAGAAGTAAGAGAGAGAGAA
AAACGAAATTTTTTCTATTTAAAAGAGAATAATAATTGTTTAGGACGTATTATTTTTTGA
TTGGTTGTAGTTCATGGTCGAGTTGACGTTTACGGGAAAAATAGAGTATAAGTAGTCGTA
AATATTTTTGGTTTATGCGTAGATTATTTGTTTATTAATTTAGAATATAGGATGTTAGCG
TTATTTTGTGACGGCGAATGTGGGGGCGGTTTTTTATAGTTTTTTTTTTTTTTTTAATAA
GAGTAAATAGGTTATTTATATTAATGAGAGTGGAGATAGAGGTTAAATTTTTAGTGTGTA
GGTAAAGGAGTTATGTATAGGATTAGTTTTTAGGTTTACAGGTTTTTATTTAGAGTAATT
TTGATTTGTTTTCGTGTCGTTTTGTTTGGGGGAAGGGAATTAGGATATTGAATTTTTATG
AAAGATGATATGTTTTTTTAGAATAGGTTTATATATGTCGTAGAGTTTTTTTATTGTAGT
GTTTAGTTTTGTAATT
>CLLTRES3-M13R
GGGAAGGAAGTTTTGTTTATATTATAAGTGCGTTTTCGTAAAATAAGGAGTGTTATACGT
TAAGATACGGCGGTTGGTTTTTAAGATAAGTTTTTTAGTTTGGAGGCATTTTTGTTTGTT
TGTTATTCGTCGCGTTTTTACGATCGGTTAGGAAGAACATAATAATTAGAATTTTTTACG
GTAAAGTTTTATTGTTTATATTTTTTTGGGTTAGAGTGTAAGAAGTAAGAGAGAGAGAAA
AACGAAATTTTTTTTATTTAAAAGAGAATAATAATTGTTTAGGACGTATTATTTTTTGAT
TGGTTGTAGTTTATGGTCGAGTTGACGTTTACGGGAAAAATAGAGTATAAGTAGTCGTAA
ATATTTTTGGTTTATGCGTAGATTATTTGTTTATTAATTTAGAATATAGGATGTTAGCGT
TATTTTGTGACGGCGAATGTGGGGGCGGTTTTTTATAGTTTTTTTTTTTTTTTTAATAAG
AGTAAATAGGTCATTTATATTAATGAGAGTGGAGATAGAGGTTAAATTTTTAGTGTGTAG
GTAAAGGAGTTATGTATAGGATTAGTTTTTAGGTTTATAGGTTTTTATTTAGAGTAATTT
TGATTTGTTTTCGTGTCGTTTTGTTTGGGGGAAGGGAATTAGGGTATTGAATTTTTATGA
AAGATGATATGTTTTCTTAGAATAGGTTTATATATGTCGTAGAGTTTTTTTATTGTAGTG
TTTAGTTTTGTAATT
>CLLTRES4-M13R
GGGAAGGAAGTTTTGTTTATATTATAAGTGCGTTTTCGTAAAATAAGGAGTGTTATACGT
TAAGATACGGCGGTTGGTTTTTAAGATAAGTTTTTTAGTTTGGAGGTATTTTTGTTTGTT
TGTTATTCGTCGCGTTTTTACGATCGGTTAGGAAGAATATAATAATTAGAATTTTTTACG
GTAAAGTTTTATTGTTTATATTTTTTTGGGGTTAGAGTGTAAGAAGTAAGAGAGAGAGAA
AAACGAAATTTTTTTTATTTAAAAGAGAATAATAATTGTTTAGGACGTATTATTTTTTGA
TTGGTTGTAGTTTATGGTCGAGTTGACGTTTACGGGAAAAATAGAGTATAAGTAGTCGTA
AATATTTTTGGTTTATGCGTAGATTATTTGTTTATTAATTTAGAATATAGGATGTTAGCG
TTATTTTGTGACGGCGAATGTGGGGGCGGTTTTTTACAGTTTTTTTTTTTTTTTTAATAA
GAGTAAATAGGTTATTTATATTAATGAGAGTGGAGATAGAGGTTAAATTTTTAGTGTGTA
GGTAAAGGAGTTATGTATAGGATTAGTTTTTAGGTTTATAGGTTTTTATTTAGAGTAATT
TTGATTTGTTTTCGTGTCGTTTTGTTTGGGGGAAGGGAATTAGGATATTGAATTTTCATG
AAAGATGATATGTTTTTTTAGAATAGGTTTATATATGTCGTAGAGTTTTTTTATTGTAGT
GTTTAGTTTTGTAATT
>CLLTRES5-M13R
GGGAAGGAAGTTTTGTTTATATTATAAGTGCGTTTTCGTAAAATAAGGAGTGTTATACGT
TAAGATACGGCGGTTGGTTTTTAAGATAAGTTTTTTAGTTTGGAGGTATTTTTGTTTGTT
TGTTATTCGTCGCGTTTTCGCGATCGGACAGGAAGAATATAATAATTAGAATTTTTTACG
GTAAAGTTTTATTGTTTATATTTTTTTGGGGTTAGAGTGGAAGAAGTAAGAGAGAGAGAA
AAACGAAATTTTTTTTATTTAAAAGAGAATAATAATTGTTTAGGACGTATTATTTTTTGA
TTGGTTGTAGTTTATGGTTGAGTTGACGTTTACGGGAAAAATAGAGTATAAGTAGTCGTA
AATATTTTTGGTTTATGCGTAGATTATTTGTTTATTAATTTAGAATATAGGATGTTAGCG
TTATTTTGTGACGGCGAATGTGGGGGCGGTTTTTTATAGTTTTTTTTTTTTTTTTTAATA
AGAGTAAATAGGTTATTTATATTAATGAGAGTGGAGATAGAGGTTAAATCTTTAGTGTGT
AGGTAAAGGAGTTATGTATAGGATTAGTTTTTAGGTTTATAGGTTTTTATTTAGAGTAAT
TTTGATTTGTTTTCGTGTCGTTTTGTTTGGGGGAAGGGAATTAGGATATTGAATTTTTAT
GAAAGATGATATGTTTTTTTAGAATAGGTTTATATATGTCGTAGAGTTTTTTTATTGTAG
TGTTTAGTTTTGTAATT
>CLLTRES6-M13R
GGGAAGGAAGTTTTGTTTATATTATAAGTGCGTTTTCGTAAAATAAGGAGTGTTATATGT
TAAGATACGGCGGTTGGTTTTTAAGATAAGTTTTTTAGTTTGGAGGTATTTTTGTTTGTT
TGTTATTCGTTGCGTTTTTATGATCGGTTAGGAAGAATATAATAATTAGAATTTTTTACG
GTAAAGTTTTATTGTTTATATTTTTTTGGGGTTAGAGTGTAAGAAGTAAGAGAGAGAGAA
AAACGAAATTTTTTTTATTTAAAAGAGAATAATAATTGTTTAGGACGTATTATTTTTTGA
TTGGTTGTAGTTTATGGTCGAGTTGATGTTTATGGGAAAAATAGAGTATAAGTAGTTGTA
AATATTTTTGGTTTATGTGTAGATTATTTGTTTATTAATTTAGAATATAGGATGTTAGCG
TTATTTTGTGACGGTGAATGTGGGGGTGGTTTTTTATAGTTTTTTTTTTTTTTTAATAAG
AGTAAATAGGTTATTTATATTAATGAGAGTGGAGATAGAGGTTAAATTTTTAGTGTGTAG
GTAAAGGAGTTATGTATAGGATTAGTTTTTAGGTTTATAGGTTTTTATTTAGAGTAATTT
TGATTTGTTTTCGTGTCGTTTTGTTTGGGGGAAGGGAATTAGGATATTGAATTTTTATGA
AAGATGATATGTTTTTTTAGAATAGGTTTATATATGTCGTAGAGTTTTTTTATTGTAGTG
TTTAGTTTTGTAATT
>CLLTRES7-M13R
GGGAAGGAAGTTTTGTTTATATTATAAGTGCGTTTTTGTAAAATAAGGAGTGTTATACGT
TAAGATACGGTGGTTGGTTTTTAAGATAAGTTTTTTAGTTTGGAGGTATTTTTGTTTGTT
TGTTATTCGTCGCGTTTTTACGATCGGTTAGGAAGAATATAATAACTAGAATTTTTTACG
GTAAAGTTTTATTGTTTATATTTTTTTGGGGTTAGAGTGTAAGAAGTAAGAGAGAGAGAA
AAACGAAATTTTTTTTATTTAAAAGAGAATAATAATTGTTTAGGACGTATTATTTTTTGA
TTGGTTGTAGTTTATGGTTGAGTTGACGTTTATGGGAAAAATAGAGTATAAGTAGTCGTA
AATATTTTTGGTTTATGTGCAGATTATTTGTTTATTAATTTAGAATATAGGATGTTAGTG
TTATTTTGTGATGGCGAATGTGGGGGTGGTTTTTTATAGTTTTTTTTTTTTTTTTTAATA
AGAGTAAATAGGTTATTTATATTAATGAGAGTGGAGATAGAGGTTAAATTTTTAGTGTGT
AGGTAAAGGAGTTATGTATAGGATTAGTTTTTAGGTTTATAGGTTTTTATTTAGAGTAAT
TTTGATTTGTTTTCGTGTCGTTTTGTTTGGGGGAAGGGAATTAGGATATTGAATTTTTAT
GAAAGATGATATGTTTTTTTAGAATAGGTTTATATATGTCGTAGAGTTTTTTTATTGTAG
TGTTTAGTTTTGTAATT
>CLLTRES8-M13R
GGGAAGGAAGTTTTGTTTATATTATAAGTGCGTTTTCGTAAAATAAGGAGTGTTATATGT
TAAGATACGGCGGTTGGTTTTTAAGATAAGTTTTTTAGTTTGGAGGTATTTTTGTTTGTT
TGTTATTCGTCGCGTTTTTACGACCGGTTAGGAAGAATATAATAACTAGAATTTTTTACG
GTAAAGTTTTATTGTTTATATTTTTTTGGGGTTAGAGTGTAAGAAGTAAGGGAGAGAGAA
AAACGAAATTTTTTTTATTTAAAAGAGAATAATAATTGTTTAGGACGTATTACTTTTTGA
TTGGTTGCAGTTTATGGTCGAGTTGACGTTTATGGGAAAAGTAGAGTATAAGTAGTCGTA
AATATTTTTGGTTTATGCGTAGATTATTTGTTTATTAATTTAGAACATAGGATGTTAGCG
TTATTTTGTGACGGCGAATGTGGGGGCGGTTTTTTATAGTTTTTTTTTTTTTTTTTTAAT
AAGAGTAAATAGGTTATCTATATTAATGAGAGTGGAGATAGAGGTTAAATTTTTAGTGTG
TAGGTAAAGGAGTTATGTATAGGATTAGTTTTTAGGTTTATAGGTTTTTATTTAGAGTAA
TTTTGATTTGTTTTCGTGTCGTTTTGTTTGGGGGAAGGGAATTAGGATATTGAATTTTTA
TGAAAGATGATATGTTTTTTTAGAATAGGTTTATATATGTCGTAGAGTTTTTTTATTGTA
GTGTTTAGTTTTGTAATT
>CLLTRES9-M13R
GGGAAGGAAGTTTTGTTTATATTATAAGTGCGTTTTCGTAAAATAAGGAGTGTTATACGT
TAAGATACGGTGGTTGGTTTTTAAGATAAGTTTTTTAGTTTGGAGGTATTTTTGTTTGTT
TGTTATTCGTCGCGTTTTTACGATCGGTTAGGAAGAATATAATAATTAGAATTTTTTACG
GCAAAGTTTTATTGTTTATATTTTTTTGGGGTTAGAGTGTAAGAAGTAAGAGAGAGAGAA
AAACGAAATTTTTTTTATTTAAAAGAGAATAATAATTGTTTAGGACGTATTATTTTTTGA
TTGGTTGTAGTTTATGGTCGAGTTGACGTTTACGGGAAAAATAGAGTATAAGTAGTCGTA
AATATTTTTGGTTTATGCGTAGATTATTTGTTTATTAATTTAGAATATAGGATGTTAGCG
TTATTTTGTGACGGCGAATGTGGGGGCGGTTTTTTATAGTTTTTTTTTTTTTTAATAAGA
GTAAATAGGTTATTTATATTAATGAGAGTGGAGATAGAGGTTAAATTTTTAGTGTGTAGG
TAAAGGAGTTATGTATAGGATTAGTTTTTAGGTTTATAGGTTTTTATTTAGAGTAATTTT
GATTTGTTTTCGTGTCGTTTTGTTTGGGGGAAGGGAATTAGGATATTGAATTTTTATGAA
AGATGATATGTTTTTTTAGAATAGGTTTATATATGTCGTAGAGTTTTTTTATTGTAGTGT
TTAGTTTTGTAATT
>CLLTRES10-M13R
GGGAAGGAAGTTTTGTTTATATTATAAGTGCGTTTTCGTAAAATAAGGAGTGTTATACGT
TAAGATACGGCGGTTGGTTTTTAAGATAAGTTTTTTAGTTTGGAGGTATTTTTGTTTGTT
TGTTATTCGTTGCGTTTTTACGATCGGTTAGGAAGAATATAATAATTAGAATTTTTTACG
GTAAAGTTTTATTGTTTATATTTTTTTGGGGTTAGAGTGTAAGAAGTAAGAGAGAGAGAA
AAACGAAATTTTTTTTATTTAAAAGAGAATAATAATTGTTTAGGACGTATTATTTTTTGA
TTGGTTGTAGTTTATGGTTGAGTTGACGTTTACGGGAAAAATAGAGTATAAGTAGTTGTA
AATATTTTTGGTTTATGCGTAGATTATTTGTTTATCAATTTAGAATATAGGATGTTAGCG
TTATTTTGTGATGGCGAATGTGGGGGCGGTTTTTTATAGTTTTTTTTTTTTTTTTAATAA
GAGTAAATAGGTTATTTATATTAATGAGAGTGGAGATAGAGGTTAAATTTTTAGTGTGTA
GGTAAAGGAGTTATGTATAGGATTAGTTTTTAGGTTTATAGGTTTTTATTTAGAGTAATT
TTGATTTGTTTTCGTGTCGTTTTGTTTGGGGGAAGGGAACTAGGATATTGAATTTTTATG
AAAGATGATATGTTTTTTTAGAATAGGTTTATATATGTCGTAGAGTTTTTTTATTGTAGT
GTTTAGTTTTGTAATT
>CLLTRES11-M13R
GGGAAGGAAGTTTTGTTTATATTATAAGTGCGTTTTTGTAAAATAAGGAGTGTTATACGT
TAAGATACGGCGGTTGGTTTTTAAGATAAGTTTTTTAGTTTGGAGGTATTTTTGTTTGTT
TGTTATTCGTCGCGTTTTTACGATCGGTTAGGAAGAATATAATAATTAGAATTTTTTGCG
GTAAAGCTTTATTGTTTATATTTTTTTGGGGTTAGAGTGTAAGAAGTAAGAGAGAGAGAA
AAATGAAATTTTTTTTATTTAAAAGAGAATAATAATTGTTTAGGATGTATTATTTTTTGA
TTGGTTGTAGTTTATGGTCGAGTTGACGTTTATGGGAAAAATAGAGTATAAGTAGTCGTA
AATATTTTTGGTTTATGCGTAGATTATTTGTTTATTAATTTAGAATATAGGATGTTAGCG
TTATTTTGTGACGGCGAATGTGGGGGTGGTTTTTTATAGTTTTTTTTTTTTTTTTTTAAT
AAGAGTAAATAGGTTATTTATATTAATGCGAGTGGAGATAGAGGTTAAATTTTTAGTGTG
TAGGTAAAGGAGTTATGTATAGGATTAGTTTTTAGGTTTATAGGTTTTTATTTAGAGTAA
TTTTGATTTGTTTTCGTGTCGTTTTGTTTGGGGGAAGGGAATTAGGATATTGAATTTTTA
TGAAAGATGATATGTTTTTTTAGAATAGGCTTATATATGTCGTAGAGTTTTTTTATTGTA
GTGTTTAGTTTTGTAATT
>CLLTRES12-M13R
GGGAAGGAAGTTTTGTTTATATTATAAGTGTGTTTTTGTAAAATAAGGAGTGTTATATGT
TAAGATATGGTGGTTGGTTTTTAAGATAAGTTTTTTAGTTTGGAGGTATTTTTGTTTGTT
TGTTATTTGTTGTGTTTTTATGATCGGTTAGGAAGAATATAATAATTAGAATTTTTTATG
GTAAAGTTTTATTGTTTATATTTTTTTGGGGTTAGAGTGTAAGAAGTAAGAGAGAGAGAA
AAATGAAATTTTTTTTATTTAAAAGAGAATAATAATTGTTTAGGATGTATTATTTTTTGA
TTGGTTGTAGTTTATGGTTGAGTTGATGTTTATGGGAAAAATAGAGTATAAGTAGTCGTA
AATATTTTTGGTTTATGTGTAGATTATTTGTTTATTAATTTAGAATATAGGATGTTAGTG
TTATTTTGTGATGGTGAATGTGGGGGTGGTTTTTTATAGTTTTTTTTTTTTTTTTAATAA
GAGTAAATAGGTTATTTATATTAATGAGAGTGGAGATAGAGGTTAAATTTTTAGTGTGTA
GGTAAAGGAGTTATGTATAGGATTAGTTTTTAGGTTTATAGGTTTTTATTTAGAGTAATT
TTGATTTGTTTTTGTGTTGTTTTGTTTGGGGGAAGGGAATTAGGATATTGAATTTTTATG
AAAGATGATATGTTTTTTTAGAATAGGTTTATATATGTCGTAGAGTTTTTTTATTGTAGT
GTTTAGTTTTGTAATT
>CLLTRES13-M13R
GGGAAGGAAGTTTTGTTTATATTATAAGTGCGTTTTCGTAAAATAAGGAGTGTTATACGT
TAAGATACGGCGGTTGGTTTTTAAGATAAGTTTTTTAGTTTGGAGGTATTTTTGTTTGTT
TGTTATTCGTCGCGTTTTTACGATCGGTTAGGAAGAATATAATAATTAGAATTTTTTACG
GTAAAGTTTTATTGTTTATATTTTTTTGGGGTTAGAGTGTAAGAAGTAAGAGAGAGAGAA
AAACGAAATTTTTTTTATTTAAAAGAGAATAATAATTGTTTAGGACGTATTATTTTTTGA
TTGGTTGTAGTTTATGGTCGAGTTGACGTTTACGGGAAAAATAGAGTATAAGTAGTCGTA
AATATTTTTGGTTTATGCGTAGATTATTTGTTTATTAATTTAGAATATAGGATGTTGGCG
TTATTTTGTGACGGCGAATGTGGGGGCGGTTTTTTATAGTTCTTTTTTTTTTTTAATAAG
AGTAAATAGGTCATTTATATTAATGAGAGTGGAGATAGAGGTTAAATTTTTAGTGTGTAG
GTAAAGGAGTTATGTATAGGATTAGTTTTTAGGTTTATAGGTTTTTATTTAGAGTAATTT
TGATTTGTTTTCGTGTCGTTTTGTTTGGGGGAAGGGAATTAGGATATTGAATTTTTATGA
AAGATGATATGTTTTTTTAGAATAGGTTTATATATGTCGTAGAGTTTTTTTATTGTAGTG
TTTAGTTTTGTAATT
>CLLTRES14-M13R
GGGAAGGAAGTTTTGTTTATATTATAAGTGCGTTTTCGTAAAATAAGGAGTGTTATACGT
TAAGATACGGCGGTTGGTTTTTAAGATAAGTTTTTTAGTTTGGAGGTATTTTTGTTTGTT
TGTTATTCGTCGCGTTTTTACGATCGGTTAGGAAGAATATAATAATTAGAATTTTTTACG
GTAAAGTTTTATTGTTTATATTTTTTTGGGGTTAGAGTGTAAGAAGTAAGAGAGAGAGAA
AAACGAAATTTTTTTTATTTAAAAGAGAATAATAATTGTTTAGGACGTATTATTTTTTGA
TTGGTTGCAGTTTATGGTCGAGTTGATGTTTATGGGAAAAATAGAGTATAAGTAGTCGTA
AATATTTTTGGTTTATGCGTAGATTATTTGTTTATTAATTTAGAATATAGGATGTTAGCG
TTATTTTGTGACGGCGAATGTGGGGGCGGTTTTTTATAGTTTTTTTTTTTTTTTTTAATA
AGAGTAAATAGGTTATTTATATTAATGAGAGTGGAGATAGAGGTTAAATTTTTAGTGTGT
AGGTAAAGGAGTTATGTATAGGATTAGTTTTTAGGTTTATAGGTTTTTATTTAGAGTAAT
TTTGATTTGTTTTCGTGTCGTTTTGTTTGGGGGAAGGGAATTAGGATATTGAATCTTTAT
GAAGGATGATATGTTTTTTTAGAATAGGTTTATATATGTCGTAGAGTTTTTTTATTGTAG
TGTTTAGTTTTGTAATT
>CLLTRES15-M13R
GGGAAGGAAGTTTTGTTTATATTATAAGTGCGTTTTCGTAAAATAAGGAGTGTTATACGT
TAAGATACGGCGGTTGGTTTTTAAGATAAGTTTTTTAGTTTGGAGGTATTTTTGTTTGTT
TGTTATTCGTCGCGTTTTTACGATCGGTTAGGAAGAATATAATAATTAGAATTTTTTACG
GTAAAGTTTTATTGTTTATATTTTTTTGGGGTTAGAGTGTAAGAAGTAAGAGAGAGAGAA
AAACGAAATTTTTTTTATTTAAAAGAGAATAATAATTGTTTAGGACGTATTATTTTTTGA
TTGGTTGTAGTCTATGGTCGAGTTGACGTTTACGGGAAAAATAGAGTATAAGTAGTCGTA
AATATTTTTGGTTTATGCGTAGATTATTTGTTTATTAATTTAGAATATAGGATGTTAGCG
TTATTTTGTGACGGCGAATGTGGGGGCGGTTTTTTATAGTTTTTTTTTTTTTTTTTAATA
AGAGTAAATAGGTTATTTATATTAATGAGAGTGGAGATAGAGGTTAAATTTTTAGTGTGT
AGGTAAAGGAGTTATGTATAGGATTAGTTTTTAGGTTTATAGGTTTTTATTTAGAGTAAT
TTTGATTTGTCTTCGTGTCGTTTTGTTTGGGGGAAGGGAATTAGGATATTGAATTTTTAT
GAAAGATGATATGTTTTTTTAGAATAGGTTTATATATGTCGTAGAGTTTTTTTATTGTAG
TGTTTAGTTTTGTAATT
>CLLTRES16-M13R
GGGAAGGAAGTTTTGTTTATATTATAAGTGCGTTTTCGTAAAATAAGGAGTGTTATACGT
TAAGATACGGCGGTTGGTTTTTAAGATAAGTTTTTTAGTTTGGAGGTATTTTTGTTTGTT
TGTTATTCGTCGCGTTTTTACGATCGGTTAGGAAGAATATAATAATTAGAATTTTTTACG
GTAAAGCTTTATTGTTTATATTTTTTTGGGGTTAGAGTGTAAGAAGTAAGAGAGAGAGAA
AAACGAAATTTTTTTTATTTAAAAGAGAATAATAATTGTTTAGGATGTATTATTTTTTGA
TTGGTTGTAGTTCATGGTTGAGTTGATGTTTATGGGAAAAATAGAGTATAAGTAGTTGTA
AATATTTTTGGTTTATGCGTAGATTATTTGTTTATTAATTTAGAATATAGGATGTTAGTG
TTATTTTGTGATGGTGAATGTGGGGGCGGTTTTTTATAGTTTTTTTTTTTTTTTTTAATA
AGAGTAAATAGGTTATTTATATTAATGAGAGTGGAGATAGAGGTTAAATTTTTAGTGTGT
AGGTAAAGGAGTTATGTATAGGATTAGTTTTTAGGTTTATAGGTTTTTATTTAGAGTAAT
TTTGATTTGTTTTCGTGTCGTTTTGTTTGGGGGAAGGGAATTAGGATATTGAATTTTTAT
GAAAGATGATATGTTTTTTTAGAATAGGTTTATATATGTCGTAGAGTTTTTTTATTGTAG
TGTTTAGTTTTGTAATT
>CLLTRES17-M13R
GGGAAGGAAGTTTTGTTTATATTATAAGTGCGTTTTCGTAAAATAAGGAGTGTTATACGT
TAAGATACGGCGGTTGGTTTTTAAGATAAGTTTTTTAGTTTGGAGGTATTTTTGTTTGTT
TGTTATTCGTCGCGTTTTTACGATCGGTTAGGAAGAATATGATAATTAGAATTTTTTACG
GTAAAGTTTTATTGTTTATATTTTTTTGGGGTTAGAGTGTAAGAAGTAAGAGAGAGAGAA
AAACGAAATTTTTTTTATTTAAAAGAGAATAATAATTGTTTAGGATGTATTATTTTTTGA
TTGGTTGTAGTTTATGGTCGAGTTGACGTTTACGGGAAAAATAGAGTATAAGTAGTCGTA
AATATTTTTGGTTTATGCGTAGATTATTTGTTTATTAATTTAGAATACAGGATGTTAGCG
TCATTTTGTGACGGCGAATGTGGGGGTGGTTTTTTATAGTTTTTTTTTTTTTTTTTAATA
AGAGTAAATAGGTCATTTATATTAATGAGAGTGGAGATAGAGGTTAAATTTTTAGTGTGT
AGGTAAAGGAGTTATGTACAGGATTAGTTTTTAGGTTTATAGGTTTTTATTTAGAGTAAT
TTTGATTTGTTTTCGTGTCGTTTTGTTTGGGGGAAGGGAATTAGGATATTGAATTTTTAT
GAAAGATGATATGTTTTTTTAGAATAGGTTTATATATGTCGTAGAGTTTTTTTATTGTAG
TGTTTAGTTTTGTAATT
>CLLTRES18-M13R
GGGAAGGAAGTTTTGTTTATATTATAAGTGCGTTTTCGTAAAATAAGGAGTGTTATACGT
TAAGATACGGCGGTTGGTTTTTAAGACAAGTTTTTTAGTTTGGAGGTATTTTTGTTTGTT
TGTTATTCGTCGCGTTTTTACGATCGGTTAGGAAGAATATAATAATTAGAATTTTTTACG
GTAAAGTTTTATTGTTTATATTTTTTTGGGGTTAGAGTGTAAGAAGTAAGAGAGAGAGAA
AAACGAAATTTTTTTTATTTAAAAGAGAATAATAATTGTTTAGGACGCATTATTTTTTGA
TTGGTTGTAGTTTATGGTCGAGTTGACGTTTACGGGAAAAATAGAGTATAAGTAGTCGTA
AATATTTTTGGTTTATGCGTAGATTATTTGTTTATTAATTTAGAATATAGGATGTTAGTG
TTATTTTGTGACGGCGAATGTGGGGGCGGTTTTTTATAGTTTTTTTTTTTTTTTTTAATA
AGAGTAAATAGGTTATTTATATTAATGAGAGTGGAGATAGAGGTTAAATTTTTAGTGTGT
AGGTAAAGGAGTTATGTATAGGATTAGTTTTTAGGTTTATAGGTTTTTATTTAGAGTAAT
TTTGATTTGTTTTCGTGTCGTTTTGTTTGGGGGAAGGGAATTAGGATATTGAATTTTTAT
GAAAGCTGATATGTTTTTTTAGAATAGGTTTATATATGTCGTAGAGTTTTTTTATTGTAG
TCTTTAGTTTTGTAATT
>CLLTRES19-M13R
GGGAAGGAAGTTTTGTTTATATTATAAGTGCGTTTTCGTAAAATAAGGAGTGTTATACGT
TAAGATACGGCGGTTGGTTTTTAAGATAAGTTTTTTAGTCTGGAGGTATTTTTGTTTGTT
TGTTATTCGTCGCGTTTTTACGATCGGTTAGGAAGAATATAATAATTAGAATTTTTTACG
GTAAAGTTTTATTGTTTATATTTTTTTGGGGTTAGAGTGTAAGAAGTAAGAGAGAGAGAA
AAACGAAATTTTTTTTATTTAAAAGAGAATAATAATTGTTTAGGACGTATTATTTTTTGA
TTGGTTGTAGTTTATGGTCGAGTTGACGTTTACGGGAAAAATAGAGTATAAGTAGTCGTA
AATATTTTTGGTTTATGCGTAGATTATTTGTTTATTAATTTAGAATATAGGATGTTAGCG
TTATTTTGTGACGGCGAATGTGGGGGCGGTTTTTTATAGTTTTTTTTTTTTTTTTTAATA
AGAGTAAATAGGTTATTTATATTAATGAGAGTGGAGATAGAGGTCAAATTTTTAGTGTGT
AGGTAAAGGAGTTATGTATAGGATTAGTTTTTAGGTTTATAGGTTTTTATTTAGAGTAAT
TTTGATTTGTTTTCGTGTCGTTTTGTTTGGGGGAAGGGAATTAGGATATTGAATTTTTAT
GAAAGATGATATGTTTTTTTAGAATAGGTTTATATATGTCGTAGAGTTTTTTTATTGTAG
TGTTTAGTTTTGTAATT
>CLLTRES20-M13R
GGGAAGGAAGTTTTGTTTATATTATAAGTGCGTTTTCGTAAAATAAGGAGTGTTATACGT
TAAGATACGGCGGTTGGTTTTTAAGATAAGTTTTTTAGTTTGGAGGTATTTTTGTTTGTT
TGTTATTTGTCGCGTTTTTACGATCGGTTAGGAAGAATATAATAATTAGAATTTTTTACG
GTAAAGTTTTATTGTTTATATTTTTTTGGGGTTAGAGTGTAAGAAGTAAGAGAGAGAGGA
AAATGAAATTTTTTTTATTTAAAAGAGAATAATAATTGTTTAGGACGTATTATTTTTTGA
TTGGTTGTAGTTTATGGTCGAGCTGACGTTTACGGGAAAAATAGAGTATAAGTAGTCGTA
AATATTTTTGGTTTATGCGTAGATTATTTGTTTATTAATTTAGAATATAGGATGTTAGCG
TTATTTTGTGACGGCGAATGTGGGGGCGGTTTTTTATAGTTTTTTTTTTTTTTTTTAATA
AGAGTAAATAGGTTATTTATATTAATGAGAGTGGAGATAGAGGTTAAATTTTTAGTGTGT
AGGTAAAGGAGTTATGTATAGGATTAGTTTTTAGGTTTATAGGTTTTTATTTAGAGTAAT
TTTGATTTGTTTTCGTGTCGTTTTGCTTGGGGGAAGGGAATTAGGATATTGAATTTTTAT
GAAAGATGATATGTTTTTTTAGAATAGGTTTATGTATGTCGTAGAGTTTTTTTATTGTAG
TGTTTAGTTTTGTAATT
