# Supplementary material for: Epigenetic interplay between mouse endogenous retroviruses and host genes
Source: Genome Biol. 2012 Oct 3;13(10):R89. doi: 10.1186/gb-2012-13-10-r89 (PMC3491417; doi:10.1186/gb-2012-13-10-r89)
Supplement: Additional file 4 — All bisulfite sequencing data. Compilation of all bisulfite sequences. [file gb-2012-13-10-r89-S4.zip › IAP6428_TE_lung.rtf]

12-21-10
CdGAP 3'LTR-B6 Lung
MP Sequences
Plate A
>3LTR-Lung_2
GGGAAGGAAGTTTTGTTTATATTATAAGTGCGTTTTCGTAAAATAAGGAGTGTTATATGT
TAAGATATGGCGGTTGGTTTTTAAGATAAGTTTTTTAGTTTGGAGGTATTTTTGTTTGTT
TGTTATTTGTTGCGTTTTTATGATTGGTTAGGAAGAATATAATAATTAGAGTTTTTTATG
GTAAAGTTTTATTGTTTATATTTTTTTGGGGTTAGAGTGTAAGAAGTAAGAGAGAGAGAA
AAATGAAATTTTTTTTATTTAAAAGAGAATAATAATTGTTTAGGACGTATTATTTTTTGA
TTGGTTGTAGTTTATGGTTGAGTTGATGTTTATGGGAAAAATAGAGTATAAGTAGTTGTA
AATATTTNCGGTTTATGTGTAGATTATTTGTTTATTAATTTAGAATATAGGATGTTAGTG
TTACTTTGTGATGGTGAATGTGGGGGTGGTTTTTTATAGTTTTTTTTTTTTTTTTTAATA
AGAGTAAATAGGTTATTTATATTAATGAGAGTGGAGATAGAGGTTAAATTTTTAGTGTGT
AGGTAAAGGAGTTATGTATAGGATTAGTTTTTAGGTTTATAGGTTTTTATTTAGAGTAAT
TTTGATTTGTTTTTGTGTTGTTTTGTTTGGGGGAAGGGAATTAGGATATTGAATTTTTAT
GAAAGATGATATGTTTTTTTAGAATAGGTTTATATATGTCGTAGAGTTTTTTTATTGTAG
TGTTTAGTTTTGTAATT
>3LTR-Lung_3
GGGAAGGAAGTTTTGTTTTATATTATAAGTGTGTTTTTGTAAAATAAGGAGTGTTATATG
TTAAGATATGGTGGTTGGTTTTTAAGATAAGTTTTTTAGTTTGGAGGTATTTTTGTTTGT
TTGTTATTTGTTGTGTTTTTATGATTGGTTAGGAAGAATATAATAATTAGAATTTTTTAT
GGTAAAGTTTTATTGTTTATATTTTTTTGGGGTTAGAGTGTAAGAAGTAGGAGAGAGAGA
AAAATGAAATTTTTTTTATTTAAAAGAGAATAATAATTGTTTAGGATGTATTATTTTTTG
ATTGGTTGTAGTTTATGGTTGAGTTGATGTTTATGGGAAAAATAGAGTATAAGTAGTTGT
AAATATTTTTGGTTTATGTGTAGATTATTTGTTTATTAATTTAGAATATAGGATGTTAGT
GTTATTTTGTGACCGTGAATGTGGGGGTGGTTTTTTATAGTTTTTTTTTTTTTTTTTTTA
ATAAGAGTAAATAGGTTATTTATATTAATGAGAGTGGAGATAGAGGTTAAATTTTTAGTG
TGTAGGTAAAGGAGTTATGTATAGGATTAGTTTTTAGGTTTATAGGTTTTTATTTAGAGT
AATTTTGATTTGTTTTTGTGTTGTTTTGTTTGGGGGAAGGGAATTAGGATATTGAATTTT
TATGAAAGATGATATGTTTTTTTAGAATAGGTTTATATATGTCGTAGAGTCTTTTTATTG
TAGTGTTTAGTTTTGTAATT
>3LTR-Lung_5
GGGAAGGAAGTTTTGTTTATTTATAAGTGCGTTTTTGTAAAATAAGGAGTGTTATATGTT
AAGATATGGTGGTTGGTTTTTAAGATAAGTTTTTTAGTTTGGAGGTATTTTTGTTTGTTT
GTTATTTGTTGTGTTTTTATGATTGGTTAGGAAGAATATAATAATTAGAATTTTTTATGG
TAAAGTTTTATTGTTTATATTTTTTCGGGGTTAGAGTGTAAGAAGTAAGAGAGAGAGGAA
AATGAAATTTTTTTTATTTAAAAGAGAATAATAATTGTTTAGGATGTATTATTTTTTGAT
TGGTTGTAGTTTATGGTTGAGCTGATGTTTATGGGAAAAATAGAGTATAAGTAGTTGTAA
ATATTTTTGGTTTATGTGTAGATTATTTGTTTATTAATTTAGAATATAGGATGTTAGTGT
TATTTTGTGATGGTGAATGTGGGGGTGGTTTTTTATAGTTTTTTTTTTTTTTTTAATAAG
AGTAAATAGGTTATTTATATTAATGAGAGTGGAGATAGAGGTTAAATTTTTAGTGTGTAG
GTAAAGGAGTTATGTATAGGATTAGTTTTAGGTTTATAGGTTTTTATTTAGAGTAATTTT
GATTTGTTTTTGTGTTGTTTTGTTTGGGGGAAGGGAATTAGGATATTGAATTTTTATGAA
AGATGATATGTTTTTTTAGAATAGGTTTATATATGTCGTAGAGTTTTTTTATTGTAGTGT
TTAGTTTTGTAATT
>3LTR-Lung_6
GGGAAGGAAGTTTTGTTTATATCATAAGTGTGTTTTTGTAAAATAAGGAGTGTTATATGT
TAAGATATGGCGGTTGGTTTTTAAGATAAGTTTTTTAGTTTGGAGGTATTTTTGTTTGTT
TGTTATTTGTTGTGTTTTTATGATTGGTTAGGGAGAATATAATAATTAGAATTTTTATGG
TAAAGTTTTATTGTTTATATTTTTTTGGGGTTAGAGTGTAAGAAGTAAGAGAGAGAGAAA
AATGAAATTTTTTTTATTTAAAAGAGAATAATAATTGTTTAGGATGTATTGTTTTTTGAT
TGGTTGTAGTTTATGGTTGAGTTGATGTTTATGGGAAAAACCGAGTATAAGTAGTTGTAA
ATATTTTTGGTTTATGTGTAGATTATTTGTTTATTAATTTAGAATATAGGATGTTAGTGT
TATTTTGTGATGGTGAATGTGGGGGTGGTTTTTTATAGTTTTTTTTTTTTTTTTAATAAG
AGTAAATAGGTTATTTATATTAATGAGAGTGGAGATAGAGGTTAAATTTTTAGTGTGTAG
GTAAAGGAGTTATGTATAGGATTAGTTTTTAGGTTTATAGGTTTTTATTTAGAGTAATTT
TGATTTGTTTTCGTGTCGTTTTGTTTGGGGGAAGGGAATTAGGATATTGAATTTTTATGA
AAGATGATATGTTTTTTTAGAATAGGTTTATATATGTCGTAGAGTTTTTTTATTGTAGTG
TTTAGTTTTGTAATT
>3LTR-Lung_8
GGGAAGGAAGTTTTTGTTTTATATTATAAGCGCGTTTTCTGTAAAATAAGGAGTGTTTAT
ACGTTAAGATATGGTGGTTGGTTTTTAAGATAAGTTTTTTAGTTTGGAGGTATTTTTGTT
TGTTTGTTATTTGTCGTGTTTTTACGATTTGTTAGGAAGAATATAATAATTAGAATTTTT
TATGGTAAAGTTTTATTGTTTATATTTTTTTGGGGTTAGAGTGTAAGAAGTTAGAGAGAG
AGAAAAATGAAATTTTTTTTATTTAAAAGAGAATAATAATTGTTTAGGATGTATTATTTT
TTGATTGGTTGTAGTTTATGGTTGAGTTGATGTTTATGGGAAAAATAGAGTATAAGTAGT
TGTAAATATTTTTGGTTTATGTGTAGATTATTTGTTTATTAATTTAGAATATAGGATGTT
AGTGTTATTTTGTGACCGTGGATGTGGGGGTGGTTTTTTATAGTTTTTTTTTTTTTTTTA
ATAAGAGTAAATAGGTTATTTATATTAATGAGAGTGGAGATAGAGGTTAAATTTTTAGTG
TGTAGGTAAAGGAGTTATGTATAGGATTAGTTTTTAGGTTTATAGGTTTTTATTTAGAGT
AATTTTGATTTGTTTTTGTGTTGTTTTGTTTGGGGGAAGGGAATTAGGATATTGAATTTT
TATGAAAGATGATATGTTTTTTTAGAATAGGTTTATATATGTCGTAGAGTCTTTTTATTG
TAGTGTTTAGTTTTGTAATT
>3LTR-Lung_10
GGGAAGGAAGTTTTGGTTTTATATTATAAGTGTGTTTTTTGTAAAATAAGGAGTGTTATA
TGTTAAGATATGGTGGTTGGTTTTCCAAGATAAGTTTTTTAGTTTGGAGGTATTTTTGTT
TGTTTGTTATTTGTCGCGTTTTTATGATCGGTTAGGAAGAATATAATAATTAGAATTTTT
TATGGTAAAGTTTTATTGTTTATATTTTTTTGGGGTTAGAGTGTAAGAAGTAAGAGAGAG
AGAAAAATGAAATTTTTTTTATTTAAAAGAGAATAATAATTGTTTAGGATGTATTATTTT
TTGATTGGTTGTAGTTTATGGTTGAGTTGATGTTTATGGGAAAAATAGAGTATAAGTAGT
TGTAAATATTTTTGGTTTATGTGTAGATTATTTGTTTATTAATTTAGAATATAGGATGTT
AGCGTTATTTTGTGACGGCGAATGTGGGGGCGGTTTTTTATAGTTTTTTTTTTTTTTTTT
AATAAGAGTAAATAGGTTATTTATATTAATGAGAGTGGAGATAGAGGTTAAATTTTTAGT
GTGTAGGTAAAGGAGTTATGTATAGGATTAGTTTTTAGGTTTATAGGTTTTTATTTAGAG
TAATTTTGATTTGTTTTCGTGTCGTTTTGTTTGGGGGAAGGGAATTAGGATATTGAATTT
TTATGAAAGATGATATGTTTTTTTAGAATAGGTTTATATATGTCGTAGAGTTTTTTTATT
GTAGTGTTTAGTTTTGTAATT
>3LTR-Lung_11
GGGAAGGAAGGTTTTTGTTTTATATTAAAAGTGTGTTTTTTTAAAATAAGGGAGGTTTAT
ATGTTAAGATATGGTGGTTGGTTTTTAAGATAAGTTTTTTAGTTTGGAGGTATTTTTGTT
TGTTTTGTTATTTGTTGTGTTTTTTATGATTTGTTAGGAAGAATATAATAATTAGAATTT
TTTATGGTAAAGTTTTATTGTTTATATTTTTTTGGGGTTAGAGTGTAAGAAGTAAGAGAG
AGAGAAAAATGAAATTTTTTTTATTTAAAAGAGAATAATAATTGTTTAGGATGTATTATT
TTTTGATTGGTTGTAGTTTATGGTTGAGTTGATGTTTATGGGAAAAATAGAGTATAAGTA
GTTGTAAATATTTTTGGTTTATGTGTAGATTATTTGTTTATTAATTTAGAATATAGGATG
TTAGTGTCATTTTGTGATGGTGAATGTGGGGGTGGTTTTTTATAGTTTTTTTTTTTTTTT
TTAATAAGAGTAAATAGGTTATTTATATTAATGAGAGTGGAGATAGAGGTTAAATTTTTA
GTGTGTAGGTAAAGGAGTTATGTATAGGATTAGTTTTTAGGTTTATAGGTTTTTATTTAG
AGTAATTTTGGTTTGTTTTTGTGTTGTTTTGTTTGGGGGAAGGGAATTAGGATATTGAAT
TTTTATGAAAGATGATATGTTTTTTTAGAATAGGTTTATATATGTCGTAGAGTTTTTTTA
TTGTAGTGTTTAGTTTTGTAAT
>3LTR-Lung_12
GGGAAGGAAGTTTTGTTTATATTATAAGTGTGTTTTTGTAAAATAAGGAGTGTTATATGT
TAAGATATGGTGGTTTGGTTTTTAAGATAAGTTTTTTAGTTTGGAGGTATTTTTGTTTGT
TTGTTATTTGTTGTGTTTTTATGATTGGTTAGGAAGAATATAATAATTAGAATTTTTTAT
GGTAAAGTTTTATTGTTTATATTTTTTTGGGGTTAGAGTGTAAGAAGTAAGAGAGAGAGA
AAAATGAAATTTTTTTTATTTAAAAGAGAATAATAATTGTTTAGGATGTATTATTTTTTG
ATTGGTTGTAGTTTATGGTTGAGTTGATGTTTATGGGAAAAATAGAGTATAAGTAGTTGT
AAATATTTTTGGTTTATGTGTAGATTATTTGTTTATTAATTTAGAATATAGGATGTTAGT
GTTATTTTGTGACCGTGGATGTGGGGGTGGTTTTTTATAGTTTTTTTTTTTTTTTTTAAT
AAGAGTAAATAGGTTATTTATATTAATGAGAGTGGAGATAGAGGTTAAATTTTTAGTGTG
TAGGTAAAGGAGTTATGTATAGGATTAGTTTTTAGGTTTATAGGTTTTTATTTAGAGTAA
TTTTGATTTGTTTTTGTGTTGTTTTGTTTGGGGGAAGGGAATTAGGATATTGAATTTTTA
TGAAAGATGATATGTTTTTTTAGAATAGGTTTATATATGTCGTAGAGTCTTTTTATTGTA
GTGTTTAGTTTTGTAATT
Plate B
>3LTR-Lung_1
GGGAAGGAAGTTTTGTTTATATTATTAGTGTGTTTTTGTAAAATAAGGAGTGTTATATGT
TAAGATATGGTGGTTGGTTTTTAAGATAAGTTTTTTAGTTTGGAGGTATTTTTGTTTGTT
TGTTATTTGTTGTGTTTTTATGATTGGTTAGGAAGAATATAATAATTAGAATTTTTTTAT
GGTAAAGTTTTATTGTTTATATTTTTTTGGGGTTAGAGTGTAAGAAGTAAGCGAGAGAGA
AAAATGAAATTTTTTTTATTTAGAAGAGAATAATAATTGTTTAGGATGTATTATTTTTTG
ATTGGTTGTAGTTTATGGTTGAGTTGATGTTTATGGGAAAAATAGAGTATAAGTAGTTGT
AAATATTTTTGGTTTATGTGTAGATTATTTGTTTATTAATTTAGAATATAGGATGTTAGT
GTTATTTTGTGACGGTGAATGTGGGGGTGGTTTTTTATAGTTTTTTTTTTTTTTTTAATA
AGAGTAAATAGGTTATTTATATTAATGAGAGTGGAGATAGAGGTTAAATTTTTAGTGTGT
AGGTAAAGGAGTTATGTATAGGATTAGTTTTTAGGTTTATAGGTTTTTATTTAGAGTAAT
TTTGATTTGTTTTTGTGTTGTTTTGTTTGGGGGAAGGGAATTAGGATATTGAATTTTTAT
GAAAGATGATATGTTTTTTTAGAATAGGTTTATATATGTCGTAGAGTCTTTTTATTGTAG
TGTTTAGTTTTGTAATT
>3LTR-Lung_2
GGGAAGGAAGTTTTGTTTATATTATAAGCGTGTTTCTGTAAAATAAGGGGTGTTATATGT
TAAGATCTGGTGGTTGGTTTTTAAGATAAGTTTTTTAGTTTTGGAGGTATTTTCGTTCGT
TTGTTATTTTGTCGTGTTTTTTATGATTGGTTAGGAAGAATATAATAATTAGAATTTTTT
ATGGCAAAGTTTTATTGTATATATTTTTTCGGGGTTAGAGTGTAAGAAGTAAGAGAGAGA
GAAAAATGAAATTTTTTTTATTTAAAAGAGAATAATAATTGTTTAGGATGTATTATTTTT
TGATTGGTTGTAGTTTATGGTTGAGTTGATGTTTATGGGAAAAATAGAGTATAAGTAGTT
GTAAATATTTTTGGTTTGTGTGTAGATTATTTGTTTATTAATTTAGAATATAGGATGTTA
GTGTTATTTTGTGATGGTGAATGTGGGGGTGGTTTTTTATAGTTTTTTTTTTTTTTAATA
AGAGTAAATAGGTTATTTATATTAATGAGAGTGGAGATAGAGGTTAAATTTTTAGTGTGT
AGGTAAAGGAGTTATGTATAGGATTAGTTTTTAGGTTTATAGGTTTTTATTTAGAGTAAT
TTTGATTTGTTTTCGTGTCGTTTTGTTTGGGGGAAGGAAATTAGGATATTGAATTTTTAT
GAAAGATGATATGTTTTTTTAGAATAGGTTTATATATGTCGTAGAGTTTTTTTATTGTAG
TGTTTAGTTTTGTAATT
>3LTR-Lung_3
GGGAAGGAAGTTTTGTTTATATTATAAGTGTGTTTTTGTAAAATTAAGGAGTGTTATATG
TTAAGATATGGTGGTTGGTTTTTAAGATAAGTTTTTTAGTTCGGAGGTATTTTTGTTTGT
TTGTTATTTGGTTGTGCTTTTATGATTGGTTAGGAAGAATATAATAATTAGAATTTTTTA
TGGTAAAGTTTTATTTTTTATATTTTTTTGGGGTTAGAGTGTAAGAAGTAAGAGAGAGAG
AAAAATGAAATTTTTTTTATTTAAAAGAGAATAATAATTGTTTTGGATGTATTATTTTTT
GATTGGTTGTAGTTTATGGTTGAGTTGATGTTTATGGGAAAAATAGAGTATAAGTAGTTG
TAAATATTTTTGGTTTATGTGTAGATTATTTGTTTATTAATTTAGAATATAGGATGTTAG
TGTTATTTTGTGATGGTGAATGTGGGGGTGGTTTTTTATAGTTTTTTTTTTTTTTTTAAT
AAGAGTAAATAGGTTATTTATATTAATGAGAGTGGAGATAGAGGTTAAATTTCTAGTGTG
TAGGTAAAGGAGTTATGTATAGGATTAGTTTTTAGGTTTATAGGTTTTTATTTAGAGTAA
TTTTGATTTGTTTTCGTGTTGTTTTGTTTGGGGGAAGGGAATTAGGATATTGAATTTTTA
TGAAAGATGATATGTTTTTTTAGAATAGGTTTATATATGTCGTAGAGTTTTTTTATTGTA
GTGTTTAGTTTTGTAATT
>3LTR-Lung_4
GGGAAGGAAAGTTTTTGTTTATATTATAAAGTGTGTTTTTGTAAGATAAGGAGTGTTATA
TGTTAAGATATGGTGGTTGGTTTTTAAGATAAGTTTTTTAGTTTGGAGGTATTTTTGTTT
TGTTTGTTATTTGTTGTGTTTTTATGATTGGTTAGGAAGAATATAATAATTAGAATTTTT
TATGGTAAAGTTTTATTGTTTATATTTTTTGGGGTTAGAGTGTAAGAAGTAAGAGAGAGA
AAAATGAAATTTTTTTTATTTAAAAGAGAATAATAATTGTTTAGGATGTATTATTTTTTT
GATTGGTTGTAGTTTATGGTTGAGTTGATGTTTATGGGAAAAATAGAGTATAAGTAGTTG
TAAATATTTTTGGTTTATGTGTAGATTATTTGTTTATTAATTTAGAATATAGGATGTTAG
TGTTATTTTGTGATGGTGAATGTGGGGGTGGTTTTTTATAGTTTTTTTTTTTTTTTTTAA
TAAGAGTAAATAGGTTATTTATATTAATGAGAGTGGAGATAGAGGTTAAATTTTTAGTGT
GTAGGTAAAGGAGTTATGTATAGGATTAGTTTTTAGGTTTATAGGTTTTTATTTACGAGT
AATTTTGATTTGTTTTCGTGTCGTTTTGTTTGGGGGAAGGGAATTAGGATATTGAATTTT
TATGAAAGATGATATGTTTTTTTAGAATAGGTTTATATATGTCGCAGAGTTTTTTTATTG
TAGTGTTTAGTTTTGTAATT
>3LTR-Lung_7
TAAGAAGTAAGAGAGAGAGAAAAATGAAATTTTTTTTATTTAAAAGAGAATAATAATTGT
TTAGGCCGTATTATTTTTTGATTGGTTGTAGTCTATGGTTGAGTTGATGTTTGTGGGAAA
AATAGAGTATAAGTAGTTGTAAATATTTCTGGTTTATGTGTAGATTATTTGTTTATTAAT
TTAGAATATAGGATGTTAGTGTTATTTTGTGATGGTGAATGTGGGGGTGGTTTTTTATAG
TTTTTTTTTTTTTTTTTAATAAGAGTAAATAGGTTATTTATATTAATGAGAGTGGAGATA
GAGGTTAAATTTTTAGTGTGTAGGTAAAGGAGTTATGTATAGGATTAGTTTTTAGGTTTA
TAGGTTTTTATTTAGAGTAATTTCGATTTGTTTTTGTGTTGTTTTGTTTGGGGGAAGGGA
ATTAGGATATTGAATTTTTATGAAAGATGATATGTTTTTTTAGAATAGGTTTATATATGT
CGTAGAGTTTTTTTATTGTAGTGTTTAGTTTTGTAATT
>3LTR-Lung_5
TAAGAAGTACGAGAGAGAGAAAAATGAATTTTTTTTTATTTAAAAGAGAATAATAATTGT
TTAGGATGTATTATTTTTTGATTGGTTGTAGTTTATGGTTGAGTTCATGTTTTATGGGAA
AAATAGAGTATAAGTAGTTGTAAATATTTTTTGGTTTATGTGTAGATTATTCGTTTTTTA
ATTTAGAATATAGGATGTAAGTGTTATTTTGTGATGGCGAATGTGGGGGGGGTTTTTTAT
AGTTTTTTTTTTTTTTTTAATAAGAGTAAATAGGTTATTTATATTAATGAGAGTGGAGAT
AGAGGTTAAATTTTTAGTGTGTAGGTAAAGGAGTTATGTATAGGATTAGTTTTTAGGTTT
ATAGGTTTTTATTTAGAGTAATTTTGCCTTGTTTTTGTGTTGTTTTGCTCGGGGGAAGGG
AATTAGGATATTGAATTTTTATGAAAGATGATATGTTTTTTTAGAATAGGTTTATATATG
TCGTAGAGTTTTTTTATTGTAGTGTTTAGTTTGTAATT
>3LTR-Lung_9
TAAGAAGTAAGAGAGAGAGAAAAATGAAATTTTTTTTATTTTAAAGAAGAATAATAATTG
TCTAGGATGTATTATTTTTTGAATTGGTTGTAGTTTATGGTTGAGCTGGTGTTTATGGGA
AAAACAGAGTATAAGTAGTTGTAAGTATTTTTGGTTTACGCGTAGATTATTTGTTTATTA
ATTTAGAATATAGGATGTTAGTGTTATTTTGTGATGGTGAATGTGGGGGTGGTTTTTTAT
AGTTTTTTTTTTTTTTTAATAAGAGTAAATAGGTTATTTATATTAATGAGAGTGGAGATA
GAGGTTAAATTTTTAGTGTGTAGGTAAAGGAGTTATGTATAGGATTAGTTTTTAGGTTTA
TAGGTTTTTATTTAGAGTAATTTTGATCTGTTTTCGTGTCGTTTTGTTTGGGGGAAGGGA
ATTAGGATATTGAATTTTTATGAAACGATGATATGTTTTTTTAGAATAGGTTTATATATG
TTGTAGGAGTTTTTTTATTGTAGTGTTTAGTTTTGTAATT
>3LTR-Lung_8
GAAGTAAGAGAGAGAGAAAAATGAAATTTTTTTTATTTAAAAGAGAATAATAATTGTTTA
GGACGTATTATTTTTTGATTGGTTGTAGTTTATGGTTGAGTTGATGTTTTATGGGAAAAA
TAGAGTATAAGTAGTTGTAAATATTTTTGGTTTATGCGTAGATTATTTGTTTATTAATTT
AGAATATAGGATGTTAGTGTTATTTTGTGACGGTGGATGTGGGGGTGGTTTTTTATAGTT
TTTTTTTTTTTTTTAATAAGAGTAAATAGGTTATTTATATTAATGAGAGTGGAGATAGAG
GTTAAATTTTTAGTGTGTAGGTAAAGGAGTTATGTATAGGATTAGTTTTTAGGTTTATAG
GTTTTTATTTAGAGTAATTTTGATTTGTTTTTGTGTTGTTTTGTTTGGGGAAAGGGAATT
AGGATATTGAATTTTTATGAAAGATGATATGTTTTTTTAGAATAGGTTTATATATGTCGT
AGAGTCTTTTTATTGTAGTGTTTAGTTTTGTAATT
	
