# Supplementary material for: Epigenetic interplay between mouse endogenous retroviruses and host genes
Source: Genome Biol. 2012 Oct 3;13(10):R89. doi: 10.1186/gb-2012-13-10-r89 (PMC3491417; doi:10.1186/gb-2012-13-10-r89)
Supplement: Additional file 4 — All bisulfite sequencing data. Compilation of all bisulfite sequences. [file gb-2012-13-10-r89-S4.zip › IAP7083_TE_brain.rtf]

Polymorphic IAP Case 42
Chr 17
3'LTR Miniprep Sequences B6AJ Brain
>42RLTRBr_74
TTTATTTGTTTTTATTAAAAGGATAGGGGGAGATGTTGGGAGTCGCGTTTATTTTTGTCG
TTATAAGAAGGCGTTGATAGTTGTGTTTTAAGTGGTAAATAAATAATTTGCGTATGTGTT
AAGGGTATTTTATGATTATTTGTGTTTTGTTTTTTTCGTGACGTTAATTCGGTCGATGGG
TTGTAGTTAATTAGGGAATGATACCTTCGAGGCGAAGGAGAATGTTTTTTAAGAGGGATG
GGGTTTCGTTTTTTTTTTTTGTTTTTCGTTTTTTTTTTTGTTTTTTCGTTTTTTTGTTTT
TTTTTTTTTTGTTTCGTTTTTTTGTTTCGTTTTTTTGTTTTTTTTTTGTTTTTTTTTTTG
TTTTTTTTTCTTTTGTTTTTTGTTTTTTTGTTTTTTGTTTTTTTGTTTTTTATACGTTTG
TTTTTGAAGATGTAAGAAATAAAGTTTTGTCGTAGAAGATTTTGGTTTGTGGTGTTTTTT
TTGGTCGGTCGTGAGAACGCGTTTAATAATAAGATAGTATCGGGTAGGTTTTTAGTTGTT
TTGATGGATATTTTTGTAGTTTTTTTAATGGAGGTTTTATAGTAGAG
>42RLTRBr_77
GTGGTTTATTTGTTTTTATTAAAAGGATAGGGGGAGATGTTGGGAGTCGCGTTTATATTC
GTTGTTATAAGAAGGCGTTGATAGTTGTGTTTTAAGTGGTAAATAAATAATTTGCGTATG
TGTTAAGGGTATTTTATGATTATTTGTGTTTTGTTTTTTTCGTGACGTCAATTCGGTCGA
TGGGTTGCAGTTAATTAGGGAATGATATGTTCGAGGCGAAGGAGAATGTTTTTTAAGAGG
GACGGGGTTTCGTTTTTTTTTTTTTTGTTTTTCGTTTTTTTTTTTGTTTTTTCGTTTTTT
TGTTTTTTTTTTTTTTGTTTCGTTTTTTTGTTTCGTTTTTTTGTTTTTTTTTTTGTTTTT
TTTTTTGTTTTTCTTTTTTTTGCTTTTTGTTTTTTTGTTTTTTGCTTTTTTGTTTTTTAT
ACGTTTGTTTTTGAAGATGTAAGAAATAAAGTTTTGTCGTAGAAGATTTTGGTTTGTGGT
GTTTTTTTTGGTCGGTCGTGAGAACGCGTTTAATAATAAGATAGTATCGGGTAGGTTTTT
AGTTGTTTTGACGGATATTTTTGTAGTTTTTTTAATGGAGG
>42RLTR_42
GTGGTTTATTTGTTTTTAATTAAAAGGATAGGGGGAGATGTTGGGAGTCGCGTTTATATT
CGCCGTTGTAAGATGGCGTTGATAGTTGTGTTTTAAGTGGTAAATAAATAATTTGCGTAT
GTGTCAAGGGTATTTTATGATTATTTGTGTTTTGTTTTTTTCGTGACGTTAATTCGGTCG
ATGGGTTGTAGTTAATTAGGGAGTGATACGTTCGAGGCGAAGGAGAATGTTTTTTAAGAG
GGACGGGGTTTCGTTTTTTTTTTTTTTTTGTTTTTTGCGTTTTTGTTTTTTGCTTTTTTG
TTTTTTGTATTTTTGTTTTTGAAGATGTAAGAAATAAAGTTTTGTCGTAGAAGATTTTGG
TTTGTGGTGTTTTTTTTGGTCGGTCGTGAGAACGCGTTTAATAATAAGATAGTATCGGGT
AGGTTTTTAGTTGTTTTGATGGATATTTTTGTAGTTTTTTTAATGGAGGTTTTATAGTAG
AG
>42RLTR_43
GTGGTTTATTTGTTTTTATTAAAAGGATAGGGGGAGATGTTGGGAGTCGCGTTTATTTTT
GTCGTTATAAGAAGGCGTTGATAGTTGTGTTTTAAGTGGTAAATAAATAATTTGCGTATG
TGTTAAGGGTATTTTATGATTGTTTGTGTTTTGTTTTTTTCGTGATGTTAATTCGGTCGA
TGGGTTGTAGTTAATTAGGGAATGATACCTTCGAGGCGAAGGAGAATGTTTTTTTAGAGG
GACGGGGTTTCGTTTTTTTTTTTTTGTTTTTCGTTTTTTTTTTGTTTTTTCGTTTTTTTG
TTTTTTTTTTTTTGTTTCGTTTTTTTGTTTCGTTTTTTTGTTTTTTTTTTTGTTTTTTTT
TTGTTTTTTTTTTTTGTTTTTTGTTTTTTTGTTTTTTGTTTTTTTGTTTTTTATACGTTT
GTTTTTGAAGATGTAAGAAATAAAGTTTTGTCGTAGAAGATTTTGGTTTGTGGTGTTTTT
CTTGGTCGGTCGTGAGAACGCGTTTAATAATAAGATAGTATCGGGTAGGTTTTTAGTTGT
TTTGACGGATATTTTTGTAGTTTTTTTAATGGAGGTTTTATAGTAGAG
>42RLTR_49
GTGGTTTATTTGTTTTTATTAAAAGGATAGGGGGAGATGTTGGGAGTCGCGTTTATGTTT
GTCGTTATAAGATGGCGTTGATAGTTGTGTTTTAAGTGGTAAATAAATAATTTGCGTATG
TGTTAAGGGTATTTTATGATTATTTGTGTTTTGTTTTTTTCGTGACGTTAATTCGGTCGA
TGGGTTGTAGTTAATTAGGGAATGATACGTTTGAGGCGAAGGAGAATGTTTTTTAAGAGG
GACGGGGTTTTGTTTTTTTTTTTTTTGTTTTTCGTTTTTTTTTTGTTTTTTCGTTTTTTT
GTTTTTTTTTTTTTTGTTTCGTTTTTTTGTCTCGTTTTTTTGTTTTTTTTTTGTTTTTCT
TTTTTGTTTTTTTTTCTCGTTTTTTGTTTTTTTGTTTTTTGTTTTTTTGTTTTTTATACG
TTTGTTTTTGAAGATGTAAGAAATAAAGTTTTGTCGTAGAAGATTTTGGTTTGTGGTGTT
TTTTTTGGTCGGTCGTGAGAACGCGTTTAATAATAAGATAGTATCGGGTAGGTTTTTAGT
TGTTTTGATGGATATTTTTGTAGTTTTTTTAATGGAGGTTTTATAGTAGAG
>42RLTRBr_42
GTGGTTTATTTGTTTTTATTAAAGGATAGGGGGAGAAGTTGGGAGTCGCGTTTATATTTG
TCGTTATAAGATGGCGTTGATAGTTGTGTTTTAAGTGGTAAATAAATAATTTGGGTATGT
GTTAAGGGTATTTTATGATTACTTGTGTTTTGTTTTTTTCGGGACGTTAATTCGGTCGAT
GGGTTGTAGTTAATTAGGGAATGATACCTTCGAGGCGAAGGAGAATGTTTTTTAAGAGGG
ACGGGGTTTCGTTTTTTTTTTTTGTTTTTCGTTTTTTTTTTGTTTTTTCGTTTTTTTGTT
TTTTTTTTTTTTTGTTTCGTTTTTTTGTTTCGTTTTTTTGTTTTTTTTTTTGTTTTTTTT
TTTTGTTTTTTTTTTTTTTGTTTTTTGTTTTTTTGTTTTTTGTTTTTTTGTTTTTTATAT
GTTTGTTTTTGAAGATGTAAGAAATAAAGTTTTGCTGTAGAAGATTTTGGTTTGTGGTGT
TTTTTTTGGTTGGTTGTGAGAACGTGTTTAATAATAAGATAGTATCGGGTAGGTTTTCAG
TTGTTTTGATGGATATTTTTGTAGTTTTTTTTAATGGAGGTTTTAATAGTAGAG
>42RLTRBr_43
GTGGTTTATTTGTTTTTATTAAAAAGATAGGGGGAGATGTTGGGAGTCGCGTTTATATTT
GTCGTTATAAGAAGGCGTTGATAATTGTGTTTTAAGTGGTAAATAAATAATTTGCGTATG
TGTTAAGGGTATTTTATGATTATTTGTGTTTTGTTTTTTTCGTGACGTTAATTCGGTCGA
TGGGTTGTAATTAAATAGGGAATGATACCTTCGAGGGGAAGGAGAATGTTTTTTAAGAGG
GACGGGGTTTCGTTTTTTTTTTTTTTGTTTTTCGTTTTTTTTTTTGTTTTTTTGATTTTT
TGTTTTTTTTTTTTTTTGTTTCGTTTTTTTGTTTCGTTTTTTTGTTTTTTTTTTTGTTTT
TTTTTTGTTTTTTTTTTTTTTGTTTTTTGTTTTTTTGTTTTTTGTTTTTTTGTTTTTTAT
ACGTTTGTTTTTGAAGATGTAAGAAATAAAGTTTTGTCGTAGAAGATTTTGGTTTGTGGT
GTTTTTTTTGGTCGGTCGTGAGAACGCGTTTAATAATAAGATAGTATCGGGTAGGTTTTT
AGTTGTTTTGACGGATATTTTTGTAGTTTTTTTAATGGAGGTTTTATAGTAGAG
>42RLTRBr_44
GTGGTTTATTTGTTTTTATTAAAAGGATAGGGGGAGATGGTGGGAGTCGCGTTCATGTTT
GTCGTTATAAGAAGGCGTTGATAGTTGTGTTTTAAGTGGTAAATAAAAAATTTGCGTATG
TGTCAAGGGTATTTTAAGATTATTTGTGTTTTGGTTTTTTCGTGACCTTAATTCGGTCGA
TGGGTTGTAGTTAATTAGGGAATGGTACCTTCGAGGCGAAGGAGGATGTTTTTTTAGAGG
GATGGGGGTTCGGTTTTTTTTTTTTGTTTTTCGTTTTTTTTTTTGTTTTTTCGTTTTTTT
GTTTTTTTTTTTTCTTTGTTTCGTTTTTTTGTTTCGTTTTTTTGTTTTTTTTTTTTGTTT
TTTTTTTTTGTTTTTTTTTTTTTTGTTTTTTGTTTTTTTGTTTTTTGTTTTTTTGTTTTT
TATACGTTTGTTTTTGAAGATGTAAGAAATAAAGTTTTGTCGTAGAAGATTTTGGTTTGT
GGTGTTTTTTTTGGTCGGTTGTGAGAACGCGTTTAATAATAAGATAGTATCGGGTAGGTT
TTTAGTTGTTCTGATGGATATTTTTGTAGTTTTTTTAATGGAGGTTTTATAGTAGAG
>42RLTRBr_56
GTGGTTTATTTGTTTTTATTAAAAGGATAGGGGGAGATGTTGGGAGTCGCGTTTATTTTT
GTCGTTATAAGAAGGCGTTGATAGTTGTGTTTTAAGTGGTAAATAAATAATTTGCGTATG
TGTTAAGGGTATTTTATGATTATTTGTGTTTTGTTTTTTTCGTGACGTTAATTCGGTCGA
TGGGTTGTAGTTAATTAGGGAATGATACCTTCGAGGCGAAGGGGAATGTTTTTTAAGAGG
GACGGGGTTTCGTTTTTTTTTTTTTTGTTTTTCGTTTTTTTTTTGTTTTTTCGTTTTTTT
GTTTTTTTTTTTGTTTCGTTTTTTTGTTTCGTTTTTTTGTTTTTTTCTTTTGTTTTTTTT
TTTGTTTTTTTTTTTTGTTTTTTGTTTTTTTGTTTTTTGTTTTTTTGTTTTTTATACGTT
TGTTTTTGAAGATGTAAGAAATAAAGTTTTGTCGTAGAAGATTTTGGTTTGTGGTGTTTT
TTTTGGTCGGTCGTGAGAACGCGTTTAATAATAAGATAGTATCGGGTAGGTTTTTAGCTG
TTTTGACGGATATTTTTGTAGTTTTTTTAATGGAGGTTTTATAGTAGAG
>42RLTRBr_63
GTGGTTTATTTGTTTTTTTTAAAAGGATAGGGGGAGATGTTGGGAGTCGCGTTTATTTTT
GTCGTTATAAGAAGGCGTTGATAGTTGTGTTTTAAGTGGTAAATAAATAATTTGTGTATG
TGTTAAGGGTATTTTATGATTATTTGTGTTTTGTTTTTTTCGTGATGTTAATTCGGTCGA
TGGGTTGTAGTTAATTAGGGAATGATACGTTTGAGGCGAAGGAGAATGTTTTTTAAGAGG
GACGGGGGTTCGTTTTTTTTTTTTTGTTTTTTGTTTTTTTTTTTGTTTTTTCGTTTTTTT
GTTTTTTTTTTTTTGTTTCGTTTTTTTGTTTCGTTTTTTTGTTTTTTTTTTTTGTTTTTT
TTTTTTGTTTTTTTTTTTTTGTTTTTTGTTTTTTTGTTTTTTGTTTTTTTGTTCTTTATA
CGTTTGTTTTTGAAGATGTAAGAAATAAAGTTTTGTCGTAGAAGATTTTGGTTTGTGGTG
TTTTTTTTGGTCGGTCGTGAGAACGCGTTTAATAATAAGATAGTATCGGGTAGGTTTTTA
GTTGTTTTGATGGATATTTTTGTAGTTTTTTTAATGGAGGTTTTATAGTAGAG
>42RLTRBr_65
GTGGTTTATTTGTTTTTATTAAAAGGATAGGGGGAGAAGTTGGGAGTTGCGTTTATATTT
GTCGTTATAAGAAGGCGTTGATAGTTGTGTTTTAAGTGGTAAATAAATAATTTGCGTATG
TGTTAAGGGTATTTTATGATTATTTGTGTTTTGCTTTTTTTGTGACGTTAAATCGGTCGA
TGGGTTGTAATTAATTAGGGAATGATACCTTTGAGGCGAAGGAGAATGTTTTTTAAGAGG
GACGGGGTTTCGTTTTTTTTTTTTTGTTTTTCGTTTTTTTTTCTTGTTTTTTCGTTTTTT
TGTTTTTTTTTTTTTTTTTGTTTCGTTTTTTTGTTTCGTTTTTTTGTTTTTTTTTTTGTT
TTTTTTTTTGTTTTTTTTTTTTGTTTTTTGTTTTTTTGTTTTTTGTTTTTTTGTTTTTTA
TATGTTTGTTTTTGAAGATGTAAGAAATAAAGTTTTGCTGTAGAAGATTTTGGTTTGTGG
TGTTTTTTTTGGTTGGTTGTGAGAACGTGTTTAATAATAAGATAGGATCGGGTAGGTTTT
CAGTTGTTTTGATGGATATTTTTGTAGTTTTTTTAATGGAGGTTTTTATAGTAGAG
>42RLTRBr_70
GTGGTTTATTTGTTTTTATTAAAAGGATAGGGGGAGATGTTGGGAGTCGCGTTTATATTT
GTCGTTATAAGAAGGCGTTGATAGTTGTGTTTTAAGTGGTAAATAAATAATTTGCGTATG
TGTTAAGGGTATTTTATGATTATTTGTGCTTTGTTTTTTTCGTGATGTTAATTCGGTCGA
TGGGTTGTAGTTAATTAGGGAATGATACGTTCGAGGCGAAGGAGAATGTTTTTTAAGAGG
GACGGGGTTTCGTTTTTTTTTTTGTTTCTTGTTTTTTTTTTTGTTTTTTCGTTTTTTTGT
TTTTTTTTTTTTTTGTTTCGTTTTTTTGTTTCGTTTTTTTGTTTTTTTTTTTGTTTTTTT
TTTTTGTTTTTTTTTTTTGTTTTTTGTTTTTTTGTTTTTTGTTTTTTTGTTTTTTATACG
TTTGTTTTTGAAGATGTAAGAAATAAAGTTTTGTTGTAGAAGATTTTGGTTTGTGGTGTT
TTTTTTGGTTGGTTGTGAGGATGTGTTTAATAATAAGATAGTATCGGGTAGGTTTTTAGT
TGTTTTGATGGATATTTTTGTAGTTTTTTTAATGGAGGTTTTATAGTAGAG
