# Supplementary material for: Epigenetic interplay between mouse endogenous retroviruses and host genes
Source: Genome Biol. 2012 Oct 3;13(10):R89. doi: 10.1186/gb-2012-13-10-r89 (PMC3491417; doi:10.1186/gb-2012-13-10-r89)
Supplement: Additional file 4 — All bisulfite sequencing data. Compilation of all bisulfite sequences. [file gb-2012-13-10-r89-S4.zip › IAP7083_TE_oppositeLTR_brain.rtf]

Polymorphic IAP Case 42
Chr 17
5' LTR B6AJ Brain Miniprep Sequences
>42LLTRBr_83
GTTTTGATTAGATTTTTTTTTTATTATGTTTCGGGAGTTAGGTGAGTATTGAGGATAGAT
AGTGTTGGGAGTCGCGTCTATTTTTGCCGTTATAAGAAGGCGTTGATAGTTGTGTTTTAA
GTGGTAAATAAATGATTTGCGTATGTGTTAAGGGTATTTTATGATTATTTGTGTTTTGTT
TTTCTCGTGACGTTAATTCGGTCGATGGGTTGTAGTTAATTAGGGAATGATACCTTCGAG
GCGAAGGAGAATGTTTTTTAAGAGGGACGGGGTTTCGTTTTTTTTTTTTTTGTTTTTCGT
TTTTTTTTTGTTTTTTCGTTTTTTTGTTTTTTTTTTTTTGTTTCGTTTTTTTGTTTCGTT
TTTTTGTTTTTTTTTTTTGTTTTTTTTTTTTGTTTTTTTTTTTTTGTTTTTTGTTTTTTT
GTTTTTTGTTTTTTTGTTTTTTATACGTTTGTTTTTGGAGATGTAAGAAATAAAGTTTTG
TCGTAGAAGATTTTGGTTTGTGGTGTTTTTTTTGGTCGGTCGTGAGAACGCGTTTAATAA
TAATTGGTGTCGAATTTCGGGACGAGAAAAAAAAATTCGGGATTGGCGTAAGGAGGATTT
TTTATTTCGGAATTAGAATTGCGGATTACGTTTATAAAGGTTTTCGTAATATAGATTGTT
GAGAAGGATTTAATTGTCGAATTTAGAATTTATTAGTTGGGGGACGACGGTGATAAAGGT
TTTCGTAAAGTAGATTGTTAAGAAGGATTTAATTGTATGAATTTAGAATTTTTTAGTTGG
GG
>42LLTRBr_14
GTTTGAATTAGATTTTTTTTTTTATTTATGTTTCCGGAAGTTAGGTGAGTATTTGAGGAT
AGAATAGTGTTGGGAGTCGCGTTTATATTTGTTGTTATTAAGAAGGCGCTGATAGTTTGT
GTTTTAAGTGGTAAATAAATAATTTGCGTATGTTTAAAGGGTATTTTATGATTATTTGTG
TTTTGTTTTTTTCGTGACGTTAATTCGGTCGATGGGTTGTAGTTAATTAGGGAATGATAC
GTTCGAGGCGAAGGAGAATGTTTTTTAAGAGGGACGGGGTTTCGTTTTTTTTTTTTTTTG
TTTTTAGTTTTTTTTTTGTTTTTTCGTTTTTTTGTTTTTTTTTTTTTGTTTCGTTTTTTG
TTTCGTTTTTTTGTTTTTTTTTTGTTTCTTTTTTTTGTTTTTTTTTTTTTTGTTTTTTGT
TTTTTTGTTTTTTGTTTTTTTGTTTTTTATACGTTTGTTTTTGAAGATGTAAGAAATAAA
GTTTTGTCGTAGAAGATTTTGGTTTGTGGTGTTTTTTTTGGTCGGCCGTGAGAACGCGTT
TAATAATAATTGGTGTCGAATTTCGGGACGAGAAAAAAAAAATTCGGGATTGGCGTAAGG
AGGATTTTTTATTTCGGAATTAGAATTGCGGATTACGTTTATAAAGGTTTTCGTAATATA
GATTGTTGAGAAGGATTTAATTGTCGAATTTAGAATTTATTAGTTGGGGAACGACGGTGA
TAAAGGTTTTCGTAAAGTAGATTGTTAAGAAGGATTTAATTGTATGAATTTAGAATTTTT
TAGTTGGGGAACGACGGTGATAAAGGTTTTCGTAAAGTAGATTGTTAAGAAGGATTTAAT
TGTATGAATTTAGAATTTTTTAGTTGGGG
>42LLTRBr_15
GTTTGATTAGATTTTTTTTTTTAATTATGTTTCGGGAGTTAGGTGAGTATTGAGGATAGA
TAGTGTTGGGAGTCGCGTTTATATTTGTTGTTATAAGATGGCGCTGATAGTTGTGTTTTA
AGTGGTAAATAAATAATTTGCGTATGTGTTAAGGGTATTTTATGATTATTTGTGTTTTGT
TTTTTTCGTGACGTTAATTCGGTCGATGGGTTGCAGTTAATTAGGGAATGATACGTTCGA
GGCGAAGGAGAATGTTTTTTTAGAGGGACGGGGTTTCGTTTTTTTTTTTTGTTTTTCGTT
TTTTTTTTTGTTTTTTCGTTTTTTTGTTGTTTTTTTTTTTTTGTTTCGTTTTTTTGTTTC
GTTTTTTTGTTTTTTTTTTTTGTTTCTCTTTTTTGTTTTTTTTTTTTTGTTTTTTGTTTT
TTTGTTTTTTGTTTTTTTGTTTTTTATACGTTTGTTTTTGAAGATGTAAGAAATAAAGTT
TTGTCGTAGAAGATTTTGGTTTGTGGTGTTTTTTTTGGTCGGTCGTGAGAACGCGTTTAA
TAATAATTGGTGTCGAATTTCGGGACGAGAAAAAAAAATTTGGGATTGGCGTAAGGAGGA
TTTTTTATTTCGGAATTAGAATTGCGGATTATGTTTACAAAGGTTTTCGTAATGTAGACT
GTTGAGAAGGATTTAATTGTCGAATTTAGAATTTATTAGTTGGGGAACGACGGTGATAAA
GGTTTTCGTAAAGTAGATTGTTAAGAAGGATTTAATTGTATGAATTTAGAATTTTTTAGT
TGGGG
>42LLTRBr_16
GTTTGATTAGATTTTTTTTTTATTATGTTTCGGGAGTTAGGTGAGTATTGAGGATAGATA
GTGTTGGGAATCGCGTTTTATATTTCGTTGTTTATAAGAAGGGCGTTGATAGTTGTGTTT
TAAGTGGTAAATAAAATAATTTGCGTATGTGTTAAGGGTATTATACGATTATTTGTGTTT
TGTTTTTTTCGTGAAGTTAAATCGGTCGATGGGTTGTAGTTAATTAAGGAATGATACGTT
CGAGGCGAAGGAGAATGTTTTTTAAGAGGGACGGGGTTTCGTTTTTTTTTTTTTTTTGTT
TTTCGTTTTTTCTTTTGTTTTTTCGTTTTTTTGTTTTTTTTTTTTTTGTTTCGTTTTTTG
TTTCGTTTTTTTGTTTTTTTTTTGTTTCTTTTTTTTGTTTTTTTTTTTTTGTTTTTTGTT
TTTTTGTTTTTTGTTTTTTTGTTTTTTATACGTTTGTTTTTGAAGATGTAAGAAATAAAG
TTTTGTCGTAGAAGATTTTGGTTTGTGGTGTTTTTTTTGGTCGGTCGTGAGAACGCGTTT
AATAATAATTGGTGTCGAATTTCGGGACGAGAAAAAAAAATTCGGGATTGGCGTAAGGAG
GATTTTTTATTTCGGAATTAGAGCTGCGGATTACGTTTATAAAGGTTTTCGTAATATAGA
TTGTTGAGAAGGATTTAATTGTCGAATTTAGAATTTATTAGTTGGGGAACGACGGTGATA
AAGGTTTTCGTAAAGTAGATTGTTAAGAAGGATTTAATTGTATGAATTTAGAATTTTTTA
GTTGGGG
>42LLTRBr_17
GTTTGATTAGATTTTTTTTTTTATTATGTTTCGGGAATTAGGTGAGTATTGAGGATAGAT
AATGTTGGGAGTCGCGTTTATATTTGTCGTTATAAAAAAGGCGTTTGATAGTTGTGTTTT
AAATGGTAAATAAATAATTTGCGTATGTGTTAAGGGTATTTTAAGATTATTTGTGTTTTG
TTTTTTTCGGGACGTTAATTCGGCCGATGGGTTGTAGTTAATTAGGGAATGATACGTTCG
AGGCGAAGGAGAATGTTTTTTAAGAGGGACGGGGTTTCGTTTTTTTTTTTTTTTGTTTTT
CGTTTTTTTTTGTTTTTTCGTTTTTTTGTTTTTTTTTTTTTTTTGTTTCGTTTTTTTGTT
TCGCTTTTTTGTTTTTTTTTTTGTTTTTTTTTTTGTTTTTTTTTTTTTTGTTTTTTGTTT
TTTTGTTTTTTGTTTTTTTGTTTCTTATACGTTTGTTTTTGAAGATGTAAGAAATAAAGT
TTTGTCGTAGAAGATTTTGGTTTGTGGTGTTTTTTTTGGTCGGTCGTGAGAACGCGTTTA
ATAATAATTGGTGTCGAATCTCGGGACGAGAAAAAAAAATTCGGGATTGGCGTAAGGAGG
ATTTTTTATTTCGGAATTAGAATTGCGGATTACGTTTATAAAGGTTTTCGTAATATAGAT
TGTTGAGAAGGATTTAATTGTCGAATTTAGAATTTATTAGTTGGGGAACGACGGTGATAA
AGGTTTTCGTAAAGTAGATTGTTAAGAAGGATTTAATTGTATGAATTTAGAATTTTTTAG
TTGGGG
>42LLTRBr_18
GATTAGATTTTTTTTTTTTATTATGTTTCGGGAATTTAGGGGGAGTATTGAGGATAGATA
GAGGTTGGGGAGTCGCGGTTTTTTTCGTTGTTATAAGAAGGCGTGGAAATTGTGTTTTAA
GTGGTAAATAAATAATTTGCGTATGTGTTAAAGGTATTTTATGATTAATTGTGTTTTGTG
TTTTTCCTGACCTTAATTCGGTCGATGGGTTGTAGTTTATTAGGGAATTATATGTTCGAG
GCGAAGGAGAATGTTTTTTAAAAGGGACGGGGGTTCCATTTTTTTTTTTTTTGTTTTTCG
TTTTTTTTTGTTTTTTCGTTTTTTTGTTTTTTTTTTTTTTTTGTTTCGTTTTTTTGTTTC
GTTTTTTTGTTTTTTTTTTTAGTTTTTTTTTTTTGTTTTTTTTTTTTTGTTTTTTGTTTT
TTTGTTTTTTGTTTTTTTGTTTTTTATACGTTTGTTTTTGAAGATGTAAGAAATAAAGTT
TTGTCGTAGAAGATTTTGGTTTGTGGTGTTTTTTTTGGTTGGTCGTGAGAACGCGTTTAA
TAATAATTGGTGTCGAATTTCGGGACGAGAAAAAAAAATTCGGGATTGGCGTAAGGAGGA
TTTTTTATTTCGGAATTAGAATTGCGGATTACGTTTATAAAGGTTTTCGTAATATAGATT
GTTGAGGAGGATTTAATTGTCGAATTTAGAATTTATTAGTTGGGGAACGACGGTGATAAA
GGTTTTCGTAAAGTAGATTGTTAAGAAGGATTTAATTGTATGAATTTAGAATTTTTTAGT
TGGGG
>42LLTRBr_20
GTTTGATTAGATTTTTTTTTTTATTTATGTTTCGGGAGTTAGGTGAGTATTGAGGATAGA
TAATGTTGGGAGTCGCGTTTATATTCGTTGTTATAAGAAGGCGTTGATAGTTGTGTTTTA
AGTGGTAAATAAATAATTTGCGTATGTGTTAAGGGTATTTTATGATTATTTGTGTTTTGT
TTTTTTCGTGATGTTAATTCGGTCGATGGGTTGTAGTTAATTAGGGAATGATACCTTCGA
GGCGAAGGAGAATGTTTTTTTAGAGGGACGGGGGTTCGTTTTTTTTTTTTTTTGTTTTTC
GTTTTTTCTTTTGTTTTTTCGTTTTTTTGTTTTTTTTTTTTTTGTTTCGTTTTTTTGTTT
CGTTTTTTTGTTTTTTTTTTTTTTGTTTTTTTTTTTGTTTTTTTTTTTTTTTTGTTTTTT
GTTTTTTTGTTTTTTGTTTTTTTGTTTTTTATACGTTTGTTTTTGAAGATGTAAGAAATA
AAGTTTTGTCGTAGAAGATTTTGGTTTGTGGTGTTTTTTTTGGTCGGTCGTGAGAACGCG
TTTAATAATAATTGGTGTCGAATTTCGGGACGAGAAAAAAAAATTCGGGATTGGCGTAAG
GAGGATTTTTTATTTCGGAATTAGAATTGCGGATTACGTTTATAAAGGTTTTCGTAATAT
AGATTGTTGAGAAGGATTTAATTGTCGAATTTAGAATTTATTAGTTGGGGAACGATGGTG
ATAAAGGTTTTCGTAAAGTAGATTGTTAAGAAGGATTTAATTGTATGAATTTAGAATTTT
TTAGTTGGGG
>42LLTRBr_22
GTTTGAATAGATTTTTTTTTTATTATGTTTCGGGAGTTAGGTGAGTATTGAGGATAGATA
GTGTTGGGAGTCGCGTTTATTTTTGTCGTTATAAGAAGGCGTTGATAGTTGTGTTTTAAG
TGGTAAATAAATAATTTGCGTATGTGTTAAGGGTATTTTATGATTATTTGTGTTTTGTTT
TTTTCGGGACGTTAATTCGGTCGATGGGTTGTAGTTAATTAGGGAATGATACCTTCGAGG
CGAAGGAGAATGCTTTTTAAGAGGGGCGGGGGTTCGTTTTTTTTTTTTTTTGTTTTTCGT
TTTTTTTTTTGTTTTTTCGTTTTTTTGTTTTTTTTTTTTTTTGTTTCGTTTTTTTGTTTC
GTTTTTTTGTTTTTTTTTTTTGTTTTTTTTTTTGTTTTTTTTTTTTTTGTTTTTTGTTTT
TTTGTTTTTTGTTTTTTTGTTTTTTATACGTTTGTTTTTGAAGATGTAAGAAATAAAGTT
TTGTCGTAGAAGATTTTGGTTTGTGGTGTTTTTTTTGGTCGGTCGTGAGAACGCGTTTAA
TAATAATTGGTGTCGAACTTCGGGACGAGAAAAAAAAATTCGGGATTGGCGTAAGGAGGA
TTTTTTATTTCGGAATTAGAATTGCGGATTACGTTTATAAAGGTTTTCGTAATATAGATT
GTTGGGAAGGATTTAATTGTCGAATTTAGAATTTATTAGTTGGGGAACGATGGTGATAAA
GGTTTTCGTAAAGTAGATTGTTAAGAAGGATTTAATTGTATGAATTTAGAATTTTTTAGT
TGGGG
>42LLTRBr_24
GTTTGATTAGATTTTTTTTTTTTTATTATTGTTTTCGGGAGTTAGGTGAGTATTGAGGAT
AGATAGTGTTGGGAGTCGCGTTTATATTTGTCGTTATAAGATGGCGTTGATAGTTGTGTT
TTAAGTGGTAAATAAATAATTTGCGTATGTGTTAAGGGTATTTTATGATTATTTGTGTTT
TGTTTTTTTCGGGACGTTAATTCGGTCGATGGGTTGTAGTTAATTAGGGAATGATACCTT
CGAGGCGAAGGAGAATGCTTTTTAAGAGGGACGGGGTCTCGTTTTTTTTTTTTTTTGTTT
TTCGTTTTTTTTTTTGTTTTTTCGTTTTTTTGTTTTTTTTTTTTTTGTTTCGTTTTTTTG
TTTCGTTTTTTTGTTTTTTTTTTTTGTTTTTTTTTTGTTTTTTTTTTTTTTGTTTTTTGT
TTTTTTGTTTTTTGTTTTTTTGTTTTTTATACGTTTGTTTTTGAAGATGTAAGAAATAAA
GTTTTGTCGTAGAAGATTTTGGTTTGTGGTGTTTTTTTTGGTCGGTCGTGAGAACGCGTT
TAATAATAATTGGTGTCGAATTTCGGGACGAGAAAAAAAAATTCGGGATTGGCGTAAGGA
GGATTTTTTATTTCGGAATTAGAATTGCGGATTACGTTTATAAAGGTTTTCGTAATATAG
ATTGTTGAGAAGGATTTAATTGTCGAATTTAGAATTTATTAGTTGGGGAACGATGGTGAT
AAAGGTTTTCGTAAAGTAGATTGTTAAGAAGGATTTAATTGTATGAATTTAGAATTTTTT
AGTTGGGG
>42LLTRBr_25
GTTTGATTAGATTTTTTTTTTTTATTATGTTTCGGGAGTTAGGTGAGTATTGAGGATAGA
TAGTGTTGGGAGTCGCGTTTATATTCGTTGTTATAAGAAGGCGTTGATAGTTGTGTTTTG
AGTGGTAAATAAATAATTTGCGTATGTGTTAAGGGTATTTTATGATTATTTGTGTTTTGT
TTTTTTCGTGATGTTAATTCCGTCGATGGGTTGTAGTTAATTAGGGAGTGATACCTTCGA
GGCGAAGGAGAATGTTTTTTAAGAGGGGCGGGGTTTCGTTTTTTTTTTTTTGTTTTTCGT
TTTTTCTTTTGTTTTTTCGTTTTTTTGTTTTTTTTTTTTTTTGTTTCGTTTTTTTGTTTC
GTTTTTTTGTTTTTTTTTTGTTTTTTTTTTTGTTTTTTTTTTTGTTTTTTGTTTTTTTGT
TTTTTGTTTTTTTGTTTTTTATACGTTTGTTTTTGAAGATGTAAGAAATAAAGTTTTGTC
GTAGAAGATTTTGGTTTGTGGTGTTTTTTTTGGTCGGTCGTGAGAACGCGTTTAATAATA
ATTGGTGTCGGATTTCGGGACGAGAAAAAAAAATTCGGGATTGGCGTAAGGAGGATTTTT
TATTTCGGAATTAGAATTGCGGATTACGTTTATAAAGGTTTTCGTAATATAGATTGTTGA
GAAGGATTTAATTGTCGAATTTAGAATTTATTAGTTGGGGAACGACGGTGATAAAGGTTT
TCGTAAAGTAGATTGTTAAGAAGGATTTAATTGTATGAATTTAGAATTTTTTAGTTGGGG
>42LLTRBr_26
GTTTGATTAGATTTTTTTTTTATTATGTTTTGGGAGTTAGGTGAGTATTGAGGATAGATA
GTGTTGGGAGTTGCGTTTATATTTGTCGTTATAAGAAGGCGTTGATAGTTGTGTTTTAAG
TGGTAAATAAATAATTTGCGTATGTGTTAAGGGTATTTTATGATTATTTGTGTTTTGTTT
TTTTCGGGACGTTAATTCGGTCGATGGGTTGTAGTTAATTAGGGAATGATACCTTCGAGG
CGAAGGAGAATGTTTTTTTAGAGGGACGGGGGTTCGTTTTTTTTTTTTTTTGTTTTTCGT
TTTTTTTTTTTGTTTTTTCGTTTTTTTGTTTTTTTTTTTTTTTTGTTTCGTTTTTTTGTT
TCGTTTTTTTGTTTTCTTTTTTTGTTTTTTTTTTTGTTTTTTTTTTTTTTGTTTTTTGTT
TTTTTGTTTTTTGTTTTTTTGTTTTTTATACGTTTGTTTTTGAAGATGTAAGAAATAAAG
TTTTGTCGTAGAAGATTTTGGTTTGTGGTGTTTTTTTTGGTCGGTCGTGAGAACGCGTTT
AATAATAATTGGTGTCGAATTTCGGGACGAGAAAAAAAAATTCGGGATTGGCGTAAGGAG
GATTTTTTATTTCGGGATTAGAATTGCGGATTATGTTTATAAAGGTTTTCGTAATATAGA
TTGTTGAGAAGGATTTAATTGTCGAATTTAGAATTTATTAGTTGGGGAACGACGGTGATA
AAGGTTTTCGTAAAGTAGATTGTTAAGAAGGATTTAATTGTATGAATTTAGAATTTTTTA
GTTG
>42LLTRBr_27
GTTTGATTAGATTTTTTTTTTATTATGTTTCGGGAGTTAGGTGAGTATTGAGGATAGATA
GTGTTGGGAGTCGCGTTTATTTTCGTTGTTATAAGATGGCGTTGATAGTTGTGTTTTAAG
TGGTAAATAAATAATTTGCGTATGTGTTAAGGGTATTTTATGATTATTTGTGTTTTGTTT
TTTTCGTGATGTTAATTCGGTCGATGGGTTGTAGTTAATTAGGGAATGATACCTTCGAGG
CGAAGGAGAATGTTTTTTAAGAGGGACGGGGTTTCGTTTTTTTTTTTTTTGTTTTTCGTT
TTTTCTTTTGTTTTTTCGTTTTTTTGTTTTTTTTTTTGTTTCGTTTTTTTGTTTCGTTTT
TTTGTTTTTTTTTTTGTTTTTTTTTTTGTTTTTTTTTTTTTGTTTTTTGTTTTTTTGTTT
TTTGTTTTTTTGTTTTTTATACGTTTGTTTTTGAAGATGTAAGAAATAAAGTTTTGTCGT
AGAAGATTTTGGTTTGTGGTGTTTTTTTTGGTCGGTCGTGAGAACGCGTTTAATAATAAT
TGGTGTCGAATTTCGGGACGAGAAAAAAAAATTCGGGATTGGCGTAAGGAGGATTTTTTA
TTTCGGAATTAGAATTGCGGATTACGTTTATAAAGGTTTTCGTAATATAGATTGTTGAGA
AGGATTTAATTGTCGAATTTAGAATTTATTAGTTGGGGAACGACGGTGATAAAGGTTTTC
GTAAAGTAGATTGTTAAGAAGGATTTAATTGTATGAATTTAGAATTTTTTAGTTGGGG
>42LLTRBr_29
GTTTGATTTAGATTTTTTTTTTTATTAATGTTTCGGGGAGTTTAGGTGAGTATTGAGGAT
AGATAGTGTTGGGGAGTCGCGTTTATATTTGTTGTTATAAGATGGCGCTGATAGTTGTGT
TTTAAGTGGTAAATAAATAATTTGCGTATGTGTTAAGGGTATTTTATGATTATTTGTGTT
TTGTTTTTTTCGTGACGTTAATTCGGTCGATGGGTTGTAGTTAATTAGGGAATGATACCT
TCGAGGCGAAGGAGAATGTTTTTTAAGAGGGACGGGGTTTCGTTCTTTTTTTTTTGTTTT
TCGTTTTTTTTTTTGTTTTTTCGTTTTTTTGTTTTTTTTTTTTTTGTTTCGTTTTTTTGT
TTCGTTTTTTTGTTTTTCTTTTTGTTTCTTTTTTTTGTTTTTTTTTTTTTGTTTTTTGTT
TTTTTGTTTTTTGTTTTTTTGTTTTTTATACGTTTGTTTTTGAAGATGTAAGAAATAAAG
TTTTGTCGTAGAAGATTTTGGTTTGTGGTGTTTTTTTTGGTCGGTCGTGAGAACGCGTTT
AATAATAATTGGTGTCGAATTTCGGGACGAGAAAAAAAGTTTGGGATTGGCGTAAGGAGG
ATTTTTTATTTCGGAATTAGAATTGCGGATTATGTTTATAAAGGTTTTCGTAATGTAGAC
TGTTGAGAAGGATTTAATTGTCGAATTTAGAATTTATTAGTTGGGGAACGACGGTGATAA
AGGTTTTCGTAAAGTAGATTGTTAAGAAGGATTTAATTGTATGAATTTAGAATTTTTTAG
TTGGGG
>42LLTRBr_33
GTTTGATTAGATTTTTTTTTTAATATGTTTCGGGAGTTAGGTGAGTATTGAAGATAGATA
GTGTTGGGAGTCGCGTTTATATTTGTCGTTATAAGAAGGCGTTGATAGTTGTGTTTTAAG
TGGTAAATAAATAATTTGCGTATGTGTCAAGGGTATTTTATGATTATTTGTGTTTTGTTT
TTTTTGTGATGTTAATTCGGTCGATGGGTTGTAGTTAATTAGGGAGTGATACGTTCGAGG
CGAAGGAGAATGTTTTTTAAGAGGGACGGGGTTTCGTTTTTTTTTTTTGTTTTTCGTTTT
TTTTTTGTTTTTTCGTTTTTTTGTTTTTTTTTTTTTTTTGTTTCGTTTTTTTGTTTCGTC
TTTTTGTTTTTTTCTTTTGTTTTTTTTTTTGTTTTTTTTTTTTGTTTTTTGTTTTTTTGT
TTTTTGTTTTTTTGTTTTTTATACGTTTGTTTTTGAAGATGTAAGAAATAAAGTTTTGTC
GTAGAAGATTTTGGTTTGTGGTGTTTTTTTTGGTCGGTCGTGAGAACGCGTTTAATAATA
ATTGGTGTTGAATTTCGGGATGAGAAAAAAAAATTCGGGATTGGCGTAAGGAGGATTTTT
TATTTCGGAATTAGAATTGCGGATTACGTTTATAAAGGTTTTCGTAATATAGATTGTTGA
GAAGGATTTAATTGTCGAATTTAGAATTTATTAGTTGGGGAACGACGGTGATAAAGGTTT
TCGTAAAGTAGATTGTTAAGAAGGATTTAATTGTATGAATTTAGAATTTTTTAGTTGGGG
>42LLTRBr_35
GTTTGATTAGATTTTTTTTTTTATTATGTTTCGGGAGTTAGGTGAGTATTGAGGATAGAT
AGTGTTGGGAGTCGCGTTTATTTTCGTCGTTATAAGATGGCGTTGATAGTTGTGTTTTAA
GTGGTAAATAAATAATTTGCGTATGTGTCAAGGGTATTTTATGATTATTTGTGTTTTGTT
TTTTTTGTGATGTTAATTCGGTCGATGGGTTGTAGTTAATTAGGGAATGATACCTTCGAG
GCGAAGGAGAATGTTTTTTAAGAGGGACGGGGTTTCGTTTTTTTTTTTTTTTGTTTTTCG
TTTTTTTTTGTTTTTTCGTTTTTTTGTTTTTTTTTTTTTTTGTTTCGTTTTTTTGTTTCG
TTTTTTTGTTTTTTTCTTTTGTTTTTTTTTTTTGTTTTTTTTTTTTTGTTTTTTGTTTTT
TTGTTTTTTGTTTTTTTGTTTTTTATACGTTTGTTTTTGAAGATGTAAGAAATAAAGTTT
TGTCGTAGAAGATTTTGGTTTGTGGTGTTTTTTTTGGTCGGTCGTGAGAACGCGTTTAAT
AATAATTGGTGTTGAATTTCGGGATGAGAAAAAAAAATTCGGGATTGGCGTAAGGAGGAT
TTTTTATTTCGGAATTAGAATTGCGGATTACGTTTATAAAGGTTTTCGTAATATAGATTG
TTGAGAAGGATTTAATTGTCGAATTTAGAATTTATTAGTTGGGGAACGACGGTGATAAAG
GTTTTCGTAAAGTAGATTGTTAAGAAGGATTTAATTGTATGAATTTAGAATTTTTTAGTT
GGG
>42LLTRBr_36
GTTTGAATAGATTTTTTTTTTATTATGTTTCGGGAGTTAGGTGAGTATTGAGGATAGATA
GTGTTGGGAGTCGCGTTTATATTCGTTGTTATAAGATGGCGTTGATAGTTGTGTTTTAAG
TGGTAAATAAATAATTTGCGTATGTGTTAAGGGTATTTTATGATTATTTGTGTTTTGTTT
TTTTCGTGATGTTAATTCGGTCGATGGGTTGTAGTTAATTAGGGAATGATACCTTCGAGG
CGAAGGAGAATGTTTTTTAAGAGGGACGGGGTTTCGTTTTTTTTTTTTTTGTTTTTCGTT
TTTTCTTTTGTTTTTTCGTTTTTTTGTTTTTTTTTTTGTTTCGTTTTTTTGTTTCGTTTT
TTTGTTTTTTTTTTTGTTTTTTTTTTTGTTTTTTTTTTTTTGTTTTTTGTTTTTTTGTTT
TTTGTTTTTTTGTTTTTTATACGTTTGTTTTTGAAGATGTAAGAAATAAAGTTTTGTCGT
AGAAGATTTTGGTTTGTGGTGTTTTTTTTGGTCGGTCGTGAGAACGCGTTTAATAATAAT
TGGTGTCGAATTTCGGGACGAGAAAAAAAAATTCGGGATTGGCGTAAGGAGGATTTTTTA
TTTCGGAATTAGAATTGCGGATTACGTTTATAAAGGTTTTCGTAATATAGATTGTTGAGA
AGGATTTAATTGTCGAATTTAGAATTTATTAGTTGGGGAACGACGGTGATAAAGGTTTTC
GTAAAGTAGATTGTTAAGAAGGATTTAATTGTATGAATTTAGAATTTTTTAGTTGGGG
