# Supplementary material for: Epigenetic interplay between mouse endogenous retroviruses and host genes
Source: Genome Biol. 2012 Oct 3;13(10):R89. doi: 10.1186/gb-2012-13-10-r89 (PMC3491417; doi:10.1186/gb-2012-13-10-r89)
Supplement: Additional file 4 — All bisulfite sequencing data. Compilation of all bisulfite sequences. [file gb-2012-13-10-r89-S4.zip › IAP8253_TE_kidney.rtf]

2/16/10 Miniprep Sequences
Catsper 3LTR-B6AJ Kidney
>CatRLTRKidney51_M13R
GGGAGATATGTTATTTTTTATGAAGGTTTAGTGTTTTAGTTTTTTTTTTTTAGGTAAAAC
GATACGGGAGTAGGTTAGGGTTGTTCTGGGTAAAAGTTTGTGAGTTTAAGAGTTAATTTT
GTATATGGTTTTTTTATTTATATATTGGGGATTTGATTTTTATTTTTATTTTTATTAATA
TGGGTGGTTTATTTGTTTTTATTAAAAGGAAAGGGGGAGATGTTGGGAGTCGCGTTTATA
TTCGTCGTTATAAGATGGCGTTGATAGTTGTGTTTTAAGTGGTAAATAAATAATTTGCGT
ATGTGTCGAGGGTGGTTTTTTATTTTATGTGTTTTGTTTTTCTCGTGACGTTAATTCGGT
CGATGGGTTGTAGTTAATTAGGGAGTGATACGTTTTAGGCGAAGGAGAGTTTTTTTTAAT
AGGGACGGGGTTTCGTTTTCGTTTTTTTTTGTTTTTTGTATTTTGGTTTTTGAAGATGTA
AGTAATAAAGTTTTGTCGTAGAAGATTTTGGTTTGTTGCGTTTTTTTTTTGGTCGGTCGT
GAGAACGCGTTTAATAATACGTGTTGTTTTAAGCGTTTTTGGGAGTTTAGTAGTGGTGGT
TTTGTAAGATTGGGTTTATTGATGTTGTTTTTTTTTTTTTGTTGTTTTTTATTTAAGGAA
GGATGGTGAGAGGTAGGGTATAGAATTCTTATTTGAGTATTTAGTTATTTTATGTTATAC
GTGTGCGGTTTTGTTTTGATTGTTGTGTAGAGTAAGGTTAGAGTG
>CatRLTRKidney52_M13R
GGGAGATATGTTATTTTTTATGAAGGTTTAGTGTTTTAGTTTTTTTTTTTAGGTAAAACG
ATACGGGAGTAGGTTAGGGTTGTTTTGGGTAAAAGTTTGTGAGTTTAAGAGCTAATTTTG
TATATGGTTTTTTTATTTATATATTGGGGATTTGATTTTTATTTTTATTTTTATTAGTAT
GGGTGGTTTATTTGTTTTTATTAAAAGGAAAGGGGGAGATGTTGGGAGTCGCGTTTATAT
TCGTCGTTATAAGATGGCGTTGATAGTTGTGTTTTAAGTGGTAAATAAATAATTTGCGTA
TGTGTCGAGGGTGGTTTTTTATTTTATGTGTTTTGTTTTTTTTGTGACGTTAATTCGGTC
GATGGGTTGTAGTTAATTAGGGAGTGATACGTTTTAGGCGAAGGAGAATTTTTTTTAATA
GGGACGGGGTTTCGTTTTCGTTTTTTTTTGTTTTTTGTATTTTGGTTTTTGAAGATGTAA
GTAATAAAGTTTTGTCGTAGAAGATTTTGGTTTGTTGCGTTTTTTTTGGTCGGTCGTGAG
AACGCGTTTAATAATACGTGTTGTTTTAAGCGTTTTTGGGAGTTTAGTAGTGGTGGTTTT
GTAAGATTGGGTTTATTGATGTTGTTTTTTTTTTTTTTGTTGTTTTTTATTTAAGGAAGG
ATGGTGAGAGGTAGGGTATAGAATTTTTATTTGAGTATTTAGTTATTTTATGTTATACGT
GTGCGGTTTTGTTTTGATTGTTGTGTAGAGTAAGGTTAGAGTG
>CatRLTRKidney53_M13R
GGGAGATATGTTATTTTTTATGAAGGTTTAGTGTTTTAGTTTTTTTTTTCAGGTAAAACG
ATACGGGAGTAGGTTAGGGTTGTTTTGGGTAAAAGTTTGTGAGTTTAAGAGTTAATTTTG
TATATGGCTTTTTTACTTATATATTGGGGATTTGATTTTTATTTTTATTTTTATTAATAT
GGGTGGTTTATTTGTTTTTATTAAAAGGAAAGGGGGAGATGTTGGGAGTCGCGTTTATAT
TCGTCGTTATAAGATGGCGTTGACAGTTGTGTTTTAAGTGGTAAATAAATAATTTGCGTA
TGTGTCGAGGGTGGTTTTTTATTTTATGTGTTTTGTTTTTTTTGTGACGTTAATTCGGTC
GATGGGTTGTAGTTAATTAGGGAGTGATACGTTTTAGGCGAAGGAGAATTTTTTTTAATA
GGGACGGGGTTTCGTTTTCGTTTTCTTTTGTTTTTTGTATTTTGGTTTTTGAAGATGTAA
GTAATAAAGTTTTGTCGTAGAAGATTTTGGTTTGTTGCGTTTTTTTTGGTCGGTCGTGAG
AACGCGTTTAATAACACGTGTTGTTTTAAGCGTTTTTGGGAGTTTAGTAGTGGTGGTTTT
GTAAGATTGGGTTTATTGATGTTGTTTTTTTTTTTTTTTTTGTTGTTTTTTATTTAAGGA
AGGATGGTGAGAGGTAGGGTATAGAATTTTTATTTGAGTATTTAGTCATTTTATGTTATA
CGTGTGCGGTTTTGTTTTGATTGTTGTGTAGAGTAAGGTTAGAGTG
>CatRLTRKidney54_M13R
GGGAGATATGTTATTTTTTATGAAGGTTTAGTGTTTTAGTTTTTTTTTTTAGGTAAAACG
ATACGGGAGTAGGTTAGGGTTGTTTTGGGTAAAAGTTTGTGAGTTTAAGAGTTAATTTTG
TATATGGTTTTTTTATTTATATATTGGGGATTTGATTTTTATTTTTATTTTTATTAATAT
GGGTGGTTTATTTGTTTTTATTAAAAGGAAAGGGGGAGATGTTGGGAGTCGCGTTTATAT
TCGTCGTTATAAGATGGCGTTGATAGTTGTGTTTTAAGTGGTAAATAAATAATTTGCGTA
TGTGTCGAGGGTGGTTTTTTATTTTATGTGTTTTGTTTTTTTCGTGACGTTAATTCGGTC
GATGGGTTGTAGTTAATTAGGGAGTGATACGTTTTAGGCGAAGGAGAATTTTTTTTAATA
GGGACGGGGTTTCGTTTTCGTTTTTTTTTGTTTTTTGTATTTTGGTTTTTGAAGATGTAA
GTAATAAAGTTTTGTCGTAGAAGATTTTGGTTTGTTGCGTTTTTTTGGTCGGTCGTGAGA
ACGCGTTTAATAATACGTGTTGTTTTAAGCGTTTTTGGGAGTTTAGTAGTGGTGGTTTTG
TAAGATTGGGTTTATTGATGTTGTTTTTTTTTTTTTTTGTTGTTTTTTATTTAAGGAAGG
ATGGTGAGAGGTAGGGTATAGAATTCTTATTTGAGTATTTAGTTATTTTATGTTATACGT
GTGCGGTTTTGTTTTGATTGTTGTGTAGAGTAAGGTTAGAGTG
>CatRLTRKidney55_M13R
GGGAGATATGTTATTTTTTATGAAGGTTTAGTGTTTTAGTTTTTTTTTTTTAGGTAAAAC
GATACGGGAGTAGGTTAGGGTTGTTTTGGGTAAAAGTTTGTGAGTTTAAGAGTTAATTTT
GTATATGGTTTTTTTATTTATATATTGGGGATTTGATTTTTATTTTTATTTTTATTAATA
TGGGTGGTTTATTTGTTTTTATTAAAAGGAAAGGGGGAGATGTTGGGAGTCGCGTTTATA
TTCGTCGTTATAAGATGGCGTTGATAGTTGTGTTTTAAGTGGTAAATAAATAATTTGTGT
ATGTGTCGAGGGTGGTTTTTTATTTTATGTGTTTTGTTTTTTTCGTGACGTTAATTCGGT
CGATGGGTTGTAGTTAATTAGGGAGTGATACGTTTTAGGCGAAGGAGAATTTTTTTTAAT
AGGGACGGGGTTTCGTTTTCGTTTTTTTTTGTTTTTTGTATTTTGGTTTTTGAAGATGTA
AGTAATAAAGTTTTGTCGTAGAAGATTTTGGTTTGTTGCGTTTTTTTTGGTCGGTCGTGA
GAACGCGTTTAATAATACGTGTTGTTTTAAGCGTTTTTGGGAGTTTAGTAGTGGTGGTTT
TGTAAGATTGGGTTTATTGATGTTGTTTTTTTTTTTTTTGTTGTTTTTTATTTAAGGAAG
GATGGTGAGAGGTAGGGTATAGAATTTTTATTTGAGTATTTAGTTATTTTATGTTATACG
TGTGCGGTTTTGTTTTGATTGTTGTGTAGAGTAAGGTTAGAGTG
>CatRLTRKidney56_M13R
GGGAGATATGTTATTTTTTATGAAGGTTTAGTGTTTTAGTTTTTTTTTTTTAGGTAAAAC
GATACGGGAGTAGGTTAGGGTTGTTTTGGGTAAAAGTTTGTGAGTTTAAGAGTTAATTTT
GTATATGGCTTTTTTATTTATATATTGGGGATTTGACTTTTATTTTTATTTTTATTAATA
TGGGTGGTTTATTTGTTTTTATTAAAAGGAAAGGGGGAGATGTTGGGAGTCGCGTTTATA
TTCGTCGTTATAAGATGGCGTTGATAGTTGTGTTTTAAGTGGTAAATAAATAATTTGCGT
ATGTGTCGAGGGTGGTTTTTTATTTTATGTGTTTTGTTTTTTTCGTGACGTTAATTCGGT
CGATGGGTTGTAGTTAATTAGGGAGTGATACGTTTTAGGCGAAGGAGAATTTTTTTTAAT
AGGGACGGGGTTTCGTTTTCGTTTTTTTTTGTTTTTTGTATTTTGGTTTTTGAAGATGTA
AGTAATAAAGTTTTGTCGTAGAAGATTTTGGTTTGTTGCGTTTTTTTTGGTCGGTCGTGA
GAACGCGTTTAATAATACGTGTTGTTTTAAGCGTTTTTGGGAGTTTAGTAGTGGTGGTTT
TGTAAGATTGGGTTTATTGATGTTGTTTTTTTTTTTTGTTGTTTTTTATTTAAGGAAGGA
TGGTGAGAGGTAGGGTATAGAATTTTTATTTGAGTATTTAGTTATTTTATGTTATACGTG
TGCGGTTTTGTTTTGATTGTTGTGTAGAGTAAGGTTAGAGTG
>CatRLTRKidney57_M13R
GGGAGATATGTTATTTTTTATGAAGGTTTAGTGTTTTAGTTTTTTTTTTTTAGGTAAAAC
GATACGGGAGTAGGTTAGGGTTGTTTTGGGTAAAAGTTTGTGAGTTTAAGAGTTAATTTT
GTATATGGTTTTTTTATTTATATATTGGGGATTTGATTTTTATTTTTATTTTTATTAATA
TGGGTGGTTTATTTGTTTTTATTAAAAGGAAAGGGGGAGATGTTGGGAGTCGCGTTTATA
TTCGTCGTTATAAGATGGCGTTGATAGTTGTGTTTTAAGTGGTAAATAAATAATTTGCGT
ATGTGTCGAGGGTGGTTTTTTATTTTATGTGTTTTGTTTTTTTCGTGACGTTAATTCGGT
CGATGGGTTGTAGTTAATTAGGGAGTGATGCGTTTTAGGCGAAGGAGAATTTTTCTTAAT
AGGGACGGGGTTTCGTTTTCGTTTTTTTTTGTTTTTTGTATTTTGGTTTTTGAAGATGTA
AGTAATAAAGTTTTGTCGTAGAAGATTTTGGTTTGTTGTGTTTTTTTTGGTCGGTCGTGA
GAACGCGTTTAATAATACGTGTTGTTTTAAGCGTTTTTGGGAGTTTAGTAGTGGTGGTTT
TGTAAGATTGGGTTTATTGATGTTGTTTTTTTTTTTTTGTTGTTTTTTATTTAAGGAAGG
ATGGTGAGAGGTAGGGTATAGAATTTTTATTTGAGTATTTAGTTATTTTATGTTATACGT
GTGCGGTTTTGTTTTGATTGTTGTGTAGAGTAAGGTTAGAGTG
>CatRLTRKidney58_M13R
GGGAGATATGTTATTTTTTATGAAGGTTTAGTGTTTTAGTTTTTTTTTTTTAGGTAAAAC
GATACGGGAGTAGGTTAGGGTTGTTTTGGGTAAAAGTTTGTGAGTTTAAGAGTTAATTTT
GTATATGGTTTTTTTATTTATATATTGGGGATTTGATTTTTATTTTTATTTTTATTAATA
TGGGTGGTTTATTTGTTTTTATTAAAAGGAAAGGGGGAGATGTCGGGAGTCGCGTTTATA
TTCGTCGTTATAAGATGGCGTTGATAGTTGTGTTTTAAGTGGTAAATAAATAATTTGCGT
ATGTGTCGAGGGTGGTTTTTCATTTTATGTGTTTTGTTTTTTTCGTGACGTTAATTCGGT
CGATGGGTTGTAGTTAATTAGGGAGTGATACGTTTTAGGCGAAGGAGAATTTTTTTTAAT
AGGGACGGGGTTTCGTTTTCGTTTTTTTTTGTTTTTTGTATTTTGGTTTTTGAAGATGTA
AGTAATAAAGTTTTGTCGTAGAAGATTTTGGTTTGTTGCGTTTTTTTTGGTCGGTCGTGA
GAACGCGTTTAATAATACGTGTTGTTTTAAGCATTTTTGGGAGTTTAGTAGTGGTGGTTT
TGTAAGATTGGGTTTATTGATGTTGTTTTTTTCTTTTTTTGTTGTTTTTTATTTAAGGAA
GGATGGTGAGAGGTAGGGTATAGAATTTTTATTTGAGTATTTAGTTATTTTATGTTATAC
GTGTGCGGTTTTGTTTTGATTGTTGTGTAGAGTAAGGTTAGAGTG
>CatRLTRKidney60_M13R
GGGAGATATGTTATTTTTTATGAAGGTTTAGTGTTTTAGTTTTTTTTTTTTAGGTAAAAC
GATACGGGAGTAGGTTAGGGTTGTCTTGGGTAGAAGTTTGTGAGTTTAAGAGTTAATTTT
GTATATGGTTTTTTTATTTATATATTGGGGATTTGATTTTTATTTTTATTTTTATTAATA
TGGGTGGTTTATTTGTTTTTATTAAAAGGAAAGGGGGAGATGTTGGGAGTCGCGTTTATA
TTCGTCGTTATAAGATGGCGTTGATAGTTGTGTTTTAAGTGGTAAATAAATAATTTGCGT
ATGTGTCGAGGGTGGTTTTTTATTTTATGTGTTTTGTTTTTTTCGTGACGTTAATTCGGT
CGATGGGTTGTAGTTAATTAGGGAGTGATACGTTTTAGGCGAAGGAGAATTTTTTTTAAT
AGGGACGGGGTTTCGTTTTCGTTTTTTTTTGTTTTTTGTGTTTTGGTTTTTGAAGATGTA
AGTAATAAAGTTTTGTCGTAGAAGATTTTGGTTTGTTGCGTTTTTTTTGGTCGGTCGTGA
GAACGCGTTTAATAATACGTGTTGTTTTAAGCGTTTTTGGGAGTTTAGCAGTGGTGGTTT
TGTAAGATTGGGTTTATTGATGTTGTTTTTTTTTTTTTGTTGTTTTTTATTTAAGGAAGG
ATGGTGAGAGGTAGGGTATAGAATTTTTATTTGAGTATTTAGTTATTTTATGTTATACGT
GTGTGGTTTTGTTTTGATTGTTGTGTAGAGTAAGGTTAGAGTG
>CatRLTRKidney61_M13R
GGGAGATATGTTATTTTTTATGAAGGTTTAGTGTTTTAGTTTTTTTTTTTAGGTAAAACG
ATATGGGAGTAGGTTAGGGTTGTTTTGGGTAAAAGTTTGTGAGTTTAAGAGTTAATTTTG
TATATGGTTTTTTTATTTATATATTGGGGATTTGATTTTTATTTTTATTTTTATTAATAT
GGGTGGTTTATTTGTTTTTATTAAAAGGAAAGGGGGAGATGTTGGGAGTCGCGTTTATAT
TCGTCGTTATAAGATGGCGTTGATAGTTGTGTTTTAAGTGGTAAATAAATAATTTGCGTA
TGTGTCGAGGGTGGTTTTTTATTTTATGTGTTTTGTTTTTTTCGTGACGTTAATTCGGTC
GATGGGTTGTAGTTAATTAGGGAGTGATACGTTTTAGGCGAAGGAGAATTTTTTTTAATA
GGGACGGGGTTTCGTTTTCGTTTTTTTTTGTTTTTTGTATTTTGGTTTTTGAAGATGTAA
GTAATAAAGTTTTGTCGTAGAAGATTTTGGTTTGTTGCGTTTTTTTTGGTCGGTCGTGAG
AACGCGTTTAATAATACGTGTTGTTTTAAGCGTTTTTGGGAGTTTAGTAGTGGTGGTTTT
GTAAGATTGGGTTTATTGATGTTGTTTTTTTTTTTTTGTTGTTTTTTATTTAAGGAAGGA
TGGTGAGAGGTAGGGTATAGAATTTTTATTTGAGTATTTAGTTATTTTATGTTATACGTG
TGCGGTTTTGTTTTGATTGTTGTGTAGAGTAAGGTTAGAGTG
>CatRLTRKidney62_M13R
GGGAGATATGTTATTTTTTATGAAGGTTTAGTGTTTTAGTTTTTTTTTTAGGTAAAACGG
TACGGGAGTAGGTTAGGGTTGTTTTGGGTAAAAGTTTGTGAGTTTAAGAGTTAATTTTGT
ATATGGTTTTTTTGTTTATATATTGGGGATTTGATTTTTATTTTTATTTTTATTAATATG
GGTGGTTTATTTGTTTTTATTAAAAGGAAAGGGGGAGATGTTGGGAGTCGCGTTTATATT
CGTCGTTATAAGATGGCGTTGATAGTTGTGTTTTAAGTGGTAAATAAATAATTTGCGTAT
GTGTCGAGGGTGGTTTTTTATTTTATGTGTTTTGTTTTTTTCGTGACGTTAATTCGGTCG
ATGGGTTGTAGTTAATTAGGGAGTGATACGTTTTAGGCGAAGGAGAATTTTTTTTAATAG
GGACGGGGTTTCGTTTTCGTTTTTTTTTGTTTTTTGTATTTTGGTTTTTGAAGATGTAAG
TAATAAAGTTTTGTCGTAGAAGATTTTGGTTTGTTGCGCTTTTTTTGGTCGGTCGTGAGA
ACGCGTTTAATAATACGTGTTGTTTTAAGCGTTTTTGGGAGTTTAGTAGTGGTGGTTTTG
TAAGATTGGGTTTATTGATGTTGTTTTTTTTTTTTTTTTGTTGTTTTTTATTTAAGGAAG
GATGGTGAGAGGTAGGGTATAGAATTTTTATTTGAGTATTTAGTTATTTTATGTTATACG
TGTGCGGTTTTGTTTTGATTGTTGTGTAGAGTAAGGTTAGAGTG
>CatRLTRKidney63_M13R
GGGAGATATGTTATTTTTTATGAAGGTTTAGTGTTTTAGTTTTTTTTTTTAGGTAAAACG
ATACGGGAGTAGGTTAGGGTTGTTTTGGGTAAAAGTTTGTGAGTTTAAGAGTTAATTTTG
TATATGGTTTTTTTATTTATATATTGGGGATTTGATTTTTATTTTTATTTTTATTAATAT
GGGTGGTTTATTTGTTTTTATTAAAAGGAAAGGGGGAGATGTTGGGAGTCGCGTTTATAT
TCGTCGTTATAAGATGGCGTTGATAGTTGTGTTTTAAGTGGTAAATAAATAATTTGCGTA
TGTGTCGAGGGTGGTTTTTTATTTTATGTGTTTTGTTTTTTTTGTGACGTTAATTCGGTC
GATGGGTTGTAGTTAATTAGGGAGTGATACGTTTTAGGCGAAGGAGAATTTTTTTTAATA
GGGACGGGGTTTCGTTTTCGTTTTTTTTTGTTTTTTGTATTTTGGTTTTTGAAGATGTAA
GTAATAAAGTTTTGTCGTAGAAGATTTTGGTTTGTTGCGTTTTTTTTGGTCGGTCGTGAG
AACGCGTTTAATAATACGTGTTGTTTTAAGCGTTTTTGGGAGTTTAGTAGTGGTGGTTTT
GTAAGATTGGGTTTATTGATGTTGTTTTTTTTTTTTTTTGTTGTTTTTTATTTAAGGAAG
GATGGTGAGAGGTAGGGTATAGAATTTTTATTTGAGTATTTAGTTATTTTATGTTATACG
TGTGCGGTTTTGTTTTGATTGTTGTGTAGAGTAAGGTTAGAGTG
>CatRLTRKidney64_M13R
GGGAGATATGTTATTTTTTATGAAGGTTTAGTGTTTTAGTTTTTTTTTTTTTTAGGTAAA
ACGATACGGGAGTAGGTTAGGGTTGTTTTGGGTAAAAGTTTGTGAGTTTAAGAGTTAATT
TTGTATATGGTTTTTTTATTTATATATTGGGGATTTGATTTTTATTTTTATTTTTATTAA
TATGGGTGGTTTATTTGTTTTTATTAAAAGGAAAGGGGGAGATGTTGGGAGTCGCGTTTA
TATTCGTCGTTATAAGATGGCGTTGATAGTTGTGTTTTAAGTGGTAAATGAATAATTTGC
GTATGTGTTGAGGGTGGTTTTTTATTTTATGTGTTTTGTTTTTTTCGTGACGTTAATTCG
GTCGATGGGTTGTAGTTAATTAGGGAATGATACGTTTTAGGCGAAGGAGAATTTTTTTTA
ATAGGGACGGGGTTTCGTTTTCGTTTTTTTTGTTTTTTGTATTTTGGTTTTTGAAGATGT
AAGTAATAAAGCTTTGTCGTAGAAGATTTTGGTTTGTTGCGTTTTTTTTGGTCGGTCGTG
AGAACGCGTTTAATAATACGTGTTGTTTTAAGCGTTTTTGGGAGTTTTGTAGTGGTGGTT
TTGTAAGATTGGGTTTATTGATGTTGTTTTTTTTTTTTTGTTGTTTTTTATTTAAGGAAG
GATGGTGAGAGGTAGGGTATAGAATTTTTATTTGAGTATTTAGTTATTTTATGTTATACG
TGTGCGGTTTTGTTTTGATTGTTGTGTAGAGTAAGGTTAGAGTG
>CatRLTRKidney65_M13R
GGGAGATATGTTATTTTTTATGAAGGTTTAGTGTTTTAGTTTTTTTTTTTTAGGTAAAAC
GATACGGGAGTAGGTTAGGGTTGTTTTGGGTAAAAGTTTGTGAGTTTAAGAGTTAATTTT
GTATATGGTTTTTTTATTTATATATTGGGGATTTGATTTTTATTTTTATTTTTATTAATA
TGGGTGGTTTATTTGTTTTTATTAAAAGGAAAGGGGGAGATGTTGGGAGTCGCGTTTATA
TTCGTCGTTATAAGATGGCGTTGATAGTTGTGTTTTAAGTGGTAAATAAATAATTTGCGT
ATGTGTCGAGGGTGGTTTTTTATTTTATGTGTTTTGTTTTTTTCGTGACGTTAATTCGGT
CGATGGGTTGTAGTTAATTAGGGAGTGATACGTTTTAGGCGAAGGAGAATTTTTTTTAAT
AGGGACGGGGTTTCGTTTTCGTTTTTTTTGTTTTTTGTATTTTGGTTTTTGAAGATGTAA
GTAATAAAGTTTTGTCGTAGAAGATTTTGGTTTGTTGCGTTTTTTTTGGTCGGTCGTGAG
AACGCGTTTAATAATACGTGTTGTTTTAAGCGTTTTTGGGAGTTTAGTAGTGGTGGTTTT
GTAAGATTGGGTTTATTGATGTTGTTTTTTTTCTTTTTGTTGTTTTTTATTTAAGGAAGG
ATGGTGAGAGGTAGGGTATAGAATTTTTATTTGAGTATTTAGTTATTTTATGTTATACGT
GTGCGGTTTTGTTTTGATTGTTGTGTAGAGTAAGGTTAGAGTG
>CatRLTRKidney66_M13R
GGGAGATATGTTATTTTTTATGAAGGTTTAGTGTTTTAGTTTTTTTTTTTTAGGTAAAAC
GATACGGGAGTAGGTTAGGGTTGTTTTGGGTAAAAGTTTGTGAGTTTAAGAGTTAATTTT
GTATATGGTTTTTTTATTTATATATTGGGGATTTGATTTTTATTTTTATTTTTATTAATA
TGGGTGGTTTATTTGTTTTTATTAAAAGGAAAGGGGGAGATGTTGGGAGTCGCGTTTATA
TTCGTCGTTATAAGATGGCGTTGATAGTTGTGTTTTAAGTGGTAAATAAATAATTTGCGT
ATGTGTCGAGGGTGGTTTTTTATTTTATGTGTTTTGTTTTTTTCGTGACGTTAATTCGGT
CGATGGGTTGTAGTTAATTAGGGAGTGATACGTTTTAGGCGAAGGAGAATTTTTTTTAAT
AGGGACGGGGTTTCGTTTTCGTTTTTTTTGTTTTTTGTATTTTGGTTTTTGAAGATGTAA
GTAATAAAGTTTTGTCGTAGAAGATTTTGGTTTGTTGCGTTTTTTTTGGTCGGTCGTGAG
AACGCGTTTAATAATACGTGTTGTTTTAAGCGTTTTTGGGAGTTTAGTAGTGGTGGTTTT
GTAAGATTGGGTTTATTGATGTTGTTTTTTTTCTTTTTGTTGTTTTTTATTTAAGGAAGG
ATGGTGAGAGGTAGGGTATAGAATTTTTATTTGAGTATTTAGTTATTTTATGTTATACGT
GTGCGGTTTTGTTTTGATTGTTGTGTAGAGTAAGGTTAGAGTG
