# Supplementary material for: Epigenetic interplay between mouse endogenous retroviruses and host genes
Source: Genome Biol. 2012 Oct 3;13(10):R89. doi: 10.1186/gb-2012-13-10-r89 (PMC3491417; doi:10.1186/gb-2012-13-10-r89)
Supplement: Additional file 4 — All bisulfite sequencing data. Compilation of all bisulfite sequences. [file gb-2012-13-10-r89-S4.zip › IAP8253_TE_testis.rtf]

Catsper3  3LTR (Closest to Gene)-B6AJ Testis
Miniprep Sequences
>CatRLTRTestis1_M13R
GGGAGATATGTTATTTTTTATGAAGGTTTAGTGTTTTAGTTTTTTTTTTTTAGGTAAAAC
GATACGGGAGTAGGTTAGGGTTGTTTTGGGTAAAAGTTTGTGAGTTTAAGAGTTAATTTT
GTATATGGTTTTTTTATTTATATATTGGGGATTTGATTTTTATTTTTATTTTTATTAATA
TGGGTGGTTTATTTGTTTTTATTAAAAGGAAAGGGGGAGATGTTGGGAGTCGCGTTTATA
TTCGTCGTTATAAGATGGCGTTGATAGTTGTGTTTTAAGTGGTAAATAAATAATTTGCGT
ATGTGTCGAGGGTGGTTTTTTATTTTATGTGTTTTGTTTTTTTCGTGACGTTAATTCGGT
CGATGGGTTGTAGTTAATTAGGGAGTGATACGTTTTAGGCGAAGGAGAATTTTTTTTAAT
AGGGACGGGGTTTCGTTTTCGTTTTTTTTTGTTTTTTGTATTTTGGTTTTTGAAGATGTA
AGTAATAAAGTTTTGTCGTAGAAGATTTTGGTTTGTTGCGTTTTTTTTGGTCGGTCGTGA
GAACGCGTTTAATAATACGTGTTGTTTTAAGCGTTTTTGGGAGTTTAGTAGTGGTGGTTT
TGTAAGATTGGGTTTATTGATGTTGTTTTTTTTTTTTTTTGTTGTTTTTTATTTAAGGAA
GGATGGTGAGAGGTAGGGTATAGAATTTTTATTTGAGTATTTAGTTATTTTATGTTATAC
GTGTGCGGTTTTGTTTTGATTGTTGTGTAGAGTAAGGTTAGAGTGA
>CatRLTRTestis2_M13R
GGGAGATATGTTATTTTTTATGAAGGTTTAGTGTTTTAGTTTTTTTTTTTTTAGGTAAAA
CGATACGGGAGTAGGTTAGGGTTGTTTTGGGTAAAAGTTTGTGAGTTTAAGAGTTAATTT
TGTATATGGTTTTTTTATTTATATATTGGGGATTTGATTTTTATTTTTATTTTTATTAAT
ATGGGTGGTTTATTTGTTTTTATTAAAAGGAAAGGGGGAGATGTTGGGAGTCGCGTTTAT
ATTCGTCGTTATAAGATGGCGTTGATAGTTGTGTTTTAAGTGGTAAATAAATAATTTGCG
TATGTGTCGAGGGTGGTTTTTTATTTTATGTGTTTTGTTTTTTTCGTGACGTTAATTCGG
TCGATGGGTTGTAGTTAATTAGGGAGTGATACGTTTTAGGCGAAGGAGAATTTTTTTTAA
TAGGGACGGGGTTTTGTTTTCGTTTTTTTTTGTTTTTTGTATTTTGGTTTTTGAAGATGT
AAGTAATAAAGTTTTGTCGTAGAAGATTTTGGTTTGTTGCGTTTTTTTTGGTCGGTCGTG
AGAACGCGTTTAATAATACGTGTTGTTTTAAGCGTTTTTGGGAGTTTAGTAGTGGTGGTT
TTGTAAGATTGGGTTTATTGATGTTGTGTTTTTTTTTTTTGCTGTTTTTTATTTAAGGAA
GGATGGTGAGAGGTAGGGTATAGAATTTTTATTTGAGTATTTAGTTATTTTATGTTATAC
GTGTGCGGTTTTGTTTTGATTGTTGTGTAGAGTAAGGTTAGAGTG
>CatRLTRTestis3_M13R
GGGAGATATGTTATTTTTTATGAAGGTTTAGTGTTTTAGTTTTTTTTTTTTAGGTAAAAC
GATACGGGAGTAGGTTAGGGTTGTTTTGGGTAAAAGTTTGTGAGTTTAAGAGTTAATTTT
GTATATGGTTTTTTTATTTATATATTGGGGATTTGATTTTTATTTTTATTTTTATTAATA
TGGGTGGTTTATTTGTTTTTATTAAAAGGAAAGGGGGAGATGTTGGGAGTCGCGTTTATA
TTCGTCGTTATAAGATGGCGTTGATAGTTGTGTTTTAAGTGGTAAATAAATAATTTGCGT
ATGTGTCGAGGGTGGTTTTTTATTTTATGTGTTTTGTTTTTTTCGTGACGTTAATTCGGT
CGATGGGTTGTAGTTAATTAGGGAGTGATACGTTTTAGGCGAAGGAGAATTTTTTTTAAT
AGGGACGGGGTTTCGTTTTCGTTTTTTTTTGTTTTTTGTATTTTGGTTTTTGAAGATGTA
AGTAATAAAGTTTTGTCGTAGAAGATTTTGGTTTGTTGCGTTTTTTTTGGTCGGTCGTGA
GAACGCGTTTAATAATACGTGTTGTTTTAAGCGTTTTTGGGAGTTTAGTAGTGGTGGTTT
TGTAAGATTGGGTTTATTGATGTTGTTTTTTTTTTTTTTTGTTGTTTTTTATTTAAGGAA
GGATGGTGAGAGGTAGGGTATAGAATTTTTATTTGAGTATTTAGTTATTTTATGTTATAC
GTGTGCGGTTTTGTTTTGATTGTTGTGTAGAGTAAGGTTAGAGTG
>CatRLTRTestis4_M13R
GGGAGATATGTTATTTTTTATGAAGGTTTAGTGTTTTAGTTTTTTTTTTTAGGTAAAACG
ATACGGGAGTAGGTTAGGGTTGTTTTGGGTAAAAGTTTGTGAGTTTAAGAGTTAATTTTG
TATATGGTTTTTTTATTTATATATTGGGGATTTGATTTTTATCTTTATTTTTATTAATAT
GGGTGGTTTATTTGTTTTTATTAAAAGGAAAGGGGGAGATGTTGGGAGTCGCGTTTATTT
TCGTCGTTATAAGATGGCGTTGATAGTTGTGTTTTAAGTGGTAAATAAATAATTTGCGTA
TGTGTCGAGGGTGGTTTTTTATTTTATGTGTTTTGTTTTTTTCGTGACGTTAATTCGGTC
GATGGGTTGTAGTTAATTAGGGAGTGATACGTTTTAGGCGAAGGAGAATTTTTTTTAATA
GGGACGGGGTTTCGTTTTTGTTTTTTTTTGTTTTTTGTATTTTGGTTTTTGAAGATGTAA
GTAATAAAGTTTTGTCGTAGAAGATTTTGGTTTGTTGCGTTTTTTTTGGTCGGTCGTGAG
AACGCGTTTAATAATACGTGTTGTTTTAAGCGTTTTTGGGAGTTTAGTAGTGGTGGTTTT
GTAAGATTGGGTTTATTGATGTTGTTTTTTTTTTTTTTTTTGTTGTTTTTTATTTAAGGA
AGGATGGTGAGAGGTAGGGTATAGAATTTTTATTTGAGTATTTAGTTATTTTATGTTATA
CGTGTGCGGTTTTGTTTTGATTGTTGTGTAGAGTAAGGTTAGAGTG
>CatRLTRTestis5_M13R
GGGAGATATGTTATTTTTTATGAAGGTTTAGTGTTTTAGTTTCTTTTTTAGGTAAAACGA
TACGGGAGTAGGTTAGGGTTGTTTTGGGTAAAAGTTTGTGAGTTTAAGAGTTAATTTTGT
ATATGGTTTTTTTATTTATATATTGGGGATTTGATTTTTATTTTTATTTTCATTAATATG
GGTGGTTTATTTGTTTTTATTAAAAGGAAAGGGGGAGATGTTGGGAGTCGCGTTTATATT
CGTCGTTATAAGATGGCGTTGATAGTTGTGTTTTAAGCGGTAAATAAATAATTTGCGTAT
GTGTCGAGGGTGGTTTTTTATTTTATGTGTTTTGTTTTTTTCGTGACGTCAATTCGGTCG
ATGGGTTGTAGTTAATTAGGGAGTGATACGTTTTAGGCGAAGGAGAATTTTTTTTAATAG
GGACGGGGTTTCGTTTTCGTTTTTTTTTGTTTTTTGTATTTTGGTTTTTGAAGATGTAAG
TAATAAAGTTTTGTCGTAGGAGATTTTGGTTTGTTGCGTTTTTTTTGGTCGGTCGTGAGA
ACGCGTTTAATAATACGTGTTGTTTTAAGCGTTTTTGGGAGTTTAGTAGTGGTGGTTTTG
TAAGATTGGGTTTATTGATGTTGTTTTTTTTTTTTTTGCTGTTTTTTATTTAAGGAAGGA
TGGTGAGAGGTAGGGTATAGAATTTTTATTTGAGTATTTAGTTATTTTATGTTATACGTG
TGCGGTTTTGTTTTGATTGTTGTGTAGAGTAAGGTTAGAGTG
>CatRLTRTestis6_M13R
GGGAGATATGTTATTTTTTATGAAGGTTTAGTGTTTTAGTTTTTTTTTTTTTAGGTAAAA
CGATACGGGAGTAGGTTAGGGTTGTTTTGGGTAAAAGTTTGTGAGTTTAAGAGTTAATTT
TGTATATGGTTTTTTTATTTATATATTGGGGATTTGATTTTTATTTTTATTTTTATTAAT
ATGGGTGGTTTATTTGTTTTTATTAAAAGGAAAGGGGGAGATGTTGGGAGTCGCGTTTAT
ATTCGTCGTTATAAGATGGCGTTGATAGTTGTGTTTTAAGTGGTAAATAAATAATTTGCG
TATGTGTCGAGGGTGGTTTTTTATTTTATGTGTTTTGTTTTTTTCGTGACGTTAATTCGG
TCGATGGGTTGTAGTTAATTAGGGAGTGATACGTTTTAGGCGAAGGAGAATTTTTTTTAA
TGGGGACGGGGTTTCGTTTTCGTTTTTTTTTGTTTTTTGTATTTTGGTTTTTGAAGATGT
AAGTAATAAAGTTTTGTCGTAGAAGATTTTGGTTTGTTGCGTTTTTTTTGGTCGGTCGTG
AGAACGCGTTTAATAATACGTGTTGTTTTAAGCGTTTTTGGGAGTTTAGTAGTGGTGGCT
TTGTAAGATTGGGTTTATTGATGTTGTTTTTTTTTTTCTTTGTTGTTTTTTATTTAAGGA
AGGATGGTGAGAGGTAGGGTATAGAATTTTTATTTGAGTATTTAGTTATTTTATGTTATA
CGTGTGCGGTTCTGTTTTGATTGTTGTGTAGAGTAAGGTTAGAGT
>CatRLTRTestis7_M13R
GGGAGATATGTTATTTTTTATGAAGGTTTAGTGTTTTAGTTTCTTTTTTTTAGGTAAAAC
GATACGGGAGTAGGTTAGGGTTGTTTTGGGTAAAAGTTTGTGAGTTTAAGAGTTAATTTT
GTATATGGTTTTTTTATTTATATATTGGGGATTTGATTTTTATTTTTATTTTTATTAATA
TGGGTGGTTTATTTGTTTTTATTAAAAGGAAAGGGGGAGATGTTGGGAGTCGCGTTTATA
TTCGTCGTTATAAGATGGCGTTGATAGTTGTGTTTTAAGTGGTAAATAAATAATTTGCGT
ATGTGTCGAGGGTGGTTTTTTATTTTATGTGTTTTGTTTTTTTCGTGACGTTAATTCGGT
CGATGGGTTGTAGTTAATTAGGGAGTGATACGTTTTAGGCGAAGGAGAATTTTTTTTAAT
AGGGACGGGGTTTTGTTTTCGTTTTTTTTTGTTTTTTGTATTTTGGTTTTTGAAGATGTA
AGTAATAAAGTTTTGTCGTAGAAGATTTTGGTTTGTTGCGTTTTTTTTGGTCGGTCGTGA
GAACGCGTTTAATAATACGTGTTGTTTTAAGTGTTTTTGGGAGTTTAGTAGTGGTGGTTT
TGTAAGATTGGGTTTATTGATGTTGTTTTTTTTTTTTTGTTGTTTTTTATTTAAGGAAGG
ATGGTGAGAGGTAGGGTATAGAATTTTTATTTGAGTATTTAGTTATTTTATGTTATACGT
GTGCGGTTTTGTTTTGATTGTTGTGTAGAGTAAGGTTAGAGTG
>CatRLTRTestis8_M13R
GGGAGATATGTTATTTTTTATGAAGGTTTAGTGTTTTAGTTTTTTTTTTTAGGTAAAACG
ATACGGGAGTAGGTTAGGGTTGTTTTGGGTAAAAGTTTGTGAGTTTAAGAGTTAATTTTG
TATATGGTTTTTTTATTTATATATTGGGGATTTGATTTTTATTTTTATTTTTATTAATAT
GGGTGGTTTATTTGTTTTTATTAAAAGGAAAGGGGGAGATGTTGGGAGTCGCGTTTATAT
TCGTCGTTATAAGATGGCGTTGATAGTTGTGTTTTAAGTGGTAAATAAATAATTTGCGTA
TGTGTCGAGGGTGGTTTTTTACTTTATGTGTTTTGTTTTTTTCGTGACGTTAATTCGGTC
GATGGGTTGTAGTTAATTAGGGAGTGATACGTTTTAGGCGAAGGAGAATTTTTTTTAATA
GGGACGGGGTTTCGTTTTCGTTTTTTTTTGTTTTTTGTATTTTGGTTTTTGAAGATGTAA
GTAATAAAGTTTTGTCGTAGAAGATTTTGGTTTGTTGCGTTTTTTTTGGTCGGCCGTGAG
AACGCGTTTAATAATACGTGTTGTTTTAAGCGTTTTTGGGAGTTTAGTAGTGGTGGTTTT
GTAAGATTGGGTTTATTGATGTTGTTTTTTTTTTTTTTTGTTGTTTTTTATTTAAGGAAG
GATGGTGAGAGGTAGGGTATAGAATTTTTATTTGAGTATTTAGTTATTTTATGTAATACG
TGTGCGGTTTTGTTTTGATTGTTGTGTAGAGTAAGGTTAGAGTG
>CatRLTRTestis9_M13R
GGGAGATATGTTATTTTTTATGAAGGTTTAGTGTTTTAGTTTTTTTTTTTTAGGTAAAAC
GATACGGGAGTAGGTTAGGGTTGTTTTGGGTAAAAGTTTGTGAGTTTAAGAGTTAATTTT
GTATATGGTTTTTTTATTTATATATTGGGGATTTGATTTTTATTTTTATTTTTATTAATA
TGGGTGGTTTATTTGTTTTTATTAAAAGGAAAGGGGGAGATGTTGGGAGTCGCGTTTATA
TTCGTCGTTATAAGATGGCGTTGATAGTTGTGTTTTAAGTGGTAAATAAATAATTTGCGT
ATGTGTCGAGGGTGGTTTTTTATTTTATGTGTTTTGTTTTTTTCGTGACGTTAATTCGGT
CGATGGGTTGTAGTTAATTAGGGAGTGATACGTTTTAGGCGAAGGAGAATTTTTTTTAAT
AGGGACGGGGTTTCGTTTTCGTTTTTTTTTGTTTTTTGTATTTTGGTTTTTGAAGATGTA
AGTAATAAAGTTTTGTCGTAGAAGATTTTGGTTTGTTGCGTTTTTTTTGGTCGGTCGTGA
GAACGCGTTTAATAATACGTGTTGTTTTAAGCGTTTTTGGGAGTTTAGTAGTGGTGGTTT
TGTAAGATTGGGTTTATTGATGTTGTTTTTTTTTTTTTTTGTTGTTTTTTATTTAAGGAA
GGATGGTGAGAGGTAGGGTATAGAATTTTTATTTGAGTATTTAGTTATTTTATGTTATAC
GTGTGCGGTTTTGTTTTGATTGTTGTGTAGAGTAAGGTTAGAGTG
>CatRLTRTestis10_M13R
GGGAGATATGTTATTTTTTATGAAGGTTTAGTGTTTTAGTTTTTTTTTTTTAGGTAAAAC
GATACGGGAGTAGGTTAGGGTTGTTTTGGGTAAAAGTTTGTGAGTTTAAGAGTTAATTTT
GTATATGGTTTTTTTATTTATATATTGGGGATTTGATTTTTATTTTTATTTTTATTAATA
TGGGTGGTTTATTTGTTTTTATTAAAAGGAAAGGGGGAGATGTTGGGAGTCGCGTTTATA
TTCGTCGTTATAAGATGGCGTTGATAGTTGTGTTTTAAGTGGTAAATAAATAATTTGCGT
ATGTGTCGAGGGTGGTTTTTTATTTTATGTGTTTTGTTTTTTTCGTGACGTTAATTCGGT
CGATGGGTTGTAGTTAATTAGGGAGTGATACGTTTTAGGCGAAGGAGAATTTTTTTTAAT
AGGGACGGGGTTTCGTTTTCGTTTTTTTTTGTTTTTTGTATTTTGGTTTTTGAAGATGTA
AGTAATAAAGTTTTGTCGTAGAAGATTTTGGTTTGTTGCGTTTTTTTTGGTCGGTCGTGA
GAACGCGTTTAATAATACGTGTTGTTTTAAGCGTTTTTGGGAGTTTAGTAGTGGTGGTTT
TGTAAGATTGGGTTTATTGATGTTGTTTTTTTTTTTTTTTGTTGTTTTTTATTTAAGGAA
GGATGGTGAGAGGTAGGGTATAGAATTTTTATTTGAGTATTTAGTTATTTTATGTTATAC
GTGTGCGGTTTTGTTTTGATTGTTGTGTAGAGTAAGGTTAGAGTG
>CatRLTRTestis11_M13R
GGGAGATATGTTATTTTTTATGAAGGTTTAGTGTTTTAGTTTTTTTTTTTAGGTAAAACG
ATACGGGAGTAGGTTAGGGTTGTTTTGGGTAAAAGTTTGTGAGTTTAAGAGTTAATTTTG
TATATGGTTTTTTTATTTATATATTGGGGATTTGATTTTTATTTTTATTTTTATTAATAT
GGGTGGTTTATTTGTTTTTATTAAAAGGAAAGGGGGAGATGTTGGGAGTCGCGTTTATAT
TCGTCGTTATAAGATGGCGTTGATAGTTGTGTTTTAAGTGGTAAATAAATAATTTGCGTA
TGTGTCGAGGGTGGTTTTTTATTTTATGTGTTTTGTTTTTTTCGTGACGTTAATTCGGTC
GATGGGTTGTAGTTAATTAGGGAGTGATACGTTTTAGGCGAAGGAGAATTTTTTTTAATA
GGGACGGGGTTTCGTTTTCGTTTTTTTTTGTTTTTTGTATTTTGGTTTTTGAAGATGTAA
GTAATAAAGTTTTGTCGTAGAAGATTTTGGTTTGTTGCGTTTTTTTTGGTCGGTCGTGAG
AACGCGTTTAATAATACGTGTTGTTTTAAGCGTTTTTGGGAGTTTAGTAGTGGTGGTTTT
GTAAGATTGGGTTTATTGATGTTGTTTTTTTTTTTTTTGTTGTTTTTTATTTAAGGAAGG
ATGGTGAGAGGTAGGGTATAGAATTTTTATTTGAGTATTTAGTTATTTTATGTTATACGT
GTGCGGTTTTGTTTTGATTGTTGTGTAGAGTAAGGTTAGAGTG
