# Supplementary material for: Epigenetic interplay between mouse endogenous retroviruses and host genes
Source: Genome Biol. 2012 Oct 3;13(10):R89. doi: 10.1186/gb-2012-13-10-r89 (PMC3491417; doi:10.1186/gb-2012-13-10-r89)
Supplement: Additional file 4 — All bisulfite sequencing data. Compilation of all bisulfite sequences. [file gb-2012-13-10-r89-S4.zip › IAP8545_gene_ES.rtf]

100922 & 101019
Pnp CGI B6 ES
>PnpES_22
AGGGTTTTTTTTAAATTTAAATATTTTTTTTAAGGTAGTTTTTATTTTTTATTGATAAGG
GTGGGGGAATAAAAGTTGATTGAAAGAAATGTATTTTGGTGGTTTGATTTTGGTAATTAT
AGAAAGTTTGTAAGGATTGTTTTTTTGGTAGTTTGTAGGTTTTATGGAAATGAAATTGTG
TTTGATTTTGTTTATAGGAGTGTTGGTTAATGAGGTGTTGTTTAGGTTTTTTATGTTTTG
GGTGTTATGGTGGTTTGTAGGTTGTGTTGTTTGTGTTTGTGTCTTTGATTGTTGGGTTGT
GGTTTTTTTGGTTGGTTTGGGTGGAATTGGGTATTTAGTTATTTGTAGATGTGAGTGTTG
TGGAGTAGTATTGGATTTAGAGTGGTGATTGTAGATTTGGGATATAGGTGAG
>PnpES_23
AGGGTTTTTTTTAAATTTATATATTTTTTTTAAGGTAGTATTTATTTTTTATCGATAAGG
GTGGGGGAATAAAAGTTGATTGAAAGAAATGTATTTTGGTGGTTTGATTTTGGTAATTAT
AGAAAGTTTGTAAGGATTGTTTTTTTGGTAGTTTGTAGGTTTTATGGAAATGAAATTGTG
TTTGATTTTGTTTATAGGAGTGTTGGTTAATGAGGTGTTGTTTAGGTTTTTTATGTTTTG
GGTGTTATGGTGGTTTGTAGGTTGTGTTGTTTGTGTTTGTGTTTTTGATTGTTGGGTTGT
GGTCTTTTTGGTTGGTTTGGGCGGAATTGGGTATTTAGTTACTTTGTACGATGTGAGTGT
TGTGGAGTACGTACTTGGATTTACGAGTGGTGATTGTAGATTTGGGATAATAGGTGAGA
>PnpES_24
AGGGTTTTTTTTAAATTTATATATTTTTTTTAAGGTAGTATTTATTTTTTATTGATAAGG
GTGGGGGAATAAAAGTTGATTGAAAGAAATGTATTTTGGTGGTTTGATTTTGGTAATTAT
AGAAAGTTTGTAAGGATTGTTTTTTTGGTAGTTTGTAGGTTTTATGGAAATGAAATTGTG
TTTGATTTTGTTTATAGGAGTGTTGGTTAATGAGGTGTTGTTTAGGTTTTTTATGTTTTG
GGTGTTATGGTGGTTTGTAGGTTGTGTTGTTTGTGTTTGTGTTTTTGATTGTTGGGTTGT
GGTTTTTTTGGTTGGTTTGGGTGGAATTGGGTATTTAGTTATTTGTAGATGTGAGTGTTG
TGGAGTAGTATTGGATTTAGAGTGGTGATTGTAGATTTGGGATATAGGTGAGA
>PCGI_21 10 19 10
AGGGTTTTTTTTAAATTTATATATTTTTTTTAAGGTAGTATTTATTTTTTATCGATAAGG
GTGGGGGAATAAAAGTTGATTGAAAGAAATGTATTTTGGTGGTTTGATTTTGGTAATTAT
AGAAAGTTTGTAAGGATTGTTTTTTTGGTAGTTTGTAGGTTTTATGGAAATGAAATTGTG
TTTGATTTTGTTTATAGGAGTGTTGGTTAATGAGGTGTTGTTTAGGTTTTTTATGTTTTG
GGTGTTGTGGTGGTTTGTAGGTTGTGTTGTTTGTGTTTGTGTTTTTGATTGTTGGGTTGT
GGTTTTTTTGGTTGGTTTGGGTGGAATTGGGTATTTAGTTATTTGTAGATGTGAGTGTTG
TGGAGTAGTATTGGATTCAGAGTGGTGATTGTAGATTTGGGATATAGGTGAGAA
>PCGI_23 10 19 10
AGGGTTTTTTTTAAAATTTATATATTTTTTTTAAGGTAGTATTTATTTTTTCATTGATAA
GGGTGGGGGAATAAAAGTTGATTGAAAGAAATGTATTTTGGTGGTTTGATTTTGGTAATT
ATAGAAAGTTTGTAAGGATTGTTTTTTTGGTAGTTTGTAGGTTTTATGGAAATGAAATTG
TGTTTGATTTTGTTTATAGGAGTGTTGGTTAATGAGGTGTTGTTTAGGTTTTTTATGTTT
TGGGTGTTATGGTGGTTTGTAGGTTGTGTTGTTTGTGTTTGTGTTTTTGATTGTTGGGTT
GTGGTTTTTTTGGTTGGTTTGGGTGGAATTGGGTATTTAGTTATTTGTAGATGTGAGTGT
TGTGGAGTAGTATTGGATTTACGACGTGGTGATTGTAGATTTGGGATATAGGTGAGAA
>PCGI_24 10 19 10
AGGGTTTTTTTTAAATTTATATATTTTTTTTAAGGTAGTATTTATTTTTTATTGATAAGG
GTGGGGGAATAAAAGTTGATTGAAAGAAATGTATTTTGGTGGTTTGATTTTGGTAATTAT
AGAAAGTTTGTAAGGATTGTTTTTTTGGTAGTTTGTAGGTTTTATGGAAATGAAATTGTG
TTTGATTTTGTTTATAGGAGTGTTGGTTAATGAGGTGTTGTTTAGGTTTTTTATGTTTTG
GGTGTTATGGTGGTTTGTAGGTTGTGTTGTTTGTGTTTGTGTTTTTGATTGTTGGGTTGT
GGTTTTTTTGGTTGGTTTGGGTGGAATTGGGTATTTAGTTATTTGTAGATGTGAGTGTTG
TGGAGTAGTATTGGATTTAGAGTGGTGATTGTAGATTTGGGATATAGGTGAGAA
>PCGI_25 10 19 10
AGGGTTTTTTTAAATTTATATATTTTTTTTTAAGGTAGTATTTATTTTTTATTGATAAGG
GTGGGGGAATAAACAGTTGATTGAAAGAAATGTATTTTGGTGGTTTGATTTTGGTAATAT
AGAAAGTTAGTAAGGATTGTTTTTTTTGGTAGTTTGTAGGTTTTATGGAAATGAAATTGT
GTTTGATTTTGTTTATAGGAGTGTTGGTTAATGAGGTGTTGTTTAGGTTTTTTATGTTTT
GGGTGTTATGGTGGTTTGTAGGTTGTGTAGTTTGTGTTTGTGTTTTTGATTGTTGGGTTG
TGGTTTTTTTGGTTGGTTTGGGTGGAATTGGGTATTTAGTTATTTGTACGATGTGACGTG
TTGTGGAGTAGTATTGGAATTTACGACGTGGTGACTTGTAAGAATTTGGGACTATAGGTG
AGAA
