# Supplementary material for: Epigenetic interplay between mouse endogenous retroviruses and host genes
Source: Genome Biol. 2012 Oct 3;13(10):R89. doi: 10.1186/gb-2012-13-10-r89 (PMC3491417; doi:10.1186/gb-2012-13-10-r89)
Supplement: Additional file 4 — All bisulfite sequencing data. Compilation of all bisulfite sequences. [file gb-2012-13-10-r89-S4.zip › IAP8545_TE_ES.rtf]

5/28/10
Pnpt1 3' LTR B6 ES gDNA
>Pnp3LTRES_22
GAGAGTTGTAAGGTTAAGTATTGTAATGGAAAGGTTTTGCGGTATATATGAGTTTATTTT
AGGGAGATATGTTATTTTTTGTGAAGGTTTAGTGTTTTAGTTTTTTTTTTAGGTAAAACG
ATATGGGAGTAGGTTAGGGTTGTTTTGGGTAAAAGTTTGTAAGTTTAAGAGTTAATTTTG
TATATGGTTTTTTTATTTATATATTGGGGATTTGATTTTTATTTTTATTTTTATTAATAT
GGGTGGTTTATTTGTTTTTATTAAAAGAAAAAGGGGGAGATGTTGGGAGTCGTTTTTATA
TTCGTCGTTATAAGATGGTGTTGATATTTTGTGTTTTAAGTGGTAAATAAATAATTTGCG
TATGTGTTAAGGGTATTTTATGATTATTTGTGTTTTGTTTTTTTCGTGACGTTAATTCGG
TCGATGGGTTGCAGTTAATTAAGGAGTGATATGTTCGAGGCGAAGGAGAATGTTTTTTAA
GAGGGACGGGGTTTTCGTTTTTTTTTTTTGTTTTTTGTTTTTTTTTTTTGAAGATGTAAG
AATAAAGTTTTGTCGTAGAAGATTTTGGTTTGTGGTGTTTTTTTTGGTCGGTTGTGAGAA
CGCGTCGAATAATATTAATGTTTTTATTTTTTTTTGATTTATGATTTAAAATTATTATTA
TTTTAATTATTTTAGAGTGATATTAGGAAATATAGATTTTTTTTTAT
>Pnp3LTRES_21
GAGAGTTGTAAGGTTAAGTATTGTAATGGAAAGGTTTTGTGGTATATATGAGTTTATTTT
AGGGAGATATGTTATTTTTTATGAAGGTTTAGTGTTTTAGTTTTTTTTTTTAGGTGAAAC
GATACGGGAGTAGGTTAGGGTTGTTTTGGGTAAAAGTTTGTAAGTTTAAGAGTTAATTTT
GTATATGGTTTTTTTATTTATATATTGGGGATTTGATTTTTATTTTTATTTTTATTAATA
TGGGTGGTTTATTTGTTTTTATTAAAAGAAAAAGGGGGAGATGTTGGGAGTCGTTTTTAT
ATTCGTCGTTATAAGATGGCGTTGATATTTTGTGTTTTAAGTGGTAAATAAATAATTTGC
GTATGTGTTAAGGGTATTTTATGATTATTTGTGTTTTGTTTTTTTCGTGACGTTAATTCG
GTCGATGGGTTGTAGTTAATTAAGGAGTGATATGTTCGGGGCGAAGGAGAATGTTTTTTA
AGAGGGACGGGGTTTTTGTTTTTTTTTTTTTGTTTTTTGTTTTTTTTTTTGAAGATGTAA
GAATAAAGTTTTGTCGTAGAAGATTTTGGTTTGTGGTGTTTTTTTTGGTCGGTCGTGAGA
ACGCGTCGAATAATATTAATGTTTTTATTTTTTTTTGATTTATGATTTAAAATTATTATT
ATTTTAATTATTTTAGAGTGATATTAGGAAATATAGATTTTTTTTTAT
>Pnp3LTRES_19
GAGAGTTGTAAGGTTAAGTATTGTAATGGAAAGGTTTTGCGGTATATATGAGTTTATTTT
AGGGAGACATGTTATTTTTTATGAAGGTTTAGTGTTTTAGTTTTTTTTTTCAGGTAAAAC
GATACGGGAGTAGGTTAGGGTTGTTTTGGGTAAAAGTTTGTAAGTTTAAGAGTTAATTTT
GTATATGGTTTTTTTATTTATATATTGGGGATTTGATCTTTATTTTTATTTTTATTAATA
TGGGTGGTTTATTTGTTTTTATTAAAAGAAAAAGGGGGAGATGTTGGGAGTCGTTTTTAT
ATTCGTCGTTATAAGATGGCGTTGATATTTTGTGTTTTAAGTGGTAAATAAATAATTTGC
GTATGTGTTAAGGGTATTTTATGATTATTTGTGTTTTGTTTTTTTCGTGACGTTAATTCG
GTCGATGGGTTGTAGTTAATTAAGGAGTGATACGTTCGAGGCGAAGGAGAATGTTTTTTA
AGAGGGACGGGGTTTTCGTTTTTTTTTTTTTTTGTTTTTTGTTTTTTTTTTTGAAGATGT
AAGAATAAAGTTTTGTCGTAGAAGATTTTGGTTTGTGGTGTTTTTTTTGGTCGGTCGTGA
GAACGCGTCGAATAATATTAATGTTTTTATTTTTTTTTGATTTATGATTTAAAATTATTA
TTATTTTAATTATTTTAGAGTGATATTAGGAAATATAGATTTTTTTTTAT
>Pnp3LTRES_17
TAAGGTTAAGTATTGTAATGGAAAGGTTTTGCGGTATATATGAGTTTATTTTAGGGAGAT
ATGTTATTTTTTATGAAGGTTTAGTGTTTTAGTTTTTTTTTAGGTAAAACGATACGGGAG
TAGGTTAGGGTTGTTTTGGGTAAAAGTTTGTAAGTTTAAGAGTTAATCTTGTATATGGTT
TTTTTATTTATATATTGGGGATTTGATTTTTATTTTTATTTTTATTAATATGGGTGGCTT
ATTTGTTTTTATTAAAAGAAAAAGGGGGAGATGTTGGGAGTCGTTCTTATATTCGTCGTT
ATAAGATGGCGTTGATATTTTGTGTTTTAAGTGGTAAATAAATAATTTGCGTATGTGTTA
AGGGTATTTTATGATTATTTGTGTTTTGTTTTTTTCGTGACGTTAATTCGGTCGATGGGT
TGTAGTTAATTAAGGAGTGATACGTTCGAGGCGAAGGAGAATGTTTTTTAAGAGGGACGG
GGTTTTCGTTTTTTTTTTTTTTTGTTTTTTGTTTTTTTTTTTGAAGATGTAAGAATAAAG
TTTTGTCGTAGAAGATTTTGGTTTGTGGTGTTTTTTTTGGTCGGTCGTGAGAACGCGTCG
AATAATATTAATGTTTTTATTTTTTTTTGATTTATGATTTAAAATTATTATTATTTTAAT
TATTTTAGAGTGATATTAGGAAATATAGATTTTTTTTTAT
>Pnp3LTRES_15
GAGAGTTGTAAGGTTAAGTATTGTAATGGAAAGGTTTTGCGGTATATATGAGTTTATTTT
AGGGAGATATGTTATTTTTTATGAAGGTTTAGTGTTTTAGTTTTTTTTTTTTAGGTAAAA
CGATACGGGAGTAGGTTAGGGTTGTTTTGGGTAAAAGTTTGTAAGTTTAAGAGTTAATTT
TGTATATGGTTTCCTTATTTATATATTGGGGATTTGATTTTTATTTTTATTTTTATTAAT
ATGGGTGGTTTATTTGTTTTTATTAAAAGAAAAAGGGGGAGATGTTGGGAGTTGTTTTTA
TATTCGTCGTTACAAGATGGCGTTGATACTTTGTGTTTTAAGTGGTAAATAAATAATTTG
TGTATGTGTTAAGGGTATTTTATGATTATTTGTGTTTTGTTTTTTTCGTGACGTTAATTC
GGTCGATGGGTTGTAGTTAATTAAGGAGTGATACGTTCGAGGCGAAGGAGAATGTTTTTT
AAGAGGGACGGGGTTTTCGTTTTTTTTTTTTTTGTTTTTTGTTTTTTTTTTTTGAAGATG
TAAGAATAAAGTTTTGTTGTAGAAGATTCTGGTCTGTGGTGTTTTTTTTGGTCGGTCGTG
AGAACGCGTCGAATAATATTAATGTTTTTATTTTTTTTTGATTTATGATTTAAAATTATT
ATTATTTTAATTATTTTAGAGTGATATTAGGAAATATAGATTTTTTTTTAT
>Pnp3LTRES_14
GAGAGTTGTAAGGTTAAGTATTGTAATGGAAAGGTTTTGCGGTATATATGAGTTTATTTT
AGGGAGATATGTTATTTTTTATGAAGGTTTAGTGTTTTAGTTTTTTTTTTTAGGTAAAAC
GATACGGGAGTAGGTTAGGGTTGTTTTGGGTAAAAGTTTGTAAGTTTAAGAGTTAATTTT
GTATATGGTTCTTTTATTTATATATTGGGGATTTGATTTTTATTTTTATTTTTATTAATA
TGGGTGGTTTATTTGTTTTTATTAAAAGAAAAAGGGGGAGATGTTGGGAGTCGTTTTTAT
ATTCGTCGTTATAAGATGGCGTTGATATTTTGTGTTTTAAGTGGTAAATAAATAATTTGC
GTATGTGTTAAGGGTATTTTATGATTATTTGTGTTTTGTTTTTTTCGTGATGTTAATTCG
GTCGATGGGTTGTAGTTAATTAAGGAGTGATACGTTCGAGGCGAAGGAGAATGTTTTTTA
AGAGGGACGGGGTTTCCGTTTTTTTTTTTTTGTTTTTTGTTTTTTTTTTTTGAAGATGTA
AGAATAAAGTTTTGTCGTAGAAGATTTTGGTTTGTGGTGTTTTTTTGGTCGGTCGTGAGA
ACGCGTCGAATAATATTAATGTTTTTATTTTTTTTTGATTTATGATTTAAAATTATTATT
ATTTTAATTATTTTAGAGTGATATTAGGAAATATAGATTTTTTTTTAT
>Pnp3LTRES_9
GAGAGTTGTAAGGTTAAGTATTGTAATGGAAAGGTTTTGCGGTATATATGAGTTTATTTT
AGGGAGATATGTTATTTTTTATGAAGGTTTAGTGTTTTAGTTTTTTTTTAGGTAAAACGA
TACGGGAGTAGGTTAGGGTTGTTTTGGGTAAAAGTTTGTAAGTTTAAGAGTTAATTTTGT
ATATGGTTTTTTTATTTATACNTTGGGGATTTGATTTTTATTTTTATTTTTATTAATATG
GGTGGTTTATTTGTTTTTATTAAAAGAAAAAGGGGGAGATGTTGGGAGTCGTTTTTATAT
TCGTCGTTATAAGATGGCGTTGATATTTTGTGTTTTAAGTGGTAAATAAATAATTTGCGT
ATGTGTTAAGGGTATTTTATGATTATTTGTGTTTTGTTTTTTTCGTGACGTTAATTCGGT
CGATGGGTTGTAGTTAATTAAGGAGTGATACGTTCGAGGCGAAGGAGAATGTTTTTTAAG
AGGGACGGGGTTTTCGTTTTTTTTTTTTTTGTTTTTTGTTTTTTTTTTTTGAAGATGTAA
GAATAAAGTTTTGTCGTAGAAGATTTTGGTTTGTGGTGTTTTTTTTGGTCGGTCGTGAGA
ACGCGTCGAATAATATTAATGTTTTTATTTTTTTTTGATTTATGATTTAAAATTATTATT
ATTTTAATTATTTTAGAGTGATATTAGGAAATATAGATTTTTTTTTAT
>Pnp3LTRES_8
GAGAGTTGTAAGGTTAAGTATTGTAATGGAAAGGTTTTGCGGTATATATGAGTTTATTTT
AGGGAGATATGTTATTTTTTATGAAGGTTTAGTGTTTTAGTTTTTTTTTTTTTAGGTAAA
ACGATACGGGAGTAGGTTAGGGTTGTTTTGGGTAAAAGCTTGTAAGTTTAAGAGTTAATT
TTGTATATGGTTTTTTTATTTATATATTGGGGATTTGATTTTNATTTTTATTTTTATTAA
TATGGGTGGTTTATTTGTTTTTATTAAAAGAAAAAGGGGGAGATGTTGGGAGTCGTTTTT
ATATTCGTTGTTATAAGATGGCGTTGATATTTTGTGTTTTAAGTGGTAAATAAATAATTT
GCGTATGTGTTAAGGGTATTTTATGATTATTTGTGTTTTGTTTTTTTCGTGACGTTAATT
CGGTCGATGGGTTGTAGTTAATTAAGGAGTGATACGTTCGAGGCGAAGGAGAATGTTTTT
TAAGAGGGATGGGGTTTTTGTTTTTTTTTTTTTTTTGTTTTTTGTTTTTTTTTGAAGATG
TAAGAATAAAGTTTTGTCGTAGAAGATTTTGGTTTGTGGTGTTTTTTTTGGTCGGTCGTG
AGAACGCGTCGAATAATATTAATGTTTTTATTTTTTTTTGATTTATGATTTAAAATTATT
ATTATTTTAATTATTTTAGAGTGATATTAGGAAATATAGATTTTTTTTTAT
>Pnp3LTRES_7
GAGAGTTGTAAGGTTAAGTATTGTAATGGAAAGGTTTTGCGGTATATATGAGTTTATTTT
AGGGAGATATGTTATTTTTTATGAAGGTTTAGTGTTTTAGTTTTTTTTTTTAGGTAAAAC
GGTACGGGAGTAGGTTAGGGTTGTTTTGGGTAAAAGTTTGTAAGTTTAAGAGTTAATTTT
GTATATGGTTTTTTTATTTATATATTGGGGATTTGATTTTTATTTTTATTTTTATTAATA
TGGGTGGTTTATTTGTTTTTATTAAAAGAAAGAGGGGGAGATGTTGGGAGTCGTTTTTAT
ATTCGTCGTTATAAGATGGCGTTGATATTTTGTGTTTTAAGTGGTAAATAAATAATTTGC
GTATGTGTTAAGGGTATTTTATGATTATTTGTGTTTTGTTTTTTTCGTGACGTTAATTCG
GTCGATGGGTTGCAGTTAATTAAGGAGTGATACGTTCGAGGCGAAGGAGAATGTTTTTTA
AGAGGGACGGGGTTTTTGTTTTTTTTTTTTTGTTTTTTGTTTTTTTTTTTTGAAGATGTA
AGAATAAAGTTTTGTTGTAGAAGATTTTGGTTTGTGGTGTTTTTTTTGGTCGGTCGTGAG
AACGCGTCGAATAATATTAATGTTTTTATTTTTTTTTGATTTATGATTTAAAATTATTAT
TATTTTAATTATTTTAGAGTGATATTAGGAAATATAGATTTTTTTTTAT
>Pnp3LTRES_6
GAGAGTTGTAAGGTTAAGTATTGTAATGGAAAGGTTTTGCGGTATATATGAGTTTATTTT
AGGGAGATATGTTATTTTTTATGAAGGTTTAGTGTTTTAGTTTTTTTTTTTAGGTAAAAC
GATACGGGAGTAGGTTAGGGTTGTTCTGGGTAAAAGTTTGTAAGTTTAAGAGTTAATTTT
GTATATGGTTTTTTTATTTATATATTGGGGATTTGATTTTTATTTTTATTTTTATTAATA
TGGGTGGTTTATTTGTTTTTATTAAAAGAAAAAGGGGGAGATGTTGGGAGTCGTTTTTAT
ATTCGTCGTTATAAGATGGCGTTGATATTTTGTGTTTTAAGTGGTAAATAAATAATTTGC
GTATGTGTTAAGGGTATTTTATGATTATTTGTGTTTTGTTTTTTTCGTGACGTTAATTCG
GTCGATGGGTTGTAGTTAATTAAGGAGTGATACGTTCGAGGCGAAGGAGAATGTTTTTTA
AGAGGGACGGGGTTTTCGTTTTTTTTTTTTTGTTTTTTGTTTTTTTTTTTTGAAGATGTA
AGAATAAAGTTTTGTCGTAGAAGATTTTGGTTTGTGGTGTTTTTTTTGGTCGGTCGTGAG
AACGCGTCGAATAATATTAATGTTTTTATTTTTTTTTGATTTATGATTTAAAATTATTAT
TATTTTAATTATTTTAGAGTGATATTAGGAAATATAGATTTTTTTTTAT
>Pnp3LTRES_5
GAGAGTTGTAAGGTTAAGTATTGTAATGGAAAGGTTTTGCGGTATATATGAGTTTATTTT
AGGGAGATATGTTATTTTTTATGAAGGTTTAGTGTTTTAGTTTTTTTTTTTTAGGTAAAA
CGATACGGGAGTAGGTTAGGGTTGTTTTGGGTAAAAGTTTGTAAGTTTAAGAGTTAATTC
TGTATATGGTTTTTTTATTTATATATTGGGGATTTGATTTTTATTTTTATTCTTATTAAT
ATGGGTGGTTTATTTGTTTTTATTAAAAGAAAAAGGGGGAGATGTTGGGAGTCGTTTTTA
TATTCGTCGTTATAAGATGGCGTTGATATTTTGTGTTTTAAGTGGTAAATAAATAATTTG
CGTATGTGTTAAGGGTATTTTATGATTATTTGTGTTTTGTTTTTTTCGTGACGTTAATTC
GGTCGATGGGTTGTAGTTAATTAAGGAGTGATACGTTCGAGGCGAAGGAGAATGTTTTTT
AAGAGGGACGGGGTTTTCGTTTTTTTTTTTTTTGTTTTTTGTTTTTTTTTTTGAAGATGT
AAGAATAAAGTTTTGTCGTAGAAGATTTTGGTTTGTGGTGTTTTTTTTGGTCGGTCGTGA
GAACGCGTCGAATAATATTAATGTTTTTATTTTTTTTTGATTTATGATTTAAAATTATTA
TTATTTTAATTATTTTAGAGTGATATTAGGAAATATAGATTTTTTTTTAT
>Pnp3LTRES_2
GAGAGTTGTAAGGTTAAGTATTGTAATGGAAAGGTTTTGCGGTATATATGAGTTTATTTT
AGGGAGATATGTTATTTTTTATGAAGGTTTAGTGTTTTAGTTTTTTTTTTTTTAGGTAAA
ACGATACGGGAGTAGGTTAGGGTTGTTTTGGGTAAAAGTTTGTAAGTTTAAGAGTTAATT
TTGTATATGGTTCTTTTATTTACATATTGGGGATTTGATTTTTATTTTCATTTTTATTAA
TATGGGTGGTTTATTTGTTTTTATTAAAAGAAAAAGGAGGAGATGTTGGGAGTCGTTTTT
ATATTCGTCGTTATAAGATGGCGTTGATATCTTGTGTTTTAAGTGGTAAATAAATAATTT
GCGTATGTGTTAAGGGTATTTTATGATTATTTGTGTTTTGTTTTTTTCGTGACGTTAATT
CGGTCGATGGGTTGTAGTTAATTAAGGAGTGATACGTTCGAGGCGAAGGAGAATGTTTTT
TAAGAGGGACGGGGTTTTCGTTTTTTCTTTTTTTTGTTTTTTGTTTTTTTTTTTTGGAGA
TGTAAGAATAAAGTTTTGTCGTAGAAGATTTTGGTTTGTGGTGTTTTTTCTGGTCGGTCG
TGAGAACGCGTCGAATAATATTAATGTTTTTATTTTTTTTTGATTTATGATTTAAAATTA
TTATTATTCTAATTATTTTAGAGTGATATTAGGAAATATAGATTTTTTTTTAT
