# Supplementary material for: Epigenetic interplay between mouse endogenous retroviruses and host genes
Source: Genome Biol. 2012 Oct 3;13(10):R89. doi: 10.1186/gb-2012-13-10-r89 (PMC3491417; doi:10.1186/gb-2012-13-10-r89)
Supplement: Additional file 4 — All bisulfite sequencing data. Compilation of all bisulfite sequences. [file gb-2012-13-10-r89-S4.zip › IAP9963_transitionregion_brain.rtf]

>1
ttgtgtag
gatttagaaaaagatgaaaaagtattgaagatttgggagtgggtgagtaataaggatggg
atttttattatagtggaaagtaggttggtttagtgaatatagggtttggttattggtttg
gaatattgtcatttatataatgttagtttatattaaatatttgaatgaaagtttgattgg
tttaagtagatataaaaatgaatggttgatttaggttgttgattggagggggaggatttt
ttttttttatttgtttatttgtttttgagatagggttataagtagtttagagtggttttg
aatttattgtgtagtttagggtgaatttgaatttttaatcgttttgtttttattttttaa
gttgatggtattattggtgtattcgtttttttttgttgttgggataagatataattaagg
taattgttagaaggatttgagtttgtggttttatttgagagggttaggagtttattgtgg
tgggaaagtttggtagggagtagtagttatagaggttggagtagagaggagggggttata
tttagaatttagaagtataaagtagagagattgaatttggaattgtttgtggttttgagt
ttcaaagttaggtttttagtgatatagggattttaataaggttatatatatcttaatagt
gttattaattggttattaagagttaaatatttaagtttatatcgaagttttttatttaaa

>2
ttgtgta
ggatttagaaaaagatgaaaaagtattgaagatttgggagtgggtgagtaataaggatgg
gatttttattatagtggaaagtaggttggtttagtgaatataaggtttggttattggttt
ggaatattgttatttatataatgttagttcatattaaatatttgaatgaaagtttgattg
gtttaagtagatataaaaatgaatggttgatttaggttgttgattggagggggaggattt
ttttttttttatttgtttatttgtttttgagatagggttataagtagtttagagtggttt
tgaatttattgtgtagtttagggtgaatttgaatttttaatcgttttgtttttatttttt
aagttgatggtattattggtgtatttgtttttttttgttgttgggataagatataattaa
ggtaattgttagaaggatttgagtttgtggttttatttgagagggttaggagtttattgt
ggtgggaaagtttggtagggagtagtagttatagaggttggagtagagaggagggggtta
tatttagaatttagaagtataaagtagagagattgaatctggaattgtttgtggttttga
gttttaaagttaggtttttagtgatatagggattttaataaggttatatatattttaata
gtgttattaattggttattaagagttaaatatttaagtttatatcgaagttttttattta
aa
>3
ttgtgtaggatttagaaaaagatgaaaaagtattgaagatttgggagtgggt
gagtaataaggatgggatttttattatagtggaaagtaggttggtttagtgaatataggg
tttggttattggtttggaatattgtcatctatataatgttagtttatattaaatatttga
atgaaagtttgattggtttaagtagatataaaaatgaatggttgatttaggttgttgatt
ggagggggaggatttttttttttttatttgtttatttgtttttgagatagggttataagt
agtttagagtggttttgaatttattgtgtagtttagggtgaatttgaatttttaatcgtt
ttgtttttattttttaagttgatggtattattggtgtattcgttttttttttgttgttgg
gataagatataattaaggtaattgttagaaggatttgagtttgtggttttatttgagagg
gttaggagtttattgcggtgggaaagtttggtagggagtagtagttatagaggttggagt
agagaggagggggttatatttagaatttagaagtataaagtagagagattgaatttggaa
ttgtttgtggttttgagtttcaaagttaggtttttagtgatatagggattttaataaggt
tatatatatcttaatagtgttattaattggttattaagagttaaatatttaagtttatat
cgaagttttttatttaaa

>4
ttgtgtaggatttagaaaaagatgaaaaagtattgaagatttgggagtgggt
gagtaataaggatgggatttttattatagtggaaagtaggttggtttagtgaatataggg
tttggttattggtttggaatattgtcatttatataatgttagtttatattaaatatttga
atgaaagtttgattggtttaagtagatataaaaatgaatggttgatttaggttgttgatt
ggagggggaggattttttttttttatttgtttatttgtttttgagatagggttataagta
gtttagagtggttttgaatttattgtgtagtttagggtgaatttgaatttttaatcgttt
tgtttttattttttaagttgatggtattattggtgtattcgtttttttttgttgttggga
taagatataattaaggtaattgttagaaggatttgagtttgtggttttatttgagagggt
taggagtttattgtggtgggaaagtttggtagggagtagtagttatagaggttggagtag
agaggagggggttatatttagaatttagaagtataaagtagagagattgaatttggaatt
gtttgtggttttgagtttcaaagttaggtttttagtgatatagggattttaataaggtta
tatatatcttaatagtgttattaattggttattaagagttaaatatttaagtttatatcg
aagttttttatttaaa

>5
ttgtgtaggatttagaaaaagatgaaaaagtattgaagatttggg
agtgggtgagtaataaggatgggatttttattatagtggaaagtaggttggtttagtgaa
tatagggtttggttattggtttggaatattgtcatttatataatgttagtttatattaaa
tatttgaatgaaagtttgattggtttaagtaggtataagaatgagtggttgatttaggtt
gttgattggagggggaggattttttttttttatttgtttatttgtttttgagatagggtt
ataagtagtttagagtggttttgaatttattgtgtagtttagggtgaatttgaattttta
atcgttttgtttttattttttaagttgatggtattatcggtgtattcgttttttttgttg
ttgggataagatataattaaggtaattgttagaaggatttgagtttgtggttttatttga
gagggttaggagtttattgtggtgggaaagtttggtagggagtagtagttatagaggttg
gagtagagaggagggggttatatttagaatttagaagtataaagtagagagattgaattt
ggaattgtttgtggttttgagtttcaaagttaggtttttagtgatatagggattttaata
aggttatatatatcttaatagtgttattaattggttattaagagttaaatatttaagttt
atatcgaagttttttatttaaa

>6
ttgtgtaggatttagaaaaagatgaaaaagtattgaagatttgggagtg
ggtgagtaataaggatgggatttttattatagtggaaagtaggttggtttagtgaatata
gggtttggttattggtttggaatattgttatttatataatgttagttcatattaaatatt
tgaatgaaagtttgattggtttaagtagatataaaaatgaatggttgatttaggttgttg
attggagggggaggattttttttttttatttgtttatttgtttttgagatagggttataa
gtagtttagagtggttttgaatttattgtgtagtttagggtgaatttgaatttttaatcg
ttttgtttttattttttaagttgatggtattattggtgtatttgtttttttttgttgttg
ggataagatataattaaggtaattgttagaaggatttgagtttgtggttttatttgagag
ggttaggagtttattgtggtgggaaagtttggtagggagtagtagttatagaggttggag
tagagaggagggggttatatttagaatttagaagtataaagtagggagattgaatctgga
attgtttgtggttttgagttttaaagttaggtttttagtgatatagggattttaataagg
ttatatatattttaatagtgttattaattggttattaagagttaaatatttaagtttata
tcgaagttttttatttaaa

>8
ttgt
gtaggatttagaaaaagatgaaaaagtattgaagatttgggagtgggtgagtaataagga
tgggatttttattatagtggaaagtaggttggtttagtgaatatagggtttggttattgg
tttggaatattgtcatttatataatgttagtttatattaaatatttgaatgaaagtttga
ttggtttaagtagatataaaaatgaatggttgatttaggttgttgattggagggggagga
ttttttttttttatttgtttatttgtttttgagatagggttataagtagtttagagtggt
tttgaatttattgtgtagtttagggtgaatttgaatttttaatcgttttgtttttatttt
ttaagttgatggtattattggtgtattcgttttttttttgttgttgggataagatataat
taaggtaattgttagaaggatttgagtttgtggttttatttgagagggttaggagtttat
tgtggtgggaaagtttggtagggagtagtagttatagaggttggagtagagaggaggggg
ttatatttagaatttagaagtataaagtagagagattgaatttggaattgtttgtggttt
tgagtttcaaagttaggtttttagtgatatagggattttaataaggttatatatatctta
atagtgttattaattggttattaagagttaaatatttaagtttatatcgaagttttttat
ttaaa

>9
ttgtgtaggatttagaaa
aagatgaaaaagtattgaagatttgggagtgggtgagtaataaggatgggatttttatta
tagtggaaagtaggttggtttagtgaatataaggtttggttattggtttggaatattgtt
atttatataatgttagttcatattaaatatttgaatgaaagtttgattggtttaagtaga
tataaaaatgaatggttgatttaggttgttgattggagggggaggattttttttttttat
ttgtttatttgtttttgagatagggttataagtagtttagagtggttttgaatttattgt
gtagtttagggtgaatttgaatttttaatcgttttgtttttattttttaagttgatggta
ttattggtgtatttgttttttttttgttgttgggataagatataattaaggtaattgcta
gaaggatttgagtttgtggttttatttgagagggttaggagtttattgtggtgggaaagt
ttggtagggagtagtagttatagaggttggagtagagaggagggggttatatttagaatt
tagaagtataaagtagagagattgaatctggaattgtttgtggttttgagttttaaagtt
aggcttttagtgatatagggattttaataaggttatatatattttaatagtgttattaat
tggttattaagagttaaatatttaagtttatatcgaagttttttatttaaa

>10
ttgtgtaggatttagaaaaagatgaaaaa
gtattgaagatttgggagtgggtgagtaataaggatgggatttttattatagtggaaagt
aggttggtttagtgaatatagggtttggttattggtttggaatattgttatttatataat
gttagttcatattaaatatttgaatgaaagtttgattggtttaagtagatataaaaatga
atggttgatttaggttgttgattggagggggaggatttttttttttttatttgtttattt
gtttttgagatagggttataagtagtttagagtggttttgaatttattgtgtagtttagg
gtgaatttgaatttttaatcgttttgtttttattttttaagttgatggtattattggtgt
atttgtttttttttgttgttgggataagatataattaaggtaattgctagaaggatttga
gtttgtggttttatttgagagggttaggagtttattgtggtgggaaagtttggtagggag
tagtagttatagaggttggagtagagaggagggggttatatttagaatttagaagtataa
agtagagagattgaatctggaattgtttgtggttttgagttttaaagttaggtttttagt
gatatagggattttaataaggttatatatattttaatagtgttattaattggttattaag
agttaaatatttaagtttatatcgaagttttttatttaaa


>ID|7092418 Atxnt-13_SP6_JM288_039.ab1
caaagggcggtttggggagctctcccatatgggtcgacctgcaggcggccgcactagtga
ttctaaacacaaaaccaactattaaccaaatataaaacttacctactataatgatttaaa
taaaaaacttcgatataaacttaaatatttaactcttaataaccaattaataacactatt
aaaatatatataaccttattaaaatccctatatcactaaaaacctaactttaaaactcaa
aaccacaaacaattccagattcaatctctctactttatacttctaaattctaaatataac
cccctcctctctactccaacctctataactactactccctaccaaactttcccaccacaa
taaactcctaaccctctcaaataaaaccacaaactcaaatccttctaacaattaccttaa
ttatatcttatcccaacaacaaaaaaaaaacaaatacaccaataataccatcaacttaaa
aaataaaaacaaaacgattaaaaattcaaattcaccctaaactacacaataaattcaaaa
ccactctaaactacttataaccctatctcaaaaacaaataaacaaataaaaaaaaaaaat
cctccccctccaatcaacaacctaaatcaaccattcatttttatatctacttaaaccaat
caaactttcattcaaatatttaatatgaactaacattatataaataacaatattccaaac
caataaccaaaccctatattcactaaaccaacctactttccactataataaaaatcccat
ccttatcactcacccactcccaaatcttcaatactttttcatctttttctaaatcctaca
caaaatcccggggccatggcggccgggagcaggcgacgtcgggccaattcgccctatagt
gagtcgtattacaatttcatgggcgtcgttttacaacgtcgtggactgggaaaaccctgg
cgttacccaacttaatcgccttgcaacacatccccctttccgcccggtgggggaaatagc
gaaaaagggcccgcaccgattggcccttcccaacaagttggcgcagcccggaagggggaa
aggggacccccccctggtag


>ID|7092431 Atxnt-15_SP6_JM288_055.ab1
caacggcgggtttggggagctctcccatatgggtcgacctgcaggcggccgcactagtga
ttctaaacacaaaaccaactattaaccaaatataaaacttacctactataataatttaaa
taaaaaacttcgatataaacttaaatatttaactcttaataaccaattaataacactatt
aagatatatataaccttattaaaatccctatatcactaaaaacctaactttgaaactcaa
aaccacaaacaattccaaattcaatctctctactttatacttctaaattctaaatataac
cccctcctctctactccaacctctataactactactccctaccaaactttcccaccacaa
taaactcctaaccctctcaaataaaaccacaaactcaaatccttctaacaattaccttaa
ttatatcttatcccaacaacaaaaaaaaaacgaatacaccaataataccatcaacttaaa
aaataaaaacaaaacgattaaaaattcaaattcaccctaaactacacaataaattcaaaa
ccactctaaactacttataaccctatctcaaaaacaaataaacaaataaaaaaaaaaaaa
tcctccccctccaatcaacaacctaaatcaaccattcatttttatatctacttaaaccaa
tcaaactttcattcaaatatttaatataaactaacattatataaatgacaatattccaaa
ccaataaccaaaccctatattcactaaaccaacctactttccactataataaaaatccca
tccttattactcacccactcccaaatcttcaatactttttcatctttttctaaatcctac
acaaatccccgcggccatggcggccgggagcatgcgacgtcgggcccaattcgccctata
gtgagtcgaattacaattcactgggccgtcgttttacaacgtcgtgactggggaaaacct
gggcgtttcccaacttaatcgccttggcaccacatccccccttttcggccgctgggggga
aataagggaaaaagggcccgccaccgattggcccctttccccaacagttggccgcaagcc
ggga

>ID|7092432 Atxnt-20_SP6_JM288_088.ab1
caaagggcggtttgggggagctctcccatatgggtcgacctgcaggcggccgcactagtg
attcctaactttgaaactcaaaaccacaaacaattccaaattcaatctctctactttata
cttctaaattctaaatataaccccctcctctctactccaacctctataactactactccc
taccaaactttcccaccacaataaactcctaaccctctcaaataaaaccacaaactcaaa
tccttctaacaattaccttaattatatcttatcccaacaacaaaaaaaaaacgaatacac
caataataccatcaacttaaaaaataaaaacaaaacgattaaaaattcaaattcacccta
aactacacaataaattcaaaaccactctaaactacttataaccctatctcaaaaacaaat
aaacaaataaaaaaaaaaaatcctccccctccaatcaacaacctaaatcaaccattcatt
tttatatctacttaaaccaatcaaactttcattcaaatatttaatataaactaacattat
ataaatgacaatattccaaaccaataaccaaaccctatattcactaaaccaacctacttt
ccactataataaaaatcccatccttattactcacccactcccaaatcttcaatacttttt
catctttttctaaatcctacacaaaatcccgcggccatggcggccgggagcatgcgacct
cgggcccaattccccctatagtgagtcgtaatacaattcactgggcgtcgttttacaacg
tcctggctgggaaaaccctggcgttacccaacttaatcgccttgcaacacatcccccttt
tgccagctggcgtaaaaacgaagaaagccccacccattgcccttccccaacattgcccaa
cccggatgggcgaagggaacccccctgtaagggcccaataaacccggcggggggtggggg
ttaccccccagcgtggacccctacatttgccaacccccctaacg

>ID|7092433 Atxnt-3_SP6_JM288_057.ab1
gggccttcaaacggggtttggggagctctcccatatgggtcgacctgcaggcggccgcac
tagtgattctaaacacaaaaccaactattaaccaaatataaaacttacctactataatga
tttaaataaaaaacttcgatataaacttaaatatttaactcttaataaccaattaataac
actattaaaatatatataaccttattaaaatccctatatcactaaaaacctaactttaaa
actcaaaaccacaaacaattccagattcaatctctctactttatacttctaaattctaaa
tataaccccctcctctctactccaacctctataactactactccctaccaaactttccca
ccacaataaactcctaaccctctcaaataaaaccacaaactcaaatccttctaacaatta
ccttaattatatcttatcccaacaacaaaaaaaaaacaaatacaccaataataccatcaa
cttaaaaaataaaaacaaaacgattaaaaattcaaattcaccctaaactacacaataaat
tcaaaaccactctaaactacttataaccctatctcaaaaacaaataaacaaataaaaaaa
aaaaaatcctccccctccaatcaacaacctaaatcaaccattcatttttatatctactta
aaccaatcaaactttcattcaaatatttaatatgaactaacattatataaataacaatat
tccaaaccaataaccaaaccttatattcactaaaccaacctactttccactataataaaa
atcccatccttattactcacccactcccaaatcttcaatactttttcatctttttctaaa
tcctacacaaaatcccgcggccatggcggccgggagcatgcgacgtcgggcccaattcgc
cctatagggagtcggaattacaattcactgggccgtcgttttacaacgcctggacgggga
aaaaccctgggggttcccaaacttaatcgccttggaggcacatcccccttttcgcccggc
ggggggtaaaagcgaaaaagggcccgcaccgaattggcccttcccaaacaggtggggcca
gccgggaagggggg

>ID|7092434 Atxnt-10_SP6_JM288_008.ab1
gggaatccaacgccgtttggggagctctcccatatggtcgacctgcaggcggccgcacta
gtgattatctctctactttatacttctaaattctaaatataaccccctcctctctactcc
aacctctataactactactccctaccaaactttctcaccacaataaactcctaaccctct
caaataaaaccacaaactcaaatccttctaacaattaccttaattatatcttatcccaac
aacaaaaaaaaaacgaatacaccaataataccatcaacttaaaaaataaaaccaaaacga
ttaaaaattcaaattcaccctaaactacacaataaactcaaaaccactctaaactactta
taaccctatctcaaaaacaaataaacaaataaaaaaaaaaaaatcctccccctccaatca
acaacctaaatcaaccattcatttttatatctacttaaaccaatcaaactttcattcaaa
tatttaatataaactaacattatataaatgacaatattccaaaccaataaccaaacccta
tattcactaaaccaacctactttccactataataaaaatcccatccttattactcaccca
ctcccaaatcttcaatactttttcatctttttctaaatcctacacaaaatcccgcggcca
tggcggccgggagcatgcgacgtcgggcccaattcgccctatagtgagtcgtattacaat
tcactggccgtcgttttacaacgtcgtgactgggaaaaccctggcgttacccaacttaat
cgccttgcagcacatccccctttcgccagctggcgtaatagcgaagaggcccgcaccgat
cgccctttccaacagttgcgcagcctgaatggcgaagggacgcgccctgtagcggcgcat
taagcgcggcgggggtggtggttacgcgcagcgtgaccgctacacttgccagcgccctag
cgcccgctcctttcgctttcttcccttcctttctcgccccgttcgccgggcttcccccgt
caacttctaaaacggggggctcccttttagggttcccaaattaagtgcttttaacgggca
cc


>ID|7092444 Atxnt-17_SP6_JM288_071.ab1
tgggattccaagggcggttggggagctctcccatatgggtcgacctgcaggcggccgcac
tagtgattacacaaaaccaactattaaccaaatataaaacttacctactataataattta
aataaaaaacttcgatataaacttaaatatttaactcttaataaccaattaataacacta
ttaagatatatataaccttattaaaatccctatatcactaaaaacctaactttgaaactc
aaaaccacaaacaattccaaattcaatctctctactttatacttctaaattctaaatata
accccctcctctctactccaacctctataactactactccctaccaaactttcccaccac
aataaactcctaaccctctcaaataaaaccacaaactcaaatccttctaacaattacctt
aattatatcttatcccaacaacaaaaaaaaacgaatacaccaataataccatcaacttaa
aaaataaaaacaaaacgattaaaaattcaaattcaccctaaactacacaataaattcaaa
accactctaaactacttataaccctatctcaaaaacaaataaacaaataaaaaaaaaaaa
atcctccccctccaatcaacaacctaaatcaaccattcatttttatatctacttaaacca
atcaaactttcattcaaatatttaatataaactaacattatataatggacaatattccaa
accaataaccaaaccctatattcactaaaccaacctactttccactataaaaaaaatccc
atccttattactcacccactcccaaatcttcaatactttttcatctttttctaattccta
cacaaaatcccgcggcctggggggcgggaagcaggcgacgtcgggcccaattcgccccat
ggggagccgaaatacaattaatggggcggcggttttacaacgccggggacggggaaaaac
ccgggggttacccaactttattcgccctggcagaaaaccccccttttcgccagctggggg
aaataggggaaaaggcccggccccgattggcccttccccaaaggtggggggagcccggga
agg

>ID|7092445 Atxnt-4_SP6_JM288_058.ab1
ccacgggcggtttggggagctctcccatatgggtcgacctgcaggcggccgcactagtga
ttctaaacacaaaaccaactattaaccaaatataaaacttacctactataataatttaaa
taaaaaacttcgatataaacttaaatatttaactcttaataaccaattaataacactatt
aagatatatataaccttattaaaatccctatatcactaaaaacctaactttgaaactcaa
aaccacaaacaattccaaattcaatctctctactttatacttctaaattctaaatataac
cccctcctctctactccaacctctataactactactccctaccaaactttcccaccacaa
taaactcctaaccctctcaaataaaaccacaaactcaaatccttctaacaattaccttaa
ttatatcttatcccaacaacaaaaaaaaaacgaatacaccaataataccatcaacttaaa
aaataaaaacaaaacgattaaaaattcaaattcaccctaaactacacaataaattcaaaa
ccactctaaactacttataaccctatctcaaaaacaaataaacaaataaaaaaaaaaaat
cctccccctccaatcaacaacctaaatcaaccattcatttttatatctacttaaaccaat
caaactttcattcaaatatttaatataaactaacattatataaatgacaatattccaaac
cattaaccaaaccctatattcactaaaccaacctactttccactataataaaaatcccat
ccttattactcacccactcccaaatcttcaatactttttcatctttttctaaatcctaca
caaaatcccggggccagggccgggcggggagcaggggacgttcgggcccattttggccct
aagggggggcggaattaaaatttcacggggccggcgttttaaaaccgtcggggacgggga
aaaaccccgggggtttccccaatttaaatcgcctttggggacaaatcc

>ID|7092446 Atxnt-5_SP6_JM288_073.ab1
ggcgtggcctcccacgccggttgtggagctctcccatatgggtcgacctgcaggcggccg
cactagtgattctaaacacaaaaccaactattaaccaaatataaaacttacctactataa
tgatttaaataaaaaacttcgatataaacttaaatatttaactcttaataaccaattaat
aacactattaaaatatatataaccttattaaaatccctatatcactaaaaacctaacttt
aaaactcaaaaccacaaacaattccagattcaatctctctactttatacttctaaattct
aaatataaccccctcctctctactccaacctctataactactactccctaccaaactttc
ccaccacaataaactcctaaccctctcaaataaaaccacaaactcaaatccttctaacaa
ttaccttaattatatcttatcccaacaacaaaaaaaaaacaaatacaccaataataccat
caacttaaaaaataaaaacaaaacgattaaaaattcaaattcaccctaaactacacaata
aattcaaaaccactctaaactacttataaccctatctcaaaaacaaataaacaaataaaa
aaaaaaaaatcctccccctccaatcaacaacctaaatcaaccattcatttttatatctac
ttaaaccaatcaaactttcattcaaatatttaatatgaactaacattatataaataacaa
tattccaaaccaataaccaaaccctatattcactaaaccaacctgctttccactataata
aaaaatcccatccttattactcacccactcccaaatcttcaatactttttcatctttttc
taaatcctaaacaaaatcccgcggccagggcggccgggagcattgcgacgtcgggcccaa
ttcgccctatagtgggtcgtattacaattcactgggcgccgtttttcaacgtcgggacgg
ggaaaaacctggcggtacccaacttaatccgccttggaggcacttcccccttttcggccc
gcggggggtaaaagggaaaaaaggcccgccccggattgcccctttcccaacaattggcgc
cagcccggaaagggc


>ID|7092456 Atxnt-19_SP6_JM288_087.ab1
caacggggggtttggggagctctcccatatgggtcgacctgcaggcggccgcactagtga
ttctaaacacaaaaccaactattaaccaaatataaaacttacctactataatgatttaaa
taaaaaacttcgatataaacttaaatatttaactcttaataaccaattaataacactatt
aaaatatatataaccttattaaaatccctatatcactaaaaacctaactttaaaactcaa
aaccacaaacaattccagattcaatctctctactttatacttctaaattctaaatataat
cccctcctctctactccaacctctataactactactccctaccaaactttcccaccacaa
taaactcctaaccctctcaaataaaaccacaaactcaaatccttctaacaattaccttaa
ttatatcttatcccaacaacaaaaaaaaacaaatacaccaataataccatcaacttaaaa
aataaaaacaaaacgattaaaaattcaaattcaccctaaactacacaataaattcaaaac
cactctaaactacttataaccctatctcaaaaacaaataaacaaataaaaaaaaaaaatc
ctccccctccaatcaacaacctaaatcaaccattcatttttatatctacttaaaccaatc
aaactttcattcaaatatttaatatgaactaacattatataaataacaatattccaaacc
aataaccaaaccctatattcactaaaccaacctactttccactataataaaaatcccatc
cttattactcacccactcccaaatcttcaatactttttcatcttttttctaatcctacac
aaaatcccgcggccatggcggccgggagcatgcgacgtcgggcccaattcgccctatagt
gagtcgtattacaattcactggccgtcgttttacaacgtcgtgaccgggaaaaccctggg
cgttacccaactaaatcgcccttgcagcacatcccccttttcgccagctggggggaataa
gcgaaaagggcccgccccgaatg

>ID|7092457 Atxnt-6_SP6_JM288_074.ab1
aaggggcggtttgagggagctctcccatatgggtcgacctgcaggcggccgcactagtga
ttctaaacacaaaaccaactattaaccaaatataaaacttacctactataataatttaaa
tgaaaaacttcgatataaacttaaatatttaactcttaataaccaattaataacactatt
aagatatatataaccttattaaaatccctatatcactaaaaacctaactttgaaactcaa
aaccacaaacaattccaaattcaatctctctactttatacttctaaattctaaatataac
cccctcctctctactccaacctctataactactactccctaccaaactttcccaccacaa
taaactcctaaccctctcaaagaaaaccacaaactcaaatccttctaacaattaccttaa
ttatatcttatcccaacaacaaaaaaaaaacgaatacaccaataataccatcaacttaaa
aaataaaaacaaaacgattaaaaattcaaattcaccctaaactacacaataaattcaaaa
ccactctaaactacttataaccctatctcaaaaacaaataaacaaataaaaaaaaaaaaa
tcctccccctccaatcaacaacctaaatcaaccattcatttttatatctacttaaaccaa
tcaaactttcattcaaatatttaatataaactaacattatataaatgacaatattccaaa
ccaataaccaaaccctatattcactaaaccaacctactttccactataataaaaatccca
tccttattactcacccactcccaaatcttcaatactttttcatctttttctaaatcctac
acaaaatccccggggccatggcggccgggagcatgcgacgtcgggccccattcggcctat
agggagtcgtattacaattcactggccggcgttttacaacgtcgtgactggggaaaaccc
gggggttacccaacttaaatcgccttgcaggcacatcccccttttcgcccggctggggta
ataagcgaaaaaggcccgccccggatggccctttcccaaacagttggggc

>ID|7092458 Atxnt-7_SP6_JM288_089.ab1
aacggggggtttgggggagctctcccatatgggtcgacctgcaggcggccgcactagtga
ttctaaacacaaaaccaactattaaccaaatataaaacttacctactataataatttaaa
taaaaaacttcgatataaacttaaatatttaactcttaataaccaattaataacactatt
aagatatatataaccttattaaaatccctatatcactaaaaacctaactttgaaactcaa
aaccacaaacaattccaaattcaatctctctactttatacttctaaattctaaatataac
cccctcctctctactccaacctctataactactactccctaccaaactttcccaccacaa
taaactcctaaccctctcaaataaaaccacaaactcaaatccttctaacaattaccttaa
ttatatcttatcccaacaacaaaaaaaaaacgaatacaccaataataccatcaacttaaa
aaataaaaacaaaacgattaaaaattcaaattcaccctaaactacacaataaattcaaaa
ccactctaaactacttataaccctatctcaaaaacaaataaacaaataaaaaaaaaaaaa
tcctccccctccaatcaacaacctaaatcaaccattcatttttatatctacttaaaccaa
tcaaactttcattcaaatatttaatataaactaacattatataaatgacaatattccaaa
ccaataaccaaaccctatattcactaaaccaacctactttccactataataaaaatccca
tccttattactcacccactcccaaatcttcaatactttttcatctttttctaaatcctac
acaaatcccgcggccatggcggccgggagcatgcgacgtcgggcccaattcgccctatag
tgagtcgtattacaattcacggggcgtcgtttttacaacggcggggcggggaaaacccgg
gcgttacccaacttaatccgcctggcagcaaatcccccttttgccagctggggggaaaag
cgaagaaggcccgcaccgat


>ID|7092467 Atxnt-2_SP6_JM288_042.ab1
tgggcttccaaggcgtttggggagctctcccatatgggtcgacctgcaggcggccgcact
agtgattctaaacacaaaaccaactattaaccaaatataaaacttacctactataataat
ttaaataaaaaacttcgatataaacttaaatatttaactcttaataaccaattaataaca
ctattaagatatatataaccttattaaaatccctatatcactaaaaacctaactttgaaa
ctcaaaaccacaaacaattccaaattcaatctctctactttatacttctaaattctaaat
ataaccccctcctctctactccaacctctataactactactccctaccaaactttcccac
cacaataaactcctaaccctctcaaataaaaccacaaactcaaatccttctaacaattac
cttaattatatcttatcccaacaacaaaaaaaaacgaatacaccaataataccatcaact
taaaaaataaaaacaaaacgattaaaaattcaaattcaccctaaactacacaataaattc
aaaaccactctaaactacttataaccctatctcaaaaacaaataaacaaataaaaaaaaa
aaaatcctccccctccaatcaacaacctaaatcaaccattcatttttatatctacttaaa
ccaatcaaactttcattcaaatatttaatataaactaacattatataaatgacaatattc
caaaccaataaccaaaccctatattcactaaaccaacctactttccactataataaaaat
cccatccttattactcacccactcccaaatcttcaatactttttcatctttttctaaaat
cctacacaaaaacccgcggccagggcggccgggagcatgcgaagtcggggcccaatcgcc
ctatagtgaggcgtattacaattcactgggccggcgttttacaacggcgtgactgggaaa
accctgggcggtacccaacttaaaccgccttgcaacacatcccccttttgcccggtgggg
gaaaaaagcgaaaaagggcccgcaccggattgcccctttcccaaccatttggcgcacccc
ggaaaggggggaaaggggac

>ID|7092468 Atxnt-8_SP6_JM288_090.ab1
gggggggtgggggggagcttctcccatatgggtcgacctgcaggcggccgcactagtgat
tctaaacacaaaaccaactattaaccaaatataaaacttacctactataataatttaaat
aaaaaacttcgatataaacttaaatatttaactcttaataaccaattaataacactatta
agatatatacaaccttattaaaatccctatatcactaaaaacctaactttgaaactcaaa
accacaaacaattccaaattcaatctctctactttatacttctaaattctaaatataacc
ccctcctctctactccaacctctataactactactccctaccaaactttcccaccacaat
aaactcctaaccctctcaaataaaaccacaaactcaaatccttctaacaattaccttaat
tatatcttatcccaacaacaaaaaaaaaacgaatacaccaataataccatcaacttaaaa
aataaaaacaaaacgattaaaaattcaaattcaccctaaactacacaataaattcaaaac
cactctaaactacttataaccctatctcaaaaacaaataaacaaataaaaaaaaaaaatc
ctccccctccaatcaacagcctaaatcaaccattcatttttatatctacttaaaccaatc
aaactttcattcaaatatttaatataaactaacattatataaatgacaatattccaaacc
aataaccaaaccctatattcactaaaccaacctactttccactataataaaaatcccatc
cttattactcacccactcccaaatcttcaatactttttcatctttttctaaatcctacac
aaaatcccgcggccatggcggccgggagcatgcgacgtcgggcccaattcgccctatagg
gggtcgtattacaatttcactggccgccgttttacaacggtcgggactgggaaaaccctg
gcgttaccccaatttaatcgccttgcaagaacaccccctttttcgccgc

>ID|7092469 Atxnt-9_SP6_JM288_007.ab1
atgggcttccaacgccgtttggggagctctcccatatgggtcgacctgcaggcggccgca
ctagtgattctaaacacaaaaccaactattaaccaaatataaaacttacctactataata
atttaaataaaaaacttcgatataaacttgaatatttaactcttaataaccaattaataa
cactattaagatatatataaccttattaaaatccctatatcactaaaaacctaactttga
aactcaaaaccacaaacaattccaaattcaatctctctactttatacttctaaattctaa
atataaccccctcccctctactccaacctctataactactactccctaccaaactttccc
accacaataaactcctaaccctctcaaataaaaccacaaactcaaatccttctaacaatt
accttaattatatcttatcccaacaacaaaaaaaaaacgaatacaccaataataccatca
acttaaaaaataaaaacaaaacgattaaaaattcaaattcaccctaaactacacaataaa
ttcaaaaccactctaaactacttataaccctatctcaaaaacaaataaacaaataaaaaa
aaaaaatcctccccctccaatcaacaacctaaatcaaccattcatttttatatctactta
aaccaatcaaactttcattcaaatatttaatataaactaacattatataaatgacaatat
tccaaaccaataaccaaaccctatattcactaaaccaacctactttccactataataaaa
atcccatccttattactcacccactcccaaatcttcaatactttttcatctttttctaaa
tcctacacaaaatcccgcggccatgggcggccgggagcatgcgacgtcgggccccattcg
ccctatagtgagtcgtatttacaattccattggccgtcgttttacaacgtcctgactggg
aaaaaccctggcgttacccaactttaatccgcctttggacaaatcccccctttcgccagc
tggggtaatagccgaaaaaggccccgccccga


>ID|7092481 Atxnt-11_SP6_JM288_023.ab1
attcaacgggcggttgtggagctctcccatatgggtcgacctgcaggcggccgcactagt
gattctaaacacaaaaccaactattaaccaaatataaaacttacctactataataattta
aataaaaaacttcgatataaacttaaatatttaactcttaataaccaattaataacacta
ttaagatatatataaccttattaaaatccctatatcactaaaaacctaactttgaaactc
aaaaccacaaacaattccaaattcaatctctctactttatacttctaaattctaaatata
accccctcctctctactccaacctctataactactactccctaccaaactttcccaccac
attaaactcctaaccctctcaaataaaaccacaaactcaaatccttctaacaattacttt
aattatttctaatcccaccaacaaaaaaaaacgaatacaccaataataccatcaacttaa
aaaataaaaacaaaacgattaaaaattcaaattcaccctaaactacacaataaattcaaa
accactctaaactacttataaccctatctcaaaaacaaataaacaaataaaaaaaaaaaa
atcctccccctccaatcaacaacctaaatcaaccattcatttttatatctacttaaacca
atcaaactttcattcaaatatttaatataaactaacattatataaatgacaatattccaa
accaataaccaaaccctatattcactaaaccaacctactttccactataataaaaatccc
atccttattactcacccactcccaaatcttcaatactttttcatctttttctaaatccta
cacaaaatcccgcggccatggcggccgggagcatgcgacgtcgggcccaattcgccctat
agggagtcgtattacaattcactgggcgtcgttttacaacgtcgtgactgggaaaacccc
tggcgttccccaacttaaatcgccttgcagcacaatccccctttcgccagcggggggaaa
taacgaaaaaggcccgcaccgatggccccttccccaacggttggccgcagcccggaaggg
gggaaagggaaggcgccccgg

>ID|7092482 Atxnt-12_SP6_JM288_024.ab1
gggaatccaacgcggtttggggagctctcccatatgggtcgacctgcaggcggccgcact
agtgattctaaacaacaaaaccaactattaaccaaatataaaacttacctactataatga
tttaaataaaaaacttcgatataaacttaaatatttaactcttaataaccaattaataac
actattaaaatatatataaccttattaaaatccctatatcactaaaaacctaactttaaa
actcaaaaccacaaacaattccagattcaatctctctactttatacttctaaattctaaa
tataaccccctcctctctactccaacctctataactactactccctaccaaactttccca
ccacaataaactcctaaccctctcaaataaaaccacaaactcaaatccttctaacaatta
ccttaattatatcttatcccaacaacaaaaaaaaaacaaatacaccaataataccatcaa
cttaaaaaataaaaacaaaacgattaaaaattcaaattcaccctaaactacacaataaat
tcaaaaccactctaaactacttataaccctatctcaaaaacaaataaacaaataaaaaaa
aaaaaaatcctccccctccaatcaacaacctaaatcaaccattcatttttatatctactt
aaaccaatcaaactttcattcaaatatttaatatgaactaacattatataaataacaata
ttccaaaccaataaccaaaccctatattcactaaaccaacctactttccactataataaa
aatcccatccttattactcacccactcccaaatcttcaatactttttcatctttttctaa
atcctacacaaaatccccgcggccatggcggccgggagcatgcgacgtcggggccaattc
gccctatagggagtcgaattacaattcacggggcgtcggttttcaacggcggtgaccggg
gaaacccctgggggttacccaacttaattcgccttggcggcacatccccccttttcgcca
gctgggggaaataaggaaagaaggcccggcaccgattggccctttcccaacaagttggcg
ccagcccggaagggg

>ID|7092483 Atxnt-14_SP6_JM288_040.ab1
agggattcccacggggtttggggagctctcccatatgggtcgacctgcaggcggccgcac
tagtgattctaaacacaaaaccaactattaaccaaaccctatattcactaaaccaaccta
ctttccactataataaaaatcccatccttattactcacccactcccaaatcttcaatact
ttttcatctttttctaaatcctacacaaaatcccgcggccatggcggccgggagcatgcg
acgtcgggcccaattcgccctatagtgagtcgtattacaattcactggccgtcgttttac
aacgtcgtgactgggaaaaccctggcgttacccaacttaatcgccttgcagcacatcccc
ctttcgccagctggcgtaatagcgaagaggcccgcaccgatcgcccttcccaacagttgc
gcagcctgaatggcgaatggacgcgccctgtagcggcgcattaagcgcggcgggtgtggt
ggttacgcgcagcgtgaccgctacacttgccagcgccctagcgcccgctcctttcgcttt
cttcccttcctttctcgccacgttcgccggctttccccgtcaagctctaaatcgggggct
ccctttagggttccgatttagtgctttacggcacctcgaccccaaaaaacttgattaggg
tgatggttcacgtagtgggccatcgccctgatagacggtttttcgccctttgacgttgga
gtccacgttctttaatagtggactcttgttccaaactggaacaacactcaaccctatctc
ggtctattcttttgatttataagggattttgccgatttcggcctattggttaaaaaatga
gctgatttaacaaaaatttaacgcgaattttaacaaaatattaacgcttaacatttcctg
atgcggtattttctccttacgcaatctgtgcggaatttcacaccgcatcaggggggactt
tttcgggaaatgggggccgaaacccctatttgtttatttttctaaataccattcaatatt
gtatccgcttcatggaaaaaaaaacccctgaataaatggcttcaataaatatgaaaaaaa
ggaaaaagtaatgattttt

>ID|7092484 Atxnt-16_SP6_JM288_056.ab1
gggccatccacggcgtttggggagctctcccatatgggtcgacctgcaggcggccgcact
agtgattctaaacacaaaaccaactattaaccaaatataaaacttacctactataatgat
ttaaataaaaaacttcgatataaacttaaatatttaactcttaataaccaattaataaca
ctattaaagtatatataaccttattaaaatccctatatcactaaaaacctaactttaaaa
ctcaaaaccacaaacaattccagattcaatctctctactttatacttctaaattctaaat
ataaccccctcctctctactccaacctctataactactactccctaccaaactttcccac
cacaataaactcctaaccctctcaaataaaaccacaaactcaaatccttctaacaattac
cttaattatatcttatcccaacaacaaaaaaaaaacaaatacaccaataataccatcaac
ttaaaaaataaaaacaaaacgattaaaaattcaaattcaccctaaactacacaataaatt
caaaaccactctaaactacttataaccctatctcaaaaacaaataaacaaataaaaaaaa
aaaaatcctccccctccaatcaacaacctaaatcaaccattcatttttatatctacttaa
accaatcaaactttcattcaaatatttaatatgaactaacattatataaataacaatatt
ccaaaccaataaccaaaccctatattcactaaaccaacctactttccactataataaaaa
tcccatccttattactcacccactcccaaatcttcaatactttttcatctttttctaaat
cctacacaaaatcccgcggccatggcggcgggggagcatgcgacgtcggggccaattcgc
cctatagtgaggcggattacaattcactggccgtcgttttacaacgtcgtggacggggaa
aaccctggcgttacccaatttaatcgcccttgaggcaaatcccccctttcggccggctgg
gcgaaaaagcgaaaaaggccccgcacccgattggcccctttcccaacaggtggg

>ID|7092485 Atxnt-18_SP6_JM288_072.ab1
tggggctcccacggcggttggggagctctcccatatgggtcgacctgcaggcggccgcac
tagtgattctaaacacaaaaccaactattaaccaaatataaaacttacctactataataa
tttaaataaaaaacttcgatataaacttaaatatttaactcttaataaccaattaataac
actattaagatatatataaccttattaaaatccctatatcactaaaaacctaactttgaa
actcaaaaccacaaacaattccaaattcaatctctctactttatacttctaaattctaaa
tataaccccctcctctctactccaacctctataactactactccctaccaaactttccca
ccacaataaactcctaaccctctcaaataaaaccacaaactcaaatccttctaacaatta
ccttaattatatcttatcccaacaacaaaaaaaaaacgaatacaccaataataccatcaa
cttaaaaaataaaaacaaaacgattaaaaattcaaattcaccctaaactacacaataaat
tcaaaaccactctaaactacttataaccctatctcaaaaacaaataaacaaataaaaaaa
aaaaatcctccccctccaatcaacaacctaaatcaaccattcatttttatatctacttaa
accaatcaaactttcattcaaatatttaatataaactaacattatataaatgacaatatt
ccaaaccaataaccaaaccctatattcactaaaccaacctactttccactataataaaaa
tcccatccttattactcacccactcccaaatcttcaatactttttcatctttttctaaat
cctacacaaatcccgcgggccatggcggccgggagcatgcgacgtcggggcccattcgcc
ctatagtgagtcgtattacaattcactggccgtcgttttacaacgtcgtgacggggaaaa
ccctgggcgttacccaacttaatcgccttgcagcacatccccctttcgcccgcgggggga
aaaagcgaaaagggcccgcaccgattg

>ID|7092486 Atxnt-1_SP6_JM288_041.ab1
gggccgggtggggggaggctctcccataatggtcgacctggcaggcggccgcactagtga
ttctaaacacaaaaccaactattaaccaaatataaaacttacctactataatgatttaaa
taaaaaacttcgatataaacttaaatatttaactcttaataaccaattaataacactatt
aaaatatatataaccttattaaaatccctatatcactaaaaacctaactttaaaactcaa
aaccacaaacaattccagattcaatctctctactttatacttctaaattctaaatataac
cccctcctctctactccaacctctataactactactccctaccaaactttcccaccacaa
taaactcctaaccctctcaaataaaaccacaaactcaaatccttctaacaattaccttaa
ttatatcttatcccaacaacaaaaaaaaacaaatacaccaataataccatcaacttaaaa
aataaaaacaaaacgattaaaaattcaaattcaccctaaactacacaataaattcaaaac
cactctaaactacttataaccctatctcaaaaacaaataaacaaataaaaaaaaaaaatc
ctccccctccaatcaacaacctaaatcaaccattcatttttatatctacttaaaccaatc
aaaccttcattcaaatatttaatacgaactaacattatataaataacaatattccaaacc
aatagccaaaccctatattcactaaaaccaacctactttccactataataaaaatcccat
ccttattactcacccactcccaaatcttcaatactttttcatctttttctaaatcctaca
caaaaatcccgcgggcatgggggccgggagcatgcgacgttcgggcccattcgcccctat
agtggggtcgtattacaatttcactgggcggtcgttttacaccgtcgggactggggaaaa
cccctggcggttccccaacttaaatcgccctggcagcaacatcccccctttccgccggct
ggggggtaaatagcgaaaaaggcccggcccccgattggccccttccccaacaggttgggc
gcgagcccga
