# Supplementary material for: Epigenetic interplay between mouse endogenous retroviruses and host genes
Source: Genome Biol. 2012 Oct 3;13(10):R89. doi: 10.1186/gb-2012-13-10-r89 (PMC3491417; doi:10.1186/gb-2012-13-10-r89)
Supplement: Additional file 4 — All bisulfite sequencing data. Compilation of all bisulfite sequences. [file gb-2012-13-10-r89-S4.zip › IAP10279_TE_oppositeLTR_thymus.rtf]

NonPolymorphic IAP Chr7-3' LTR B6 Thymus

>3LTR7Thy_72
TGGTTTATTTGTTTTTATTAAAAGAAAAAGGGGGAGATGTTGGGAGTCGTTTTTATATTC
GTCGTTATAAGATGGCGTTGATAGTTGTGTTTTAAGTGGTAAATAAATAATTTGCGTATG
TGTTAAGGGTATTTTATGATTATTTGTGTTTTGTTTTTTTCGTGACGTTAATTCGGTCGA
TGGGTTGTAGTTAATTAAGGAGTGATACGTTCGAGGCGAAGGAGAATGTTTTTTAAGAGG
GACGGGGTTTTCGTTTTTTTTTTTTTTTGTTTTTTGTTTTTTTGTTTTTTGTTTTTTTGT
TTTTTGTTTTTTTTTTTGAAGATGTAAGAATAAAGTTTTGTCGTAGAAGATTTTGGTTTG
TGGTGTTTTTTTTGGTCGGTCGTGAGAACGCGTTTAATAATATTTATGTTGTATTAAATA
TTGTTTTATAAATAAAATAATTTTATTTATGGTATTTATTTTTTTATTATTTAAAGGAAA
TATTATATTATAGAGATAGAAGTTAGAGAATAATTGGGGGAATTAGTTTTTTTTTTTATT
GTACGGATTTTTGGTTTTGAATTTAAGTTATTAGGATTGAT
>3LTRChr7_65
TGGTTTATTTGTTTTTATTAAAAGAAAAAGGGGGAGATGTTGGGAGTCGTTTTTATATTT
GTCGTTATAAGATGGCGTTGATAGTTGTGTTTTAAGTGGTAAATAAATAATTTGCGTATG
TGTTAAGGGTATTTTATGATTATTTGTGTTTTGTTTTTTTCGTGACGTTAATTCGGTCGA
TGGGTTGTAGTTAATTAAGGAGTGATACGTTCGAGGCGAAGGAGAATGTTTTTTAAGAGG
GACGGGGTTTTCGTTTTTTTTTTTTTTGTTTTTTGCTTTTTTGTTTTTTGTTTTTTTGTT
TTTTGTTTTTTTTTTTTGAAGATGTAAGAATAAAGTTTTGTCGTAGAAGATTTTGGTTTG
TGGTGTTTTTTTTGGTCGGTCGTGAGAACGCGTTTAATAATATTTATGTTGTATTAAATA
TTGTTTTATAAATAAAATAATTTTATTTATGGTATTTATTTTTTTATTATTTAAAGGAAA
TATTATATTATAGAGATAGAAGTTAGAGAATAATTGGGGGAATTAGTTTTTTTTTTTTAT
TGTACGGATTTTTGGTTTTGAATTTAAGTTATTAGGATTGAT
>3LTRChr7_6
TGGTTTATTTGTTTTTATTAAAAGAAAAAGGGGGAGATGTTGGGAGTCGTTTTTATATTC
GTCGTTATAAGATGGCGTTGATAGTTGTGTTTTAAGTGGTAAATAAATAATTTGCGTATG
TGTTAAGGGTATTTTATGATTATTTGTGTTTTGTTTTTTTCGTGACGTTAATTCGGTCGA
TGGGTTGTAGTTAATTAAGGAGTGATACGTTCGAGGCGAAGGAGAATGTTTTTTAAGAGG
GACGGGGTTTTCGTTTTTTTTTTTTTTTGTTTTTTGTTTTTTTGTTTTTTGTTTTTTTGT
TTTTTGTTTTTTTTTTTTGAAGATGTAAGAATAAAGTTTTGTCGTAGAAGATTTTGGTTT
GTGGTGTTTTTTTTGGTCGGTCGTGAGAACGCGTTTAATAATATTTATGTTGTATTAAAT
ATTGTTTTATAAATAAAATAATTTTATTTATGGTATTTATTTCTTTATTATTTAAAGGAA
ATATTATATTATAGAGATAGAAGTTAGAGAATAATTGGGGGAATTAGTTTTTTTTTATTG
TACGGATTTTTGGTTTTGAATTTAAGTTATTAGGATTGAT
>3LTRChr7_7
TGGTTTATTTGTTTTTATTAAAAGAAAAAGGGGGAGATGTTGGGAGTCGTTTTTATATTC
GTCGTTATAAGATGGCGTTGATAGTTGTGTTCTAAGTGGTAAATAAATAATTTGCGTATG
TGTCAAGGGTATTTTATGATTATTTGTGTTTTGTTTTTTTCGTGACGTTAATTCGGTCGA
TGGGTTGTAGTTAATTAAGGAGTGATACGTTCGAGGCGAAGGAGAATGTTTTTTAAGAGG
GACGGGGTTTTCGTTTTTTTTTTTTTTTGTTTTTTGTTTTTTTGTTTTTTGTTTTTTTGT
TTTTTGTTTTTTTTTTTGAAGATGTAAGAATAAAGTTTTGTCGTAGAAGATTTTGGTTTG
TGGTGTTTTTTTTGGTCGGTCGTGAGAACGCGTTTAATAATATTTATGTTGTATTAAATA
TTGTTTTATAAGTAAAATAATTTTATTTATGGTATTTATTTTTTTATTATTTAAAGGAAA
TATTATATTATAGAGATAGAAGTTAGAGAATAATTGGGGGAATTAGTTTTTTTTTTTTAT
TGTACGGATTTTTGGTTTTGAATTTAAGTTATTAGGA
>3LTRChr7_8
TGGTTTATTTGTTTTTATTAAAAGAAAAAGGGGGAGATGTTGGGAGTCGTTTTCATATTC
GTCGTTATAAGATGGCGTTGATAGTTGTGTTTTAAGTGGTAAATAAATAATTTGCGTATG
TGTTAAGGGTATTTTATGATTATTTGTGTTTTGTTTTTTTCGTGACGTTAATTCGGTCGA
TGGGTTGTAGTTAATTAAGGAGTAATACGTTCGAGGCGAAGGAGAATGTTTTTTAAGAGG
GACGGGGTTTTCGTTTTTTTTTTTTTGTTTTTTGTTTTTTTGTTTTTTGTTTTTTTGTTT
TTTGTTTTTTTTTTTGAAGATGTAAGAATAAAGTTTTGTCGTAGAAGATTTTGGTTTGTG
GTGTTTTTTTTGGTCGGTCGTGAGAACGCGTTTAATAATATTTATGTTGTATTAAATATT
GTTTTATAAATAAAATAATTTTATTTATGGTATTTATTTTTTTATTATTTAAAGGAAATA
TTATATTATAGAAATAGAAGTTAGAGAATAATTGGGGGAATTAGTTTTTTCTTTTTATTG
TACGGATTTTTGGTTTTGAATTTAAGTTATTAGGATTGAT
>3LTRChr7_9
TGGTTTATTTGTTTTTATTAAAAGAAAAAGGGGGAGATGTTGGGAGTCGTTTTTATATTC
GTCGTTATAAGATGGCGTTGATAGTTGTGTTTTAAGTGGTAAATAAATAATTTGCGTATG
TGTTAAGGGTATTTTATGATTGTTTGTGTTTTGTTTTTTTCGTGACGTTAATTCGGTCGA
TGGGTTGTAGTTAATTAAGGAGTGATACGTTCGAGGCGAAGGAGAATGTTTTTTAAGAGG
GACGGGGTTTTTGTTTTTTTTTTTTTTGTTTTTTGTTTTTTTGTTTTTTGTCTTTTTGTT
TTTTGTTTTTTTTTTTGAAGATGTAAGAATAAAGTTTTGTCGTAGAAGATTTTGGTTTGT
GGTGTTTTTTTGGTCGGTCGTGAGAACGCGTTTAATAATATTTATGTTGTATTAAATATT
GTTTTATAAATAAAATAATTTTATTTATGGTATTTATTTTTTTATTATTTAAAGGAAATA
TTATATTATAGAGATAGAAGTTAGAGAATAATTGGGGGAATTAGTTTTTTTTTTTTATTG
TACGGATTTTTGGTTTTGAATTTAAGTTATTAGGATTGAT
>3LTRChr7_11
TGGTTTATTTGTTTTTATTAAAAGAAAAAGGGGGAGATGTTGGGAGTCGTTTTTATATTC
GTCGTTATAAGATGGCGTTGATAGTTGTGTTTTAAGTGGTAAATAAATAATTTGTGTATG
TGTTAAGGGTATTTTATGATTATTTGTGTTTTGTTTTTTTTGTGACGTTAATTCGGTCGA
TGGGTTGTAGTTAATTAAGGAGTGATACGTTCGAGGCGAAGGAGAATGTTTTTTAAGAGG
GACGGGGTTTTTGTTTTTTTTTTCTTTTGTTTTTTGTTTTTTTGTTTTTTGTTTTTTTGT
TTTTTGTTCTTTTTTTTTGAAGATGTAAGAATAAAGTTTTGTCGTAGAAGATTTTGGTTT
GTGGTGTTTTTTTTGGTCGGTCGTGAGAACGCGTTTAATAATATTTATGTTGTATTAAAT
ATTGTTTTATAAATAAAATAATTTTATTTATGGTATTTATTTTTTTAGTATTTAAAGGAA
GTATTGTATTATAGAGATAGAAGTTAGAGAATAATTGGGGGAATCAGTTTTTTTTTTTTA
TTGTACGGATTTTTGGTTTTGAATTTAAGTTATTAGGATTGAT
>3LTRChr7_16
TGGTTTATTTGTTTTTATTAAAAGAAAAAGGGGGAGATGTTGGGAGTCGTTTTTATATTC
GTCGTTATAAGATGGCGTTGATAGTTGTGTTTTAAGTGGTAAATAAATAATTTGCGTATG
TGTTAAGGGTATTTTATGATTATTTGTGTTTTGTTTTTTTCGTGACGTTAATTCGGTCGA
TGGGTTGTAGTTAATTAAGGAGTGATACGTTCGAGGCGAAGGAGAATGTTTTTTAAGAGG
GACGGGGTTTTCGTTTTTTTTTTTTTGTTTTTTGTTTTTTTGTTTTTTGTTTTTTTGTTT
TTTGTTTTTTTTTTTTGAAGATGTAAGAATAAAGTTTTGTCGTAGAAGATTTTGGTTTGT
GGTGTTTTTTTTGGTCGGCCGTGAGAACGCGTTTAATAATATTTATGTTGTATTAAATAT
TGTTTTATAAATAAAATAATTTTATTTATGGTATTTATTTTTTTATTATTTAAAGGAAAT
ATTATATTATAGAGATAGAAGTTAGAGAATAATTGGGGGAATTAGTTTTTTTTTTATTGT
ACGGATTTTTGGTTTTGAATTTAAGTTATTAGGATTGAT
>3LTRChr7_19
TGGTTTATTTGTTTTTATTAAAAGAAAAAGGGGGAGATGTTGGGAGTCGTTTTTGTATTC
GTCGTTATAAGATGGCGTTGATAGTTGTGTTTTAAGTGGTAAATAAATGATTTGCGTATG
TGTTAAGGGTATTTTATGGTTATTTGTGTTTTGTTTTTTTCGTGACGTCAATTCGGTCGA
TGGGTTGTAGTTAATTAAGGAGTGATACGTTCGAGGCGAAGGAGAATGTTTTTTAAGAGG
GACGGGGTTTTCGTTTTTTTTTTTTTTTGTTTTTTGTTTTTTTGTTTTTTGTTTTTTTGT
TTTTTGTTTTTTTTTTTTGAAGATGTAAGAATAAAGTTTTGTCGTAGAAGATTTTGGTTT
GTGGTGTTTTTTTTGGTCGGTCGTGAGAACGCGTTTAATAATATTTATGTTGTATTAAAT
ATTGTTTTATAAATAAAATAATTTTATTTATGGTATTTATTTTTTTATTATTTAAAGGAA
ATATTATATTATAGAGATAGAAGTTAGAGAATAATTGGGGGAATTAGTTTTTTTTTTTTA
TTGTACGGATTTTTGGTTTTGAATTTAAGTTATTAGGATTGAT
>3LTRChr7_21
TGGTTTATTTGTTTTTATTAAAAGAAAAAGGGGGAGATGTTGGGAGTCGTTTTCATATTC
GTCGTTATAAGATGGCGTTGATAGTTGTGTTTTAAGTGGTAAATAAATAATTTGCGTATG
TGTTAAGGGTATTTTATGATTATTTGTGTTTTGTTTTTTTCGTGACGTTAATTCGGTCGA
TGGGTTGTAGTTAATTAAGGAGTGATACGTTCGAGGCGAAGGAGAATGTTTTTTAAGAGG
GACGGGGTTTTCGTTTTTTTTTTTTTTTGTTTTTTGTTTTTTTGTTTTTTGTTTTTTTGT
TTTTTGTTTTTTTTTTTTGAAGATGTAAGAATAAAGTTTTGTCGTAGAAGATTTTGGTTT
GTGGTGTTTTTTTTGGTCGGTCGTGAGAACGCGTTTAATAATATTTATGTTGTATTAAAT
ATTGTTTTATAAATAAAATAATTTTATTTATGGTATTTATTTTTTTATTATTTAAAGGAA
ATATTATATTATAGAGATAGAAGTTAGAGAATAATTGGGGGAATTAGTTTTTTTTTTTTA
TTGTACGGATTTTTGGTTTTGAATTTAAGTTATTAGGATTGAT
