# Supplementary material for: Epigenetic interplay between mouse endogenous retroviruses and host genes
Source: Genome Biol. 2012 Oct 3;13(10):R89. doi: 10.1186/gb-2012-13-10-r89 (PMC3491417; doi:10.1186/gb-2012-13-10-r89)
Supplement: Additional file 4 — All bisulfite sequencing data. Compilation of all bisulfite sequences. [file gb-2012-13-10-r89-S4.zip › IAP13596_TE_ES.rtf]

5/28/10
Chr1. 3' LTR-B6 ES gDNA
>Chr1ES_5
AATTGAATTTTTTTTTTTTGTATTTGGGGTTTTGTTTTAGTTTGGGTTTGTATTGTATTG
AAGGGATATTACGATTAAGGTAATATTTAATTGGGGTTGGTTTATAATTTTAGAGGTTTA
TTATTATTATGGTAAGTATAGTAGTATTTAGGTAGATAATGGTATTGGAGAAGAAGTTGA
GGGTTTTATATTTTGTTTTAAAGGTAGTTAGGAGAAGATTGTTTTTTAAGTAGTTAGGAG
GAGGGTTTTAAAGTTTATTTTTGTTATTAGACGCGTTTTTACGATCGGTTAGGAAGAATA
TTATAGATTAGAATTTTTTGCGGTAAAGTTTTATTTTTATATTTTTAGGAAAAGAGAGTA
AGAAGTAAGAGAGAGAGAAAACGAAAATTTCGTTTTTTTTAAGGAGTATTTTTTTTCGTT
TCGGACGTGTTATTTTTTGATTGGTTGTAGTTTATCGGTCGAGTTGACGTTACGGGGAAG
GTAGAGTATAAGTAGTTATAAGATATTTTTGGTATATGTGTAGATTATTTGTTTATTATT
TAGAATATAGGATGTTAGCGTTATTTTGTAACGGCGAATGTGGGGGCGGTTTTTAATATT
TTTTTTTTTTTTTTTTAATAAGAGTAAATAGGTTATTTATATTAATGAGAGTGGAGA
>Chr1ES_6
AATTGAATTTTTTTTTTTTTGTATTTGGGGTTTTGTTTTAGTTTGGGTTTGTATTGTATT
GAAGGGATATTACGATTAAGGTAATATTTAATTGGGGTTGGTTTATAATTTTAGAGGTTT
ATTATTATTATGGTAAGTATAGTAGTATTTAGGTAGATAATGGTATTGGAGAAGAAGTTG
AGGGTTTTATATTTTGTTTTAAAGGTAGTTAGGAGAAGATTGTTTTTTAAGTAGTTAGGA
GGAGGGTTTTAAAGTTTATTTTTGTTATTAGACGCGTTTTTATGATCGGTTAGGAAGAAT
ATTATAGATTAGAATTTTTTGCGGTAAAGTTTTATTTTTACATTTTTAGGAAAAGAGAGT
AAGAAGTAAGAGAGAGAGAAAACGAAAATTTCGTTTTTTTTAAGGAGTATTTTTTTTCGC
CTCGGACGTGTCATTTTTTGATTGGTTGCAGTCCATCGGCCGAGTTGACGTTACGGGGAA
GGTAGAGTATAAGTAGTTATAAGATATTTTTGGTATATGCGTAGATTATTTGTTTATCAT
TTAGAACACAGGATGTCAGCGTCATTTTGTAACGGCGAATGTGGGGGCGGCTTTTAACAT
TTTCTTTTTTTTTTTTTAATAAGAGTAAATAGGTTATTTATATTAATGAGAGTGGAGA
>Chr1ES_7
ATTGAATTTTTTTTTTTTGTATTTGGGGTTTTGTTTTAGTTTGGGTTTGTATTGTATNGA
AGGGATATTACGATTAAGGTAATATTTAATTGGGGTTGGTTTATAATTTTAGAGGTTTAT
TATTATTATGGTAAGTATAGTAGTATTTAGGTAGATAATGGTATTGGAGAAGAAGTTGAG
GGTTTTATATTTTGTTTTAAAGGTAGTTAGGAGAAAATTGTTTTTTAAGTAGTTAGGAGG
AGGGTTTTAAAGTTTATTTTTGTTATTAGACNCGTTTTTACGATCGGTTAGGAAGAATAT
TATAGATTAGAATTTTTTGCGGTAAAGTTTTATTTTTATATTTTTAGGAAAAGAGAGTAA
GAAGTAAGAGAGAGAGAAAACGAAAATTTCGTTTTTTTTAAGGAGTATTTTTTTTCGTTT
CGGACGTGTTATTTTTTGATTGGTTGTAGTTTATCGGTCGAGTTGACGTTACGGGGAAGG
TAGAGTATAAGTAGTTATAAGATATTTTTGGTATATGCGTAGATTATTTGTTTATTATTT
AGAATATAGGATGTTAGCGTTATTTTGTAACGGCGAATGTGGGGGCGGTTTTTAATATTT
TTTTTTTTTTTTTAATAAGAGTAAATAGGTTATTTATATTAATGAGAGTGGAGATACGAG
GTTAAA
>Chr1ES_8
ATTGAATTTTTTTTTTTTTGTATTTGGGGTTTTGTTTTAGTTTGGGTTTGTATTGTATTG
AAGGGATATTACGATTAAGGTAATATTTAATTGGGGTTGGTTTATAATTTTAGAGGTTTA
TTATTATTATGGTAAGTATAGTAGTATTTAGGTAGATAATGGTATTGGAGAAGAAGTTGA
GGGTTTTATATTTTGTTTTAAAGGTAGTCAGGAGAAGATTGTTTTTTAAGTAGTTAGGAG
GAGGGTTTTAAAGTTTATTTTTGTTATTAGACGCGTTTTACGATTGGTTAGGAAGAATAT
TATAGATTAGAATTTTTTGCGGTAAAGTTTTATTTTTATATTTTTAGGAAAAGAGAGTAA
GAAGTAAGAGAGAGAGAAAACGAAAATTTTGTTTTTTTTAAGGAGTATTTTTTTTCGTTT
CGGACGTGTTATTTTTTGATTGGTTGTAGTTTATCGGTCGAGTTGACGTTACGGGGAAGG
TAGAGTATAAGTAGTTATAAGATATTTTTGGTATATGCGTAGATTATTTGTTTATTATTT
AGAATATAGGATGTTAGCGTTATTTTGTAACGGCGAATGTGGGGGCGGTTTTTAATATTT
TTTTTTTTTTTTTTTAATAAGAGTAAATAGGTTATTTATATTAATGAGAGTGGAGA
>Chr1ES_9
AATTGAATTTTTTTTTTTTGTATTTGGGGTTTTGTTTTAGTTTGGGTTTGTATTGTATTG
AAGGGGTATTACGATTAAGGTAATATTTAATTGGGGTTGGTTTATAATTTTAGAGGTTTA
TTATTATTATGGTAAGTATAGTAGTATTTAGGTAGATAATGGTATTGGAGAAGAAGTTGA
GGGTTTTATATTTTGTTTTAAAGGTAGTCAGGAGAAGATTGTTTTTTAAGTAGTTAGGAG
GAGGGTTTTAAAGTTTATTTTTGTTATTAGACGCGTTTTACGATTGGTTAGGAAGAATAT
TATAGATTAGAATTTTTTGCNGTAAAGTTTTATTTTTATATTTTTAGGAAAAGAGAGTAA
GAAGTAAGAGAGAGAGAAAACGAAAATTTTGTTTTTTTTAAGGAGTATTTTTTTTCGTTT
CGGACGTGTTATTTTTTGATTGGTTGTAGTTTATCGGTCGAGTTGACGTTACGGGGAAGG
TAGAGTATAAGTAGTTATAAGATATTTTTGGTATATGCGTAGATTATTTGTTTATTATTT
AGAATATAGGATGTTAGCGTTATTTTGTAACGGCGAATGTGGGGGCGGTTTTTAATATTT
TTTTTTTTTTTTTTTAATAAGAGTAAATAGGTTATTTATATTAATGAGAGTGGAGA
>Chr1ES_3
AATTGAATTTTTTTTTTTTGTATTTGGGGTTTTGTTTTAGTTTGGGTTTGTATTGTATTG
AAGGGATATTACGATTAAGGTAATATTTAATTGGGGTTGGTTTATAATTTTAGAGGTTTA
TTATTATTATGGTAAGTATAGTAGTATTTAGGTAGATAATGGTATTGGAGAAGAAGTTGA
GGGTTTTATATTTTGTTTTAAAGGTAGTTAGGAGAAGATTGTTTTTTAAGTAGTTAGGAG
GAGGGTTTTAAAGTTTATTTTTGTTATTAGACGCGTTTTTACGATCGGTTAGGAAGAATA
TTATAGATTAGAATTTTTTGCGGTAAAGTTTTATTTTTATATTTTTAGGAAAAGAGAGTA
AGAAGTAAGAGAGAGAGAAAACGAAAATTTCGTTTTTTTTAAGGAGTATTTTTTTTCGTT
TCGGATGTGTTATTTTTTGATTGGTTGTAGTTTATCGGTCGAGTTGACGTTACGGGGAAG
GTAGAGTATAAGTAGTTATAAGATATTTTTGGTATATGCGTAGATTATTTGTTTATTATT
TAGAATATAGGATGTTAGCGTTATTTTGTAACGGCGAATGTGGGGGCGGTTTTTAATATC
TTTTTTTTTTTTTTTAATAAGAGTAAATAGGTTATTCATATTAATGAGAGTGGAGAT
