# Supplementary material for: Epigenetic interplay between mouse endogenous retroviruses and host genes
Source: Genome Biol. 2012 Oct 3;13(10):R89. doi: 10.1186/gb-2012-13-10-r89 (PMC3491417; doi:10.1186/gb-2012-13-10-r89)
Supplement: Additional file 4 — All bisulfite sequencing data. Compilation of all bisulfite sequences. [file gb-2012-13-10-r89-S4.zip › IAPrr1_TE_oppositeLTR_thymus.rtf]

Liane's Case Chromosome 11-5' LTR
B6 Thymus-Miniprep Sequences
>5LTRChr11_26
GTAGTTGAATTTTTTTTAATAGTTTGTGTTACGGGAATTTTATAATTTTGATTCGTAGTT
TTGGTTTTGGAATGAAGTATTTTTTTTGCGTTAGTTCGGAGTTTTTTTTCGTTTCGGATT
TTTTCGTTTCGGAATTCGGTATTAATTGTTATTAGACGCGTTTTTACGATCGGTTAGGAA
GAATATTATAGATTAGAATTTTTTGCGGTAAAGTTTTATTTTTATATTTTTAGGAAAAGA
GAGTAAGAAGTAAGAGAGAGAGAAAACGAAAATTTCGTTTTTTTTAAGGAGTATTTTTTT
TCGTTTTGGACGTATTATTTTTTGATTGGTTGTAGTTTATCGGTCGAGTTGACGTTACGG
GGAAGGTAGAGTATAAGTAGTTATAAGATATTTTTGGTATATGCGTAGATTATTTGTTTA
TTATTTAGAATATAGGATGTTAGTGTTATTTTGTAACGGCGAATGTGGGGGCGGTTTTTA
ATATTGGGGTATTAGTGTATTTTATAGGTTCGTTTTTAGTTTTTTATATAGTTTTTATAA
TGTTGGATTTTTGTGTTAATTTTGTTGATAATAGAGTTGAGTTTTTAAATTTGGAGGTTT
G
>5LTRChr11_27
GTAGTTGAATTTTTTTTAATAGTTTGTGTTACGGGAATTTTATAATTTTGATTCGTAGTT
TTGGTTTTGGAGTGAAGTATTTTTTTTGCGTTAGTTCGGAGTTTTTTTTCGTTTCGGATC
TTTTCGTTTCGGAATTCGGTATTAATTGTTATTAGACGCGTTTTTACGATCGGTTAGGAA
GAATATTATAGATTAGAATTTTTTGCGGTAAAGTTTTATTTTTATATTTTTAGGAAAAGA
GAGTAAGAAGTAAGAGAGAGAGAAAACGAAAATTTCGTTTTTTTTAAGGAGTATTTTTTT
TCGTTTCGGACGTATTATTTTTTGATTGGTTGTAGTTTATCGGTCGAGTCGACGTTACGG
GGAAGGCAGAGTATAAGTAGTTATAAGATATTTTTGGTATATGCGTAGATTATTTGTTTA
TTATTTAGAATATAGGATGTTAGTGTTATTTTGTAACGGCGAATGTGGGGGCGGTTTTTA
ATATTGGGGTATTAGTGTATTTTATAGGTTCGTTTTTAGTTTTTTATATAGTTTTTATAA
TGTTGGATTTTTGTGTTAATTTTGTTGATAATAGAGTTGAGTTTTTAAATTTGGAGGTTT
G
>5LTRChr11_29
GTAGTTGAATTTTTTTTAATAGTTTGTGTTACGGGAATTTTATAATTTTGATTCGTAGTT
TTGGTTTTGGAATGAAGTATTTTTTTTGCGTTAGTTCGGAGTTTTTTTTCGTTTCGGATT
TTTTCGTTTCGGAATTCGGTATTAATTGTTATTAGACGCGTTTTTACGATCGGTTAGGAA
GAATATTATAGATTAGAATTTTTTGCGGTAAAGTTTTATTTTTATATTTTTAGGAAAAGA
GAGTAAGAAGTAAGAGAGAGAGAAAACGAAAATTTCGTTTTTTTTAAGGAGTATTTTTTT
TCGTTTCGGACGTATTATTTTTTGATTGGTTGTAGTTTATCGGTCGAGTTGACGTTACGG
GGAAGGTAGAGTATAAGTAGTTATAAGATATTTTTGGTATATGCGTAGATTATTTGTTTA
TTATTTAGAATATAGGATGTTAGTGTTATTTTGTAACGGCGAATGTGGGGGCGGTTTTTA
ATATTGGGGTATTAGTGTATTTTATAGGTTCGTTTTTAGTTTTTTATATAGTTTTTATAA
TGTTGGATTTTTGTGTTAATTTTGTTGATAATAGAGTTGAGTTTTTAAATTTGGAGGTT
>5LTRChr11_30
GTAGTTGAATTTTTTTTAATAGTTTGTGTTACGGGAATTTTATAATTTTGATTCGTAGTT
TTGGTTTTGGAATGAAGTATTTTTTTTGCGTTAGTTCGGAGTTTTTTTTCGTTTCGGATT
TTTTCGTTTCGGAATTCGGTATTAATTGTTATTAGACGCGTTTTTACGATCGGTTAGGAA
GAATATTATAGATTAGAATTTTTTGCGGTAAAGTTTTATTTTTATATTTTTAGGAAAAGA
GAGTAAGAAGTAAGAGAGAGAGAAAACGAAAATTTCGTTTTTTTTAAGGAGTATTTTTTT
TCGTTTCGGACGTATTATTTTTTGATTGGTTGTAGTTTATCGGTCGAGTTGACGTTACGG
GGAAGGTAGAGTATAAGTAGTTATAAGATATTTTTGGTATATGCGTAGATTATTTGTTTA
TTATTTAGAATATAGGATGTTAGCGTTATTTTGTAACGGCGAATGTGGGGGCGGTTTTTA
ATATTGGGGTATTAGTGTATTTTATAGGTTCGTTTTTAGTTTTTTATATAGTTTTTATAA
TGTTGGATTTTTGTGTTAATTTTGTTGATAATAGAGTTGAGTTTTTAAATTTGGAGGTTT
G
>5LTRChr11_31
GTAGTTGAATTTTTTTTAATAGTTTGTGTTACGGGAATTTTATAATTTTGATTCGTAGTT
TTGGTTTTGGAATGAAGTATTTTTTTTGTGTTAGTTCGGAGTTTTTTTTCGTTTCGGATT
TTTTCGTTTCGGAATTCGGTATTAATTGTTATTAGACGCGTTTTTACGATCGGTTAGGAA
GAATATTATAGATTAGAATTTTTTGCGGTAAAGTTTTATTTTTATATTTTTAGGAAAAGA
GAGTAAGAAGTAAGAGAGAGAGAAAACGAAAATTTCGTTTTTTTTAAGGAGTATTTTTTT
TCGTTTCGGACGTATTATTTTTTGATTGGTTGTAGTTTATCGGTCGAGTTGACGTTACGG
GGAAGGTAGAGTATAAGTAGTTATAAGATATTTTTGGTATATGCGTAGATTATTTGTTTA
TTACTTAGAATATAGGATGTTAGCGTTATTTTGTAACGGCGAATGTGGGGGCGGTTTTCA
ATATTGGGGTATTAGTGTATTTTATAGGTTCGTTTTTAGTTTTTTATATAGTTTTTATAA
TGTTGGATTTTTGTGTTAATTTTGTTGATAATAGAGTTGAGTTTTTAAATTTGGAGGTTT
G
>5LTRChr11_32
GTAGTTGAATTTTTTTTAATAGTTTGTGTTACGGGAATTTTATAATTTTGATTCGTAGTT
TTGGTTTTGGAATGAAGTATTTTTTTTGCGTTAGTTCGGAGTTTTTTTTCGTTTCGGATT
TTTTCGTTTCGGAATTCGGTATTAATTGTTATTAGACGCGTTTTTACGATCGGTTAGGAA
GAATATTATAGATTAGAATTTTTTGCGGTAAAGTTTTATTTTTATATTTTTAGGAAAAGA
GAGTAAGAAGTAAGAGAGAGAGAAAACGAAAATTTCGTTTTTTTTAAGGAGTATTTTTTT
TCGTTCCGGACGTATTATTTTTTGATTGGTTGTAGTTTATCGGTCGAGTTGACGTTACGG
GGAAGGTAGAGTATAAGTAGTTATAAGATATTTTTGGTATATGCGTAGATTATTTGTTTA
TTATTTAGAATATAGGATGTTAGCGTTATTTTGTAACGGCGAATGTGGGGGCGGTTTTTA
ATATTGGGGTATTAGTGTATTTTATAGGTTCGTTTTTAGTTTTTTATATAGTTTTTATAA
TGTTGGATTTTTGTGTTAATTTTGTTGATAATAGAGTTGAGTTTTTAAATTTGGAGGTTT
G
>5LTRChr11_33
GTAGTTGAATTTTTTTTAATAGTTTGTGTTACGGGAATTTTATAATTTTGATTCGTAGTT
TTGGTTTTGGAATGAAGTATTTTTTTTGCGTTAGTTCGGAGTTTTTTTTCGTTTCGGATT
TTTTCGTTTCGGAATTCGGTATTAATTGTTATTAGACGCGTTTTTACGATCGGTTAGGAA
GAATATTATAGATTAGAATTTTTTGCGGTAAAGTTTTATTTTTATATTTTTAGGAAAAGA
GAGTAAGAAGTAAGAGAGAGAGAAAACGAAAATTTCGTTTTTTTTAAGGAGTATTTTTTT
TCGTTTCGGACGTATTATTTTTTGATTGGTTGTAGTTTATCGGTCGAGTTGACGTTACGG
GGAAGGTAGAGTATAAGTAGTTATAAGATATTTTTGGTATATGCGTAGATTATTTGTTTA
TTATTTAGAATATAGGATGTTAGCGTTATTTTGTAACGGCGAATGTGGGGGCGGTTTTTA
ATATTGGGGTATTAGTGTATTTTATAGGTTTGTTTTTAGTTTTTTATATAGTTTTTATAA
TGTTGGATTTTTGTGTTAATTTTGTTGATAATAGAGTTGAGTTTTTAAATTTGGAGGTTT
G
>5LTRChr11_34
GTAGTTGAATTTTTTTTAATAGTTTGTGTTACGGGAATTTTATAATTTTGATTCGTAGTT
TTGGTTTTGGAATGAAGTATTTTTTTTGCGTTAGTTCGGAGTTTTTTTTCGTTTCGGATT
TTTTCGTTTCGGAATTCGGTATTAATTGTTATTAGACGCGTTTTTACGATCGGCCAGGAA
GAATATTATAGATTAGAATTTTTTGCGGTAAAGTTTTATTTTTATATTTTTAGGAAAAGA
GAGTAAGAAGTAAGAGAGAGAGAAAACGAAAATTTCGTTTTTTTTAAGGAGTATTTTTTT
TCGTTTCGGACGTATTATTTTTTGATTGGTTGTAGTTTATCGGTCGAGTTGACGTTACGG
GGAAGGTAGAGTATAAGTAGTTATAAGATATTTTTGGTATATGCGTAGATTATTTGTTTA
TTATTTAGAATATAGGATGTTAGCGTTATTTTGTAACGGCGAATGTGGGGGCGGTTTTTA
ATATTGGGGTATTAGTGTATTTTATAGGTTCGTCTTTAGTTTTTTATATAGTTTTTATAA
TGTTGGATTTTTGTGTTAATTTTGTTGATAATAGAGTTGAGTTTTTAAATTTGGAGGTTT
G
>5LTRChr11_35
GTAGTTGAATTTTTTTTAATAGTTTGTGTTACGGGAATTTTATAATTTTGATTCGTAGTT
TTGGTTTTGGAATGAAGTATTTTTTTTGCGTTAGTTCGGAGTTTTTTTTCGTTTCGGATT
TTTTCGTTTCGGAATTCGGTATTAATTGTTATTAGACGCGTTTTTACGATCGGTTAGGAA
GAATATTATAGATTAGAATTTTTTGCGGTAAAGTTTTATTTTTATATTTTTAGGAAAAGA
GAGTAAGAAGTAAGAGAGAGAGAAAACGAAAATTTCGTTTTTTTTAAGGAGTATTTTTTT
TCGTTTCGGACGTATTATTTTTTGATTGGTTGTAGTTTATCGGTCGAGTTGACGTTACGG
GGAAGGTAGAGTATAAGTAGTTATAAGATATTTTTGGTATATGCGTAGATTATTTGTTTA
TTATTTAGAATATAGGATGTTAGCGTTATTTTGTAACGGCGAATGTGGGGGCGGTTTTTA
ATATTGGGGTATTAGTGTATTTTATAGGTTCGTTTTTAGTTTTTTATATAGTTTTTATAA
TGTTGGATTTTTGTGTTAATTTTGTTGATAATAGAGTTGAGTTTTTAAATTTGGAGGTTT
G
>5LTRChr11_37
GTAGTTGAATTTTTTTTAATAGTTTGTGTTACGGGAATTTTATAATTTTGATTCGTAGTT
TTGGTTTTGGAATGAAGTATTTTTTTTGCGTTAGTTCGGAGTTTTTTTTCGTTTCGGATT
TTTTCGTTTCGGAATTCGGTATTAATTGTTATTAGACGCGTTTTTACGATCGGTTAGGAA
GAATATCATAGATTAGAATTTTTTGCGGTAAAGTTTTATTTTTATATTTTTAGGAAAAGA
GAGTAAGAAGTAAGAGAGAGAGAAAACGAAAATTTCGTTTTTTTTAAGGAGTATTTTTTT
TCGTTTCGGACGTATTACTTTTTGATTGGTTGTAGTTTATCGGTCGAGTTGACGTTACGG
GGAAGGTAGAGTATAAGTAGTTATAAGATATTTTTGGTATATGCGTAGATTATTTGTTTA
TTATTTAGAATATAGGATGTTAGCGTTATTTTGTAACGGCGAATGTGGGGGCGGTTTTTA
ATATTGGGGTATTAGTGTATTTTATAGGTTCGTTTTTAGTTTTTTATATAGTTTTTATAA
TGTTGGATTTTTGTGTTAATTTTGTTGATAATAGAGTTGAGTTTTTAAATTTGGAGGTTT
G
>5LTRChr11_38
GTAGTTGAATTTTTTTTAATAGTTTGTGTTACGGGAATTTTATAATTTTGATTCGTAGTT
TTGGTTTTGGAATGAAGTATTTTTTTTGCGTTAGTTTGGAGTTTTTTTTCGTTTCGGATT
TTTTCGTTTCGGAATTCGGTATTAATTGTTATTAGACGCGTTTTTACGATCGGTTAGGAA
GAATATTATAGATTAGAATTTTTTGCGGTAAAGTTTTATTTTTATATTTTTAGGAAAAGA
GAGTAAGAAGTAAGAGAGAGAGAAAACGAAAATTTCGTTTTTTTTAAGGAGTATTTTTTT
TCGTTTCGGACGTATTATTTTTTGATTGGTTGTAGTTTATCGGTCGAGTTGACGTTACGG
GGAAGGTAGAGTATAAGTAGTTATAAGATATTTTTGGTATATGCGTAGATTATTTGTTTA
TTATTTAGAATATAGGATGTTAGCGTCATTTTGTAACGGCGAATGTGGGGGCGGTTTTTA
ATATTGGGGTATTAGTGTATTTTATAGGTTCGTTTTTAGTTTTTTATATAGTTTTTATAA
TGTTGGATTTTTGTGTTAATTTTGTTGATAATAGAGTTGAGTTTTTAAATTTGGAGGTTT
G
