# Supplementary material for: Epigenetic interplay between mouse endogenous retroviruses and host genes
Source: Genome Biol. 2012 Oct 3;13(10):R89. doi: 10.1186/gb-2012-13-10-r89 (PMC3491417; doi:10.1186/gb-2012-13-10-r89)
Supplement: Additional file 4 — All bisulfite sequencing data. Compilation of all bisulfite sequences. [file gb-2012-13-10-r89-S4.zip › IAPrr1_TE_thymus.docx]

Liane’s NonPolymorphic IAP Cases

Chr11 3’ LTR-B6 Thymus Miniprep Sequences

>3LTR11Thy_2

TTTGTTTTTTTTTTAGATATTTATTTTGTGTGGAGGTGATGGTATATGTGTGTGGTTGTA

TAATTGTTATAGTGGGTGTGGTTAGAGGATAAGTTGTAGTAGTTGGTGTTTTTTATTTAT

ATTGTGGGATTTTAGGGGTTAGATTTAGGTATTTAGGTTTGGGGTGTTATTAGACGCGTT

TTTATGATTGGTTAGGAAGAATATTATAGATTAGAATTTTTTGCGGTAAAGTTTTATTTT

TATATTTTTAGGAAAAGAGAGTAAGAAGTAAGAGAGAGAGAAAACGAAAATTTCGTTTTT

TTTAAGGAGTATTTTTTTTCGTTTCGGACGTATTATTTTTTGATTGGTTGTAGTTTATCG

GTCGAGTTGACGTTACGGGGAAGGTAGAGTATAAGTAGTTATAAGATATTTTTGGTATAT

GCGTAGATTATTTGTTTATTATTTAGAATATAGGATGTTAGCGTTATTTTGTAACGGCGA

ATGTGGGGGCGGTTTTTAATATTTTTTTTTTTTTTTTTTTAATAAGAGTAAATAGGTCAT

CTATATTAATGAGAGTGGAGATAGAGGTTAAATTTTTAGTGTGTAGGTAAAGGAGTTATG

TATAGGATTAGTTTTTAGGTTTATAGGTTTTTATTTAGAGTAA

>3LTR11Thy_3

TTTGTTTTTTTTTTAGATATTTATTTTGTGTGGAGGTGATGGTATATGTGTGTGGTTGTA

TAATTGTTATAGTGGGTGTGGTTAGAGGATAAGTTGTAGTAGTTGGTGTTTTTCATTTAT

ATTGTGGGATTTTAGGGGTTAGATTTAGGTATTTGGGTTTGGGGTGTTATTAGACGCGTT

TTTACGACCGGTTAGGAAGAATATTATAGATTAGAATTTTTTGCGGTAAAGTTTTATTTT

TATATTTTTAGGAAAAGAGAGTAAGAAGTAAGAGAGAGAGAAAACGAAAATTTCGTTTTT

TTTAAGGAGTATTTTTTTTCGTTTTGGACGTATTATTTTTTGATTGGTTGTAGTTTACCG

GTCGAGTTGACGTTACGGGGAAGGTAGAGTATAAGTAGTTATAAGATATTTTTGGTATAT

GCGTAGATTATTTGTTTATTATTTAGAATATAGGATGTTAGCGTTATTTTGTAACGGCGA

ATGTGGGGGCGGTTTTTAATATTTTTTTTTTTTTTTTTTAATAAGAGTAAATAGGTTATC

TATATTAATGAGAGTGGAGATAGAGGTTAAATTTTTAGTGTGTAGGTAAAGGAGTTATGT

ATAGGATTAGTTTTTAGGTTTATAGGTTTTTATTTAGAGTAA

>3LTR11Thy_6

TTTGTTTTTTTTTTAGATATTTATTTTGTGTGGAGGTGATGGTATATGTGTGTGGTTGTA

TAATTGTTATAGTGGGTGTGGTTAGAGGATAAGTTGTAGTAGTTGGTGTTTTTTATTTAT

ATTGTGGGATTTTAGGGGTTAGATTTAGGTATTTAGGTTTGGGGTGTTATTAGACGCGTT

TTTATGATTGGTTAGGAAGAATATTATAGATTAGAATTTTTTGCGGTAAAGTTTTATTTT

TATATTTTTAGGAAAAGAGAGTAAGAAGTAAGAGAGAGAGAAAACGAAAATTTCGTTTTT

TTTAAGGAGTATTTTTTTTCGTTTCGGACGTATTATTTTTTGATTGGTTGTAGTTTATCG

GTCGAGTTGACGTTACGGGGAAGGTAGAGTATAAGTAGTTATAAGATATTTTTGGTATAT

GCGTAGATTATTTGTTTATTATTTAGAATATAGGATGTTAGCGTTATTTTGTAACGGCGA

ATGTGGGGGCGGTTTTTAATATTTTTTTTTTTTTTTTTTTAATAAGAGTAAATAGGTCAT

CTATATTAATGAGAGTGGAGATAGAGGTTAAATTTTTAGTGTGTAGGTAAAGGAGTTATG

TATAGGATTAGTTTTTAGGTTTATAGGTTTTTATTTAGAGTAA

>3LTR11Thy_11

TTTGTTTTTTTTTTAGATATTTATTTTGTGTGGAGGTGATGGTATATGTGTGTGGTTGTA

TAATTGTCATAGTGGGTGTGGTTAGAGGATAAGTTGTAGCAGTTGGTGTTTTTCATTTAT

ATTGTGGGATTTTAGGGGTTAGATTTAGGTATTTAGGTTTGGGGTGTTATTAGACGTGTT

TTTACGATCGGTTAGGAAGAATATTATAGATTAGAATTTTTTGCGGTAAAGTTTTATTTT

TACATTTTTAGGAAAAGAGAGTAAGAAGTAAGAGAGAGAGAAAACGAAAATTTCGTCTTT

TTTAAGGAGTATTTTTTTTTCGTTTCGGACGTATTATTTCTTGATTGGTTGTAGTTTATC

GGCCGAGTTGACGTTACGGGGAAGGTAGAGTATAAGTAGTTATAAGATATTTTTGGTATA

TGCGTAGATTATTTGTTTATTATTTAGAATATAGGATGTTAGCGCTATCTTGTAACGGCG

AATGTGGGGGCGGTTTTTAACATTTTTTTTTTTTTTTTTAATAAGAGTAAATAGGTTATT

TATATTAATGAGAGTGGAGATAGAGGTTAAATTTTTAGTGTGTAGGTAAAGGAGTTATGT

ATAGGATTAGTTTTTAGGTTTATAGGTTTTTATTTAGAGTAA

>3LTR11Thy_16

TTTGTTTTTTTTTTAGATATTTATTTTGTGTGGAGGTGATGGTATATGTGTGTGGTTGTA

TAATTGTTATAGTGGGTGTGGTTAGAGGATAAGTTGTAGTAGTTGGTGTTTTTTATTTAT

ATTGTGGGATTTTAGGGGTTAGATTTAGGTATTTAGGTTTGGGGTGTTATTAGACGCGTT

TTTACGATCGGTTAGGAAGAATATTATAGATTAGAATTTTTTGCGGTAAAGTTTTATTTT

TATATTTTTAGGAAAAGAGAGTAAGAAGTAAGAGAGAGAGAAAACGAAAATTTCGTTTTT

TTTAAGGAGTATTTTTTTTCGTTTCGGACGTATTATTTTTTGATTGGTTGTAGTTTATCG

GTCGAGTTGACGTTACGGGGAAGGTAGAGTATAAGTAGTTATAAGATATTTTTGGTATAT

GCGTAGATTATTTGTTTATTATTTAGAATATAGGATGTTAGCGTTATTTTGTGACGGCGA

ATGTGGGGGCGGTTTTTAATATTTCTTTTTTTTTTTTTAATAAGAGTAAGTAGGTTATTT

ATATTAATGAGAGTGGAGATAGAGGTTAAATTTTTAGTGTGTAGGCAAAGGAGTTATGTA

TAGGATTAGTTTTTAGGTTTATAGGTTTTTATTTAGAGTAA

>3LTR11Thy_18

TTTGTTTTTTTTTTAGATATTTTATTTTGTGTGGAGGTGATGGTATATGTGTGTGGTTGT

ATAATTGTTATAGTGGGTGTGGTTAGAGGATAAGTTGTAGTAGTTGGTGTTTTTTATTTA

TATTGTGGGATTTTAGGGGTTAGATTTAGGTATTTAGGTTTGGGGTGTTATTAGACGCGT

TTTTACGATCGGTTAGGAAGAATATTATAGATTAGAATTTTTTGCGGTAAAGTTTTATTT

TTATATTTTTAGGAAAAGAGAGTAAGAAGTAAGAGAGAGAGAAAACGAAAATTTCGTTTT

TTTTAAGGAGTATTTTTTTTGTTTCGGACGTATCATTTTTTGATCGGTTGTAGTTTATCG

GTCGAGTTGACGTTACGGGGAAGGTAGAGTATAAGTAGTTATAAGATATTTTTGGTATAT

GCGTAGATTATTTGTTTATTATTTAGAATATAGGATGTTAGCGTTATCTTGTAACGGCGA

ATGTGGGGGCGGTTTTTAATATTTTTTTTTTTTTTTAATAAGAGTAAGTAGGTTATTTAT

ATTAATGAGAGTGGAGATAGAGGTTAAATTTTTAGTGTGTAGGTAAAGGAGTTATGTATA

GGATTAGTTTTTAGGTTTATAGGTTTTTATTTAGAGTAA

>3LTR11Thy_19

TTTGTTTTTTTTTTAGATATTTATTTTGTGTGGAGGTGATGGTATATGTGTGTGGTTGTA

TAATTGTTATAGTGGGTGTGGTTAGAGGATAAGTTGTAGTAGTTGGTGTTTTTTATTTAT

ATTGTGGGATTTTAGGGGTTAGATTTAGGTATTTAGGTTTGGGGTGTTATTAGACGCGTT

TTTACGATCGGTTAGGAAGAATATTATAGATTAGAATTTTTTGCGGTAAAGTTTTATTTT

TATATTTTTAGGAAAAGAGAGTAAGAAGTAAGAGAGAGAGAAAACGAAAATTTCGTTTTT

TTTAAGGAGTATTTTTTTTCGTTTCGGACGTATTATTTTTTGATTGGTTGTAGTTTATCG

GTCGAGTTGACGTTACGGGGAAGGTAGAGTATAAGTAGTTATAAGATATTTTTGGTATAT

GCGTAGATTATTTGTTTATTATTTAGAATATAGGATGTTAGCGTTATTTTGTAACGGCGA

ATGTGGGGGCGGTTTTTAATATTTTTTTTTTTTTTTTAATAAGAGTAAATAGGTTATTTA

TATTAATGAGAGTGGAGATAGAGGTTAAATTTTTAGTGTGTAGGTAAAGGAGTTATGTAT

AGGATTAGTTTTTAGGTTTATAGGTTTTTATTTAGAGTAA

>3LTR11Thy_26

TTTGTTTTTTTTTTAGATATTTATTTTGTGTGGAGGTGATGGTATATGTGTGTGGTTGTA

TAATTGTTATAGTGGGTGTGGTTAGAGGATAAGTTGTAGTAGTTGGTGTTTTTTATTTAT

ATTGTGGGATTTTAGGGGTTAGATTTAGGTATTTAGGTTTGGGGTGTTATTAGACGCGTT

TTTACGATCGGTTAGGAAGAATATTATAGATTAGAATTTTTTGCGGTAAAGTTTTATTTT

TATATTTTTAGGAAAAGAGAGTAAGAAGTAAGAGAGAGAGAAAACGAAAATTTCGTTTTT

TTTAAGGAGTATTTTTTTTCGTTTCGGACGTATTATTTTTTGATTGGTTGTAGTTTATCG

GTCGAGTTGATGTCACGGGGAAGGTAGAGTATAAGTAGTTATAAGATATTTTTGGTATAT

GCGTAGATTATTTGTTTATTATTTAGAATATAGGATGTTAGCGTTATTTTGTAACGGCGA

ATGTGGGGGCGGTTTTTAATATTTTTTTTTTTTTTTTTTAATAAGAGTAAATAGGTTATT

TATATTAATGAGAGTGGAGATAGAGGTTAAATTTTTAGTGTGTAGGTAAAGGAGTTATGT

ATAGGATTAGTTTTTAGGTTTATAGGTTTTTATTTAGAGTAA

>3LTR11Thy_27

TTTGTTTTTTTTTTAGATATTTATTTTGTGTGGAGGTGATGGTATATGTGTGTGGTTGTA

TAATTGTTATAGTGGGTGTGGTTAGAGGATAAGTTGTAGTAGTTGGTGTTTTCTATTTAT

ATTGTGGGATTTTAGGGGTTAGATTTAGGTATTTAGGTTTGGGGTGTTATTAGACGCGTT

TTTACGATCGGTTAGGAAGAATATTATAGATTAGAATTTTTTGCGGTAAAGTTTTATTTT

TATATTTTTAGGAAAAGAGAGTAAGAAGTAAGAGAGAGAGAGAACGAAAATTTCGTTTTT

TTTAAGGAGTATTTTTTTCGTTTCGGACGTATTATTCTTTGATTGGCTGTAGTTTATCGG

TCGAGTTGACGTTACGGGGAAGGTAGAGTATAAGTAGTTATAAGATATTTTTGGTATATG

CGTAGATTATTTGTTTATTATTTAGAATATAGGATGTTAGCGTTATTTTGTAGCGGCGAA

TGTGGGGGCGGTTTTCAATATTTTTTTTTTTTTTTTTTTAATAAGAGTAAATAGGTTATT

TATGTTAATGAGAGTGGAGATAGAGGTTAAATTTTTAGTGTGTAGGTAAAGGAGTTATGT

ATAGGATTAGTTTTTAGGTTTATAGGTTTTTATTTAGAGTAA

>3LTR11Thy_31

TTTGTTTTTTTTTTAGATATTTATTTTGTGTGGAGGTGATGGTATATGTGTGTGGTTGTA

TAATTGTTATAGTGGGTGTGGTTAGAGGATAAGTTGTAGTAGTTGGTGTTTTTTATTTAT

ATTGTGGGATTTTAGGGGTTAGATTTAGGTATTTAGGTTTGGGGTGTTATTAGACGCGTT

TTTACGATCGGTTAGGAAGAATATTATAGATTAGAATTTTTTGCGGTAAAGTTTTATTTT

TATATTTTTAGGAAAAGAGAGTAAGAAGTAAGAGAGAGAGAAAACGAAAATTTCGTTTTT

TTTAAGGAGTATTTTTTTTCGTTTCGGACGTATTATTTTTTGATTGGTTGTAGTTTATCG

GTCGAGTTGACGTTACGGGGAAGGTAGAGTATAAGTAGTTATAAGATATTTTTGGTATAT

GCGTAGATTATTTGTTTATTATTTAGAATATAGGATGTTAGCGTTATTTTGTAATGGCGA

ATGTGGGGGCGGTTTTTAATATTTTTTTTTTTTTTTTTAATAAGAGTAAATAGGTTATTT

ATATTAATGAGAGTGGAGATAGAGGTTAAATTTTTAGTGTGTAGGTAAAGGAGTTATGTA

TAGGATTAGTTCTTAGGTTTATAGGTTTTTATTTAGAGTAA

>3LTR11Thy_85

TTTGTTTTTTTTTTAGATATTTATTTTGTGTGGAGGTGATGGTATATGTGTGTGGTTGTA

TAATTGTTATAGTGGGTGTGGTTAGAGGATAAGTTGTAGTAGTTGGTGTCTTTTATTTAT

ATTGTGGGATTTTAGGGGTTAGATTTAGGTATTTAGGTTTGGGGTGTTATTAGACGCGTT

TTTACGATCGGTCAGGAAGAATATTATAGATTAGAATTTTTTGCGGTAAAGTTTTATTTT

TATATTTTTAGGAAAAGAGAGTAAGAAGTAAGAGGGAGAGAAAACGAAAATTTCGTTTTT

TTTAAGGAGTATTTTTCTTCGTTTCGGACGTATTATTTTTTGATTGGTTGTAGTTTATCG

GTCGAGTTGACGTTACGGGGAAGGTAGAGTATAAGTAGTTATAAGATATTTTTGGTATAT

GCGTAGATTATTTGTTTATTATTTAGAATATAGGATGTTAGCGTTATTTTGTAACGGCGA

ATGTGGGGGCGGTTTTTAATATTTTCTTTTTTTTTTTTTAATAAGAGTAAATAGGTTATT

TATATTAATGAGAGTGGAGATAGAGGTTAAATTTTTAGTGTGTAGGTAAAGGAGTTATGT

ATAGGATTAGTTTTTAGGTTTATAGGTTTTTATTTAGAGTAA

>3LTR11Thy_86

TTTGTTTTTTTTTTAGATATTTATTTTGTGTGGAGGTGATGGTATATGTGTGTGGTTGTA

TAATTGTTATAGTGGGTGTGGTTAGAGGATAAGTTGTAGTAGTTGGTGTTTTTTATTTAT

ATTGTGGGATTTTAGGGGTTAGATTTAGGTATTTAGGTTTGGGGTTTTATTAGACGCGTT

TTTACGATCGGTTAGGAAGAATATTATAGATTAGAATTTTTTGCGGTAAAGTTTTATTTT

TATATTTTTAGGAAAAGAGAGTAAGAAGTAAGAGAGAGAGAAAACGAAAATTTCGTTTTT

TTTAAGGAGTATTTTCTTTCGTTTCGGACGTATTATTTTTTGATTGGTTGTAGTTTATCG

GTCGAGTTGACGTTACGGGGAAGGTAGAGTATAAGTAGTTATAAGATATTTTTGGTATAT

GCGTAGATTATTTGTTTATTATTTAGAATATAGGATGTTAGCGTTATTTTGTAACGGCGA

ATGTGGGGGCGGTTTTTAATATTTTTTTTTTTTTTAATAAGAGTAAATAGGTTATTTATA

TTAATGAGAGTGGAGATAGAGGTTAAATTTTTAGTGTGTAGGTAAAGGAGTTATGTATAG

GATTAGTTTTTAGGTTTATAGGTTTTTATTTAGAGTAA

>3LTR11Thy_87

TTTGTTTTTTTTTTAGATATTTATTTTGTGTGGAGGTGATGGTATATGTGTGTGGTTGTA

TAATTGTTATAGTGGGTGTGGTTAGAGGATAAGTTGTAGTAGTTGGTGTTTTTTATTTAT

ATTGTGGGATTTTAGGGGTTAGATTTAGGTATTTAGGTTTGGGGTGTTATTAGACGCGTT

TTTACGATCGGTTAGGAAGAATATTATAGATTAGAATTTTTTGCGGTAAAGTTTTATTTT

TATATTTTTAGGAAAAGAGAGTAAGAAGTAAGAGAGAGAGAAAACGAAAATTTCGTTTTT

TTTAAGGAGTATTTTTTTCGTTTCGGACGTATTATTTTTTGATTGGTTGTAGTTTATCGG

TCGAGTTGACGTTACGGGGAAGGTAGAGTATAAGTAGTTATAAGATATTTTTGGTACATG

CGTAGATTATTTGTTTATTATTTAGAATATAGGATGTTAGCGTTATTTTGTAACGGCGAA

TGTGGGGGCGGTTTTTAATATTTTTTTTTTTTTTTTTTAATAAGAGTAAATAGGTTATTT

ATATTAATGAGAGTGGAGATAGAGGTTAAATTTTTAGTGTGTAGGTAAAGGAGTTATGTA

TAGGATTAGTTTTTAGGTTTATAGGTTTTTATTTAGAGTAA

>3LTR11Thy_88

TTTGTTTTTTTTTAGATATTTATTTTGTGTGGAGGTGATGGTATATGTGTGTGGTTGTAT

AATTGTTATAGTGGGTGTGGTTAGAGGATAAGTTGTAGTAGTTGGTGTTTTTTATTTATA

TTGTGGGATTTTAGGGGTTAGATTTAGGTATTTAGGTTTGGGGTGTTATTAGACGCGTTT

TTACGATCGGTTAGGAAGAATATTATAGATTAGAATTTTTTGCGGTAAAGTTTTATTTTT

ATATTTTTAGGAAAAGAGAGTAAGAAGTAAGAGAGAGAGAAAACGAAAATTTCGTTTTTT

TTAAGGAGTATTTTTTTTCGTTTCGGACGTATTATTTTTTGATTGGTTGTAGTTTATCGG

TCGAGTTGACGTTACGGGGAAGGTAGAGTATAAGTAGTTATAAGATATTTTTGGTATATG

CGTAGATTATTTGTTTATTATTTAGAATATAGGATGTTAGCGTTATTTTGTAACGGCGAA

TGTGGGGGCGGTTTTTAATATATTTTTTTTTTTTAATAAGAGTAAATAGGTTATTTATAT

TAATGAGAGTGGAGATAGAGGTTAAATTTTTAGTGTGTAGGTAAAGGAGTTATGTATAGG

ATTAGTTTTTAGGTTTATAGGTTTTTATTTAGAGTAA
