# Supplementary material for: Epigenetic interplay between mouse endogenous retroviruses and host genes
Source: Genome Biol. 2012 Oct 3;13(10):R89. doi: 10.1186/gb-2012-13-10-r89 (PMC3491417; doi:10.1186/gb-2012-13-10-r89)
Supplement: Additional file 4 — All bisulfite sequencing data. Compilation of all bisulfite sequences. [file gb-2012-13-10-r89-S4.zip › IAPti1072970530_TE_gene_brain_129allelefromB6129hybrids.rtf]

>129brain2
TTATTTTTTGATTGGTTGTAGTTTATCGGTCGAGTTGATGTTACGGGGAAGGTAGAGTAT
AAGTAGTTATAAGATATTTTTGGTATATGCGTAGATTATTTGTTTATTATTTAGAATATA
GGATGTTAGCGTTATTTTGTAACGGTGAATGTGGGGGCGGTTTTTAATATTTATTAAAGT
AGAATATCGGTGTTAATAATATTAAGAGTTGAATTATCGATTTTGTTTTTTATAAAAATT
GAAGATAGTTTATTAGTAGGGAAGAAAAAAAATGATTTTTTTTTTATTTTGAGGATAGTA
AGGGTGATTTATTGTTAGGGATGGGGAAAGAAGTTTTGGGAAGCGAAGGGTATGAGGGTA
GAGGATGTTAGAGGGTTAGAAAAGAAGCTTTGAGGTTAGATGTTTAGAGGAGGGTGTGGT
TTAGTTGGGTAGTAGGATTATTTATAATTGTGTTGGTTGTGAGTGTATATTGGGGTGTTT
TTGATTTTGGTTGTTAGAGGGTGTAGTTTGAGTTGGTGTGGTGTTGGGAAGGAGGAGGGG
AGTGTGAGAAGGGTTAGGTGTGTAGGGTGTTTGTGTAGTTTGGTTTTGGGAAGGGTGTGG
AGTTTTTGTTTTTGGGATATGGTTTTTTGTGTTGTTGGTGTTGTTCAGTTGTTTTGTGGG
TAAGTAGTGAGTGGTTTGGGTGGGTGTGGGGAGGGAGTTTTGGGGTTGTTGGTTATTTTT
GTGTGGTTTTGGTTGTTGTTTGTTTTGTTTTGTTTTGTTTTGGTTGTAGTGTTTTTTAGT
GGTGTTATTTTTGGTTTGTGTTTTTTTTATTTTTGTTTTTTTTTGTTGGGGTATGTGGAT
TGGTGGGGAGGGTGTGGTTGGGTTGGGAGTTAGAGTTGTGTAATTGTTAGAAATTTTATA
AGAGGT


>129brain9
GAAAAAAAACGATTTTTTTTTTTTTATTTTGAGGATAGTAAGGGTGATTTATTGTTAGGG
ATGGGGAAAGAAGTTTTGGGAAGTGAAGGGTATGAGGGTAGAGGATGTTAGAGGGTTAGA
AAAGAAGTTTTGAGGTTAGATGTTTAGAGGAGGGTGTGGTTTAGTTGGGTAGCAGGATTA
TTTATAATTGTGTTGGTTGTGAGTGTATATTGGGGCGTTTTTGATTTTGGTTGTTAGAGG
GTGTAGTTTGAGTTGGTGCGGTGTTGGGAAGGAGGAGGGGAGTGCGAGAAGGGTTAGGTG
CGTAGGGTGTTTGTGTAGCTTGGTTTTGGGAAGGGTGTGGAGTTTTTGTTTTTGGGATGT
GGTTTTTTGTGTTGTTGGTGTTGTTTAGTTGTTTTGTGGGTAAGTAGTGAGTGGTTTGGG
TGGGTGTGGGGAGGGAGTTTTGGGGTTGTTGGTTATTTTTGTGTGGTTTTGGTTGTTGTT
TGTTTTGTTTTGTTTTGTTTTGGTTGTAGTGTTTTTTAGTGGTGTTATTTTTGGTTTGTG
TTTTTTTTATTTTTGTTTTTTTTTGTTGGGGTATGTGGGTTGGTGGGGAGGGTGTGGTTG
GGTTGGGAGTTAGAGTTGTGTAATTGTTAGAAATTTTATAAGAGGT


>129brain10
GTATATGCGTAGATTATTTGTTTATTATTTAGAATATAGGATGTTAGCGTCATTTTGTAA
CGGCGAATGTGGGGGTGGTTTTTAATATTTATTAAAGTAGAATATCGGTGTTAATAATAT
TAAGAGTTGAATTATCGATTTTGTTTTTTATAAAAATTGAAGATAGTTTATTAGTAGGGA
AGAAAAAAAACGATTTTTTTTTTTTTATTTTGAGGATAGTAAGGGTGATTTATTGTTAGG
GATGGGGAAAGAAGTTTTGGGAAGCGAAGGGTATGAGGGTAGAGGATGTTAGAGGGTTAG
AAAAGAAGTTTTGAGGTTAGATGTTTAGAGGAGGGTGTGGTTTAGTTGGGTAGTAGGATT
ATTTATAATTGTGTTGGTTGTGAGTGTATATTGGGGTGTTTTTGATTTTGGTTGTTAGAG
GGTGTAGTTTGAGTTGGTGTGGTGTTGGGAAGGAGGAGGGGAGTGTGAGAAGGGTTAGGT
GTGTAGGGTGTTTGTGTAGTTTGGTTTTGGGAAGGGTGTGGAGTTTTTGTTTTTGGGATG
TGGTTTTTTGTGTTGTTGGTGTTGTTTAGTTGTTTTGTGGGTAAGTAGTGAGTGGTTTGG
GTGGGTGTGGGGAGGGAGTTTTGGGGTTGTTGGTTATTTTTGTGTGGTTTTGGTTGTTGT
TTGTTTTGTTTTGTTTTGTTTTGGTTGTAGTGTTTTTTAGTGGTGTTATTTTTGGTTTGT
GTTTTTTTTATTTTTGTTTTTTTTTGTTGGGGTATGTGGGTTGGTGGGGAGGGTGTGGTT
GGGTTGGGAGTTAGAGTTGTGTAATTGTTAGAAATTTTATAAGAGGT

>129brain10-1
TTATTTTTTGTTGGTTGTAGTTTATCGGTCGAGTTGATGTTATGGGGAAGGTAGAGTATA
AGTAGTTATAAGATATTTTTGGTATATGCGTAGATTAATTTGTTTATTATTTAGAATATA
GGGTGTTAGCGTTATTTTGTAATGGCGAATGTGGGGGCGGTTTTTAATATTTATTAAAGT
AGAATATCGGTGTTAATAATATTAAGAGTTGAATTATTGATTTTGTTTTTTATAAAAATT
GAAGATAGTTTATTAGTAGGGAAGAAAAAAAACGATTTTTTTTTTTTTATTTTGAGGATA
GTAAGGGTGATTTATTGTTAGGGATGGGGAAAGAAGTTTTGGGAAGTGAAGGGTATGAGG
GTAGAGGATGTTAGAGGGTTAGAAAAGAAGTTTTGAGGTTAGATGTTTAGAGGAGGGTGT
GGTTTAGTTGGGTAGTAGGATTATTTATAATTGTGTTGGTTGTGAGTGTATATTGGGGCG
TTTTTGATTTTGGTTGTTAGAGGGTGTAGTTTGAGTTGGTGCGGTGTTGGGAAGGAGGAG
GGGAGTGCGAGAAGGGTTAGGTGCGTAGGGTGTTTGTGTAGCTTGGTTTTGGGAAGGGTG
TGGAGTTTTTGTTTTTGGGATGTGGTTTTTTGTGTTGTTGGTGTTGTTTAGTTGTTTTGT
GGGTAAGTAGTGAGTGGTTTGGGTGGGTGTGGGGAGGGAGTTTTGGGGTTGTTGGTTATT
TTTGTGTGGTTTTGGTTGTTGTTTGTTTTGTTTTGTTTTGTTTTGGTTGTAGTGTTTTTT
AGTGGTGTTATTTTTGGTTTGTGTTTTTTTTATTTTTGTTTTTTTTTGTTGGGGTATGTG
GGTTGGTGGGGAGGGTGTGGTTGGGTTGGGAGTTAGAGTTGTGTAATTGTTAGAAATTTT
ATAAGAGG


>B3-129b1
AGTTTATCGGTCGAGTTGATGTTACGGGGAAGGTAGAGTATAAGTAGTTATAAGATATTT
TTGGTATATGCGTAGATTATTTGTTTATTATTTAGAATATAGGATGTTAGCGTTATTTTG
TAACGGCGAATGTGGGGGCGGTTTTTAATATTTATTAAAGTAGAATATCGGTGTTAATAA
TATTAAGAGTTGAATTATCGATTTTGTTTTTTATAAAAATTGAAGATAGTTTATTAGTAG
GGAAGAAAAAAACGATTTTTTTTTTTTATTTTGAGGATAGTAAGGGTGATTTATTGTTAG
GGATGGGGAAAGAAGTTTTGGGAAGTGAAGGGTATGAGGGTAGAGGATGTTAGAGGGTTA
GAAAAGAAGTTTTGAGGTTAGATGTTTAGAGGAGGGTGTGGTTTAGTTGGGTAGTAGGAT
TATTTATAATTGTGTTGGTTATGAGTGTATATTGGGGTGTTTTTGATTTTGGTTGTTAGA
GGGTGTAGTTTGAGTTGGTGTGGTGTTGGGAAGGAGGAGGGGAGTGTGAGAAGGGTTAGG
TGTGTAGGGTGTTTGTGTAGTTTGGTTTTGGGAAGGGTGTGGAGTTTTTGTTTTTGGGAT
GTGGTTTTTTGTGTTGTTGGTGTTGTTTAGTTGTTTTGTGGGTAAGTAGTGAGTGGTTTG
GGTGGGTGTGGGGAGGGAGTCTTGGGGTTGTTGGTTATTTTTGTGTGGTTTTGGTTGTTG
TTTGTTTTGTTTTGTTTTGTTTTGGTTGTAGTGTTTTTTAGTGGTGTTATTTTTGGTTTG
TGTTTTTTTTATTTTTGTTTTCTTTTGTTGGGGTATGTGGGTTGGTGGGGAGGGTGTGGT
CGGGTTGGGAGTTAGAGTTGTGTAATTGTTAGAAATTTTATAAGAGGT

>B3-129b2
TTATTTTTTGATTGGTTGTAGTTTATCGGTCGAGTTGATGTTACGGGGAAGGTAGAGTAT
AAGTAGTTATAAGATATTTTTGGTATATGCGTAGATTATTTGTTTACTATTTAGAATATA
GGATGTTAGCGTTATTTTGTAACGGTGAATGTGGGGGCGGTTTTTAATATTTATTAAAGT
AGAATATCGGTGTTAATAATATTAAGAGTTGAATTATCGATTTTGTTTTTTATAAAAATT
GAAGATAGTTTATTAGTAGGGAAGAAAAAGAATGATTTTTTTTTTTTTATTTTGAGGATA
GTAAGGGTGATTTATTGTTAGGGATGGGGAAAGAAGTTTTGGGAAGTGAAGGGTATGAGG
GTAGAGGATGTTAGAGGGTTAGAAAAGAAGTTTTGAGGTTAGATGTTTAGAGGAGGGTGT
GGTTTAGTTGGGTAGTAGGATTATTTATAATTGTGTTGGTTGTGAGTGTATACTGGGGTG
TTTTTGATTTTGGTTGTTAGAGGGTGTAGTTTGAGTCGGTGTGGTGTTGGGAAGGAGGAG
GGGAGTGTGAGAAGGGTTAGGTGTGTAGGGTGTTTGTGTAGTTTGGTTTTGGGAAGGGTG
TGGAGTTTTTGTTTTTGGGATGTGGTTTTTTGTGTTGTTGGTGTTGTTTAGTTGTTTTGT
GGGTAAGTAGTGAGTGGTTTGGGTGGGTGTGGGGAGGGAGTTTTGGGGTTGTTGGTTATT
TTTGTGTGGTTTTGGTTGTTGTTTGTTTTGTTTTGTTTTGTTTTGGTTGTAGTGTTTTTT
AGTGGCGTTATTTTTGGTTTGTGTTTTTTTTATTTTTGTTTTTTTTTGTTGGGGTATGTG
GGTTGGTGGGGAGGGTGTGGTTGGGTTGGGAGTTAGAGTTGTGTAATTGTTAGAAATTTT
ATAAGAGGT

>B3-129b5
TTATTTTTTGATTGGTTGTAGTTTATCGGTCGAGTTGATGTTACGGGGAAGGTAGAGTAT
AAGTAGTTATAAGATATTTTTGGTATATGCGTAGATTATTTGTTTACTATTTAGAATATA
GGATGTTAGCGTTATTTTGTAACGGTGAATGTGGGGGCGGTTTTTAATATTTATTAAAGT
AGAATATCGGTGTTAATAATATTAAGAGTTGAATTATTGATTTTGTTTTTTATAAAAATT
GAAGATAGTTTATTAGTAGGGAAGAAAAAAAACGATTTTTTTTTTTTTATTTTGAGGATA
GTAAGGGTGATTTATTGTTAGGGATGGGGAAAGAAGTTTTGGGAAGTGAAGGGTATGAGG
GCCAGAGGATGTTAGAGGGTTAGAAAAGAAGTTTTGAGGTTAGATGTTTAGAGGAGGGTG
TGGTTTAGTTGGGTAGTAGGATTATTTATAATTGTGTTGGTTGTGAGTGTATATTGGGGT
GTTTTTGATTTTGGTTGTTAGAGGGTGTAGTTTGAGTTGGTGTGGTGTTGGGAGGGAGGA
GGGGAGTGTGAGAAGGGTTAGGTGTGTAGGGTGTTTGTGTAGTTTGGTTTTGGGAAGGGT
GTGGAGTTTTTGTTTTTGGGATGTGGTTTTTTGTGTTGTTGGTGTTGTTTAGTTGTTTTG
TGGGTAAGTAGTGAGTGGTTTGGGTGGGTGTGGGGAGGGAGTTCTGGGGTTGTTGGTTAT
TTTTGTGTGGTTTTGGTTGTTGTTTGTTTTGTTTTGTTTTGTTTTGGTTGTAGTGTTTTT
TAGTGGTGTTATTTTTGGTTTGTGTTTTTTTTATTTTTGTTTTTTTTTGTTGGGGTATGT
GGGTTGGTGGGGAGGGTGTGGTTGGGTTGGGAGTTAGAGTTGTGTAATTGTTAGAAATTT
TATAAGAGGT

>B3-129b6
TTATTTTTTGATTGGTTGTAGTTTATCGGTCGAGTTGACGTTACGGGGAAGGTAGAGTAT
AAGTAGTTATAAGATATTTTTGGTATATGCGTAGATTATTTGTTTATTATTTAGAATATA
GGATGTTAGCGTTATTTTGTAACGGCGAATGTGGGGGCGGCTTTTAATATTTATTAAAGT
AGAATATCGGTGTTAATAATATTAAGAGTTGAATTATCGATTTTGTTTTTTATAAAAATT
GAAGATAGTTTATTAGCAGGGAAGAAAAAAAATGATTTTTTTTTTTTTATTTTGAGGATA
GTAAGGGTGATTTATTGTTAGGGATGGGGAAAGAAGTTTTGGGAAGTGAAGGGTATGAGG
GTAGAGGATGTTAGAGGGTTAGAAAAGAAGTTTTGAGGTTAGATGTTTAGAGGAGGGTGT
GGTTTAGTTGGGTAGTAGGATTATTTATAATTGTGTTGGTTGTGAGTGTATATTGGGGTG
TTTTTGATTTTGGTTGTTAGAGGGTGTAGTTTGAGTTGGTGTGGTGTTGGGAAGGAGGAG
GGGAGTGTGAGAAGGGTTAGGTGTGTAGGGTGTTCGTGTAGTTTGGTTTTGGGAAGGGTG
TGGAGTTTTTGTTTTTGGGATGTGGTTTTTTGTGTTGTTGGTGTTGTTTAGTTGTTTTGT
GGGTAAGTAGTGAGTGGTTTGGGTGGGTGTGGGGAGGGAGTCTTGGGGTTGTTGGTTATT
TTTGTGTGGTTTTGGTTGTTGTTTGTTTTGTTTTGTTTTGTTTTGGTTGTAGTGTTTTTT
AGTGGTGTTATTTTTGGCTTGTGTTTTTTTTATTTTTGTTTTTTTTTGTTGGGGTATGTG
GGTTGGTGGGGAGGGTGTGGTTGGGTTGGGAGTTAGAGTTGTGTAATTGTTAGAAATTTT
ATAAGAGGT

>B3-129b7
TTATTTTTTGATTGGTTGTAGTTTATCGGTCGAGTTGACGTTACGGGGAAGGTAGAGTAT
AAGTAGTTATAAAGATATTTTTGGTATATGCGTAGATTATTTGTTTATTATTTAGAATAT
AGGATGTTAGCGTTATTTTGTAACGGCGAATGTGGGGGCGGTTTTTAATATTTATTAAAG
TAGAATATCGGTGTTAATAATATTAAGAGTTGAATTATCGATTTTGTTTTTTATAAAAAT
TGAAGATAGTTTATTAGTAGGGAAGAAAAAAAATGATTTTTTTTTTTTATTTTGAGGATA
GTAAGGGTGATTTATTGTTAGGGATGGGGAAAGAAGTTTTGGGAAGTGAAGGGTATGAGG
GTAGAGGATGTTAGAGGGTTAGAAAAGAAGTTTTGAGGTTAGATGTTTAGAGGAGGGTGT
GGTTTAGTTGGGTAGTAGGATTATTTATAATTGTGTTGGTTGTGAGTGTATATTGGGGTG
TTTTTGATTTTGGTTGTTAGAGGGTGTAGTTTGAGTTGGTGTGGTGTTGGGAAGGAGGAG
GGGAGTGTGAGAAGGGTTAGGTGTGTAGGGTGTTTGTGTAGTTTGGTTTTGGGAAGGGTG
TGGAGTTTTTGTTTTTGGGATGTGGTTTTTTGTGTTGTTGGTGTTGTTTAGCTGTTTTGT
GGGTAAGTAGTGAGTGGTTTGGGTGGGTGTGGGGAGGGAGTTTTGGGGTTGTTGGTTATT
TTTGTGTGGTTTTGGTTGTTGTTTGTTTTGTTTTGTTTTGTTTTGGTTGTAGTGTTTTTT
AGTGGTGTTATTTTTGGTTTGTGTTTTTTTTATTTTTGTTTTTTTTTGTTGGGGTATGTG
GGTTGGTGGGGAGGGTGTGGTTGGGTTGGGAGTTAGAGTTGTGTAATTGTTAGAAATTTT
ATAAGAGGT
