# Supplementary material for: Epigenetic interplay between mouse endogenous retroviruses and host genes
Source: Genome Biol. 2012 Oct 3;13(10):R89. doi: 10.1186/gb-2012-13-10-r89 (PMC3491417; doi:10.1186/gb-2012-13-10-r89)
Supplement: Additional file 4 — All bisulfite sequencing data. Compilation of all bisulfite sequences. [file gb-2012-13-10-r89-S4.zip › IAPti1072970530_TE_gene_brain_B6allelefromB6129hybrids.rtf]

>B6brain11
GGGTTAAAGTTATTAATTAAATTTTATTTAAGAAGAGGATATTAAGTAAATTAGTTGAGT
TGATTAAAAATATTTTTATTAAAGTAGAATATCGGTGTCAATAATATTAAGAGTTGAATT
ATTGATTTTGTTTTTTATAAAAATTGAAGATAGTTTATTAGTAGGGAAGAAAAAAATGAT
TTTTTTTTTTTTATTTTGAGGATAGTAAGGGTGATTTATTGTTAGGGATGGGGAAAGAAGT
TTTGGGAAGTGAAGGGTATGATGGTAGAGTTTGTTAGAGGGTTAGAAAAGAAGTTTTGAG
GTTAGATGTTTAGAGGAGGGTGTGGTTTAGTTGGGTAGTAGGATTATTTATAATTGTGTT
GGTTGTGAGTGTATATTGGGGTGTTTTTGATTTTGGTTGTTAGAGGGTGTGGTTTGAGTT
GGTGTGGTGTTGGGAAGGAGGAGGGGAGTGTGAGAAGGGTTAGGTGTGTAGGGTGTTTGT
GTAGTTTGGTTTTGGGAAGGGTGTGGAGTTTTTGTTTTTGGGATGTGGTTTTTTGTGTTG
TTGGTGTTGTTTAGTTGTTTTGTGGGTAAGTAGTGAGTGGTTTGGGTGGGTGTGGGGAGG
GAGTTTTGGGGTTGTTGGTTATTTTTGTGTGGTTTTGGTTGTTGTTTGTTTTGTTTTGTT
TTGTTTTGGTTGTAGTGTTTTTTAGTGGTGTTATTTTTGGTTTGTGTTTTTTTTATTTTT
GTTTTTTTTGTTGGGGTATGTGGGTTGGTGGGGAGGGTGTGGTTGGGTTGGGAGTTAGAG
TTGTGTAATTGTTAGAAATTTTATAAGAGG

>B6brain1
GGGTTAAAGTTATTAATTAAATTTTATTTAAGAAGAGGATATTAAGTAAATTAGTTGAGT
TGATTAAAAATATCTTTATTAAAGTAGAATATCGGTGCTAATAATATTAAGAGTTGAATT
ATCGATTTTGTTTTTTATAAAAATTGAAGATAGTTTATTAGTAGGGAAGAAAAAAATGAT
TTTTTTTTTTTTATTTTGAGGATAGTAAGGGTGATTTATTGTTAGGGATGGGGAAAGAAG
TTTTGGGAAGTGAAGGGTATGATGGTAGAGTTTATTAGAGGGTTAGAAAAGAAGTTTTGA
GGTTAGATGTTTAGAGGAGGGTGTGGTTTAGTTGGGTAGTAGGATTATTTATAATTGTGT
TGGTTGTGAGTGTATATTGGGGTGTTTTTGATTTTGGTTGTTAGAGGGTGTAGTTTGAGT
TGGTGTGGTGTTGGGAAGGAGGAGGGGAGTGTGAGAAGGGTTAGGTGTGTAGGGTGTTTG
TGTAGTTTGGTTTTGGGAAGGGTGTGGAGTTTTTGTTTCTGGGATGTGGTTTTTTGTGTT
GTTGGTGTTGTTTAGTTGTTTTGTGGGTAAGTAGTGAGTGGTTTGGGTGGGTGTGGGGAG
GGAGTTTTGGGGTTGTTGGTTATTTTTGTGTGGTTTTGGTTGTTGTTTGTTTTGNTTTGT
TTTGTTTTGGTTGTAGTGTTTTTTAGTGGTGTTATTTTTGGTTTGTGTTTTTTTTATTTT
TGTTTTTTTTTGTTGGGGTATGTGGGTTGGTGGGGAGGGTGTGGTCGGGTTGGGAGTTAG
AGTTGTGTAATTGTTAGAAATTTTATAAGAGG

>B6brain3
GGGTTAAAGTTATTAATTAAATTTTATTTAAGAAGAGGATATTAAGTAAATTAGTTGAGT
TGATTAAAAATATTTTTATTAAAGTAGAATATTGGTGTTAATAATATTAAGAGTTGAATT
ATTGATTTTGTTTCTTTATAAAAATTGAAGATAGTTTATTAGTAGGGAAGAAAAAAATGA
TTTTTTTTTTTTTATTTTGAGGATAGTAAGGGTGATTTATTGTTAGGGATGGGGAAAGAAGTT
TTGGGAAGTGAAGGGTATGATGGTAGAGTTTGTTAGAGGGTTAGAAAAGAAGTTTTGAGG
TTAGATGTTTAGAGGAGGGTGTGGTTTAGTTGGGTAGTAGGATTATTTATAATTGTGTTG
GTTGTGAGTGTATATTGGGGTGTTTTTGATTTTGGTTGTTAGAGGGTGTAGTTTGAGTTG
GTGTGGTGTTGGGAAGGAGGAGGGGAGTGTGAGAAGGGTTAGGTGTGTAGGGTGTCTGTG
TAGTTTGGTTTTGGGAAGGGTGTGGAGTTTTTGTTTTTGGGATGTGGTTTTTTGTGTTGT
TGGTGTTGTTTAGTTGTTTTGTGGGTAAGTAGTGAGTGGTTTGGGTGGGTGTGGGGAGGG
AGTTTTGGGGTTGTTGGTTATTTTTGTGTGGTTTTGGTTGTTGTTTGTTTTGTTTTGTTT
TGTTTTGGTTGTAGTGTTTTTTAGTGGTGTTATTTTTGGTTTGTGTTTTTTTTATTTTTG
TTTTTTTTTGTTGGGGTATGTGGGTTGGTGGGGAGGGTGTGGTTGGGTTGGGAGTTAGAG
TTGTGTAATTGTTAGAAATTTTATAAGAGG

>B6brain4
ATTAGTAGGGAAGAAAAAAATGATTTTTTTTTTTCCATTTTGAGCACAGTAAGGGTGATT
TATTGTTAGGGATGGGGAAAGAAGTTTTGGGAAGTGAAGGGTATGATGGTAGAGTTTGTC
AGAGGGTTAGAAAAAAAGTTTTGAGGTTAGATGTTCAGAGGAGGGTGTGGTTTAGTTGGG
TAGTAGGATTATTTATAGTTGTGTTGGTTGTGAGTGTATATTGGGGTGTTTTTGATTTTG
GTTGTTAGAGGGTGTAGTTTGAGTTGGTGTGGTGTTGGGAAGGAGGAGGGGAGTGTGAGA
AGGGTTAGGTGTGTAGGGTGTTTGTGTAGTTTGGTTTCGGGAAGGGTGTGGAGTTTTTGT
TTTTGGGATGTGGTTTTTTGTGTTGTTGGTGTTGTTTAGTTGTTTTGTGGGTAAGTAGTG
AGTGGTTTGGGTGGGTGTGGGGAGGGAGTTTCGGGGTTGTTGGTTATTTTTGTGTGGTTT
TGGTTGTTGTTTGTTTTGTTTTGTTTTGTTTTGGTTGTAGTGTTTTTTAGTGGTGTCATT
TTTGGTTTGTGTTTTTTTTATTTTTGTTTTTTTTTGTTGGGGTATGTGGGTTGGTGGGGA
GGGTGTGGTTGGGTTGGGAGTCAGAGTTGTGTAATTGTTAGAAATTTTATAAGAGG


>B6brain5
TCGATTTTGTTTTTTATAAAAATTGAAGATAGTTTATTAGTAGGGAAGAAAAAAATGATT
TTTTTTTTTTTATTTTGAGGATAGTAAGGGTGATTTATTGTTAGGGATGGGGAAAGAAGTT
TTGGGAAGTGAAGGGTATGATGGTAGAGTTTGTTAGAGGGTTAGAAAAGAAGTTTTGAGG
TTAGATGTTTAGAGGAGGGTGTGGTTTAGTTGGGTAGTAGGATTATTTATAATTGTGTTG
GTTGTGAGTGTATATTGGGGTGTTTTTGATTTTGGTTGTTAGAGGGTGTAGTTTGAGTTG
GTGTGGTGTCGGGAAGGAGGAGGGGAGTGTGAGAAGGGTTAGGTGTGTAGGGTGTTTGTG
TAGTTTGGTTTTGGGAAGGGTGTGGAGTTTTTGTTTTTGGGATGTGGTTTTTTGTGTTGT
TGGTGTTGTTTAGTTGTTTTGTGGGTAAGTAGTGAGTGGTTTGGGTGGGTGTGGGGAGGG
AGTTTCGGGGTTGTTGGTTATTTTTGTGTGGTTTTGGTTGTTGTTTGTTTTGTTTTGTTT
TGTTTTGGTTGTAGTGTTTTTTAGTGGTGTTATTTTTGGTTTGTGTTTTTTTTATTTTTG
TTTTTTTTTGTTGGGGTATGTGGGTTGGTGGGGAGGGTGTGGTTGGGTTGGGAGTTAGAG
TTGTGCAATTGTTAGAAATTTTATAAGAGG

>B6brain6
GGGTTAAAGTTATTAATTAAATTTTATTTAAGAAGAGGATATTAAGTAAATTAGTTGAGT
TGATTAAAAATATTTTTATTAAAGTAGAACATCGGTGTTAATAATATTAAGAGTTGAATT
ATTGATTTTGTTTTTTATAAAAATTGAAGATAGTTTATTAGTAGGGAAGAAAAAAATGAT
TTTTTTTTTTTTATTTTGAGGACAGTAAGGGTGATTTATTGTTAGGGATGGGGAAAGAAG
TTTTGGGAAGTGAAGGGTATGATGGTAGAGTTTGTTAGAGGGTTAGAAAAGAAGTTTTGA
GGTTAGATGTTCAGAGGAGGGTGTGGTTTAGTTGGGTAGTAGGATTATTTATAGTTGTGT
TGGTTGTGAGTGTATATTGGGGTGTTTTTGATTTTGGTTGTTAGAGGGTGTAGTTTGAGT
TGGTGTGGTGTTGGGAAGGAGGAGGGGAGTGTGAGAAGGGTTAGGTGTGTAGGGTGTTTG
TGTAGTTTGGTTTCGGGAAGGGTGTGGAGTTTTTGTTTTTGGGATGTGGTTTTTTGTGTT
GTTGGTGTTGTTTAGTTGTTTTGTGGGTAAGTAGTGAGTGGTTTGGGTGGGTGTGGGGAG
GGAGTTTCGGGGTTGTTGGTTATTTTTGTGTGGTTTTGGTTGTTGTTTGTTTTGTTTTGT
TTTGTTTTGGTTGTAGTGTTTTTTAGTGGTGTCATTTTTGGTTTGTGTTTTTTTTATTTT
TGTTTTTTTTTGTTGGGGTATGTGGGTTGGTGGGGAGGGTGTGGTTGGGTTGGGAGTCAG
AGTTGTGTAATTGTTAGAAATTTTATAAGAGG

>B6brain7
GGTTAGAAAAGAAGTTTTGAGGTTAGATGTTCAGAGGAGGGTGTGGTTTAGTTGGGTAGT
AGGATTATTTATAGTTGTGTTGGTTGTGAGTGTATATTGGGGTGTTTTTGATTTTGGTTG
TTAGAGGGTGTAGTTTGAGTTGGTGTGGTGTTGGGAAGGAGGAGGGGAGTGTGAGAAGGG
TTAGGTGTGTAGGGTGTTTGTGTAGTTTGGTTTCGGGAAGGGTGTGGAGTTTTTGTTTTT
GGGATGTGGTTTTTTGTGTTGTTGGTGTTGTTTAGTTGTTTTGTGGGTAAGTAGTGAGTG
GTTTGGGTGGGTGTGGGGAGGGAGTTTCGGGGTTGTTGGTTATTTTTGTGTGGTTTTGGT
TGTTGTTTGTTTTGTTTTGTTTTGTTTTGGTTGTAGTGTTTTTTAGTGGTGTCATTTTTG
GTTTGTGTTTTTTTTATTTTTGTTTTTTTTGTTGGGGTATGTGGGTTGGTGGGGAGGGTG
TGGTCGGGTTGGGAGTTAGAGTTGTGTAATTGTTAGAAATTTTATAAGAGG

>B6brain10
GGGTTAAAGTTATTAATTAAATTTTATTTAAGAAGAGGATATTAAGTAAATTAGTTGAGT
TGATTAAAAATATCTTTATTAAAGTAGAATATCGGTGTTAATAATATTAAGAGTTGAATT
ATCGATTTTGTTTTTTATAAAAATTGAAGATAGTTTATTAGTAGGGAAGAAAAAAATGAT
TTTTTTTTTTTTATTTTGAGGATAGTAAGGGTGATTTATTGTTAGGGATGGGGAAAGAAGTT
TTGGGAAGTGAAGGGTATGATGGTAGAGTTTGTTAGAGGGTTAGAAAAGAAGTTTTGAGG
TTAGATGTTTAGAGGAGGGTGTGGTTTAGTTGGGTAGTAGGATTATTTATAATTGTGTTG
GTTGTGAGTGTATATTGGGGTGTTTTTGATTTTGGTTGTTAGAGGGTGTAGTTTGAGTTG
GTGTGGTGTTGGGAAGGAGGAGGGGAGTGTGAGAAGGGTTAGGTGTGTAGGGTGTTTGTG
TAGTTTGGTTTTGGGAAGGGTGTGGAGTTTTTGTTTTTGGGATGCGGTTTTTTGTGTTGT
TGGTGTTGTTTAGTTGTTTTGTGGGTAAGTAGTGAGTGGTTTGGGTGGGTGTGGGGAGGG
AGTTTTGGGGTTGTTGGTTATTTTTGTGTGGTTTTGGTTGTTGTTTGTTTTGTTTTGTTT
TGTTTTGGTTGTAGTGTTTTTTAGTGGTGTTATTTTTGGTTTGTGTTTTTTTTATTTTTG
TTTTTTTTTGTTGGGGTATGCGGGTTGGTGGGGAGGGTGTGGTTGGGTTGGGAGTTAGAG
TTGTGTAATTGTTAGAAATTTTATAAGAGG
