# Supplementary material for: Epigenetic interplay between mouse endogenous retroviruses and host genes
Source: Genome Biol. 2012 Oct 3;13(10):R89. doi: 10.1186/gb-2012-13-10-r89 (PMC3491417; doi:10.1186/gb-2012-13-10-r89)
Supplement: Additional file 4 — All bisulfite sequencing data. Compilation of all bisulfite sequences. [file gb-2012-13-10-r89-S4.zip › IAPti1072970530_TE_gene_kidney_129allelefromB6129hybrids.rtf]

>129kidney3
TTATTTTTTGATTGGTTGTAGTTTATCGGTCGAGTTGATGTTACGGGGAAGGTAGAGTAT
AAGTAGTTGTAAGATATTTTTGGTATATGCGTAGATTATTTGTTTATTATTTAGAACTAG
GATGTCAGCGTTATTTTGTAACGCGAATGTGGGGGCGGTTTTTAATATTTATTAAAGTAG
AATATCGGTGTTAATAATATTAAGAGTTGAATTATCGATTTTGTTTTTTATAAAAATTGA
AGATAGTTTATTAGTAGGGAAGAAAAAAAATGATTCTTTTTTTTTTATTTTGAGGATAGT
AAGGGTGATTTATTGTTAGGGATGGGGAAAGAAGTTTTGGGAAGTGAAGGGTATGAGGGT
AGAGGATGTTAGAGGGTTAGAAAAGAAGTTTTGAGGTTAGATGTTTAGAGGAGGGTGTGG
TTTAGTTGGGTAGTAGGATTATTTATAATTGTGTTGGTTGTGAGTGTATATTGGGGTGTT
TTTGATTTTGGTTGTTAGAGGGTGTAGTTTGAGTTGGTGTGGTGTTGGGAAGGAGGAGGG
GAGTGTGAGAAGGGTTAGGTGTGTAGGGTGTTTGTGTAGTTTGGTTTTGGGAAGGGTGTG
GAGTTTTTGTTTTTGGGATGTGGTTTTTTGTGTTGTTGGTGTTGTTTAGTTGTTTTGTGG
GTAAGTAGTGAGTGGTTCGGGTGGGTGTGGGGAGGGAGTTTTGGGGTTGTTGGTTATTTT
TGTGTGGTTTTGGTTGTTGTTTGTTTTGTTTTGTTTTGTTTTGGTTGTAGTGTTTTTTAG
TGGTGTTATTTTTGGTTTGTGTTTTTTTTATTTTTGTTTTTTTTTGTCGGGGTATGTGGG
TTGGTGGGGAGGGTGTGGTTGGGTTGGGAGTTAGAGTTGTGTAATTGTTAGAAATTTTAT
AAGAGG


>129kidney10
GGAAGGTTAGAATATATGGGGTGGAGAATTATTTTGGTATATGCGTAGATTATTTGTTTT
ATTATTTAGAATATAGGATGTTTAGCGTTATTTTGTAAACGGCGAATGTGGGGGCGGTTT
TTTAATATTTATTAAAGTAGAATATCGGTGTTAATAATATTAAGAGTTGAATTATCGATT
TTGTTTTTTATAAAAATTGAAGATAGTTTATTAGTAGGGAAGAAAAAAAACGATTTTTTT
TTTTTTTATTTTTGAGGATAGTAAGGGTGATTTATTGTTAGGGATGGGGAAAGAAGTTTT
GGGAAGTGAAGGGTATGAGGGTAGAGGATGTTAGAGGGTTAGAAAAGAAGTTTTGAGGTT
AGATGTTTAGAGGAGGGTGTGGTTTAGTTGGGTAGTAGGATTATTTATAATTGTGTTGGT
TGTGAGTGTATATTGGGGTGTTTTTGATTTTGGTTGTTAGAGGGTGTAGTTTGAGTTGGT
GTGGTGTCGGGAAGGAGGAGGGGAGTGTGAGAAGGGTTAGGTGTGCAGGGTGTTCGTGTA
GTTTGGTTTTGGGAAGGGTGTGGAGTTTTTGTTTTTGGGATGTGGTTTTTTGTGTTGTTG
GTGTTGTTTAGTTGTTTTGTGGGTAAGTAGTGAGTGGTTTGGGTGGGTGTGGGGAGGGAG
TTTTGGGGTTGCTGGTTATTTTTGTGTGGTTTTGGTTGTTGTTTGTTTTGTTTTGTTTTG
TTTTGATTGTAGTGTTTTTTAGTGGTGTTATTTTTGGTTTGTGTTTTTTTTATTTTTGTT
TTTTTTTGTTGGGGTATGTGGGTTGGTGGGGAGGGTGTGGTTGGGTTGGGAGCTAGAGTT
GTGTAATTGTTAGAAATTTTATAAGAGGT

>B3-129k2
TTATTTTTTGATTGGTTGTAGTTTATCGGTCGAGTTGATGTTACGGGGAAGGTAGAGTAT
AAGTAGTTATAAGATATTTTTTGGTATATGCGTAGATTATTTGTTTATTATTTAGAATAT
AGGATGTTAGCGTTATTTTGTAACGCCGAATGTGGGGGCGGTTTTTAATATTTATTAAAG
TAGAATATCGGTGTTAATAATATTAAGAGTTGAATTATCGATTTTGTTTTTTATAAAAAT
TGAAGATAGTTTATTAGTAGGGAAGAAAAAAAACGATTTTTTTTTTTTTATTTTGAGGAT
AGTAAGGGTGATTTATTGTTAGGGATGGGGAAAGAAGTTTTGGGAAGTGAAGGGTATGAG
GGTAGAGGATGTTAGAGGGTTAGAAAAGAAGTTTTGAGGTTAGATGTTTAGAGGAGGGTG
TGGTTTAGTTGGGTAGTAGGATTATTTATAATTGTGTTGGTTGTGAGTGTATATTGGGGT
GTTTTTGATTTTGGTTGTTAGAGGGTGTAGTTTGAGTTGGTGTGGTGTTGGGAAGGAGGA
GGGGAGTGTGAGAAGGGTTAGGTGTGTAGGGTGTTTGTGTAGTTTGGTTTTGGGAAGGGT
GTGGGGTTTTTGTTTTTGGGATGTGGTTTTTTGTGTTGTTGGTGTTGTTTAGTTGTTTTG
TGGGTAAGTAGTGAGTGGTTTGGGTGGGTGTGGGGAGGGAGTTTTGGGGTTGTTGGTTAT
TCTTGTGTGGTTTTGGTTGTTGTTTGTTTTGTTTTGTTTTGTTTTGGTTGTAGTGTTTTT
TAGTGGTGTTATTTTTGGTTTGTGTTTTTTTTATTTTTGTTTTTTTTTGTTGGGGTATGT
GGGTTGGTGGGGAGGGTGTGGTTGGGTTGGGAGTTAGAGTTGTGTAATTGTTAGAAATTT
TATAAGAGGT

>B3-129k3
TTATTTTTTGATTGGTTGTAGTTTATCGGTCGAGTTGACGTTACGGGGAAGGTAGAGTAC
AAGTAGTTATAAGATATTTTTGGTATATGCGTAGATTATTTGTTTATTATTTAGAATATA
GGATGTTACGTTATTTTGTAACGGCGAATGTGGGGCGGTTTTTAATATTTATTAAAGTAG
AATATCGGTGTTAATAATATTAAGAGTTGAATTATCGATTTTGTTTTTTATAAAAATTGA
AGATAGTTTATTAGTAGGGAAGAAAAAAAATGATTTTTTTTTTTCATTTTGAGGATAGTA
AGGGTGATTTATTGTTAGGGATGGGGAAAGAAGTTTTGGGAAGTGAAGGGTATGAGGGTA
GAGGATGTTAGAGGGTTAGAAAAGAAGTTTTGAGGTTAGATGTTTAGAGGAGGGTGTGGT
TTAGTTGGGTAGTAGGATTATTTATAATTGTGTTGGTTGTGAGTGTATATTGGGGTGTTT
TTGATTTTGGTTGTTAGAGGGTGTAGTTTGAGTTGGTGTGGTGTTGGGAAGGAGGAGGGG
AGTGTGAGAAGGGTTAGGTGTGTAGGGTGTTTGTGTAGTTTGGTTTTGGGAAGGGTGTGG
AGTTTTTGTTTTTGGGATGTGGTTTTTTGTGTTGTTGGTGTTGTTTAGTTGTTTTGTGGG
TAAGTAGTGAGTGGTTTGGGTGGGTGTGGGGAGGGAGTTTTGGGGTTGTTGGTTATTTTT
GTGTGGTTTTGGTTGTTGTTTGTTTTGTTTTGTTTTGTTTTGGTTGTAGTGTTTTTTAGT
GGTGTTATTTTTGGTTTGTGTTTTTTTTATTTTTGTTTTTTTTGTTGGGGTATGTGGGTT
GGTGGGGAGGGTGTGGTTGGGTTGGGAGTTAGAGTTGTGTAATTGTTAGAAATTTTATAA
GAGGT

>B3-129k4
TTAATTTTTTGATTGGTTGTAGTTTATCGGTCGAGTCGATGTTACGGGGAAGGTAGAGTA
TAAGTAGTTATAAGATATTTTTGGTATATGCGTAGATTATTTGTTTATTATTTAGAATAT
AGGATGTTAGCGTTATTTTGTAACGGCGAATGTGGGGGCGGTTTTTAATATTTATTAAAG
TAGAATATCGGTGTTAATAATATTAAGAGTTGAATTATCGATTTTGTTTTTTACAAAAAT
TGAAGATAGTTTATTAGTAGGGAAGAAAAAAACGATTTTTTTTTTTTTATTTTGAGGATA
GTAAGGGTGATTTATTGTTAGGGATGGGGAAAGAAGTTTTGGGAAGTGAAGGGTATGAGG
GTAGAGGATGTTAGAGGGTTAGAAAAGAAGTTTTGAGGTTAGATGTTTAGAGGAGGGTGT
GGTTTAGTTGGGTAGTAGGATTATTTATAATTGTGTTGGTTGTGAGTGTATATTGGGGTG
TTTTTGATTTTGGTTGTTAGAGGGTGTAGTTTGAGTTGGTGTGGTGTTGGGAAGGAGGAG
GGGAGTGTGAGAAGGGTTAGGTGTGTAGGGTGTTTGTGTAGTTTGGTTTTGGGAAGGGTG
TGGAGTTTTTGTTTTTGGGATGTGGTTTTTTGTGTTGTTGGTGTTGTTTAGTTGTTTTGT
GGGTAAGTAGTGAGTGGTTTGGGTGGGTGTGGGGAGGGAGTTTTGGGGTTGTTGGTTATT
TTTGTGTGGTTTTGGTTGTTGTTTGTTTTGTTTTGTTTTGTTTTGGTTGTAGTGTTTTTT
AGTGGTGTTATTTTTGGTTTGTGTTTTTTTATTTTTGTTTTTTTTTGTTGGGGTATGTGG
GTTGGTGGGGAGGGTGTGGTTGGGTTGGGAGTTAGAGCTGTGTAATTGTTAGAAATTTTA
TAAGAGGT

>B3-129k6
TTATTTTTTGATTGGTTGTAGTTTATCGGTCGAGTTGATGTTACGGGGAAGGTAGAGTAT
AAGTAGTTATAAGATATTTTTGGTATATGCTAGATTATTTGTTTATTATTTAGAATATAG
GATGTTAGCGTTATTTTGTAACGGCGAATGTGGGGGCGGTTTTTAATATTTATTAAAGTA
GAATATCGGTGTTAATAATATTAAGAGTTGAATTATCGATTTTGTTTTTTATAAAAATTG
AAGATAGTTTATTAGTAGGGAAGAAAAAAAATGATTTTTTTTTTTTATTTTGAGGATAGT
AAGGGTGATTTATTGTTAGGGATGGGGAAAGAAGTTTTGGGAAGTGAAGGGTATGAGGGT
AGAGGATGTTAGAGGGTTAGAAAAGAAGTTTTGAGGTTAGATGTTTAGAGGAGGGTGTGG
TTTAGTTGGGTAGTAGGATTATTTATAATTGTGTTGGTTGTGAGTGTATATTGGGGTGTT
TTTGATTTTGGTTGTTAGAGGGTGTAGTTTGAGTTGGTGTGGTGTCGGGAAGGAGGAGGG
GAGTGTGAGAAGGGTTAGGTGTGTAGGGTGTTTGTGTAGTTTGGTTTTGGGAAGGGTGTG
GAGTTTTTGTTTTTGGGATGTGGTTTTTTGTGTTGTTGGTGTTGTTTAGTTGTTTTGTGG
GTAAGTAGTGAGTGGTTTGGGTGGGTGTGGGGAGGGAGTTTTGGGGTTGTTGGTTATTTT
TGTGTGGTTTTGGTTGTTGTTTGTTTTGTTTTGTTCTGTTTTGGTTGTAGTGTTTTTTAG
TGGTGTTATTTTTGGTTTGCGTTTTTTTTATTTTTGTTTTTTTTTGTTGGGGTATGTGGG
TTGGTGGGGAGGGTGTGGTTGGGTTGGGAGTTAGAGTTGGGTAATTGTTAGAAATTTTAT
AAGAGGT

>B3-129k7
TTATTTTTTGATTGGTTGTAGTTTATCGGTCGAGTTGACGTTACGGGGAAGGTAGAGTAT
AAGTAGTTATAAGATATTTTTGGTATATGCGCAGATTATTTGTTTATTAATTTAGAATAT
AGGATGTTAGCGTTATTTTGTAACGGCGAATGTGGGGGCGGTTTTTAATATTTATTAAAG
TAGAATATCGGTGTTAATAATATTAAGAGTTGAATTATCGATTTTGTTTTTTATAAAAAT
TGAAGATAGTTTATTAGTAGGGAAGAAAAAAAACGATTTTTTTTTTTTATTTTGAGGATA
GTAAGGGTGATTTATTGTTAGGGATGGGGAAAGAAGTTTTGGGAAGTGAAGGGTATGAGG
GTAGAGGATGTTAGAGGGTTAGAAAAGAAGTTTTGAGGTTAGATGTTTAGAGGAGGGTGT
GGTTTAGTTGGGTAGTAGGATTATTTATAATTGTGTTGGTTGTGAGTGTATATTGGGGTG
TTTTTGATTTTGGTTGTTAGAGGGTGTAGTTTGAGTTGGTGTGGTGTTGGGAAGGAGGAG
GGGAGTGTGAGAAGGGTTAGGTGTGTAGGGTGTTTGTGTAGTTTGGTTTTGGGAAGGGTG
TGGAGTTTTTGTCTTTGGGATGTGGTTTTTTGTGTTGTTGGTGTTGTTTAGTTGTTTTGT
GGGTAAGTAGTGAGTGGTTTGGGTGGGTGTGGGGAGGGAGTTTTGGGGTTGTTGGTTATT
TTTGTGTGGTTTTGGTTGTTGTTTGTTTTGTTTTGTCTTGTTTTGGTTGTAGTGTTTTTT
AGTGGTGTTATTTTTGGTTTGTGTTTTTTTTATTTTTGTTTTTTTTTGCTGGGGTATGTG
GGTTGGTGGGGAGGGTGTGGTTGGGTTGGGAGTTAGAGTTGTGTAATTGTTAGAAATTTT
ATAAGAGGT

>B3-129k8
TTATTTTTTGATTGGTTGTAGTTTATCGGTCGAGTTGACGTTACGGGGAAGGTAGAGTAC
AAGTAGTTATAAGATATTTTTGGTATATGCGTAGATTATTTGTTTATTATTTAGAATATA
GGATGTTAGCGTTATTTTGTAACGGCGAATGTGGGGGCGGTTTTTAATATTTATTAAAGT
AGAATATCGGTGTTAATAATATTAAGAGTTGAATTATCGATTTTGTTTTTTATAAAAATT
GAAGATAGTTTATTAGTAGGGAAGAAAAAAAATGATTTTTTTTTTTTCATTTTGAGGATA
GTAAGGGTGATTTATTGTTAGGGATGGGGAAAGAAGTTTTGGCAAGTGAAGGGTATGAGG
GTAGAGGATGTTAGAGGGTTAGAAAAGAAGTTTTGAGGTTAGATGTTTAGAGGAGGGTGT
GGTTTAGTTGGGTAGTAGGATTATTTATAATTGTGTTGGTTGTGAGTGTATATTGGGGTG
TTTTCGATTTTGGTTGCTAGAGGGTGTAGTTTGAGTTGGTGTGGTGTTGGGAAGGAGGAG
GGGAGCGTGAGAAGGGTTAGGTGTGTAGGGTGTTTGTGTAGTTTGGTTTTGGGAAGGGTG
TGGAGTTTTTGTTTTTGGGATGTGGTTTTTTGTGTTGTTGGCGTTGTTTAGTTGTTTTGT
GGGTAAGTAGTGAGTGGTTTGGGTGGGTGTGGGGAGGGAGTTTTGGGGTTGTTGGTTATT
TTTGTGTGGTTTTGGTTGTTGTTTGTTTTGTTTTGTTTTGTTTTGGTTGTAGTGTTTTTT
AGTGGTGTTATTTTTGGTTTGTGTTTTTTTTATTTTTGTTTTTTTTCGTTGGGGTATGTG
GGTTGGTGGGGAGGGTGTGGTTGGGTTGGGAGTTAGAGCTTGTGTAATTGTTAGAAATTT
TATAAGAGGT
