# Supplementary material for: Epigenetic interplay between mouse endogenous retroviruses and host genes
Source: Genome Biol. 2012 Oct 3;13(10):R89. doi: 10.1186/gb-2012-13-10-r89 (PMC3491417; doi:10.1186/gb-2012-13-10-r89)
Supplement: Additional file 4 — All bisulfite sequencing data. Compilation of all bisulfite sequences. [file gb-2012-13-10-r89-S4.zip › IAPti1072970530_TE_gene_kidney_B6allelefromB6129hybrids.rtf]

>B6kidney1_SP6
GGGTTAAAGTTATTAATTAAATTTTATTTAAGAAGAGGATATTAAGTAAATTAGTTGAGT
TGATTAAAAATATTTTTATTAAAGTAGAATATCGGTGTTAATAATATTAAGAGTTGAATT
ATTGATTTTGTTTTCTATAAAAATTGAAGATAGTTTATTAGTAGGGAAGAAAAAAATGAT
TTTTTTTTTTTTATTTTGAGGATAGTAAGGGTGATTTATTGTTAGGGATGGGGAAAGAAG
TTTTGGGAAGTGAAGGGTATGATGGTAGAGTTTGTTAGAGGGTTAGAAAAGAAGTTTTGA
GGTTAGATGTTTAGAGGAGGGTGTGGTTTAGTTGGGTAGTAGGATTATTTATAATTGTGT
TGGTTGTGAGTGTATATTGGGGTGTTTTTGATTTTGGTTGTTAGAGGGTGTAGTTTGAGT
TGGCGTGGTGTTGGGAAGGAGGAGGGGAGTGTGAGAAGGGTTAGGCGTGTAGGGTGTTTG
TGTAGTTTGGTTTTGGGAAGGGTGTGGAGTCTTTGTTTTTGGGATGTGGTTTTTTGTGTT
GTTGGCGTTGTTTAGTTGTTTTGTGGGTAAGTAGTGAGTGGTTTGGGTGGGTGTGGGGAG
GGAGTTTTGGGGTTGTTGGTTATTTTTGTGTGGTTTTGGTTGTTGTTTGTTTTGTTTTGT
TTTGTTTTGGCTGTAGTGTTTTTTGGTGGTGTTATTTTTGGTTTGTGTTTTTTTTATTTT
TGTTTTTTTTGTTGGGGTATGTGGGTTGGTGGGGAGGGTGTGGTCGGGTTGGGAGTTAGA
GTTGTGTAATTGTTAGAAATTTTATAAGAGG

>B6kidney2_SP6
GGGTTAAAGTTATTAATTAAATTTTATTTAAGAAGAGGATATTAAGTAAATTTAGTTGAG
TTGATTAAAAATATTTTTATTAAAGTAGAATATTGGTGTTAATAATATTAAGAGTTGAAT
TATCGATTTTGTTTTTTATAAAAATTGAAGATAGTTTATTAGTAGGGAAGAAAAAAATGA
TTCTTTTTTTTTTATTTTGAGGATAGTAAGGGTGATTTATTGTTAGGGATGGGGAAAGAA
GTTTTGGGAAGTGAAGGGTATGATGGTAGAGTTTGTTAGAGGGTTAGAAATGAAGTTTTG
AGGTTAGATGTTTAGAGGAGGGTGTGGTTTAGTTGGGTAGTAGGATTATTTATAATTGTG
TTGGTTGTGAGTGTATATTGGGGTGTTTTTGATTCNGGTTGTTAGAGGGTGTAGTTTGAG
TCGGTGTGGTGTTGGGAAGGAGGAGGGGAGTGTGAGAAGGGTTAGGTGTGTAGGGTGTTT
GTGTAGTTTGGTTTTGGGAAGGGTGTGGAGTTTTTGTTTTTGGGATGTGGTTTTTTGTGT
TGTTGGTGTTGTTTAGTTGTTTTGTGGGTAAGTAGTGAGTGGTTTGGGTGGGTGTGGGGA
GGGAGTTTTGGGGTTGTTGGTTATTTTTGTGTGGTTTTGGTTGTTGTTTGTTTTGTTTTG
TTTTGTTTTGGTTGTAGTGTTTTTTAGTGGTGTTATTTTTGGTTTGTGTTTTTTTTATTT
TTGTTTTTTTTTGTTGGGGTATGTGGGTTGGTGGGGAAGGTGTGGTTGGGTTGGGAGTTA
GAGTTGTGTAATTGTTAGAAATTTTATAAGAGG

>B6kidney3_SP6
GGGTTAAAGTTATTAATTAAATTTTATTTAAGAAGAGGATATTAAGTAAATTAGTTGAGT
TGATTAAAAATATTTTTATTAAAGTAGAATATTGGTGTTAATAATATTAAGAGTTGAATT
ATTGATTTTGTTTTTTATAAAAATTGAAGATAGTTTATTAGTAGGGAAGAAAAAAATGAT
TTTTTTTTTTTTATTTTGAGGATAGTAAGGGTGATTTATTGTTAGGGATGGGGAAAGAAG
TTTTGGGAAGTGAAGGGTATGATGGTAGAGTTTGTTAGAGGGTTAGAAAAGAAGTTTCGA
GGTTAGATGTTTAGAGGAGGGTGTGGTTTAGTTGGGTAGTAGGATTATTTATAATTGTGT
TGGTTGTGAGTGTATATTGGGGTGTTTTTGATTTTGGTTGTTAGAGGGTGTAGTTTGAGT
TGGTGTGGTGTTGGGAAGGAGGAGGGGAGTGTGAGAAGGGTTAGGTGTGTAGGGTGTTTG
TGTAGTTTGGTTTTGGGAAGGGTGTGGAGTTTTTGTTTTTGGGATGTGGTTTTTTGTGTT
GTTGGTGTTGTTTAGTTGTTTTGTGGGTAAGTAGTGAGTGGTTTGGGTGGGTGTGGGGAG
GGAGTTTTGGGGTTGTTGGTTATTTTTGTGTGGTTTTGGTTGTTGTTTGTTTTGTTTCGT
TTTGTTTTGGTTGTAGTGTTTTTTAGTGGTGTTATTTTTGGTTTGTGTTTTTTTTATTTT
TGTTTTTTTTTGTTGGGGTATGTGGGTTGGTGGGGAGGGTGTGGTTGGGTTGGGAGTTAG
AGTTGTGTAATTGTTAGAAATTTTATAAGAGG

>B6kidney4_SP6
GGGTTAAAGTTATTAATTAAATTTTATTTAAGAAGAGGATATTAAGTAAATTAGTTGAGT
TGATTAAAAATATTTTTATTAAAGTAGAATATTGGTGTTAATAATATTAAGGGTTGAATT
ATTGATTTTGTTTTTTATAAAAATTGAAGATAGTTTATTAGTAGGGAAGAAAAAAATGAT
TTTTTTTTTTTTATTTTGAGGATAGTAAGGGTGATTTATTGTTAGGGATGGGGAAAGAAG
TTTTGGGAAGTGAAGGGTATGATGGTAGAGTTTGTTAGAGGGTTAGAAAAGAAGTTTTGA
GGTTAGATGTTTAGAGGAGGGTGTGGTTTAGTTGGGTAGTAGGATTATTTATAATTGTGT
TGGTTGTGAGCGTATATTGGGGTGTTTTTGATTTTGGTTGTTAGAGGGTGTAGTTTGAGT
TGGTGTGGTGTTGGGAAGGAGGAGGGGAGTGTGAGAAGGGTTAGGTGTGTAGGGTGTTCG
TGTAGTTTGGTTTTGGGAAGGGTGTGGAGTTTTTGTTTTTGGGATGTGGTTTTTTGTGTT
GTTGGTGTTGTTTAGTTGTTTTGTGGGTAAGTAGTGAGTGGTTTGGGTGGGTGTGGGGAG
GGAGTTTTGGGGTTGTTGGTTATTTTTGTGTGGTTTTGGTTGTTGTTTGTTTTGTTTTGT
TTTGTTTTGGTTGTAGTGTTTTTTAGTGGTGTTATTTTTGGCTTGTGTTTTTTTTATTTT
CGTTTTTTTTTGTTGGGGTATGTGGGTTGGTGGGGAGGGTGTGTCTGGGTTGGGAGTTAG
AGTGTGTAATTGTTAGAAATTTTATAAGAGG

>B6kidney5_SP6
GGGTTAAAGTTATTAATTAAATTTTATTTAAGAAGAGGATATTAAGTAAATTAGTTGAGT
TGATTAAAAATATTTTTATTAAAGTAGAATATCGGTGTTAATAATATTAAGAGTTGAATT
ATCGATTTTGTTTTTTATAAAAATTGAAGATAGTTTATTAGTAGGGAAGAAAAAAATGAT
TTTTTTTTTTTTATTTTGAGGATAGTAAGGGTGATTTATTGTTAGGGATGGGGAAAGAAG
TTTTGGGAAGTGAAGGGTATGATGGTAGAGTTTGTTAGAGGGTTAGAAAAGAAGTTTTGA
GGTTAGATGTTTAGAGGAGGGTGTGGTTTAGTTGGGTAGTAGGATTATTTATAATTGTGT
TGGTTGTGAGTGTATATTGGGGTGTTTTTGATTTTGGTTGTTAGAGGGTGTAGTTTGAGT
TGGTGTGGTGTTGGGAAGGAGGAGGGGAGTGTGAGAAGGGTTAGGTGTGTAGGGTGTTTG
TGTAGTTTGGTTTTGGGAAGGGTGTGGAGTTTTTGTTTTTGGGATGTGGTTTTTTGTGTT
GTTGGTGTTGTTTAGTTGTTTTGTGGGTAAGTAGTGAGTGGTTTGGGTGGGTGTGGGGAG
GGAGTTTCGGGGTTGTTGGTTATTTTTGTGTGGTTTTGGTTGTTGTTTGTTTTGTTTTGT
TTTGTTTCGGTTGTAGTGTTTTTTAGTGGTGTTATTTTTGGTTTGTGTTTTTTTTATTTT
TGTTTTTTTTTGTTGGGGTATGTGGGTTGGTGGGGAGGGTGTGGTTGGGTTGGGAGTTAG
AGTTGTGTAATTGTTAGAAATTTTATAAGAGG

>B6kidney7_SP6
GGGTTAAAGTTATTAATTAAATTTTATTTAAGAAGAGGATATTAAGTAAATTAGTTGAGT
TGATTAAAAATATTTTTATTAAAGTAGAATACGGTGTTAATAATATTAAGAGTTGAATTA
TCGATTTTGTTTTTTATAAAAATTGAAGATAGTTTATTAGTAGGGAAGAAAAAAATGATT
TTTTTTTTTTTATTTTGAGGATAGTAAGGGTGATTTATTGTTAGGGATGGGGAAAGAAGT
TTTGGGAAGTGAAGGGTATGATGGTAGAGTTTGTTAGAGGGTTAGAAAAGAAGTTTTGAG
GTTAGATGTTTAGAGGAGGGTGTGGTTTAGTTGGGTAGTAGGATTATTTATAATTGTGTT
GGTTGTGAGTGTATATTGGGGTGTTTTTGATTTTGGTTGTTAGAGGGTGTAGTTTGAGTT
GGTGTGGTGTTGGGAAGGAGGAGGGGAGTGTGAGAAGGGTTAGGTGTGTAGGGTGTTTGT
GTAGTTTGGTTTTGGGAAGGGTGTGGAGTTTTTGTTTTTGGGATGTGGTTTTTTGCGTTG
TTGGTGTTGTTTAGTTGTTTTGTGGGTAAGTAGTGAGTGGTTTGGGTGGGTGTGGGGAGG
GAGTTTTGGGGTTGTTGGTTATTTTTGTGTGGTTTTGGTTGTTGTTTGTTTTGTTTTGTT
TTGTTTTGGTTGTAGTGTTTTTTAGTGGTGTTATTTTTGGTTTGTGTTTTTTTTATTTTT
GTTTTTTTTTGTTGGGGTACGTGGGTTGGTGGGGAGGGTGTGGTTGGGTTGGGAGTTAGA
GTTGTGTAATTGTTAGAAATTTTATAAGAGG

>B6kidney8_SP6
GGGTTAAAGTTATTAATTAAATTTTATTTAAGAAGAGGATATTAAGTAAATTAGTTGAGT
TGATTAAAAATATTTTTATTAAAGTAGAATATCGGTGTTAATAATATTAAGAGTTGAACT
ATCGATTTTGTTTTTTATAAAAATTGAAGATAGTTTATTAGTAGGGAAGAAAAAAATGAT
TTTTTTTTTTTTATTTTGAGGATAGTAAGGGTGATTTATTGTTAGGGATGGGGAAAGAAG
TTTTGGGAAGTGAAGGGTATGATGGTAGAGTTTGTTAGAGGGTTAGAAAAGAAGTTTTGA
GGTTAGATGTTTAGAGGAGGGTGTGGTTTAGTTGGGTAGTAGGATTATTTATAATTGTGT
TGGTTGTGAGTGTATATTGGGGTGTTTTTGATTTTGGTTGTTAGAGGGTGTAGTTTGAGT
TGGTGTGGTGTTGGGAAGGAGGAGGGGAGTGTGAGAAGGGTTAGGTGTGTAGGGTGTTTG
TGTAGTTTGGTTTTGGGAAGGGTGTGGAGTTTTTGTTTTTGGGATGTGGTTTTTTGTGTT
GCTGGTGTTGTTTAGTTGTTTTGTGGGTAAGTAGTGAGTGGTTTGGGTGGGTGTGGGGAG
GGAGTTTTGGGGTTGTGGGTTATTTTTGTGTGGTTTTGGTTGTTGTTTGTTTTGTTTTGT
TTTGTTTTGGTTGTAGTGTTTTTTAGTGGTGTTATTTTTGGTTTGTGTTTTTTTTATTTT
TGTTTTTTTTTGTTGGGGTATGTGGGTTGGTGGGGAGGGTGTGGTTGGGTTGGGAGTTAG
AGTTGTGTAATTGTTAGAAATTTTATAAGAGG

>B6kidney9_SP6
GGGTTAAAGTTATTAATTAAATTTTATTTAAGAAGAGGATATTAAGTAAATTAGTTGAGT
TGATTAAAAATATTTTTATTAAAGTAGAAAATCGGTGTTAATAATATTAAGAGTTGAATT
ATCGATTTTGTTTTTTATAAAAATTGAAGATAGTTTATTAGTAGGGAAGAAAAAAATGAT
TTTTTTTTTTTATTTTGAGGATAGTAAGGGTGATTTATTGTTAGGGATGGGGAAAGAAGT
TTTGGGAAGTGAAGGGTATGATGGTAGAGTTTGTTAGAGGGTTAGAAAAGAAGTTTTGAG
GTTAGATGTTTAGAGGAGGGTGTGGTTTAGTTGGGTAGTAGGATTATTTATAATTGTGTT
GGTTGTGAGTGTATATTGGGGTGTTTTTGATTTTGGTTGTTAGAGGGTGTAGTTTGAGTT
GGTGTGGTGTTGGGAAGGAGGAGGGGAGTGTGAGAAGGGTTAGGTGTGTAGGGTGTTTGT
GTAGTTTGGTTTTGGGAAGGGTGTGGAGTTTTTGTTTTTGGGATGTGGTTTTTTGCGTTG
TTGGTGTTGTTTAGTTGTTTTGTGGGTAAGTAGTGAGTGGTTTGGGTGGGTGTGGGGAGG
GAGTTTTGGGGTTGTTGGTTATTTTTGTGTGGTTTTGGTTGTTGTTTGTTTTGTTTTGTT
TTGTTTTGGTTGTAGTGTTTTTTAGTGGTGTTATTTTTGGTTTGTGTTTTTTTTATTTTT
GTTTTTTTTTGTTGGGGTATGTGGGTTGGTGGGGAGGGTGTGGTTGGGTTGGGAGTTAGA
GTTGTGTAATTGTTAGAAATTTTATAAGAGG

>B6kidney10_SP6
GGGTTAAAGTTATTAATTAAATTTTATTTAAGAAGAGGATATTAAGTAAATTAGTTGAGT
TGATTAAAAATATTTTTATTAAAGTAGAATATCGGTGTTAATAATATTAAGAGTTGAATT
ATTGATTTTGTTTTTTATAAAAATTGAAGATAGTTTATTAGTAGGGAAGAAAAAAATGAT
TTTTTTTTTTTTATTTTGAGGATAGTAAGGGTGATTTATTGTTAGGGATGGGGAAAGAAG
TTTTGGGAAGTGAAGGGTATGATGGTAGAGTTTGTTAGAGGGTTAGAAAAGAAGTTTTGA
GGTTAGATGTTTAGAGGAGGGTGTGGTTTAGTTGGGTAGTAGGATTATTTATAATTGTGT
TGGTTGTGAGTGTATATTGGGGTGTTTTCGATTTTGGTTGTTAGAGGGTGTAGTTTGAGT
TGGTGTGGTGTTGGGAAGGAGGAGGGGAGTGTGAGAAGGGTTAGGTGTGTAGGGTGTTTG
TGTAGTTTGGTTTTGGGAAGGGTGTGGAGTTTTTGTTTTTGGGATGTGGTTTTTTGTGTT
GTTGGTGTTGTTTAGTTGTTTTGTGGGTAAGTAGTGAGTGGTTTGGGTGGGTGTGGGGAG
GGAGTTTTGGGGTTGTTGGTTATTTTTGTGTGGTTTTGGTTGTTGTTTGTTTTGTTTTGT
TTTGTTTTGGTTGTAGTGTTTTTTAGTGGTGTTATTTTTGGTTTGTGTTTTTTTTATTTT
TGTTTTTTTTTGTTGGGGTATGTGGGTTGGTGGGGAGGGTGTGGTTGGGTTGGGAGTTAG
AGTTGTGTAATTGTTAGAAATTTTATAAGAGG
>B6kidney11_SP6
GGGTTAAAGTTATTAATTAAATTTTATTTAAGAAGAGGATATTAAGTAAATTAGTTGAGT
TGATTAAAAATATTTTTATTAAAGTAGAATATCGGTGTTAATAATATTAAGAGTTGAATT
ATCGATTTTGTTTTTTATAAAAATTGAAGATAGTTTATTAGTAGGGAAGAAAAAAATGAT
TTTTTTTTTTTTATTTTGAGGATAGTAAGGGTGATTTATTGTTAGGGATGGGGAAAGAAG
TTTTGGGAAGTGAAGGGTATGATGGTAGAGTTTGTTAGAGGGCTAGAAAAGAAGTTTTGA
GGTTAGATGTTTAGAGGAGGGTGTGGTTTAGTTGGGTAGTAGGATTATTTATAATTGTGT
TGGTTGTGAGTGTATATTGGGGTGTTTTTGATTTTGGTTGTTAGAGGGTGTAGTTTGAGT
TGGTGTGGTGTTGGGAAGGAGGAGGGGAGTGTGAGAAGGGTTAGGTGTGTAGGGTGTTTG
TGTAGTTTGGTTTTGGGAAGGGTGTGGAGTTTTTGTTTTTGGGATGTGGTTTTTTGTGTT
GTTGGTGTTGTTTAGTTGTTTTGTGGGTAAGTAGTGAGTGGTTTGGGTGGGTGTGGGGAG
GGAGTTTTGGGGTTGTTGGTTATTTTTGTGTGGTTTTGGTTGTTGTTTGTTTTGTTTTGT
TTCGTTTTGGTTGTAGTGTTTTTTAGTGGTGTTATTTTTGGTTTGTGTTTTTAATTGATT
TATTTTTATTAAGTGTGTTTGTAATTGTTAGAAATTTTATAAGAGG
