# Supplementary material for: Epigenetic interplay between mouse endogenous retroviruses and host genes
Source: Genome Biol. 2012 Oct 3;13(10):R89. doi: 10.1186/gb-2012-13-10-r89 (PMC3491417; doi:10.1186/gb-2012-13-10-r89)
Supplement: Additional file 4 — All bisulfite sequencing data. Compilation of all bisulfite sequences. [file gb-2012-13-10-r89-S4.zip › IAP1859_TE_oppositeLTR_brain.doc]

Ying’s Polymorphic Case 74

Right LTR-B6AJ Brain (5’LTR-Chr 6)

>74RLTR_1

ATGTGAAAAAGAATAAAAGGGTTTTTAATATTAGAAAAAATTTAAGGTTAAATATATTTA

TTGGTATTTTCGTTTTTTAGTTGAAAAGTTTTGAATTTATATAGTTGAATTTTTTTTAAT

AGTTTGTTTTACGGGAATTTTTATTATCGTCGTTTTTTAGTTGATGAGTTTTGAATTCGG

TAGTTGAATTTTTTTAATAGTTTGTGTTACGGGAATTTTTATAAACGTGATTCGTAGTTT

TGGTTTTGAAATGAAGTATTTTTTTTGCGTTAGTTCGGAGTTTTTTTTCGTTTCGGAATT

CGGTATTAATTGTTATTCGATGCGTTTTTACGATCGGTTAGGAAGAATATAATAATTAGA

ATTTTTTACGGTAAAGTTTTATTGTTTATATTTTTATGGGGTTAGAGTGTAAGAAGTAAG

AGCGAGAGTAAGAGAGAGAGAAAAACGAAATTTTTTTTATTTTAAAGAGAATAATTATTG

TTTAGGGCGTATTATTTTTTGATTGGTTGTAGTTTATGGTCGAGTTGACGTTTACGGGAA

AAATAGAGTATAAGTAGTCGTAAATATTTTTGGTTTATGCGTAGATTATTTGTTTATTAA

TTTAGAATATAGGATGTTAGCGTTATTTTGTGACGGCGAATGTGGGGGCGGTTTTTTATA

TATATTTGTTATAGTTTAGTAAGGATGGGTTGGTAGAATAAATTTTTAGAAGAGTTGGTT

TGTTTTTTG

>74RLTR_6

ATGTGAAAAAGAATAAAAGGGTTTTTAATACTAGAAAAAATTTAAGGTTAAATATATTTA

TTGGTATTTTCGTTTTTTAGTTGAAAAGTTTTGAATTTATATAGTTGAATTTTTTTTAAT

AGTTTGTTTTACGGGAATTTTTATTATCGTCGTTTTTTAGTTGATGAGTTTTGAATTCGG

TAGTTGAATTTTTTTTAATAGTTTGTGTTACGGGAATTTTTATAAACGTGATTCGTAGTT

TTGGTTTTGAAATGAAGTATTTTTTTTGCGTTAGTTCGGAGTTTTTTTTCGTTTCGGAAT

TCGGTATCAATTGTTATTCGATGCGTTTTTACGATCGGTTAGGAAGAATATAATAATTAG

AATTTTTTACGGTAAAGTTTTATTGTTTATATTTTTATGGGGTTAGAGTGTAAGAAGCAA

GAGCGAGAGTAAGAGAGAGAGAAAAACGAAATTTTTTTTATTTTAAAGAGAATAATTATT

GTTTAGGGCGTATTATTTTTTGATTGGTTGTAGTTTATGGTCGAGTTGACGTTTACGGGA

AAAACAGAGTATAAGTAGTCGTAAATATTTTTGGTTTATGCGTAGATTATTTGTTTATTA

ATTTAGAATATAGGATGTTAGCGTTATTTTGTGACGGCGAATGTGGGGGCGGTTTTTCAT

ATATATTTGTTATAGTTTAGTAAGGATGGGTTGGTAGAATAAATTTTTAGAAGAGTTGGT

TTGTTTT

>74RLTR_7

ATGTGAAAAAGAATAAAAGGGTTTTTAATATTAGAAAAAATTTAAGGTTAAATATATTTA

TTGGTATTTCGTTTTTTAGTTGAAAAGTTTTGAATTTATATAGTTGAATTTTTTTTAATA

GTTTGTTTTACGGGAACTTTTATTATCGTCGTTTTTTAGTTGATGAGTTTTGAATTCGGT

AGTTGAATTTTTTTTAATAGTTTGTGTTACGGGAATTTTTATAAACGTGATTCGTAGTTT

TGGTTTTGAAATGAAGTATTTTTTTTGCGTTAGTTCGGAGTTTTTTTTCGTTTCGGAATT

CGGTATTAATTGTTATTCGATGCGTTTTTACGATCGGTTAGGAAGAATATAATAATTAGA

ATTTTTTACGGTAAAGTTTTATTGTTTATATTTTTATGGGGTTAGAGTGTAAGAAGTAAG

AGCGAGAGTAAGAGAGAGAGAAAAACGAAATTTTTTTTATTTTAAAGAGAATAATTATTG

TTTAGGGCGTGTTATTTTTTGATTGGTTGTAGTTTATGGTCGAGTTGACGTTTATGGGAA

AAATAGAGTATAAGTAGTTGTAAATATTTTTGGTTTATGCGTAGATTATTTGTTTATTAA

TTTAGAATATAGGATGTTAGCGTTATTTTGTGACGGCGAATGTGGGGGCGGTTTTTTATA

TATATTTGTTATAGTTTAGTAAGGATGGGTTGGTAGAATAAATTTTTAGAAGAGTTGGTT

TGTTTTTTGTTTTT

>74RLTR_8

ATGTGAAAAAGAATAAAAGGGTTTTTAATACTAGAAAAAATTTAAGGTTAAATATATTTA

TTGGTATTTTCGTTTTTTAGTTGAAAAGTTTTGAATTTATATAGTTGAATTTTTTTTAAT

AGTTTGTTTTACGGGAATTTTTATTATCGTCGTTTTTTAGTTGATGAGTTTTGAATTCGG

TAGTTGAATTTTTTTTAATAGTTTGTGTTACGGGAATTTTTATAAACGTGATTCGTAGTT

TTGGTTTTGAAATGAAGTATCTTTTTTGCGTTAGTTCGGAGTTTTTTTTCGTTTCGGAAT

TCGGTATTAATTGTTATTCGATGCGTTTTTACGATCGGTTAGGAAGAATATAATAATTAG

AATTTTTTACGGTAAAGTTTTATTGTTTATATTTTTATGGGGTTAGAGTGTAAGAAGCAA

GAGCGAGAGTAAGAGAGAGAGAAAAACGAAATTTTTTTTATTTTAAAGAGAATAATTATT

GTTTAGGGCGTATTATTTTTTGATTGGTTGTAGTTTATGGTCGAGTTGACGTTTACGGGA

AAAACAGAGTATAAGTAGTCGTAAATATTTTTGGTTTATGCGTAGATTATTTGTTTATTA

ATTTAGAATATAGGATGTTAGCGTTATTTTGTGACGGCGAATGTGGGGGCGGTTTTTCAT

ATATATTTGTTATAGTTTAGTAAGGATGGGTTGGTAGAATAAATTTTTAGAAGAGTTGGT

TTGTTTTTTGTTTTTG

>74RLTR_10

ATGTGAAAAAGAATAAAAGGGTTTTTAATATTAGAAAAAATTTAAGGTTAAATATATTTA

TTGGTATTTTCGTTTTTTAGTTGAAAAGTTTTGAATTTATATAGTTGAATTTTTTTTAAT

AGTTTGTTTTACGGGAATTTTTATTATCGTCGTTTTTTAGTTGATGAGTTTTGAATTCGG

TAGTTGAATTTTTTTTAATAGTTTGTGTTACGGGAATTTTTATAAACGTGATTCGTAGTT

TTGGTTTTGAAATGAAGTATTTTTTTTGCGCTAGTTCGGAGTTTTTTTTCGTTTCGGAAT

TCGGTATTAATTGTTATTCGATGCGTTTTTACGATCGGTTAGGAAGAATATAATAATTAG

AATTTTTTACGGTAAAGTTTTATTGTTTATATTTTTATGGGGTTAGAGTGTAAGAAGTAA

GAGCGAGAGTAAGAGAGAGAGAAAAACGAAATTTTTTTTATTTTAAAGAGAATAATTATT

GTTTAGGGCGTATTATTTTTTGATTGGTTGTAGTTTATGGTCGAGTTGACGTTTACGGGA

AAAATAGAGTATAAGTAGTCGTAAATATTTTTGGTTTATGCGTAGATTATTTGTTTATTA

ATTTAGAATATAGGATGTTAGCGTTATTTTGTGACGGCGAATGTGGGGGCGGTTTTTTAT

ATATATTTGTTATAGTTTAGTAAGGATGGGTTGGTAGAATAAATTTTTAGAAGAGTTGGT

TTGTTTTTTGTTTT

>74RLTR_11

ATGTGAAAAAGAATAAAAGGGTTTTTAATATTAGAAAAAATTTAAGGTTAAATATATTTA

TTGGTATTTTCGTTTTTTAGTTGAAAAGTTTTGAATTTATATAGTTGAATTTTTTTTAAT

AGTTTGTTTTACGGGAACTTTTATTATCGTCGTTTTTTAGTTGATGAGTTTTGAATTCGG

TAGTTGAATTTTTTTAATAGTTTGTGTTACGGGAATTTTTATAAACGTGATTCGTAGTTT

TGGTTTTGAAATGAAGTATTTTTTTTGCGTTAGTTCGGAGTTTTTTTTCGTTTCGGAATT

CGGTATTAATTGTTATTCGATGCGTTTTTACGATCGGTTAGGAAGAATATAATAATTAGA

ATTTTTTACGGTAAAGTTTTATTGTTTATATTTTTATGGGGTTAGAGTGTAAGAAGTAAG

AGCGAGAGTAAGAGAGAGAGAAAAACGAAATTTTTTTTATTTTAAAGAGAATAATTATTG

TTTAGGGCGTGTTATTTTTTGATTGGTTGTAGTTTATGGTCGAGTTGACGTTTATGGGAA

AAATAGAGTATAAGTAGTTGTAAATATTTTTGGTTTATGCGTAGATTATTTGTTTATTAA

TTTAGAATATAGGATGTTAGCGTTATTTTGTGACGGCGAATGTGGGGGCGGTTTTTTATA

TATATTTGTTATAGTTTAGTAAGGATGGGTTGGTAGAATAAATTTTTAGAAGAGTTGGTT

TGTTTTTTGTTTTTG

>74RLTR_12

ATGTGAAAAAGATAAAAGGGTTTTTAATGCTAGAAAAAATTTGAGGTTAAATATATTTAT

TGGTATTTTCGTTTTTTAGTTGAAAAGTTTTGAATTTATATAGTTGAATTTTTTTTAATA

GTTTGTTTTACGGGAATTTTTATTATCGTCGTTTTTTAGTTGATGAGTTTTGAATTCGGT

AGTTGAATTTTTTTTAATAGTTTGTGTTACGGGAATTTTTATAAACGTGATTCGTAGTTT

TGGTTTTGAAATGAAGTATCTTTTTTGCGTTAGTTCGGAGTTTTTTTTCGTTTCGGAATT

CGGTATTAATTGTTATTCGATGCGTTTTTACGATCGGTTAGGAAGAATATAATAATTAGA

ATTTTTTACGGTAAAGTTTTATTGTTTATATTTTTATGGGGTTAGAGCGTAAGAAGCAAG

AGCGAGAGTAAGAGAGAGAGAAAAACGAAATTTTTTTTATTTTAAAGAGAATAATTATTG

TTTAGGGCGTATTATTTTTTGATTGGTTGTAGTTTATGGTCGAGTTGACGTTTACGGGAA

AAACAGAGTATAAGTAGTCGTAAATATTTTTGGTTTATGCGTAGATTATTTGTTTATTAA

TTTAGAATATAGGATGTTAGCGTTATTTTGTGACGGCGAATGTGGGGGCGGTTTTTTATA

TATATTTGTTATAGTTTAGTAAGGATGGGTTGGTAGAATAAATTTTTAGAAGAGTTGGTT

TGTTTTTTGTTTTTG

>74RLTR_13

ATGTGAAAAAGAATAAAAGGGTTTTTAATACTAGAAAAAATTTAAGGTTAAATATATTTA

TTGGTATTTTCGTTTTTTAGTTGAAAAGTTTTGAATTTATATAGTTGAATTTTTTTTAAT

AGTTTGTTTTACGGGAATTTTTATTATCGTCGTTTTTTAGTTGATGAGTTTTGAATTCGG

TAGTTGAATTTTTTTTAATAGTTTGTGTTACGGGAATTTTTATAAACGTGATTCGTAGTT

TTGGTTTTGAAATGAAGTATCTTTTTTGCGTTAGTTCGGAGTTTTTTTTCGTTTCGGAAT

TCGGTATTAATTGTTATTCGATGCGTTTTTACGATCGGTTAGGAAGAATATAATAATTAG

AATTTTTTACGGTAAAGTTTTATTGTTTATATTTTCATGGGGTTAGAGTGTAAGAAGCAA

GAGCGAGAGTAAGAGAGAGAGAAAAACGAAATTTTTTTTATTTTAAAGAGAATAATTATT

GTTTAGGGCGTATTATTTTTTGATTGGTTGTAGTTTATGGTCGAGTTGACGTTTACGGGA

AAAACAGAGTATAAGTAGTCGTAAATATTTTTGGTTTATGCGTAGATTATTTGTTTATTA

ATTTAGAATATAGGATGTTAGCGTTATTTTGTGACGGCGAATGTGGGGGCGGTTTTTCAT

ATATATTTGTTATAGTTTAGTAAGGATGGGTTGGTGGAATAAATTTTTAGAAGAGTTGGT

TTGTTTTTTGTT

>74RLTR_14

ATGTGAAAAAGAATAAAAGGGTTTTTAATATTAGAAAAAATTTAAGGTTAAATATATTTA

TTGGTATTTTCGTTTTTTAGTTGAAAAGTTTTGAATTTATATAGTTGAATTTTTTTTAAT

AGTTTGTTTTACGGGAATTTCTATTATCGTCGTTTTTTAGTTGATGAGTTTTGAATTCGG

TAGTTGAATTTTTTTTAATAGTTTGTGTTACGGGAATTTTTATAAACGTGATTCGTAGTT

TTGGTTTTGAAATGAAGTATTTTTTTTGCGTTAGTTCGGAGTTTTTTTTCGTTTCGGAAT

TCGGTATTAATTGTTATTCGATGCGTTTTTACGATCGGTTAGGAAGAATATAATAATTAG

AATTTTTTACGGTAAAGTTTTATTGTTTATATTTTTATGGGGTTAGAGTGTAAGAAGTAA

GAGCGAGAGTAAGAGAGAGAGAAAAACGAAATTTTTTTTATTTTAAAGAGAATAATTATT

GTTTAGGGCGTATTATTTTTTGATTGGTTGTAGTTTATGGTCGAGTTGACGTTTACGGGA

AAAATAGAGTATAAGTAGTCGTAAATATTTTTGGTTTATGCGTAGATTAGTTGTTTATTA

ATTTAGAATATAGGATGTTAGCGTTATTTTGTGACGGCGAATGTGGGGGCGGTTTTTTAT

ATATATTTGTTATAGTTTAGTAAGGATGGGTTGGTAGAATAAATTTTTAGAAGAGTTGGT

TTGTTTTTTGTTTTTG

>74RLTR_15

ATGTGAAAAAGAATAAAAGGGTTTTTAATACTAGAAAAAATTTAAGGTTAAATATATTTA

TTGGTATTTTCGTTTTTTAGTTGAAAAGTTTTGAATTTATATAGTTGAATTTTTTTTAAT

AGTTTGTTTTACGGGAATTTTTATGATCGTCGTTTTTTAGTCGATGAGTTTTGAATTCGG

TAGTTGAATTTTTTTTAATAGTTTGTGTTACGGGAATTTTTATAAACGTGATTCGTAGTT

TTGGTTTTGAAATGAAGTATCTTTTTTGCGTTAGTTCGGAGTTTTTTTTCGTTTCGGAAT

TCGGTATTAATTGTTATTCGATGCGTTTTTACGATCGGTTAGGAAGAATATAATAATTAG

AATTTTTTACGGTAAAGTTTTATTGTTTATATTTTTATGGGGTTAGAGTGTAAGAAGCAA

GAGCGAGAGTAAGAGAGAGAGAAAAACGAAATTTTTTTTATTTTAAAGAGAATAATTATT

GTTTAGGGCGTGTTATTTTTTGATTGGTTGTAGTTTATGGTCGAGTTGACGTTTACGGGA

AAAATAGAGTATAAGTAGTCGTAAATATTTTTGGTTTATGCGTAGATTATTTGTTTATTA

ATTTAGAATATAGGATGTTAGCGTTATTTTGTGACGGCGAATGTGGGGGCGGTTTTTTAT

ATATATTTGTTATAGTTTAGTAAGGATGGGTTGGTAGAATAAATTTTTAGAAGAGTTGGT

TTGTTT

>74RLTR_16

ATGTGAAAAAGAATAAAAGGGTTTTTAATATTAGAAAAAATTTAAGGTTAAATATATTTA

TTGGTATTTTCGTTTTTTAGTTGAAAAGTTTTGAATTTATATAGTTGAATTTTTTTTAAT

AGTTTGTTTTACGGGAATTTTTATTATCGTCGTTTTTTAGTTGATGAGTTTTGAATTCGG

TAGTTGAATTTTTTTTAATAGTTTGTGTTACGGGAATTTTTATAAACGTGATTCGTAGTT

TTGGTTTTGAAATGAAGTATTTTTTTTGCGTTAGTTCGGAGTTTTTTTTCGTTTCGGAAT

TCGGTATTAATTGTTATTCGATGCGTTTTTACGATCGGTTAGGAAGAATATAATAATTAG

AATTTTTTACGGTAAAGTTTTATTGTTTATATTTTTATGGGGTTAGAGTGTAAGAAGTAA

GAGCGAGAGTAAGAGAGAGAGGAAAACGAAATTTTTTTTATTTTAAAGAGAATAATTATT

GTTTAGGGCGTATTATTTTTTGATTGGTTGTAGTTTATGGTCGAGTTGACGTTTACGGGA

AAAATAGAGTATAAGTAGTCGTAAATATTTTTGGTTTATGCGTAGATTATTTGTTTATTA

ATTTAGAATATAGGATGTTAGCGTTATTTTGTGACGGCGAATGTGGGGGCGGTTTTTTAT

ATATATTTGTTATAGTTTAGTAAGGATGGGTTGGTAGAATAAATTTTTAGAAGAGTTGGT

TTGTTTTTTGTTTTTG

>74RLTR_17

ATGTGAAAAAGAATAAAAGGGTTTTTAATATTAGAAAAAATTTAAGGTTAAATATATTTA

TTGGTATTTTCGTTTTTTAGTTGAAAAGTTTTGAATTTATATAGTTGAATTTTTTTTAAT

AGTTTGTTTTACGGGAATTTTTATGATCGTCGTTTTTTAGTTGATGAGTTTTGAATTCGG

TAGTTGAATTTTTCTTAATAGTTTGTGTTACGGGAATTTTTATAAACGTGATTCGTAGTT

TTGGTTTTGAAATGAAGTATTTTTTTTGCGTTAGTTCGGAGTTTTTTTTCGTTTCGGAAT

TCGGTATTAATTGTTATTCGATGCGTTTTTACGATCGGTTAGGAAGAATATAATAATTAG

AATTTTTTACGGTAAAGTTTTATTGTTTATATTTTTATGGGGTTAGAGTGTAAGAAGTAA

GAGCGAGAGTAAGAGAGAGAGAAAAACGAAATTTTTTTTATTTTAAAGAGAATAATTATT

GTTTAGGGCGTATTATTTTTTGATTGGTTGTAGTTTATGGTTGAGTTGATGTTTATGGGA

AAAATAGAGTACAAGTAGTTGTAAATATTTTTGGTTTATGTGTAGATTATTTGTTTATCA

ATTTAGAATATAGGATGTTAGTGTTATTTTGTGATGGCGAATGTGGGGGCGGTTTTTTAT

ATATATTTGTTATAGTTTAGTAAGGATGGGTTGGTAGAATAAATTTTTAGAAGAGTTGTT

TGTTTTTTGTTTTTG

>74RLTR_18

GAAAAGAATAAAAGGGTTTTTAATACTAGAAAAAATTTAAGGTTAAATATATTTATTGGT

ATTTTCGTTTTTTAGTTGAAAAGTTTTGAATTTATATAGTTGAATTTTTTTTAATAGTTT

GTTTTACGGGAATTTTTATTATCGTCGTTTTTTAGCTGATGAGTTTTGAATTCGGTAGTT

GAATTTTTTTTAATAGTTTGTGTTACGGGAATTTTTATAAACGTGATTCGTAGTTTTGGT

TTTGAAATGAAGTATCTTTTTTGCGTTAGTTCGGAGTTTTTTTTCGTTTCGGAATTCGGT

ATTAATTGTTATTCGATGCGTTTTTACGATCGGTTAGGAAGAGTATAATAATTAGAATTT

TTTACGGTAAAGTTTTATTGTTTATATTTTTATGGGGTTAGAGTGTAAGAAGCAAGAGCG

AGAGTAAGAGAGAGAGAAAAACGAAATTTTTTTTATTTTAAAGAGAATAATTATTGTTTA

GGGCGTATTATTTTTTGATTGGTTGTAGTTTATGGTCGAGTTGACGTTTACGGGAAAAAC

AGAGTATAAGTAGTCGTAAATATTTTTGGTTTATGCGTAGATTATTTGTTTATTAATTTA

GAATATAGGATGTTAGCGTTATTTTGTGACGGCGAATGTGGGGGCGGTTTTTCATATATA

TTTGTTATAGTTTAGTAAGGATGGGTTGGTAGAATAAATTTTTAGAAGAGTTGGTTTGTT

TTTTGTTTTTG

>74RLTR_19

ATGTGAAAAAGAATAAAAGGGTTTTTAATACTAGAAAAAATTTAAGGTTAGATATATTTA

TTGGTATTTTCGTTTTTTAGTTGAAAAGTTTTGAATTTATATAGTTGAATTTTTTTTAAT

AGTTTGTTTTACGGGAATTTTTATTATCGTCGTTTTTTAGTTGATGAGTTTTGAATTCGG

TAGTTGAATTTTTTTTAATAGTTTGTGTTACGGGAATTTTTATAAACGTGATTCGTAGTT

CTGGTTTTGAAATGAAGTATCTTTTTTGCGTTAGTTCGGAGTTTTTTTTCGTTTCGGAAT

TCGGTATTAATTGTTATTCGATGCGTTTTTACGATCGGTTAGGAAGAATATAATAATTAG

AATTTTTTACGGTAAAGTTTTATTGTTTATATTTTTATGGGGTTAGAGTGTAAGAAGCAA

GAGCGAGAGTAAGAGAGAGAGAAAAACGAAATTTTTTTTATTTTAAAGAGAATAATTATT

GTTTAGGGCGTATTATTTTTTGATTGGTTGTAGTTTATGGTCGAGTTGACGTTTACGGGA

AAAACAGAGTATAAGTAGTCGTAAATATTTTTGGTTTATGCGTAGATTATTTGTTTATTA

ATTTAGAATATAGGATGTTAGCGTTATTTTGTGACGGCGAATGTGGGGGCGGTTTTTCAT

ATATATTTGTTATAGTTTAGTAAGGATGGGTTGGTAGAATAAATTTTTAGAAGAGTTGGT

TTGTTTTTTGTTT

>74RLTR_20

ATGTGAAAAAGAATAAAAGGGTTTTTAATATTAGAAAAAATTTAAGGTTAAATATATTTA

TTGGTATTTTCGTTTTTTAGTTGAAAAGTTTTGAATTTATATAGTTGAATTTTTTTTAAT

AGTTTGTTTTACGGGAACTTTTATTATCGTCGTTTTTTAGTTGATGAGTTTTGAATTCGG

TAGTTGAATTTTTTTTAATAGTTTGTGTTACGGGAATTTTTATAAACGTGATTCGTAGTT

TTGGTTTTGAAATGAAGTATTTTTTTTGCGTTAGTTCGGAGTTTTTTTTCGTTTCGGAAT

TCGGTATTAATTGTTATTCGATGCGTTTTTACGATCGGTTAGGAAGAATATAATAATTAG

AATTTTTTACGGTAAAGTTTTATTGTTTATATTTTTATGGGGTTAGAGTGTAAGAAGTAA

GAGCGAGAGTAAGAGAGAGAGAAAAACGAAATTTTTTTTATTTTAAAGAGAATAATTATT

GTTTAGGGCGTGTTATTTTTTGATTGGTTGTAGTTTATGGTCGAGTTGACGTTTATGGGA

AAAATAGAGTATAAGTAGTTGTAAATATTTTTGGTTTATGCGTAGATTATTTGTTTATTA

ATTTAGAATATAGGATGTTAGCGTTATTTTGTGACGGCGAATGTGGGGGCGGTTTTTTAT

ATATATTTGTTATAGTTTAGTAAGGATGGGTTGGTAGAATAAATTTTTAGAAGAGTTGGT

TTGTTTTTTGTTT

>74RLTR_23

ATGTGAAAAAGAATAAAAGGGTTTTTAATACTAGAAAAAATTTAAGGTTAAATATATTTA

TTGGTATTTTCGTTTTTTAGTTGAAAAGTTTTGAATTTATATAGTTGAATTTTTTTTAAT

AGTTTGTTTTACGGGAATTTTTATTATCGTCGTTTTTTAGTTGATGAGTTTTGAATTCGG

TAGTTGAATTTTTTTTAATAGTTTGTGTTACGGGAATTTTTATAAACGTGATTCGTAGTT

TTGGTTTTGAAATGAAGTATCTTTTTTGCGTTAGTTCGGAGTTTTTTTTCGTTTCGGAAT

TCGGTATTAATTGTTATTCGATGCGTTTTTACGATCGGTTAGGAAGAATATAATAATTAG

AATTTTTTACGGTAAAGTTTTATTGTTTATATTTTTATGGGGTTAGAGTGTAAGAAGCAA

GAGCGAGAGTAAGAGAGAGAGAAAAACGAAATTTTTTTTATTTTAAAGAGAATAATTATT

GTTTAGGGCGTATTATTTTTTGATTGGTTGTAGTTTATGGTCGAGTTGACGTTTACGGGA

AAAACAGAGTATAAGTAGTCGTAAATATTTTTGGTTTATGCGTAGATTATTTGTTTATTA

ATTTAGAATATAGGATGTTAGCGTTATTTTGTGACGGCGAATGTGGGGGCGGTTTTTCAT

ATATATTTGTTATAGTTTAGTAAGGATGGGTTGGTAGAATAAATTTTTAGAAGAGTTGGT

TTGTTTTTTGTTTTTG

>74RLTR_24

ATGTGAAAAAGAATAAAAGGGTTTTTAATACTAGAAAAAATTTAAGGTTAAATATATTTA

TTGGTATTTTCGTTTGTTAGTTGAAAAGTTTTGAATTTATATAGTTGAATTTTTTTTAAT

AGTTTGTTTTACGGGAATTTTTATTATCGTCGTTTTTTAGTTGATGAGTTTTGAATTCGG

TAGTTGAATTTTTTTTAATAGTTTGTGTTACGGGAATTTTTATAAACGTGATTCGTAGTT

TTGGTTTTGAAATGAAGTATTTTTTTTGCGTTAGTTCGGAGTTTTTTTTCGTTTCGGAAT

TCGGTATTAATTGTTATTCGATGCGTTTTTACGATCGGTTAGGAAGAATATAATAATTAG

AATTTTTTACGGTAAAGTTTTATTGTTTATATTTTTATGGGGTTAGAGTGTAAGAAGTAA

GAGCGAGAGTAAGAGAGAGAAAAAAACGAAATTTTTTTTATTTTAAAGAGAATAATTATT

GTTTAGGGCGTATTATTTTTTGATTGGTTGTAGTTTATGGTCGAGTTGACGTTTACGGGA

AAAATAGGGTATAAGTAGTCGTAAATATTTTTGGTTTATGCGTAGATTATTTGTTTATTA

ATTTAGAATATAGGATGTTAGCGTTATTTTGTGACGGCGAATGTGGGGGCGGTTTTTTAT

ATATATTTGTTATAGTTTAGTAAGGATGGGTTGGTAGAATAAATTTTTAGAAGAGTTGGT

TTGTTTTTTGTTTTTG

>74RLTR_25

ATGTGAAAAAGAATAAAAGGGTTTTTAATATTAGAAAAAATTTAAGGTTAAATATATTTA

TTGGTATTTTCGTTTTTTAGTTGAAAAGTTTTGAATTTATATAGTTGAGTTTTTTTTAAT

AGTTTGTTTTACGGGAATTTTTATTATCGTCGTTTTTTAGTTGATGAGTTTTGAATTCGG

TAGTTGAATTTTTTTTAATAGTTTGTGTTACGGGAATTTTTATAAACGTGATTCGTAGTT

TTGGTTTTGGAATGAAGTATTTTTTTTGCGTTAGTTCGGAGTTTTTTTTCGTTTCGGAAT

TCGGTATTAATTGTTATTCGATGCGTTTTTACGATCGGTTAGGAAGAATATAATAATTAG

AATTTTTTACGGTAAAGTTTTATTGTTTATATTTTTATGGGGTTAGAGTGTAAGAAGTAA

GAGCGAGAGTAAGAGAGAGAGAAAAACGAAATTTTTTTTATTTTAAAGAGAATAATTATT

GTTTAGGGCGTGTTATTTTTTGATTGGTTGTAGTTTGTGGTCGAGTTGACGTTTATGGGA

AAAATAGAGTATAAGTAGTTGTAAATATTTTTGGTTTATGCGTAGATTATTTGTTTATTA

ATTTAGAATATAGGATGTTAGCGTTATTTTGTGACGGCGAATGTGGGGGCGGTTTTTTAT

ATATATTTGTTATAGTTTAGTAAGGATGGGTTGGTAGAATAAATTTTTAGAAGAGTTGGT

TTGTTTTTTGTTTTTG

>74RLTR_73

ATGTGAAAAGAATAAAAGGGTTTTTAATACTAGAAAAAATTTAAGGTTAAATATATTTAT

TGGTATTTTCGTTTTTTAGTTGAAAAGTTTTGAATTTATATAGTTGAATTTTTTTTAATA

GTTTGTTTTACGGGAATTTTTATTATCGTCGTTTTTTAGTTGATGAGTTTTGAATTCGGT

AGTTGAATTTTTTTTAATAGTTTGTGTTACGGGAATTTTTATAAACGTGATTCGTAGTTT

TGGTTTTGAAATGAAGTATCTTTTTTGCGTTAGTTCGGAGTTTTTTTTCGTTTCGGAATT

CGGTATTAATTGTTATTCGATGCGTTTTTACGATCGGTTAGGAAGAATATAATAATTAGA

ATTTTTTACGGTAAAGTTTTATTGTTTATATTTTTATGGGGTTAGAGTGTAAGAAGCAAG

AGCGAGAGTAAGAGAGAGAGAAAAACGAAATTTTTTTTATTTTAAAGAGAATAATTATTG

TTTAGGGCGTATTATTTTTTGATTGGTTGTAGTTTATGGTCGAGTTGACGTTTACGGGAA

AAACAGAGTATAAGTAGTCGTAAATATTTTTGGTTTATGCGTAGATTATTTGTTTATTAA

TTTAGAATATAGGATGTTAGCGTTATTTTGTGACGGCGAATGTGGGGGCGGTTTTTCATA

TATATTTGTTATAGTTTAGTAAGGATGGGTTGGTAGAATAAATTTTTAGAAGAGTTGGTT

TGTTTTTTGTTTTTG

>74RLTR_74

ATGTGAAAAAGAATAAAAGGGTTTTTAATACTAGAAAAAATTTAAGGTTAAATATATTTA

TTGGTATTTTCGTTTTTTAGTTGAAAAGTTTTGAATTTATATAGTTGAATTTTTTTTAAT

AGTTTGTTTTACGGGAATTTTTATTATCGTCGTTTTTTAGTTGATGAGTTTTGAATTCGG

TAGTTGAATTTTTTTTAATAGTTTGTGTTACGGGAATTTTTATAAACGTGATTCGTAGTT

TTGGTTTTGAAATGAAGTATCTTTTTCGCGTTAGTTCGGAGTTTTTTTTCGTTTCGGAAT

TCGGTATTAATTGTTATTCGATGCGTTTTTACGATCGGTTAGGAAGAATATAATAATTAG

AATTTTTTACGGTAAAGTTTTATTGTTTATATTTTTATGGGGTTAGAGTGTAAGAAGCAA

GAGCGAGAGTAAGAGAGAGAGAAAAACGAAATTTTTTTTATTTTAAAGAGAATAATTATT

GTTTAGGGCGTATTATTTTTTGATTGGTTGTAGTTTATGGTCGAGTTGACGTTTACGGGA

AAAACAGAGTATAAGTAGTCGTAAATATTTTTGGTTTATGCGTAGATTATTTGTTTATTA

ATTTAGAATATAGGATGTTAGCGTTATTTTGTGACGGCGAATGTGGGGGCGGTTTTTCAT

ATATATTTGTTATAGTTTAGTAAGGATGGGTTGGTAGAATAAATTTTTAGAAGAGTTGGT

TTGTTTTTTGTTTTTG
