# Supplementary material for: Epigenetic interplay between mouse endogenous retroviruses and host genes
Source: Genome Biol. 2012 Oct 3;13(10):R89. doi: 10.1186/gb-2012-13-10-r89 (PMC3491417; doi:10.1186/gb-2012-13-10-r89)
Supplement: Additional file 4 — All bisulfite sequencing data. Compilation of all bisulfite sequences. [file gb-2012-13-10-r89-S4.zip › IAP6428_TE_brain.doc]

6 11 10 CdGAP 3’ LTR B6129 Brain Miniprep Sequences

>GAP3LTRBr_52

GGGAAGGAAGTTTTGTTTATATTATAAGTGTGTTTTTGTAAAATAAGGAGTGTTATATGT

TAAGATATGGTGGTTGGTTTTTAAGATAAGTTTTTTAGTTTGGAGGTATTTTTGTTTGTT

TGTTATTTGTTGTGTTTTTATGATTGGTTAGGAAGAATATAATAATTAGAATTTTTTATG

GTAAAGTTTTATTGTTTATATTTTTTTGGGGTTAGAGTGTAAGAAGTAAGAGAGAGAGAA

AAATGAAATTTTTTTTATTTAAAAGAGAATAATAATTGTTTAGGATGTATTATTTTTTGA

TTGGTTGTAGTTTATGGTTGAGTTGATGTTTATGGGAAAAATAGAGTATAAGTAGTTGTA

AATATTTTTGGTTTATGTGTAGATTATTTGTTTATTAATTTAGAATATAGGATGTTAGTG

TTATTTTGTGATGGTGAATGTGGGGGTGGTTTTTTATAGTTTTTTTTTTTTTTTTTAATA

AGAGTAAATAGGTTATTTATATTAATGAGAGTGGAGATAGAGGTTAAATTTTTAGTGTGT

AGGTAAAGGAGTTATGTATAGGATTAGTTTTTAGGTTTATAGGTTTTTATTTAGAGTAAT

TTTGATTTGTTTTTGTGTTGTTTTGTTTGGGGGAAGGGAATTAGGATATTGAATTTTTAT

GAAAGATGATATGTTTTTTTAGAATAGGTTTATATATGTCGTAGAGTTTTTTTATTGTAG

TGTTTAGTTTTGTAATT

>GAP3LTRBr_54

GGGAAGGAAGTTTTGTTTATATTATAAGTGTGTTTTTGTAAAATAAGGAGTGTTATATGT

TAAGATATGGTGGTTGGTTTTTAAGATAAGTTTTTTAGTTTGGAGGTATTTTTGTTTGTT

TGTTATTTGTTGTGTTTTTATGATTGGTTAGGAAGAATATAATAATTAGAATTTTTTATG

GTAAAGTTTTATTGTTTATATTTTTTTGGGGTTAGAGTGTAAGAAGTAAGAGAGAGAGAA

AAATGAAATTTTTTTTATTTAAAAGAGAATAATAATTGTTTAGGATGTATTATTTTTTGA

TTGGTTGTAGTTTATGGTTGAGTTGATGTTTATGGGAAAAATAGAGTATAAGTAGTTGTA

AATATTTTTGGTTTATGTGTAGATTATTTGTTTATTAATTTAGAATATAGGATGTTAGTG

TTATTTTGTGATGGTGAATGTGGGGGTGGTTTTTTATAGTTTTTTTTTTTTTTTTTAATA

AGAGTAAATAGGTTATTTATATTAATGAGAGTGGAGATAGAGGTTAAATTTTTAGTGTGT

AGGTAAAGGAGTTATGTATAGGATTAGTTTTTAGGTTTATAGGTTTTTATTTAGAGTAAT

TTTGATTTGTTTTCGTGTCGTTTTGTTTGGGGGAAGGGAATTAGGATATTGAATTTTTAT

GAAAGATGACATGTTTTTTTAGAATAGGTTTATATATGTCGTAGAGTTTTTTTATTGTAG

TGTTTAGTTTTGTAATT

>GAP3LTRBr_55

GGGAAGGAAGTTTTGTTTATATTATAAGTGTGTTTTTGTAAAATAAGGAGTGTTATATGT

TAAGATATGGTGGTTGGTTTTTAAGATAAGTTTTTAGTTTGGAGGTATTTTTGTTTGTTT

GTTATTCGTCGTGTTTTTATGATCGGTTAGGAAGAATATAATAATTAGAATTTTTTATGG

TAAAGTCTTATTGTTTATATTTTTTTGGGGTTAGAGTGTAAGAAGTAAGAGAGAGAGAAA

AATGAAATTTTTTTTATTTAAAAGAGAATAATAATTGTTTAGGATGTATTATTTTTTGAT

TGGTTGTAGTTTATGGTTGAGTTGATGTTTACGGGAAAAATAGAGTATAAGTAGTCGTAA

ATATTTTTGGTTTATGTGTAGATTATTTGTTTATTAATTTAGAATATAGGATGTTAGCGT

TATTTTGTGACGGCGAATGTGGGGGCGGTTTTTTATAGTTTTTTTTTTTTTTTTTAATAA

GAGTAAATAGGTTATTTATATTAATGAGAGTGGAGATAGAGGTTAAATTTTTAGTGTGTA

GGTAAAGGAGTTATGTATAGGATTAGTTTTTAGGTTTATAGGTTTTTATCTAGAGTAATT

TTGATTTGTTTTCGTGTCGTTTTGTTTGGGGGAAGGGAATTAGGATATTGAATTTTTATG

AAAGATGATATGTTTTTTTAGAATAGGTTTATATATGTCGTAGAGTTTTTTTATTGTAGT

GTTTAGTTTTGTAATT

>GAP3LTRBr_56

GGGAAGGAAGTTTTGTTTATATTATAAGTGTGTTTTTGTAAAATAAGGAGTGTTATATGT

TAAGATATGGTGGTTGGTTTTTAAGATAAGTTTTTTAGTTTGGAGGTATTTTTGTTTGTT

TGTTATTTGTTGTGTTTTTATGATTGGTTAGGAAGAATATAATAATTAGAATTTTTTATG

GTAAAGTTTTATTGTTTATATTTTTTTGGGGTTAGAGTGTAAGAAGTAAGAGAGAGAGAA

AAATGAAATTTTTTTTATTTAAAAGAGAATAATAATTGTTTAGGATGTATTATTTTTTGA

TTGGTTGTAGTTTATGGTTGAGTTGATGTTTATGGGAAAAATAGAGTATAAGTAGTTGTA

AATATTTTTGGTTTATGTGTAGATTATTTGTTTATTAATTTAGAATATAGGATGTTAGTG

TTATTTTGTGATGGTGAATGTGGGGGTGGTTTTTTATAGTTTTTTTTTTTTTTAATAAGA

GTAAATAGGTTATTTATATTAATGAGAGTGGAGATAGAGGTTAAATTTTTAGTGTGTAGG

TAAAGGAGTTATGTATAGGATTAGTTTTTAGGTTTATAGGTTTTTATTTAGAGTAATTTT

GATTTGTTTTCGTGTCGTTTTGTTTGGGGGAAGGGAATTAGGATATTGAATTTTTATGAA

AGATGATATGTTTTTTTAGAATAGGTTTATATATGTCGTAGAGTTTTTTTATTGTAGTGT

TTAGTTTTGTAATT

>GAP3LTRBr_57

GGGAAGGAAGTTTTGTTTATATTATAAGTGTGTTTTTGTAAAATAAGGAGTGTTATATGT

TAAGATATGGTGGTTGGTTTTTAAGATAAGTTTTTTAGTTTGGAGGTATTTTTGTTTGTT

TGTTATTTGTTGTGTTTTTATGATTGGTTAGGAAGAATATAATAATTAGAATTTTTTATG

GTAAAGTTTTATTGTTTATATTTTTTTGGGGTTAGAGTGTAAGAAGTAAGAGAGAGAAAA

ATGAAATTTTTTTTATTTAAAAGAGAATAATAATTGTTTAGGATGTATTATTTTTTGATT

GGTTATAGTTTATGGTTGAGTTGATGTTTATGGGAAAAATAGAGTATAAGTAGTTGTAAA

TATTTTTGGTTTATGTGTAGATTATTTGTTTATTAATTTAGAATATAGGATGTTAGTGTT

ATTTTGTGATGGTGAATGTGGGGGTGGTTTTTTATAGTTTTTTTTTTTTTTTAATAAGAG

TAAATAGGTTATTTATATTAATGAGAGTGGAGATAGAGGTTAAATTTTTAGTGTGTAGGT

AAAGGAGTTATGTATAGGATTAGTTTTTAGGTTTATAGGTTTTTATTTAGAGTAATTTTG

ATTTGTTTTCGTGTCGTTTTGTTTGGGGGAAGGGAATTAGGATATTGAATTTTTATGAAA

GATGATATGTTTTTTTAGAATAGGTTTATATATGTCGTAGAGTTTTTTTATTGTAGTGTT

TAGTTTTGTAATT

>GAP3LTRBr_58

GGGAAGGAAGTTTTGTTTATATTATAAGTGTGTTTTTGTAAAATAAGGAGTGTTATATGT

TAAGATATGGTGGTTGGTTTTTAAGATAAGTTTTTTAGTTTGGAGGTATTTTTGTTTGTT

TGTTATTTGTTGTGTTTTTATGATTGGTTAGGAAGAATATAATAATTAGAATTTTTTATG

GTAAAGTTTTATTGTTTATATTTTTTTGGGGTTAGAGTGTAAGAAGTAAGAGAGAGAGAA

AAATGAAATTTTTTTTATTTAAAAGAGAATAATAATTGTTTAGGATGTATTATTTTTTGA

TTGGTTGTAGTTTATGGTTGAGTTGATGTTTATGGGAAAAATAGAGTATAAGTAGTTGTA

AATATTTTTGGTTTATGTGTAGATTATTTGTTTATTAATTTAGAATATAGGATGTTAGTG

TTATTTTGTGATGGTGAATGTGGGGGTGGTTTTTTATAGTTTTTTTTTTTTTTTAATAAG

AGTAAATAGGTTATTTATATTAATGAGAGTGGAGATAGAGGTTAAATTTTTAGTGTGTAG

GTAAAGGAGTTATGTATAGGATTAGTTTTTAGGTTTATAGGTTTTTATTTAGAGTAATTT

TGATTTGTTTTCGTGTCGTTTTGTTTGGGGGAAGGGAATTAGGATATTGAATTTTTATGA

AAGATGATATGTTTTTTTAGAATAGGTTTATATATGTCGTAGAGTTTTTTTATTGTAGTG

TTTAGTTTTGTAATT

>GAP3LTRBr_60

GGGAAGGAAGTTTTGTTTATATTATAAGTGCGTTTTCGTAAAATAAGGAGTGTTATACGT

TAAGATACGGCGGTTGGTTTTTAAGATAAGTTTTTTAGTTTGGAGGTATTTTTGTTTGTT

TGTTATTCGTCGCGTTTTTATGATCGGTTAGGAAGAATATAATAATTAGAATTTTTTACG

GTAAAGTTTTATTGTTTATATTTTTTTGGGGTTAGAGTGTAAGAAGTAAGAGAGAGAGAA

AAACGAAATTTTTTTTATTTAAAAGAGAATAATAATTGTTTAGGATGTATTATTTTTTGA

TTGGTTGTAGTTTATGGTCGAGTTGACGTTTATGGGAAAAATAGAGTATAAGTAGTTGTA

AATATTTTTGGTTTATGTGTAGATTATTTGTTTATTAATTTAGAATATAGGATGTTAGTG

TTATTTTGTGATGGTGAATGTGGGGGTGGCTTTTTATAGTTTTTTTTTTTTTTTTTTAAT

AAGAGTAAATAGGTTATTTATATTAATGAGAGTGGAGATAGAGGTTAAATTCTTAGTGTG

TAGGTAAAGGAGTTATGTATAGGATTAGTTTTTAGGTTTATAGGTTTTTATTTAGAGTAA

TTTTGATTTGTTTTTGTGTTGTTTTGTTTGGGGGAAGGGAATTAGGATATTGAATTTTTA

TGAAAGATGATATGTTTTTTTAGAATAGGTTTATATATGTCGTAGAGTTTTTTTATTGTA

GTGTTTAGTTTTGTAATT

>GAP3LTRBr_62

GGGAAGGAAGTTTTGTTTATATTATAAGTGCGTTTTCGTAAAATAAGGAGTGTTATACGT

TAAGATACGGCGGTTGGTTTTTAAGATAAGTTTTTTAGTTTGGAGGTATTTTTGTTTGTT

TGTTATTCGTCGCGTTTTTACGATCGGTTAGGAAGAATATAATAATTAGAATTTTTTACG

GTAAAGTTTTATTGTTTATATTTTTTTGGGGTTAGAGTGTAAGAAGTAAGAGAGAGAGAA

AAACGAAATTTTTTTTATTTAAAAGAGAATAATAATTGTTTAGGACGTATTATTTTTTGA

TTGGTTGCAGTTTATGGTCGAGTTGATGTTTATGGGAAAAATAGAGTATAAGTAGTTGTA

AATATTTTTGGTTTATGCGTAGATTATTTGTTTATTAATTTAGAATATAGGATGTTAGCG

TTATTTTGTGACGGTGAATGTGGGGGCGGTTTTTTATAGTTTTTTTTTTTTTTTTTAATA

AGAGTAAATAGGTTATTTATATTAATGAGAGTGGAGATAGAGGTTAAATTTTTAGTGTGT

AGGTAAAGGAGTTATGTATAGGATTAGTTTTTAGGTTTATAGGTTTTTATTTAGAGTAAT

TTTGATTTGTTTTCGTGTCGTTTTGTTTGGGGGAAGGGAATTAGGATATTGAATTTTTAT

GAAAGATGATATGTTTTTTTAGAATAGGTTTATATATGTCGTAGAGTTTTTTTATTGTAG

TGTTTAGTTTTGTAATT

>GAP3LTRBr_64

GGGAAGGAAGTTTTGTTTATATTATAAGTGTGTTTTTGTAAAATAAGGAGTGTTATATGT

TAAGATATGGTGGTTGGTTTTTAAGATAAGTTTTTTAGTTTGGAGGTATTTTTGTTTGTT

TGTTATTTGTTGTGTTTTTATGATTGGTTAGGAAGAATATAATAATTAGAATTTTTTATG

GTAAAGTTTTATTGTTTATATTTTTTTGGGGTTAGAGTGTAAGAAGTAAGAGAGAGAGAA

AAATGAAATTTTTTTTATTTAAAAGAGAATAATAATTGTTTAGGATGTATTATTTTTTGA

TTGGTTGTAGTTTATGGTTGAGTTGATGTTTATGGGAAAAATAGAGTATAAGTAGTTGTA

AATATTTTTGGTTTATGTGTAGATTATTTGTTTATTAATTTAGAATATAGGATGTTAGTG

TTATTTTGTGATGGTGAATGTGGGGGTGGTTTTTTATAGTTTTTTTTTTTTTTTTAATAA

GAGTAAATAGGTTATTTATATTAATGAGAGTGGAGATAGAGGTTAAATTTTTAGTGTGTA

GGTAAAGGAGTTATGTATAGGATTAGTTTTTAGGTTTATAGGTTTTTATTTAGAGTAATT

TTGATTTGTTTTCGTGTCGTTTTGTTTGGGGGAAGGGAATTAGGATATTGAATTTTTATG

AAAGATGATATGTTTTTTTAGAATAGGTTTATATATGTCGTAGAGTTTTTTTATTGTAGT

GTTTAGTTTTGTAATT

>GAP3LTRBr_65

GGGAAGGAAGTTTTGTTTATATTATAAGTGTGTTTTTGTAAAATAAGGAGTGTTATATGT

TAAGATATGGTGGTTGGTTTTTAAGATAAGTTTTTTAGTTTGGAGGTATTTTTGTTTGTT

TGTTATTTGTTGTGTTTTTATGATTGGTTAGGAAGAATATAATAATTAGAATTTTTTATG

GTAAAGTTTTATTGTTTATATTTTTTTGGGGTTAGAGTGTAAGAAGTAAGAGAGAGAGAA

AAATGAAATTTTTTTTATTTAAAAGAGAATAATAATTGTTTAGGATGTATTATTTTTTGA

TTGGTTGTAGTTTATGGTTGAGTTGATGTTTATGGGAAAAATAGAGTATAAGTAGTTGTA

AATATTTTTGGTTTATGTGTAGATTATTTGTTTATTAATTTAGAATATAGGATGTTAGTG

TTATTTTGTGATGGTGAATGTGGGGGTGGTTTTTTATAGTTTTTTTTTTTTTTTTTAATA

AGAGTAAATAGGTTATTTATATTAATGAGAGTGGAGATAGAGGTTAAATTTTTAGTGTGT

AGGTAAAGGAGTTATGTATAGGATTAGTTTTTAGGTTTATAGGTTTTTATTTAGAGTAAT

TTTGATTTGTTTTCGTGTCGTTTTGTTTGGGGGAAGGGAATTAGGATATTGAATTTTTAT

GAAAGATGATATGTTTTTTTAGAATAGGTTTATATATGTCGTAGAGTTTTTTTATTGTAG

TGTTTAGTTTTGTAATT

>GAP3LTRBr_67

GGGAAGGAAGTTTTGTTTATATTATAAGTGTGTTTTTGTAAAATAAGGAGTGTTATATGT

TAAGATATGGTGGTTGGTTTTTAAGATAAGTTTTTTAGTTTGGAGGTATTTTTGTTTGTT

TGTTATTTGTTGTGTTTTTATGATTGGTTAGGAAGAATATAATAATTAGAATTTTTTATG

GTAAAGTTTTATTGTTTATATTTTTTTGGGGTTAGAGTGTAAGAAGTAAGAGAGAGAGAA

AAATGAAATTTTTTTTATTTAAAAGAGAATAATAATTGTTTAGGATGTATTATTTTTTGA

TTGGTTGTAGTTTATGGTTGAGTTGATGTTTATGGGAAAAATAGAGTATAAGTAGTTGTA

AATATTTTTGGTTTATGTGTAGATTATTTGTTTATTAATTTAGAATATAGGATGTTAGTG

TTATTTTGTGATGGTGAATGTGGGGGTGGTTTTTTATAGTTTTTTTTTTTTTTAATAAGA

GTAAATAGGTTATTTATATTAATGAGAGTGGAGATAGAGGTTAAATTTTTAGTGTGTAGG

TAAAGGAGTTATGTATAGGATTAGTTTTTAGGTTTATAGGTTTTTATTTAGAGTAATTTT

GATTTGTTTTTGTGTTGTTTTGTTTGGGGGAAGGGAATTAGGATATTGAATTTTTATGAA

AGATGATATGTTTTTTTAGAATAGGTTTATATATGTCGTAGAGTTTTTTTATTGTAGTGT

TTAGTTTTGTAATT

>GAP3LTRBr_69

GGGAAGGAAGTTTTGTTTATATTATAAGTGCGTTTTCGTAAAATAAGGAGTGTTATACGT

TAAGATACGGCGGTTGGTTTTTAAGATAAGTTTTTTAGTTTGGAGGTATTTTTGTTTGTT

TGTTATTCGTCGCGTTTTTACGATCGGTTAGGAAGAATATAATAATTAGAATTTTTTACG

GTAAAGTTTTATTGTTTATATTTTTTTGGGGTTAGAGTGTAAGAAGTAAGAGAGAGAGAA

AAACGAAATTTTTTTTATTTAAAAGAGAATAATAATTGTTTAGGACGTATTATTTTTTGA

TTGGTTGCAGTTTATGGTCGAGTTGATGTTTATGGGAAAAATAGAGTATAAGTAGTTGTA

AATATTTTTGGTTTATGCGTAGATTATTTGTTTATTAATTTAGAATATAGGATGTTAGCG

TTATTTTGTGACGGTGAATGTGGGGGCGGTTTTTTATAGTTTTTTTTTTTTTTTTTAATA

AGAGTAAATAGGTTATTTATATTAATGAGAGTGGAGATAGAGGCTAAATTTTTAGTGTGT

AGGTAAAGGAGTTATGTATAGGATTAGTTTTTAGGTTTATAGGTTTTTATTTAGAGTAAT

TTTGATTTGTTTTCGTGTCGTTTTGTTTGGGGGAAGGGAATTAGGATATTGAATTTTTAT

GAAAGATGATATGTTTTTTTAGAATAGGTTTATATATGTCGTAGAGTTTTTTTATTGTAG

TGTTTAGTT

>GAP3LTRBr_70

GGGAAGGAAGTTTTGTTTATATTATAAGTGCGTTTTCGTAAAATAAGGAGTGTTATACGT

TAAGATACGGCGGTTGGTTTTTAAGATAAGTTTTTTAGTTTGGAGGTATTTTTGTTTGTT

TGTTATTCGTCGCGTTTTTACGATCGGTTAGGAAGAATATAATAATTAGAATTTTTTACG

GTAAAGTTTTATTGTTTATATTTTTTTGGGGTTAGAGTGTAAGAAGTAAGAGGGAGAGAA

AAACGAAATTTTTTTTATTTAAAAGAGAATAATAATTGTTTAGGACGTATTATTTTTTGA

TTGGTTGCAGTTTATGGTCGAGTTGATGTTTATGGGAAAAATAGAGTATAAGTAGTTGTA

AATATTTTTGGTTTATGCGTAGATTATTTGTTTATTAATTTAGAATATAGGATGTTAGCG

TTATTTTGTGACGGTGAATGTGGGGGCGGTTTTTTATAGTTTTTTTTTTTTTTTAATAAG

AGTAAATAGGTTATTTATATTAATGAGAGTGGAGATAGAGGTTAAATTTTTAGTGTGTAG

GTAAAGGAGTTATGTATAGGATTAGTTTTTAGGTTTATAGGTTTTTATTTAGAGTAATTT

TGATTCGTTTTCGTGTCGTTTTGTTTGGGGGAAGGGAATTAGGATATTGAATTTTTATGA

AAGATGATATGTTTTTTTAGAATAGGTTTATATATGTCGTAGAGTTTTTTTATTGTAGTG

TTTAGTTTTGTATT

>GAP3LTRBr_72

GGGAAGGAAGTTTTGTTTATATTATAAGTGCGTTTCGTAAAATAAGGAGTGTTATACGTT

AAGATACGGCGGTTGGTTTTTAAGATAAGTTTTTTAGTTTGGAGGTATTTTTGTTTGTTT

GTTATTCGTCGCGTTTTTACGATCGGTTAGGAAGAATATAATAATTAGAATTTTTTACGG

TAAAGTTTTATTGTTTATATTTTTTTGGGGTTAGAGTGCAAGAAGTAAGAGAGAGAGAAA

AACGAAATTTTTTTTATTTAAAAGAGAATAATAATTGTTTAGGACGTATTATTTTTTGAT

TGGTTGCAGTTTATGGTCGAGTTGATGTTTATGGGAAAAATAGAGTATAAGTAGTTGTAA

ATATTTTTGGTTTATGCGTAGATTATTTGTTTATTAATTTAGAATATAGGATGTTAGCGT

TATTTTGTGACGGTGAATGTGGGGGCGGTTTTTTATAGTTTTTTTTTTTTTTTTTAATAA

GAGTAAATAGGTTATTTATATTAATGAGAGTGGAGATAGAGGTTAAATTTTTAGTGTGTA

GGTAAAGGAGTTATGTATAGGATTAGTTTTTAGGTTTATAGGTTTTTATTTAGAGTAATT

TTGATTTGTTTTCGTGTCGTTTTGTTTGGGGGAAGGGAATTAGGATATTGAATTTTTATG

AAAGATGATATGTTTTTTTAGAATAGGTTTATATATGTCGTAGAGTTTTTTTATTGTAGT

GTTTAGTTTTGTAATT
