# Supplementary material for: Epigenetic interplay between mouse endogenous retroviruses and host genes
Source: Genome Biol. 2012 Oct 3;13(10):R89. doi: 10.1186/gb-2012-13-10-r89 (PMC3491417; doi:10.1186/gb-2012-13-10-r89)
Supplement: Additional file 4 — All bisulfite sequencing data. Compilation of all bisulfite sequences. [file gb-2012-13-10-r89-S4.zip › IAP6428_TE_oppositeLTR_ES.doc]

CdGAP 5LTR

B6129 ES Cells

B6 Clones

>CRLTRES1-M13R

AAAAGAATAAAAGGGTTTTTAATATTAGAAAAAATTTAAGGTTAAATATATTTATTGGTA

TTTTCGTTTTTTAGTTGAAAAGTTTTGAATTTATACAGTTGAATTTTTTTTAATAGTTTG

TTTTACGGGAATTTTTATTATCGTCGTTTTTTAGTTGATGAGTTTTGAATTCGGTAGTTG

AATTTTTTTTAATAGTTTGTGTTACGGGAATTTTTATAAACGTGATTCGTAGTTTTGGTT

TTGAAATGAAGTATTTTTTTTGCGTTAGTTCGGAGTTTTTTTTCGTTTCGGAATTCGGTA

TTAATTGTTATTCGTCGCGTTCTTACGATCGGTTAGGAAGAATATAATAATTAGAATTTT

TTACGGTAAAGTTTTATTGTTTATATTTTTTTGGGGTTAGAGTGTAAGAAGTAAGAGAGA

GAGAAAAACGAAATTTTTTTTATTTAAAAGAGAACAATAATTGTTTAGGACGTATTATTT

TTTGATTGGTTGTAGTTTATGGTCGAGTTGACGTTTACGGGAAAAATAGAGTATAAGTAG

TCGTAAATATTTTTGGTTTATGCGTAGATTATTTGTTTATTAATTTAGAATATAGGATGT

TAGCGTTATTTTGTGACGGCGAATGTGGGGGCGGTTTTTTATATTTGTTTGTAGTATTTG

TTTGGTTTTTGTAGTTATTGGAATTTATGT

>CRLTRES3-M13R

AAAAGAATAAAAGGGTTTTTAATATTAGAAAAAATTTAAGGTTAAATATATTTATTGGTA

TTTTCGTTTTTTAGTTGAAAAGTTTTGAATTTATATAGTTGAATTTTTTTTAATAGTTTG

TTTTACGGGAATTTTTATTACCGTCGTTTTTTAGTTGATGAGTTTTGAATTCGGTAGTTG

AATTTTTTTTAATAGTTTGTGTTACGGGAATTTTTATAAATGTGATTCGTAGTTTTGGTT

TTGAAATGAAGTATTTTTTTTGCGTTAGTTCGGAGTTTTTTTTCGTTTCGGAATTCGGTA

TTAATTGTTATTCGTCGCGTTTTTACGATCGGTTAGGAAGAATATAATAATTAGAATTTT

TTACGGTAGAGTTTTATTGTTTATATTTTTTTGGGGTTAGAGTGTAAGAAGTAAGAGGGA

GAGAAAAATGAAATTTTTTTATTTAAAAGAGAATAATAATTGTTTAGGACGTATTATTTT

TTGATTGGTTGCAGTTTATGGTCGAGTTGACGTTTACGGGAAAAATAGAGTATAAGTAGT

CGTAAATATTTTTGGTTTATGTGTAGATTATTTGTTTATTAATTTAGAATATAGGATGTT

AGTGTTATTTTGTGACGGCGAATGTGGGGGCGGTTTTTTATATCTGTTTGTAGTATTTGT

TTGGTTTTTGTAGTTATTGGAATTTATGT

>CRLTRES5-M13R

AAAAGAATAAAAGGGTTTTTAATATTAGAAAAAATTTAAGGTTAAATATATTTATTGGTA

TTTTCGTTTTTTAGTTGAAAAGTTTTGAATTTATATAGTTGAATTTTTTTTAATAGTTTG

TTTTACGGGAATTTTTATTATCGTCGTTTTTTAGTTGATGAGTTTTGAATTCGGTAGTTG

AATTTTTTTTAATAGCTTGTGTTACGGGAATTTTTATAAACGTGATTCGTAGTTTTGGTT

TTGAAATGAAGTATTTTTTTTGCGTTAGTTCGGAGTTTTTTTTCGTTTCGGAATTCGGTA

TTAATTGTTATTCGTCGCGTTTTTACGATCGGTTAGGAAGAATATAATAATTAGAATTTT

TTACGGTAAAGTTTTATTGTTTATATTTTTTTGGGGTTAGAGTGTAAGAAGTAAGAGAGA

GAAAAACGAAATTTTTTTTATTTAAAAGAGAATAATAATTGTTTAGGACGTATTATTTTT

TGATTGGTTGTAGTTTATGGTCGAGTTGACGTTTACGGGAAAAATAGAGTATAAGTAGTC

GTAAATATTTTTGGTTTATGCGTAGATTATTTGTTTATTAATTTAGAATATAGGATGTTA

GCGTTATTTTGTGACGGCGAATGTGGGGGCGGTTTTTTACATTTGTTTGTAGTATTTGTT

TGGTTTTTGTAGTTATTGGAATTA

>CRLTRES6-M13R

AAAAGAATAAAAGGGTTTTTAATATTAGAAAAAATTTAAGGTTAAATATATTTATTGGTA

TTTTCGTTTTTTAGTTGAAAAGTTTTGAATTTATATAGTTGAATTTTTTTAATAGTTTGT

TTTACGGGAATTTTTATTATCGTCGTTTTTTAGTTGATGAGTTTTGAATTCGGTAGTTGA

ATTTTTTTTAATAGTTTGTGTTACGGGAATTTTTATAAATGTGATTCGTAGTTTTGGTTT

TGAAATGAAGTATTTTTTTTGCGTTAGTTCGGAGTTTTTTTTCGTTTCGGAATTCGGTAT

TAATTGTTATTCGTCGCGTTTTTACGATCGGTTAGGAAGAATATAATAATTAGAATTTTT

TACGGTAGAGTTTTATTGTTTATATTTTTTTGGGGTTAGAGTGTAAGAAGTAAGAGAGAG

AGAAAAATGAAATTTTTTTTATTTAAAAGAGAATAATAATTGTTTAGGACGTATTATTTT

TTGATTGGTTGCAGTTTATGGTCGGGTTGACGTTTACGGGAAAAATAGAGTATAAGTAGT

CGTAAATATTTTTGGTTTATGTGTAGATTATTTGTTTATTAATTTAGAATATAGGATGTT

AGTGTTATTTTGTGACGGCGAATGTGGGGGCGGTTTTTTATATCTGTTTGTAGTATTTGT

TTGGTTTTTGTAGTTATTGGAATTTATGT

>CRLTRES7-M13R

AAAAGAATAAAAGGGTTTTTAATATTAGAAAAAATTTAAGGTTAAATATATTTATTGGTA

TTTTTGTTTTTTAGTTGAAAAGTTTTGAATTTATATAGTTGAATTTTTTTTAATAGTTTG

TTTTACGGGAATTTTTATTATCGTCGTTTTTTAGTTGATGAGTTTTGAATTCGGTAGTTG

AATTTTTTTTAATAGTTTGTGTTACGGGAATTTTTATAAACGTGATTCGTAGTTTTGGTT

TTGAAATGAAGTATTTTTTTTGCGTTAGTTCGGAGTTTTTTTTCGTTTTGGAATTTGGTA

TTAATTGTTATTCGTCGTGTTTTTACGATCGGTTAGGAAGAATATAATAATTAGAATTTT

TTACGGTAAAGTTTTATTGTTTATATTTTTTTGGGGTTAGAGTGTAAGAAGTAAGAGAGA

GAGAAAAATGAAATTTTTTTTATTTAAAAGAGAATAATAATTGTTTAGGACGTATTATTT

TTTGATTGGTTGTAGTTTATGGTCGAGTTGATGTTTACGGGAAAAATAGAGTATAAGTAG

TTGTAAATATTTTTGGTTTATGCGTAGATTATTTGTTTATTAATTTAGAATATAGGATGT

TAGCGTTATTTTGTGACGGCGAATGTGGGGGTGGTTTTTTATATTTGTTTGTAGTATTTG

TTTGGTTTTTGTAGTTATTGGAATTTATGT

>CRLTRES11-M13R

AAAAGAATAAAAGGGTTTTTAATATTAGAAAAAATTTAAGGTTAAATATATTTATTGGTA

TTTTCGTTTTTTAGTTGAAAAGTTTTGAATTTATATAGTTGAATTTTTTTTAATAGTTTG

TTTTACGGGAATTTTTATTATCGTCGTTTTTTAGTTGATGAGTTTTGAATTCGGTAGTTG

AATTTTTTTTAGTAGTTTGTGTTACGGGAATTTTTATAAACGTGATTCGTAGTTTTGGTT

TTGAAATGAAGTATTTTTTTTGCGTTAGTTCGGAGTTTTTTTTCGTTTCGGAATTCGGTA

TTAATTGTTATTCGTCGCGTTTTTACGATCGGTTAGGAAGAATATAATAATTAGAATTTT

TTACGGTAAAGTTTTATTGTTTATATTTTTTTGGGGTTAGAGTGTAAGAAGTAAGAGAGA

GAGAAAAACGAAATTTTTTTTATTTAAAAGAGAATAATAATTGTTTAGGACGTATTATTT

TTTGATTGGTTGTAGTTTATGGTTGAGTTGACGTTTACGGGAAAAATAGAGTATGAGTAG

TCGTAAATATTTTTGGTTTATGCGTAGATTATTTGTTTATTAATTTAGAATATAGGATGT

TAGCGTTATTTTGTGACGGCGAATGTGGGGGCGGTTTTTTATATTTGTTTGTAGTATTTG

TTTGGTTTTTGTAGTTATTGGAATTTATGT

>CRLTRES1_M13R

AAAAGAATAAAAGGGTTTTTAATATTAGAAAAAATTTAAGGTCAAATATACTTATTGGTA

TTTTCGTTTTTTAGTTGAAAAGTTCTGAATTTATATAGTTGAATCTTTTTTAATAGTTTG

TTTTACGGGAATTTTTATNATCGTCGTTCCTCAGTTGATGAGTTTTGAATTCGGCAGTTG

AATCTTTCTTAACAGTTTGTGTTACGGGAACTTTTATAAACGTGATTCGTAGTTTTGGTT

TTGAAATGAAGTATTTTTTTTGTGTTAGTCCGGAGTTTTTTTTCGTTTCGGAATTCGGCA

CTAATTGTTATTCGTCGCGTTTTTACGATCGGTTGGGAAGAATATAATAATTAGAATTTT

TTACGGCAAAGTTTTATTGCTTATATTTTTTTGGGGTTAGAGTGTAAGAAGTAAGAGAGA

GAGAAAAATGAAATCTTTTTTATTTAAAAGAGAATAATAATTGTTTAGGACGTATCATTT

TTTGATTGGTTGTAGTTTATGGTCGAGTTGACGTTTACGGGAAAAATAGAGTATAAGTAG

TCGTAAATATTTTTGGTTCATGCGTAGATTATTTGTTTATTAATTTAGAATACAGGATGT

TAGCGTTATCTTGTGACGGCGAATGTGGGGGCGGCTTCTTATATTTGTTTGTAGTATTTG

TTTGGTTTTTGTAGTTATTGGAATTTATGT

>CRLTRES3_M13R

AAAAGAATAAAAGGGTTTTTAATATTAGAAAAAATTTAAGGTTAAATATATTTATTGGTA

TTTTTGTTTTTTAGTTGAAAAGTTTTGAATTTATATAGTTGAATTTTTTTTAATAGTTTG

TTTTACGGGAATTTTTATNATCGTCGTTTTTTAGTTGATGAGTTTTGAATTTGGTAGTTG

AATTTTTTTTAATAGTTTGTGTTACGGGAATTTTTATAAATGTGATTCGTAGTTTTGGTT

TTGAAATGAAGTATTTTTTTTGCGTTAGTTCGGAGTTTTTTTTCGTTTCGGAATTCGGTA

TTAATTGTTATTCGTCGCGTTTTTACGATCGGTTAGGAAGAATATAATAATTAGAATTTT

TTACGGTAAAGTTTTATTGTTTATATTTTTTGGGGTTAGAGTGTAAGAAGTAAGAGAGAG

AGAAAAATGAAATTTTTTTTATTTAAAAGAGAATAATAATTGTTTAGGACGTATTATTTT

TTGATTGGTTGTAGTTTATGGTTGAGTTGACGTTTACGGGAAAAATAGAGTATAAGTAGT

CGTAAATATTTTTGGTTTATGTGTAGATTATTTGTTTATTAATTTAGAATATAGGATGTT

AGTGTTATTTTGTGACGGCGAATGTGGGGGTGGTTTTTTATATTTGTTTGTAGTATTTGT

TTGGTTTTTGTAGTTATTGGAATTTATGT
