# Supplementary material for: Epigenetic interplay between mouse endogenous retroviruses and host genes
Source: Genome Biol. 2012 Oct 3;13(10):R89. doi: 10.1186/gb-2012-13-10-r89 (PMC3491417; doi:10.1186/gb-2012-13-10-r89)
Supplement: Additional file 4 — All bisulfite sequencing data. Compilation of all bisulfite sequences. [file gb-2012-13-10-r89-S4.zip › IAP6428_TE_oppositeLTR_lung.doc]

3 23 11 Mp Sequences

B6 Lung-CdGAP 5’LTR

>5LTRL_65

AAAAGAATAAAAGGGTTTTTAATATTAGAAAAAATTTAAGGTTAAACATATTTATTGGTA

TTTTCGTTTTTTAGTTGAAAAGTTTTGAATTTATATAGTTGAATTTTTTTTAATAGTTTG

TTTTACGGGAATTTTTATTATCGTCGTTTTTTAGTTGATGAGTTTTGAATTCGGTAGTTG

AATTTTTTTTAATAGTTTGTGTTACGGGAATTTTTATAAACGTGATTCGTAGTTTTGGTT

TTGAAATGAAGTATTTTTTTTGCGTTAGTTCGGAGTTTTTTTTCGTTTCGGAATTCGGTA

TTAATTGTTATTCGTTGCGTTTTTACGATCGGTTAGGAAGAATATAATAATTAGAATTTT

TTACGGTAAAGTTTTATTGTTTATATTTTTTTGGGGTTAGAGTGTAAGAAGTAAGAGAGA

GAGAAAAACGAAATTTTTTTTATTTAAAAGAGAATAATAATTGTTTAGGACGTATTATTT

TTTGATTGGTTGTAGTTTATGGTCGAGTTGACGTTTACGGGAAAAATAGAGTATAAGTAG

TTGTAAATATTTTTGGTTTATGTGTAGATTATTTGTTTATTAATTTAGAATATAGGATGT

TAGCGTTATTTTGTGACGGCGAATGTGGGGGCGGTTTTTTATATTTGTTTGTAGT

>5LTRL_66

AAAAGAATAAAAGGGTTTTTAATATTAGAAAAAACTTAAGGTTAAATATATTTATTGGTA

TTTTCGTTTTTTAGTTGAAAAGTTTTGAATTTATATAGTTGAATTTTTTTTAATAGTTTG

TTTTACGGGAATTTTTATNATCGTCGTTTTTTAGTTGATGAGTTTTGAATTCGGTAGTTG

AATTTTTTTTAATAGTTTGTGTTACGGGAATTTTTATAAATGTGATTCGTAGTTTTGGTT

TTGAAATGAAGTATTTTTTTTGCGTTAGTTCGGAGTTTTTTTTCGTTTCGGAATTCGGTA

TTAATTGTTATTCGTCGCGTTTTCACGATCGGTTAGGAAGAATATAATAATTAGAATTTT

TTACGGTAAAGTTTTATTGTTTATATTTTTTTGGGGTTAGAGTGTAAGAAGTAAGAGAGA

GAGAAAAACGAAATTTTTTTTATTTAAAAGAGAATAATAATTGTTTAGGACGTATTATTT

TTTGATTGGTTGTAGTTTATGGTCGAGTTGACGTTTACGGGAAAAATAGAGTATAAGTAG

TCGTAAATATTTTTGGTTTATGCGTAGATTATTTGTTTATTAATTTAGAATATAGGATGT

TAGCGTTATTTTGTGACGGCGAATGTGGGGGCGGTTTTTTATATTTGTTTGTAGTATTTG

TTTGGTTTTTGTAGTTATTGGAATTTATGT

>5LTRL_67

AAAAGAATAAAAGGGTTTTTAATATTAGAAAAAATTTAAGGTTAAATATATTTATTGGTA

TTTTCGTTTTTTAGTTGAAAAGTTTTGAANTTATATGGTTGGATTTTTTTTAATAGTTTG

TTTTACGGGAATTTTTATCATCGTCGTTTTTTAGTTGATGAGTTTTGAATTTGGTAGTTG

AATTTTTTTTAATAGTTTGTGTTACGGGAATTTTTATAAATGTGATTCGTAGTTTTGGTT

TTGAAATGAAGTATTTTTTTTGCGTTAGCTTGGAGTTTTTTTTCGTTTCGGAATTCGGTA

TTAATTGTTATTCGTCGCGTTTTTACGATCGGTTAGGAAGAATATAATAATTAGAATTTT

TTACGGTAAAGTTTTATTGTTTATATTTTTTTGGGGTTAGAGTGTAAGAAGTAAGAGAGA

GAGAAAAACGAAATTTTTTTTATTTAAAAGAGAATAATAATTGTTTAGGACGTATTATTT

TTTGATTGGTTGTAGTTTATGGTCGAGTTGACGTTTACGGGAAAAATAGAGTATAAGTAG

TCGTAAATATTTTTGGTTTATGCGTAGATTATTTGTTTATTAATTTAGAATATAGGATGT

TAGCGTTATTTTGTGACGGTGAATGTGGGGGCGGTTTTTTATATTTGTTTGTAGTATTTG

TTTGGTTTTTGTAGTTATTGGAATTTATGT

>5LTRL_68

AAAAGATTAAAAGGGTTTTTAATATTAGAAAAAATTTAAGGTTAAATATATTTATTGGTA

TTTTCGTTTTTTAGTTGAAAAGTTTTGAATTTATATAGTTGAATTTTTTTTAATAGTTTG

TTTTACGGGAATTTTTATNATCGTCGTTTTTTAGTNGATGAGTTTTGAATTCGGTAGTTG

AATTTTTTTTAATAGTTTGTGTTACGGGAATTTTTATAAATGTGATTCGTAGTTTTGGTT

TTGAAATGAAGTATTTTTTTTGCGTTAGTTCGGAGTTTTTTTTCGCTTCGGAATTCGGTA

TTAATTGTTATTCGTCGCGTTTTCACGATCGGTTAGGAAGAATATAATAATTAGAATTTT

TTACGGTAAAGTTTTATTGTTTATATTTTTTTGGGGTTAGAGTGTAAGAAGTAAGAGAGA

GAGAAAAACGAAATTTTTTTTATTTAAAAGAGAATAATAATTGTTTAGGACGTATTATTT

TTTGATTGGTTGTAGTTTATGGTCGAGTTGACGTTTACGGGAAAAATAGAGTATAAGTAG

TCGTAAATATTTTTGGTTTATGCGTAGATTATTTGTTTATTAATTTAGAATATAGGATGT

TAGCGTTATTTTGTGACGGCGAATGTGGGGGCGGTTTTTTATATTTGTTTGTAGTATTTG

TTTGGTTTTTGTAGTTATTGGAATTTATGT

>5LTRL_69

TAAAAGGGTTTTTAATATTAGAAAAAATTTAAGGTTAAGTATATTTATTGGTATTTTCGT

TTTTTAGTTGAAAAGTTTTGAATTTATATAGTTGAATTTTTTTTAATAGTTTGTTTTACG

GGAATTTTTATATCGTCGTTTTTTAGTTGATGAGTTTTGAATTCGGTAGTTGAATTTTTT

TTAATAGTTTGTGTTACGGGAATTTTTATAAACGTGATTCGTAGTTTTGGTTTTGAAATG

AAGTATTTTTTTTGCGTTAGTTCGGAGTTTTTTTTCGTTTCGGAATTCGGTATTAATTGT

TATTCGTTGCGTTTTTACGATCGGTTAGGAAGAATATAATAATTAGAATTTTTTACGGTA

AAGTTTTATTGTTTATATTTTTTTGGGGTTAGAGTGTAAGAAGTAAGAGAGAGAGAAAAA

CGAAATTTTTTTTATTTAAAAGAGAATAATAATTGTTTAGGACGTATTATTTTTTGATTG

GTTGTAGTTTATGGTCGAGTTGACGTTTACGGGAAAAATAGAGTATAAGTAGTCGTAAAT

ATTTTTGGTTTATGCGTAGATTATTTGTTTATTAATTTAGAATATAGGATGTTGGCGTTA

TTTTGTGACGGTGAATGTGGGGGCGGTTTTTTATATTTGTTTGTAGTATTTGTTTGGTTT

TTGTAGTTATTGGAA

>5LTRL_70

GAATAAAAGGGTTTTTAATATTAGAAAAAATTTAAGGTTAAGTATATTTATTGGTATTTT

CGTTTTTTAGTTGAAAAGTTTTGAATTTATATAGTTGAATTTTTTTTAATAGTTTGTTTT

ACGGGAATTTTTATTATCGTCGTTTTTTAGTTGATGAGTTTTGAATTCGGTAGTTGAATT

TTTTTTAATAGTTTGTGTTACGGGAATTTTTATAAACGTGATTCGTAGTTTTGGTTTTGA

AATGAAGTATTTTTTTTGCGTTAGTTCGGAGTTTTTTTTCGTTTCGGAATTCGGTATTAA

TTGTTATTCGTTGCGTTTTTACGATCGGTTAGGAAGAATATAATAATTAGAATTTTTTAC

GGTAAAGTTTTATTGTTTATATTTTTTTGGGGTTAGAGTGTAAGAAGTAAGAGAGAGAGA

AAAACGAAATTTTTTTTATTTAAAAGAGAATAATAATTGTTTAGGACGTATTATTTTTTG

ATTGGTTGTAGTTTATGGTCGAGTTGACGTTTACGGGAAAAATAGAGTATAAGTAGTCGT

AAATATTTTTGGTTTATGCGTAGATTATTTGTTTATTAATTTAGAATATAGGATGTTGGC

GTTATTTTGTGACGGTGAATGTGGGGGCGGTTTTTTATATTTGTTTGTAGTATTTGTTTG

GTTTTTGTAGTTATTGGAA

>5LTRL_71

AAAAGAATAAAAGGGTTTTTAATATTAGAAAAAATTTAAGGTTAAGTATATTTATTGGTA

TTTTCGTTTTTTAGTTGAAAAGTTTTGAATTTATATAGTTGAATTTTTTTAATAGTTCGT

TTTACGGGAATTTTTATCATCGTCGTTTTTTAGTTGATGAGTTTTGAATTCGGTAGTTGA

ATTTTTTTTAATAGTTTGTGTTACGGGAATTTTTATAAACGTGATTCGTAGTTTTGGTTT

TGAAATGAAGTATTTTTTTTGCGTTAGTTCGGAGTTTTTTTTCGTTTCGGAATTCGGTAT

TAATTGCTATTCGTCGCGTTTTTATGATCGGTTAGGAAGAATATAATAATTAGAATTTTT

TACGGTAAAGTTTTATTGTTTATATTTTTTTGGGGTTAGAGTGTAAGAAGTAAGAGAGAG

AGAAAAACGAAATTTTTTTTATTTAAAAGAGAATAATAATTGTTTAGGACGTATTATTTT

TTGATTGGTTGTAGTTTATGGTCGAGTTGACGTTTACGGGAAAAATAGAGTATAGGTAGT

CGTAAATATTTTTGGTTTATGCGTAGATTATTTGTTTATTAATTTAGAATATAGGATGTT

AGCGTTATTTTGTGATGGCGAATGTGGGGGCGGTTTTTTATATTTGTTTGTAGTATTTGT

TTGGTTTTTGTAGTTATTGGAATTTATGT

>5LTRL_72

AAAAGAATAAAAGGGTTTTTAATATTAGAAAAAATTTAAGGTTAAATATATTTATTGGTA

TTTTCGTTTTTTAGTTGAAAAGTTTTGAATTTATATAGTTGAATTTTTTTTAATAGTTTG

TTTTACGGGAATTTTTATTATCGTCGTTTTTTAGTTGATGAGTTTTGAATTTGGTAGTTG

AATTTTTTTTAATAGTTTGTGTTACGGGAATTTTTATAAACGTGATTCGTAGTTTTGGTT

TTGAAATGAAGTATTTTTTTTGCGTTAGTTCGGAGTTTTTTTTCGTTTCGGAATTCGGTA

TTAATTGTTATTCGTCGCGTTTTTACGATCGGTTAGGAAGAATATAATAATTAGAATTTT

TTACGGTAAAGTTTTATTGTTTATATTTTTTTGGGGTTAGAGTGTAAGAAGTAAGAGAGA

GAGAAAAACGAAATTTTTTTTATTTAAAAGAGGATAATAATTGTTTAGGACGTATTATTT

TTTGATTGGTTGTAGTTTATGGTCGAGTTGACGTTTACGGGAAAAATAGAGTATAAGTAG

TCGTAAATATTTTTGGTTTATGCGTAGATTATTTGTTTATTAATTTAGAATATAGGATGT

TAGCGTTATTTTGTGACGGCGAATGTGGGGGCGGTTTTTTATATTTGTTTGTAGTATTTG

TTTGGTTTTTGTAGTTATTGGAATTTATGT

>5LTRL_73

AAAAGAATAAAAGGGTTTTTAATATTAGAAAAAATTTAAGGTTAAATATATTTATTGGTA

TTTTCGTTTTTTAGTTGAAAAGTTTTGAATTTATATAGTTGAATTTTTTTTAATAGTTTG

TTTTACGGGAACTTTTATTATCGTCGTTTTTTAGTTGATGAGTTTTGAATTCGGTAGTTG

AATTTTTTTTAATAGTTTGTGTTACGGGAATTTTTATAAATGTGATTCGTAGTTTTGGTT

TTGAAATGAGGTATTTTTTTTGCGTTAGTTCGGAGTTTTTTCTCGTTCCGGAATTCGGTA

TTAATTGTTATTCGTCGCGTTTTTACGATCGGTTAGGAAGAATATAATAATTAGAATTTT

TTACGGTAAAGTTTTATTGTTTATATTTTTTTGGGGTTAGAGTGTAAGAAGTAAGAGAGA

GAGAAAAACGAAATTTTTTTTATTTAAAAGAGAATAATAATTGTTTAGGACGTATTATTT

TTTGATTGGTTGTAGTTTATGGTCGAGTTGACGTTTACGGGAAAAATAGAGTATAAGTAG

TCGTAAATATTTTTGGTTTATGCGTAGATTATTTGTTTATTAATTTAGAATATAGGATGT

TAGCGTTATTTTGTGACGGCGAATGTGGGGGCGGTTTTTTATATTTGTTTGTAGTATTTG

TTTGGTTTTTGTAGTTATTGGAATTTATGT

>5LTRL_74

TAAAGGGTTTTTAATATTAGAAAAAATTTAAGGTTAAATATATTTATTGGTATTTTCGTT

TTTTAGTTGAAAAGTTTTGAATTTATATGGTTGAATTTTTTTTAATAGTTTGTTTTACGG

GAATTTTTATTATCGTCGTTTTTTAGTTGATGAGTTTTGAATTTGGTAGTTGAATTTTTT

TTAATAGTTTGTGTTACGGGAATTTTTATAAATGTAATTCGTAGTTTTGGTTTTGAAATG

AAGTATTTTTTTTGCGTTAGTTTGGAGTTTTTTTTCGTTTCGGAATTCGGTATTAATTGT

TATTCGTCGCGTTTTTACGATCGGTTAGGAAGAATATAATAATTAGAATTTTTTACGGTA

AAGTTTTATTGTTTATATTTTTTGGGGTTAGAGTGTAAGAAGTAAGAGAGAGAGAAAAAC

GAAATTTTTTTTATTTAAAAGAGAATAATAATTGTTTAGGACGTATTATTTTTTGATTGG

TTGTAGTTTATGGTCGAGTTGACGTTTACGGGAAAAATAGAGTATAAGTAGTCGTAAATA

TTTTTGGTTTATGCGTAGATTATTTGTTTATTAATTTAGAATATAGGATGTTAGCGTTAT

TTTGTGACGGTGAGTGTGGGGGCGGTTTTTTATATTTGTTTGTAGTATTTGTTTGGTTTT

TGTAGTTATTGGAATTTATGT

>5LTRL_75

GAATAAAAGGGTTTTTAATATTAGAAAAAGTTTAAGGTTAAATATATTTATTGGTATTTT

CGTTTTTTAGTTGAAAAGTTTTGAATTTATATAGTTGAATTTTTTTTAATAGTTTGTTTT

ACGGGAATTTTTATTATCGTCGTTTTTTAGTTGATGAGTTTTGAATTCGGTAGTTGAATT

TTTTTTAATAGTTTGTGTTACGGGAATTTTTATAAACGTGATTCGTAGTTTTGGTTTTGA

AATGAAGTATTTTTTTTGCGTTAGTTCGGAGTTTTTTTTCGTTTCGGAATTCGGTATTAA

TTGTTATTCGTCGCGTTTTTACGATCGGTTAGGAAGAATATAATAATTAGAATTTTTTAC

GGTAAAGTTTTATTGTTTATATTTTTTTGGGGTTAGAGTGTAAGAAGTAAGAGAGAGAGA

AAAATGAAATTTTTTTTATTTAAAAGAGAATAATAATTGTTTAGGACGTATTATTTTTTG

ATTGGTTGTAGTTTATGGTCGAGTTGACGTTTACGGGAAAAATAGAGTATAAGTAGTCGT

AAATATTTTTGGTTTATGCGTAGATTATTTGTTTATTAATTTGGAATATAGGATGTTAGC

GTTATTTTGTGACGGCGAATGTGGGGGCGGTTTTTTATATTTGTTTGTAGTATTTGTTTG

GTTTTTGTAGTTATTGGAATTTATGT

>5LTRL_76

AAAAGAATAAAAGGGTTTTTAATATTAGAAAAAATTTAAGGTTAAGTATATTTATTGGTA

TTTTCGTTTTTTAGTTGAAAAGTTTTGAATTTATATAGTTGAATTTTTTTTAATAGTTTG

TTTTACGGGAATTTTTATATCGTCGTTTTTTAGTTGATGAGTTTTGAATTCGGTAGTTGA

ATTTTTTTTAATAGTTTGTGTTACGGGAATTTTTATAAACGTGATTCGTAGTTTTGGTTT

TGAAATGAAGTATTTTTTTTGCGTTAGTTCGGAGTTTTTTTTCGTTTCGGAATTCGGTAT

TAATTGTTATTCGTCGCGTTTTTATGATCGGTTAGGAAGAATATAATAATTAGAATTTTT

TACGGTAAAGTTTTATTGTTTATATTTTTTTGGGGTTAGAGTGTAAGAAGTAAGAGAGAG

AGAAAAACGAAATTTTTTTTATTTAAAAGAGAATAATAATTGTTTAGGACGTATTATTTT

TTGATTGGTTGTAGTTTATGGTCGAGTTGACGTTTACGGGAAAAATAGAGTATAAGTAGT

CGTAAATATTTTTGGTTTATGCGTAGATTATTTGTTTATTAATTTAGAATATAGGATGTT

AGCGTTATTTTGTGATGGCGAATGTGGGGGCGGTTTTTTATATTTGTTTGTAGTATTTGT

TTGGTTTTTGTAGTTATTGGAATTTATGT

>5LTRL_77

GGGTTTTTAATATTAGAAAAAATTTAAGGTTAAATATATTTATTGGTATTTTCGTTTTTT

AGTTGAAAAGTTTTGAATTTATATAGTTGAATTTTTTTTAATAGTTTGTTTTACGGGAAT

TTTTATTATCGTCGTTTTTTAGTTGATGAGTTTTGAATTCGGTAGTTGAATTTTTTTTAA

TAGTTTGTGTTACGGGAATTTTTATAAACGTGATTCGTAGTTTTGGTTTTGAAATGAAGT

ATTTTTTTTGCGTTAGTTCGGAGTTTTTTTTCGTTTCGGAATTCGGTATTAATTGTTATT

CGTTGCGTTTTTACGATCGGTTAGGAAGAATATAATAATTAGAATTTTTTACGGTAAGGT

TTTATTGTTTATATTTTTTTGGGGTTAGAGTGTAAGAAGTAAGAGAGAGAGAAAAATGAA

ATTTTTTTTATTTAAAAGAGAATAATAATTGTTTAGGACGTATTATTTTTTGATTGGTTG

TAGTTTATGGTCGAGTTGACGTTTACGGGAAAAATAGAGTACAAGTAGTCGTAAATATTT

TTGGTTTATGCGTAGATTATTTGTTTATTAATTTAGAATATAGGATGTTAGTGTTATTTT

GTGACGGCGAATGTGGGGGCGGTTTTTTATATTTGTTTGTAGTATTTGTTTGGTTTTTGT

AGTTATTGGAATTTATGT

>5LTRL_78

GGGTTTTTAATATTAGAAAAAGTTTAAGGTTAAATATATTTATTGGTATTTTCGTTTTTT

AGTTGAAAAGTTTTGAATTTATATAGTTGAATTTTTTTTAATAGTTTGTTTTACGGGAAT

TTTTATCATCGTCGTTTCTTAGTTGATGAGTTTTGAATTCGGTAGTTGAATTTTTTTTAA

TAGTTTGTGTTACGGGAATTTTTATAAACGTGATTCGTAGTTTTGGTTTTGAAATGAAGT

ATTTTTTTTGCGTTAGTTCGGAGTTTTTTTTCGTTTCGGAATTCGGTATTAATTGTTATT

CGTCGCGTTTTTACGATCGGTTAGGAAGAATATAATAATTAGAATTTTTTACGGTAAAGT

TTTATTGTTTATATTTTTTTGGGGTTAGAGTGTAAGAAGTAAGAGAGAGAGAAAAATGAA

ATTTTTTTTATTTAAAAGAGAATAATAATTGTTTAGGACGTATTATTTTTTGATTGGTTG

TAGTTTATGGTCGAGTTGACGTTTACGGGAAAAATAGAGTATAAGTAGTCGTAAATATTT

TTGGTTTATGCGTAGATTATTTGTTTATTAATTTAGAATATAGGATGTTAGCGTTATTTT

GTGACGGCGAATGTGGGGGCGGTTTTTTATATTTGTTTGTAGTATTTGTTTGGTTTTTGT

AGCTATTGGAATTTATGTAATCACTAGT

>5LTRL_79

AAAAGAATAAAAGGGTTTTTAATATTAGAAAAAATTTAAGGTTAAATATATTNATTGGTA

TTTTCGTTTTTTAGTTGAAAAGTTTTGAATTTATATGGTTGAATTTTTTTTAATAGTTTG

TTTTACGGGAATTTTTATTATCGTCGTTTTTTAGTTGATGAGTTTTGAATTTGGTAGTTG

AATTTTTTTTAATAGTTTGTGTTACGGGAATTTTTATAAATGTGATTCGTAGTTTTGGTT

TTGAAATGAAGTATTTTTTTTGCGTTAGTTTGGAGTTTTTTTTCGTTTCGGAATTCGGTA

TTAATTGTTATTCGTCGCGTTTTTACGATCGGTTAGGAAGAATATAATAATTAGAATTTT

TTACGGTAAAGTTTTATTGTTTATATTTTTTTGGGGTTAGAGTGTAAGAAGTAAGAGAGA

GAGAAAAACGAAATTTTTTTTATTTAAAAGAGAATAATAATTGTTTAGGACGTATTATTT

TTTGATTGGTTGTAGTTTATGGTCGAGTTGACGTTTACGGGAAAAATAGAGTATAAGTAG

TCGTAAATATTTTTGGTTTATGCGTAGATTATTTGTTTATTAATTTAGAATATAGGATGT

TAGCGTTATTTTGTGACGGTGAATGTGGGGGCGGTTTTTTATATTTGTTTGTAGTATTTG

TTTGGTTTTTGTAGTTATTGGAATTTATGT

>5LTRL_80

AAAAGAATAAAAGGGTTTTTAATATTAGAAAAAATTTAAGGTTAAATATATTTATTGGTA

TTTTCGTTTTTTAGTTGAAAAGTTTTGAATTTATATAGTTGAATTTTTTTTAATAGTTTG

TTTTACGGGAATTTTTATATCGTCGTTTTTTAGTTGATGAGTTTTGAATTCGGTAGTTGA

ATTTTTTTTAATAGTTTGTGTTACGGGAATTTTTATAAATGTGATTCGTAGTTTTGGTTT

TGAAATGAAGTATTTTTTTTGCGTTAGTTCGGAGTTTTTTTTCGTTTCGGAATTCGGTAT

TAATTGTTATTCGTCGCGTTTTTACGATCGGTTAGGAAGAATATAATAATTAGAATTTTT

TACGGTAAAGTTTTATTGTTTATATTTTTTTGGGGTTAGAGTGTAAGAAGTAAGAGAGAG

AGAAAAACGAAATTTTTTTTATTTAAAAGAGAATAATAATTGTTTAGGACGTATTATTTT

TTGATTGGTTGTAGTTTATGGTTGAGTTGATGTTTACGGGAAAAATAGAGTATAAGTAGT

CGTAAATATTTTTGGTTTATGCACAGATTATTTGTTTATTAATTTAGAATATAGGATGTT

AGCGTTATTTTGTGACGGCGAATGTGGGGGCGGTTTTTTATATTTGTTTGTAGTATTTGT

TTGGTTTTTGTAGTTATTGGAATTTATGT

%Me-CpG

86.7%

86.7%

90%

93.3%

93.3%

96.7%

96.7%

96.7%

96.7%

96.7%
