# Supplementary material for: Epigenetic interplay between mouse endogenous retroviruses and host genes
Source: Genome Biol. 2012 Oct 3;13(10):R89. doi: 10.1186/gb-2012-13-10-r89 (PMC3491417; doi:10.1186/gb-2012-13-10-r89)
Supplement: Additional file 4 — All bisulfite sequencing data. Compilation of all bisulfite sequences. [file gb-2012-13-10-r89-S4.zip › IAP6428_TE_oppositeLTR_thymus.doc]

CdGAP 5LTR

B6129 Thymus

B6 Clones

>CRLTRThyMP1-M13R

AAAAGAATAAAAGGGTTTTTAATATTAGAAAAAATTTAAGGTTAAATATATTTATTGGTA

TTTTCGTTTTTTAGTTGAAAAGTTTTGAATTTATATAGTTGAATTTTTTTTAATAGTTTG

TTTTACGGGAATTTTTATTATCGTCGTTTTTTAGTTGATGAGTTTTGAATTCGGTAGTTG

AATTTTTTTTAATAGTTTGTGTTACGGGAATTTTTGTAAACGTGATTCGTAGTTTTGGTT

TTGAAATGAAGTATTTTTTTTGCGTTAGTTCGGAGTTTTTTTTCGTTTCGGAATTCGGTA

TTAATTGTTATTCGTCGCGTTTTTACGATCGGTTAGGAAGAATATAATAATTAGAATTTT

TTACGGTAAAGTTTTATTGTTTATATTTTTTTGGGGTTAGAGTGTAAGAAGTAAGAGAGA

GAGAAAAACGAAATTTTTTTTATTTAAAAGAGAATAATAATTGTTTAGGACGTATTATTT

TTTGATTGGTTGTAGTTTATGGTCGAGTTGACGTTTACGGGAAAAATAGAGTATAAGTAG

TCGTAAATATTTTTGGTTTATGCGTAGATTATTTGTTTATTAATTTAGAATATAGGATGT

TAGCGTTATTTTGTGACGGCGAATGTGGGGGCGGTTTTTTATATTTGTTTGTAGTATTTG

TTTGGTTTTTGTAGTTATTGGAATTTATGT

>CRLTRThyMP5-M13R

AAAAGAATAAAAGGGTTTTTAATATTAGAAAAAATTTAAGGTTAAATATATTTATTGGTA

TTTTCGTTTTTTAGTTGAAAAGTTTTGAATTTATATAGTTGAATTTTTTTTAATAGTTTG

TTTTACGGGAATTTTTATATCGTCGTTTTTTAGTTGATGAGTTTTGAATTTGGTAGTTGA

ATTTTTTTTAATAGTTTGTGTTATGGGAATTTTTATAAATGTGATTTGTAGTTTTGGTTT

TGAAATGAAGTATTTTTTTTGCGTCAGTCCGGAGTTTTTTCTCGTTTCGGAATTCGGCAT

CAATTGTTATTCGTCGCGTTCTCACGACCGGTTAGGAAGAACACAACAATTAGAATTTTT

TACGGCAAAGTTTTATTGTTTATATCTTTTTGGGGTTAGAGTGTAAGAAGTAAGAGAGAG

AGAAAAACGAAATCCCTTTTATTTAAAAGAGAATAATAATTGTTTAGGACGTATCATTTC

TTGATTGGTTGTAGTTTATGGTCGAGTTGACGTTCACGGGAAAAATAGAGTATAAGTAGT

CGTAAATATTTTTGGTTTATGCGTAGATTATTTGTTTATTAATTTAGAATATAGGATGTT

AGCGTTATTTTGTGACGGCGAATGTGGGGGCGGTTTTTTATATTTGTTTGTAGTATTTGT

TTGGTTTTTGTAGTTATTGGAATTTATGT

>CRLTRThyMP9-M13R

AAAAGAATAAAAGGGTTTTTAATATTAGAAAAAATTTAAGGTTAAATATATTTATTGGTA

TTTTCGTTTTTTAGTTGAAAAGTTTTGAATTTATATAGTTGAATTTTTTTTAATAGTTTG

TTTTACGGGAATTTTTATTATCGTCGTTTTTTAGTTGATGAGTTTTGAATTCGGTAGTTG

AATTTTTTTTAATAGTTTGTGTTACGGGAATTTTTATAAACGTGATTCGTAGTTTTGGTT

TTGAAATGAAGTATTTTTTTTGCGTTAGTTCGGAGTTTTTTTTCGTTACGGAATTCGGTA

TTAATTGTTATTCGTCGCGTTTTTACGATCGGTTAGGAAGAATATAATAATTAGAATTTT

TTACGGTAAAGTTTTATTGTTTATATTTTTTTGGGGTTAGAGTGTAAGAAGTAAGAGAGA

GAGAAAAACGAAATTTTTTTTATTTAAAAGAGAATAATAATTGTTTAGGACGTATTATTT

TTTGATTGGTTGTAGTTTATGGTCGAGTTGACGTTTACGGGAAAAATAGAGTATAAGTAG

TCGTAAATATTTTTGGTTTATGCGTAGATTATTTGTTTATTAATTTAGAATATAGGATGT

TAGCGTTATTTTGTGACGGCGAATGTGGGGGCGGTTTTTTATATTTGTTTGTAGTATTTG

TTTGGTTTTTGTAGTTATTGGAATTTATGT

>CRLTRThyMP10-M13R

AAAAGAATAAAAGGGTTTTTAATATTAGAAAAAATTTAAGGTTAAATATATTTATTGGTA

TTTTCGTTTTTTAGTTGAAAAGTTTTGAATTTATATAGTTGAATTTTTTTTAATAGTTTG

TTTTACGGGAATTTTTATATCGTCGTTTTTTAGTTGATGAGTTTTGAATTCGGTAGTTGA

ATTTTTTTTAATAGTTTGTGTTACGGGAATTTTTATAAACGTGATTCGTAGTTTTGGTTT

TGAAATGAAGTATTTTTTTTGCGTTAGTTCGGAGTTTTTTTTCGTTTCGGAATTCGGTAT

TAATTGTTATTCGTCGCGTTTTTACGATCGGTTAGGAAGAATATAATAATTAGAATTTTT

TACGGTAAAGTTTTATTGTTTATATTTTTTTGGGGTTAGAGTGTAAGAAGTAAGAGAGAG

AGAAAAACGAAATTTTTTTTATTTAAAAGAGAATAATAATTGTTTAGGACGTATTATTTT

TTGATTGGTTGTAGTTTATGGTCGAGTTGACGTTTACGGGAAAAATAGAGTATAAGTAGT

CGTAAATACTTTTGGTTTATGCGCAGATTATTTGTTTATTAATTTAGAATATAGGATGTT

AGCGTTATTTTGTGACGGCGAATGTGGGGGCGGTTTTTTATACTTGTTTGTAGTATTTGT

TTGGTTTTTGTAGTTATTGGAATTTATGT

“B” Miniprep Sequences B6 Clones

>CRLTRThy2B-R

AAAAGAATAAAAGGGTTTTTAATATTAGAAAAAATTTAAGGTTAAATATATTTATTGGTA

TTTTCGTTTTTTAGTTGAAAAGTTTTGAATTTATATAGTTGAATTTTTTTTAATAGTTTG

TTTTACGGGAATTTTTATATCGTCGTTTTTTAGTTGATGAGTTTTGAATTCGGTAGTTGA

ATTTTTTTTAATAGTTTGTGTTACGGGAATTTTTATAAACGTGATTCGTAGTTTTGGTTT

TGAAATGAAGTATTTTTTTTGCGTTAGTTCGGAGTTTTTTTTCGTTTCGGAATTCGGTAT

TAATTGTTATTCGTCGCGTTTTTACGATCGGTTAGGAAGAATATAATAATTAGAATTTTT

TACGGTAAAGTTTTATTGTTTATATTTTTTTGGGGTTAGAGTGTAAGAAGTAAGAGAGAG

AGAAAAACGAAATTTTTTTTATTTAAAAGAGAATAATAATTGTTTAGGACGTATTATTTT

TTGATTGGTTGTAGTTTATGGTCGAGTTGACGTTTACGGGAAAAATAGAGTATAAGTAGT

CGTAAATATTTTTGGTTTATGCGTAGATTATTTGTTTATTAATTTAGAATATAGGATGTT

AGCGTTATTTTGTGACGGCGAATGTGGGGGCGGTTTTTTATATTTGTTTGTAGTATTTGT

TTGGTTTTTGTAGTTATTGGAATTTATGT

>CRLTRThy3B-R

AAAAGAATAAAAGGGTTTTTAATATTAGAAAAAATTTAAGGTTAAATATATTCATTGGTA

TTTTCGTTTTTTAGTTGAAAAGTTTTGAATTTATATAGTTGAATTTTTTTTAATAGTTTG

TTTTACGGGAATTTTTATATCGTCGTTTTTTAGTTGATGAGTTTTGAATTCGGTAGTTGA

ATTTTTTTTAATAGTTTGTGTTACGGGAATTTTTATAAACGTGATTCGTAGTTTTGGTTT

TGAAATGAAGTATTTTTTTTGCGTTAGTTCGGAGTTTTTTTTCGTTTCGGAATTCGGTAT

TAGTTGTTATTCGTCGCGTTTTTACGACCAGTTAGGAAGAATATAATAATTAGAATTTTT

TACGGTAAAGTTTTATTGTTTATATTTTTTTGGGGTTAGAGTGTAAGAAGTAAGAGAGAG

AGAAAAACGAAATTTTTTTTATTTAAAAGAGAATAATAATTGTTTAGGACGTATTATTTT

TTGATTGGTTGTAGTTTATGGTCGAGTTGACGTTTACGGGAAAAATAGAGTATAAGTAGT

CGTAAATATTTTTGGTTTATGCGTAGATTATTTGTTTATTAATTTAGAATATAGGATGTT

AGCGTTATTTTGTGACGGCGAATGTGGGGGCGGTTTTTTATATTTGTTTGTAGTATTTGT

TTGGTTTTTGTAGTTATTGGAATTTATGT

>CRLTRThy4B-R

AAAAGAATAAAAGGGTTTTTAATATTAGAAAAAATTTAAGGTTAAATATATTTATTGGTA

TTTTCGTTTTTTAGTTGAAAAGTTTTGAATTTATATAGTTGAATTTTTTTTAATAGTTTG

TTTTACGGGAATTTTTATCATCGTCGTTTTTTAGTTGATGAGTTTTGAATTCGGTAGTTG

AATTTTTTTTAATAGTTTGTGTTACGGGAATTTTTATAAACGTGATTCGTAGTTTTGGTT

TTGAAATGAAGTATTTTTTTTGCGTTAGTTCGGAGTTTTTTTTCGTTTCGGAATTCGGTA

TTAGTTGTTATTCGTCGCGTTTTTACGACCGGTTAGGAAGAATATAATAATTAGAATTTT

TTACGGTAAAGTTTTATTGTTTATATTTTTTTGGGGTTAGAGTGTAAGAAGTAAGAGAGA

GAGAGAAACGAAATTTTTTTTATTTAAAAGAGAATAATAATTGTTTAGGACGTATTATTT

TTTGATTGGTTGTAGTTTATGGTCGAGTTGACGTTTACGGGAAAAATAGAGTATAAGTAG

TCGTAAATATTTTTGGTTTATGCGTAGATTATTTGTTTATTAATTTAGAATATAGGATGT

TAGCGTTATTTTGTGACGGCGAATGTGGGGGCGGTTTTTTATATTTGTTTGTAGTATTTG

TTTGGTTTTTGTAGTTATTGGAATTTATGT

>CRLTRThy5B-R

AAAAGAATAAAAGGGTTTTTAATATTAGAAAAAATTTAAGGTTAAATATATTTATTGGTA

TTTTCGTTTTTTAGTTGAAAAGTTTTGAATTTATATAGTTGAATTTTTTTTAATAGTTTG

TTTTACGGGAATCTTTATATCGTCGTTTTTTAGTTGATGAGTTTTGAATTCGGTAGTTGA

ATTTTTTTTAATAGTTTGTGTTACGGGAATTTTTATAAACGTGATTCGTAGTTTTGGTTT

TGAAATGAAGTATTTTTTTTGCGTTAGTTCGGAGTTTTTTTTCGTTTCGGAATTCGGTAT

TAATTGTTATTCGTCGCGTTTTTACGATCGGTTAGGAAGAATATAATAATTAGAATTTTT

TACGGTAAAGTTTTATTGTTTATATTTTTTTGGGGTTAGAGTGTAAGAAGTAAGAGAGAG

AGAAAAACGAAATTTTTTTTATTTAAAAGAGAATAATAATTGTTTAGGACGTATTATTTT

TTGATTGGTTGTAGTTTATGGTCGAGTTGACGTTTACGGGAAAAATAGAGTATAAGTAGT

CGTAAATATTTTTGGTTTATGCGTAGATTATTTGTTTATTAATTTAGAATATAGGATGTT

AGCGTTATTTTGTGACGGCGAATGTGGGGGCGGTTTTTTATATTTGTTTGTAGTATTTGT

TTGGTTTTTGTAGTTATTGGAATTTATGT

>CRLTRThy6B-R

AAAAGAATAAAAGGGTTTTTAATATTAGAAAAAATTTAAGGTTAAATATATTTATTGGTA

TTTTCGTTTTTTAGTTGAAAAGTTTTGAATTTATATAGTTGAATTTTTTTTAATAGTTTG

TTTTACGGGAATTTTTATATCGTCGTTTTTTAGTTGATGAGTTTTGAATTCGGTAGTTGA

ATTTTTTTTAATAGTTTGTGTTACGGGAATTTTTATAAACGTGATTCGTAGTTTTGGTTT

TGAAATGAAGTATTTTTTTTGCGTTAGTTCGGAGTTTTTTTTCGTTTCGGAATTCGGTAT

TAATTGTTATTCGTCGCGTTTTTACGATCGGTTAGGAAGAATATAATAATTAGAATTTTT

TACGGTAAAGTTTTATTGTTTATATTTTTTTGGGGTTAGAGTGTAAGAAGTAAGAGAGAG

AGAAAAACGAAATTTTTTTTATTTAAAGGAGAATAATAATTGTTTAGGACGTATTATTTT

TTGATTGGTTGTAGTTTATGGTCGAGTTGACGTTTACGGGAAAAATAGAGTATAAGTAGT

CGTAAATATTTTTGGTTTATGCGTAGATTATTTGTTTATTAATTTAGAATATAGGATGTT

AGCGCTATTTTGTGACGGCGAATGTGGGGGCGGTTTTTTATATTTGTTTGTAGTATTTGT

TTGGTTTTTGTAGTTATTGGAATTTATGT

>CRLTRThy7B-R

AAAAGAATAAAAGGGTTTTTAATATTAGAAAAAATTTAAGGTTAAATATATTTATTGGTA

TTTTCGTTTTTTAGTTGAAAAGTTTTGAATTTATATAGTTGAATTTTTTTTAATAGTTTG

TTTTACGGGAATTTTTATCATCGTCGTTTTTTAGTTGATGAGTTTTGAATTCGGTAGTTG

AATTTTTTTTAATAGTTTGTGTTACGGGAATTTTTATAAACGTGATTCGTAGTTTTGGTT

TTGAAATGAAGTATTTTTTTTGCGTTAGTTCGGAGTTTTTTTTCGTTTCGGAATTCGGTA

TTAGTTGTTATTCGTCGCGTTTTTACGACCGGTTAGGAAGAATATAATAATTAGAATTTT

TTACGGTAAAGTTTTATTGTTTATATTTTTTTGGGGTTAGAGTGTAAGAAGTAAGAGAGA

GAGAGAAACGAAATTTTTTTTATTTAAAAGAGAATAATAATTGTTTAGGACGTATTATTT

TTTGATTGGTTGTAGTTTATGGTCGAGTTGACGTTTACGGGAAAAATAGAGTATAAGTAG

TCGTAAATATTTTTGGTTTATGCGTAGATTATTTGTTTATTAATTTAGAATATAGGATGT

TAGCGTTATTTTGTGACGGCGAATGTGGGGGCGGTTTTTTATATTTGTTTGTAGTATTTG

TTTGGTTTTTGTAGTTATTGGAATTTATGT

>CRLTRThy8B-R

AAAAGAATAAAAGGGTTTTTAATATTAGAAAAAATTTAAGGTTAAATATATTTATTGGTA

TTTTCGTTTTTTAGTTGAAAAGTTTTGAATTTATATAGTTGAATTTTTTTTAATAGTTTG

TTTTACGGGAATTTTTATATCGTCGTTTTTTAGTTGATGAGTTTTGAATTCGGTAGTTGA

ATTTTTTTTAATAGTTTGTGTTACGGGAATTTTTATAAACGTGATTCGTAGTTTTGGTTT

TGAAATGAAGTATTTTTTTTGCGTTAGTTTGGAGTTTTTTTTCGTTTCGGAATTCGGTAT

TAATTGTTATTCGTCGCGTTTTTACGATCGGTTAGGAAGAATATAATAATTAGAATTTTT

TACGGTAAAGTTTTATTGTTTATATTTTTTTGGGGTTAGAGTGTAAGAAGTAAGAGAGAG

AGAAAAACGAAATTTTTTTTATTTAAAAGAGAATAATAATTGTTTAGGACGTATTATTTT

TTGATTGGTTGTAGTTTATGGTCGAGTTGACGTTTACGGGAAAATAGAGTATAAGTAGTC

GTAAATATTTTTGGTTTATGCGTAGATTATTTGTTTATTAATTTAGAATATAGGATGTTA

GCGTTATTTTGTGACGGCGAATGTGGGGGCGGTTTTTTATATTTGTTTGTAGTATTTGTT

TGGTTTTTGTAGTTATTGGAATTTATGT
