# Supplementary material for: Epigenetic interplay between mouse endogenous retroviruses and host genes
Source: Genome Biol. 2012 Oct 3;13(10):R89. doi: 10.1186/gb-2012-13-10-r89 (PMC3491417; doi:10.1186/gb-2012-13-10-r89)
Supplement: Additional file 4 — All bisulfite sequencing data. Compilation of all bisulfite sequences. [file gb-2012-13-10-r89-S4.zip › IAP6428_TE_thymus.doc]

CdGAP 3LTR Thymus

B6 Clones

>CLLTRThyMP3-M13R

GGGAAGGAAGTTTTGTTTATATTATAAGTGTGTTTTTGTAAAATAAGGAGTGTTATATGT

TAAGATATGGTGGTTGGTTTTTAAGATAAGTTTTTTAGTTTGGAGGTATTTTTGTTTGTT

TGTTATTTGTTGTGTTTTTATGATTGGTTAGGAAGAATATAATAATTAGAATTTTTTATG

GTAAAGTTTTATTGTTTATATTTTTTTGGGGTTAGAGTGTAAGAAGTAAGAGAGAGAGAA

AAATGAAATTTTTTTTATTTAAAAGAGAATAATAATTGTTTAGGATGTATTATTTTTTGA

TTGGTTGTAGTTTATGGTTGAGTTGATGTTCCTGGGAAAAATAGAGTATAAGTAGTTGTA

AATATTTTTGGTTTATGTGTAGATTATTTGTTTATTAATTTAGAATATAGGATGTTAGTG

TTATTTTGTGATGGTGAATGTGGGGGTGGTTTTTTATAGTTTTTTTTTTTTTTTTTAATA

AGAGTAAATAGGTTATTTATATTAATGAGAGTGGAGATAGAGGTTAAATTTTTAGTGTGT

AGGTAAAGGAGTTATGTATAGGATTAGTTTTTAGGTTTATAGGTTTTTATTTAGAGTAAT

TTTGATTTGTTTTCGTGTCGTTTTGTTTGGGGGAAGGGAATTAGGATATTGAATTTTTAT

GAAAGATGATATGTTTTTTTAGAATAGGTTTATATATGTCGTAGAGTTTTTTTATTGTAG

TGTTTAGTTTTGTAATT

>CLLTRThyMP4-M13R

GGGAAGGAAGTTTTGTTTATATTATAAGTGTGTTTTTGTAAAATAAGGAGTGTTATATGT

TAAGATATGGTGGTTGGTTTCTAAGATAAGTTTTTTAGTTTGGAGGTATTTTTGTTTGTT

TGTTATTTGTTGTGTTTTTATGATTGGTTAGGAAGAATATAATAATTAGAATTTTTTATG

GTAAAGTTTTATTGTTTATATTTTTTTGGGGTTAGAGTGTAAGAAGTAAGAGAGAGAGAA

AAATGAAATTTTTTTTATTTAAAAGAGAATAATAATTGTTTAGGATGTATTATTTTTTGA

TTGGTTGTAGTTTATGGTTGAGTTGATGTTTATGGGAAAAATAGAGTATAAGTAGTTGTA

AATATTTTTGGTTTATGTGTGGATTACTTGTTTATTAATTTAGAATATAGGATGTTAGTG

TCATTTTGTGATGGTGAATGTGGGGGTGGTTTTTTATAGTTTTTTTTTTTTTTTTAATAA

GAGTAAATAGGTTATTTATATTAATGAGAGTGGAGATAGAGGTTAAATTTTTAGTGTGTA

GGTAAAGGAGTTATGTATAGGATTAGTTTTTAGGTTTATAGGTTTTTATTTAGAGTAATT

TTGATTTGTTTTCGTGTTGTTTTGTTTGGGGGAAGGGAATTAGGATATTGAATTTTTATG

AAAGATGATATGTTTTTTTAGAATAGGTTTATATATGTCGTAGAGTTTTTTTATTGTAGT

GTTTAGTTTTGTAATT

>CLLTRThyMP7-M13R

GGGAAGGAAGTTTTGTTTATATTATAAGTGTGTTTTTGTAAAATAGGGAGTGTTATATGT

TAAGATATGGTGGTTGGTTTTTAAGATAAGTTTTTTAGTTTGGAGGTATTTTTGTTTGTT

TGTTATTTGTTGTGTTTTTATGATTGGTTAGGAAGAATATAATAATTAGAATTTTTTATG

GTAAAGTTTTATTGTTTATATTTTTTTGGGGTTAGAGTGTAAGAAGTAAGAGAGAGAGAA

AAATGAAATTTTTTTTATTTAAAAGAGAATAATAATTGTTTAGGATGTATTATTTTTTGA

TTGGTTGTAGTTTATGGTTGAGTTGATGTTTATGGGAAAAATAGAGTATAAGTAGTTGTA

AATATTTTTGGTTTATGTGTAGATTATTTGTTTATTAATTTAGAATATAGGATGTTAGTG

TTATTTTGTGATGGTGAATGTGGGGGTGGTTTTTTATAGTTTTTTTTTTTTTTTTTAATA

AGAGTAAATAGGTTATTTATATTAATGAGAGTGGAGATAGAGGTTAAATTTTTAGTGTGT

AGGTAAAGGAGTTATGTATAGGATTAGTTTTTAGGTTTATAGGTTTTTATTTAGAGTAAT

CTTGATTTGTTTTTGTGTCGTTTTGTTTGGGGGAAGGGAATTAGGATATTGAATTTTTAT

GAAAGATGATATGTTTTTTTAGAATAGGTTTATATACGTCGTAGAGTTTTTTTATTGTAG

TGTTTAGTTTTGTAATT

>CLLTRThyMP8-M13R

GGGAAGGAAGTTTTGTTTATATTATAAGTGCGTTTTTGTAAAATAAGGAGTGTTATATGT

TAAGATATGGTGGTTGGTTTTTAAGATAAGTTTTTTAGTTCGGAGGTATTTTTGTTTGTT

TGTTATTTGTTGTGTTTTTATGATTGGTTAGGAAGAATATAATAATTAGAATTTTTTATG

GTAAAGTTTTATTGTTTATATTTTTTTGGGGTTAGAGTGTAAGAAGTAAGAGAGAGGGAA

AAATGAAATTTTTTTTATTTAAAAGAGAATAATAATTGTTTAGGATGTATTATTTTTTGA

TTGGTTGTAGTTTATGGTTGAGTTGATGTTTATGGGAAAAATAGAGTATAAGTAGTTGTA

AATATTTTTGGTTTATGTGTAGATTATTTGTTTATTAATTTAGAATATAGGATGTTAGTG

TTATTTTGTGATGGTGAATGTGGGGGTGGTTTTTTATAGTTTTTTTTTTTTTTTTAATAA

GAGTAAATAGGTTATTTATATTAATGAGAGTGGAGATAGAGGTTAAATTTTTAGTGTGTA

GGTAAAGGAGTTATGTATAGGATTAGTTTTTAGGTTTATAGGTTTTTATTTAGAGTAATT

TTGATTTGTTTTTGTGTTGTTTTGTTTGGGGGAAGGGAATTAGGCCCATTGAATTTTTAT

GAAAGATGATATGTTTTTTTAGAATAGGTTTATATATGTTGTAGAGTTTTTTTATTGTAG

TGTTTAGTTTTGTAATT

>CLLTRThyMP6-M13R

GGGAAGGAAGTTTTGTTTATATTATAAGTGTGTTTTTGTAAAATAAGGAGTGTTATATGT

TAAGATATGGTGGTTGGTTTTTAAGATAAGTTTTTTAGTTTGGAGGTATTTTTGTTTGTT

TGTTATTTGTTGTGTTTTTATGATTGGTTAGGAAGAATATAATAATTAGAATTTTTTATG

GTAAAGTTTTATTGTTTATATTTTTTTGGGGTTAGAGTGTAAGAAGTAAGAGAGAGAGAA

AAATGAAATTTTTTTTATTTAAAAGAGAATAATAATTGTTTAGGATGTATTATTTTTTGA

TTGGTTGTAGTTTATGGTTGAGTTGATGTTTATGGGAAAAATAGAGTATAAGTAGTTGTA

AATATTTTTGGTTTATGTGTAGATTATTTGTTTATTAATTTAGAATATAGGATGTTAGTG

TTATTTTGTGATGGTGAATGTGGGGGTGGTTTTTTATAGTTTTTTTTTTTTTTTTTAATA

AGAGTAAATAGGTTATTTATATTAATGAGAGTGGAGATAGAGGTTAAATTTTTAGTGTGT

AGGTAAAGGAGTTATGTATAGGATTAGTTTTTAGGTTTATAGGTTTTTATTTAGAGTAAT

TTTGATTTGTTTTTGTGTTGTTTTGTTTGGGGGAAGGGAATTAGGGTATTGAATTTTTAT

GAAAGATGATATGTTTTTTTAGAATAGGTTTATATATGTCGTAGAGTTTTTTTATTGTAG

TGTTTAGTTTTGTAATT

>CLLTRThyMP9-M13R

GGGAAGGAAGTTTTGTTTATATTATAAGTGTGTTTTTGTAAAATAAGGAGTGTTATATGT

TAAGATGTGGTGGTTGGTTTTTAAGATAAGTTTTTTAGTTTGGAGGTATTTTTGTTTGTT

TGTTATTTGTTGTGTTTTTATGATTGGTTAGGAAGAATATAATAATTAGAATTTTTTATG

GTAAAGTTTTATTGTTTATATTTTTTTGGGGTTAGAGTGTAAGAAGTAAGAGAGAGAGAA

AAATGAAATTTTTTTTATTTAAAAGAGAATAATAATTGTTTAGGATGTATTATTTTTTGA

TTGGTTGTAGTTTATGGTTGAGTTGATGTTTACGGGAAAAATAGAGTATAAGTAGTTGTA

AATATTTTTGGTTTATGTGTAGATTATTTGTTTATTAATTTAGAATATAGGATGTTAGTG

TTATTTTGTGATGGTGAATGTGGGGGTGGTTTTTTATAGTTTTTTTTTTTTTTTTAATAA

GAGTAAATAGGTTATTTATATTAATGAGAGTGGAGATAGAGGTTAAATTTTTAGTGTGTA

GGTAAAGGAGTTATGTATAGGATTAGTTTTTAGGTTTATAGGTTTTTATTTAGAGTAATT

TTGATTTGTTTTTGTGTTGTTTTGTTTGGGGGAAGGGAATTAGGATATTGAATTTTTATG

AAAGATGATATGTTTTTTTAGAATAGGTTTATATATGTCGTAGAGTTTTTTTATTGTAGT

GTTTAGTTTTGTAATT

>CLLTRThyMP10-M13R

GGGAAGGAAGTTTTGTTTATATTATAAGTGTGTTTTTGTAAAATAAGGAGTGTTATATGT

TAAGATATGGTGGTTGGTTTTTAAGATAAGTTTTTTAGTTTGGAGGTATTTTTGTTTGTT

TGTTATTTGTTGTGTTTTTATGATTGGTTAGGAAGAATATAATAATTAGAATTTTTTATG

GTAAAGTTTTATTGTTTATATTTTTTTGGGGTTAGAGTGTAAGAAGTAAGAGAGAGAGAA

AAATGAAATTTTTTTTATTTAAAAGAGAATAATAATTGTTTAGGATGTATTATTTTTTGA

TTGGTTGTAGTTTATGGTTGAGTTGATGTTTATGGGAAAAATAGAGTATAAGTAGTTGTA

AATATTTTTGGTTTATGTGTAGATTATTTGTTTATTAATTTAGAATATAGGATGTTAGTG

TTATTTTGTGATGGTGAATGTGGGGGTGGTTTTTTATAGTTTTTTTTTTTTTTTTTAATA

AGAGTAAATAGGTTATTTATATTAATGAGAGTGGAGATAGAGGTTAAATTTTTAGTGTGT

AGGTAAAGGAGTTATGTATAGGATTAGTTTTTAGGTTTATAGGTTTTTATTTAGAGTAAT

TTTGATTTGTTTTTGTGTTGTTTTGTTTGGGGGAAGGGAATTAGGATATTGAATTTTTAT

GAAAGATGATATGTTTTTTTAGAATAGGTTTATATATGTCGTAGAGTTTTTTTATTGTAG

TGTTTAGTTTTGTAATT

“B” Miniprep Sequences

B6 Clones

>CLLTRThy1B-R

GGGAAGGAAGTTTTGTTTATATTATAAGTGTGTTTTTGTAAAATAAGGAGTGCTATATGT

TAAGATATGGTGGTTGGTTTTTAAGATAAGTTTTTTAGTTTGGAGGTATTTTTGTTTGTT

TGTTATTTGTTGTGTTTTTATGATTGGTTAGGAAGAATATAATAATTAGAATTTTTTATG

GTAAAGTTTTATTGTTTATATTTTTTTGGGGTTAGAGTGTAAGAAGTAAGAGAGAGAGAA

AAATGAAATTTTTTTTATTTAAAAGAGAATAATAATTGTTTAGGATGTATTATTTTTTGA

TTGGTTGTAGTTTATGGTTGAGTTGATGTTTCTGGGAAAAATAGAGTATAAGTAGTTGTA

AATATTTTTGGTTTATGTGTAGATTATTTGTTTATTAATTTAGAATATAGGATGTTAGTG

TTATTTTGTGATGGTGAATGTGGGGGTGGTTTTTTATAGTTTTTTTTTTTTTTTAATAAG

AGTAAATAGGTTATTTATATTAATGAGAGTGGAGATAGAGGTTAAATTTTTAGTGTGTAG

GTAAAGGAGTTATGTATAGGATTAGTTTTTAGGTTTATAGGTTTTTATTTAGAGTAATTT

TGATTTGTTTTCGTGTCGTTTTGTTTGGGGGAAGGGAATTAGGATATTGAATTTTTATGA

AAGATGATATGTTTTTTTAGAATAGGTTTATATATGTCGTAGAGTTTTTTTATTGTAGTG

TTTAGTTTTGTAATT

>CLLTRThy2B-R

GGGAAGGAAGTTTTGTTTATATTATAAGTGTGTTTTTGTAAAATAAGGAGTGTTATATGT

TAAGATATGGTGGTTGGTTTTTAAGATAAGTTTTTTAGTTTGGAGGTATTTTTGTTTGTT

TGTTATTTGTTGTGTTTTTATGATTGGTTAGGAAGAATATAATAATTAGAATTTTTTATG

GTAAAGTTTTATTGTTTATATTTTTTTGGGGTTAGAGTGTAAGAAGTAAGAGAGAGAGAA

AAATGAAATTTTTTTTATTTAAAAGAGAATAATAATTGTTTAGGATGTATTATTTTTTGA

TTGGTTGTAGTTTATGGTTGAGTTGATGTTTATGGGAAAAATAGAGTATAAGTAGTTGTA

AATATTTTTGGTTTATGTGTAGATTATTTGTTTATTAATTTAGAATATAGGATGTTAGTG

TTATTTTGTGATGGTGAATGTGGGGGTGGTTTTTTATAGTTTTTTTTTTTTTTTTTAATA

AGAGTAAATAGGTTATTTATATTAATGAGAGTGGAGATAGAGGTTAAATTTTTAGTGTGT

AGGTAAAGGAGTTATGTATAGGATTAGTTTTTAGGTTTATAGGTTTTTATTTAGAGTAAT

TTTGATTTGTTTTTGTGTTGTTTTGTTTGGGGGAAGGGAATTAGGGTATTGAATTTTTAT

GAAAGATGATATGTTTTTTTAGAATAGGTTTATATATGTCGTAGAGTTTTTTTATTGTAG

TGTTTAGTTTTGTAATT

>CLLTRThy3B-R

GGGAAGGAAGTTTTGTTTATATTATAAGTGCGTTTTCGTAAAATAAGGAGTGTTATACGT

TAAGATACGGCGGTTGGTTTTTAAGATAAGTTTTTTAGTTTGGAGGTATTTTTGTTTGTT

TGTTATTCGTCGCGTTTTTACGATCGGTTAGGAAGAATATAATAATTAGAATTTTTTACG

GTAAAGTTTTATTGTTTATATTTTTTTGGGGTTAGAGTGTAAGAAGTAAGAGAGAGAGAA

AAACGAAATTTTTTTTATTTAAAAGAGAATAATAATTGTTTAGGACGTATTATTTTTTGA

TTGGTTGTAGTTTATGGTTGAGTTGACGTTTACGGGAAAAATAGAGTATAAGTAGTTGTA

AATATTTTTGGTTTATGTGTAGATTATTTGTTTATTAATTTAGAATATAGGATGTTAGCG

TTATTTTGTGATGGTGAATGTGGGGGCGGTTTTTTATAGTTTTTTTTTTTTTTTTTAATA

AGAGTAAATAGGTTATTTATATTAATGAGAGTGGAGATAGAGGTTAAATTTTTAGTGTGT

AGGTAAAGGAGTTATGTATAGGATTAGTTTTTAGGTTTATAGGTTTTTATTTAGAGTAAT

TTTGATTTGTTTTCGTGTCGTTTTGTTTGGGGGAAGGGAATTAGGATATTGAATTTTTAT

GAAAGATGATATGTTTTTTTAGAATAGGTTTATATATGTCGTAGAGTTTTTTTATTGTAG

TGTTTAGTTTTGTAATT

>CLLTRThy4B-R

GGGAAGGAAGTTTTGTTTATATTATAAGTGTGTTTTTGTAAAGTAAGGAGTGTTATATGT

TAAGATATGGTGGTTGGTTTTTAAGATAAGTTTTTTAGTTTGGAGGTATTTTTGTTTGTT

TGTTATTTGTTGTGTTTTTATGATCGGTTAGGAAGAATATAATAATTAGAATTTTTTATG

GTAAAGTTTTATTGTTTATATTTTTTTGGGGTTAGAGTGTAAGAAGTAAGAGAGAGAGAA

AAATGAAATTTTTTTTATTTAGAAGAGAATAATAATTGTTTAGGATGTATTATTTCTTGA

TTGGTTGTAGTTTATGGTTGAGTTGATGTTTATGGGAAAAATAGAGTATAAGTAGTTGTA

AATATTTTTGGTTTATGTGTAGATTATTTGTTTATTAATTTAGAATATAGGATGTTAGCG

TTATTTTGTGATGGTGAATGTGGGGGCGGTTTTTTATAGTTTTTTTTTTTTTTTTAATAA

GAGTAAATAGGTTATTTATATTAATGAGAGTGGAGATAGAGGTTAAATTTTTAGTGTGTG

GGTAAAGGAGTCATGTATAGGATTAGTTTTTAGGTTTATAGGTTTTTATTTAGAGTAATT

TTGATTTGTTTTTGTGTCGTTTTGTTTGGGGGAAGGGAATTAGGATATTGAATTTTTATG

AAAGATGATATGTTTTTTTAGAATAGGTTTATATATGTCGTAGAGTTTTTTTATTGTAGT

GTTTAGTTTTGTAATT

>CLLTRThy5B-R

GGGAAGGAAGTTTTGTTTATATTATAAGTGTGTTTTTGTAAAATAAGGAGTGTTATATGT

TAAGATATGGTGGTTGGTTTTTAAGATAAGTTTTTTAGTTTGGAGGTATTTTTGTTTGTT

TGTTATTTGTTGTGTTTTTATGATTGGTTAGGAAGAATATAATAATTAGAATTTTTTATG

GTAAAGTTTTATTGTTTATATTTTTTTGGGGTTAGAGTGTAAGAAGTAAGAGAGAGAGAA

AAATGAAATTTTTTTTATTTAAAAGAGAATAATAATTGTTTAGGATGTATTATTTTTTGA

TTGGTTGTAGTTTATGGTTGAGTTGATGTTTATGGGAAAAATAGAGTATAAGTAGTTGTA

AATATTTTTGGTTTATGTGTAGATTATTTGTTTATTAATTTAGAATATAGGATGTTAGTG

TTATTTTGTGATGGTGAATGTGGGGGTGGTTTTTTATAGTTTTTTTTTTTTTTTTTAATA

AGAGTAAATAGGTTATTTATATTAATGAGAGTGGAGATAGAGGTTAAATTTTTAGTGTGT

AGGTAAAGGAGTTATGTATAGGATTAGTTTTTAGGTTTATAGGTTTTTATTTAGAGTAAT

TTTGATTTGTTTTTGTGTTGTTTTGTTTGGGGGAAGGGAATTAGGATATTGAATTTTTAT

GAAAGATGATATGTTTTTTTAGAATAGGTTTATATATGTCGTAGAGTTTTTTTATTGTAG

TGTTTAGTTTTGTAATT

>CLLTRThy6B-R

GGGAAGGAAGTTTTGTTTATATTATAAGTGTGTTTTTGTAAAATAAGGAGTGTTATATGT

TAAGATATGGTGGTTGGTTTTTAAGATAAGTTTTTTAGTTTGGAGGTATTTTTGTTTGTT

TGTTATTTGTTGTGTTTTTATGATTGGTTAGGAAGAATATAATAATTAGAATTTTTTATG

GTAAAGTTTTATTGTTTATATTTTTTTGGGGTTAGAGTGTAAGAAGTAAGAGAGAGAGAA

AAATGAAATTTTTTTTATTTAAAAGAGAATAATAATTGTTTAGGATGTATTATTTTTTGA

TTGGTTGTAGTTTATGGTTGAGTTGATGTTTATGGGAAAAATAGAGTATAAGTAGTTGTA

AATATTTTTGGTTTATGTGTAGATTATTTGTTTATTAATTTAGAATATAGGATGTTAGTG

TTATTTTGTGATGGTGAATGTGGGGGTGGTTTTTTATAGTTTTTTTTTTTTTTTTTAATA

AGAGTAAATAGGTTATTTATATTAATGAGAGTGGAGATAGAGGTTAAATTTTTAGTGTGT

AGGTAAAGGAGTTATGTATAGGATTAGTTTTTAGGTTTATAGGTTTTTATTTAGAGTAAT

TTTGATTTGTTTTTGTGTTGTTTTGTTTGGGGGAAGGGAATTAGGATATTGAATTTTTAT

GAAAGATGATATGTTTTTTTAGAATAGGTTTATATATGTCGTAGAGTTTTTTTATTGTAG

TGTTTAGTTTTGTAATT

>CLLTRThy7B-R

GGGAAGGAAGTTTTGTTTATATTATAAGTGTGTTTTTGTAAAATAAGGAGTGTTATATGT

TAAGATATGGTGGTTGGTTTTTAAGATAAGTTTTTTAGTTTGGAGGTATTTTTGTTTGTT

TGTTATTCGTTGTGTTTTTATGATTGGTTAGGAAGAATATAATAATTAGAATTTTTTATG

GTAAAGTTTTATTGTTTATATTTTTTTGGGGTTAGAGTGTAAGAAGTAAGAGAGAGAGAA

AAATGAAATTTTTTTTATTTAAAAGAGAATAATAATTGTTTAGGATGTATTATTTTTTGA

TTGGTTGTAGTTTATGGTTGAGTTGATGTTTATGGGAAAAATAGAGTATAAGTAGTTGTA

AATATTTTTGGTTTATGTGTAGATTATTTGTTTATTAATTTAGAATATAGGATGTTAGTG

TTATTTTGTAATGGTGAATGTGGGGGTGGTTTCTTATAGTTTTTTTTTTTTTTTTAATAA

GAGTAAATAGGTTATTTATATTAATGAGAGTGGAGATAGAGGTTAAATTTTTAGTGTGTA

GGTAAAGGAGTTATGTATAGGATTAGTTTTTAGGTTTATAGGTTTTTATTTAGAGTAATT

TTGATTTGTTTTCGTGTTGTTTTGTTTGGGGGAAGGGAATTAGGATATTGAATTTTTATG

AAAGATGATATGTTTTTTTAGAATAGGTTTATATATGTCGTAGAGTTTTTTTATTGTAGT

GTTTAGTTTTGTAATT

>GAPLLTRThy-1R

GGGAAGGAAGTTTTGTTTATATTATAAGTGTGTTTTTGTAAAATAAGGAGTGTTATATGT

TAAGATATGGTGGTTGGTTTTTAAGATAAGTTTTTTAGTTTGGAGGTATTTTTGTTTGTT

TGTTATTTGTTGTGTTTTTATGATTGGTTAGGAAGAATATAATAATTAGAATTTTTTATG

GTAAAGTTTTATTGTTTATATTTTTTTGGGGTTAGAGTGTAAGAAGTAAGAGAGAGAGAA

AAATGAAATTTTTTTTATTTAAAAGAGAATAATAATTGTTTAGGATGTATTATTTTTTGA

TTGGTTGTAGTTTATGGTTGAGTTGATGTTTATGGGAAAAATAGAGTATAAGTAGTTGTA

AATATCCTTGGTTTATGTGTAGATTATTTGTTTATTAATTTAGAATATAGGATGTTAGTG

TTATTTTGTGATGGTGAATGTGGGGGTGGTTTTTTATAGTTTTTTTTTTTTTTTTTAATA

AGAGTAAATAGGTTATTTATATTAATGAGAGTGGAGATAGAGGTTAAATTTTTAGTGTGT

AGGTAAAGGAGTTATGTATAGGATTAGTTTTTAGGTTTATAGGTTTTTATTTAGAGTAAT

TTTGATTTGTTTTTGTGTTGTTTTGTTTGGGGGAAGGGAATTAGGATATTGAATTTTTAT

GAAAGATGATATGTTTTTTTAGAATAGGTTTATATATGTCGTAGAGTTTTTTTATTGTAG

TGTTTAGTTTTGTAATT

>GAPLLTRThy-2R

GGGAAGGAAGTTTTGTTTATATTATAAGTGCCGTTTTCGTAAAATAAGGAGTGTTATACC

GTTAAGATACCGGTGGTTGGTTTTTAAGATAAGTTTTTTAGTTTGGAGGTATTTTTGTTT

GTTTGTTATTCGTCGCGTTTTTACGATCGGTTAGGAAGAATATAATAATTAGAATTTTTT

ATGGTAAAGTTTTATNGTTTATATTTTTTTGGGGTTAGAGTGTAAGAAGTAAGAGAGAGA

GAAAAATGAAATTTTTTTTATTTAAAAGAGAATAATAATTGTTTAGGATGTATTATTTTT

TGATTGGTTGTAGTTTATGGTTGAGTTGATGTTTATGGGAAAAATAGAGTATAAGTAGTT

GTAAATATTTTTGGTTTATGTGTAGATTATTTGTTTATTAATTTAGAATATAGGATGTTA

GCGTTATTTTGTGACGGCGAATGTGGGGGCGGTTTTTTATAGTTTTTTTTTTTTTTTTTT

AATAAGAGTAAATAGGTTATTTATATTAATGAGAGTGGAGATAGAGGTTAAATTTTTAGT

GTGTAGGTAAAGGAGTTATGTATAGGATTAGTTTTTAGGTTTATAGGTTTTTATTTAGAG

TAATTTTGATTTGTTTTTGTGTCGTTTTGTTTGGGGGAAGGGAATTAGGATATTGAATTT

TTATGAAAGATGATATGTTTTTTTAGAATAGGTTTATATATGTCGTAGAGTTTTTTTATT

GTAGTGTTTAGTTTTGTAATT

>GAPLLTRThy-3R

GGGAAGGAAGTTTTGTTTATATTATAAGTGTGTTTTTGTAAAATAAGGAGTGTTATATGT

TAAGATATGGTGGTTGGTTTTTAAGATAAGTTTTTTAGTTTGGAGGTATTTTTGTTTGTT

TGTTATTTGTTGTGTTTTTATGATTGGTTAGGAAGAATATAATAATTAGAATTTTTTATG

GTAAAGTTTTATTGTTTATATTTTTTTGGGGTTAGAGTGTAAGAAGTAAGAGAGAGAGAA

AAATGAAATTTTTTTTATTTAAAAGAGAATAATAATTGTTTAGGATGTATTATTTTTTGA

TTGGCTGTAGTTTATGGTTGAGTTGATATTTATGGGAAAAATAGAGTATAAGTAGTCGTA

AATATTTTTGGTTTATGTGTAGACTATTTGTTTATTAATTTAGAATACCGGATGTTAGTG

TTATTTTGTGATGGCGAATGTGGGGGTGGTTTTTCATAGCTTTTTTTTTTTTCTTAATAA

GAGTAAATAGGTTATTTATATTAATGAGAGTGGAGATAGAGGTTAAATTCTTAGTGTGTA

GGTAAAGGAGTTATGTATAGGATTAGTTTTTAGGTTTATAGGTTTTTATTCAGAGTAATT

CTGATTTGTTTTTGTGTTGTTTTGTTTGGGGGAAGGGAATTAGGATATTGAATTTTTATG

AAAGATGATATGTTTTTTTAGAATAGGTTTATATATGTCGTAGAGTTTTTTTATCGTAGT

GTTTAGTTTTGTAATT

>GAPLLTRThy-5R

GGGAAGGAAGTTTTGTTTATATTATAAGTGTGTTTTCGTAAAATAAGGAGTGTTATATGT

TAAGATATGGTGGTTGGTTTTTAAGATAAGTTTTTTAGTTTGGAGGTATTTTTGTTTGTT

TGTTATTTGTCGCGTTTTTACGATCGGTTAGGAAGAATATAATAATTAGAATTTTTTATG

GTAAAGTTTTATTGTTTATATTTTTTTGGGGTTAGAGTGTAAGAAGTAAGAGAGAGAGAA

AAATGAAATTTTTTTTATTTAAAAGAGAATAATAATTGTTTAGGACGTATTATTTTTTGA

TTGGTTGTAGTTTATGGTTGAGTTGATGTTTATGGGAAAAATAGAGTATAAGTAGTTGTA

AATATTTTTGGTTTATGTGTAGATTATTTGTTTATTAATTTAGAATACAGGATGTTAGTG

TTATTTTGTGATGGTGAATGTGGGGGTGGTTTTTTATAGTTTTTTTTTTTTTTTTTAATA

AGAGTAAATAGGTTATTTATATTAATGAGAGTGGAGATAGAGGTTAAATTTTTAGTATGT

AGGTAAAGGAGTTATGTATAGGATTAGTTTTTAGGTTTATAGGTTTTTATTTAGAGTAAT

TTTGATTTGTTTTTGTGTCGTCTTGTTTGGGGGAAGGGAATTAGGATATTGAATTTTTAT

GAAAGATGATATGTTTTTTTAGAATAGGTTTATATATGTCGTAGAGTTTTTTTATTGTAG

TGTTTAGTTTTGTAATT

>GAPLLTRThy-9R

GGGAAGGAAGTTTTGTTTATATCATAAGTGCGTTTTTGTAAAATAAGGAGTGTTATACGT

TAAGATACGGCGGTTGGTTTTTAAGATAAGTTTTTTAGTTTGGAGGTATTTTTGTTTGTT

TGTTATTCGTCGCGTTTTTACGATCGGTTAGGAAGAATATAATAATTAGAATTTTTTATG

GTAAAGTTTTATTGTTTATATTTTTTTGGGGTTAGAGTGTAAGAAGTAAGAGAGAGAAAA

AAATGAAATTTTTTTTATTTAAAAGAGAATAATAATTGTTTAGGATGCCATTATTTTTTG

ATTGGTTGTAGTTTATGGTTGAGTTGATGTTTATGGGAAAAATAGAGTATAAGTAGTTGT

AAATATTTTTGGTTTATGTGTAGATTATTTGTTTATTAATTTAGAATATAGGATGTTAGT

GTTATTTTGTGATGGTGAATGTGGGGGTGGTTTTTTATAGTTTTTTTTTTTTTTTTTAAT

AAGAGTAAATAGGTTATTTATATTAATGAGAGTGGAGATAGAGGTTAAATTTTTAGTGTG

TAGGTAAAGGAGTTATGTATAGGATTAGTTTTTAGGTTTATAGGTTTTTATTTAGAGTAA

TTTTGATTTGTTTTCGTGTTGTTTTGTTTGGGGGAAGGGAATTAGGATATTGAATTTTTA

TGAAAGATGATATGTTTTTTTAGAATAGGTTTATATATGTCGTAGAGTTTTTTTATTGTA

GTGTCTAGTTTTGTAATT

>GAPLLTRThy-11R

GGGAAGGAAGTTTTGTTTATATTATAAGTGTGTTTTTGTAAAATAAGGAGTGTTATATGT

TAAGATATGGTGGTTGGTTTTTAAGATAAGTTTTTTAGTTCGGAGGTATTTTTGTTTGTT

TGTTATTTGTTGTGTTTTTATGATTGGTTAGGAAGAATATAATAATTAGAATTTTTTATG

GTGAAGTTTTATTGTTTATATTTTTTTGGGGTTAGAGTGTAAGAAGTAAGAGAGAGAGAA

AAATGAAATTTTTTTATTTAAAAGAGAATAATAATTGTTTAGGATGTATTATTTTTTGAT

TGGTTGTAGTTTATGGTTGAGTTGATGTTTATGGGAAAAATAGAGTATAAGTAGTTGTAA

ATATTTTTGGTTTATGTGTAGATTATTTGTTTATTAATTTAGAATATAGGATGTTAGTGT

TATTTTGTGATGGTGAATGTGGGGGTGGTTTTTTATAGTTTTTTTTTTTTTTTTTAATAA

GAGTAAATAGGTTATTTATATTAATGAGAGTGGAGATAGAGGTTAAATTTTTAGTGTGTA

GGTAAAGGAGTTATGTATAGGATTAGTTTTTAGGTTTATAGGTTTTTATTTAGAGTAATT

TTGATTTGTTTTCGTGTCGTTTTGTTTGGGGGAAGGGAATTAGGATATTGAATTTTTATG

AAAGATGATATGTTTTTTTAGAATAGGTTTATATATGTTGTAGAGTTTTTTATTGTAGTG

TTTAGTTTTGTAAT

>GAPLLTRThy-12R

GGGAAGGAAGTTTTGTTTATATTATAAGTGTGTTTTTGTAAAATAAGGAGTGTTATATGT

TAAGATATGGTGGTTGGTTTTTAAGATAAGTTTTTTAGTTCGGAGGTATTTTTGTTTGTT

TGTTATTTGTTGTGTTTTTATGATTGGTTAGGAAGAATATAATAATTAGAATTTTTTATG

GTAAAGTTTTATTGTTTATATTTTTTTGGGGTTAGAGTGTAAGAAGTAAGAGAGAGAGAA

AAATGAAATTTTTTTTATTTAAAAGAGAATAATAATTGTTTAGGATGTATTATTTTTTGA

TTGGTTGTAGTTTATGGTTGAGTTGATGTTTATGGGAAAAATAGAGTATAAGTAGTTGTA

AATATTTTTGGTTTATGTGTAGATTATTTGTTTATTAATTTAGAATATAGGATGTTAGTG

TTATTTTGTGATGGTGAATGTGGGGGTGGTTTTTTATAGTTTTTTTTTTTTTTTTTAATA

AGAGTAAATAGGTTATTTATATTAATGAGAGTGGAGATAGAGGTTAAATTTTTAGTGTGT

AGGTAAAGGAGTTATGTATAGGATTAGTTTTTAGGTTTATAGGTTTTTATTTAGAGTAAT

TTTGATTTGTTTTTGTGTTGTTTTGTTTGGGGGAAGGGAATTAGGATATTGAATTTTTAT

GAAACGATGATATGTTTTTTTAGAATAGGTTTATATATGTCGTAGAGTTTTTTTATTGTA

GTGTTTAGTTTTGTAATT
