# Supplementary material for: Epigenetic interplay between mouse endogenous retroviruses and host genes
Source: Genome Biol. 2012 Oct 3;13(10):R89. doi: 10.1186/gb-2012-13-10-r89 (PMC3491417; doi:10.1186/gb-2012-13-10-r89)
Supplement: Additional file 4 — All bisulfite sequencing data. Compilation of all bisulfite sequences. [file gb-2012-13-10-r89-S4.zip › IAP8545_gene_thymus.doc]

B6 Clones

>NPnpThy_30

AGGGTTTTTTTTAAATTTATATATTTTTTTTAAGGTAGTACTTATTTTTTATTGATAAGG

GTGGGGGAATAAAAGTTGATTGAAAGAAATGTATTTTGGTGGTTTGATTTTGGTAATTAT

AGAAGGTTTGTAAGGATTGTTTTTTTGGTAGTTTGTAGGTTTTATGGAAATGAAATTGTG

TTTGATTTTGTTTATAGGAGTGTTGGTTAATGAGGTGTTGTTTAGGTTTTTTATGCTTTG

GGTGTTATGGTGGTTTGTAGGTTGTGTTGTTTGTGTTTGTGTTTTTGATTGTTGGGTTGT

GGTTTTTTGGTTGGTTTGGGTGGAATNGGGTATTTAGTTATTTGTAGATGTGAGTGTTGT

GGAGTAGTATTGGATTTAGAGTGGTGATTGTAGATTTGGGATATAGGTGAGA

>NPnpThy_5

AGGGTTTTTTTTAAATTTATATATTTTTTTTAAGGTAGTATTTATTTTTTATTGATAAGG

GTGGGGGAATAAAAGTTGATTGAAAGAAATGTATTTTGGTGGTTTGATTTTGGTAATTAT

AGAAAGTTTGTAAGGATTGTTTTTTTGGTAGTTTGTAGGTTTTATGGAAATGAAATTGTG

TTTGATTTTGTTTATAGGAGTGTTGGTTAATGAGGTGTTGTTTAGGTTTTTTATGTTCTG

GGTGTTATGGTGGTTTGTAGGTTGTGTTGTTTGTGTTTGTGTTTTTGATTGTTGGGTTGT

GGTTTTTTAGGTTGGTTTGGGTGGAATTGGGTATTTAGTTATTTGTAGATGTGAGTGTTG

TGGAGTAGTATTGGATTTAGAGTGGTGATTGTAGATTTGGGATATAGGTGAGA

>NPnpThy_29

AGGGTTTTTTTTAAATTTATATATTTTTTTTAAGGTAGTATTTATTTTTTATTGATAAGG

GTGGGGGAATAAAAGTTGATTGAAAGAAATGTATTTTGGTGGTTTGATTTTGGTAATTAT

AGAAAGTTTGTAAGGATTGTTTTTTTGGTAGTTTGTAGGTTTTATGGAAATGAAATTGTG

TTTGATTTTGTTTATAGGAGTGTTGGTTAATGAGGTGTTGTTTAGGTTTTTTATGTTTTG

GGTGTTATGGTGGTTTGTAGGTTGTGTTGTTTGTGTTTGTGTTTTTGATTGTTGGGTTGT

GGTTTTTTTGGTTGGTTTGGGTGGAATTGGGTATTTAGTTATTTGTAGATGTGAGTGTTG

TGGAGTAGTATTGGATTTAGAGTGGTGATTGTAGATTTGGGATATAGGTGAGA

>NPnpThy_9

AGGGTTTTTTTTAAATTTATATATTTTTTTTAAGGTAGTATTTATTTTTTATTGATAAGG

GTGGGGGAATAAAAGTTGATTGAAAGAAATGTATTTTGGTGGTTTGATTTTGGTAATTAT

AGAAAGTTTGTAAGGATTGTTTTTTTGGTAGTTTGTAGGTTTTATGGAAATGAAATTGTG

TTTGATTTTGTTTATAGGAGTGTTGGTTAATGAGGTGTTGTTTAGGTTTTTTATGTTTTG

GGTGTTATGGTGGTTTGTAGGTTGTGTTGTTTGTGCTTGTGTTTTTGATTGTTGGGTTGT

CGTTTTTTTGGTTGGTTTGGGTGGAATTGGGTATTTAGTTATTTGTAGATGTGAGTGTTG

TGGAGTAGTATTGGATTTAGAGTGGTGATTGTAGATTTGGGATATAGGTGAGA

>NPnpThy_11

AGGGTTTTTTTTAAATTTATATATTTTTTTTAAGGTAGTATTTATTTTTTATTGATAAGG

GTGGGGGAATAAAAGTTGATTGAAAGAAATGTATTTTGGTGGTTTGATTTTGGTAATTAT

AGAAAGTTTGTAAGGATTGTTTTTTTGGTAGTTTGTAGGTTTTATGGAAATGAAATTGTG

TTTGATTTTGTTTATAGGAGTGTTGGTTAATGAGGTGTTGTTTAGGTTTTTTATGTTTTG

GGTGTTATGGTGGTTTGTAGGTTGTGTTGTTTGTGTTTGTGTTTTTGATTGTTGGGTTGT

GGTTTTTTTGGTTGGTTTGGGTGGAATTGGGTATTTAGTTATTTGTAGATGTGAGTGTTG

TGGAGTAGTATTGGATTTAGAGTGGTGATTGTAGATTTGGGATATAGGTGAGA

>NPnpThy_12

AGGGTTTTTTTTAAATTTATATATTTTTTTTAAGGTAGTATTTATTTTTTATTGATAAGG

GTGGGGGAATAAAAGTTGATTGAAAGAAATGTATTTTGGTGGTTTGATTTTGGTAATTAT

AGAAAGTTTGTAAGGATTGTTTTTTTGGTAGTTTGTAGGTTTTATGGAAATGAAATTGTG

TTTGATTTTGTTTATAGGAGTGTTGGTTAATGAGGTGTTGTTTAGGTTTTTTATGTTTTG

GGTGTTATGGTGGTTTGTAGGTTGTGTTGTTTGTGTTTGTGTTTTTGATTGTTGGGTTGT

GGTTTTTTTGGTTGGTTTGGGTGGAATTGGGTATTTAGTTATTTGTAGATGTGAGTGTTG

TGGAGTACGTATTGGATTTAGAGTGGTGATTGTAGATTTGGGATATAGGTGAGA

>NPnpThy_15

AGGGTTTTTTTTAAATTTATATATTTTTTTTAAGGTAGTATTTATTTTTTGTTGATAAGG

GTGGGGGAATAAAAGTTGATTGAAAGAAATGTATTTTGGTGGTTTGACTTTGGTAATTAT

AGAAAGTTTGTAAGGATTGTTTTTTTGGTAGTTTGTAGGTTTTATGGAAATGAAATTGTG

TTTGATTTTGTTTATAGGAGTGTTGGTTAATGAGGTGTTGTTTAGGTTTTTTATGTTTTG

GGTGTTATGGTGGTTTGTAGGTTGTGTTGTTTGTGTTTGTGTTTTTGATTGTTGGGTTGT

GGTTTTTTAGGTTGGTTTGGGTGGAATTGGGTATTTAGTTATTTGTAGATGTGAGTGTTG

TGGAGTAGTATTGGATTTAGAGTGGTGATTGTAGATTTGGGATATAGGTGAGA

>NPnpThy_17

AGGGTTTTTTTTAAATTTATATATTTTTTTTAAGGTAGTATTTATTTTTTATTGATAAGG

GTGGGGGAATAAAAGTTGATTGAAAGAAATGTATTTTGGTGGTTTGATTTTGGTAATTAT

AGAAAGTTTGTAAGGATTGTTTTTTTGGTAGTTTGTAGGTTTTATGGAAATGAAATTGTG

TTTGATTTTGTTTATAGGAGTGTTGGTTAATGAGGTGTTGTCTAGGTTTTTTATGTTTTG

GGTGTTATGGTGGTTTGTAGGTTGTGTTGTTTGTGTTTGTGTTTTCGATTGTTGGGTTGT

GGCTTTTTTGGTTGGTTTGGGTGGAATTGGGTATTTAGTTATTTGTAGATGTGAGTGTTG

TGGAGTAGTATTGGATTTAGAGTGGTGATTGTAGATTTGGGATATAGGTGAGA

>NPnpThy_19

AGGGTTTTTTTTAAATTTATATATTTTTTTTAAGGTAGTATTTATTTTTTATTGATAAGG

GTGGGGGAATAAAAGTTGATTGAAAGAAATGTATTTTGGTGGTTTGATTTTGGTAATTAT

AGAAAGTTTGTAAGGATTGTTTTTTTGGTAGTTTGTAGGTTTTATGGAAATGAAATTGTG

TTTGATTTTGTTTATAGGAGTGTTGGTTAATGAGGTGTTGTTTAGGTTTTTTATGTTTTG

GGTGTTATGGTGGTTTGTAGGTTGTGTTGTTTGTGTTTGTGTTTTTGATTGTTGGGTTGT

GGTTTTTTTGGTTGGTTTGGGTGGAATTGGGTATTTAGTTATTTGTAGATGTGAGTGTTG

TGGAGTAGTATTGGATTTAGAGTGGTGATTGTAGATTTGGGATATAGGTGAGA

>NPnpThy_20

AGGGTTTTTTTTAAATTTATATATTTTTTTTAAGGTAGTATTTATTTTTTATTGATAAGG

GTGGGGGAATAAAAGTTGATTGAAAGAAATGTATTTTGGTGGTTTGATTTTGGTAATTAT

AGAAAGTTTGTAAGGATTGTTTTTTTGGTAGTTTGTAGGTTTTATGGAAATGAAATTGTG

TTTGATTTTGTTTATAGGAGTGTTGGTTAATGAGGTGTTGTTTAGGTTTTTTATGTTTTG

GGTGTTATGGTGGTTTGTAGGTTGTGTTGTTTGTGTTTGTGTTTTTGATTGTTGGGTTGT

GGTTTTTTAGGTTGGTTTGGGTGGAATTGGGTATTTAGTTATTTGTAGATGTGAGTGTTG

TGGAGTAGTATTGGATTTAGAGTGGTGATTGTAGATTTGGGATATAGGTGAGA

>NPnpThy_22

AGGGTTTTTTTTAAATTTATATATTTTTTTTAAGGTAGTATTTATTTTTTATTGATAAGG

GTGGGGGAATAAAAGTTGATTGAAAGAAATGTATTTTGGTGGTTTGATTTTGGTAATTAT

AGAAAGTTTGTAAGGATTGTTTTTTTGGTAGTTTGTAGGTTTTATGGAAACGAAATTGTG

TTTGATTTTGTTTATAGGAGTGTTGGTTAATGAGGTGTTGTTTAGGTTTTTTATGTTTTG

GGTGTTATGGTGGTTTGTAGGTTGTGTTGTTTGTGTTTGTGTTTTTGATTGTTGGGTTGT

GGTTTTTTAGGTTGGTTTGGGTGGAATTGGGTATTTAGTTATTTGTAGATGTGACGTGTT

GTGGAGTAGTATTGGATTTAGAGTGGTGATTGTAGATTTGGGATATAGGTGAGA

>NPnpThy_23

AGGGTTTTTTTTAAATTTATATATTTTTTTTAAGGTAGTATTTATTTTTTATTGATAAGG

GTGGGGGAATAAAAGTTGATTGAAAGAAATGTATTTTGGTAGTTTGATTTTGGTAATTAT

AGAAAGTTTGTAAGGATTGTTTTTTTGGTAGTTTGTAGGTTTTATGGAAATGAAATTGTG

TTTGATTTTGTTTATAGGAGTGTTGGTTAATGAGGTGTTGTTTAGGTTTTTTATGTTTTG

GGTGTTATGGTGGTTTGTAGGTTGTGTTGTTTGTGTTTGTGTTTTTGATTGTTGGGTTGT

GGTTTTTTTGGTTGGTTTGGGTGGAATTGGGTATTTAGTTATTTGTAGATGTGAGTGTTG

TGGAGTAGTATTGGATTTAGAGTGGTGATTGTAGATTTGGGATATAGGTGAGA

>NPnpThy_24

AGGGTTTTTTTTAAATTTATATATTTTTTTTAAGGTAGTATTTATTTTTTACTGATAAGG

GTGGGGGAATAAAAGTTGATTGAAAGAAATGTATTTTGGTGGTTTGATTTTGGTAATTAT

AGAAAGTTTGTAAGGATTGTTTTTTTGGTAGTTTGTAGGTTTTATGGAAATGAAATTGTG

TTTGATTTTGTTTATAGGAGTGTTGGTTAATGAGGTGTTGTTTAGGTTTTTTATGTTTTG

GGTGTTATGGTGGTTTGTAGGTTGTGTTGTTTGTGTTTGTGTTTTTGATTGTTGGGTTGT

GGTTTTTTTGGTTGGTTTGGGTGGAATTGGGTATTTAGTTATTTGTAGATGTGAGTGTTG

TGGAGTAGTATTGGATTTAGAGTGGTGATTGTAGATTTGGGATATAGGTGAGA

>NPnpThy_27

AGGGTTTTTTTTAAATTTATATATTTTTTTTAAGGTAGTATTTATTTTTTATTGATAAGG

GTGGGGGAATAAAAGTTGATTGAAAGAAATGTATTTTGGTGGTTTGATTTTGGTAATTAT

AGAAAGTTTGTAAGGATTGTTTTTTTGGTAGTTTGTAGGTTTTATGGAAATGAAATTGTG

TTTGATTTTGTTTATAGGAGTGTTGGTTAATGAGGTGTTGTTTAGGTTTTTTATGTTTTG

GGTGTTATGGTGGTTTGTAGGTTGTGTTGTTTGTGTTTGTGTTTTTGATTGTTGGGTTGT

GGTTTTTTAGGTTGGTTTGGGTGGAATTGGGTATTTAGTTATTTGTAGATGTGAGTGTTG

TGGAGTAGTATTGGATTTAGAGTGGTGATTGTAGATTTGGGATATAGGTGAGA
