# Supplementary material for: Epigenetic interplay between mouse endogenous retroviruses and host genes
Source: Genome Biol. 2012 Oct 3;13(10):R89. doi: 10.1186/gb-2012-13-10-r89 (PMC3491417; doi:10.1186/gb-2012-13-10-r89)
Supplement: Additional file 4 — All bisulfite sequencing data. Compilation of all bisulfite sequences. [file gb-2012-13-10-r89-S4.zip › IAP8545_TE_embryo.doc]

Pnpt1 3’ LTR Miniprep Sequences

B6AJ Embryo

>Pnp3LTREmb_44

GAGAGTTGTAAGGTTAAGTATTGTAATGGAGAGGTTTTGCGGTATATATGAGTTTATTTT

AGGGAGATATGTTATTTTTTATGAAGGTTTAGTGTTTTAGTTTTTTTTTTAGGTAAAATG

ATATGGGAGTAGGTTAGGGTTGTTTTGGGTAAAAGTTTGTAAGTTTAAGAGTTAATTTTG

TATATGGTTTTTTTATTTATATATTGGGGATTTGATTTTTATTTTTATTTTTATTAATAT

GGGTGGTTTATTTGTTTTTATTAAAAGAAAAAGGGGGAGATGTTGGGAGTCGTTTTTATA

TTTGTCGTTATAAGATGGCGTTGATATTTTGTGTTTTAAGTGGTAAATAAATAATTTGCG

TATGTGTTAAGGGTATTTTATGATTATTTGTGTTTTGTTTTTCTTGTGATGTTAATTCGG

TCGATGGGTTGTAGTTAATTAAGGAGTGATATGTTTGAGGTGAAGGAGAATGTTTTTTAA

GAGGGATGGGGTTTTTGTTTTTTTTTTTTTTTGTTTTTTGTTTTTTTTTTTTTGAAGATG

TAAGAATAAAGTTTTGTCGTAGAAGATTTTGGTTTGTGGTGTTTTTTTTGGTTGGTTGTG

AGAACGTGTCGAATAATATTAATGTTTTTATTTTTTTTTGATTTATGATTTAAAATTATT

ATTATTTTAATTATTTTAGAGTGATATTAGGAAATATAGATTTTTTTTTAT

>Pnp3LTREmb_45

GAGAGTTGTAAGGTTAAGTATTGTAATGGAAAGGTTTTGCGGTATATATGAGTTTATTTT

AGGGAGATATGTTATTTTTTATGAAGGTTTAGTGTTTTAGTTTTTTTTTTTTAGGTAAAA

CGATACGGGAGTAGGTTAGGGTTGTTTTGGGTAAAAGTTTGTAAGTTTAAGAGTTAATTT

TGTATATGGTTTTTTATTTATATATTGGGGATTTGATTTTTATTTTTATTTTTATTAATA

TGGGTGGTTTATTTGTTTTTATTAAAAGAAAAAGGGGGAGATGTTGGGAGTCGTTTTTAT

ATTCGTCGTTATAAGATGGCGTTGATATTTTGTGTTTTAAGTGGTAAATAAATAATTTGC

GTATGTGTTAAGGGTATTTTATGATTATTTGTGTTTTGTTTTTTTCGTGACGTTAATTCG

GTCGATGGGTTGTAGTTAATTAAGGAGTGATACGTTCGAGGCGAAGGAGAATGTTTTTTA

AGAGGGACGGGGTTTTCGTTTTTTTTTTTTTTGTTTTTTGTTTTTTTTTTTTGAAGATGT

AAGAATAAAGTTTTGTCGTAGAAGATTTTGGTTTGTGGTGTTTTTTTTGGTCGGTCGTGA

GAACGCGTCGAATAATATTAATGTTTTTATTTTTTTTTGATTTATGATTTAAAATTATTA

TTATTTTAATTATTTTAGAGTGATATTAGGAAATATAGATTTTTTTTTAT

>Pnp3LTREmb_46

GAGAGTTGTAAGGTTAAGTATTGTAATGGAAAGGTTTTGCGGTATATATGAGTTTATTTT

AGGGAGATATGTTATTTTTTATGAAGGTTTAGTGTTTTAGTTTTTTTTTTTTAGGTAAAA

CGATACGGGAGTAGGTTAGGGTTGTTTTGGGTAAAAGTTTGTAAGTTTGAGAGTTAATTT

TGTATATGGTTTTTTTATTTATATATTGGGGATTTGATTTTTATTTTTATTTTTATTAAT

ATGGGTGGTTTATTTGTTTTTATTAAAAGAAAAAGGGGGAGATGTTGGGAGTCGTTTTTA

TTTTCGTCGTTATAAGATGGTGTTGATATTTTGTGTTTTAAGTGGTAAATAAATAATTTG

CGTATGTGTTAAGGGTATTTTATGATTATTTGTGTTTTGTTTTTTTCGTGACGTTAATTC

GGTCGATGGGTTGTAGTTAATTAAGGAGTGATACGTTCGAGGCGAAGGAGAATGTTTTTT

AAGAGGGACGGGGTTTTCGTTTTTTTTTTTTTTGTTTTTTGTTTTTTTTTTTTTGAAGAT

GTAAGAATAAAGTTTTGTCGTAGAAGATTTTGGTCTGTGGTGTTTTTTTTGGTCGGTCGT

GAGAACGCGTCGAATAATATTAATGTTTTTATTTTTTTTTGATTTATGATTTAAAATTAT

TATTATTTTAATTATTTTAGAGTGATATTAGGAAATATAGATTTTTTTTTAT

>Pnp3LTREmb_47

GAGAGTTGTAAGGTTAAGTATTGTAATGGAAAGGTTTTGCGGTATATATGAGTTTATTTT

AGGGAGATATGTTATTTTTTATGAAGGTTTAGTGTTTTAGTTTTTTTTTTTAGGTAAAAC

GATACGGGAGTAGGTTAGGGTTGTTTTGGGTAAAAGTTTGTAAGTTTAAGAGTTAATTTT

GTATATGGTTTTTTTATTTATATATTGGGGATTTGATTTTTATTTTTATTTTTATTAATA

TGGGTGGTTTATTTGTTTTTATTAAAAGAAAAAGGGGGAGATGTTGGGAGTCGTTTTTAT

ATTCGTCGTTATAAGATGGCGTTGATATTTTGTGTTTTAAGTGGTAAATAAATAATTTGC

GTATGTGTTAAGGGTATTTTATGATTATTTGTGTTTTGTTTTTTTCGTGACGTTAATTCG

GTCGATGGGTTGTAGTTAATCAAGGAGTGATACGTTCGAGGCGAAGGAGAATGTTTTTTA

AGAGGGACGGGGTTTTCGTTTTTTTTTTTTTTTGTTTTTTGTTTTTTTTTTTGAAGATGT

AAGAATAAAGTTTTGTCGTAGAAGATTTTGGTTTGTGGTGTTTTTTTTGGTCGGTCGTGA

GAACGCGTCGAATAACATTAATGTTTTTATTTTTTTTTGATTTATGATTTAAAATTATTA

TTATTTTAATTATTTTAGAGTGATATTAGGAAATATAGATTTTTTTTTAT

>Pnp3LTREmb_48

GAGAGGTTGTAAGGTTAAGTATTGTAATGGAAAGGTTTTGCGGTATATATGAGTTTATTT

TAGGGAGATATGTTATTTTTTATGAAGGTTTAGTGTTTTAGTTTTTTTTTTTTAGGTAAA

ACGATATGGGAGTAGGTTAGGGTTGTTTTGGGTAAAAGTTTGTAAGTTTAAGAGTTAATT

TTGTATATGGTTTTTTTATTTATATATTGGGGATTTGATTTTTATTTTTATTTTTATTAA

TATGGGTGGTTTATTTGTTTTTATTAAAAGAAAAAGGGGGAGATGTTGGGAGTCGTTTTT

ATTTTTGTCGTTATAAGATGGCGTTGATATTTTGTGTTTTAAGTGGTAAATAAATAATTT

GTGTATGTGTTAAGGGTATTTTATGATTATTTGTGTTTTGTTTTTTTCGTGACGTCAATT

CGGTCGATGGGTTGTAGTTAATTAAGGAGTGATACGTCCGAGGCGAAGGAGAATGTTTTT

TAAGAGGGACGGGGTTTTCGTTTTTTTTTTTTTTTTGTTTTTTGTTTTTTTTTTTTGAAG

ATGTAAGAATAAAGTTTTGTCGTAGAAGATTTTGGTTTGTGGTGTTTTTTTTGGTCGGTC

GTGAGAACGTGTCGAATAATATTAATGTTTTTATTTTTTTTTGATTTATGATTTAAAATT

ATTATTATTTTAATTATTTTAGAGTGATATTAGGAAATATAGATTTTTTTTTAT

>Pnp3LTREmb_49

TAAGGTTAAGTATTGTAATGGAAAGGTTTTGCGGTATATATGAGTTTATTTTAGGGAGAT

ATGTTATTTTTTATGAAGGTTTAGTGTTTTAGTTTTTTTTTTTTAGGTAAAACGATACGG

GAGTAGGTTAGGGTTGTTTTGGGTAAAAGTTTGTAAGTTTAAGAGTTAATTTTGTATATG

GTTTTTTTATTTATATATTGGGGATTTGATTTTTATTTTTATTTTTATTAATATGGGTGG

TTTATTTGTTTTTATTAAAAGAAAAAGGGGGAGATGTTGGGAGTCGTTTTTATATTCGTC

GTTATAAGATGGTGTTGATATTTTGTGTTTTAAGTGGTAAATAAATAATTTGCGTATGTG

TTAAGGGTATTTTATGATTATTTGTGTTTTGTTTTTTTCGTGACGTTAATTCGGTCGATG

GGTTGTAGTTAATTAAGGAGTGATACGTTCGAGGCGAAGGAGAATGTTTTTTAAGAGGGA

CGGGGTTCTCGTTTTTTTTTTTTTTTTGTTTTTTGTTTTTTTTTTTTTGAAGATGTAAGA

ATAAAGTTTTGTCGTAGAAGATTTTGGTCTGTGGTGTTTTTTTTGGTCGGTCGTGAGAAC

GCGTCGAATAATATTAATGTTTTTATTTTTTTTTGATTTATGATTTAAAATTATTATTAT

TTTAATTATTTTAGAGTGATATTAGGAAATATAGATTTTTTTTTAT

>Pnp3LTREmb_50

GAGAGTTGTAAGGTTAAGTATTGTAATGGAAAGGTTTTGCGGTATATATGAGTTTATTTT

AGGGAGATATGTTATTTTTTATGAAGGTTTAGTGTTTTAGTTTTTTTTTTTAGGTAAAAC

GATACGGGAGTAGGTTAGGGTTGTTTTGGGTACAAGTTTGTAAGTTTAAGAGTTAATTTT

GTATATGGTTTTTTTATTTATATATTGGGGATTTGATTTTTATTTTTATTTTTATTAATA

TGGGTGGCTTATTTGTTTTTATTAAAAGAAAAAGGGGGAGATGTTGGGAGTCGTTTTCAT

TTTTGTCGTTATAAGATGGCGTTGATATTTTGTGTTTTAAGTGGTAAATAAATAATTTGC

GTATGTGTTAAGGGTATTTTATGATTATTTGTGTTTTGTTTTTTTCGTGACGTTAATTCG

GTCGATGGGTTGTAGTTAATTAAGGAATGATACGTTCGAGGCGAAGGAGAATGTTTTTTA

AGAGGGACGGGGTTTTCGTTTTTTTTTTTTTTTGTTTTTTGTTTTTTTTTTTGAAGATGT

AAGAATAAAGTTTTGTCGTAGAAGATTTTGGTTTGTGGTGTTTTTTTTGGTCGGTCGTGA

GAACGCGTCGAATAATATTAATGTTTTTATTTTCTTTTGATTTATGATTTAAAATTATTA

TTATTTTAATTATTTTAGAGTGATATTAGGAAATATAGATTTTTTTTTAT

>Pnp3LTREmb_51

GAGAGTTGTAAGGTTAAGTATTGTAATGGAAAGGTTTGCGGTATATATGAGTTTATTTTA

GGGAGATATGTTATTTTTTATGAAGGTTTAGTGTTTTAGTTTTTTTTTTTTAGGTAAAAC

GATACGGGAGTAGGTTAGGGTTGTTTTGGGTAAAAGTTTGTAAGTTTAAGAGTTAATTTT

GTATATGGTTTTTTTATTTATATATTGGGGATTTGATTTTTATTTTTATTTTTATTAATA

TGGGTGGTTTATTTGTTTTTATTAAAAGAAAAAGGGGGAGATGTTGGGAGTCGTTTTTAT

ATTCGTCGTTATAAGATGGCGTTGATATTTTGTGTTTTAAGTGGTAAATAAATAATTTGC

GTATGTGTTAAGGGTATTTTATGATTATTTGTGTTTTGTTTTTTTCGTGACGTTAATTCG

GTCGATGGGCTGTAGTTAATTGAGGAGTGATACGTTCGAGGCGAAGGAGAATGTTTTTTA

AGAGGGACGGGGTTTTCGTTTTTTCTTTTTTTTGTTTTTTGTTTTTTTTTTGAAGATGTA

AGAATAAAGTTTTGTCGTAGAAGATTTTGGTTTGTGGTGTTTTTCTTGGTCGGTCGTGAG

AACGCGTCGAATAATATTAATGTTTTTATTTTTTTTTGATTTATGATTTAAAATTATTAT

TATTTTAATTATTTTAGAGTGATATTAGGAAATATAGATTTTTTTTTAT

>Pnp3LTREmb_52

GAGAGTTGTAAGGTTAAGTATTGTAATGGAAAGGTTTTGCGGTATATATGAGTTTATTTT

AGGGAGATATGTTATTTTTTATGAAGGTGTAGTGTTTTAGTTTTTTTTTTTTAGGTAAAA

CGATACGGGAGTAGGTTAGGGTTGTTTTGGGTAAAAGTTTGTAAGTTTAAGAGTTAATTT

TGTATATGGTTTTTTTATTTATATATTGGGGATTTGATTTTTATTTTTATTTTTATTAAT

ATGGGTGGTTTATTTGTTTTTATTAAAAGAAAAAGGGGGAGATGTTGGGAGTCGTTTTTA

TATTTGTCGTTATAAGATGGTGTTGATATTTTGTGTTTTAAGTGGTAAATAAATAATTTG

TGTATGTGTTAAGGGTATTTTATGATTATTTGTGTTTTGTTTTTTTTGTGACGTTAATTC

GGTCGATGGGTTGTAGTTAATTAAGGAGTGATACGTTTGAGGCGAAGGAGAATGTTTTTT

AAGAGGGATGGGGTTTTTGTTTTTTTTTTTTTTTGTTTTTTGTTTTTTTTTTTGAAGATG

TAAGAATAAAGTTTTGTCGTAGAAGATTTTGGTTTGTGGTGTTTTTTTTGGTCGGTCGTG

AGAACGCGTCGAATAATATTAATGTTTTTATTTTTTTTTGATTTATGATTTAAAATTATT

ATTATTTTAATTATTTTAGAGTGATATTAGGAAATATAGATTTTTTTTTAT

>Pnp3LTREmb_53

GAGAGTTGTAAGGGTTAAGTATTGTAATGGAAAGGTTTTGCGGTATATATGAGTTTATTT

TAGGGAGATATGTTATTTTTTATGAAGGTTTAGTGTTTTAGTTTTTTTTTTTTAAGTAAA

ACGATACGGGAGTAGGTTAGGGTTGTTTTGGGTAAAAGTTTGTAAGTTTAAGAGTTAATT

TTGTATATGGTTTTTTTATTTATATATTGGGGATTTGATTTTTATTTTTATTTTTATTAA

TATGGGTGGTTTATTTGTTTTTATTAAAAGAAAAAGGGGGAGATGTTGGGAGTCGTTTTT

ATTTTCGTCGTTATAAGATGGTGTTGATATTTTGTGTTTTAAGTGGTAAATAAATAATTT

GCGTATGTGTTAAGGGTATTTTATGATTATTTGTGTTTTGTTTTTTTCGTGACGTTAATT

CGGTCGATGGGTTGTAGTTAATTAAGGAGTGATACGTTCGAGGCGAAGGAGAATGTTTTT

TAAGAGGGACGGGGTTTTCGTTTTTTTTTTTTGTTTTTTGTTTTTTTTTTTGAAGATGTA

AGAATAAAGTTTTGTCGTAGAAGATTTTGGTCTGTGGTGTTTTTTTTGGTCGGTCGTGAG

AACGCGTCGAATAATATTAATGTTTTTATTTTTTTTTGATTTATGATTTAAAATTATTAT

TATTTTAATTATTTTAGAGTGATATTAGGAAATATAGATTTTTTTTTAT

>Pnp3LTREmb_54

GAGAGTTGTAAGGTTAAGTATTGTAATGGAAAGGTTTTGCGGTATATATGAGTTTATTTT

AGGGAGATATGTTATTTTTTATGAAGGTTTAGTGTTTTAGTTTTTTTTTTTAGGTAAAAC

GATACGGGAGTAGGTTAGGGTTGTTTTGGGTAAAAGTTTGTAAGTTTAAGAGTTAATTTT

GTATATGGTTTTTTTATTTATATATTGGGGATTTGATTTTTATTTTTATTTTTATTAATA

TGGGTGGTTTATTTGTTTTTATTAAAAGAAAAAGGGGGAGATGTTGGGAGTCGTTTTTAT

ATTCGTCGTTATAAGATGGCGTTGATATTTTGTGTTTTAAGTGGTAAATAAATAATTTGC

GTATGTGTTAAGGGTATTTTATGATTATTTGTGTTTTGTTTTTTTCGTGACGTTAATTCG

GTCGATGGGTTGTAGTTAATCAAGGAGTGATACGTTCGAGGCGAAGGAGAATGTTTTTTA

AGAGGGACGGGGTTTTCGTTTTTTTTTTTTTGTTTTTTGTTTTTTTTTTTTGAAGATGTA

AGAATAAAGTTTTGTCGTAGAAGATTTTGGTTTGTGGTGTTTTTTTTGGTCGGTCGTGAG

AACGCGTCGAATAACATTAATGTTTTTATTTTTTTTTGATTTATGATTTAAAATTATTAT

TATTTTAATTATTTTAGAGTGATATTAGGAAATATAGATTTTTTTTTAT

>Pnp3LTREmb_55

GAGAGTTGTAAGGTTAAGTATTGTAATGGAAAGGTTTTGCGGTATATATGAGTTTATTTT

AGGGAGATATGTTATTTTTTATGAAGGTTTAGTGTTTTAGTTTTTTTTTTTAGGTAAAAT

GATATGGGAGTAGGTTAGGGTTGTTTTGGGTAAAAGTTTGTAAGTTTAAGAGTTAATTTT

GTATATGGTTTTTTTATTTATATATTGGGGATTTGATTTTTATTTTTATTTTTATTAATA

TGGGTGGTTTATTTGTTTTTATTAAAAGAAAAAGGGGGAGATGTTGGGAGTCGTTTTTAT

ATTTGTCGTTATAAGATGGCGTTGATATTTTGTGTTTTAAGTGGTAAATAAATAATTTGC

GTATGTGTTAAGGGTATTTTATGATTATTTGTGTTTTGTTTTTCTTGTGATGTTAATTCG

GTCGATGGGTTGTAGTTAATTAAGGAGTGATATGTTTGAGGTGAAGGAGAATGTTTTTTA

AGAGGGATGGGGTTTTTGTTTTTTTTTTTTTTGTTTTTTGTTTTTTTTTTTGAAGATGTA

AGAATAAAGTTTTGTCGTAGAAGATTTTGGTTTGTGGTGTTTTTTTTGGTTGGTTGTGAG

AACGTGTCGAATAATATTAATGTTTTTATTTTTTTTTGATTTATGATTTAAAATTATTAT

TATTTTAATTATTTTAGAGTGATATTAGGAAATATAGATTTTTTTTTAT

>Pnp3LTREmb_56

GTTGTAAGGTTAAGTATTGTAATGGAAAGGTTTTGCGGTATATATGAGTTTATTTTAGGG

AGATATGTTATTTTTTATGAAGGTTTAGTGTTTTAGTTTTTTTTTTTTAGGTAAAACGAT

ACGGGAGTAGGTTAGGGTTGTTTTGGGTAAAAGTTTGTAAGTTTAAGAGTTAATTTTGTA

TATGGTTTTTTTATTTATATATTGGGGATTTGATTTTTATTTTTATTTTTATTAATATGG

GTGGTTTATCTGTTTTTATTAAAAGAAAAAGGGGGAGATGTTGGGAGTCGTTTTTATATT

TGTCGTTATAAGATGGTGTTGATATTTTGTGTTTTAAGTGGTAAATAAATAATTTGTGTA

TGTGTTAAGGGTATTTTATGATTATTTGTGTTTTGTTTTTTTTGTGACGTTAATTCGGTC

GATGGGTTGTAGTTAATTAAGGAGTGATACGTTTGAGGCGAAGGAGAATGTTTTTTAAGA

GGGATGGGGTTTTTGTTTTTTTTTTTTTTGTTTTTTGTTTTTTTTTTTTGAAGATGTAAG

AATAAAGTTTTGTCGTAGAAGATTTTGGTTTGTGGTGTTTTTTTTGGTCGGTCGTGAGAA

CGCGTCGAATAATATTAATGTTTTTATTTTTTTTTGATTTATGATTTAAAATTATTATTA

TTTTAATTATTTTAGAGTGATATTAGGAAATATAGATTTTTTTTTAT

>Pnp3LTREmb_57

GAGAGTTGTAAGGTTAAGTATTGTAATGGAAAGGTTTTGCGGTATATATGAGTTTATTTT

AGGGAGATATGTTATTTTTTATGAAGGTTTAGTGTTTTAGTTTTTTTTTTTAGGTAAAAC

GATACGGGAGTAGGTTAGGGTTGTTTTGGGTAAAAGTTTGTAAGTTTAAGAGTTAATTTT

GTATATGGTTTTTTTATTTATATATTGGGGATTTGATTTTTATTTTTATTTTTATTAATA

TGGGTGGTTTATTTGTTTTTATTAAAAGAAAAAGGGGGAGATGTTGGGAGTCGTTTTTAT

ATTTGTCGTTATAAGATGGTGTTGATATTTTGTGTTTTAAGTGGTAAATAAATAATTTGT

GTATGTGTTAAGGGTATTTTATGATTATTTGTGTTTTGTTTTTTTTGTGACGTTAATTCG

GTCGATGGGTTGTAGTTAATTAAGGAGTGATACGTTTGAGGCGAAGGAGAATGTTTTTTA

AGAGGGATGGGGTTTTTGTTTTTTTTTTTTTTGTTTTTTGTTTTTTTTTTTTGAAGATGT

AAGAATAAAGTTTTGTCGTAGAAGATTTTGGTTTGTGGTGTTTTTTTTGGTCGGTCGTGA

GAACGCGTCGAATAATATTAATGTTTTTATTTTTTTTTGATTTATGATTTAAAATTATTA

TTATTTTAATTATTTTAGAGTGATATTAGGAAATATAGATTTTTTTTTAT

>Pnp3LTREmb_58

GAGAGGTTGTAAGGTTAAGTATTGTAATGGAAAGGTTTTGCGGTATATATGAGTTTATTT

TAGGGAGATATGTTATTTTTTATGAAGGTTTAGTGTTTTAGTTTTTTTTTTAGGTAAAAC

GATACGGGAGTAGGTTAGGGTTGTTTTGGGTAAAAGTTTGTAAGTTTAAGAGTTAATTTT

GTATATGGTTTTTTTATTTATATATTGGGGATTTGATTTTTATTTTTATTTTTATTAATA

TGGGTGGTTTATTTGTTTTTATTAAAAGAAAAAGGGGGAGATGTTGGGAGTCGTTTTTAT

ATTCGTCGTTATAAGATGGTGTTGATATTTTGTGTTTTAAGTGGTAAATAAATAATTTGC

GTATGTGTTAAGGGTATTTTGTGATTATTTGTGTTTTGTTTTTTTCGTGACGTTAATTCG

GTCGATGGGTTGTAGTTAATTAAGGAGTGATACGTTCGAGGCGAAGGAGAATGTTTTTTA

AGAGGGACGGGGTTTTCGTTTTTTTTTTTTTTTTGTTTTTTGTTTTTTTTTTTGAAGATG

TAAGAATAAAGTTTTGTCGTAGAAGATTTTGGTCTGTGGTGTTTTTTTTGGTCGGTCGTG

AGAACGCGTCGAATAATATTAATGTTTTTATTTTTTTTTGATTTATGATTTAAAATTATT

ATTATTTTAATTATTTTAGAGTGATATTAGGAAATATAGATTTTTTTTTAT

>Pnp3LTREmb_59

GAGAGTTGTAAGGTTAAGTATTGTAATGGAAAGGTTTTGCGGTATATATGAGTTTATTTT

AGGGAGATATGTTATTTTTTATGAAGGTTTAGTGTTTTAGTTTTTTTTTTAGGTAAAACG

ATACGGGAGTAGGTTAGGGTTGTTTTGGGTAAAAGTTTGTAAGTTTAAGAGTTAATTTTG

TATATGGTTTTTTTATTTATATATTGGGGATTTGATTTTTATTTTTATTTTTATTAATAT

GGGTGGTTTATTTGTTTTTATTAAAAGAAAAAGGGGGAGATGTTGGGAGTCGTTTTTATA

TTCGTCGTTACAAGATGGCGTTGATATTTTGTGTTTTAAGTGGTAAATAAATAATCTGCG

TATGTGTTAAGGGTATTTTATGATTATTTGTGTTTTGTTTTTTTCGTGACGTTAATTCGG

TCGATGGGCTGTAGTTAATTAAGGAGTGATACGTTCGAGGCGAAGGAGAATGTTTTTTAA

GAGGGACGGGGTTTTCGTTTTTTTTTTTTTTGTTTTTTGTTTTTTTTTTTTTGAAGATGT

AAGAATAAAGTTTTGTCGTAGAAGATTTTGGTTTGTGGTGTTTTTTTTGGTCGGTCGTGA

GAACGCGTCGAATAACATTAATGTTTTTATTTTCTTTTGATTTATGATTTAAAATTATTA

TTATTTTAATTATTTTAGAGTGATATTAGGAAATATAGATTTTTTTTTAT

>Pnp3LTREmb_60

GAGAGTTGTAAGGTTAAGTATTGTAATGGAAAGGTTTTGCGGTATATATGAGTTTATTTT

AGGGAGATATGTTATTTTTTATGAAGGTTTGGTGTTTTAGTTTTTTTTTTTAGGTAAAAC

GATATGGGAGTAGGTTAGGGTTGTTTTGGGTAAAAGTTTGTAAGTTTAAGAGTTAATTTT

GTATATGGTTTTTTTATTTATATATTGGGGATTTGATTTTTATTTTTATTTTTATTAATA

TGGGTGGTTTATTTGTTTTTATTAAAAGAAAAAGGGGGAGATGTTGGGAGTCGTTTTTAT

TTTCGTCGTTATAAGATGGTGTTGATATTTTGCGTTTTAAGTGGTAAATAAATAATTTGC

GTATGTGTTAAGGGTATTTTATGATTATTTGTGTTTTGTTTTTTTCGTGACGTTAATTCG

GTCGATGGGTTGTAGTCAATTAAGGAGTGATACGTTCGAGGCGAAGGAGAATGTTTTTTA

AGAGGGATGGGGTTTTTGTTTTTTTTTTTTTTTGTTTTTTGTTTTTTTTTTTTGAAGATG

TAAGAATAAAGTTTTGTCGTAGAAGATTTTGGTTTGTGGTGTTTTTTTGGTCGGTCGTGA

GAACGCGTCGAATAATATTAATGTTTTTATTTTTTTTTGATTTATGATTTAAAATTATTA

TTATTTTAATTATTTTAGAGTGATATTAGGAAATATAGATTTTTTTTTAT

>Pnp3LTREmb_61

GAGAGTTGTAAGGTTAAGTATTGTAATGGAAAGGTTTTGCGGTATATATGAGTTTATTTT

AGGGAGATATGTTATTTTTTATGAAGGTTTAGTGTTTTAGTTTTTTTTTTTAGGTAAAAC

GATACGGGAGTAGGTTAGGGTTGTTTTGGGTAAAAGTTTGTAAGTTTTAGAGTTAATTTT

GTATATGGTTTTTTTATTTATATATTGGGGATTTGATTTTTATTTTTATTTTTATTTATA

TGGGTGGTTTATTTGTTTTTATTAAAAGAAAAAGGGGGAGATGTTGGGAGTCGTTTTTAT

TTTTGTCGTTACCAGAAGGCGTTGATATTTTGTGTTTTAAGTGGTAAATAAATTATTTGC

GTATGTGTTAAGGGTATTTTATGATTATTTGTGTTTTGTTTTTTTCGTGACGTTAATTCG

GTCGATGGGCTGTAGTTAATTAAGGAATGATACCTTCGAGGCGAAGGAGAATGTTTTTTA

AGAGGGACGGGGTTTTCGTTTTTTTTTTTTTTGTTTTTTGTTTTTTTTTTTGAAGATGTA

AGAATAAAGTTTTGTCGTAGAAGATTTTGGTTTGTGGTGTTTTTTTTGGTCGGTCGTGAG

AACGCGTCGAATAACATTAATGTTTTTATTTTCTTTTGATTTATGATTTAAAATTATTAT

TATTTTAATTATTTTAGAGTGATATTAGGAAATATAGATTTTTTTTTAT

>Pnp3LTREmb_63

GAGAGTTGTAAGGTTAAGTATTGTAATGGAAAGGTTTGCGGTATATATGAGTTTATTTTA

GGGAGATATGTTATTTTTTATGAAGGTTTAGTGTTTTAGTTTTTTTTTTTTAGGTAAAAC

GATACGGGAGTAGGTTAGGGTTGTTTTGGGTAAAAGTTTGTAAGTTTAAGAGTTAATTTT

GTATATGGTTTTTTTATTTATATATTGGGGATTTGATTTTTATTTTTATTTTTATTAATA

TGGGTGGTTTATTTGTTTTTATTAAAAGAAAAAGGGGGAGATGTTGGGAGTCGTTTTTAT

TTTCGTCGTTATAAGATGGTGTTGATATTTTGTGTTTTAAGTGGTAAATAAATAATTTGC

GTATGTGTTAAGGGTATTTTATGATTATTTGTGTTTTGTTTTTTTCGTGACGTTAATTCG

GTCGATGGGTTGTAGTTAATTAAGGAGTGATACGTTCGAGGCGAAGGAGAATGTTTTTTA

AGAGGGACGGGGTTTTCGTTTTTTTTTTTTTTTGTTTTTTGTTTTTTTTTTTTGAAGATG

TAAGAATAAAGTTTTGTCGTAGAAGATTTTGGTCTGTGGTGTTTTTTTTGGTCGGTCGTG

AGAACGCGTCGAATAATATTAATGTTTTTATTTTTTTTTGATTTATGGTTTAAAATTATT

ATTATTTTAATTATTTTAGAGTGATATTAGGAAATATAGATTTTTTTTTAT

>Pnp3LTREmb_64

GAGAGTTGTAAGGTTAAGTATTGTAATGGAAAGGTTTTGCGGTATATATGAGTTTATTTT

AGGGAGATATGTTATTTTTTATGAAGGTTTAGTGTTTTAGTTTTTTTTTTTAGGTAAAAC

GATACGGGAGTAGGTTAGGGTTGTTTTGGGTAAAAGTTTGTAAGTTTAAGAGTTAATTTT

GTATATGGTTTTTTTATTTATATATTGGGGATTTGATTTTTATTTTTATTTTTATTAATA

TGGGTGGTTTATTTGTTTTTATTAAAAGAAAAAGGGGGAGATGTTGGGAGTCGTTTTTAT

ATTCGTCGTTATAAGATGGCGTTGATATTTTGTGTTTTAAGTGGTAAATAAATAATTTGT

GTATGTGTTAAGGGTATTTTATGATTATTTGTGTTTTGTTTTTTTCGTGACGTTAATTCG

GTCGATGGGTTGTAGTTAATTAAGGAGTGATACGTTCGAGGCGAAGGAGAATGTTTTTTA

AGAGGGACGGGGTCTTCGTTTTTTTTTTTTTTGTTTTTTGTTTTTTTTTTTTGAAGATGT

AAGAATAAAGTTTTGTCGTAGAAGATTTTGGTTTGTGGTGTTTTTTTTGGTCGGTCGTGA

GAACGCGTCGAATAATATTAATGTTTTTATTTTTTTTTGATTTATGATTTAAAATTATTA

TTATTTTAATTATTTTAGAGTGATATTAGGAAATATAGATTTTTTTTTAT

>Pnp3LTREmb_65

GAGAGTTGTAAGGTTAAGTATTGTAATGGAAAGGTTCTGCGGTATATATGAGTTTATTTT

AGGGAGATATGTTATTTTTTATGAAGGTTTAGTGTTTTAGTTTTTTTTAGGTAAAACGAT

ACGGGAGTAGGTTAGGGTTGTTTTGGGTAAAAGTTTGTAAGTTTAAGAGTTAATTTTGTA

TATGGTTTTTTTATTTATATATTGGGGATTTGATTTTTATTTTTATTTTTATTAATATGG

GTGGTTTATTTGTTTTTATTAAAAGAAAAAGGGGGAGATGTTGGGAGTTGTTTTTATATT

TGTCGTTATAAGATGGTGTTGATATTTTGTGTTTTAAGTGGTAAATAAATAATTTGTGTA

TGTGTTAAGGGTATTTTATGATTATTTGTGTTTTGTTTTTTTCGTGGTGCTAATTCGGTC

GATGGGTTGTAGTTAATTAAGGAGTGATATGTTCGAGGCGAAGGAGAATGTTTTTTAAGA

GGGATGGGGTTTTCGTTTTTTTTTTTTTTGTTTTTTGTTTTTTTTTGAAGATGTAAGAAT

AAAGTTTTGTCGTAGAAGATTTTGGTTTGTGGTGTTTTTCTTGGTCGGTTGTGAGAACGT

GTCGAATAATATCAATGTTTTTATTTTTTTTTGATTTATGATTTAAAATTATTATTATTT

TAATTATTTTAGAGTGATATTAGGAAATATAGATTTTTTTTTAT

>Pnp3LTREmb_66

GAGAGTTGTAAGGTTAAGTATTGTAATGGAAAGGTTTTGCGGTATATATGAGTTTATTTT

AGGGAGATATGTTATTTTTTATGAAGGTTTAGTGTTTTAGTTTTTTTTTTTAGGTAAAAC

GATACGGGAGTAGGTTAGGGTTGTTTTGGGTAAAAGTTTGTAAGTCTAAGAGTTAATTTG

GTATATGGTTTTTTTATTTATATATTGGGGATTTGATTTTTATTTTTATTTTTATTAATA

TGGGTGGTTTATTTGTTTTTATTAAAAGAAAAAGGGGGAGATGTTGGGAGTCGTTTTTAT

TTTGGTCGTTATAAGAGGGCGTTGATATTTTGTGTTTTAAGTGGTAAATAAATAATTTGC

GTATGTGTTAAGGGTATTTTATGATTATTTGTGTTTTGTTTTTTTCGTGACGTTAATTCG

GTCGATGGGTTGTAGTTAATTAAGGATTGATCCTTTCGAGGCGAAGGAGAATGTTTTTTA

AGAGGGACGGGGTTTTCGTTTTTTTTTTTTTTGTTTTTTGTTTTTTTTTTTGAAGATGTA

AGAATAAAGTTTTGTCGTAGAAGATTTTGGTTTGTGGTGTTTTTTTTGGTCGGTCGTGAG

AACGCGTCGAATAATATTAATGTTTTTATTTTTTTTTGATTTATGATTTAAAATTATTAT

TATTTTAATTATTTTAGAGTGATATTAGGAAATATAGATTTTTTTTTAT

%Me-CpG (18)

43.5%

43.5%

60.9%

69.6%

69.6%

69.6%

82.6%

82.6%

95.7%

95.7%

95.7%

95.7%

95.7%

100%

100%

100%

100%

100%
