# Supplementary material for: Epigenetic interplay between mouse endogenous retroviruses and host genes
Source: Genome Biol. 2012 Oct 3;13(10):R89. doi: 10.1186/gb-2012-13-10-r89 (PMC3491417; doi:10.1186/gb-2012-13-10-r89)
Supplement: Additional file 4 — All bisulfite sequencing data. Compilation of all bisulfite sequences. [file gb-2012-13-10-r89-S4.zip › IAP8545_TE_thymus.doc]

Pnpt1 B6AJ Thymus-3’ LTR

Miniprep Sequences

>Pnp3LTR_26

AGAGTTGTAAGGTTAAGTATTGTAATGGAAAGGTTGTGCGGTATATATGAGTTTATTTTA

GGGAGATATGTTATTTTTTATGAAGGTTTAGTGTTTTAGTTTTTTTTTTTAGGTAAAACG

ATACGGGAGTAGGTCAGGGTTGTTTTGGGTAAAAGTTTGTAAGTTTAAGAGTTAATTTTG

TATATGGTTTTTTTATTTATATATTGGGGATTTGATTTTTATTTTTATTTTTATTAATAT

GGGTGGTTTATTTGTTTTTATTAAAAGAAAAAGGGGGAGATGTTGGGAGTCGTTTTTATT

TTCGTCGTTATAAGATGGCGTTGATATTTTGTGTTTTAAGTGGTAAATAAATAATTTGCG

TATGTGTTAAGGGTATTTTATGATTATTTGTGTTTTGTTTTTTTCGTGACGTTAATTCGG

TCGATGGGTTGTAGTTAATTAAGGAGTGATACGTTCGAGGCGAAGGAGAATGTTTTTTAA

GAGGGACGGGGTTTTCGTTTTTTTTTTTTTTGTTTTTTGTTTTTTTTTTGAAGATGTAAG

AATAAAGTTTTGTCGTAGAAGATTTTGGTTTGTGGTGTTTTTTTTGGTCGGTCGTGAGAA

CGCGTCGAATAATATTAATGTTTTTATTTTTTTTTGATTTATGATTTAAAATTATTATTA

TTTTAATTATTTTAGAGTGATATTAGGAAATATAGATTTTTTTTTAT

>Pnp3LTR_28

AGAGTTGTAAGGTTAAGTATTGTAATGGAAAGGTTTTGCGGTATATATGAGTTTATTTTA

GGGAGATATGTTATTTTTTATGAAGGTTCAGTGTTTTAGTTTTTTTTTTCAGGTAAAACG

ATACGGGAGCAGGTTAGGGTTGTTTTGGGTAAAAGTTTGTAAGTTTAAGAGTTAATTTTG

TATATGGTTTTTTTATTTATATATTGGGGATTTGATTTTTATTTTTATTTTTATTAATAT

GGGTGGTTTATTTGTTTTTATTAAAAGAAAAAGGGGGAGATGTTGGGAGTCGTTTTTATT

TTTGTCGTTATAAGATGGCGTTGATATTTTGTGTTTTAAGTGGTAAATAAATAATTTGCG

TATGTGTTAAGGGTATTTTATGATTATTTGTGTTTTGTTTTTTTCGTGACGTTAATTCGG

TCGATGGGTTGTAGTTAATTAAGGAGTGATACGTTTGAGGCGAAGGAGAATGTTTTTTAA

GAGGGACGGGGTTTTCGTTTTTTTTTTTTTTTGTTTTTTGTTTTTTTTTTTTGAAGATGC

AAGAATAAAGTTTTGTCGTAGAAGATTTTGGTTTGTGGTGTTTTTTTTGGTCGGTCGTGA

GAACGCGTCGAATAATATTAATGTTTTTATTTTTTTTTGATTTATGATTTAAAATTATTA

TTATTTTAATTATTTTAGAGTGATATTAGGAAATATAGATTTTTTTTTAT

>Pnp3LTR_29

AGAGTTGTAAGGTTAAGTATTGTAATGGAAAGGTTTTGCGGTATATATGAGTTTATTTTA

GGGAGATATGTTATTTTTTATGAAGGTTTAGTGTTTTAGTTTTTTTTTTTTAGGTAAAAC

GATACGGGAGTAGGTTAGGGTTGTTTTGGGTAAAAGTTTGTAAGTTTAAGAGTTAATTTT

GTATATGGTTTTTTTATTTATATATTGGGGATTTGATTTTTATTTTTATTTTTATTAATA

TGGGTGGTTTATTTGTTTTTATTAAAAGAAAAAGGGGGAGATGTTGGGAGTCGTTTTTAT

TTTTGTCGTTATAAGAGGGCGTTGATATTTTGTGTTTTAAGTGGTAAATAAATAATTTGG

GTATGTGTTAAGGGTATTTTATGATTATTTGTGTTTTGTTTTTTTCGTGACGTTAATTCG

GTCGATGGGTTGTAGTTAATTAAGGATTGATATTTTCGAGGGGAAGGAGAATGTTTTTTA

AGAGGGACGGGGTTTTCGTTTTTTTTTTTTTTGTTTTTTGTTTTTTTTTTTTGAAGATGT

AAGAATAAAGTTTTGTCGTAGAAGATTTTGGTTTGTGGTGTTTTTTTTGGTCGGTCGTGA

GAACGCGTCGAATAATATTAATGTTTTTATTTTTTTTTGATTTATGATTTAAAATTATTA

TTATTTTAATTATTTTAGAGTGATATTAGGAAATATAGATTTTTTTTTAT

>Pnp3LTR_30

AGAGTTGTAAGGTTAAGTATTGTAATGGAAAGGTTTTGCGGTATATATGAGTTTATTTTA

GGGAGATATGTTATTTTTTATGAAGGTTTAGTGTTTTAGTTTTTTTTTTTAGGTAAAACG

ATACGGGAGTAGGTTAGGGTTGTTTTGGGTAAAAGTTTGTAAGCTTAAGAGTTAATTTTG

TATATGGTTTTTTTATTTATATATTGGGGATTTGATTTTTATTTTTATTTTTATTAATAT

GGGTGGTTTATTTGTTTTTATTAAAAAAAAAAGGGGGAGATGTTGGGAGTCGTTTTTATT

TTTGTCGTTATAAGATGGCGTTGATATTTTGTGTTTTAAGTGGTAAATAAATAATTTGCG

TATGTGTTAAGGGTATTTTATGATTATTTGTGTTTTGTTTTTTTCGTGACGTTAATTCGG

TCGATGGGTTGTAGTTAATTAAGGAGTGATACGTTCGAGGCGAAGGAGAATGTTTTTTAA

GAGGGACGGGGTTTTTGTTTTTTTTTTTTTTTGTTTTTTGTTTTTTTTTTTTGAAGATGT

AAGAATAAAGTTTTGTCGTAGAAGATTTTGGTTTGTGGTGTTTTTTTTGGTCGGTCGTGA

GAACGTGTCGAATAATATTAATGTTTTTATTTTTTTTTGATTTATGATTTAAAATTATTA

TTATTTTAATTATTTTAGAGTGATATTAGGAAATATAGATTTTTTTTTAT

>Pnp3LTR_31

AGAGTTGTAAGGTTAAGTATTGTAATGGAAAGGTTTTGCGGTATATATGAGTTTATTTTA

GGGAGATATGTTATTTTTTATGAAGGTTTAGTGTTTTAGTTTTTTTTTTTTAGGTAAAAC

GATACGGGAGTAGGTTAGGGTTGTTTTGGGTAAAAGTTTGTAAGTTTAAGAGTTAATTTT

GTATATGGTTTTTTATTTATATATTGGGGATTTGATTTTTATTTTTATTTTTATTAATAT

GGGTGGTTTATTTGTTTTTATTAAAAGAAAAAGGGGGAGATGTTGGGAGTCGTTTTTATT

TTTGTCGTTATAAGATGGCGTTGATATTTTGTGTTTTAAGTGGTAAATAGATAATTTGCG

TATGTGTTAAGGGTATTTTATGATTATTCGTGTTTTGTTTTTTTCGTGACGTTAATTCGG

TCGATGGGTTGTAGTTAATTAAGGAATGATACGTTCGAGGCGAAGGAGAATGTTTTTTAA

GAGGGACGGGGTTTTCGTTTTTTTTTTTTTTTGTTTTTTGTTTTTTTTTTTTTGAAGATG

TAAGAATAAAGTTTTGTCGTAGAAGATTTTGGTTTGTGGTGTTTTTTTTGGTCGGTCGTG

AGAACGCGTCGAATAATATTAATGTTTTTATTTTTTTTTGATTTATGATTTAAAATTATT

ATTATTTTAATTATTTTAGAGTGATATTAGGAAATATAGATTTTTTTTTAT

>Pnp3LTR_33

AGAGTTGTAAGGTTAAGTATTGTAATGGAAAGGTTTGCGGTATATATGAGTTTATTTTAG

GGAGATATGTTATTTTTTATGAAGGTTTAGTGTTTTAGTTTTTTTTTTTTTAGGTAAAAC

GATACGGGAGTAGGTTAGGGTTGTTTTGGGTAAAAGTTTGTAAGTTTAAGAGTTAATTTT

GTATATGGTTTTTTTATTTATATATTGGGGATTTGATTTTTATTTTTATTTTTATTAATA

TGGGTGGTTTATTTGTTTTTATTAAAAGAAAAAGGGGGAGATGTTGGGAGTCGTTTTTAT

TTTCGTCGTTATAAGATGGCGTTGATATTTTGTGTTTTAAGTGGTAAATAAATAATTTGC

GTATGTGTTAAGGGTATTTTATGATTATTTGTGTTTCGTTTTTTTCGTGACGTTAATTCG

GTCGATGGGTTGTAGTTAATTAAGGAGTGATACGTTCGAGGCGAAGGAGAATGTTTTTTA

AGAGGGACGGGGTTTTCGTTTTTTTTTTTTTGTTTTTTGTTTTTTTTTTGAAGATGTAAG

AATAAAGTTTTGTCGTAGAAGATTTTGGTTTGTGGTGTTTTTTTTGGTCGGTTGTGAGAA

CGCGTCGAATAATATTAATGTTTTTATTTTTTTTTGATTTATGATTTAAAATTATTATTA

TTTTAATTATTTTAGAGTGATATTAGGAAATATAGATTTTTTTTTAT

>Pnp3LTR_34

TAAGGTTAAGTATTGTAATGGAAAGGTTTTGCGGTATATATGAGTTTATTTTAGGGAGAT

ATGTTATTTTTTATGAAGGTTTAGTGTTTTAGTTTTTTTTTTTTAGGTAAAACGATACGG

GAGCAGGTTAGGGTTGTTTTGGGTAAAAGTTTGTAAGTCTAAGAGTTAATTTTGTATATG

GTTTTTTTATTTATATATTGGGGATTTGATTTTTATTTTTATTTTTATTAATATGGGTGG

TTTATTTGTTTTTATTAAAAGAAAAAGGGGGAGATGTTGGGAGTCGTTTTTATATTTGTC

GTTATAAGAGGGCGTTGATATTTTGTGTTTTAAGTGGTAAATAAATAATTTGCGTACGTG

TTAAGGGTATTTTACGATTATTTGTGTTTTGTTTTTTTCGTGACGTTAATTCGGTCGATG

GGTTGTAGTTAATTAAGGATTGATACGTTCGAGGCGAAGGAGAATGTTTTTTAAGAGGGA

CGGGGTTTTCGTTTTTTTTTTTTTGTTTTTTGTTTTTTTTTTTTGAAGATGTAAGAATAA

AGTTTTGTCGTAGAAGATTTTGGTTTGTGGTGTTTTTTTTGGTCGGTCGTGAGAACGCGT

CGAATAATATTAATGTTTTTATTTTTTTTTGATTTATGATTTAAAATTATTATTATTTTA

ATTATTTTAGAGTGATATTAGGAAATATAGATTTTTTTTTAT

>Pnp3LTR_35

AGAGTTGTAAGGTTAAGTATTGTAATGGAAAGGTTTTGCGGTATATATGAGTTTATTTTA

GGGAGATATGTTATTTTTTATGAAGGTTTAGTGTTTTAGTTTTTTTTTTGGGTAAAACGA

TACGGGAGTAGGTTAGGGTTGTTTTGGGTAAAAGTTTGTAAGTTTAAGAGTTAATTTTGT

ATATGGTTTTTTTATTTATATATTGGGGATTTGATTTTTATTTTTATTTTTATTAATATG

GGTGGTTTATTTGTTTTTATTAAAAGAAAAAGGGGGAGATGTTGGGAGTCGTTTTTATTT

TCGTCGTTATAAGATGGCGTTGATATTTTGTGTTTTAAGTGGTAAATAAATAATTTGCGT

ATGTGTTAAGGGTATTTTATGATTATTTGTGTTTTGTTTTTTTCGTGACGTTAATTCGGT

CGATGGGTTGCAGTTAATTAAGGAGTGATACGTTCGAGGCGAAGGAGAATGTTTTTTAAG

AGGGACGGGGTTTTCGTTTTTTTTTTTTGTTTTTTGTTTTTTTTTTTTGAAGATGTAAGA

ATAAAGTTTTGTCGTAGAAGATTTTGGTTTGTGGTGTTTTTTTTGGTCGGTCGTGAGAAC

GCGTCGAATAATATTAATGTTTTTATTTTTTTTTTGATTTATGATTTAAAATTATTATTA

TTTTAATTATTTTAGAGTGATATTAGGAAATATAGATTTTTTTTTAT

>Pnp3LTR_36

AGAGTTGTAAGGTTAAGTATTGTAATGGAAAGGTTTGCGGTATATATGAGTTTATTTTAG

GGAGATATGTTATTTTTTATGAAGGTTTAGTGTTTTAGTTTTTTTTTTTAGGTAAAACGA

TACGGGAGTAGGTTAGGGTTGTTTTGGGTAAAAGTTTGTAAGTTTAAGAGTTAATTTTGT

ATATGATTTTTTTATTTATATATTGGGGATTTGATTTTTATTTTTATTTTTATTAATATG

GGTGGTTTATTTGTTTTTATTAAAAGAAAAAGGGGGAGATGTTGGGAGTCGTTTTTATTT

TTGTCGTTATAAGATGGCGTTGATATCTTGTGTTTTAAGTGGTAAATAAATAATTTGCGT

ATGTGTTAAGGGTATTTTATGATTATTTGTGTTTTGTTTTTTTCGTGACGTTAATTCGGT

CGATGGGTTGTAGTTAATTAAGGAATGATACGTTTGAGGCGAAGGAGAATGTTTTTTAAG

AGGGACGGGGTTTTCGTTTTTTTTTTTTTTTGTTTTTTGTTTTTTTTTTTTGAAGATGTA

AGAATAAAGTTTTGTCGTAGAAGATTTTGGTTTGTGGTGTTTTTTTTGGTCGGTCGTGAG

AACGCGTCGAATAATATTAATGTTTTTATTTTTTTTTGATTTATGATTTAAAATTATTAT

TATTTTAATTATTTTAGAGTGATATTAGGAAATATAGATTTTTTTTTAT

>Pnp3LTR_37

AGAGTTGTAAGGTTAAGTATTGTAATGGAAAGGTTTTGCGGTATATATGAGTTTATTTTA

GGGAGATATGTTATTTTTTATGAAGGTTTAGTGTTTTAGTTTTTTTTTTTTAGGTAAAAC

GATACGGGAGTAGGTTAGGGTTGTTTTGGGTAAAAGTTTGTAAGTTTAAGAGTTAATTTT

GTATATGGTTTTTTTATCTATATATTGGGGATTTGATTTTTATTTTTATTTTTATTAATA

TGGGTGGTTTATTTGTTTTTATTAAAAGAAAAAGGGGGAGATGTTGGGAGTCGTTTTTAT

TTTTGTCGTTATAAGATGGCGTTGATATTTTGTGTTTTAAGTGGTAAATAAATAATTTGC

GTATGTGTTAAGGGTATTTTATGATTATTTGTGTTTTGTTTTTTTCGTGACGTTAATTCG

GTCGATGGGTTGTAGTTAATTAAGGAGTGATACGTTCGAGGCGAAGGAGAATGTTTTTTA

AGAGGGACGGGGTTTTCGTTTTTTTTTTTTTTGTTTTTTGTTTTTTTTTTTGAAGATGTA

AGAATAAAGTTTTGTCGTAGAAGATTTTGGTTTGTGGTGTTTTTTTTGGTCGGTCGTGAG

AACGCGTCGAATAATATTAATGTTTTTATTTTTTTTTGATTTATGATTTAAAATTATTGT

TATTTTAATTATTTTAGAGTGATATTAGGAAATATAGATTTTTTTTTAT

>Pnp3LTR_41

AGAGTTGTAAGGTTAAGTATTGTAATGGAAAGGTTTTGCGGTATATATGAGTTTATTTTA

GGGAGATATGTTATTTTTTATGAAGGTTTAGTGTTTTAGTTTTTTTTTTTTAGGTAAAAC

GATACGGGAGTAGGTTAGGGTTGTTTTGGGTAAAAGTTTGTAAGTTTAAGAGTTAATTTT

GTATATGGTTTTTTTATTTATATATTGGGGATTTGATTTTTATTTTTATTTTTATTAATA

TGGGTGGCTTATTTGTTTTTATTAAAGGAAAAAGGGGGAGATGTTGGGAGTCGTTTTTAT

TTTTGTCGTTATAAGATGGCGTTGATATTTTGTGTTTTAAGTGGTAAATAAATAATTTGC

GTATGTGTTAAGGGTATTTTATGATTATTTGTGTTTTGTTTTTTTCGTGACGTTAATTCG

GTCGATGGGTTGTAGTTAATTAAGGAGTGATACGTTCGAGGCGAAGGAGAATGTTTTTTA

AGAGGGACGGGGTTTTCGTTTTTTTTTTTTTTGTTTTTTGTTTTTTTTTTTTGAAGATGT

AAGAATAAAGTTTTGTCGTAGAAGATTTTGGTTTGTGGTGTTTTTTTTGGTCGGTCGTGA

GAACGCGTCGAATAATATTAATGTTTTTATTTTTTTTTGATTTATGATTTAAAATTATTA

TTATTTTAATTATTTTAGAGTGATATTAGGAAATATAGATTTTTTTTTAT

>Pnp3LTR_42

AGAGTTGTAAGGTTAAGTATTGTAATGGAAAGGTTATGCGGTATATATGAGTTTATTTTA

GGGAGATATGTTATTTTTTATGAAGGTTTAGTGTTTTAGTTTTTTTTTCAGGTAAAACGA

TACGGGAGTAGGTTAGGGTTGTTTTGGGTAAAAGTTTGTAAGTTTAAGAGTTAATTTTGT

ATATGGTTTTTTTATTTATATATTGGGGATTTGATTTTTATTTTTATTTTTATTAATATG

GGTGGTTTATTTGTTTTTATTAAAAGAAAAAGGGGGAGATGTTGGGAGTCGTTTTTATTT

TTGTCGTTATAAGATGGCGTTGATATTTTGTGTTTTAAGTGGTAAATAAATAATTTGCGT

ATGTGTTAAGGGTATTTTATGATTATTTGTGTTTTGTTTTTTTCGTGACGTTAATTCGGT

CGATGGGTTGTAGTTAATTAAGGAGTGATACGTTCGAGGCGAAGGAGAATGTTTTTTAAG

AGGGACGGGGTTTTCGTTTTTTTTTTTTCTGTTTTTTGTTTTTTTTTTTGAAGATGTAAG

AATAAAGTTTTGTCGTAGAAGATTTTGGTTTGTGGTGTTTTTTTTGGTCGGTCGTGAGAA

CGCGTCGAATAATATTAATGTTTTTATTTTTTTTTGATTTATGATTTAAAATTATTATTA

TTTTAATTATTTTAGAGTGATATTAGGAAATATAGATTTTTTTTTAT

>Pnp3LTR_44

TAAGTATTGTAATGGAAAGGTTTTGCGGTATATATGAGTTTATTTTAGGGAGATATGTTA

TTTTTTATGAAGGTTTAGTGTTTTAGTTTTTTTTTTTTAGGTAAAACGATACGGGAGTAG

GTTAGGGTTGTCTTGGGTAAAAGTTTGTAAGTTTAAGAGTTAATTTTGTATATGGTTTTT

TTATTTATATATTGGGGATTTGATTTTTATTTTTATTTTTATTAATATGGGTGGTTTATT

TGTTTTTATTAAAAGAAAAAGGGGGAGATGTTGGGAGTCGTTTTTATTTTCGTCGTTATA

AGATGGCGTTGATATTTTGTGTTTTAAGTGGTAAATAAATAATTTGCGTATGTGTTAAGG

GTATTTTATGATTATTTGTGTTTTGTTTTTTTCGTGACGTTAATTCGGTCGGTGGGTTGT

AGTTAATTAAGGAGTGATACGTTCGAGGCGAAGGAGAATGTTTTTTAAGAGGGATGGGGT

TTTCGTTTTTTTTTTTTTGTTTTTTGTTTTTTTTTTTTGAAGATGTAAGAATAAAGTTTT

GTCGTAGAAGATTTTGGTTTGTGGTGTTTTTTTTGGTCGGTCGTGAGAACGCGTCGAATA

ATATTAATGTTTTTATTTTTTTTTGATTTATGATTTAAAATTATTATTATTTTAATTATT

TTAGAGTGATATTAGGAAATATAGATTTTTTTTTAT

>Pnp3LTR_45

AGAGTTGTAAGGTTAAGTATTGTAATGGAAAGGTTTGCGGTATATATGAGTTTATTTTAG

GGAGATATGTTATTTTTTATGAAGGTTTAGTGTTTTAGTTTTTTTTTTTAGGTAAAATGA

TACGGGAGTAGGTTAGGGTTGTTTTGGGTAAAAGTTTGTAAGTTTAAGAGTTAATTTTGT

ATATGGTTTTTTTATTTATATATTGGGGATTTGATTTTTATTTTTATTTTTATTAATATG

GGTGGTTTATTTGTTTTTATTAAAAGAAAAAGGGGGAGATGTTGGGAGTCGTTTTTATAT

TCGTCGTTATAAGATGGCGTTGACATTTTGTGTTTTAAGTGGTAAATAAATAATTTGCGT

ATGTGTTAAGGGTATTTTATGATTATTTGTGTTTTGTTTTTTTCGTGACGTTAATTCGGT

CGATGGGTTGTAGTTAATTAAGGAGTGATACGTTCGAGGTGAAGGAGAATGTTTTTTAAG

AGGGACGGGGTTTTCGTTTTTTTTTTTTTTGTTTTTTGTTTTTTTTTTTTTGAAGATGTA

AGAATAAAGTTTTGTCGTAGAAGATTTTGGTTTGTGGTGTTTTTTTTGGTCGGTCGTGAG

AACGCGTCGAATAATATTAATGTTTTTATTTTTTTTTGATTTATGATTTAAAATTATTAT

TATTTTAATTATTTTAGAGTGATATTAGGAAATATAGATTTTTTTTTAT

>Pnp3LTR_46

AGAGTTGTAAGGTTAAGTATTGTAATGGAAAGGTTTGCGGTATATATGAGTTTATTTTAG

GGAGATATGTTATTTTTTATGAAGGTTTAGTGTTTTAGTTTTTTTTTTAGGTAAAACGAT

ACGGGAGTAGGTTAGGGTTGTTTTGGGTAAAAGTTTGTAAGTTTAAGAGTTAATTTTGTA

TATGGTTTTTTTATTTATATATTGGGGATTTGATTTTTATTTTTATTTTTATTAATATGG

GTGGTTTATTTGTTTTTATTAAAAGAAAAAGGGGGAGATGTTGGGAGTCGTTTTTATTTT

CGTCGTTATAAGATGGCGTTGATATTTTGTGTTTTAAGTGGTAAATAAATAATTTGCGTA

TGTGTTAAGGGTATTTTATGATTATTTGTGTTTTGTTTTTTTCGTGACGTTAATTCGGTC

GATGGGTTGTAGTTAATTAAGGAGTGATACGTTCGAGGCGAAGGAGAATGTTTTTTAAGA

GGGACGGGGTTTTCGTTTTTTTTTTTTGTTTTTTGTTTTTTTTTTGAAGATGTAAGAATA

AAGTTTTGTCGTAGAAGATTTTGGTTTGTGGTGTTTTTTTTGGTCGGTCGTGAGAACGCG

TCGAATAATATTAATGTTTTTGTTTTTTTTTGATTTATGATTCAAAATTATTATTATTTT

AATTATTTTAGAGTGATATTAGGAAATATAGATTTTTTTTTAT

>Pnp3LTR_89

AGAGTTGTAAGGTTAAGTATTGTAATGGAAAGGTTTGCGGTATATATGAGTTTATTTTAG

GGAGATATGTTATTTTTTATGAAGGTTTAGTGTTTTAGTTTTTTTTTTTAGGTAAAACGA

TACGGGAGTAGGTTAGGGTTGTTTTGGGTAAAAGTTTGTAAGTTTAAGAGTTAATTTTGT

ATATGGTTTTTTTATTTATATATTGGGGATTTGATTTTTATTTTTATTTTTATTAATATG

GGTGGTTTATTTGTTTTTATTAAAAGAAAAAGGGGGAGATGTTGGGAGTCGTTTTTATTT

TCGTCGTTATAAGATGGCGTTGATATTTTGTGTTTTAAGTGGTAAATAAATAATTTGCGT

ATGTGTTAAGGGTATTTTATGATTATTTGTGTTTTGTTTTTTTTGTGACGTTAATTCGGT

CGATGGGTTGTAGTTAATTAAGGAGTGATACGTTCGAGGCGAAGGAGAATGTTTTTTAAG

AGGGACGGGGTTTTCGTTTTTTTTTTTTTTTGTTTTTTGTTTCTTTTTTTTGAAGATGTA

AGAATAAAGTTTTGTCGTAGAAGATTTTGGTTTGTGGTGTTTTTTTGGTCGGTCGTGAGA

ACGCGTCGAATAACATTAATGTTTTTATTTTTTTTGATTTATGATTTAAAATTATTATTA

TTTTAATTATTTTAGAGTGATATTAGGAAATATAGATTTTTTTTTAT

%Me-CpG

87%

91.3%

91.3%

91.3%

95.7%

95.7%

95.7%

95.7%

95.7%

95.7%

100.0%

100.0%
