# Supplementary material for: Epigenetic interplay between mouse endogenous retroviruses and host genes
Source: Genome Biol. 2012 Oct 3;13(10):R89. doi: 10.1186/gb-2012-13-10-r89 (PMC3491417; doi:10.1186/gb-2012-13-10-r89)
Supplement: Additional file 4 — All bisulfite sequencing data. Compilation of all bisulfite sequences. [file gb-2012-13-10-r89-S4.zip › IAP10279_TE_thymus.doc]

Chr7 Non Polymorphic IAP

5’ LTR Miniprep Sequences B6 Thymus

>5LTRChr7_83

GATAAAATTTGTTTTGAATAAAATTTTAATTTTTTTTAGTTTTTATAAATGTAAAATTTT

TTATGTGTTGGGAGTCGTTTTTATTTTCGTCGTTATAAGATGGCGTTGATAGTTGTGTTT

TAAGTGGTAAATAAATAATTTGCGTATGTGTTAAGGGTATTTTATGATTATTTGTGTTTT

GTTTTTTTCGTGACGTTAATTCGGTCGATGGGTTGTAGTTAATTAAGGAGTGATACGTTC

GAGGCGAAGGAGAATGTTTTTTAAGAGGGACGGGGTTTTCGTTTTTTTTTTTTTTGTTTT

TTGTTTTTTTGTTTTTTGTTTTTTTGTTTTTTGTTTTTTTTTTTTGAAGATGTAAGAATA

AAGTTTTGTCGTAGAAGATTTTGGTTTGTGGTGTTTTTTTTGGTCGGTCGTGAGAACGCG

TTTAATAATAATTGGTGTCGAATTTCGGGACGAGAAAATTCGGGATGAGAAAAAATTTCG

GATTGGCGTAGGAGGGATATTTTATTTTAGAATTAGAATT

>5LTRChr7_84

GATAAAATTTGTTTTGAATAAAATTTTAATTTTTTTTAGTTTTTATAAATGTAAAATTTT

TTATGTGTTGGGAGTCGTTTTTATTTTCGTCGTTATAAGATGGCGTTGATAGTTGTGTTC

TAAGTGGTAAATAAATAATCTGCGTATGTGTTAAGGGTATTTTATGATTATTTGTGTTTT

GTTTTTTTCGTGACGTTAATTCGGTCGATGGGTTGTAGTTAATTAAGGAGTGATACGTTC

GAGGCGAAGGAGAATGTTTTTTAAGAGGGACGGGGTTTTCGTTTTTTTTTTTTTTGTTTT

TTGTTTTTTTGTTTTTTGTTTTTTTGTTTTTTGTTTTTTTTTTTTGAAGGTGTAAGAATA

AAGTTTTGTCGTAGAAGATTTTGGTTTGTGGTGTTTTTTTTGGTCGGTCGTGAGAACGCG

TTTAATAATAATTGGTGTCGAATTTCGGGACGAGAAAATTCGGGACGAGAAAAAATTTCG

GATTGGCGTAGGAGGGATATTTTATTTTAGAATTAGAATT

>5LTRChr7_85

GATAAAATTTGTTTTGAATAAAATTTTAATTTTTTTTAGTTTTTATAAATGTAAAATTTT

TTATGTGTTGGGGGTCGTTTTTATATTCGTCGTTATAAGATGGCGTTGATAGTTGTGTTT

TAAGTGGTAAATAAATAATTTGCGTATGTGTTAAGGGTATTTTATGATTATTTGTGTTTT

GTTTTTCTCGTGACGTTAATTCGGTCGATGGGTTGTAGTTAATTAAGGAGTGATACGTTC

GAGGCGAAGGAGAATGTTTTTTAAGAGGGACGGGGTTTTCGTTTTTTTTTTTGTTTTTTG

TTTTTTTGTTTTTTGTTTTTTTGTTTTTTGTTTTTTTTTTTTGAAGATGTAAGAATAAAG

TTTTGTCGTAGAAGATTTTGGTTTGTGGTGTTTTTTTTGGTCGGTCGTGAGAACGCGTTT

AATAATAATTGGTGTCGAATTTCGGGACGAGAAAATTCGGGACGAGGAAAAATTTCGGAT

TGGCGTAGGAGGGATATTTTATTTTAGAATTAGAATT

>5LTRChr7_86

GATAAAATTTGTTTTGAATAAAATTTTAATTTTTTTTAGTTTTTATAAATGTAAAATTTT

TTATGTGTTGGGAGTCGTTTTTATATTCGTCGTTATAAGATGGCGTTGATAGTTGTGTTT

TAAGTGGTAAATAAATAATTTGTGTATGTGTTAAGGGTATTTTATGATTATTTGTGTTTT

GTTTTTTTCGTGACGTTAATTCGGTCGATGGGTTGTAGTTAATTAAGGAGTGATACGTTC

GAGGCGAAGGAGAATGTTTTTTAAGAGGGACGGGGTTTTCGTTTTTTTTTTTTTGTTTTT

TGTTTTTTTGTTTTTTGTTTTTTTGTTTTTTGTTTTTTTTTTTGAAGATGTAAGAATAAA

GTTTTGTCGTAGAAGATTTTGGTTTGTGGTGTTTTTTTTGGTCGGTCGTGAGAACGCGTT

TAATAATAATTGGTGTCGAATTTCGGGACGGGAAAATTCGGGACGAGAAAAAATTTCGGA

TTGGCGTAGGAGGGATATTTTATTTTAGAATTAGAATT

>5LTRChr7_87

GATAAAATTTGTTTTGAATAAAATTTTAATTTTTTTTAGTTTTTATAAATGTAAAATTTT

TTATGTGTTGGGGGTCGTTTTTATATTCGTCGTTATAAGATGGCGTTGATAGTTGTGTTT

TAAGTGGTAAATAAATAATTTGCGTATGTGTTAAGGGTATTTTATGATTATTTGTGTTTT

GTTTTTCTCGTGACGTTAATTCGGTCGATGGGTTGTAGTTAATTAAGGAGTGATACGTTC

GAGGCGAAGGAGAATGTTTTTTAAGAGGGACGGGGTTTTCGTTTTTTTTTTTGTTTTTTG

TTTTTTTGTTTTTTGTTTTTTTGTTTTTTGTTTTTTTTTTTTGAAGATGTAAGAATAAAG

TTTTGTCGTAGAAGATTTTGGTTTGTGGTGTTTTTTTTGGTCGGTCGTGAGAACGCGTTT

AATAATAATTGGTGTCGAATTTCGGGACGAGAAAATTCGGGACGAGGAAAAATTTCGGAT

TGGCGTAGGAGGGATATTTTATTTTAGAATTAGAATT

>5LTRChr7_88

GATAAAATTTGTTTTGAATAAAATTTTAATTTTTTTTAGTTTTTATAAATGTAAAATTTT

TTATGTGTTGGGAGTCGTTTTTATATTCGTCGTTATAAGATGGCGTTGATAGTTGTGTTT

TAAGTGGTAAATAAATAATTTGCGTATGTGTTAAGGGTATTTTATGATTATTTGTGTTTT

GTTTTTTTCGTGACGTTAATTCGGTCGATGGGTTGTAGTTAATTAAGGAGTGATACGTTC

GAGGCGAAGGAGAATGTTTTTTAAGAGGGACGGGGTTTTCGTTTTTTTTTTTTTGTTTTT

TGTTTTTTTGTTTTTTGTTTTTTTGTTTTTTGTTTTTTTTTTTGAAGATGTAAGAATAAA

GTTTTGTCGTAGAAGATTTTGGTTTGTGGTGTTTTTTTTGGTCGGTCGTGAGAACGCGTT

TAATAATAATTGGTGTCGAATTTCGGGACGAGAAAATTCGGGACGAGAAAAAATTTCGGA

TTGGCGTAGGAGGGATATTTTATTTTAGAATTAGAATT

>5LTRChr7_89

GATAAAATTTGTTTTGAATAAAATTTTAATTTTTTTTAGTTTTTATAAATGTAAAATTTT

TTATGTGTTGGGAGTCGTTTTTATATTCGTCGTTATAAGATGGCGTTGATAGTTGTGTTT

TAAGTGGTAAATAAATAATTTGCGTATGTGTTAAGGGTATTTTATGATTATTTGTGTTTT

GTTTTTTTCGTGACGTTAATTCGGTCGATGGGTTGTAGTTAATTAAGGAGTGATACGTTC

GAGGCGAAGGAGAATGTTTTTTAAGAGGGACGGGGTTTTCGTTTTTTTTTTTTTTTGTTT

TTTGTTTTTTTGTTTTTTGTTTTTTTGTTTTTTGTTTTTTTTTTTGAAGATGTAAGAATA

AAGTTTTGTCGTAGAAGATTTTGGTTTGTGGTGTTTTTTTTGGTCGGTCGTGAGAACGCG

TTTAATAATAATTGGTGTCGAATTTCGGGACGAGAAAATTCGGGACGAGAAAAAATTTCG

GATTGGCGCAGGAGGGATATTTTATTTTAGAATTAGAATT

>5LTRChr7_90

GATAAAATTTGTTTTGAATAAAATTTTAATTTTTTTTAGTTTTTATAAATGTAAAATTTT

TTATGTGTTGGGAGTCGTTTTTATATTCGTCGTTATAAGATGGCGTTGATAGTTGTGTTT

TAAGTGGTAAATAAATAATTTGCGTATGTGTTAAGGGTATTTTATGATTATTTGTGTTTT

GTTTTTTTCGTGACGTTAATTCGGTCGATGGGTTGTAGTTAATTAAGGAGTGATACGTTC

GAGGCGAAGGAGAATGTTTTTTAAGAGGGACGGGGTTTTCGTTTTTTTTTTTTTTTGTTT

TTTGTTTTTTTGTTTTTTGTTTTTTTGTTTTTTGTTTTTTTTTTTTGAAGATGTAAGAAT

AAAGTTTTGTCGTAGAAGATTTTGGTTTGTGGTGTTTTTTTTGGTCGGTCGTGAGAACGC

GTTTAATAATAATTGGTGTCGAATTTCGGGACGAGAAAATTCGGGACGAGAAAAAATTTC

GGATTGGCGTAGGAGGGATATTTTATTTTAGAATT

>5LTRChr7_91

GATAAAATTTGTTTTGAATAAAATTTTAATTTTTTTTAGTTTTTATAAATGTAAAATTTT

TTATGTGTTGGGAGTCGTTTTTATATTCGTCGTTATAAGATGGCGTTGATAGTTGTGTTT

TAAGTGGTAAATAAATAATCTGCGTATGTGCTAAGGGTATTTTATGATTATTTGTGTTTT

GTTTTTTTCGTGACGTTAATTCGGTCGATGGGTTGTAGTTAATTAAGGAGTGACACGTTC

GAGGCGAAGGAGAATGTTTTTTAAGAGGGACGGGGTTTTCGTTTTTTTTTTTTTTGTTTT

TTGTTTTTTTGTTTTTTGTTTTTTTGTTTTTTGTTTTTTTTTTTTTGAAGATGTAAGAAT

AAAGTTTTGTCGTAGAAGATTTTGGTTTGTGGTGTTTTTTTTGGTCGGTCGTGAGAACGC

GTTTAATAATAATTGGTGTCGAATTTCGGGACGAGAAAATTCGGGACGAGAAAAAATTTC

GGATTGGTGTAGGAGGGATATTTTATTTTAGAATTAGAATT

>5LTRChr7_93

GATAAAATTTGTTTTGAATAAAATTTTAATTTTTTTTAGTTTTTATAAATGTAAAATTTT

TTATGTGTTGGGAGTCGTTTTTATATTCGTCGTTATAAGATGGCGTTGATAGTTGTGTTT

TAAGTGGTAAATAAATAATTTGCGTATGTGTTAAGGGTATTTTATGATTATTTGTGTTTT

GTTTTTTTCGTGACGTTAATTCGGTCGATGGGTTGTAGTTAATTAAGGAGTGATACGTTC

GAGGCGAAGGAGAATGTTTTTTAAGAGGGACGGGGTTTTCGTTTTTTTTTTTTTGTTTTT

TGTTTTTTTGTTTTTTGTTTTTTTGTTTTTTGTTTTTTTTTTTTGAAGATGTAAGAATAA

AGTTTTGTCGTAGAAGATTTTGGCTTGTGGTGTTTTTTTTGGTCGGTCGTGAGAACGCGT

TTAATAATAATTGGTGTCGAATTTCGGGACGAGAAAATTCGGGACGAGAAAAAATTTCGG

ATTGGCATAGGAGGGATATTTTATTTTAGAATTAGATT

>5LTRChr7_1

GATAAAATTTGTTTTGAATAAAATTTTAATTTTTTTTAGTTTTTATAAATGTAAAATTTT

TTATGTGTTGGGAGTCGTTTTTATATTCGTCGTTATAAGATGGCGTTGATAGTTGTGTTT

TAAGTGGTAAATAAATAATTTGCGTATGTGTTAAGGGTATTTTATGATTATTTGTGTTTT

GTTTTTTTCGTGACGTTAATTCGGTCGATGGGTTGTAGTTAATTAAGGAGTGATACGTTC

GAGGCGAAGGAGAATGTTTTTTAAGAGGGACGGGGTTTTCGTTTTTTTTTTTTTTGTTTT

TTGTTTTTTTGTTTTTTGTTTTTTTGTTTTTTGTTTTTTTTTTTTGAAGATGTAAGAATA

AAGTTTTGTCGTAGAAGATTTTGGTTTGTGGTGTTTTTTTTGGTCGGTCGTGAGAACGCG

TTTAATAATAATTGGTGTCGAATTTCGGGACGAGAAAATTCGGGACGAGAAAAAATTTCG

GATTGGCGTAGGAGGGATATTTTATTTTAGAATTAGAATT

>5LTRChr7_2

GATAAAATTTGTTTTGAATAAAATTTTAATTTTTTTTAGTTTTTATAAATGTAAAATTTT

TTATGTGTTGGGAGTCGTTTTTATATTCGTCGTTATAAGATGGCGTTGATAGTTGTGTTT

TAAGTGGTAAATAAATAATTTGCGTATGTGTTAAGGGTATTTTATGATTATTTGTGTTTT

GTTTTTTTCGTGACGTTAATTCGGTCGATGGGTTGTAGTTAATTAAGGAGTGATACGTTC

GAGGCGAAGGAGAATGTTTTTTAAGAGGGACGGGGTTTTCGTTTTTTTTTTTTTTTGTTT

TTTGTTTTTTTGTTTTTTGTTCTTTTGTTTTTTGTTTTTTTTTTTGAAGATGTAAGAATA

AAGTTTTGTCGTAGAAGATTTTGGTTTGTGGTGTTTTTTTTGGTCGGTCGTGAGAACGCG

TTTAATAATAATTGGTGTCGAATTTCGGGACGAGAAAATTCGGGACGAGAAAAAATTTCG

GATTGGTGTAGGAGGGATATTTTATTTTAGAATTAGATT
